# α-Tocopherol and Trolox as Effective Natural Additives for Polyurethane Foams: A DFT and Experimental Study

**DOI:** 10.3390/molecules29246037

**Published:** 2024-12-21

**Authors:** Dalal K. Thbayh, Dóra Mentes, Zsanett R. Boros, Marcin Palusiak, László Farkas, Béla Viskolcz, Béla Fiser

**Affiliations:** 1 Institute of Chemistry, University of Miskolc, 3515 Miskolc-Egyetemváros, Hungary; kemdalal@uni-miskolc.hu (D.K.T.); bela.viskolcz@uni-miskolc.hu (B.V.); 2 Polymer Research Center, University of Basrah, Basrah 61004, Iraq; 3 Higher Education and Industrial Cooperation Centre, University of Miskolc, 3515 Miskolc-Egyetemváros, Hungary; dora.mentes@uni-miskolc.hu; 4 Wanhua-BorsodChem Zrt, Bolyai tér 1., 3700 Kazincbarcika, Hungary; renata.boros@borsodchem.eu (Z.R.B.); laszlo.farkas@borsodchem.eu (L.F.); 5 Department of Physical Chemistry, Faculty of Chemistry, University of Lodz, 90-236 Lodz, Poland; marcin.palusiak@chemia.uni.lodz.pl; 6 Department of Biology and Chemistry, Ferenc Rakoczi II Transcarpathian Hungarian College of Higher Education, 90200 Beregszász, Ukraine

**Keywords:** DFT, bond dissociation enthalpy, HAT, ionization potential, vitamin E, FPUF

## Abstract

In this work, α-tocopherol and trolox were studied as compounds that have high biological activity. α-Tocopherol is considered a food additive because the refining process of vegetable oils causes the depletion of this vitamin, and thus, its inclusion is required to keep them from oxidizing. Computational tools have determined the antioxidant activity of these additives. The geometries of the studied molecules were optimized using two density functional methods, including M05-2X and M06-2X, in combination with the 6-311++G(2d,2p) basis set. The results indicated that when comparing the antioxidant activity of α-tocopherol and trolox, they were very similar to each other, but α-tocopherol had an antioxidant activity slightly higher, around 1.2 kJ/mol, than trolox. Thus, these additives can be used as polymer additives to protect materials from free-radical-induced stress. To test their applicability in polymeric formulations, flexible polyurethane foams were prepared with varying α-tocopherol ratios and NCO indices (1.0 and 1.1). Increasing the α-tocopherol content reduced the compressive force and altered the mechanical properties, likely due to its presence in the foam structure. This additive not only fine-tuned the mechanical properties but also provided antioxidant effects, enabling multiple enhancements in polymeric products with a single additive.

## 1. Introduction

Free radicals are produced not only as a result of normal metabolic processes in the human body (endogenous sources) but also as a result of environmental variables (exogenous sources), including ozone radiation, stress, industrial chemicals, pesticides, and pollution [1,2,3,4,5]. There are three categories of plant-based antioxidants: phenolic chemicals, vitamins, and carotenoids [6]. The molecules of phenolic compounds range from simple structures such as ferulic acid, vanillin, gallic acid, caffeic acid, and butylated hydroxytoluene to polyphenols like tannins and flavonoids [7].

**Figure 1:**
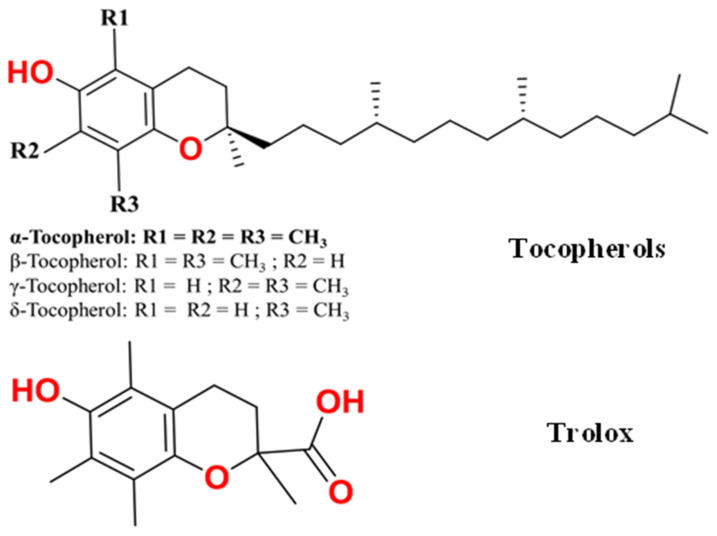
Two-dimensional chemical structure for the types of tocopherols and trolox.

Many researchers have recently studied synthetic antioxidants and have used them in a variety of applications due to their availability and decreased production costs. Nevertheless, there are some health hazards associated with synthetic antioxidants because they are petroleum-based compounds, and many of them cause environmental problems after their emission [7]. Researchers have recently begun looking into natural or bio-based antioxidants as substitute additives in an effort to reduce environmental pollution [8,9]. The compounds derived from plants often have substantial phenolic contents that can be used as antioxidants, like tocotrienols, tocopherols, chlorophylls, polyphenols, ascorbates, and carotenoids [10]. Tocotrienols have a similar chemical structure to tocopherols, but the difference between them is that the former have an aliphatic chain with three double bonds [11]. Tocotrienols are present in extracts rich in tocopherols (α-tocopherol, β-tocopherol, γ-tocopherol, and δ-tocopherol) [12]. Tocopherols are considered one of the most important, safe, and harmless groups of additives [13]. Vitamin E is a mixture of four tocopherols (alpha, beta, gamma, and delta) and four corresponding tocotrienols. Vitamin E activity is required for life because it protects human cells from oxidation and assists in the absorption of vitamin K. These compounds are naturally found in many foodstuffs, mainly fruits, oils, fats, and vegetables [14]. Tocopherols are classified into several categories based on the number and location of methyl groups bound to their benzene unit. α-Tocopherol has three methyl groups bound to the aromatic ring, while in β- and γ-tocopherol, there are two, and δ-tocopherol has only one (Figure 1) [12,13].

Trolox (6-hydroxy-2,5,7,8-tetramethylchroman-2-carboxylic acid) is a water-soluble tocopherol derivative that has numerous medicinal and industrial applications (Figure 1). Trolox is also capable of decreasing oxidation; therefore, it would be worth considering using it as an antioxidant additive to prevent oxidation and free radical intracellular formation [15,16]. Trolox is well known for its antioxidant action in a variety of systems and under varied circumstances, and it works well as a food preservative. It has the ability to prevent the loss of serotonin and dopamine receptor binding sites in animal brain membranes as well as the radical chain oxidation of proteins and enzymes [17]. Trolox has a chromanol structure that contributes to its antioxidant activity, as well as a carboxyl group, which influences its water solubility [15,17]. Although trolox and α-tocopherol are both important antioxidants and have been extensively studied with polymers, there is still a need for further understanding of their modes of action and their applicability in polymeric formulations [18,19].

Polymers and plastics frequently have added antioxidants to prevent or slow down polymer degradation due to oxidation processes. This aids in the preservation of the material’s physical and mechanical qualities over time [20]. One of the degradation processes that stabilizers try to combat is oxidation, which is why antioxidants might be considered a subgroup of stabilizers. One thing they all have in common is that antioxidants can act as stabilizers, particularly when preventing oxidation-induced degradation. In this sense, antioxidants are a type of stabilizer that specifically address the oxidative degradation of materials. Some stabilizers may have antioxidant properties, and some antioxidants may offer stability against factors beyond oxidation [21].

Polyurethanes (PUs) are a significant group of polymers that are widely used in various industries worldwide. The hydroxyl groups (-OH) of a polyol react with isocyanate groups (-NCO) in isocyanates to form PU [22]. In this study, flexible polyurethane foams (FPUFs) were used as model systems. FPUFs are complex porous materials that are widely used in various comfort applications, such as automotive seats and other types of cushioning [23].

The present study focused on the antioxidant potential of natural compounds, including α-tocopherol and trolox. The antioxidant characteristics of these additives were determined and compared using computational tools. Then, to test the applicability of these additives, FPUFs were synthesized, and they were studied with the aim of using a single additive material to achieve multiple effects in materials and objects made with this polymer.

## 2. Results and Discussion

### 2.1. Computational Results

#### 2.1.1. Optimized Geometries

**Figure 2:**
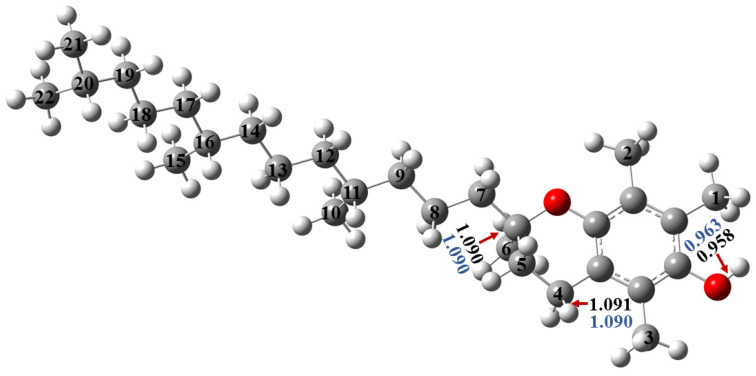
Three-dimensional structure of α-tocopherol optimized at the M06-2X/6-311++G(2d,2p) level of theory, and the appropriate bond lengths (in Å) for the strongest and weakest X-H (X=O, C) bonds are also displayed, and the gas-phase and water-phase results are indicated with black and blue, respectively.

In this work, the structures of α-tocopherol and trolox were optimized. The former has three methyl groups bound to the aromatic ring, and optimizations were carried out by using M05-2X (Figure S1) and M06-2X (Figure 2) in the gas and water phase. There is one hydroxyl group (OH) in α-tocopherol, and its corresponding bond length is equal to 0.958 Å in the gas phase and 0.963 Å in the water phase, whereas the C-H bonds cover a range between 1.084–1.097 Å. In the gas phase, the shortest carbon–hydrogen bond is C2-H, which is in the methyl group, whilst the longest are C11-H and C16-H, which are on the isoprenoid sidechain (Figure 2 and Table 1). In the solvent phase, there are little differences in the values for the same bonds (Figure 2 and Table 2). Both methods, M05-2X and M06-2X, provided similar results with minor changes in the values in all of the gas and water phases (Figure S1, Tables S1 and S2).

**Figure 3:**
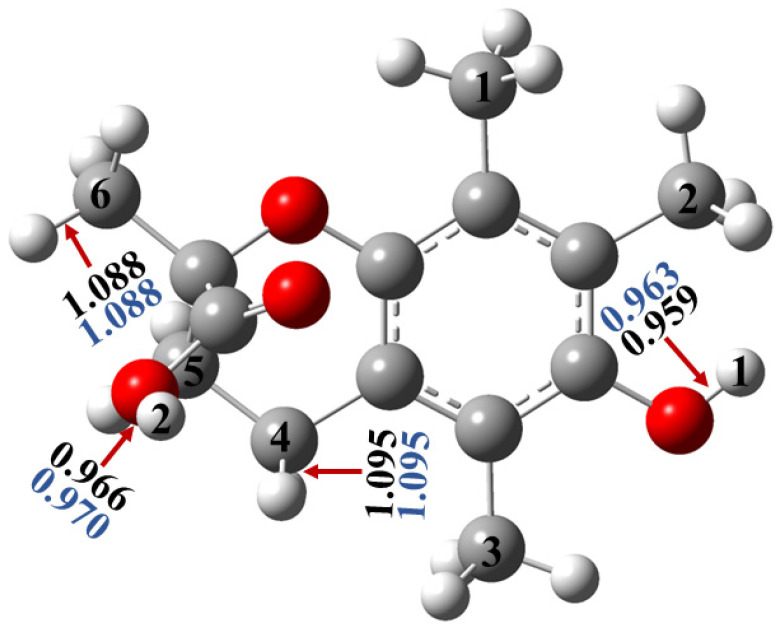
Three-dimensional structure of trolox optimized at the M06-2X/6-311++G(2d,2p) level of theory, and the appropriate bond lengths (in Å) for the strongest and weakest X-H (X=O, C) bond are also displayed, and the gas-phase and water-phase results are indicated with black and blue, respectively.

Trolox is a smaller tocopherol derivative. As for trolox, the structure has two hydroxyl groups (OH), and the corresponding optimized bond lengths are equal to 0.959 Å (O1-H) and 0.966 Å (O2-H) in the gas phase while equal to 0.963 Å (O1-H) and 0.970 Å (O2-H) in the water phase. The C-H bonds cover a range from 1.084 (C1-H) to 1.095 Å (C4-H) and from 1.085 (C1-H) to 1.095 Å (C4-H), as determined at the M06-2X/6-311++G(2d,2p) level of theory in the gas phase and water phase, respectively (Figure 3, Table 3 and Table 4). The shortest C-H bond is C1-H, and it is located on the methyl group, while the longest one is located on the heterocyclic ring. The behavior is similar to the M05-2X/6-311++G(2d,2p) level of theory, with only minor differences in the values experienced (Figure S2, Tables S3 and S4).

#### 2.1.2. Antioxidant Capability and Scavenging Mechanisms

The antioxidant efficacy of α-tocopherol and trolox was evaluated, and three mechanisms, including hydrogen atom transfer (HAT), sequential proton loss electron transfer (SPLET), and single electron transfer–proton transfer (SET-PT), in the gas and water phases were studied. The most important parameters from these mechanisms were calculated to compare the antioxidant ability of the studied molecules.

As for HAT, hydrogen atoms can be transferred from numerous hydrogen donor positions of the antioxidant additive, like the methyl group (CH_3_), hydroxyl group (OH), benzene ring, and others, to free radicals. Within the HAT mechanism, antioxidant compounds participate in a one-step process by donating a hydrogen atom, resulting in the formation of a radical. Simultaneously, the original free radical is neutralized. Thus, X-H bond dissociation enthalpy (BDE) values can be used to measure the antioxidant’s ability to donate a hydrogen atom. Although there are numerous descriptors that can demonstrate antioxidant activity, BDE is currently the most trustworthy and is often used when taking the HAT mechanism into account [24]. The lower the value of BDE, the better the antioxidant properties of a compound [16,25,26,27,28,29].

**Figure 4:**
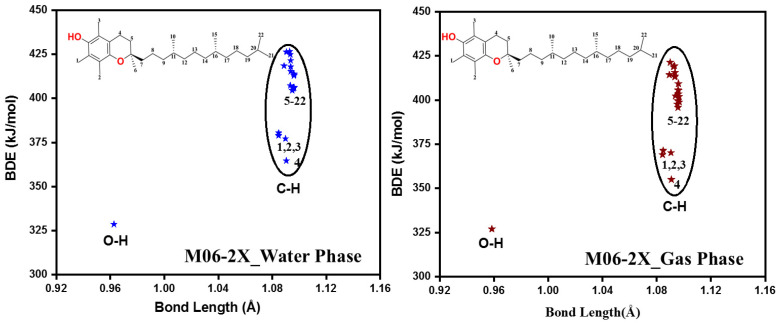
Bond dissociation enthalpy vs. bond length plot of α-tocopherol. The calculations were performed in the gas and water phases using the M06-2X/6-311++G(2d,2p) level of theory.

**Figure 5:**
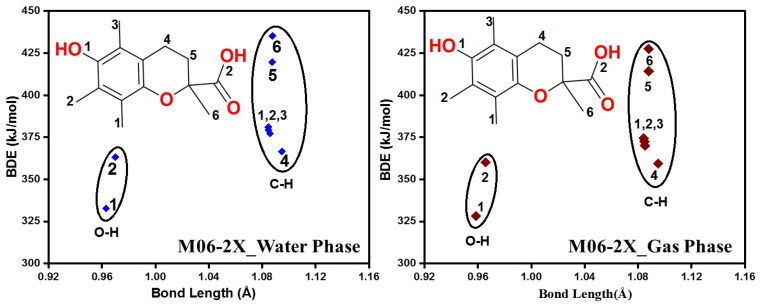
Bond dissociation enthalpy vs. bond length plot of trolox. The calculations were performed in the gas and water phases using the M06-2X/6-311++G(2d,2p) level of theory.

The values of BDE for all the unique X-H bonds of α-tocopherol were computed in the gas and water phases by using two methods, including M05-2X and M06-2X (Table 3, Table 4, Tables S3 and S4, Figure 5 and Figure S3). As for M062X in the two phases, α-tocopherol has one hydroxyl group, and the corresponding BDE value is equal to 327.0 and 328.6 kJ/mol in the gas and water phases, respectively, which is in excellent agreement with experimental [30,31,32,33] and theoretical studies [34,35]. The BDEs of the C-H bonds in the molecule cover a range from 355.0 to 421.3 kJ/mol in the gas phase and from 364.6 to 426.3 kJ/mol in the water phase. The weakest C-H bond is C4-H, and it is located on the heterocyclic ring, while the strongest one is C6-H, located on the methyl group (Figure 4 and Figure S3). The O-H bond has the highest antioxidant potential, but in some cases, C-H bonds can serve as hydrogen donor sites and contribute to the antioxidant effect of the compound. The behavior is similar to M05-2X/6-311++G(2d,2p), with only minor shifts in the values experienced (Figure S3, Tables S1 and S2).

The values of BDE for all the possible sites of trolox were also studied (Table 3, Table 4, Tables S3 and S4, Figure 5 and Figure S4). The results indicated that in the case of trolox, the two hydroxyl groups O1-H and O2-H are quite far from each other in terms of their antioxidant activity because their BDEs are 328.2 and 360.2 kJ/mol in the gas phase and 332.8 and 363.2 kJ/mol in the water phase. The C-H bonds cover a range between 359.4 and 427.5 kJ/mol (Table 3) and 366.6 and 435.2 kJ/mol (Table 4) in gas and water phases, respectively. The smallest one belongs to C4-H, which is located on the heterocyclic ring, and the strongest one is C6-H, which is located on a methyl group (Figure 5). It has to be noted that the same behavior was experienced in both studied species, which was expected due to their structural similarities. There is no difference in the BDE values according to M05-2X/6-311++G(2d,2p) (Figure S4, Tables S3 and S4).

The lowest value of BDE belongs to the OH group of the phenol ring of trolox, which is in good agreement with previous studies [16,36].

The experimental values of the BDEs for the regularly used polymers were collected from the literature and compared with the smallest BDEs of trolox and α-tocopherol [25,26]. We noticed that the BDE values for commonly used polymers are in a range of 393.7 and 406.2 kJ/mol [37], and in both the studied species, there is at least one X-H bond that has a smaller BDE than these. Thus, both trolox and α-tocopherol can be potentially applied as antioxidant additives to protect polymers from free radicals.

Single electron transfer–proton transfer (SET-PT) has two steps to neutralize radicals. In the first step, an electron from the antioxidant molecule is transferred, which is followed by a proton transfer. The ionization potential (IP) and proton dissociation enthalpy (PDE) are used in this process to determine the antioxidant potential of the species. The antioxidant capacity of the compounds increases with decreasing IPs and PDE values [16,25,26,27,32,38].

IPs and PDE values for α-tocopherol were computed (Table 1, Table 2, Tables S1 and S2) with two methods and in two phases. As for M06-2X, the results indicate that the IP of α-tocopherol in the gas phase is higher than the value in the water phase, which is equal to 662.7 kJ/mol in the gas phase and equal to 423.6 kJ/mol in the water phase (Table 1 and Table 2). In the gas phase and water phases, the PDE of the O-H bond is equal to 972.9 and 63.7 kJ/mol, respectively. These values are similar to those found in previous studies [32,35]. The PDE values of C-H bonds cover a range from 1006.1 (C4-H) to 1069.7 kJ/mol (C6-H) in the gas phase and from 100.6 to 162.4 kJ/mol in the water phase with M06-2X (Table 1 and Table 2). In the case of the second method (M05-2X), the behavior was the same with a slight change in the values (Tables S1 and S2). From the results, we can see that the O-H group is a more potent proton donor in the second step of the SET-PT mechanism of tocopherol than the C-H bonds, which is expected. Consequently, O-H has a higher antioxidant activity than C-H bonds depending on their IP + PDE values. The results corresponding to the O-H and C-H bonds for α-tocopherol in both methods agree with the HAT results.

In the case of trolox, the corresponding IP and PDE values were determined (Table 3, Table 4, Tables S3 and S4) using both M05-2X and M06-2X. In M06-2X, it was found that the IP is slightly higher (677.2 kJ/mol) in the gas phase and in the water phase (436.2 kJ/mol) compared to α-tocopherol (Table 3 and Table 4). The PDE values of the O-H bonds are equal to 961.9 and 991.4 kJ/mol in the gas phase, while they are equal to 56.2 and 86.6 kJ/mol in the water phase, where the lowest bond is the O1-H bond, and the strongest one is O2-H (Table 1 and Table 2, Figure 3). However, for the C-H bonds, the PDEs are in a range from 993.3 to 1058.5 kJ/mol in the gas phase, and they are in a range from 90.0 to 158.6 kJ/mol in the water phase in the M06-2X, where the weakest one refers to the C4-H bond, located on the heterocyclic ring, while the strongest one is C6-H, which is located on the methyl group (Table 1 and Table 2, Figure 3), and this is in good agreement with previous studies [16]. Moreover, the IP+ PDE results show that the O-H activity is higher than the C-H activity in both M06-2X and M05-2X (Tables S3 and S4). The IP results for α-tocopherol and trolox indicate that the former donates an electron more easily than the latter. All in all, by comparing the lowest values of IP + PDE of trolox and α-tocopherol, the latter has a slightly higher antioxidant activity than the former, which is in good agreement with the HAT mechanism.

Sequential proton loss electron transfer (SPLET) is the third antioxidant mechanism considered and calculated to compare the antioxidant potential of the studied species. This process is another two-step mechanism, which includes a proton transfer followed by an electron transfer from the antioxidant to the radicals. There are two important parameters that determine the antioxidant ability in this case: proton affinity (PA) and electron transfer enthalpy (ETE). The smaller the PA and ETE, the better the antioxidant ability is the studied molecule. The calculated values of PA and ETE for all the possible sites of X-H (X=O or C) bonds of α-tocopherol were collected and compared in two phases by using two methods (Table 1, Table 2, Tables S1 and S2). The O-H bond has the smallest PA + ETE value within the molecule, 1635.6 and 486.5 kJ/mol in the gas phase and water phase, respectively, and this is in good agreement with previous studies [32,35]. The values of PA +ETE of the C-H bonds cover a range between 1668.8 and 1732.7 kJ/mol in the gas phase, while in the water phase, they cover a range between 521.6 to 586.1 (Table 3 and Table 4). The weakest C-H bond was located on the heterocyclic ring, while the strongest one was located on the methyl group. As for trolox, the PA and ETE values of the O-H and C-H bonds were also computed (Table 3, Table 4, Tables S3 and S4).

**Figure 6:**
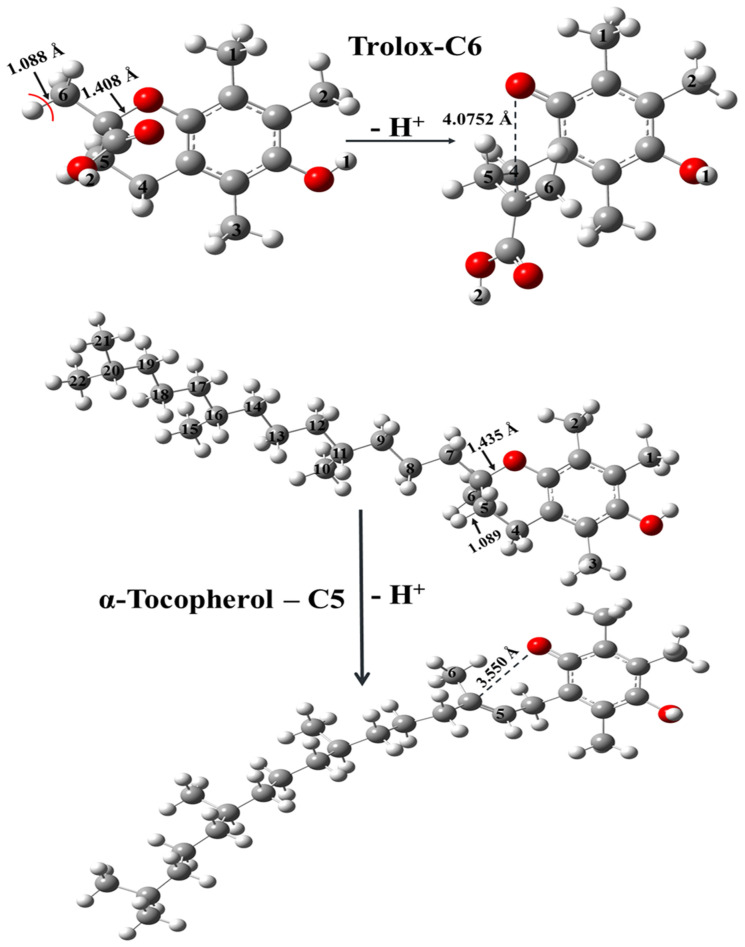
Single-proton-loss (SPL) step of the SPLET mechanism in the case of the C5-H bond of α-tocopherol and the C6-H bond of trolox. Intramolecular rearrangement occurs in both cases. The species were computed at the M06-2X/6-311++G(2d,2p) level of theory in the gas phase, and the corresponding bond lengths (in Å) are also shown.

There is a special site of both trolox (C6-H) and α-tocopherol (C5-H), which, during the corresponding anion formation in the SPL step of the SPLET mechanism, will lead to intramolecular rearrangement (Figure 6). Thus, the SPLET was not comparable with other cases. The proton loss from the C5-H of α-tocopherol and C6-H of trolox initiate a successive C-O bond break, similar to the results of other studies [21].

All in all, the antioxidant capability of O-H bonds is bigger than that of C-H bonds for both α-tocopherol and trolox, but at the same time, there are some C-H bonds, such as C4-H, which may contribute to the antioxidant capability of the species. These results are in good agreement across all mechanisms. If the antioxidant potential of α-tocopherol and trolox is compared, they approximately have the same values, but still, α-tocopherol has higher antioxidant activity.

#### 2.1.3. Quantum Theory of Atoms in Molecules (QTAIM)

Because in the case of more classic methods of estimating antioxidant efficiency based on the energy effects of bond dissociation models, like HAT and others, the final numerical results are to some extent dependent on the model itself, we additionally analyzed the electron density parameters of the X-H bonds in the molecular closed-shell ground states according to the QTAIM approach. In Tables S5 and S6, there is a collection of data obtained using this approach. We can see that the BCP values are very similar when comparing them within the group of the given bond type. Thus, in the case of the C-H bonds, the values are in the range between 0.276 and 0.286 of a.u. for both tocopherol and trolox, which is a very narrow span of values. The same can be observed in the case of O-H bonds; thus, almost the same values of the electron density in the O-H BCPs were obtained, with these being 0.374 a.u. for hydroxyl O-H and 0.364 a.u. for carboxylic O-H bonds.

Assuming that, according to QTAIM, the electron density at the bond critical point is in direct relation with the given bond strength, one may conclude that the C-H and O-H bond strengths in isolated and unperturbed molecules of tocopherol and trolox are very similar, and there is no clear indication of which bonds are the most easily dissociating ones. Therefore, it can be expected that the thermodynamic properties of these molecular systems are not the only factors directly responsible for their antioxidant properties, but the thermodynamic properties of the dissociated mediums or, more generally, the kinetic properties of these two chemical species are also factors.

### 2.2. Experimental Results

#### Mechanical Tests

Adding antioxidant additives to FPUF foam structures can influence their mechanical properties like the compression force (F). The BDE of antioxidants influences the oxidative stability of PU materials, which, in turn, affects their compression force deflection (CFD). By selecting antioxidants with an appropriate BDE, oxidative degradation can be minimized, preserving the mechanical properties of PU, including its ability to resist compression. Therefore, this study incorporated α-tocopherol, a natural antioxidant additive, into a FPUF foam structure. Compression force deflection tests were carried out according to ASTM D3574 test C on different FPUF samples prepared by using additives of 0.0, 0.25, 0.50, 0.75, and 1.0 wt% and an NCO index 1.0 and 1.1. The force was measured at 50% of the original sample height compression after 1 min.

**Figure 7:**
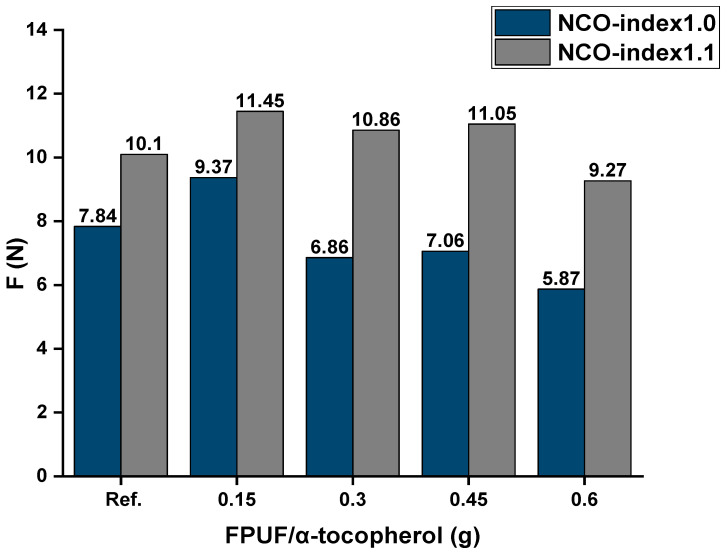
Compression force (F) deflection of FPUF samples with different ratios of α-tocopherol of 0.0 (Ref), 0.25, 0.50, 0.75, 1.0 wt %. Ref: foam without α-tocopherol.

**Figure 8:**
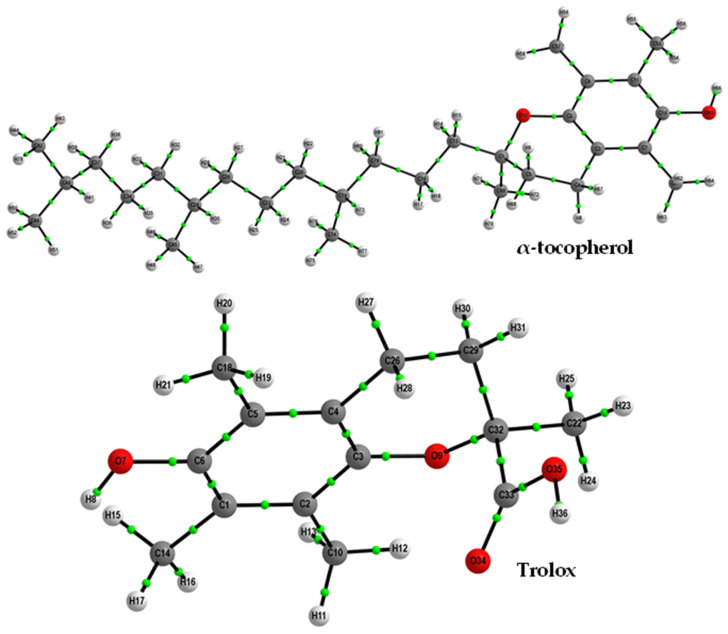
Optimized structures of α-tocopherol and trolox were computed at the M06-2X/6-311++G (2d,2p) level of theory in the gas phase, and bond critical points were determined.

The compression force was recorded for each NCO index (Figure 8). The results show that increasing the concentration of α-tocopherol in the foams led to a decrease in the compression force, meaning the foam became softer or less resistant to compression (Table 5, Figure 7). The compressive force (F) value was 10.10 N (for reference, 0.0% wt α-tocopherol) and 9.27 N for the FPUF that contained 1.0% wt additive. This means that the foams with a high concentration of additive were softer than the reference. This effect was generally due to the interaction of α-tocopherol with the polymer matrix and the flexibility it introduced. An antioxidant additive like α-tocopherol, which has a relatively low molecular weight, can act as a plasticizer in the polymer matrix [39]. As a plasticizer, it increases the flexibility of the polymer chains, leading to greater mobility within the matrix. This increased molecular mobility reduces the resistance of the foam to compressive forces, making it softer and decreasing its compression force.

As the hardness of the foams increased with the NCO index, the compressive force (F) gradually increased from 7.84 N for an NCO index of 1.0 to 10.10 N in the case of the samples with a 1.1 NCO index (Table 5). The compressive force of the samples with an NCO index of 1.0 with different amounts of additive ranged from 7.84 N to 5.87 N for 0.0 and 1.0 wt %, respectively. The change in the foam heights was very small, <1%.

## 3. Computational Details

### 3.1. Methods

The Gaussian 09 program was used to carry out the calculations [40]. The structures of the studied species, α-tocopherol, trolox, and their corresponding species (radicals, radical cations, and anions), were optimized by using two density functional methods, M05-2X [41] and M06-2X [42], along with the 6-311++G(2d,2p) basis set in the gas and water phase. The solvent effect of water was approximated by using the Solvation Model based on Density (SMD). The applicability of these methods has been verified before [25,26,29,43,44,45,46,47]. The experimental value of the bond dissociation enthalpy (BDE) for a specific O–H bond determined in the case of α-tocopherol was used to validate the calculations and to select the most suitable combination of method and basis set for the subsequent analysis and discussion [30,31,32,33].

To gain an understanding of the bonding between the atoms within the molecules under investigation, we employed the study of electron density based on the Quantum Theory of Atoms in Molecules (QTAIM) [48,49]. For fully optimized molecular geometries, we used AIMAll (Version 15.05. 18) software package [50]. To achieve this objective, we utilized the Gaussian 09 software to calculate the electron density of specific molecular equilibrium geometries. We employed a wave function format with the M06-2X/6-311++G(2d,2p) method. A graphical representation of the molecules, including the all-atoms-numbering scheme, can be found in Figure 8.

### 3.2. Antioxidant Mechanism

The antioxidant activity of the examined geometries was calculated using three alternative mechanisms, including hydrogen atom transfer (HAT), sequential proton loss electron transfer (SPLET), and single electron transfer followed by proton transfer (SET-PT), as detailed in our previous papers [25,26,51].

## 4. Experimental Details

### 4.1. Preparation of Flexible Polyurethane Foam and α-Tocopherol as Additives (FPUF/α-Tocopherol)

Flexible polyurethane foam samples were synthesized by using Ongronat TR4040 (a mixture of methylene-diphenyl-diisocyanate isomers from Wanhua-BorsodChem in Kazincbarcika, Hungary) and Ongropur FFP-303 (a polyether-type polyol premix, also from Wanhua-BorsodChem in Kazincbarcika, Hungary). In order to produce the applied polyol, catalysts and supplementary additives, including blowing agents and surfactants, were combined into a base polyol (Alcupol F2831, Repsol, Madrid, Spain). FPUF samples with different isocyanate indexes and, thus, different hardness was prepared. Samples with 1.0 and 1.1 isocyanate indexes were prepared (Table 6). The isocyanate index is the ratio of the total number of NCO groups to the sum of the OH groups of the polyol premix, which includes additives (e.g., water) within the reaction mixture. The hardness of the samples increased with increasing the isocyanate index due to the greater proportion of hard segments. Different ratios, 0.0, 0.25, 0.5, 0.75, and 1.0% wt, of α-tocopherol were added to the polyol (Table 7). Thus, α-tocopherol was used as an additive for the FPUF, and the samples’ mechanical properties were tested. Cylinder-shaped samples were cut out from each product with a diameter of 30 mm and a total sample height of approximately 35 mm, and these were used for the mechanical tests.

### 4.2. Mechanical Measurements

The mechanical properties of the prepared samples (FPUF/α-tocopherol) were studied by using the mechanical testing method C of ASTM D3574 (complex test standard for flexible polyurethanes) [39]. Compression force deflection (CFD) was measured by pressing the samples on their entire surface up to 50% of their height, holding for 1 min, and then measuring the change in force.

## 5. Conclusions

Computational tools were employed to investigate the radical scavenging activities of α-tocopherol and trolox via their different X-H sites. Three different mechanisms were considered to determine the antioxidant capability of the molecules. All unique positions were studied, and the corresponding thermodynamic parameters, including BDE, IP, PDE, PA, and ETE, were computed. It was discovered that O-H bonds have a higher antioxidant potential than C-H bonds for both α-tocopherol and trolox. The antioxidant activity of α-tocopherol is slightly higher, around 1.2 kJ/mol, than that of trolox. The BDE values of the additives were compared to those of synthetic polymers, which range in value from 393.7 to 406.2 kJ/mol, and it was discovered that there is at least one X-H bond that has a smaller BDE than these conventional polymers. To test their applicability, flexible polyurethane foam samples were prepared with different ratios of α-tocopherol and two NCO indices (1.0 and 1.1). As the ratio of α-tocopherol in the foams increased, the mechanical properties of the FPUF changed, and the compressive force decreased. The presence of the additives in the foam structure likely caused this. Thus, the mechanical properties of the foams were fine-tuned by using α-tocopherol, which could also express antioxidant effects. Thus, by using one additive, multiple improvements in the properties of the polymeric products can be achieved.

## Figures and Tables

**Table 1:** Bond lengths (B.L.) (in Å) and bond dissociation enthalpy (BDE), ionization potential (IP), proton dissociation enthalpy (PDE), proton affinity (PA), and electron transfer enthalpy (ETE) values (in kJ/mol) of α-tocopherol computed using the M06-2X/6-311++G(2d,2p) level of theory in the gas phase.

Compound	B.L.	BDE	IP	PDE	IP + PDE	PA	ETE	PA + ETE
α-Tocopherol			662.7					
O-H	0.958	327.0		972.9	1635.6	1461.4	174.2	1635.6
C1-H	1.085	371.4		1020.6	1683.3	1564.5	118.0	1682.5
C2-H	1.084	369.1		1019.8	1682.5	1583.4	99.1	1682.5
C3-H	1.090	370.2		1018.6	1681.3	1585.5	95.8	1681.3
C4-H	1.091	355.0		1006.1	1668.8	1569.1	99.6	1668.8
C5-H *	1.089	414.3		- *	- *	- *	- *	- *
C6-H	1.090	421.3		1069.7	1732.4	1674.3	58.1	1732.4
C7-H	1.093	413.6		1059.3	1722.0	1675.2	49.5	1724.7
C8-H	1.094	402.4		1049.9	1712.6	1668.0	46.3	1714.3
C9-H	1.096	405.6		1054.5	1717.2	1685.7	31.5	1717.2
C10-H	1.093	413.2		1062.6	1725.3	1686.8	38.4	1725.3
C11-H	1.097	398.9		1047.3	1710.0	1673.0	36.5	1709.5
C12-H	1.096	409.2		1057.6	1720.4	1686.5	28.6	1715.1
C13-H	1.096	395.8		1046.2	1709.0	1691.7	15.2	1706.9
C14-H	1.096	405.5		1053.3	1716.0	1700.8	20.8	1721.7
C15-H	1.093	415.6		1064.0	1726.7	1694.0	31.5	1725.5
C16-H	1.097	402.0		1050.4	1713.1	1683.8	29.4	1713.2
C17-H	1.096	403.4		1051.8	1714.5	1693.7	20.2	1713.8
C18-H	1.096	399.6		1047.9	1710.6	1696.7	13.8	1710.5
C19-H	1.096	409.3		1053.6	1716.3	1700.3	15.9	1716.2
C20-H	1.095	397.7		1046.1	1708.8	1697.7	11.2	1708.9
C21-H	1.092	418.8		1067.1	1729.8	1713.9	15.9	1729.9
C22-H	1.093	419.4		1068.3	1731.0	1707.0	25.6	1732.7

* Rearrangement after deprotonation.

**Table 2:** Bond lengths (B.L.) (in Å), bond dissociation enthalpy (BDE), ionization potential (IP), proton dissociation enthalpy (PDE), proton affinity (PA), and electron transfer enthalpy (ETE) values in kJ/mol of α-tocopherol computed using the M06-2X/6-311++G(2d,2p) level of theory in combination with the SMD solvent model.

Compound	B.L	BDE	IP	PDE	IP + PDE	PA	ETE	PA + ETE
α-Tocopherol			423.6					
O-H	0.963	328.6		63.7	487.3	174.1	312.5	486.5
C1-H	1.085	380.4		110.6	534.2	310.8	229.0	539.8
C2-H	1.085	378.9		114.6	538.2	319.7	218.5	538.2
C3-H	1.090	377.1		113.5	537.1	319.8	216.8	536.7
C4-H	1.090	364.6		100.6	524.2	323.8	197.8	521.6
C5-H	1.089	418.5		149.5	573.1	383.6	191.7	575.3
C6-H	1.090	426.3		162.2	585.9	380.7	205.1	585.9
C7-H	1.094	415.3		151.0	574.6	397.3	177.6	574.9
C8-H	1.093	407.3		140.6	564.3	406.0	160.6	566.6
C9-H	1.096	413.9		149.4	573.1	405.9	167.5	573.4
C10-H	1.094	421.4		157.4	581.0	390.6	187.9	578.5
C11-H	1.096	406.3		142.2	565.8	411.7	148.4	560.1
C12-H	1.096	412.9		148.7	572.3	407.3	165.0	572.3
C13-H	1.095	405.5		141.4	565.0	409.7	155.3	565.0
C14-H	1.096	413.1		149.0	572.6	407.7	164.9	572.6
C15-H	1.094	418.0		156.7	580.3	393.8	183.8	577.5
C16-H	1.096	406.1		142.0	565.7	412.3	153.2	565.5
C17-H	1.096	413.0		148.8	572.4	404.5	168.0	572.5
C18-H	1.096	406.0		142.1	565.7	409.9	155.5	565.4
C19-H	1.096	413.7		149.5	573.2	409.0	164.3	573.2
C20-H	1.095	404.5		140.3	564.0	410.2	153.8	564.0
C21-H	1.093	426.5		162.4	586.1	397.3	188.8	586.1
C22-H	1.093	424.9		160.8	584.4	394.8	189.4	584.2

**Table 3:** Bond lengths (B.L) (in Å), bond dissociation enthalpy (BDE), ionization potential (IP), proton dissociation enthalpy (PDE), proton affinity (PA), and electron transfer enthalpy (ETE) values in kJ/mol of trolox in the gas phase computed using the M06-2X/6-311++G(2d,2p) level of theory.

Compound	B.L	BDE	IP	PDE	IP + PDE	PA	ETE	PA + ETE
Trolox			677.2					
O1-H	0.959	328.2		961.9	1639.1	1460.5	178.8	1639.3
O2-H	0.966	360.2		991.4	1668.6	1406.0	262.4	1668.4
C1-H	1.084	374.5		1007.4	1684.6	1588.0	97.6	1685.6
C2-H	1.085	372.5		1006.5	1683.7	1564.1	119.5	1683.6
C3-H	1.085	370.1		1003.9	1681.2	1584.6	96.5	1681.2
C4-H	1.095	359.4		993.3	1670.5	1566.7	103.8	1670.5
C5-H	1.088	414.3		1048.2	1725.4	1625.2	100.2	1725.4
C6-H*	1.088	427.5		- *	- *	- *	- *	- *

* Rearrangement after deprotonation.

**Table 4:** Bond lengths (B.L.) (in Å), bond dissociation enthalpy (BDE), ionization potential (IP), proton dissociation enthalpy (PDE), proton affinity (PA), and electron transfer enthalpy (ETE) values in kJ/mol of trolox computed using the M06-2X/6-311++G(2d,2p) level of theory in combination with the SMD solvent model.

Compound	B.L.	BDE	IP	PDE	IP + PDE	PA	ETE	PA + ETE
Trolox			436.2					
O1-H	0.963	332.8		56.2	492.3	171.8	320.6	492.3
O2-H	0.970	363.2		86.6	522.8	102.1	420.6	522.8
C1-H	1.085	379.4		103.6	539.8	315.8	222.9	538.8
C2-H	1.085	381.1		104.4	540.5	309.9	230.7	540.6
C3-H	1.086	377.2		100.6	536.8	315.8	221.0	536.8
C4-H	1.095	366.6		90.0	526.2	318.0	208.1	526.2
C5-H	1.088	419.7		143.7	579.8	363.8	215.4	579.2
C6-H	1.088	435.2		158.6	594.8	346.1	248.6	594.7

**Table 5:** Compression force deflection test results for flexible polyurethane foam and α-tocopherol as an additive (FPUF/α-tocopherol); h is the sample height after the measurement of the F50% (N).

NCO Index = 1.0		
Percentage of α-Tocopherol (% wt)	F (N)	h (mm)
0	7.84	34.33
0.25	9.37	34.14
0.5	6.86	34.66
0.75	7.06	35.69
1	5.87	34.67
**NCO Index = 1.1**		
0	10.10	34.55
0.25	11.45	35.70
0.5	11.05	36.21
0.75	10.86	35.21
1	9.27	36.63

**Table 6:** Formulation of flexible polyurethane foam.

Polyol Premix Ongropur FFP-303		Ratio pphb	
Wanol F3160		100	
Water		3.6	
Alcupol F3231		1	
Tegostab B4113		0.5	
Tegoamin DEOA 85		0.5	
DABCO 33LV		0.15	
Jaffcat ZF-22		0.1	
**NCO Index**	**Polyol Premix Ongropur FFP-303 (%m/m)**		**Isocyanate Ongronat TR4040 (%m/m)**
1.0	63.61%		36.39%
1.1	59.98%		40.02%

**Table 7:** Formulation of flexible polyurethane foam and α-tocopherol as additives (FPUF/α-tocopherol).

		NCO-Index-1.0	
Percentage of α-Tocopherol (% wt)	Weight of α-Tocopherol [g]	Polyol Premix	Isocyanate
		Ongropur FFP-303 [g]	Ongronat TR4040 [g]
0	0	38.16	21.83
0.25	0.15	38.16	21.83
0.5	0.3	38.16	21.83
0.75	0.45	38.16	21.83
1	0.6	38.16	21.83
		**NCO-Index-1.1**	
**Percentage of α-Tocopherol (% wt)**	**Weight of α-Tocopherol** **[g]**	**Polyol Premix**	**Isocyanate**
		**Ongropur FFP-303** **[g]**	**Ongronat TR4040** **[g]**
0	0	35.98	24.01
0.25	0.15	35.98	24.01
0.5	0.3	35.98	24.01
0.75	0.45	35.98	24.01
1	0.6	35.98	24.01

## Data Availability

Data are provided within the manuscript and Supplementary Information files.

## Supplementary Materials

The following supporting information can be downloaded at: https://www.mdpi.com/article/10.3390/molecules29246037/s1, Figures S1 and S2: Optimized geometries of the studied natural antioxidant additives α-tocopherol (vitamin E) and trolox. Figures S3 and S4: Bond dissociation enthalpy (BDE) vs bond length plot for studied natural antioxidant additives α-tocopherol (vitamin E) and trolox; Tables S1–S4: Antioxidant properties of the studied species calculated at the M05-2X/6-311++G(2d,2p) level of theory in gas solvent (water) phases. Tables S5 and S6: Numerical data from QTAIM analysis for the studied α-tocopherol and Trolox, including electron density and their Laplacian, both estimated in C-H and O-H bond critical points and additionally, delocalization index values obtained for atoms forming corresponding bonds, all of them were calculated at the M06-2X/6-311++G (2d,2p) level of theory, cartesian coordinates of the optimized structures are given.
