# Supplementary material for: α-Tocopherol and Trolox as Effective Natural Additives for Polyurethane Foams: A DFT and Experimental Study
Source: Molecules. 2024 Dec 21;29(24):6037. doi: 10.3390/molecules29246037 (PMC11678614; doi:10.3390/molecules29246037)
Supplement: Supplementary file 1 [file molecules-29-06037-s001.zip › molecules-3361494-supplementary.pdf]

Supplementary Information

α-Tocopherol and Trolox as Effective Natural Additives for Polyurethane Foams: A DFT and Experimental Study

Dalal K. Thbayh [1,2,3], Dóra Mentes [3], Zsanett R. Boros [4], Marcin Palusiak [5], László Farkas [4], Béla Viskolcz [1,3] and Béla Fiser [1,5,6,*]

[1] Institute of Chemistry, University of Miskolc, 3515 Miskolc-Egyetemváros, Hungary; kemdalal@uni-miskolc.hu (D.K.T.); bela.viskolcz@uni-miskolc.hu (B.V.)
[2] Polymer Research Center, University of Basrah, Basrah 61004, Iraq
[3] Higher Education and Industrial Cooperation Centre, University of Miskolc, 3515 Miskolc-Egyetemváros, Hungary; dora.mentes@uni-miskolc.hu
[4] Wanhua-BorsodChem Zrt, Bolyai tér 1., 3700 Kazincbarcika, Hungary; renata.boros@borsodchem.eu (Z.R.B.); laszlo.farkas@borsodchem.eu (L.F.)
[5] Department of Physical Chemistry, Faculty of Chemistry, University of Lodz, 90-236 Lodz, Poland; marcin.palusiak@chemia.uni.lodz.pl
[6] Department of Biology and Chemistry, Ferenc Rakoczi II Transcarpathian Hungarian College of Higher Education, 90200 Beregszász, Ukraine
* Correspondence: bela.fiser@uni-miskolc.hu; Tel.: +36-46-565-111

List of abbreviations

HAT: hydrogen atom transfer

BDE: bond dissociation enthalpy

SET-PT: single electron transfer-proton transfer

PA: proton affinity

ETE: electron transfer enthalpy

SPLET: sequential proton loss electron transfer

IP: ionization potential

PDE: proton dissociation enthalpy

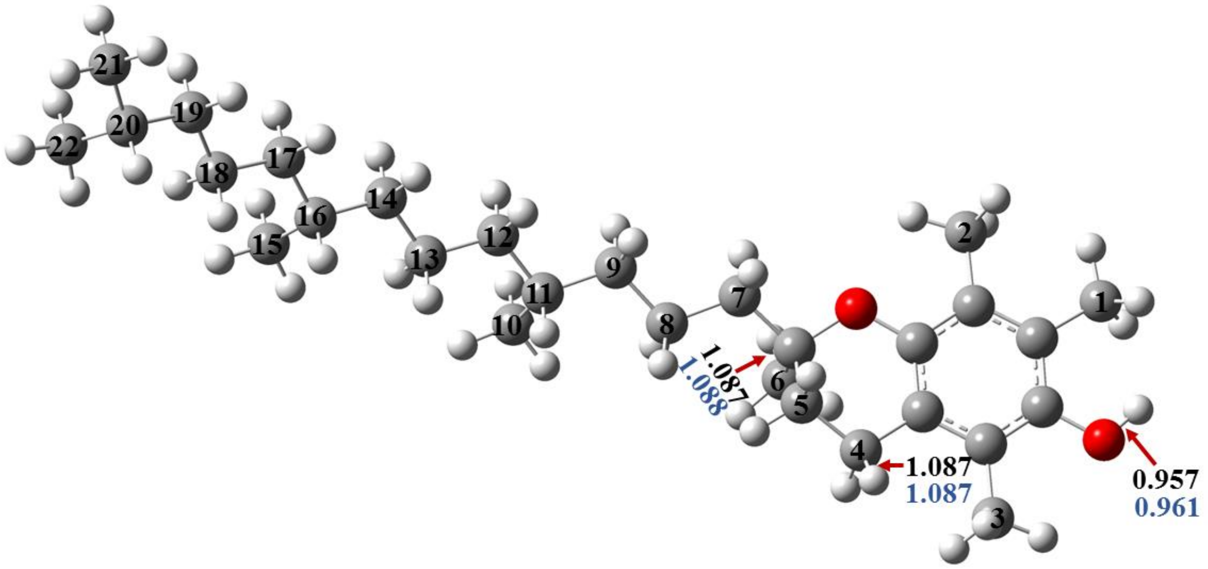

Figure S1. Optimized geometries of the studied natural antioxidant additives α-Tocopherol (vitamin E). Geometry optimizations have been carried out at the M05-2X/6-311++G(2d,2p) level of theory in gas and water phase, and the appropriate bond lengths (in Å) for the strongest and weakest X-H (X=O, C) bond are also displayed in the black (gas) and blue (water) phases.

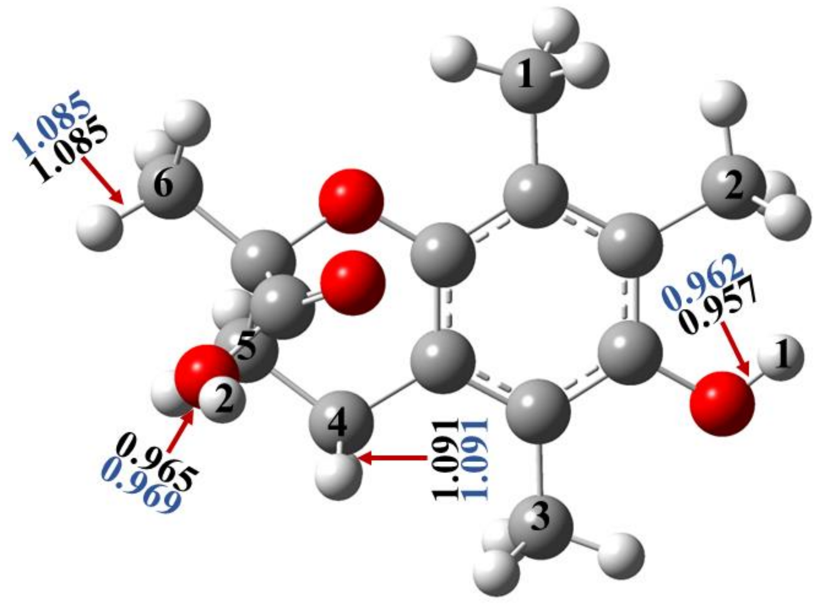

Figure S2. Optimized geometries of the studied natural antioxidant additives Trolox. Geometry optimizations have been carried out at the M05-2X/6-311++G(2d,2p) level of theory in gas and water phase, and the appropriate bond lengths (in Å) for the strongest and weakest X-H (X=O, C) bond are also displayed in the black (gas) and blue (water) phases.

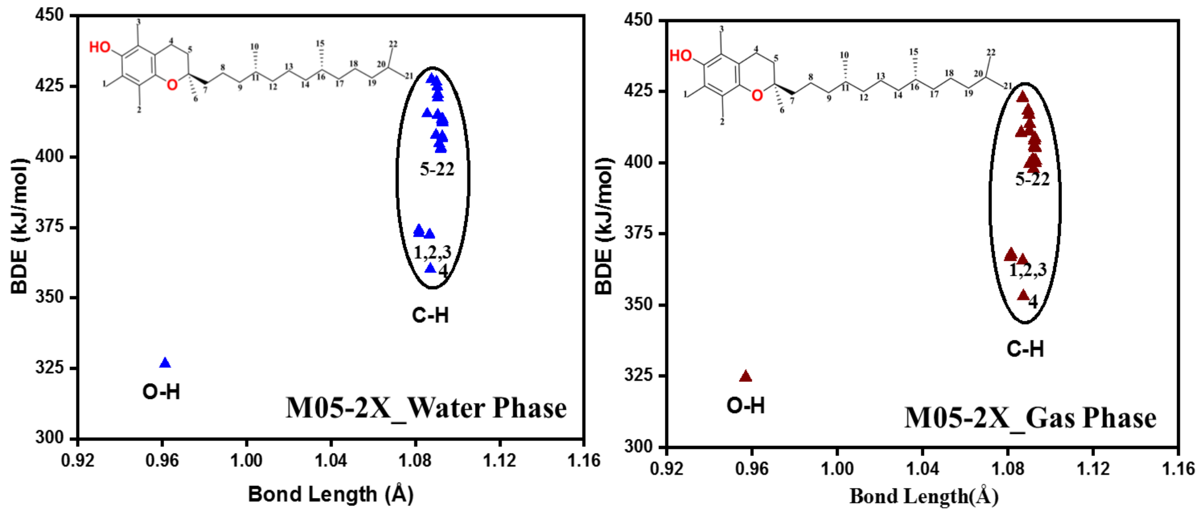

Figure S3. Bond dissociation enthalpy (BDE) vs bond length plot for studied natural antioxidant additive α-Tocopherol (vitamin E). The calculations have been carried out at the M05-2X/6-311++G(2d,2p) level of theory in the gas and water phases.

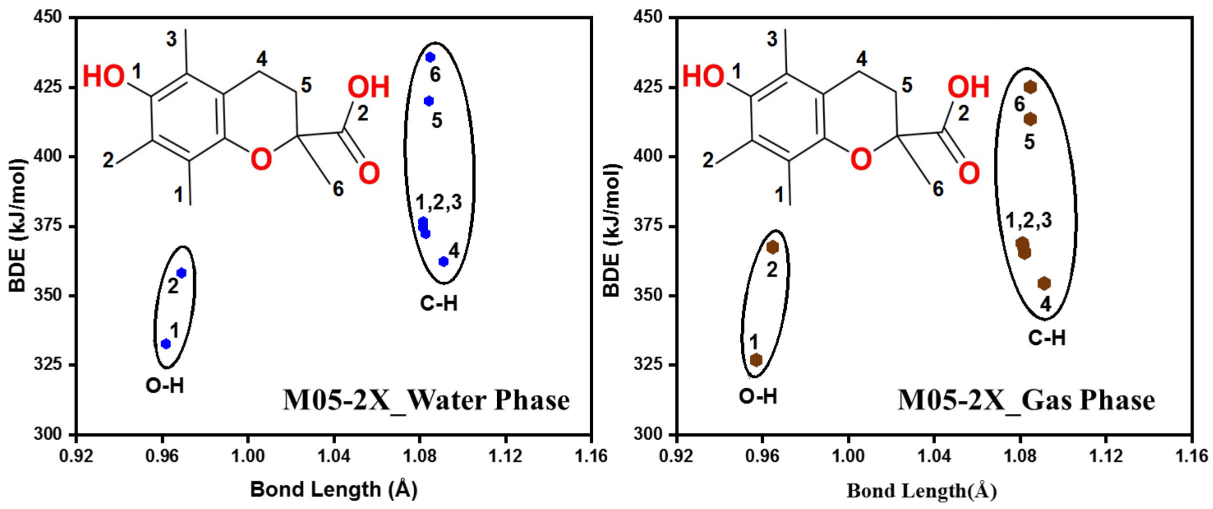

Figure S4. Bond dissociation enthalpy (BDE) vs bond length plot for studied natural antioxidant additive Trolox. The calculations have been carried out at the M05-2X/6-311++G(2d,2p) level of theory in the gas and water phases.

Table S1. Bond lengths (B.L) (in Å), Bond dissociation enthalpy (BDE), ionization potential (IP), proton dissociation enthalpy (PDE), proton affinities (PAs) and electron transfer enthalpies (ETE) values in kJ/mol for α-Tocopherol (vitamin E) calculated at the M05-2X/6-311++G(2d,2p) level of theory in gas phase.

Compound	B.L	BDE	IP	PDE	IP+PDE	PA	ETE	PA+ETE
**α-Tocopherol**			664.5					
O-H	0.957	324.7		974.1	1638.7	1460.7	178.0	1638.7
C1-H	1.082	367.9		1017.5	1682.1	1566.8	115.1	1681.8
C2-H	1.081	367.0		1016.5	1681.0	1591.4	89.8	1681.2

C3-H	1.087	365.6		1017.4	1681.9	1593.1	88.8	1681.9
C4-H	1.087	353.1		1002.5	1667.0	1576.6	90.4	1667.0
C5-H*	1.086	410.6						
C6-H	1.087	422.9		1072.3	1736.8	1680.2	58.9	1739.0
C7-H	1.090	411.0		1060.4	1724.9	1685.3	39.6	1724.9
C8-H	1.090	399.6		1048.9	1713.4	1680.4	33.0	1713.4
C9-H	1.092	405.9		1055.4	1719.9	1695.1	24.8	1719.9
C10-H	1.090	411.1		1060.5	1725.0	1696.8	28.3	1725.0
C11-H	1.093	400.8		1051.9	1716.4	1686.6	25.4	1712.0
C12-H	1.093	405.1		1051.8	1716.3	1697.1	24.4	1721.5
C13-H	1.092	400.9		1050.4	1714.9	1700.1	15.0	1715.1
C14-H	1.092	407.9		1057.4	1721.9	1700.8	20.8	1721.7
C15-H	1.090	413.7		1065.5	1730.1	1701.0	26.3	1727.3
C16-H	1.093	399.9		1049.8	1714.4	1697.0	18.1	1715.1
C17-H	1.093	408.7		1055.4	1720.0	1706.1	15.5	1721.6
C18-H	1.092	397.8		1047.3	1711.8	1707.4	5.2	1712.6
C19-H	1.093	405.6		1058.1	1722.6	1709.5	10.0	1719.6
C20-H	1.092	401.0		1050.1	1714.7	1708.4	6.3	1714.7
C21-H	1.090	418.4		1067.9	1732.4	1724.6	7.8	1732.4
C22-H	1.093	416.9		1066.4	1730.9	1711.1	19.8	1730.9

* Rearrangement after deprotonation

Table S2. Bond lengths (B.L) (in Å), Bond dissociation enthalpy (BDE), ionization potential (IP), proton dissociation enthalpy (PDE), proton affinities (PAs) and electron transfer enthalpies (ETE) values in kJ/mol for α-Tocopherol (vitamin E) calculated at the M05-2X/6-311++G(2d,2p) level of theory in solvent phase (water).

Compound	B.L	BDE	IP	PDE	IP+PDE	PA	ETE	PA+ETE
**α-Tocopherol**			438.7					
O-H	0.961	326.6		67.6	506.3	172.1	335.2	507.3
C1-H	1.082	373.0		110.9	549.6	318.2	235.9	554.1
C2-H	1.082	374.1		114.7	553.4	325.2	227.8	553.1
C3-H	1.087	372.5		112.9	551.6	325.0	226.2	551.2
C4-H	1.087	360.2		100.9	539.6	329.8	209.8	539.6
C5-H	1.086	415.3		156.7	595.4	388.1	206.6	594.7
C6-H	1.088	427.6		168.2	606.9	380.5	223.9	604.5
C7-H	1.091	415.0		155.8	594.5	402.8	189.5	592.3
C8-H	1.090	407.8		148.4	587.0	413.0	173.3	586.2
C9-H	1.093	413.3		153.9	592.6	417.2	175.4	592.7
C10-H	1.091	420.9		159.8	598.5	401.1	199.6	600.7
C11-H	1.093	406.6		147.4	586.1	421.8	164.2	585.9
C12-H	1.093	412.2		152.8	591.5	415.8	175.7	591.5
C13-H	1.092	402.6		143.2	581.9	418.7	163.0	581.7
C14-H	1.093	412.8		153.4	592.1	415.9	175.9	591.9
C15-H	1.091	422.4		158.9	597.6	401.6	195.9	597.6
C16-H	1.093	407.3		147.0	585.7	422.4	160.3	582.8
C17-H	1.093	412.8		153.5	592.2	416.0	175.8	591.8
C18-H	1.092	403.3		141.0	579.7	418.6	163.8	582.4
C19-H	1.093	413.5		154.2	592.9	417.9	174.6	592.5
C20-H	1.091	404.8		143.0	581.7	423.1	161.0	584.1
C21-H	1.090	426.7		167.3	606.0	405.2	200.8	606.0
C22-H	1.090	424.9		165.9	604.6	402.2	202.1	604.2

Table S3. Bond dissociation enthalpy (BDE), ionization potential (IP), proton dissociation enthalpy (PDE), proton affinities (PAs) and electron transfer enthalpies (ETE) values in kJ/mol for Trolox calculated at the M05-2X/6-311++G(2d,2p) level of theory in gas phase.

Compound	B.L	BDE	IP	PDE	IP+PDE	PA	ETE	PA+ETE
**Trolox**			674.7					
O1-H	0.957	326.9		966.9	1641.7	1461.2	179.7	1640.9
O2-H	0.965	367.5		1029.5	1704.2	1403.1	279.2	1682.3
C1-H	1.081	368.9		1008.1	1682.9	1592.6	90.3	1682.8
C2-H	1.082	367.9		1007.2	1682.0	1567.6	114.2	1681.9
C3-H	1.082	365.5		1004.7	1679.4	1588.7	90.7	1679.4
C4-H	1.091	354.5		993.7	1668.5	1570.6	97.9	1668.5
C5-H	1.085	413.6		1052.8	1727.6	1629.1	98.5	1727.6
C6-H*	1.085	425.1						

* Rearrangement after deprotonation

Table S4. Bond dissociation enthalpy (BDE), ionization potential (IP), proton dissociation enthalpy (PDE), proton affinities (PAs) and electron transfer enthalpies (ETE) values in kJ/mol for Trolox calculated at the M05-2X/6-311++G(2d,2p) level of theory in solvent phase (water).

Compound	B.L	BDE	IP	PDE	IP+PDE	PA	ETE	PA+ETE
**Trolox**			450.9					
O1-H	0.962	332.7		61.6	512.5	172.4	338.9	511.4
O2-H	0.969	358.2		86.2	537.1	100.7	436.4	537.1
C1-H	1.082	374.6		103.6	554.5	321.3	232.6	553.9
C2-H	1.082	376.6		105.0	555.9	315.8	240.0	555.9
C3-H	1.083	372.4		102.0	552.9	319.3	232.4	551.7
C4-H	1.091	362.3		90.7	541.6	321.7	220.0	541.6
C5-H	1.084	420.1		149.0	599.9	369.2	230.2	599.4
C6-H	1.085	435.8		165.3	616.1	349.5	265.6	615.1

Table S5. Contains numerical data from QTAIM analysis for the studied trolox including electron density rho and its laplacian. both estimated in C-H and O-H bond critical points and. additionally. Delocalisation index values obtained for atoms forming corresponding bonds. All of them calculated at the M06-2X/6-311++G(2d.2p) level of theory.

Atoms	Rho	DelSqRho	Ellipticity	K	BPL - GBL_I
O1-H	0,373646	-2,761325	0,021058	+0,765991	0,000467
O2-H	0,364443	-2,717715	0,015865	+0,749031	0,000572
C1-H*	0,278996	-0,996899	0,010831	+0,294738	0,000170
C1-H	0,286492	-1,052311	0,007459	+0,305616	0,000079
C1-H**	0,277534	-0,986861	0,011858	+0,292506	0,000133
C2-H*	0,276675	-0,978916	0,012115	+0,290451	0,000154

C2-H	0,284392	-1,036892	0,007437	+0,303419	0,000101
C2-H**	0,275741	-0,972938	0,012799	+0,289015	0,000136
C3-H**	0,276712	-0,981466	0,010709	+0,290972	0,000109
C3-H*	0,278787	-0,995882	0,009810	+0,294472	0,000155
C3-H	0,285343	-1,043941	0,007202	+0,303689	0,000072
C6-H	0,282085	-1,020952	0,007805	+0,298773	0,000123
C6-H*	0,281750	-1,017425	0,006763	+0,297499	0,000146
C6-H**	0,281328	-1,014547	0,005093	+0,297183	0,000211
C4-H*	0,281743	-1,014834	0,006385	+0,297946	0,000056
C4-H	0,278001	-0,986273	0,010446	+0,291178	0,000074
C5-H*	0,281693	-1,012161	0,004001	+0,297044	0,000177
C5-H	0,284010	-1,032245	0,002415	+0,301328	0,000109

H represents hydrogen atoms which were removed from the structure to study the antioxidant potential, while H* and H** represent the second and third hydrogen atoms bonding to the same C atom.

Table S6. Contains numerical data from QTAIM analysis for the studied α-Tocopherol including electron density rho and its laplacian. both estimated in C-H and O-H bond critical points and additionally. Delocalisation index values obtained for atoms forming corresponding bonds. All of them calculated at the M06-2X/6-311++G(2d.2p) level of theory.

Atoms	Rho	DelSqRho	Ellipticity	K	BPL - GBL_I
C5-H	0,281916	-1,017323	0,002464	+0,299593	0,000101
C4-H	0,281259	-1,010817	0,006105	+0,297391	0,000052
C4-H*	0,277985	-0,986163	0,008814	+0,291704	0,000063
C5-H*	0,280710	-1,005050	0,004323	+0,295819	0,000128
C7-H*	0,280446	-1,003046	0,002663	+0,295216	0,000186
C7-H	0,279863	-0,998614	0,001394	+0,294161	0,000183
C-H*	0,278969	-0,998000	0,006057	+0,295191	0,000125
C8-H*	0,281478	-1,011151	0,001413	+0,298740	0,000064
C8-H	0,278907	-0,993562	0,003709	+0,294315	0,000093
C11-H	0,278677	-0,989789	0,001588	+0,292862	0,000004
C9-H*	0,279335	-0,995447	0,004150	+0,294339	0,000128
C12-H	0,277121	-0,980541	0,002355	+0,290622	0,000122
C12-H*	0,279124	-0,994000	0,003936	+0,294125	0,000127
C13-H*	0,282114	-1,015743	0,000773	+0,300219	0,000056

C13-H	0,277169	-0,981691	0,003883	+0,291120	0,000113
C14-H*	0,279058	-0,993494	0,003968	+0,294026	0,000126
C14-H	0,277120	-0,980483	0,002595	+0,290645	0,000120
C18-H*	0,281726	-1,012985	0,000847	+0,299395	0,000058
C18-H	0,277173	-0,981762	0,003642	+0,291130	0,000110
C16-H	0,278736	-0,990391	0,001695	+0,292990	0,000004
C17-H	0,277154	-0,980630	0,002546	+0,290718	0,000121
C17-H*	0,279021	-0,993247	0,003939	+0,293972	0,000126
C19-H	0,276998	-0,979518	0,002730	+0,290433	0,000126
C19-H*	0,278959	-0,993099	0,003601	+0,293893	0,000119
C21-H*	0,279446	-1,001406	0,005543	+0,295798	0,000112
C20-H	0,280037	-1,000323	0,001519	+0,295351	0,000003
C21-H**	0,279382	-1,000938	0,005591	+0,295611	0,000110
C21-H	0,277751	-0,987998	0,004636	+0,292597	0,000131
C15-H	0,276564	-0,980164	0,004833	+0,290784	0,000134
C15-H*	0,280665	-1,009276	0,005825	+0,298246	0,000095
C15-H**	0,280541	-1,008474	0,005926	+0,298064	0,000098
C22-H*	0,279324	-0,999818	0,005042	+0,295574	0,000119
C22-H	0,277014	-0,983016	0,004694	+0,291397	0,000133
C22-H**	0,280524	-1,009060	0,005992	+0,297886	0,000083
C1-H*	0,275852	-0,973456	0,012590	+0,289290	0,000143
C1-H	0,284366	-1,036750	0,007428	+0,303440	0,000101
C1-H**	0,276409	-0,977184	0,012195	+0,290023	0,000143
C2-H	0,286418	-1,051629	0,007527	+0,305496	0,000072
C2-H*	0,277798	-0,988390	0,011434	+0,292881	0,000139
C2-H**	0,278307	-0,991930	0,011323	+0,293907	0,000158
C3-H	0,278795	-0,995892	0,009739	+0,294500	0,000150
C3-H*	0,276797	-0,982081	0,010401	+0,291099	0,000105
C3-H**	0,285218	-1,043039	0,007252	+0,303540	0,000073
O-H	0,373794	-2,760875	0,021116	+0,766044	0,000461
C6-H**	0,280817	-1,009534	0,002717	+0,296897	0,000133
C6-H	0,279741	-1,002622	0,004524	+0,295483	0,000157
C10-H*	0,280440	-1,007818	0,006052	+0,298000	0,000102

C10-H**	0,280680	-1,009347	0,005967	+0,298201	0,000096
C10-H	0,276663	-0,980790	0,004870	+0,290895	0,000137
C9-H	0,277470	-0,983000	0,003115	+0,291090	0,000123

H represents hydrogen atoms which were removed from the structure to study the antioxidant potential, while H* and H** represent the second and third hydrogen atoms bonding to the same C atom.

Structure of α-Tocopherol and the corresponding radical species in the HAT mechanism optimized at the M05-2X/6-311++G(2d,2p) level of theory in gas phase.

**M05-2X/6-311++G(2d,2p)**											
**α-Tocopherol**				**α-Tocopherol (O•)**				**α-Tocopherol (C1•)**			
C	3.59651900	0.43682900	0.33058300	C	3.656504	-0.526953	-0.329526	C	3.638949	-0.412994	-0.336597
C	5.77121200	-0.56232900	0.03334700	C	5.808399	0.534859	-0.048093	C	5.808774	0.604120	-0.045912
C	6.16792900	0.45700700	-0.82590000	C	6.241109	-0.479333	0.824732	C	6.218464	-0.429888	0.806706
C	5.16441600	1.45924100	-1.33378700	C	5.259792	-1.505109	1.328863	C	5.224142	-1.441474	1.306252
C	3.73717700	0.99743800	-1.07688200	C	3.826156	-1.055530	1.084920	C	3.791090	-0.990779	1.062164
C	6.67778500	-1.51871600	0.49745500	C	6.663636	1.570177	-0.505034	C	6.692884	1.566651	-0.497418
C	7.50515700	0.55122000	-1.21615000	C	7.557484	-0.500881	1.216871	C	7.565567	-0.536038	1.177360
H	5.33669100	2.42427900	-0.85506500	H	5.442905	-2.463145	0.842085	H	5.401873	-2.399411	0.814272
H	3.47117700	0.20436000	-1.77423900	H	3.565330	-0.253297	1.773872	H	3.519253	-0.210540	1.771540
C	8.40005700	-0.40192000	-0.75889800	C	8.478742	0.524983	0.760941	C	8.458294	0.416312	0.735725
C	8.00476400	-1.43910200	0.08402800	C	7.972952	1.576489	-0.113928	C	8.050527	1.513287	-0.074304
O	4.46828900	-0.70399300	0.44996700	O	4.542597	0.611574	-0.502614	O	4.500316	0.737694	-0.442450
C	2.21067000	-0.14981400	0.57036800	C	2.273494	0.067573	-0.558594	C	2.247698	0.166430	-0.561558
H	2.24190400	-0.67725700	1.52392600	H	2.294758	0.580793	-1.519869	H	2.268592	0.701558	-1.511039
H	2.03262800	-0.90018500	-0.19957100	H	2.108731	0.829212	0.203124	H	2.070990	0.909732	0.215442
C	1.06331100	0.85431900	0.58573900	C	1.121653	-0.931693	-0.544595	C	1.106139	-0.844244	-0.575864
H	1.24033400	1.60137700	1.35577100	H	1.286148	-1.691027	-1.305231	H	1.284663	-1.589192	-1.347652
H	1.00861200	1.38258000	-0.36635400	H	1.074506	-1.444683	0.416179	H	1.056356	-1.374214	0.375564
C	-1.50126600	1.05480900	0.68351800	C	-1.444960	-1.118392	-0.622742	C	-1.458354	-1.054514	-0.662530
C	-2.77112400	0.22248500	0.87473500	C	-2.709039	-0.282383	-0.835584	C	-2.731424	-0.229253	-0.862956
H	-2.84082400	-0.07719700	1.92322300	H	-2.779876	-0.016470	-1.893004	H	-2.805498	0.052450	-1.916084
H	-2.67683800	-0.69825400	0.29738300	H	-2.605610	0.655806	-0.288767	H	-2.638230	0.701338	-0.301484
C	-4.06245700	0.92067300	0.45959400	C	-4.004598	-0.955798	-0.394026	C	-4.019046	-0.924413	-0.431684
H	-3.98483200	1.22324600	-0.58672900	H	-3.930071	-1.216174	0.663688	H	-3.939566	-1.202808	0.621141
H	-4.19550200	1.82944200	1.04050400	H	-4.143142	-1.886302	-0.938097	H	-4.147633	-1.846745	-0.991884
C	-5.27901300	0.01647300	0.63358700	C	-5.214322	-0.050615	-0.606344	C	-5.239930	-0.030181	-0.625553
H	-5.06562600	-0.94779600	0.16998500	H	-4.996461	0.928338	-0.176729	H	-5.030921	0.945294	-0.183795
H	-5.43410200	-0.17997900	1.69700500	H	-5.362283	0.108641	-1.677005	H	-5.395903	0.141788	-1.693083
C	-6.57944700	0.56121200	0.03970200	C	-6.521871	-0.564645	-0.000837	C	-6.537877	-0.567328	-0.019469
H	-6.41363000	0.73696100	-1.02621200	H	-6.362168	-0.707765	1.070883	H	-6.370824	-0.719731	1.049850
C	-7.68249800	-0.48977300	0.17857000	C	-7.613915	0.492066	-0.178353	C	-7.644743	0.476524	-0.180056
H	-7.31249200	-1.43541800	-0.22047200	H	-7.241020	1.443298	0.204393	H	-7.279981	1.430243	0.204335
H	-7.87446400	-0.65755300	1.24112500	H	-7.790150	0.637471	-1.246915	H	-7.833487	0.625678	-1.245949
C	-8.99413000	-0.14802300	-0.52124400	C	-8.938051	0.176917	0.510367	C	-8.957668	0.141114	0.520415
H	-8.79374900	0.09236500	-1.56749500	H	-8.753960	-0.047613	1.563132	H	-8.760126	-0.083983	1.570613
H	-9.43196000	0.73930100	-0.07153800	H	-9.379284	-0.713303	0.069867	H	-9.391368	-0.753720	0.081672
C	-9.98987600	-1.30094200	-0.44998700	C	-9.920120	1.339057	0.406550	C	-9.956355	1.290134	0.429716
H	-9.50768100	-2.20497900	-0.82491200	H	-9.433548	2.243779	0.774053	H	-9.478016	2.200591	0.793889
H	-10.24108200	-1.49593200	0.59553200	H	-10.155010	1.520135	-0.645209	H	-10.204525	1.470073	-0.619197
C	-11.28601700	-1.08055300	-1.23009700	C	-11.228855	1.143568	1.172151	C	-11.254502	1.076450	1.208367
H	-11.02318100	-0.88985800	-2.27222800	H	-10.981689	0.964127	2.220084	H	-10.994521	0.899830	2.253669
C	-12.14877000	-2.33765600	-1.17145500	C	-12.077087	2.408917	1.085647	C	-12.120374	2.330360	1.130925
H	-11.61424800	-3.20097200	-1.56083400	H	-11.538002	3.271829	1.469619	H	-11.589258	3.200117	1.510519
H	-12.42665700	-2.55124100	-0.13986300	H	-12.340560	2.611285	0.048041	H	-12.396009	2.529828	0.095928
C	-6.98054900	1.88334000	0.68937900	C	-6.933037	-1.902850	-0.609748	C	-6.934364	-1.904431	-0.640516
H	-7.14367800	1.73829300	1.75760400	H	-7.098267	-1.789003	-1.681411	H	-7.102014	-1.782329	-1.710910
H	-6.20912200	2.63743700	0.56459400	H	-6.166886	-2.658577	-0.464083	H	-6.158771	-2.651985	-0.502946
H	-7.89567900	2.27858900	0.25882100	H	-7.849720	-2.278335	-0.165109	H	-7.846018	-2.295060	-0.198565
C	-12.07257500	0.11709200	-0.70398700	C	-12.021555	-0.052530	0.651832	C	-12.036184	-0.129941	0.695124
H	-12.28757800	-0.01517600	0.35629800	H	-12.221556	0.067628	-0.412821	H	-12.247883	-0.012030	-0.367512
H	-11.52753600	1.04844000	-0.82545700	H	-11.488607	-0.988050	0.793080	H	-11.489322	-1.058273	0.830607
H	-13.02065300	0.21379500	-1.22790100	H	-12.977275	-0.131733	1.164657	H	-12.985820	-0.222228	1.217010
C	9.03854600	-2.44411700	0.52002900	C	8.944952	2.630003	-0.555344	C	8.955344	2.514464	-0.429962
H	9.82113300	-1.97637900	1.11954800	H	9.042243	2.642910	-1.638845	H	8.660620	3.324191	-1.069236

H	8.60250900	-3.23155600	1.11962300	H	8.616879	3.618440	-0.241374	H	9.951482	2.563193	-0.027192
H	9.51116100	-2.91772600	-0.34074600	H	9.914257	2.427083	-0.119180	C	6.228547	2.678982	-1.391186
C	6.21047200	-2.61286300	1.42214900	C	6.072771	2.610185	-1.414806	H	5.195883	2.531819	-1.675125
H	5.17189900	-2.46572200	1.68393100	H	5.705572	2.151509	-2.329785	H	6.313199	3.640709	-0.887377
H	6.30674600	-3.58966300	0.95198500	H	5.221595	3.092232	-0.940941	H	6.835571	2.726627	-2.293129
H	6.79711100	-2.62675900	2.33775700	H	6.803803	3.364924	-1.672546	C	8.029928	-1.671797	2.047273
C	7.96525200	1.65727700	-2.12538000	C	8.083151	-1.560040	2.133609	H	7.687768	-1.542000	3.072851
H	7.61719700	1.49460300	-3.14459500	H	7.734653	-1.394759	3.153251	H	7.638161	-2.620396	1.689510
H	7.57566000	2.61725400	-1.79664200	H	7.743829	-2.546879	1.828615	H	9.110675	-1.723757	2.058306
H	9.04608900	1.70939700	-2.14478200	H	9.165194	-1.530984	2.138723	O	9.768675	0.290141	1.111799
O	9.70784700	-0.29661000	-1.17568900	O	9.671667	0.507511	1.118063	H	10.318405	0.778522	0.497765
H	10.23692600	-0.96076800	-0.73416100	H	5.421538	-1.666679	2.391241	H	5.382615	-1.615450	2.367991
H	5.31437700	1.61873200	-2.39882500	H	3.136892	-1.877852	1.251907	H	3.107422	-1.823704	1.198701
H	3.04324600	1.82049300	-1.22178100	C	4.018233	-1.560082	-1.387310	C	4.018253	-1.412472	-1.422287
C	3.97276200	1.45514300	1.39978200	H	3.474544	-2.485421	-1.217768	H	3.484039	-2.349255	-1.286800
H	3.43343800	2.38701800	1.25084900	H	3.768203	-1.179552	-2.373920	H	3.771969	-1.003478	-2.398652
H	3.73077000	1.06035600	2.38315000	H	5.082239	-1.776333	-1.363656	H	5.084561	-1.617561	-1.398590
H	5.03790700	1.66455600	1.36904700	H	-1.453200	-1.486409	0.406390	H	-1.461632	-1.435347	0.361997
H	-1.50515700	1.44477000	-0.33756200	C	-1.403994	-2.317588	-1.567223	C	-1.406470	-2.241974	-1.621264
C	-1.45549000	2.23389400	1.65275400	H	-2.307865	-2.914260	-1.492405	H	-2.301182	-2.852842	-1.549029
H	-2.34967200	2.84549700	1.58033500	H	-1.309768	-1.974498	-2.597500	H	-1.322261	-1.885485	-2.647896
H	-1.37746200	1.86855600	2.67677200	H	-0.563230	-2.969932	-1.349975	H	-0.554824	-2.883915	-1.415923
H	-0.60264800	2.87749000	1.45812400	H	-13.000131	2.302663	1.650620	H	-13.036657	2.211390	1.704316
H	-13.06376900	-2.21362900	-1.74581600	C	-0.210987	-0.231155	-0.795773	C	-0.232494	-0.154310	-0.825120
C	-0.27150300	0.15856900	0.83907100	H	-0.214783	0.182885	-1.806639	H	-0.237805	0.269161	-1.832093
H	-0.27121500	-0.26754400	1.84495800	H	-0.293632	0.616059	-0.114062	H	-0.324859	0.685837	-0.135897
H	-0.36344500	-0.68009900	0.14796800								
**α-Tocopherol (C2$^•$)**				**α-Tocopherol (C3$^•$)**				**α-Tocopherol (C4$^•$)**			
C	3.625525	-0.462924	-0.332050	C	3.628982	-0.458334	-0.287312	C	3.626712	-0.336815	-0.329225
C	5.784950	0.566568	-0.069735	C	5.801920	0.540519	0.008896	C	5.808182	0.586689	0.067647
C	6.208341	-0.421324	0.798619	C	6.205834	-0.483778	0.837929	C	6.165127	-0.520858	0.877732
C	5.226484	-1.430510	1.334514	C	5.214042	-1.485366	1.359294	C	5.158063	-1.375017	1.332741
C	3.792227	-0.984828	1.087016	C	3.781208	-1.033718	1.111916	C	3.734602	-1.138430	0.963962
C	6.678808	1.561316	-0.564813	C	6.698182	1.520975	-0.460187	C	6.749598	1.491629	-0.394525
C	7.556398	-0.482049	1.174681	C	7.579718	-0.596393	1.200014	C	7.533471	-0.715927	1.203780
H	5.402893	-2.401453	0.870086	H	5.392316	-2.455628	0.892970	H	5.400612	-2.193992	1.985873
H	3.532138	-0.176192	1.768499	H	3.511055	-0.251621	1.819899	H	3.221664	-0.586873	1.758617
C	8.440754	0.494404	0.704294	C	8.464515	0.400597	0.722913	C	8.453933	0.195742	0.739590
C	8.030772	1.524915	-0.117916	C	8.040241	1.445615	-0.079451	C	8.090979	1.299781	-0.046989
O	4.496576	0.676150	-0.500198	O	4.498142	0.686874	-0.397687	O	4.492837	0.817564	-0.230388
C	2.236356	0.117230	-0.565347	C	2.241048	0.128313	-0.513302	C	2.242083	0.272228	-0.504844
H	2.255122	0.624879	-1.529839	H	2.264220	0.661064	-1.464194	H	2.247630	0.850633	-1.428731
H	2.066578	0.883062	0.191064	H	2.067827	0.874058	0.262198	H	2.091786	0.979189	0.310463
C	1.090549	-0.888547	-0.546807	C	1.094406	-0.876659	-0.525677	C	1.089065	-0.725345	-0.532567
H	1.260352	-1.651186	-1.303049	H	1.270787	-1.625173	-1.294420	H	1.259621	-1.465109	-1.310904
H	1.045875	-1.396853	0.416668	H	1.040845	-1.402906	0.427599	H	1.036737	-1.262806	0.414428
C	-1.475756	-1.090464	-0.621992	C	-1.470908	-1.075744	-0.621629	C	-1.477174	-0.911799	-0.646934
C	-2.744960	-0.263927	-0.841137	C	-2.739063	-0.245987	-0.834408	C	-2.739862	-0.056615	-0.773549
H	-2.819530	-0.010041	-1.901246	H	-2.806019	0.028619	-1.889910	H	-2.762656	0.389704	-1.770587
H	-2.645505	0.680728	-0.304825	H	-2.644600	0.688075	-0.278936	H	-2.670529	0.771238	-0.066493
C	-4.035666	-0.939839	-0.389255	C	-4.032330	-0.932045	-0.405533	C	-4.047487	-0.802464	-0.526770
H	-3.957856	-1.186206	0.671567	H	-3.961132	-1.200906	0.650337	H	-3.994381	-1.315982	0.435544
H	-4.168519	-1.878190	-0.921210	H	-4.160796	-1.859034	-0.958024	H	-4.178927	-1.568043	-1.286295
C	-5.251800	-0.045615	-0.611103	C	-5.248136	-0.034664	-0.616027	C	-5.250334	0.135865	-0.531949
H	-5.039262	0.940523	-0.195442	H	-5.038593	0.943465	-0.180386	H	-5.061339	0.949209	0.170105
H	-5.403174	0.098116	-1.683511	H	-5.394904	0.129499	-1.686119	H	-5.345243	0.594991	-1.518924
C	-6.554710	-0.559610	0.004426	C	-6.553034	-0.561748	-0.016063	C	-6.582586	-0.522745	-0.167211
H	-6.391293	-0.687670	1.077503	H	-6.394296	-0.708924	1.055261	H	-6.468255	-0.991809	0.813341
C	-7.653628	0.488042	-0.183819	C	-7.653371	0.486809	-0.190292	C	-7.668420	0.549229	-0.052624
H	-7.286240	1.445817	0.187744	H	-7.287646	1.439944	0.194633	H	-7.318078	1.326664	0.627795
H	-7.831933	0.620308	-1.253738	H	-7.831462	0.633598	-1.258358	H	-7.794964	1.025882	-1.027614
C	-8.975063	0.172354	0.509847	C	-8.974351	0.159555	0.498799	C	-9.021131	0.043579	0.439187
H	-8.788304	-0.040770	1.564526	H	-8.787418	-0.065236	1.551007	H	-8.883007	-0.506373	1.372551
H	-9.411705	-0.724733	0.078702	H	-9.408757	-0.733573	0.057292	H	-9.434310	-0.657144	-0.281600
C	-9.963889	1.327821	0.395967	C	-9.966074	1.313744	0.397932	C	-10.010555	1.181451	0.667115
H	-9.481919	2.238883	0.753754	H	-9.486060	2.222093	0.765135	H	-9.552461	1.919687	1.327114
H	-10.201283	1.497153	-0.657182	H	-10.204491	1.493733	-0.653227	H	-10.201992	1.690096	-0.280780
C	-11.270449	1.132520	1.165318	C	-11.271688	1.107503	1.166012	C	-11.348021	0.751409	1.269597
H	-11.020814	0.964681	2.214567	H	-11.021062	0.929615	2.213384	H	-11.142922	0.244861	2.214427
C	-12.125969	2.392130	1.067754	C	-12.130141	2.366068	1.081648	C	-12.209953	1.977599	1.556203
H	-11.591195	3.261785	1.442443	H	-11.597313	3.232986	1.465320	H	-11.696826	2.674262	2.215097
H	-12.392043	2.582888	0.028629	H	-12.396715	2.566956	0.044547	H	-12.437812	2.499081	0.627311
C	-6.959144	-1.908202	-0.585676	C	-6.952244	-1.900282	-0.632230	C	-6.968070	-1.601524	-1.176814
H	-7.128612	-1.809374	-1.658172	H	-7.115006	-1.782787	-1.703902	H	-7.096159	-1.157141	-2.164154
H	-6.187251	-2.656591	-0.432919	H	-6.180348	-2.650328	-0.487752	H	-6.206480	-2.371976	-1.249428

H	-7.871820	-2.283929	-0.133022	H	-7.867334	-2.285014	-0.192140	H	-7.898603	-2.089030	-0.903087
C	-12.057066	-0.073057	0.657787	C	-12.055846	-0.094699	0.646769	C	-12.101235	-0.214064	0.358184
H	-12.258814	0.035396	-0.407794	H	-12.258982	0.024394	-0.417415	H	-12.250974	0.239465	-0.621591
H	-11.518727	-1.004124	0.807664	H	-11.514925	-1.025893	0.786292	H	-11.565256	-1.148402	0.220502
H	-13.011799	-0.152500	1.172450	H	-13.009838	-0.181997	1.161536	H	-13.079611	-0.447718	0.771637
C	8.984197	2.602969	-0.553177	C	9.054255	2.468188	-0.523050	C	9.170560	2.245950	-0.496208
H	9.351990	2.438385	-1.565536	H	9.823964	2.018720	-1.151676	H	9.907086	1.741193	-1.123600
H	8.490866	3.569746	-0.536856	H	8.595512	3.263214	-1.094929	H	8.767624	3.068097	-1.071915
H	9.838450	2.688896	0.112830	H	9.544563	2.931710	0.333522	H	9.696066	2.678187	0.356806
C	6.215411	2.517045	-1.464442	C	6.201877	2.627071	-1.350062	C	6.319930	2.656855	-1.246564
H	5.189163	2.506938	-1.777387	H	5.157098	2.481688	-1.585922	H	5.259162	2.600604	-1.449486
H	6.867853	3.262701	-1.877568	H	6.309467	3.596076	-0.865273	H	6.520367	3.602283	-0.746852
C	8.043918	-1.570779	2.086202	H	6.763743	2.661229	-2.281090	H	6.852135	2.663170	-2.194832
H	7.735588	-1.384401	3.115016	C	8.064197	-1.638914	1.985970	C	7.952688	-1.885003	2.045067
H	7.634587	-2.534328	1.794202	H	7.415484	-2.415070	2.345747	H	7.487244	-1.840137	3.028378
H	9.124127	-1.629646	2.067809	H	9.107107	-1.687486	2.234870	H	7.648700	-2.821663	1.581320
O	9.747791	0.380932	1.125085	O	9.773671	0.279511	1.096035	H	9.026495	-1.897013	2.175619
H	10.306742	0.925364	0.571827	H	10.284693	0.991835	0.710716	O	9.773805	-0.005404	1.073714
H	5.390239	-1.568495	2.400401	H	5.369922	-1.625427	2.426607	H	10.309530	0.688144	0.689675
H	3.104898	-1.807366	1.262373	H	3.095414	-1.864627	1.250348	H	3.208676	-2.084528	0.849867
C	3.989977	-1.505878	-1.380535	C	4.002860	-1.463193	-1.369784	C	4.043912	-1.158300	-1.541221
H	3.456098	-2.435248	-1.199853	H	3.461107	-2.395158	-1.231418	H	3.401249	-2.026969	-1.650871
H	3.732718	-1.137117	-2.369918	H	3.760778	-1.055911	-2.348004	H	3.969349	-0.547620	-2.437216
H	5.056299	-1.710344	-1.359620	H	5.067152	-1.676903	-1.341405	H	5.067743	-1.504364	-1.436930
H	-1.481615	-1.450497	0.410037	H	-1.482556	-1.452089	0.404472	H	-1.470197	-1.354974	0.352089
C	-1.427955	-2.297066	-1.556767	C	-1.417904	-2.267583	-1.574893	C	-1.458837	-2.040058	-1.675444
H	-2.328711	-2.898211	-1.478270	H	-2.317118	-2.872447	-1.508079	H	-2.321966	-2.689992	-1.569993
H	-1.334153	-1.961462	-2.589568	H	-1.322357	-1.915958	-2.602213	H	-1.468353	-1.624772	-2.683379
H	-0.583394	-2.942348	-1.333464	H	-0.572146	-2.913893	-1.359338	H	-0.572016	-2.658350	-1.573427
H	-13.047631	2.286234	1.635090	H	-13.051464	2.252117	1.647946	H	-13.151459	1.696088	2.021828
C	-0.246537	-0.198098	-0.802192	C	-0.240290	-0.181306	-0.780417	C	-0.242004	-0.018526	-0.774693
H	-0.253430	0.208971	-1.815952	H	-0.239989	0.240640	-1.788106	H	-0.234498	0.431234	-1.770054
H	-0.333447	0.653602	-0.126583	H	-0.331653	0.660324	-0.092833	H	-0.332759	0.804443	-0.064540
**α-Tocopherol (C5$^{\bullet}$)**				**α-Tocopherol (C6$^{\bullet}$)**				**α-Tocopherol (C7$^{\bullet}$)**			
C	3.571552	0.761853	-0.151879	C	-3.616162	-0.354836	0.319919	C	-3.581260	-0.352721	-0.385477
C	5.698435	-0.179834	0.453013	C	-5.787488	0.598322	-0.046926	C	-5.789352	0.066347	0.471964
C	5.828434	-0.850221	-0.753449	C	-6.221639	-0.555151	-0.690205	C	-6.086331	0.922900	-0.583354
C	4.658000	-0.946370	-1.701230	C	-5.241980	-1.630954	-1.082067	C	-5.004490	1.374428	-1.530494
C	3.433541	-0.366542	-1.108863	C	-3.798623	-1.160188	-0.959285	C	-3.621096	1.058306	-0.981026
C	6.745392	-0.113390	1.343601	C	-6.673560	1.618184	0.303813	C	-6.773023	-0.348159	1.373144
C	7.048976	-1.468938	-1.079076	C	-7.579280	-0.717707	-0.971837	C	-7.398453	1.363985	-0.766458
H	4.935425	-0.437240	-2.633182	H	-5.398961	-2.504206	-0.447752	H	-5.142750	0.895520	-2.500629
H	2.459546	-0.656108	-1.471554	H	-3.534693	-0.513786	-1.794524	H	-3.369089	1.751762	-0.181332
C	8.074161	-1.425466	-0.157309	C	-8.456809	0.297074	-0.625823	C	-8.369365	0.958395	0.134145
C	7.937852	-0.754287	1.048062	C	-8.023845	1.464537	-0.000098	C	-8.075019	0.114856	1.203890
O	4.542626	0.420549	0.858368	O	-4.458985	0.810876	0.246249	O	-4.515882	-0.400138	0.706041
C	2.291230	1.034864	0.627378	C	-2.206884	0.239079	0.429736	C	-2.248132	-0.620058	0.219071
H	2.555526	1.773886	1.382684	H	-2.187574	0.863430	1.322678	H	-2.129935	-0.390357	1.265656
H	1.993455	0.127035	1.145274	H	-2.065863	0.899205	-0.425924	C	-1.079168	-1.037915	-0.597014
C	1.135175	1.519299	-0.247010	C	-1.078027	-0.782398	0.495283	H	-1.215967	-2.067702	-0.937778
H	1.370215	2.495332	-0.666531	H	-1.286981	-1.503261	1.283690	H	-1.018056	-0.437519	-1.512228
H	1.023085	0.848604	-1.101211	H	-1.018593	-1.338480	-0.440377	C	1.490674	-1.198132	-0.654771
C	-1.433792	1.655481	-0.369602	C	1.483164	-1.011221	0.599484	C	2.731272	-0.910554	0.193702
C	-2.709980	1.450809	0.452864	C	2.761561	-0.200797	0.824204	H	2.765975	-1.625779	1.019009
H	-2.799904	2.266630	1.170330	H	2.828097	0.064244	1.882128	H	2.621297	0.076146	0.645757
H	-2.620423	0.527575	1.017221	H	2.682742	0.738849	0.275703	C	4.054178	-0.950290	-0.564832
C	-3.995161	1.350828	-0.369513	C	4.045983	-0.902760	0.394446	H	3.999986	-0.271280	-1.418414
H	-3.823562	0.660622	-1.203553	H	3.975642	-1.158625	-0.664708	H	4.219483	-1.947116	-0.964406
H	-4.227628	2.315870	-0.825125	H	4.155659	-1.837978	0.937116	C	5.229862	-0.551818	0.322164
C	-5.178157	0.853685	0.466370	C	5.275691	-0.028480	0.620584	H	4.994850	0.396131	0.808141
H	-4.835499	0.001586	1.049937	H	5.082636	0.959573	0.199881	H	5.344291	-1.288374	1.121057
H	-5.499657	1.613279	1.183851	H	5.423752	0.116638	1.693245	C	6.567181	-0.405546	-0.406680
C	-6.356841	0.387485	-0.378795	C	6.572362	-0.567309	0.013297	H	6.438402	0.337513	-1.197808
H	-5.983777	-0.372971	-1.076216	H	6.412114	-0.693797	-1.060470	C	7.624123	0.121807	0.565986
C	-7.425372	-0.292739	0.506399	C	7.690033	0.459483	0.205414	H	7.227642	1.010283	1.059517
H	-6.951363	-1.087822	1.081392	H	7.339831	1.425327	-0.161820	H	7.782112	-0.622446	1.350188
H	-7.765069	0.450645	1.230403	H	7.871571	0.584057	1.275687	C	8.964732	0.476820	-0.069072
C	-8.656584	-0.884094	-0.193662	C	9.004500	0.122760	-0.491440	H	8.798214	1.159769	-0.904871
H	-8.370490	-1.724063	-0.830273	H	8.812471	-0.079035	-1.547393	H	9.426749	-0.417092	-0.479523
H	-9.118619	-0.142291	-0.836956	H	9.424050	-0.785922	-0.067442	C	9.914795	1.127470	0.931250
C	-9.689339	-1.333829	0.840505	C	10.016305	1.257487	-0.370447	H	9.407768	1.972527	1.399350
H	-9.204752	-2.001960	1.556971	H	9.550559	2.181593	-0.716129	H	10.137140	0.418239	1.732279
H	-9.990398	-0.448574	1.398935	H	10.262134	1.410831	0.683193	C	11.233485	1.619099	0.334651
C	-10.917733	-2.054431	0.309050	C	11.314980	1.045412	-1.148587	H	10.998455	2.332586	-0.457307
H	-10.612147	-3.033556	-0.057275	H	11.057080	0.896758	-2.198706	C	12.054312	2.335532	1.402917

C	-11.921127	-2.273870	1.441212	C	12.196629	2.285756	-1.038539	H	11.498495	3.162682	1.838470
H	-11.470300	-2.823820	2.266867	H	11.677786	3.171240	-1.398264	H	12.309352	1.642825	2.204170
H	-12.270785	-1.311371	1.811487	H	12.472020	2.456636	0.001572	C	7.002176	-1.721436	-1.047061
C	-6.939737	1.536735	-1.204081	C	6.948105	-1.922616	0.607050	H	7.125874	-2.485603	-0.279369
H	-7.304353	2.333389	-0.560586	H	7.110752	-1.826394	1.680860	H	6.268120	-2.078070	-1.763376
H	-6.194476	1.952731	-1.879263	H	6.163955	-2.656888	0.448385	H	7.945997	-1.615697	-1.573235
H	-7.772823	1.203678	-1.810378	H	7.857520	-2.314750	0.161846	C	12.047392	0.478238	-0.270049
C	-11.599024	-1.300736	-0.830634	C	12.078745	-0.182888	-0.660934	H	12.224945	-0.291536	0.481025
H	-11.909334	-0.315013	-0.493453	H	12.285978	-0.093838	0.405399	H	11.541776	0.017859	-1.113725
H	-10.938665	-1.179828	-1.689133	H	11.521196	-1.100712	-0.822128	H	13.013908	0.837808	-0.615592
H	-12.492802	-1.824432	-1.165364	H	13.030043	-0.274303	-1.179951	C	-9.188050	-0.269079	2.143884
C	9.125350	-0.684156	1.970621	C	-9.042273	2.524359	0.329950	H	-9.968092	-0.830979	1.627727
H	9.963944	-0.175984	1.489619	H	-9.786876	2.154161	1.036442	H	-8.831300	-0.887759	2.956051
H	8.898045	-0.120330	2.857416	H	-8.582954	3.395880	0.776227	H	-9.647320	0.614223	2.588445
H	9.462537	-1.674775	2.274379	H	-9.565410	2.858343	-0.566669	C	-6.413980	-1.276520	2.504319
C	6.557849	0.650946	2.627097	C	-6.162188	2.857066	0.992317	H	-5.379847	-1.582126	2.427383
H	5.608477	1.175748	2.632772	H	-5.102996	2.768525	1.190473	H	-6.554442	-0.790784	3.468016
H	6.575252	-0.025172	3.485499	H	-6.318965	3.739772	0.375489	H	-7.037261	-2.167542	2.488656
H	7.353616	1.380627	2.751853	H	-6.675763	3.018058	1.937282	C	-7.750410	2.273699	-1.911206
C	7.257508	-2.157482	-2.404416	C	-8.079818	-1.964720	-1.647039	H	-7.374872	3.281381	-1.737973
H	6.524665	-2.949377	-2.569241	H	-7.779969	-1.988373	-2.694028	H	-7.311397	1.916752	-2.839187
H	7.156281	-1.441539	-3.219976	H	-7.672494	-2.853053	-1.171160	H	-8.823843	2.332804	-2.035890
H	8.246707	-2.591098	-2.449264	H	-9.160083	-2.013910	-1.606093	O	-9.649591	1.425977	-0.058272
O	9.249820	-2.050138	-0.466184	O	-9.786966	0.117507	-0.930745	H	-10.233639	1.024251	0.584863
H	9.925454	-1.722159	0.131409	H	-10.293187	0.854270	-0.589076	H	-5.094952	2.444107	-1.704906
H	4.493088	-1.987897	-1.975315	H	-5.439822	-1.953360	-2.101729	H	-2.864933	1.149130	-1.756768
C	4.068276	2.027698	-0.852573	H	-3.123703	-2.011569	-0.963967	C	-3.956426	-1.417961	-1.414172
H	3.402956	2.279023	-1.671796	C	-3.971573	-1.143799	1.529831	H	-3.306024	-1.351153	-2.282896
H	4.079828	2.844025	-0.128856	H	-3.862063	-2.214085	1.549483	H	-3.852388	-2.403237	-0.969351
H	5.071920	1.865987	-1.235585	H	-4.259406	-0.623463	2.426650	H	-4.984792	-1.291276	-1.737512
H	-1.373319	0.846656	-1.111336	H	1.497324	-1.385088	-0.427526	H	1.497795	-0.509698	-1.503849
C	-1.460380	2.981765	-1.120339	C	1.404363	-2.204987	1.548670	C	1.498292	-2.627879	-1.190894
H	-2.325936	3.025971	-1.773105	H	2.294260	-2.824148	1.486971	H	2.393793	-2.831220	-1.770041
H	-1.511916	3.809494	-0.416227	H	1.306135	-1.855122	2.576330	H	1.462862	-3.335207	-0.362303
H	-0.576283	3.110535	-1.731302	H	0.549590	-2.836491	1.324438	H	0.644997	-2.819459	-1.834725
H	-12.786421	-2.832000	1.086715	H	13.112908	2.168930	-1.612385	H	12.981461	2.726054	0.990038
C	-0.192587	1.545157	0.520283	C	0.263482	-0.101167	0.750636	C	0.235000	-0.915291	0.170681
H	-0.190376	2.362125	1.246582	H	0.264618	0.326236	1.755879	H	0.212364	-1.593456	1.026107
H	-0.254005	0.623722	1.088833	H	0.367033	0.735770	0.058696	H	0.303061	0.094271	0.575674
**α-Tocopherol (C8•)**				**α-Tocopherol (C9•)**				**α-Tocopherol (C10•)**			
C	-3.387265	1.505561	-0.275473	C	3.586629	-0.619190	-0.199295	C	-3.593631	-0.280867	0.299231
C	-4.918683	-0.324826	-0.562798	C	5.706974	0.526396	-0.196896	C	-5.832226	0.520032	-0.093498
C	-4.844988	-0.647225	0.788863	C	6.103710	-0.125004	0.966210	C	-6.213712	-0.696646	-0.649143
C	-3.811984	0.001755	1.674347	C	5.116292	-0.944498	1.755828	C	-5.181532	-1.747816	-0.964026
C	-2.747933	0.711984	0.851837	C	3.686170	-0.663206	1.318422	C	-3.771474	-1.176636	-0.917459
C	-5.852136	-0.930569	-1.406907	C	6.597871	1.310812	-0.933562	C	-6.768071	1.518610	0.186884
C	-5.740223	-1.573906	1.327213	C	7.426366	-0.018819	1.400731	C	-7.562890	-0.944721	-0.909794
H	-4.299058	0.710248	2.345816	H	5.341937	-2.004978	1.635136	H	-5.273198	-2.573158	-0.256451
H	-2.078466	-0.018418	0.399399	H	3.366192	0.306891	1.695485	H	-3.586068	-0.569375	-1.802351
C	-6.661831	-2.175796	0.486328	C	8.305988	0.763759	0.670880	C	-8.487714	0.048715	-0.633432
C	-6.723691	-1.876354	-0.873305	C	7.909974	1.434775	-0.484653	C	-8.109672	1.278547	-0.098077
O	-4.067045	0.585175	-1.146940	O	4.417927	0.457761	-0.671828	O	-4.519164	0.817744	0.185968
C	-2.326611	2.144808	-1.171916	C	2.191326	-0.219149	-0.663021	C	-2.231060	0.401313	0.308573
H	-2.857266	2.532528	-2.049184	H	2.251752	-0.005196	-1.729696	H	-2.250861	1.152152	1.098643
H	-1.671773	1.351720	-1.532648	H	1.925042	0.711788	-0.165457	H	-2.122228	0.937045	-0.634283
C	-1.521703	3.219932	-0.533197	C	1.099037	-1.260147	-0.424919	C	-1.037170	-0.522411	0.519368
H	-2.028169	4.015706	-0.013125	H	1.360181	-2.181165	-0.957387	H	-1.133594	-1.053323	1.464819
C	0.752378	2.321691	0.161532	H	1.046962	-1.533043	0.631554	H	-0.996950	-1.276774	-0.266485
C	2.199293	2.250122	-0.323734	C	-1.495657	-1.423281	-0.405398	C	1.517506	-0.642646	0.675179
H	2.636862	3.249956	-0.265492	C	-2.712351	-0.594863	-0.822497	C	2.787521	0.213176	0.564172
H	2.201404	1.972613	-1.378840	H	-2.737272	-0.537862	-1.912887	H	2.815885	0.903305	1.410097
C	3.083101	1.270983	0.442012	H	-2.573667	0.424152	-0.460558	H	2.726812	0.823688	-0.337431
H	2.624741	0.279813	0.424787	C	-4.048525	-1.126231	-0.314929	C	4.071725	-0.606808	0.534920
H	3.143978	1.573229	1.484320	H	-3.988031	-1.288406	0.763356	H	4.049054	-1.278564	-0.325709
C	4.485399	1.193101	-0.153539	H	-4.251196	-2.092983	-0.768289	H	4.106811	-1.235901	1.422702
H	4.400086	1.018538	-1.226990	C	-5.193536	-0.163692	-0.613763	C	5.313876	0.274192	0.456872
H	4.978107	2.161409	-0.036279	H	-4.938761	0.814838	-0.204587	H	5.177804	0.999943	-0.346633
C	5.386762	0.110148	0.443149	H	-5.282946	-0.032148	-1.694887	H	5.405732	0.850304	1.380692
H	4.904841	-0.857066	0.279884	C	-6.553525	-0.588104	-0.055459	C	6.623285	-0.478371	0.213667
C	6.725877	0.103318	-0.296762	H	-6.434420	-0.775082	1.014782	H	6.522904	-1.031117	-0.723865
H	6.530109	0.087730	-1.369983	C	-7.556352	0.555142	-0.224309	C	7.767256	0.524090	0.049137
H	7.242004	1.044485	-0.091864	H	-7.129680	1.458229	0.214234	H	7.466162	1.277966	-0.679848
C	7.650936	-1.061519	0.041951	H	-7.680542	0.758289	-1.290770	H	7.910992	1.050343	0.995835
H	7.125483	-2.002666	-0.132330	C	-8.924553	0.308018	0.403624	C	9.094421	-0.080226	-0.398651
H	7.908694	-1.034575	1.097664	H	-8.794550	0.016055	1.448005	H	8.936801	-0.659669	-1.310726

C	8.925165	-1.032139	-0.795486	H	-9.413401	-0.523134	-0.097643	H	9.458444	-0.772283	0.356293
H	8.651339	-0.952654	-1.848645	C	-9.818567	1.541593	0.333416	C	10.147692	0.991652	-0.659487
H	9.493082	-0.130059	-0.555134	H	-9.279880	2.391068	0.755877	H	9.730696	1.734624	-1.341136
C	9.837500	-2.245815	-0.619069	H	-10.014032	1.788192	-0.713073	H	10.367528	1.516730	0.273248
H	9.263151	-3.137842	-0.875684	C	-11.155688	1.401347	1.061019	C	11.458843	0.472336	-1.249226
C	11.025791	-2.146614	-1.571105	H	-10.945370	1.159546	2.104543	H	11.225916	-0.045644	-2.181374
H	10.695958	-2.052689	-2.603154	C	-11.916336	2.723424	1.015948	C	12.387497	1.640917	-1.565885
H	11.625425	-1.269916	-1.328256	H	-11.327401	3.531644	1.443403	H	11.914643	2.347369	-2.244260
C	5.577761	0.308040	1.944767	H	-12.148712	2.985445	-0.015772	H	12.643234	2.173187	-0.650472
H	6.018296	1.286413	2.137897	C	-7.053177	-1.870004	-0.717821	C	6.909592	-1.475244	1.334019
H	4.632891	0.252172	2.476858	H	-7.215561	-1.698002	-1.781981	H	6.992229	-0.950152	2.285834
H	6.234257	-0.447013	2.366249	H	-6.338075	-2.680210	-0.611767	H	6.117474	-2.212567	1.423475
C	10.329608	-2.386558	0.818867	H	-7.990395	-2.204521	-0.283453	H	7.837888	-2.011724	1.162439
H	10.841267	-1.475316	1.128362	C	-12.013756	0.285320	0.471149	C	12.156934	-0.510152	-0.312725
H	9.514214	-2.569656	1.512345	H	-12.177104	0.463209	-0.591707	H	12.334028	-0.040025	0.654529
H	11.033139	-3.211397	0.904911	H	-11.549225	-0.689756	0.584362	H	11.567761	-1.407833	-0.150144
C	-7.743176	-2.590957	-1.721553	H	-12.985786	0.251018	0.957678	H	13.119458	-0.811211	-0.719732
H	-8.760837	-2.338466	-1.419330	C	8.926835	2.275159	-1.212066	C	-9.177438	2.310486	0.155692
H	-7.645465	-2.332215	-2.767142	H	9.755185	1.667330	-1.579374	H	-9.897841	1.963650	0.898269
H	-7.626829	-3.672261	-1.644145	H	8.493990	2.776309	-2.067068	H	-8.758679	3.237202	0.523989
C	-5.899387	-0.560234	-2.866459	H	9.337758	3.045767	-0.559235	H	-9.722267	2.542772	-0.759658
H	-5.202934	0.239963	-3.074746	C	6.129580	2.007163	-2.185044	C	-6.316957	2.827988	0.780294
H	-5.637055	-1.410017	-3.493834	H	5.099339	1.754016	-2.393497	H	-5.254005	2.806440	0.976538
H	-6.895606	-0.232009	-3.152949	H	6.198987	3.088055	-2.079655	H	-6.520201	3.654547	0.102242
C	-5.701040	-1.919654	2.790266	H	6.734206	1.718291	-3.041774	H	-6.834368	3.031609	1.714920
H	-4.836308	-2.540363	3.021804	C	7.887066	-0.729908	2.643159	C	-8.004190	-2.260587	-1.488633
H	-5.630174	-1.021539	3.398363	H	7.494345	-0.247196	3.537099	H	-7.722671	-2.339183	-2.537958
H	-6.591258	-2.464231	3.077028	H	7.541719	-1.760551	2.650569	H	-7.539218	-3.090872	-0.963288
O	-7.520513	-3.097645	1.040830	H	8.967443	-0.724779	2.706968	H	-9.079224	-2.365866	-1.421272
H	-8.162058	-3.368128	0.384172	O	9.598400	0.865844	1.133277	O	-9.808206	-0.216478	-0.917427
H	-3.349984	-0.750432	2.309177	H	10.119498	1.372535	0.510527	H	-10.350517	0.511334	-0.613761
H	-2.151442	1.375328	1.472821	H	5.227516	-0.729065	2.815663	H	-5.376368	-2.168869	-1.947439
C	-4.387149	2.538879	0.228514	H	3.013957	-1.418088	1.716501	H	-3.040269	-1.979706	-0.900757
H	-3.940902	3.158902	1.001354	C	4.049301	-1.917553	-0.849481	C	-3.864565	-1.017584	1.605328
H	-4.705884	3.173236	-0.594608	H	3.549997	-2.772650	-0.401085	H	-3.285217	-1.935781	1.656651
H	-5.264569	2.051196	0.642220	H	3.826601	-1.896126	-1.913311	H	-3.596090	-0.383829	2.446654
H	0.308795	1.328033	0.067340	H	5.121168	-2.042880	-0.728212	H	-4.917724	-1.268521	1.688890
C	0.662513	2.764638	1.618807	H	-1.473807	-1.469212	0.689143	H	1.515486	-1.358415	-0.148701
H	1.081919	2.024826	2.294335	C	-1.589658	-2.871854	-0.915504	C	1.487626	-1.386422	1.963478
H	1.208015	3.698254	1.756684	H	-2.441345	-3.387489	-0.480758	H	1.597735	-0.841936	2.888323
H	-0.371515	2.936038	1.906976	H	-1.696196	-2.878179	-1.999088	H	1.313294	-2.445993	2.016401
H	11.666087	-3.022291	-1.494578	H	-0.693925	-3.430465	-0.658235	H	13.312269	1.295281	-2.021824
C	-0.049992	3.286013	-0.739344	H	-12.853640	2.656320	1.563293	C	0.274979	0.253431	0.521234
H	0.318059	4.296329	-0.560501	C	-0.235727	-0.776632	-0.868808	H	0.266476	0.980412	1.335148
H	0.180053	3.039474	-1.779295	H	-0.283740	-0.049305	-1.666324	H	0.364279	0.818176	-0.407476

**α-Tocopherol (C11•)**				**α-Tocopherol (C12•)**				**α-Tocopherol (C13•)**			
C	3.412469	0.683920	-0.142399	C	-3.517157	0.645779	-0.028688	C	-3.567420	-0.161441	0.257949
C	5.537903	-0.257933	0.490735	C	-5.634693	-0.468348	-0.322401	C	-5.875390	0.444093	-0.082888
C	5.732726	-0.776197	-0.785452	C	-5.992626	-0.226575	0.999798	C	-6.228284	-0.877788	-0.333216
C	4.611298	-0.766169	-1.791699	C	-4.979769	0.312075	1.976364	C	-5.164855	-1.929423	-0.514533
C	3.274412	-0.464895	-1.130062	C	-3.564182	0.205648	1.427173	C	-3.791388	-1.306297	-0.719414
C	6.553501	-0.280477	1.449340	C	-6.548391	-0.990255	-1.241428	C	-6.842661	1.440832	0.067832
C	6.976483	-1.306076	-1.133830	C	-7.299438	-0.481894	1.419645	C	-7.576858	-1.230124	-0.413584
H	4.819245	-0.024743	-2.564556	H	-5.212621	1.352674	2.207139	H	-5.149754	-2.587091	0.355741
H	2.929174	-1.335658	-0.574684	H	-3.225034	-0.828394	1.469179	H	-3.713174	-0.898646	-1.726295
C	7.980461	-1.329386	-0.180033	C	-8.201317	-1.004123	0.507289	C	-8.533088	-0.238236	-0.271161
C	7.784608	-0.833460	1.107642	C	-7.843170	-1.269935	-0.813079	C	-8.186091	1.091646	-0.040156
O	4.336040	0.280793	0.886956	O	-4.363277	-0.235759	-0.791520	O	-4.563514	0.847732	0.003379
C	2.117525	0.936920	0.619744	C	-2.138848	0.432037	-0.642181	C	-2.253385	0.562686	-0.006792
H	2.348898	1.629546	1.429263	H	-2.230891	0.608814	-1.713999	H	-2.238750	1.448862	0.628366
H	1.815706	-0.001712	1.084012	H	-1.880050	-0.618777	-0.516122	H	-2.270937	0.914201	-1.038066
C	0.967100	1.496493	-0.208880	C	-1.023240	1.306128	-0.079578	C	-0.989456	-0.254626	0.236291
H	1.262774	2.440245	-0.662142	H	-1.275940	2.355499	-0.212655	H	-0.992012	-0.644395	1.251457
H	0.712665	0.816824	-1.021171	H	-0.913362	1.135671	0.991541	H	-0.958736	-1.112549	-0.435828
C	-1.478635	2.199088	-0.080377	C	1.516700	1.698508	-0.140334	C	1.572220	-0.194308	0.038984
C	-2.825882	2.037993	0.541188	C	2.779183	1.235327	-0.782019	C	2.752158	0.748722	-0.214280
H	-3.285516	3.019656	0.701118	H	2.733051	0.809185	-1.774216	H	2.840567	1.440813	0.628007
H	-2.732165	1.574443	1.523006	C	4.112961	1.550250	-0.204944	H	2.516205	1.374613	-1.085337
C	-3.792121	1.200074	-0.309466	H	4.044184	1.561110	0.886285	C	4.047384	0.052402	-0.426299
H	-3.394583	0.189326	-0.406808	H	4.417605	2.561662	-0.493637	H	4.036951	-0.906090	-0.926984
H	-3.831140	1.620440	-1.311995	C	5.188066	0.554757	-0.641784	C	5.347221	0.756988	-0.279963
C	-5.188337	1.151070	0.301229	H	4.823950	-0.452320	-0.439650	H	5.507185	1.427816	-1.134794
H	-5.098840	0.875733	1.353346	H	5.321879	0.625783	-1.723464	H	5.315725	1.407834	0.598985
H	-5.624399	2.152760	0.280679	C	6.546873	0.732430	0.037578	C	6.553638	-0.178726	-0.162365

C	-6.157923	0.179809	-0.374934	H	6.392258	0.683401	1.118442	H	6.531509	-0.860127	-1.016270
H	-5.722038	-0.821074	-0.324249	C	7.465069	-0.425150	-0.359536	C	7.850174	0.626961	-0.233014
C	-7.477918	0.159896	0.398536	H	6.946642	-1.363142	-0.156304	H	7.845811	1.214912	-1.152011
H	-7.258588	-0.004487	1.454530	H	7.625963	-0.389546	-1.439761	H	7.863748	1.342592	0.592670
H	-7.938128	1.148597	0.329445	C	8.816220	-0.453468	0.348295	C	9.124235	-0.210803	-0.192540
C	-8.481698	-0.894465	-0.057502	H	8.660517	-0.404424	1.428154	H	9.064473	-0.995380	-0.950126
H	-8.004695	-1.876665	-0.047047	H	9.394509	0.424475	0.071986	H	9.203237	-0.708416	0.770736
H	-8.780618	-0.699472	-1.084175	C	9.604874	-1.713788	0.009170	C	10.370485	0.632747	-0.438201
C	-9.716331	-0.923236	0.837450	H	8.979780	-2.584276	0.214052	H	10.239906	1.188236	-1.368102
H	-9.394749	-1.026168	1.874927	H	9.815192	-1.729408	-1.063078	H	10.466037	1.377480	0.355893
H	-10.234086	0.036543	0.767969	C	10.922840	-1.870304	0.767964	C	11.674264	-0.160777	-0.523643
C	-10.712525	-2.039345	0.522700	H	10.699692	-1.854416	1.836406	H	11.562098	-0.905412	-1.313892
H	-10.184795	-2.991823	0.598701	C	11.562661	-3.213258	0.428816	C	12.826089	0.768008	-0.896133
C	-11.844041	-2.031681	1.546137	H	10.891576	-4.038048	0.657100	H	12.629177	1.282268	-1.833910
H	-11.459898	-2.135392	2.558248	H	11.798906	-3.255931	-0.633900	H	12.963608	1.521876	-0.121559
H	-12.392645	-1.092178	1.489088	C	7.172555	2.083525	-0.300054	C	6.463657	-1.001764	1.119742
C	-6.373617	0.539212	-1.842862	H	7.348224	2.154236	-1.373651	H	6.575723	-0.350371	1.986724
H	-6.787815	1.544517	-1.922436	H	6.527675	2.906908	-0.008236	H	5.497179	-1.493979	1.193208
H	-5.441819	0.512330	-2.399669	H	8.123051	2.219499	0.207007	H	7.235916	-1.763774	1.165896
H	-7.062021	-0.148590	-2.324501	C	11.896732	-0.735674	0.461640	C	11.988818	-0.886333	0.781845
C	-11.282852	-1.915416	-0.887682	H	12.084036	-0.685020	-0.610866	H	12.039062	-0.171287	1.602847
H	-11.755618	-0.941465	-1.014181	H	11.515044	0.228231	0.785000	H	11.236389	-1.630338	1.026248
H	-10.515376	-2.021173	-1.648778	H	12.848914	-0.902491	0.959829	H	12.949987	-1.391179	0.718476
H	-12.036890	-2.678919	-1.065066	C	-8.880944	-1.854384	-1.735580	C	-9.288715	2.109946	0.089545
C	8.925214	-0.912562	2.088780	H	-9.719148	-1.170289	-1.875837	H	-9.926288	1.898113	0.949161
H	9.778189	-0.319476	1.755605	H	-8.472244	-2.066937	-2.714116	H	-8.896897	3.109735	0.218295
H	8.640423	-0.544791	3.065151	H	-9.273226	-2.791955	-1.340142	H	-9.917321	2.122798	-0.801563
H	9.260580	-1.942108	2.217882	C	-6.123179	-1.246023	-2.664071	C	-6.422992	2.862618	0.337259
C	6.303198	0.281665	2.824659	H	-5.110158	-0.903374	-2.822381	H	-5.351050	2.921079	0.465695
H	5.310688	0.706308	2.883464	H	-6.162140	-2.308082	-2.898495	H	-6.701735	3.515766	-0.487468
H	6.386293	-0.493253	3.584131	H	-6.774336	-0.728175	-3.364293	H	-6.897983	3.244802	1.237593
H	7.024068	1.059727	3.064934	C	-7.719451	-0.207562	2.837328	C	-7.985825	-2.656003	-0.660753
C	7.220124	-1.850414	-2.514336	H	-7.291047	-0.940560	3.519783	H	-7.793309	-2.943733	-1.693643
H	6.711827	-2.803742	-2.653042	H	-7.382571	0.774385	3.159409	H	-7.426908	-3.335921	-0.023100
H	6.844775	-1.167454	-3.272064	H	-8.796727	-0.252238	2.931287	H	-9.042217	-2.789013	-0.467431
H	8.277680	-2.007461	-2.681837	O	-9.477772	-1.262461	0.953203	O	-9.854223	-0.611345	-0.371610
O	9.191282	-1.872302	-0.546243	H	-10.010610	-1.579898	0.224327	H	-10.412100	0.150573	-0.216357
H	9.808061	-1.792716	0.181265	H	-5.052612	-0.230574	2.915720	H	-5.414765	-2.558627	-1.365529
H	4.565410	-1.727395	-2.297749	H	-2.883466	0.808253	2.021770	H	-3.015277	-2.056892	-0.598611
H	2.526549	-0.225639	-1.880766	C	-4.003499	2.077770	-0.216500	C	-3.684361	-0.610749	1.709543
C	3.936863	1.954571	-0.800725	H	-3.484967	2.752087	0.460148	H	-3.049923	-1.472769	1.898487
H	3.349669	2.202865	-1.681271	H	-3.821397	2.395935	-1.239788	H	-3.386258	0.199075	2.370326
H	3.884202	2.781287	-0.096983	H	-5.069568	2.147662	-0.022047	H	-4.710026	-0.881262	1.942559
H	4.972300	1.827996	-1.102165	H	1.556103	1.428314	0.920550	H	1.544739	-0.921138	-0.776405
C	-1.326267	3.165700	-1.205053	C	1.396118	3.230812	-0.212450	C	1.765506	-0.945186	1.353575
H	-2.289344	3.400363	-1.650692	H	2.286681	3.704335	0.191521	H	2.744153	-1.417399	1.384938
H	-0.886349	4.109409	-0.861110	H	1.282820	3.546191	-1.248280	H	1.700814	-0.249998	2.190875
H	-0.674773	2.788554	-1.991902	H	0.540498	3.588407	0.354251	H	1.013681	-1.716413	1.494168
H	-12.546267	-2.841318	1.361994	H	12.486505	-3.360120	0.983262	H	13.758522	0.217566	-0.996633
C	-0.273355	1.717787	0.655975	C	0.306151	1.002605	-0.764184	C	0.261862	0.590996	0.017606
H	-0.011152	2.434798	1.448181	H	0.244337	1.283489	-1.817532	H	0.306041	1.369880	0.782447
H	-0.517750	0.789761	1.175449	H	0.478325	-0.073500	-0.738661	H	0.177241	1.103841	-0.941456
**α-Tocopherol (C14$^{\bullet}$)**				**α-Tocopherol (C15$^{\bullet}$)**				**α-Tocopherol (C16$^{\bullet}$)**			
C	3.523650	-0.531974	-0.170710	C	-3.576805	-0.304691	0.332216	C	3.535483	-0.535123	-0.236532
C	5.681229	0.542218	-0.181765	C	-5.802255	0.505429	-0.108373	C	5.702546	0.519350	-0.154105
C	6.049202	-0.093018	0.999619	C	-6.173374	-0.705305	-0.683824	C	6.039900	-0.169518	1.006173
C	5.030528	-0.859535	1.802486	C	-5.136802	-1.757070	-0.982288	C	4.998764	-0.964438	1.750282
C	3.612692	-0.544273	1.348214	C	-3.726578	-1.191023	-0.895182	C	3.593422	-0.616243	1.281668
C	6.602499	1.276424	-0.932665	C	-6.742562	1.502469	0.162375	C	6.644062	1.282591	-0.848974
C	7.372155	-0.020542	1.440009	C	-7.516692	-0.947721	-0.977806	C	7.352742	-0.124546	1.479579
H	5.222987	-1.929413	1.710456	H	-5.249143	-2.588057	-0.284325	H	5.185162	-2.030459	1.612327
H	3.320599	0.443581	1.701158	H	-3.517478	-0.577017	-1.769978	H	3.301426	0.356970	1.673605
C	8.282014	0.712596	0.695935	C	-8.445860	0.044436	-0.711386	C	8.282639	0.636934	0.791053
C	7.915790	1.366396	-0.479092	C	-8.077949	1.267556	-0.154047	C	7.946292	1.346109	-0.360754
O	4.392818	0.506409	-0.661869	O	-4.494916	0.798194	0.201771	O	4.425788	0.512871	-0.665295
C	2.144066	-0.100183	-0.653146	C	-2.212023	0.371334	0.380793	C	2.171086	-0.064607	-0.725866
H	2.218584	0.082999	-1.725097	H	-2.257602	1.134950	1.157555	H	2.260843	0.138978	-1.793047
H	1.912744	0.855931	-0.184376	H	-2.064125	0.891315	-0.565354	H	1.956615	0.886748	-0.239864
C	1.016125	-1.092175	-0.388906	C	-1.030865	-0.553953	0.653194	C	1.017318	-1.033653	-0.490645
H	1.233454	-2.031187	-0.891598	H	-1.175407	-1.055505	1.606878	H	1.222181	-1.975555	-0.993789
H	0.942615	-1.309130	0.676961	H	-0.971089	-1.329563	-0.110757	H	0.918863	-1.253017	0.572590
C	-1.535252	-1.413064	-0.534277	C	1.538821	-0.643099	0.800314	C	-1.543867	-1.258544	-0.616865
C	-2.815911	-0.668644	-0.915715	C	2.781941	0.234010	0.637848	C	-2.799271	-0.506309	-1.062812
H	-2.827510	-0.516137	-1.997141	H	2.798879	0.971810	1.443510	H	-2.827762	-0.484324	-2.154920
H	-2.793762	0.322333	-0.462935	H	2.690403	0.795502	-0.293291	H	-2.717163	0.531086	-0.735565

C	-4.111772	-1.362126	-0.494845	C	4.104648	-0.524543	0.622645	C	-4.110640	-1.075543	-0.531956
H	-4.068450	-1.606018	0.570301	H	4.046885	-1.338617	-0.103001	H	-4.068369	-1.125461	0.557036
H	-4.193045	-2.321695	-1.015793	H	4.290065	-0.981453	1.592702	H	-4.248360	-2.092067	-0.891956
C	-5.316566	-0.533280	-0.764485	C	5.281737	0.377202	0.272449	C	-5.303616	-0.219792	-0.953565
H	-5.320244	0.105436	-1.636245	H	5.113955	0.831743	-0.704774	H	-5.088288	0.825646	-0.723791
C	-6.581033	-0.715644	0.002882	H	5.342508	1.193678	0.994604	H	-5.384588	-0.261206	-2.050232
H	-6.346701	-0.632061	1.070289	C	6.633617	-0.358523	0.250467	C	-6.620458	-0.579931	-0.343522
C	-7.592009	0.376440	-0.349681	H	6.553910	-1.182740	-0.460316	C	-7.720380	0.414950	-0.531410
H	-7.109164	1.345885	-0.223444	C	7.734490	0.593064	-0.241683	H	-7.305452	1.418627	-0.424220
H	-7.843179	0.288522	-1.408924	H	7.419988	1.040233	-1.185310	H	-8.087956	0.362323	-1.567264
C	-8.872672	0.348884	0.476646	H	7.833909	1.409748	0.476411	C	-8.919930	0.273666	0.405134
H	-8.618446	0.360207	1.538846	C	9.085478	-0.087086	-0.426173	H	-8.569904	0.241669	1.438106
H	-9.408729	-0.578344	0.290337	H	8.981337	-0.904372	-1.142777	H	-9.424922	-0.669218	0.210992
C	-9.779243	1.534433	0.162215	H	9.384097	-0.536284	0.519375	C	-9.903939	1.425466	0.235622
H	-9.200891	2.454626	0.257349	C	10.158048	0.879539	-0.914395	H	-9.363510	2.367672	0.337333
H	-10.098643	1.476776	-0.881354	H	9.790279	1.393510	-1.804094	H	-10.309134	1.409210	-0.779134
C	-11.021018	1.645319	1.046987	H	10.321459	1.650688	-0.157616	C	-11.066682	1.421701	1.227752
H	-10.688937	1.712950	2.084625	C	11.500509	0.228916	-1.247610	H	-10.647649	1.441804	2.235387
C	-11.793029	2.915818	0.703534	H	11.322928	-0.538152	-2.003674	C	-11.920050	2.671861	1.035639
H	-11.165357	3.798063	0.805841	C	12.454142	1.268184	-1.829793	H	-11.324765	3.575576	1.144434
H	-12.145369	2.870887	-0.326432	H	12.029101	1.740950	-2.712134	H	-12.357736	2.675704	0.037965
C	-7.158302	-2.124039	-0.221747	H	12.652721	2.046408	-1.093573	C	-6.960266	-2.010650	-0.101805
H	-7.490966	-2.229226	-1.253086	C	6.965703	-0.907531	1.593138	H	-7.275255	-2.506883	-1.029590
H	-6.405066	-2.882980	-0.028304	H	6.983733	-1.962282	1.800152	H	-6.111381	-2.567089	0.283817
H	-8.001869	-2.316093	0.435427	H	7.164243	-0.225537	2.405255	H	-7.777797	-2.114574	0.605581
C	-11.930971	0.427772	0.909555	C	12.131166	-0.434730	-0.026385	C	-11.932336	0.172095	1.090363
H	-12.215827	0.291281	-0.133563	H	12.259845	0.297551	0.770604	H	-12.304278	0.086201	0.069485
H	-11.447815	-0.483079	1.250484	H	11.520718	-1.247402	0.355834	H	-11.382368	-0.733734	1.327789
H	-12.841001	0.561308	1.489849	H	13.110961	-0.838055	-0.271569	H	-12.791065	0.225248	1.755634
C	8.965435	2.150584	-1.222441	C	-9.149843	2.297748	0.089354	C	9.015396	2.160825	-1.040807
H	9.777979	1.505480	-1.560130	H	-9.885409	1.943425	0.813312	H	9.828119	1.528529	-1.401816
H	8.557032	2.635663	-2.098446	H	-8.737923	3.219844	0.476448	H	8.625961	2.699128	-1.894152
H	9.393764	2.929819	-0.591315	H	-9.675935	2.540816	-0.834088	H	9.439552	2.898120	-0.358596
C	6.165451	1.955997	-2.204477	C	-6.302671	2.804393	0.780170	C	6.240340	2.022008	-2.098228
H	5.127091	1.736701	-2.410914	H	-5.243340	2.780560	0.994954	H	5.208639	1.812172	-2.344015
H	6.275952	3.035895	-2.128149	H	-6.494211	3.639404	0.109157	H	6.345703	3.097137	-1.966664
H	6.761648	1.621261	-3.050359	H	-6.836620	2.996085	1.708045	H	6.860501	1.729276	-2.942382
C	7.801233	-0.715073	2.702924	C	-7.946869	-2.256489	-1.580629	C	7.749142	-0.879083	2.718486
H	7.413031	-0.201652	3.581607	H	-7.632830	-2.328135	-2.621181	H	7.337326	-0.408741	3.610338
H	7.428060	-1.735627	2.730222	H	-7.502748	-3.093520	-1.047937	H	7.376360	-1.899673	2.686595
H	8.880639	-0.737790	2.777826	H	-9.023954	-2.356437	-1.547687	H	8.826210	-0.905908	2.820570
O	9.574866	0.781776	1.163304	O	-9.760316	-0.215458	-1.026692	O	9.564718	0.679015	1.290645
H	10.113789	1.264453	0.536729	H	-10.308061	0.509797	-0.726663	H	10.124432	1.166790	0.686633
H	5.141959	-0.621221	2.857422	H	-5.308022	-2.168948	-1.973938	H	5.088935	-0.776294	2.817295
H	2.914971	-1.268794	1.758600	H	-2.997423	-1.995769	-0.866700	H	2.882095	-1.353070	1.643727
C	3.946774	-1.859585	-0.787673	C	-3.885242	-1.048605	1.625934	C	3.956164	-1.839561	-0.902756
H	3.412076	-2.685621	-0.325573	H	-3.314792	-1.971977	1.683886	H	3.400402	-2.678325	-0.491274
H	3.733882	-1.853649	-1.853687	H	-3.632226	-0.423230	2.478365	H	3.768517	-1.782557	-1.971916
H	5.012170	-2.020805	-0.653678	H	-4.942113	-1.291607	1.682167	H	5.016021	-2.021087	-0.751485
H	-1.545979	-1.568341	0.547584	H	1.532422	-1.369511	-0.016446	H	-1.570826	-1.340815	0.472702
C	-1.452642	-2.775837	-1.218007	C	1.561919	-1.403953	2.123608	C	-1.495030	-2.665552	-1.207797
H	-2.296429	-3.406539	-0.954868	H	2.440118	-2.037611	2.201886	H	-2.379455	-3.238953	-0.947075
H	-1.449510	-2.648163	-2.300634	H	1.576306	-0.700830	2.956588	H	-1.436224	-2.608759	-2.294890
H	-0.549162	-3.309309	-0.938152	H	0.689214	-2.040706	2.233656	H	-0.630873	-3.218266	-0.851251
H	-12.659818	3.035274	1.349250	H	13.404618	0.818333	-2.106628	H	-12.732414	2.708527	1.757607
C	-0.323188	-0.542873	-0.872778	C	0.283597	0.221648	0.671192	C	-0.300686	-0.451109	-0.994366
H	-0.284845	-0.399103	-1.955031	H	0.265828	0.940922	1.493168	H	-0.260616	-0.349963	-2.081378
H	-0.471190	0.445056	-0.434993	H	0.355309	0.804512	-0.248080	H	-0.408660	0.557640	-0.593999
**α-Tocopherol (C17$^{\bullet}$)**				**α-Tocopherol (C18$^{\bullet}$)**				**α-Tocopherol (C19$^{\bullet}$)**			
C	3.539500	-0.515342	-0.136770	C	-3.557036	-0.287177	0.388903	C	3.538223	-0.424106	-0.230061
C	5.717980	0.514351	-0.201496	C	-5.784196	0.488907	-0.105483	C	5.737287	0.543068	-0.045557
C	6.090258	-0.102566	0.988204	C	-6.147458	-0.752363	-0.617344	C	6.107348	-0.381384	0.925658
C	5.068259	-0.830714	1.821995	C	-5.105495	-1.814514	-0.853027	C	5.076807	-1.288930	1.545821
C	3.650310	-0.498070	1.380679	C	-3.698054	-1.238415	-0.790143	C	3.662317	-0.822004	1.233284
C	6.642631	1.213856	-0.980754	C	-6.729499	1.496427	0.102812	C	6.671360	1.407013	-0.621987
C	7.420470	-0.046980	1.408646	C	-7.487620	-1.014541	-0.908752	C	7.443092	-0.471976	1.321585
H	5.238844	-1.905944	1.751854	H	-5.218341	-2.607338	-0.112050	H	5.221446	-2.306967	1.181412
H	3.381128	0.501917	1.717447	H	-3.487391	-0.671722	-1.695892	H	3.417984	0.053682	1.832817
C	8.333792	0.651909	0.636342	C	-8.421380	-0.011853	-0.705528	C	8.365634	0.389834	0.751714
C	7.963646	1.287896	-0.547148	C	-8.061466	1.240725	-0.211840	C	7.998420	1.331585	-0.207580
O	4.422369	0.494351	-0.662466	O	-4.480239	0.802650	0.198030	O	4.436694	0.675804	-0.472469
C	2.162542	-0.064443	-0.608583	C	-2.196363	0.398457	0.404094	C	2.166538	0.160460	-0.544660
H	2.221492	0.083178	-1.687060	H	-2.243712	1.195235	1.146681	H	2.224056	0.600352	-1.540460
H	1.964796	0.911794	-0.166581	H	-2.055921	0.877428	-0.564585	H	1.985314	0.978272	0.152364
C	1.013336	-1.015888	-0.292109	C	-1.007365	-0.506254	0.709293	C	1.005655	-0.827286	-0.497269

H	1.199851	-1.980427	-0.758031	H	-1.142606	-0.969420	1.683715	H	1.183777	-1.629918	-1.208345
H	0.946671	-1.186962	0.782571	H	-0.945714	-1.312015	-0.022528	H	0.934568	-1.283698	0.490162
C	-1.551252	-1.254909	-0.377322	C	1.565675	-0.571274	0.811347	C	-1.556275	-1.024274	-0.676683
C	-2.812336	-0.499356	-0.801171	C	2.797825	0.313974	0.614233	C	-2.815426	-0.169493	-0.836499
H	-2.827366	-0.424995	-1.891190	H	2.833921	1.051098	1.419982	H	-2.820509	0.263078	-1.839909
H	-2.746806	0.522024	-0.423676	H	2.675416	0.875682	-0.312917	H	-2.756311	0.667977	-0.140037
C	-4.124123	-1.111094	-0.320022	C	4.125532	-0.435157	0.556436	C	-4.129120	-0.908541	-0.601587
H	-4.077400	-1.261695	0.760666	H	4.056337	-1.234287	-0.184902	H	-4.092382	-1.412812	0.366594
H	-4.260550	-2.091284	-0.769723	H	4.323075	-0.909619	1.513992	H	-4.253634	-1.680915	-1.355600
C	-5.318235	-0.223872	-0.654313	C	5.284850	0.487600	0.194485	C	-5.326380	0.036127	-0.634671
H	-5.129896	0.780826	-0.274934	H	5.029576	1.029480	-0.717251	H	-5.135073	0.866914	0.045680
H	-5.410921	-0.135243	-1.739045	H	5.405672	1.239472	0.978221	H	-5.415687	0.468992	-1.634052
C	-6.654093	-0.708708	-0.089408	C	6.622322	-0.220718	-0.016056	C	-6.664698	-0.602660	-0.256625
H	-6.546037	-0.818254	0.996005	H	6.495851	-0.951635	-0.818357	H	-6.572354	-1.000274	0.757260
C	-7.730754	0.288131	-0.349272	C	7.685708	0.785613	-0.464720	C	-7.754228	0.471143	-0.247585
H	-7.563201	1.060972	-1.085745	H	7.271276	1.390350	-1.282500	H	-7.392749	1.338347	0.304599
C	-9.095870	0.131021	0.218027	H	7.876154	1.489286	0.350993	H	-7.927553	0.806058	-1.272570
H	-9.033351	-0.373524	1.186073	C	8.964033	0.162486	-0.895353	C	-9.083771	0.033388	0.366963
H	-9.694759	-0.526588	-0.421061	H	8.926338	-0.811580	-1.363744	H	-8.915248	-0.328156	1.384584
C	-9.824833	1.464591	0.382611	C	10.215402	0.954056	-1.015923	H	-9.478133	-0.823336	-0.189997
H	-9.193048	2.135876	0.964132	H	10.170270	1.592648	-1.908532	C	-10.089107	1.128607	0.389777
H	-9.951084	1.927183	-0.598870	H	10.299393	1.642135	-0.169397	H	-10.103170	1.833669	-0.429194
C	-11.191379	1.361092	1.058905	C	11.484886	0.103412	-1.091730	C	-11.214237	1.137730	1.365629
H	-11.049345	0.888624	2.032661	H	11.363209	-0.603333	-1.914285	H	-10.795151	1.022761	2.369870
C	-11.768205	2.756370	1.280485	C	12.698827	0.979515	-1.379416	C	-11.990239	2.449821	1.306512
H	-11.091623	3.373451	1.867080	H	12.581317	1.522364	-2.314423	H	-11.337695	3.297778	1.497145
H	-11.929741	3.250028	0.322816	H	12.831576	1.708438	-0.580640	H	-12.430541	2.575794	0.318531
C	-7.042711	-2.090224	-0.643902	C	7.084338	-0.952873	1.241005	C	-7.019814	-1.751369	-1.197885
H	-7.078185	-2.057020	-1.731695	H	7.129343	-0.257271	2.079190	H	-7.057750	-1.389988	-2.225638
H	-6.329923	-2.853063	-0.343326	H	6.413068	-1.763467	1.508271	H	-6.285865	-2.550031	-1.147345
H	-8.022703	-2.387946	-0.281310	H	8.078355	-1.369516	1.098397	H	-7.986648	-2.182429	-0.956413
C	-12.170452	0.514784	0.249461	C	11.688407	-0.683052	0.199178	C	-12.161008	-0.051938	1.133173
H	-12.280011	0.929006	-0.752681	H	11.812693	0.004458	1.035494	H	-12.611288	0.019973	0.144874
H	-11.840338	-0.515535	0.155823	H	10.833709	-1.319659	0.413211	H	-11.628776	-0.997179	1.197685
H	-13.151561	0.509703	0.718647	H	12.577739	-1.306288	0.140817	H	-12.957774	-0.055990	1.874112
C	9.017610	2.035353	-1.321630	C	-9.138072	2.279154	-0.032875	C	9.061727	2.241023	-0.766162
H	9.812074	1.367260	-1.657424	H	-9.883345	1.957422	0.696207	H	9.833376	1.675865	-1.290907
H	8.605978	2.510873	-2.201363	H	-8.732828	3.219209	0.316224	H	8.650042	2.952348	-1.469418
H	9.470612	2.818735	-0.713199	H	-9.651319	2.477943	-0.974068	H	9.543895	2.813761	0.026850
C	6.200873	1.874531	-2.260860	C	-6.298461	2.830994	0.653673	C	6.233930	2.399937	-1.667613
H	5.156782	1.667614	-2.450408	H	-5.241133	2.821504	0.879262	H	5.184817	2.270369	-1.894367
H	6.329289	2.953832	-2.207875	H	-6.485500	3.628427	-0.062764	H	6.383178	3.421177	-1.322974
H	6.781448	1.513334	-3.106669	H	-6.841695	3.070629	1.564958	H	6.801376	2.273991	-2.586782
C	7.854013	-0.722510	2.680326	C	-7.909579	-2.355151	-1.443754	C	7.872502	-1.477807	2.353845
H	7.491107	-0.180940	3.552979	H	-7.585994	-2.482320	-2.475961	H	7.528412	-1.189471	3.346183
H	7.459474	-1.733764	2.736577	H	-7.468836	-3.160981	-0.862402	H	7.456939	-2.458367	2.136317
H	8.933731	-0.767379	2.739196	H	-8.986759	-2.455553	-1.415169	H	8.951452	-1.557179	2.383044
O	9.634435	0.704490	1.083827	O	-9.732151	-0.292660	-1.017783	O	9.672262	0.290281	1.173089
H	10.171893	1.172340	0.444906	H	-10.282559	0.453040	-0.778703	H	10.212448	0.909700	0.682773
H	5.199255	-0.571137	2.869658	H	-5.270275	-2.279622	-1.821945	H	5.223845	-1.327682	2.622425
H	2.945358	-1.200982	1.815503	H	-2.965964	-2.037424	-0.714917	H	2.946065	-1.603534	1.470494
C	3.926510	-1.864041	-0.731271	C	-3.865271	-0.963610	1.719006	C	3.895594	-1.566042	-1.173845
H	3.378923	-2.668626	-0.246812	H	-3.290153	-1.879555	1.827149	H	3.342892	-2.466073	-0.916595
H	3.701514	-1.874568	-1.794782	H	-3.618539	-0.293017	2.538142	H	3.657181	-1.285841	-2.196797
H	4.989610	-2.046119	-0.605909	H	-4.920926	-1.209489	1.784859	H	4.957392	-1.786743	-1.119480
H	-1.557189	-1.333502	0.712854	H	1.552778	-1.311210	0.006862	H	-1.559841	-1.441551	0.333496
C	-1.518469	-2.664168	-0.963618	C	1.613953	-1.309870	2.146487	C	-1.532983	-2.178049	-1.676276
H	-2.384834	-3.243013	-0.658211	H	2.501971	-1.929630	2.226204	H	-2.388035	-2.834500	-1.547461
H	-1.511616	-2.612753	-2.052567	H	1.626227	-0.592961	2.967666	H	-1.555292	-1.788178	-2.694040
H	-0.634018	-3.208076	-0.645518	H	0.751938	-1.957469	2.276043	H	-0.638436	-2.783551	-1.566252
H	-12.723545	2.708301	1.797672	H	13.606242	0.383508	-1.441754	H	-12.794909	2.461361	2.037463
C	-0.315859	-0.450091	-0.784906	C	0.300887	0.279980	0.686281	C	-0.317709	-0.136941	-0.816305
H	-0.291637	-0.369690	-1.874143	H	0.291855	1.018604	1.491039	H	-0.281474	0.259183	-1.833677
H	-0.422520	0.565979	-0.402957	H	0.351358	0.841239	-0.247596	H	-0.428466	0.722656	-0.154075
**α-Tocopherol (C20$^{\bullet}$)**				**α-Tocopherol (C21$^{\bullet}$)**				**α-Tocopherol (C22$^{\bullet}$)**			
C	3.543918	-0.400253	-0.290029	C	3.541641	-0.440280	-0.328075	C	3.546251	-0.398611	-0.296892
C	5.748127	0.532937	-0.010495	C	5.718494	0.555529	-0.035877	C	5.749201	0.538485	-0.019540
C	6.109176	-0.479110	0.872734	C	6.110649	-0.456731	0.833749	C	6.115515	-0.477189	0.857356
C	5.071362	-1.432211	1.405824	C	5.103325	-1.451278	1.349159	C	5.081759	-1.435733	1.388503
C	3.660195	-0.932833	1.130602	C	3.677905	-0.988014	1.084890	C	3.668561	-0.938189	1.120593
C	6.689037	1.441197	-0.502264	C	6.628808	1.504682	-0.507541	C	6.686215	1.451951	-0.509106
C	7.443185	-0.614291	1.261687	C	7.446838	-0.551043	1.227543	C	7.450994	-0.610822	1.241778
H	5.214013	-2.415531	0.955213	H	5.273940	-2.421329	0.880029	H	5.225310	-2.416260	0.932142
H	3.415519	-0.115847	1.807796	H	3.412820	-0.187595	1.774139	H	3.424319	-0.125258	1.802806
C	8.372476	0.291086	0.776525	C	8.345405	0.395022	0.762835	C	8.376377	0.299747	0.758903

C	8.014011	1.320334	-0.092191	C	7.954758	1.425185	-0.090798	C	8.012645	1.332575	-0.103356
O	4.449514	0.711943	-0.427057	O	4.416715	0.697083	-0.456147	O	4.448921	0.716274	-0.431383
C	2.176682	0.219635	-0.552177	C	2.157917	0.147945	-0.576043	C	2.176679	0.219493	-0.551131
H	2.232254	0.733832	-1.511808	H	2.193270	0.668509	-1.533233	H	2.227505	0.738462	-1.508444
H	2.008638	0.983723	0.206455	H	1.979483	0.904216	0.188021	H	2.009764	0.979505	0.211835
C	1.005818	-0.757142	-0.569699	C	1.008063	-0.853410	-0.587760	C	1.008088	-0.759961	-0.568802
H	1.172602	-1.511263	-1.334776	H	1.184141	-1.604575	-1.353993	H	1.172909	-1.509144	-1.339146
H	0.933270	-1.279486	0.384388	H	0.950904	-1.376946	0.366808	H	0.941637	-1.288189	0.382472
C	-1.558865	-0.913184	-0.722096	C	-1.557109	-1.047503	-0.689808	C	-1.556554	-0.922435	-0.704797
C	-2.811982	-0.043768	-0.845939	C	-2.823989	-0.211135	-0.882914	C	-2.813686	-0.057070	-0.815397
H	-2.827911	0.410426	-1.839523	H	-2.890881	0.089362	-1.931352	H	-2.840545	0.399079	-1.807841
H	-2.736002	0.777713	-0.132206	H	-2.727536	0.708967	-0.304912	H	-2.734093	0.763176	-0.100626
C	-4.127072	-0.779059	-0.606775	C	-4.118551	-0.904806	-0.470244	C	-4.123660	-0.797835	-0.565283
H	-4.078382	-1.302837	0.350271	H	-4.042137	-1.211879	0.574861	H	-4.064201	-1.322127	0.390927
H	-4.266292	-1.535752	-1.373867	H	-4.256511	-1.810543	-1.054768	H	-4.266780	-1.554604	-1.331587
C	-5.321037	0.170417	-0.601014	C	-5.330313	0.006669	-0.640013	C	-5.321931	0.146067	-0.548090
H	-5.119612	0.980004	0.101911	H	-5.111268	0.967730	-0.172473	H	-5.116216	0.959080	0.149646
H	-5.419049	0.633183	-1.585978	H	-5.484946	0.208426	-1.702487	H	-5.434217	0.604882	-1.533388
C	-6.656350	-0.477099	-0.227484	C	-6.633240	-0.533415	-0.047426	C	-6.648952	-0.507359	-0.156141
H	-6.540230	-0.943558	0.754043	H	-6.466840	-0.716669	1.017121	H	-6.520678	-0.962385	0.829298
C	-7.733069	0.603513	-0.110956	C	-7.729615	0.525631	-0.177920	C	-7.734252	0.564957	-0.041704
H	-7.368752	1.385314	0.557059	H	-7.352309	1.466536	0.225340	H	-7.366941	1.363441	0.604976
H	-7.868621	1.071256	-1.089129	H	-7.923605	0.700321	-1.238902	H	-7.890921	1.012362	-1.026110
C	-9.082047	0.113300	0.404215	C	-9.040979	0.188247	0.524511	C	-9.068837	0.071108	0.506165
H	-8.940864	-0.420422	1.345217	H	-8.838520	-0.060998	1.568334	H	-8.901254	-0.450454	1.450458
H	-9.512468	-0.594271	-0.300322	H	-9.487453	-0.692304	0.070000	H	-9.515176	-0.652071	-0.173826
C	-10.058081	1.268306	0.619121	C	-10.026634	1.349795	0.464878	C	-10.057033	1.208992	0.732807
H	-9.588988	2.020359	1.255873	H	-9.538655	2.246600	0.848342	H	-9.628031	1.928119	1.431259
H	-10.216437	1.765915	-0.349750	H	-10.285344	1.556195	-0.575153	H	-10.220440	1.743730	-0.205655
C	-11.379561	0.892611	1.204549	C	-11.320913	1.127199	1.256332	C	-11.410826	0.738754	1.265811
C	-12.231573	1.993957	1.733890	H	-11.027640	0.892559	2.289155	H	-11.239720	0.166984	2.180456
H	-11.638647	2.733188	2.268298	C	-12.143781	2.363732	1.269135	C	-12.287006	1.950182	1.634345
H	-12.745748	2.524443	0.922277	H	-11.675728	3.333383	1.247558	H	-11.794696	2.557945	2.391016
C	-7.055215	-1.556295	-1.231308	C	-7.043943	-1.848845	-0.704648	H	-12.457718	2.566973	0.754655
H	-7.177268	-1.115847	-2.221166	H	-7.207826	-1.696081	-1.771643	C	-7.048964	-1.600168	-1.144585
H	-6.303442	-2.336755	-1.299262	H	-6.277086	-2.608540	-0.585865	H	-7.170637	-1.173145	-2.140339
H	-7.992018	-2.030341	-0.955135	H	-7.960780	-2.241341	-0.275091	H	-6.298098	-2.382390	-1.201897
C	-12.044514	-0.370553	0.776917	C	-12.129708	-0.058911	0.727917	H	-7.986528	-2.068462	-0.861466
H	-12.337957	-0.328807	-0.279896	H	-12.357814	0.086112	-0.326528	C	-12.114104	-0.126583	0.282257
H	-11.392640	-1.234428	0.885571	H	-11.588053	-0.993322	0.838108	H	-11.985974	0.044863	-0.774499
H	-12.947084	-0.550861	1.354799	H	-13.069014	-0.149983	1.267555	H	-12.880439	-0.814430	0.595775
C	9.084662	2.271753	-0.558811	C	8.992313	2.423025	-0.534259	C	9.079379	2.289392	-0.567853
H	9.848795	1.755947	-1.142380	H	9.774516	1.947463	-1.128119	H	9.843320	1.778810	-1.156263
H	8.678855	3.055994	-1.183300	H	8.559717	3.206268	-1.141789	H	8.669682	3.075741	-1.187122
H	9.575128	2.753975	0.287206	H	9.464895	2.902964	0.323013	H	9.570842	2.768475	0.279352
C	6.260651	2.528259	-1.453690	C	6.166635	2.591162	-1.443771	C	6.252145	2.542880	-1.453512
H	5.214047	2.419832	-1.701888	H	5.128063	2.444827	-1.706019	H	5.205215	2.432668	-1.699540
H	6.404849	3.512667	-1.012709	H	6.265276	3.572238	-0.983100	H	6.394590	3.525317	-1.007586
H	6.837725	2.489984	-2.374700	H	6.754972	2.594116	-2.358403	H	6.826932	2.511297	-2.376199
C	7.863370	-1.713305	2.198390	C	7.901784	-1.649444	2.148562	C	7.876876	-1.713725	2.171307
H	7.516872	-1.515394	3.211849	H	7.551788	-1.475812	3.165307	H	7.532964	-1.522171	3.186868
H	7.443347	-2.667377	1.890394	H	7.510339	-2.611521	1.828308	H	7.458381	-2.667223	1.859471
H	8.941777	-1.800967	2.223655	H	8.982428	-1.704323	2.171041	H	8.955574	-1.798714	2.192836
O	9.677091	0.145828	1.191231	O	9.652049	0.290088	1.183294	O	9.682543	0.156019	1.169273
H	10.227727	0.783549	0.737379	H	10.184329	0.946788	0.734556	H	10.230433	0.796500	0.715974
H	5.213491	-1.566375	2.475492	H	5.250725	-1.600985	2.415989	H	5.227600	-1.575357	2.456964
H	2.940671	-1.730636	1.291188	H	2.981359	-1.807678	1.236314	H	2.951441	-1.738474	1.279502
C	3.897520	-1.452141	-1.334265	C	3.917202	-1.469348	-1.387096	C	3.898596	-1.444439	-1.347605
H	3.334363	-2.366565	-1.166362	H	3.375192	-2.398450	-1.230689	H	3.337580	-2.360705	-1.182627
H	3.669033	-1.074174	-2.327482	H	3.678088	-1.082997	-2.374522	H	3.666295	-1.061796	-2.338138
H	4.956803	-1.688014	-1.294440	H	4.981727	-1.681215	-1.352474	H	4.958446	-1.678535	-1.312244
H	-1.561863	-1.365472	0.272909	H	-1.562894	-1.435881	0.331865	H	-1.550530	-1.379079	0.288216
C	-1.549192	-2.032257	-1.760857	C	-1.514731	-2.228148	-1.657218	C	-1.550751	-2.037000	-1.748467
H	-2.418339	-2.675349	-1.662589	H	-2.410228	-2.837617	-1.582979	H	-2.417089	-2.683325	-1.646776
H	-1.553834	-1.607359	-2.764799	H	-1.436802	-1.864588	-2.681865	H	-1.564089	-1.607719	-2.750467
H	-0.668060	-2.659382	-1.664006	H	-0.663155	-2.873311	-1.462303	H	-0.666899	-2.661683	-1.660829
H	-13.002059	1.616916	2.402881	H	-13.210593	2.314051	1.407635	H	-13.252185	1.632522	2.018728
C	-0.312015	-0.034062	-0.835456	C	-0.324535	-0.155207	-0.845939	C	-0.313186	-0.039079	-0.823277
H	-0.284325	0.411867	-1.832210	H	-0.321185	0.267710	-1.853173	H	-0.293385	0.410856	-1.818404
H	-0.403422	0.792353	-0.129507	H	-0.415464	0.685931	-0.157711	H	-0.402223	0.784250	-0.113427

Structures of α-Tocopherol in the SET-PT (SET_step) mechanism at M05-2X/6-311++G(2d,2p) level of theory in gas phase.

M05-2X/6-311++G(2d,2p)			
α-Tocopherol $^{\bullet+}$			
C	3.61562300	-0.70332800	-0.36363800
C	5.73924300	0.49103200	-0.14835500
C	6.20744800	-0.44450300	0.82315500
C	5.25569500	-1.47198900	1.37254800
C	3.81080100	-1.08785400	1.08736500
C	6.55239500	1.57137000	-0.64450300
C	7.50794200	-0.35715500	1.23729100
H	5.48335400	-2.44716300	0.94449000
H	3.51667500	-0.24422100	1.70993200
C	8.31028000	0.69794600	0.72091600
C	7.84444900	1.66264700	-0.20741500
O	4.53940000	0.42768400	-0.65668800
C	2.24538500	-0.10536000	-0.63588300
H	2.24047700	0.26595800	-1.66010900
H	2.11312800	0.75441100	0.01980600
C	1.08246000	-1.07625700	-0.44416200
H	1.23491900	-1.96118700	-1.05677600
H	1.03149000	-1.40538200	0.59359100
C	-1.48389300	-1.23134600	-0.45847600
C	-2.73606400	-0.42367000	-0.80649300
H	-2.78028300	-0.29382700	-1.89031900
H	-2.63507500	0.57548300	-0.37995300
C	-4.04818100	-1.02768900	-0.31613900
H	-3.97707100	-1.22407400	0.75553500
H	-4.21939500	-1.98419300	-0.80226400
C	-5.22948200	-0.09882800	-0.58022700
H	-4.99873500	0.88382200	-0.16624600
H	-5.34617100	0.03949500	-1.65769700
C	-6.56356500	-0.57043500	0.00185700
H	-6.42594100	-0.72885700	1.07449600
C	-7.61390300	0.52710700	-0.18139000
H	-7.21778000	1.45808200	0.22699900
H	-7.76096800	0.69683300	-1.25075300
C	-8.96292100	0.24489400	0.47252100
H	-8.80937200	-0.01036100	1.52320300
H	-9.42617400	-0.61822100	0.00216000
C	-9.90186300	1.44282800	0.37694000
H	-9.39200800	2.32190700	0.77400400
H	-10.11147400	1.65499500	-0.67427000
C	-11.22971700	1.27377400	1.11546100
H	-11.00672900	1.06349700	2.16315500
C	-12.03442100	2.56817600	1.04352000
H	-11.47316700	3.40493600	1.45288400
H	-12.27710400	2.80017000	0.00707900
C	-7.01515500	-1.88615700	-0.62730900
H	-7.17658500	-1.75083400	-1.69685700
H	-6.27639900	-2.67084100	-0.49264100
H	-7.94388400	-2.23760200	-0.18885200
C	-12.05196900	0.11619600	0.55548400
H	-12.22887300	0.26522600	-0.50957600
H	-11.55419000	-0.84007800	0.68653200
H	-13.01884600	0.05909700	1.04957900
C	8.80020900	2.72611100	-0.66610900
H	9.68190100	2.28424200	-1.13013700
H	8.34656300	3.37699800	-1.40007800
H	9.12258000	3.34971400	0.16782100
C	5.97128800	2.54307900	-1.62843200
H	4.91981800	2.35733200	-1.79097800
H	6.09135700	3.56059500	-1.26731300
H	6.48532800	2.46547400	-2.58385800
C	8.13245900	-1.29969700	2.21866500
H	8.27401000	-0.80654000	3.17873600
H	7.52467900	-2.18186300	2.36536500
H	9.11407600	-1.60536900	1.87120300
O	9.55136100	0.74353900	1.17894600
H	10.04423700	1.48417200	0.80874500
H	5.40465700	-1.55813500	2.44424700
H	3.15116200	-1.91666000	1.32165700
C	3.98254700	-1.80630300	-1.34074600
H	3.41120400	-2.70083600	-1.11247700
H	3.75518300	-1.49595500	-2.35591200
H	5.03900100	-2.05201200	-1.28345400

H	-1.47853500	-1.39436500	0.62214900
C	-1.47997500	-2.59038000	-1.15387900
H	-2.36961300	-3.16042500	-0.90747300
H	-1.45424600	-2.45665700	-2.23538400
H	-0.62187200	-3.19100200	-0.86428400
H	-12.96804100	2.48083300	1.59381300
C	-0.24171500	-0.41396500	-0.81771800
H	-0.25094000	-0.21036900	-1.89040800
H	-0.30481000	0.55250200	-0.31627800

Structures of α-Tocopherol in the SET-PT (PT_step) mechanism at M05-2X/6-311++G(2d,2p) level of theory in gas phase.

M05-2X/6-311++G(2d,2p)											
**α-Tocopherol $^{•+}$**				**α-Tocopherol (O$^•$)**				**α-Tocopherol (C1$^•$)**			
C	3.61562300	-0.70332800	-0.36363800	C	3.656534	-0.526742	-0.329553	C	3.651911	-0.686793	-0.360377
C	5.73924300	0.49103200	-0.14835500	C	5.808538	0.534811	-0.047938	C	5.772694	0.516798	-0.169971
C	6.20744800	-0.44450300	0.82315500	C	6.241276	-0.479760	0.824437	C	6.244292	-0.399044	0.818513
C	5.25569500	-1.47198900	1.37254800	C	5.259935	-1.505660	1.328283	C	5.295737	-1.418387	1.388229
C	3.81080100	-1.08785400	1.08736500	C	3.826309	-1.055865	1.084677	C	3.849572	-1.043368	1.097412
C	6.55239500	1.57137000	-0.64450300	C	6.663781	1.570265	-0.504540	C	6.582569	1.589637	-0.687298
C	7.50794200	-0.35715500	1.23729100	C	7.557684	-0.501527	1.216453	C	7.544987	-0.300599	1.229519
H	5.48335400	-2.44716300	0.94449000	H	5.442902	-2.463522	0.841113	H	5.525444	-2.400887	0.978404
H	3.51667500	-0.24422100	1.70993200	H	3.565638	-0.253867	1.773961	H	3.553936	-0.188883	1.704266
C	8.31028000	0.69794600	0.72091600	C	8.478945	0.524487	0.760869	C	8.344092	0.746599	0.692456
C	7.84444900	1.66264700	-0.20741500	C	7.973140	1.576349	-0.113562	C	7.874837	1.692423	-0.253410
O	4.53940000	0.42768400	-0.65668800	O	4.542707	0.611759	-0.502338	O	4.572491	0.440825	-0.675718
C	2.24538500	-0.10536000	-0.63588300	C	2.273543	0.067982	-0.558233	C	2.279868	-0.097545	-0.642374
H	2.24047700	0.26595800	-1.66010900	H	2.294767	0.581641	-1.519275	H	2.272952	0.254362	-1.673419
H	2.11312800	0.75441100	0.01980600	H	2.108886	0.829280	0.203847	H	2.146093	0.774110	-0.002884
C	1.08246000	-1.07625700	-0.44416200	C	1.121635	-0.931217	-0.544614	C	1.119627	-1.067596	-0.431106
H	1.23491900	-1.96118700	-1.05677600	H	1.286025	-1.690199	-1.305623	H	1.273712	-1.963541	-1.027075
H	1.03149000	-1.40538200	0.59359100	H	1.074528	-1.444656	0.415920	H	1.070572	-1.377204	0.612728
C	-1.48389300	-1.23134600	-0.45847600	C	-1.444981	-1.117773	-0.622833	C	-1.446335	-1.229433	-0.439711
C	-2.73606400	-0.42367000	-0.80649300	C	-2.709044	-0.281611	-0.835190	C	-2.700925	-0.431646	-0.801552
H	-2.78028300	-0.29382700	-1.89031900	H	-2.779867	-0.015052	-1.892449	H	-2.746597	-0.322395	-1.887587
H	-2.63507500	0.57548300	-0.37995300	H	-2.605611	0.656247	-0.287807	H	-2.602048	0.575632	-0.394053
C	-4.04818100	-1.02768900	-0.31613900	C	-4.004613	-0.955292	-0.394070	C	-4.010986	-1.029627	-0.298467
H	-3.97707100	-1.22407400	0.75553500	H	-3.930165	-1.216212	0.663515	H	-3.938266	-1.205569	0.776645
H	-4.21939500	-1.98419300	-0.80226400	H	-4.143069	-1.885525	-0.938630	H	-4.180259	-1.995567	-0.766269
C	-5.22948200	-0.09882800	-0.58022700	C	-5.214373	-0.050072	-0.606031	C	-5.194929	-0.108914	-0.578757
H	-4.99873500	0.88382200	-0.16624600	H	-4.996592	0.928706	-0.175979	H	-4.966266	0.881957	-0.183644
H	-5.34617100	0.03949500	-1.65769700	H	-5.362311	0.109639	-1.676627	H	-5.313086	0.008752	-1.658519
C	-6.56356500	-0.57043500	0.00185700	C	-6.521909	-0.564470	-0.000809	C	-6.527200	-0.572831	0.013567
H	-6.42594100	-0.72885700	1.07449600	H	-6.362300	-0.707863	1.070888	H	-6.388062	-0.710630	1.088855
C	-7.61390300	0.52710700	-0.18139000	C	-7.614104	0.492115	-0.178154	C	-7.580528	0.518391	-0.189222
H	-7.21778000	1.45808200	0.22699900	H	-7.241367	1.443326	0.204800	H	-7.186363	1.457909	0.201096
H	-7.76096800	0.69683300	-1.25075300	H	-7.790327	0.637714	-1.246691	H	-7.729133	0.667530	-1.261437
C	-8.96292100	0.24489400	0.47252100	C	-8.938222	0.176630	0.510452	C	-8.928143	0.245151	0.471359
H	-8.80937200	-0.01036100	1.52320300	H	-8.754143	-0.048039	1.563189	H	-8.772855	0.010162	1.526504
H	-9.42617400	-0.61822100	0.00216000	H	-9.379273	-0.713595	0.069783	H	-9.389676	-0.627860	0.017872
C	-9.90186300	1.44282800	0.37694000	C	-9.920484	1.338622	0.406790	C	-9.870241	1.438683	0.354205
H	-9.39200800	2.32190700	0.77400400	H	-9.434122	2.243327	0.774612	H	-9.362224	2.326390	0.734056
H	-10.11147400	1.65499500	-0.67427000	H	-10.155244	1.519947	-0.644955	H	-10.081481	1.630440	-0.700594
C	-11.22971700	1.27377400	1.11546100	C	-11.229305	1.142688	1.172135	C	-11.196894	1.280233	1.097222
H	-11.00672900	1.06349700	2.16315500	H	-10.982264	0.962743	2.220011	H	-10.972286	1.090333	2.148456
C	-12.03442100	2.56817600	1.04352000	C	-12.077598	2.408032	1.086152	C	-12.004977	2.571002	1.001738
H	-11.47316700	3.40493600	1.45288400	H	-11.538660	3.270759	1.470742	H	-11.445439	3.416759	1.394630
H	-12.27710400	2.80017000	0.00707900	H	-12.340841	2.610974	0.048599	H	-12.249324	2.782777	-0.038633
C	-7.01515500	-1.88615700	-0.62730900	C	-6.932764	-1.902578	-0.610144	C	-6.976078	-1.901334	-0.590168
H	-7.17658500	-1.75083400	-1.69685700	H	-7.097697	-1.788502	-1.681830	H	-7.138959	-1.786632	-1.661904
H	-6.27639900	-2.67084100	-0.49264100	H	-6.166575	-2.658256	-0.464433	H	-6.235180	-2.681461	-0.441516
H	-7.94388400	-2.23760200	-0.18885200	H	-7.849535	-2.278263	-0.165859	H	-7.903451	-2.246794	-0.144151
C	-12.05196900	0.11619600	0.55548400	C	-12.021885	-0.053186	0.651120	C	-12.016765	0.110211	0.560081
H	-12.22887300	0.26522600	-0.50957600	H	-12.221920	0.067590	-0.413455	H	-12.195151	0.238663	-0.507410
H	-11.55419000	-0.84007800	0.68653200	H	-11.488839	-0.988737	0.791796	H	-11.516409	-0.842154	0.708612
H	-13.01884600	0.05909700	1.04957900	H	-12.977587	-0.132801	1.163916	H	-12.982981	0.059997	1.056212
C	8.80020900	2.72611100	-0.66610900	C	8.945159	2.629961	-0.554681	C	8.827401	2.749459	-0.733126
H	9.68190100	2.28424200	-1.13013700	H	9.042163	2.643418	-1.638201	H	9.709739	2.301148	-1.189687
H	8.34656300	3.37699800	-1.40007800	H	8.617293	3.618280	-0.240119	H	9.149041	3.389506	0.088538
H	9.12258000	3.34971400	0.16782100	H	9.914548	2.426693	-0.118862	C	5.997963	2.541126	-1.688760

C	5.97128800	2.54307900	-1.62843200	C	6.072895	2.610656	-1.413863	H	4.946803	2.349679	-1.846633
H	4.91981800	2.35733200	-1.79097800	H	5.705691	2.152335	-2.329025	H	6.115805	3.565577	-1.347037
H	6.09135700	3.56059500	-1.26731300	H	5.221740	3.092502	-0.939776	H	6.511211	2.446805	-2.643107
H	6.48532800	2.46547400	-2.58385800	H	6.803926	3.365504	-1.671316	C	8.172925	-1.222864	2.227828
C	8.13245900	-1.29969700	2.21866500	C	8.083408	-1.561054	2.132732	H	8.314209	-0.711317	3.178268
H	8.27401000	-0.80654000	3.17873600	H	7.735610	-1.395709	3.152606	H	7.567554	-2.103642	2.391808
H	7.52467900	-2.18186300	2.36536500	H	7.743463	-2.547700	1.827831	H	9.154960	-1.532550	1.885134
H	9.11407600	-1.60536900	1.87120300	H	9.165467	-1.532463	2.137239	O	9.585525	0.803975	1.148200
O	9.55136100	0.74353900	1.17894600	O	9.671904	0.506797	1.117861	H	10.076124	1.538736	0.763554
H	10.04423700	1.48417200	0.80874500	H	5.421793	-1.667638	2.390582	H	5.446027	-1.483913	2.461201
H	5.40465700	-1.55813500	2.44424700	H	3.136989	-1.878187	1.251434	H	3.192295	-1.869274	1.348017
H	3.15116200	-1.91666000	1.32165700	C	4.018029	-1.559514	-1.387771	C	4.020640	-1.807079	-1.316893
C	3.98254700	-1.80630300	-1.34074600	H	3.474271	-2.484865	-1.218512	H	3.451821	-2.698590	-1.071165
H	3.41120400	-2.70083600	-1.11247700	H	3.767915	-1.178580	-2.374204	H	3.791434	-1.516524	-2.337488
H	3.75518300	-1.49595500	-2.35591200	H	5.082018	-1.775873	-1.364336	H	5.077777	-2.048984	-1.256118
H	5.03900100	-2.05201200	-1.28345400	H	-1.453227	-1.486388	0.406083	H	-1.439442	-1.372013	0.643791
H	-1.47853500	-1.39436500	0.62214900	C	-1.404033	-2.316418	-1.568014	C	-1.439664	-2.601336	-1.109348
C	-1.47997500	-2.59038000	-1.15387900	H	-2.307923	-2.913111	-1.493588	H	-2.327587	-3.168882	-0.851265
H	-2.36961300	-3.16042500	-0.90747300	H	-1.309753	-1.972731	-2.598087	H	-1.415396	-2.487984	-2.193211
H	-1.45424600	-2.45665700	-2.23538400	H	-0.563298	-2.968918	-1.351117	H	-0.579730	-3.194207	-0.809399
H	-0.62187200	-3.19100200	-0.86428400	H	-13.000769	2.301396	1.650845	H	-12.937801	2.491694	1.554592
H	-12.96804100	2.48083300	1.59381300	C	-0.210980	-0.230478	-0.795362	C	-0.206622	-0.415831	-0.815660
C	-0.24171500	-0.41396500	-0.81771800	H	-0.214780	0.184138	-1.805992	H	-0.217477	-0.232542	-1.891990
H	-0.25094000	-0.21036900	-1.89040800	H	-0.293579	0.616347	-0.113163	H	-0.271667	0.559765	-0.332479
H	-0.30481000	0.55250200	-0.31627800								
**α-Tocopherol (C2$^{\bullet}$)**				**α-Tocopherol (C3$^{\bullet}$)**				**α-Tocopherol (C4$^{\bullet}$)**			
C	3.625550	-0.462827	-0.331833	C	3.627547	-0.418429	-0.234518	C	-3.634109	-0.280028	0.316778
C	5.784836	0.566871	-0.069365	C	5.833904	0.512324	0.018033	C	-5.826372	0.591124	-0.123883
C	6.208606	-0.421517	0.798226	C	6.196018	-0.480041	0.903518	C	-6.212341	-0.618520	-0.756260
C	5.227059	-1.431194	1.333752	C	5.165001	-1.411152	1.477052	C	-5.221946	-1.530234	-1.127833
C	3.792633	-0.985715	1.086821	C	3.750633	-0.923344	1.194605	C	-3.785376	-1.252014	-0.848887
C	6.678421	1.562020	-0.564105	C	6.771033	1.422855	-0.508872	C	-6.748665	1.555043	0.244800
C	7.556806	-0.482371	1.173775	C	7.564535	-0.631086	1.271058	C	-7.591242	-0.851010	-0.998452
H	5.403592	-2.401836	0.868749	H	5.309571	-2.412617	1.068394	H	-5.488479	-2.431869	-1.650029
H	3.532398	-0.177669	1.768949	H	3.504463	-0.094980	1.857140	H	-3.302149	-0.815346	-1.729152
C	8.440931	0.494395	0.703624	C	8.490693	0.296973	0.736231	C	-8.495345	0.117233	-0.621564
C	8.030567	1.525397	-0.117787	C	8.110018	1.310620	-0.126029	C	-8.103253	1.317532	-0.011749
O	4.496307	0.676560	-0.499342	O	4.535781	0.690197	-0.395018	O	-4.500464	0.853663	0.087401
C	2.236212	0.117184	-0.564476	C	2.260798	0.199311	-0.503394	C	-2.240956	0.333995	0.362052
H	2.254577	0.625177	-1.528796	H	2.314188	0.696962	-1.471877	H	-2.210916	1.032840	1.198048
H	2.066590	0.882731	0.192257	H	2.095381	0.976174	0.242737	H	-2.115196	0.922318	-0.546124
C	1.090529	-0.888730	-0.545888	C	1.089175	-0.776742	-0.501652	C	-1.092166	-0.661448	0.483646
H	1.260483	-1.651496	-1.301971	H	1.253334	-1.542427	-1.255588	H	-1.248014	-1.307841	1.343771
H	1.045799	-1.396847	0.417683	H	1.019544	-1.284359	0.460579	H	-1.061034	-1.304317	-0.396383
C	-1.475794	-1.090772	-0.621228	C	-1.475472	-0.936202	-0.651013	C	1.474065	-0.853412	0.561577
C	-2.744962	-0.264281	-0.840713	C	-2.729022	-0.068576	-0.783874	C	2.743545	0.002696	0.607968
H	-2.819278	-0.010441	-1.900853	H	-2.742156	0.379427	-1.780321	H	2.782119	0.516286	1.571654
H	-2.645620	0.680398	-0.304422	H	-2.656079	0.757340	-0.074962	H	2.667981	0.779571	-0.153913
C	-4.035791	-0.940146	-0.389108	C	-4.044049	-0.804004	-0.545119	C	4.047233	-0.761625	0.399296
H	-3.958148	-1.186707	0.671682	H	-3.998021	-1.322862	0.414748	H	4.003121	-1.302325	-0.548580
H	-4.168685	-1.878385	-0.921249	H	-4.179220	-1.564803	-1.308777	H	4.159571	-1.506111	1.181775
C	-5.251792	-0.045736	-0.610952	C	-5.239886	0.143179	-0.548857	C	5.259456	0.163945	0.404452
H	-5.039229	0.940264	-0.194979	H	-5.045347	0.953998	0.154605	H	5.091942	0.969272	-0.312407
H	-5.402938	0.098301	-1.683351	H	-5.331883	0.604550	-1.535042	H	5.343793	0.634847	1.386924
C	-6.554871	-0.559769	0.004180	C	-6.576205	-0.507527	-0.184579	C	6.591185	-0.512810	0.070894
H	-6.391686	-0.688127	1.077256	H	-6.464548	-0.977080	0.796069	H	6.503848	-0.951484	-0.926313
C	-7.653668	0.488017	-0.184022	C	-7.656359	0.570131	-0.070030	C	7.697322	0.542418	0.025856
H	-7.286185	1.445730	0.187606	H	-7.300937	1.347070	0.608320	H	7.382458	1.347116	-0.640816
H	-7.831941	0.620370	-1.253936	H	-7.782649	1.045571	-1.045631	H	7.797962	0.983942	1.020073
C	-8.975141	0.172437	0.509620	C	-9.010267	0.071569	0.425739	C	9.056551	0.025316	-0.434644
H	-8.788421	-0.040554	1.564333	H	-8.872354	-0.476912	1.360003	H	8.942477	-0.491164	-1.390293
H	-9.411783	-0.724700	0.078581	H	-9.427999	-0.629074	-0.292514	H	9.427115	-0.707878	0.277036
C	-9.963959	1.327895	0.395560	C	-9.994613	1.213870	0.653553	C	10.079616	1.146006	-0.583979
H	-9.481944	2.239034	0.753091	H	-9.531902	1.952010	1.310388	H	9.668566	1.916512	-1.237831
H	-10.201447	1.496984	-0.657607	H	-10.186824	1.720865	-0.295101	H	10.244006	1.617695	0.387633
C	-11.270450	1.132783	1.165081	C	-11.331920	0.790167	1.260819	C	11.429366	0.698212	-1.143934
H	-11.020718	0.965268	2.214358	H	-11.125926	0.285310	2.206359	H	11.252033	0.250099	-2.123034
C	-12.126017	2.392334	1.067218	C	-12.188683	2.020137	1.546724	C	12.347133	1.904419	-1.322029
H	-11.591217	3.262127	1.441552	H	-11.671106	2.716682	2.202250	H	11.881020	2.666916	-1.941765
H	-12.392236	2.582744	0.028067	H	-12.417433	2.540004	0.617144	H	12.571227	2.349520	-0.353822
C	-6.959276	-1.908176	-0.586375	C	-6.967426	-1.584505	-1.193904	C	6.920895	-1.626959	1.060911
H	-7.128328	-1.809078	-1.658911	H	-7.091389	-1.140096	-2.181756	H	7.009210	-1.217893	2.068071
H	-6.187543	-2.656706	-0.433500	H	-6.210812	-2.359979	-1.264936	H	6.148003	-2.389731	1.073379
H	-7.872173	-2.283887	-0.134151	H	-7.901442	-2.065720	-0.920930	H	7.857313	-2.116617	0.811522
C	-12.057073	-0.072970	0.657978	C	-12.091102	-0.175029	0.354072	C	12.102291	-0.341611	-0.250944

H	-12.258865	0.035130	-0.407630	H	-12.242215	0.276496	-0.626420	H	12.218276	0.053698	0.758227
H	-11.518713	-1.003980	0.808139	H	-11.558721	-1.111544	0.217219	H	11.529037	-1.261897	-0.190098
H	-13.011783	-0.152259	1.172710	H	-13.069039	-0.404255	0.771048	H	13.091588	-0.588675	-0.629283
C	8.983749	2.603764	-0.552818	C	9.167133	2.258751	-0.630427	C	-9.163802	2.321544	0.346065
H	9.351055	2.439806	-1.565453	H	9.913987	1.738577	-1.231558	H	-9.885547	1.905725	1.050549
H	8.490385	3.570513	-0.535663	H	8.742952	3.038171	-1.248578	H	-8.738624	3.205374	0.803626
H	9.838333	2.689316	0.112812	H	9.680425	2.749275	0.197143	H	-9.710493	2.650316	-0.539553
C	6.214587	2.518353	-1.462877	C	6.321834	2.494384	-1.463603	C	-6.288177	2.830958	0.899025
H	6.866809	3.264341	-1.875743	H	5.270263	2.383751	-1.687841	H	-5.215923	2.812110	1.039281
H	5.188210	2.508402	-1.775405	H	6.477109	3.485159	-1.039693	H	-6.535435	3.695365	0.286536
C	8.044655	-1.571658	2.084456	H	6.879907	2.444527	-2.396227	H	-6.759552	2.966977	1.869223
H	7.735459	-1.386650	3.113249	C	8.005499	-1.647085	2.115260	C	-8.038433	-2.121100	-1.658831
H	7.636287	-2.535239	1.791158	H	7.324305	-2.371615	2.520189	H	-7.594910	-2.212658	-2.649428
H	9.124914	-1.629646	2.066688	H	9.046056	-1.727751	2.365276	H	-7.727651	-2.989618	-1.081063
O	9.748157	0.380719	1.123788	O	9.794324	0.141122	1.115764	H	-9.115262	-2.142407	-1.758114
H	10.306804	0.925750	0.570810	H	10.337454	0.795446	0.675415	O	-9.828343	-0.127050	-0.862177
H	5.391057	-1.569751	2.399528	H	5.311301	-1.494873	2.551693	H	-10.343984	0.639554	-0.613763
H	3.105564	-1.808585	1.261658	H	3.035197	-1.720933	1.372973	H	-3.253943	-2.174950	-0.624450
C	3.990057	-1.504971	-1.381110	C	3.976455	-1.490044	-1.259767	C	-4.009808	-0.926438	1.643047
H	3.456313	-2.434535	-1.201051	H	3.407481	-2.397612	-1.075537	H	-3.363366	-1.773564	1.853561
H	3.732674	-1.135511	-2.370199	H	3.750792	-1.128990	-2.259904	H	-3.905630	-0.196362	2.441829
H	5.056405	-1.709332	-1.360423	H	5.033805	-1.732780	-1.213587	H	-5.036030	-1.279638	1.622679
H	-1.481752	-1.450519	0.410902	H	-1.477743	-1.377555	0.348882	H	1.453077	-1.376721	-0.397622
C	-1.427872	-2.297637	-1.555658	C	-1.465890	-2.066616	-1.677394	C	1.454602	-1.894583	1.679374
H	-2.328547	-2.898874	-1.476956	H	-2.332798	-2.711026	-1.568987	H	2.299255	-2.572480	1.611161
H	-1.334134	-1.962328	-2.588562	H	-1.475092	-1.653002	-2.685996	H	1.495003	-1.400896	2.650623
H	-0.583224	-2.942746	-1.332178	H	-0.582779	-2.690233	-1.576409	H	0.553510	-2.499293	1.646829
H	-13.047602	2.286598	1.634711	H	-13.129795	1.743222	2.015878	H	13.288564	1.615930	-1.783425
C	-0.246583	-0.198424	-0.801559	C	-0.229218	-0.057517	-0.774801	C	0.247144	0.057671	0.622059
H	-0.253402	0.208372	-1.815428	H	-0.202294	0.376707	-1.776753	H	0.261640	0.608430	1.565296
H	-0.333569	0.653461	-0.126190	H	-0.320824	0.776996	-0.078471	H	0.328406	0.800486	-0.172698
**α-Tocopherol (C5$^{\bullet}$)**				**α-Tocopherol (C6$^{\bullet}$)**				**α-Tocopherol (C7$^{\bullet}$)**			
C	3.574499	0.044926	0.204548	C	-3.616201	-0.355131	0.320077	C	3.624913	-0.702641	-0.370598
C	5.813971	-0.383681	-0.523132	C	-5.787467	0.598254	-0.046631	C	5.748540	0.492190	-0.158009
C	6.102267	0.984227	-0.582627	C	-6.221673	-0.555027	-0.690208	C	6.217113	-0.441694	0.814911
C	4.986376	2.000459	-0.617736	C	-5.242084	-1.630868	-1.082129	C	5.265596	-1.468310	1.366333
C	3.651988	1.413278	-0.354964	C	-3.798728	-1.160143	-0.959345	C	3.820588	-1.084730	1.080982
C	6.821757	-1.350972	-0.480671	C	-6.673463	1.618164	0.304209	C	6.561471	1.571740	-0.656229
C	7.424846	1.410319	-0.586681	C	-7.579304	-0.717353	-0.972053	C	7.517738	-0.353584	1.228474
H	5.204809	2.807063	0.086544	H	-5.399078	-2.504120	-0.447811	H	5.493167	-2.444187	0.939832
H	2.786174	1.829709	-0.841785	H	-3.534875	-0.513481	-1.794409	H	3.526620	-0.240072	1.702234
C	8.423319	0.448196	-0.561475	C	-8.456745	0.297472	-0.625963	C	8.319850	0.700694	0.710072
C	8.147652	-0.920275	-0.495969	C	-8.023707	1.464775	0.000035	C	7.853663	1.663817	-0.219718
O	4.509329	-0.813841	-0.509036	O	-4.458985	0.810619	0.246752	O	4.548534	0.427928	-0.665842
C	2.214643	-0.598976	-0.047645	C	-2.206901	0.238733	0.429996	C	2.254554	-0.105202	-0.643393
H	2.316319	-1.665354	0.146817	H	-2.187545	0.862831	1.323114	H	2.122466	0.755657	0.010901
H	2.001710	-0.496357	-1.111865	H	-2.065918	0.899110	-0.425478	C	1.091744	-1.075839	-0.449667
C	1.063714	-0.032476	0.777888	C	-1.078041	-0.782754	0.495205	H	1.244050	-1.961784	-1.060851
H	1.273141	-0.163134	1.838061	H	-1.286932	-1.503828	1.283436	H	1.041132	-1.403231	0.588651
H	0.965027	1.035705	0.606851	H	-1.018649	-1.338584	-0.440606	C	-1.474605	-1.231088	-0.462881
C	-1.471843	0.013835	1.087724	C	1.483163	-1.011572	0.599414	C	-2.726933	-0.424062	-0.811837
C	-2.758014	-0.734074	0.711481	C	2.761557	-0.201133	0.824124	H	-2.771515	-0.296034	-1.895864
H	-2.755407	-1.698903	1.225142	H	2.828167	0.063793	1.882072	H	-2.625858	0.575808	-0.387002
H	-2.739747	-0.950872	-0.356928	H	2.682683	0.738570	0.275733	C	-4.038857	-1.027330	-0.320044
C	-4.041780	0.015582	1.046243	C	4.045966	-0.903021	0.394198	H	-3.967385	-1.221919	0.751934
H	-3.978542	1.028723	0.642161	H	3.975585	-1.158703	-0.664997	H	-4.210179	-1.984655	-0.804512
H	-4.128647	0.121193	2.125548	H	4.155680	-1.838329	0.936705	C	-5.220295	-0.098975	-0.585297
C	-5.290959	-0.678191	0.511039	C	5.275668	-0.028760	0.620450	H	-4.989465	0.884379	-0.173036
H	-5.088734	-1.029578	-0.501484	H	5.082562	0.959384	0.199986	H	-5.337344	0.037540	-1.662959
H	-5.497720	-1.568320	1.105328	H	5.423795	0.116111	1.693135	C	-6.554161	-0.569679	-0.001988
C	-6.532703	0.217780	0.477012	C	6.572319	-0.567413	0.012962	H	-6.416177	-0.726299	1.070869
H	-6.319879	1.054070	-0.193124	H	6.412064	-0.693504	-1.060849	C	-7.604618	0.527500	-0.186726
C	-7.716092	-0.550387	-0.097663	C	7.690013	0.459286	0.205448	H	-7.208412	1.459177	0.219976
H	-7.427000	-0.984986	-1.055500	H	7.339814	1.425271	-0.161417	H	-7.752043	0.695429	-1.256323
H	-7.946748	-1.392099	0.560233	H	7.871572	0.583454	1.275763	C	-8.953407	0.246309	0.468099
C	-8.974336	0.292113	-0.294636	C	9.004466	0.122810	-0.491558	H	-8.799500	-0.007181	1.519156
H	-8.703253	1.208916	-0.823986	H	8.812386	-0.078828	-1.547530	H	-9.416768	-0.617616	-0.000666
H	-9.353570	0.590803	0.677299	H	9.424140	-0.785899	-0.067740	C	-9.892444	1.444031	0.370822
C	-10.066614	-0.462381	-1.048114	C	10.016136	1.257642	-0.370446	H	-9.382507	2.323800	0.766249
H	-9.630353	-0.886639	-1.954386	H	9.550337	2.181677	-0.716250	H	-10.102411	1.654429	-0.680672
H	-10.393173	-1.318584	-0.452298	H	10.261762	1.411070	0.683230	C	-11.220047	1.276142	1.110060
C	-11.296268	0.347257	-1.483381	C	11.314975	1.045703	-1.148350	H	-10.996704	1.067629	2.158031
H	-10.942362	1.174525	-2.100707	H	11.057263	0.896566	-2.198447	C	-12.024844	2.570379	1.036219
C	-12.199772	-0.516705	-2.350568	C	12.196194	2.286391	-1.038654	H	-11.463501	3.407852	1.443999
H	-11.647522	-0.892875	-3.209723	H	11.677204	3.171511	-1.399053	H	-12.267880	2.800626	-0.000529

H	-12.555392	-1.368324	-1.774265	H	12.471139	2.457921	0.001470	C	-7.005887	-1.886475	-0.628805
C	-6.860748	0.791444	1.855060	C	6.948010	-1.922948	0.606224	H	-7.167675	-1.752949	-1.698524
H	-7.094677	-0.020526	2.544750	H	7.110444	-1.827166	1.680106	H	-6.267045	-2.670893	-0.493067
H	-6.014838	1.339730	2.256253	H	6.163918	-2.657185	0.447098	H	-7.934453	-2.237236	-0.189456
H	-7.714969	1.461164	1.817823	H	7.857528	-2.314856	0.161030	C	-12.042420	0.117585	0.552290
C	-12.096286	0.931311	-0.320943	C	12.079110	-0.182138	-0.660127	H	-12.219682	0.264825	-0.512960
H	-12.492565	0.133975	0.309272	H	12.286513	-0.092404	0.406115	H	-11.544547	-0.838442	0.684773
H	-11.491763	1.589535	0.296232	H	11.521747	-1.100185	-0.820676	H	-13.009132	0.061261	1.046796
H	-12.938621	1.508187	-0.697373	H	13.030337	-0.273636	-1.179258	C	8.809216	2.726563	-0.680503
C	9.314664	-1.873795	-0.428117	C	-9.042028	2.524732	0.329988	H	9.690779	2.283966	-1.144081
H	9.948348	-1.654032	0.433964	H	-9.786713	2.154679	1.036467	H	8.355294	3.376198	-1.415411
H	8.998144	-2.900824	-0.329586	H	-8.582628	3.396247	0.776195	H	9.131826	3.351577	0.152277
H	9.936007	-1.809021	-1.320494	H	-9.565074	2.858718	-0.566684	C	5.979989	2.541772	-1.641591
C	6.448711	-2.804243	-0.392468	C	-6.161997	2.856807	0.993073	H	4.928476	2.355697	-1.803482
H	5.370768	-2.906979	-0.403812	H	-5.103048	2.767628	1.192231	H	6.100122	3.559896	-1.282214
H	6.861187	-3.358936	-1.232800	H	-6.317610	3.739550	0.375993	H	6.493720	2.462596	-2.597055
H	6.819402	-3.247805	0.529131	H	-6.676365	3.018223	1.937523	C	8.142628	-1.294451	2.211218
C	7.768268	2.869987	-0.639960	C	-8.079893	-1.964196	-1.647534	H	8.284467	-0.799681	3.170416
H	7.109729	3.380265	-1.336047	H	-7.779600	-1.987882	-2.694392	H	7.534943	-2.176402	2.359592
H	7.636834	3.339254	0.336577	H	-7.672991	-2.852655	-1.171519	H	9.124147	-1.600651	1.863946
H	8.795565	3.018834	-0.951960	H	-9.160184	-2.013109	-1.607042	O	9.561079	0.747119	1.167618
O	9.728309	0.878743	-0.589710	O	-9.786888	0.118140	-0.931086	H	10.053794	1.487158	0.796018
H	10.293654	0.102754	-0.584391	H	-10.293039	0.854967	-0.589453	H	5.414914	-1.552656	2.438126
H	4.971964	2.487307	-1.596151	H	-5.439954	-1.953253	-2.101790	H	3.161070	-1.913178	1.316876
C	3.925155	-0.004757	1.690305	H	-3.123797	-2.011512	-0.964323	C	3.991576	-1.807229	-1.345980
H	3.215423	0.604004	2.238759	C	-3.971608	-1.144382	1.529794	H	3.420356	-2.701409	-1.116028
H	3.872761	-1.030252	2.042350	H	-3.861447	-2.214603	1.549396	H	3.763863	-1.498591	-2.361589
H	4.921385	0.375414	1.888145	H	-4.259876	-0.624286	2.426604	H	5.048062	-2.052786	-1.288624
H	-1.525362	1.018628	0.660420	H	1.497303	-1.385421	-0.427601	H	-1.468884	-1.392300	0.618014
C	-1.318047	0.114311	2.605175	C	1.404428	-2.205352	1.548591	C	-1.470842	-2.591283	-1.156011
H	-2.236445	0.432785	3.086137	H	2.294381	-2.824439	1.486920	H	-2.360368	-3.160963	-0.908361
H	-1.049725	-0.868922	3.000466	H	1.306145	-1.855492	2.576246	H	-1.445475	-2.459367	-2.237747
H	-0.541246	0.817684	2.891682	H	0.549718	-2.836931	1.324335	H	-0.612612	-3.191374	-0.865694
H	-13.062713	0.035649	-2.707697	H	13.112722	2.169542	-1.612097	H	-12.958279	2.483907	1.586963
C	-0.269488	-0.703405	0.466272	C	0.263470	-0.101550	0.750630	C	-0.232589	-0.414243	-0.823896
H	-0.255995	-1.730452	0.840120	H	0.264589	0.325773	1.755907	H	-0.242176	-0.212442	-1.896922
H	-0.405773	-0.755561	-0.615723	H	0.367005	0.735440	0.058753	H	-0.295571	0.553058	-0.324052
**α-Tocopherol (C8$^{\bullet}$)**				**α-Tocopherol (C9$^{\bullet}$)**				**α-Tocopherol (C10$^{\bullet}$)**			
C	3.509250	-0.935440	0.370474	C	3.586592	-0.618903	-0.198641	C	-3.593679	-0.281211	0.299388
C	5.670538	-0.066599	-0.228005	C	5.707117	0.526327	-0.196823	C	-5.832234	0.519983	-0.093155
C	5.336026	1.198879	0.243484	C	6.103942	-0.124935	0.966341	C	-6.213847	-0.696529	-0.649065
C	3.915706	1.518727	0.633974	C	5.116510	-0.944083	1.756298	C	-5.181775	-1.747780	-0.964027
C	2.948808	0.456739	0.131232	C	3.686366	-0.662608	1.319091	C	-3.771694	-1.176656	-0.917509
C	6.975164	-0.378601	-0.617175	C	6.598008	1.310444	-0.933791	C	-6.767962	1.518672	0.187230
C	6.325934	2.177208	0.357630	C	7.426691	-0.018930	1.400572	C	-7.563029	-0.944317	-0.910013
H	3.845589	1.604113	1.719335	H	5.341918	-2.004630	1.635764	H	-5.273425	-2.573136	-0.256464
H	2.794481	0.571388	-0.940959	H	3.366668	0.307661	1.695947	H	-3.586393	-0.569188	-1.802284
C	7.617481	1.866239	-0.034479	C	8.306317	0.763411	0.670449	C	-8.487732	0.049194	-0.633579
C	7.954456	0.606788	-0.527098	C	7.910174	1.434431	-0.485021	C	-8.109571	1.278837	-0.097872
O	4.736880	-1.068019	-0.364987	O	4.417958	0.457864	-0.671467	O	-4.519186	0.817445	0.186624
C	2.597999	-2.010164	-0.236970	C	2.191296	-0.218767	-0.662250	C	-2.231082	0.400922	0.308768
H	3.123298	-2.961234	-0.122155	H	2.251608	-0.004899	-1.728947	H	-2.250715	1.151449	1.099135
H	2.526674	-1.808492	-1.304890	H	1.925121	0.712222	-0.164727	H	-2.122375	0.937001	-0.633904
C	1.238956	-2.076972	0.361505	C	1.098934	-1.259633	-0.423922	C	-1.037172	-0.522921	0.519043
H	1.130744	-2.381219	1.390677	H	1.359750	-2.180712	-0.956402	H	-1.133517	-1.054208	1.464288
C	-1.291930	-1.978899	0.299930	H	1.047069	-1.532467	0.632599	H	-0.997061	-1.276937	-0.267143
C	-2.448330	-1.296093	-0.430051	C	-1.495813	-1.423405	-0.405612	C	1.517587	-0.643094	0.674882
H	-2.498058	-1.686989	-1.449242	C	-2.712402	-0.594381	-0.821845	C	2.787492	0.212849	0.564125
H	-2.227393	-0.231417	-0.517485	H	-2.736957	-0.535763	-1.912162	H	2.815809	0.902725	1.410263
C	-3.809564	-1.458037	0.238711	H	-2.573877	0.424107	-0.458363	H	2.726747	0.823655	-0.337275
H	-3.743748	-1.120934	1.275500	C	-4.048749	-1.126491	-0.315509	C	4.071763	-0.607023	0.534618
H	-4.080413	-2.510293	0.265178	H	-3.988683	-1.290180	0.762571	H	4.049154	-1.278491	-0.326235
C	-4.894556	-0.664037	-0.482470	H	-4.251251	-2.092605	-0.770310	H	4.106889	-1.236409	1.422188
H	-4.542567	0.358882	-0.623447	C	-5.193647	-0.163534	-0.613506	C	5.313858	0.274087	0.456864
H	-5.041452	-1.080520	-1.481928	H	-4.938684	0.814648	-0.203615	H	5.177770	1.000036	-0.346460
C	-6.245369	-0.617177	0.234008	H	-5.283098	-0.031165	-1.694525	H	5.405634	0.849965	1.380838
H	-6.085590	-0.181126	1.223430	C	-6.553671	-0.588123	-0.055472	C	6.623325	-0.478330	0.213511
C	-7.199593	0.298182	-0.535602	H	-6.434689	-0.775358	1.014738	H	6.522949	-1.030977	-0.724079
H	-6.690829	1.242833	-0.732922	C	-7.556481	0.555158	-0.224161	C	7.767210	0.524239	0.049050
H	-7.405518	-0.150496	-1.510440	H	-7.129888	1.458123	0.214704	H	7.466087	1.278099	-0.679939
C	-8.518007	0.597936	0.170731	H	-7.680493	0.758602	-1.290584	H	7.910856	1.050489	0.995763
H	-8.310717	0.999455	1.164935	C	-8.924802	0.307842	0.403445	C	9.094441	-0.079983	-0.398672
H	-9.078814	-0.322194	0.312958	H	-8.795025	0.015561	1.447767	H	8.936884	-0.659484	-1.310724
C	-9.365846	1.596761	-0.610040	H	-9.413524	-0.523171	-0.098174	H	9.458492	-0.771978	0.356314
H	-8.754756	2.469895	-0.844088	C	-9.818846	1.541407	0.333371	C	10.147642	0.991948	-0.659533

H	-9.653283	1.155399	-1.567555	H	-9.280149	2.390880	0.755818	H	9.730673	1.734787	-1.341344
C	-10.629768	2.066009	0.110363	H	-10.014414	1.788033	-0.713093	H	10.367322	1.517182	0.273151
H	-10.327942	2.517430	1.057308	C	-11.155913	1.401101	1.061072	C	11.458903	0.472648	-1.249039
C	-11.342713	3.125692	-0.724281	H	-10.945506	1.159567	2.104640	H	11.226132	-0.045410	-2.181183
H	-10.685790	3.965470	-0.938917	C	-11.916765	2.723049	1.015750	C	12.387557	1.641248	-1.565636
H	-11.664208	2.698849	-1.673687	H	-11.327916	3.531458	1.442963	H	11.914729	2.347712	-2.244015
C	-6.832817	-2.015267	0.412086	H	-12.149273	2.984788	-0.016011	H	12.643246	2.173497	-0.650197
H	-6.984069	-2.481706	-0.561625	C	-7.053166	-1.869899	-0.718207	C	6.909771	-1.475312	1.333735
H	-6.174061	-2.653070	0.993927	H	-7.215375	-1.697671	-1.782357	H	6.992575	-0.950302	2.285582
H	-7.790266	-1.983076	0.923081	H	-6.338015	-2.680072	-0.612212	H	6.117641	-2.212614	1.423250
C	-11.579281	0.908956	0.408845	H	-7.990431	-2.204585	-0.284075	H	7.838019	-2.011816	1.161973
H	-11.839658	0.392779	-0.514999	C	-12.013809	0.284789	0.471500	C	12.156910	-0.509745	-0.312374
H	-11.138486	0.185275	1.088328	H	-12.177111	0.462317	-0.591427	H	12.333725	-0.039595	0.654921
H	-12.499472	1.273906	0.859254	H	-11.549148	-0.690183	0.585058	H	11.567829	-1.407510	-0.149925
C	9.379794	0.356137	-0.945380	H	-12.985869	0.250516	0.957973	H	13.119566	-0.810668	-0.719170
H	10.064328	0.444524	-0.100290	C	8.926886	2.274900	-1.212554	C	-9.177227	2.310831	0.156142
H	9.506655	-0.635495	-1.358142	H	9.754017	1.666835	-1.582189	H	-9.898308	1.963384	0.897773
H	9.694672	1.066963	-1.709863	H	8.493235	2.778159	-2.065914	H	-8.758501	3.237007	0.525824
C	7.299170	-1.757916	-1.130063	H	9.339609	3.043955	-0.559019	H	-9.721291	2.544287	-0.759372
H	6.427297	-2.394935	-1.076695	C	6.129638	2.006449	-2.185424	C	-6.316723	2.827929	0.780034
H	7.629701	-1.721219	-2.166280	H	5.100167	1.751186	-2.395134	H	-5.253630	2.806559	0.976316
H	8.094178	-2.214577	-0.545092	H	6.196490	3.087458	-2.079367	H	-6.520675	3.654695	0.103253
C	5.996969	3.546136	0.886285	H	6.735832	1.719372	-3.041622	H	-6.833499	3.031064	1.715925
H	5.436242	4.123684	0.152483	C	7.887499	-0.729949	2.643004	C	-8.004420	-2.260021	-1.489166
H	5.387494	3.478452	1.783604	H	7.495079	-0.247039	3.536966	H	-7.722111	-2.338763	-2.538263
H	6.901990	4.091245	1.120911	H	7.541911	-1.760516	2.650621	H	-7.540176	-3.090473	-0.963432
O	8.568837	2.855232	0.071118	H	8.967892	-0.725075	2.706583	H	-9.079542	-2.364876	-1.422617
H	9.425058	2.497765	-0.163305	O	9.598899	0.865126	1.132462	O	-9.808240	-0.215744	-0.917782
H	3.637157	2.490875	0.234779	H	10.119823	1.372077	0.509784	H	-10.350380	0.512472	-0.614781
H	1.983355	0.549175	0.622723	H	5.227982	-0.728474	2.816069	H	-5.376669	-2.168799	-1.947437
C	3.780258	-1.208834	1.843277	H	3.014062	-1.417238	1.717489	H	-3.040517	-1.979757	-0.901090
H	2.882952	-1.034199	2.431413	C	4.048966	-1.917497	-0.848598	C	-3.864485	-1.018293	1.605316
H	4.092877	-2.241762	1.971544	H	3.549930	-2.772404	-0.399530	H	-3.285255	-1.936587	1.656288
H	4.571542	-0.564568	2.214925	H	3.825679	-1.896570	-1.912317	H	-3.595800	-0.384818	2.446786
H	-1.244850	-1.575139	1.314099	H	5.120901	-2.042732	-0.727842	H	-4.917669	-1.269104	1.688968
C	-1.489705	-3.490515	0.383455	H	-1.474399	-1.471113	0.688862	H	1.515589	-1.358671	-0.149170
H	-2.350610	-3.752846	0.991009	C	-1.589560	-2.871106	-0.918147	C	1.487726	-1.387222	1.962962
H	-1.641702	-3.897563	-0.616285	H	-2.441492	-3.387405	-0.484668	H	1.599116	-0.843226	2.887944
H	-0.615883	-3.974435	0.811691	H	-1.695592	-2.875641	-2.001791	H	1.311381	-2.446480	2.015641
H	-12.224157	3.502862	-0.211289	H	-0.693978	-3.430188	-0.661377	H	13.312355	1.295634	-2.021538
C	0.031262	-1.653389	-0.395024	H	-12.854010	2.655934	1.563197	C	0.274956	0.252940	0.521072
H	0.034297	-2.119706	-1.386807	C	-0.235818	-0.775803	-0.867503	H	0.266418	0.979810	1.335080
H	0.076214	-0.574404	-0.589876	H	-0.283731	-0.047082	-1.663753	H	0.364276	0.817807	-0.407564
**α-Tocopherol (C11$^{\bullet}$)**				**α-Tocopherol (C12$^{\bullet}$)**				**α-Tocopherol (C13$^{\bullet}$)**			
C	-3.542262	0.522882	-0.257494	C	3.519588	0.497889	-0.215602	C	-3.567543	-0.161305	0.258032
C	-5.721156	-0.504410	-0.177003	C	5.708836	-0.262327	0.446031	C	-5.875432	0.444186	-0.083153
C	-6.013678	0.114174	1.033920	C	6.010390	-0.656913	-0.853352	C	-6.228272	-0.877684	-0.333610
C	-4.938436	0.844274	1.796032	C	4.937624	-0.679824	-1.910924	C	-5.164808	-1.929293	-0.514819
C	-3.552381	0.509144	1.263775	C	3.549242	-0.571827	-1.297144	C	-3.791286	-1.306171	-0.719384
C	-6.695611	-1.203402	-0.893423	C	6.681530	-0.250917	1.448287	C	-6.842752	1.440861	0.067641
C	-7.313402	0.059868	1.541362	C	7.316304	-1.029845	-1.177219	C	-7.576824	-1.230084	-0.414023
H	-5.112505	1.919375	1.732858	H	5.100144	0.137790	-2.614702	H	-5.149871	-2.587014	0.355420
H	-3.261763	-0.489826	1.585290	H	3.275261	-1.516836	-0.830618	H	-3.712827	-0.898512	-1.726243
C	-8.275980	-0.639037	0.831574	C	8.276688	-1.024473	-0.179267	C	-8.533108	-0.238240	-0.271615
C	-7.985172	-1.275619	-0.373765	C	7.977504	-0.646870	1.128488	C	-8.186165	1.091658	-0.040628
O	-4.458704	-0.486355	-0.722345	O	4.439729	0.110760	0.822537	O	-4.563576	0.847852	0.003195
C	-2.200742	0.070315	-0.821147	C	2.173206	0.543984	0.497129	C	-2.253437	0.562825	-0.006368
H	-2.329181	-0.059221	-1.896147	H	2.296321	1.167500	1.382741	H	-2.239039	1.449061	0.628709
H	-1.982229	-0.914088	-0.408173	H	1.947288	-0.462794	0.847523	H	-2.270659	0.914243	-1.037680
C	-1.029019	1.009987	-0.561430	C	1.012172	1.076005	-0.336320	C	-0.989549	-0.254406	0.237219
H	-1.255429	1.997703	-0.956176	H	1.221582	2.099649	-0.637111	H	-0.992200	-0.643660	1.252582
H	-0.858496	1.121614	0.508413	H	0.900314	0.491275	-1.249660	H	-0.958799	-1.112680	-0.434452
C	1.501747	1.227242	-0.891261	C	-1.530147	1.443496	-0.361567	C	1.572115	-0.194362	0.040097
C	2.785282	0.551548	-1.254560	C	-2.776591	1.213414	0.422431	C	2.752234	0.748414	-0.213223
H	2.951930	0.636497	-2.338755	H	-2.701765	1.008460	1.480501	H	2.840746	1.440574	0.629003
H	2.684541	-0.517287	-1.060433	C	-4.122529	1.438634	-0.167380	H	2.516515	1.374276	-1.084364
C	4.034117	1.067700	-0.540108	H	-4.096766	1.205502	-1.235722	C	4.047352	0.051803	-0.425065
H	3.861300	1.059611	0.537201	H	-4.386755	2.499195	-0.102157	H	4.036792	-0.906770	-0.925583
H	4.215765	2.100432	-0.825239	C	-5.211054	0.607147	0.511581	C	5.347224	0.756384	-0.278927
C	5.261599	0.223232	-0.866707	H	-4.889808	-0.434109	0.528274	H	5.506766	1.427602	-1.133533
H	5.018443	-0.827151	-0.700676	H	-5.303516	0.921706	1.553427	H	5.315989	1.406886	0.600295
H	5.496694	0.322472	-1.929174	C	-6.585693	0.688788	-0.154321	C	6.553740	-0.179274	-0.162132
C	6.510064	0.565345	-0.051150	H	-6.479740	0.344077	-1.186038	H	6.531417	-0.860271	-1.016356
H	6.261585	0.445233	1.006332	C	-7.557627	-0.254478	0.558471	C	7.850194	0.626538	-0.232743
C	7.628944	-0.423725	-0.384902	H	-7.069994	-1.220487	0.696241	H	7.845434	1.215112	-1.151342

H	7.231499	-1.436703	-0.306350	H	-7.757002	0.136766	1.558964	H	7.864066	1.341612	0.593424
H	7.921855	-0.286348	-1.428444	C	-8.878328	-0.480500	-0.170622	C	9.124364	-0.211116	-0.193388
C	8.863081	-0.316014	0.505337	H	-8.672467	-0.846851	-1.178468	H	9.064451	-0.995004	-0.951666
H	8.556314	-0.377266	1.551737	H	-9.407291	0.462692	-0.280396	H	9.203770	-0.709585	0.769414
H	9.330740	0.655793	0.370365	C	-9.769822	-1.481938	0.556589	C	10.370379	0.632854	-0.438781
C	9.880691	-1.414584	0.215272	H	-9.189025	-2.380169	0.771685	H	10.239637	1.188549	-1.368538
H	9.376800	-2.381282	0.256978	H	-10.066278	-1.066664	1.522943	H	10.465689	1.377396	0.355526
H	10.253650	-1.303172	-0.805737	C	-11.029216	-1.888355	-0.208172	C	11.674382	-0.160261	-0.524403
C	11.072873	-1.443536	1.171276	H	-10.717684	-2.320565	-1.160909	H	11.562277	-0.905065	-1.314501
H	10.686160	-1.559848	2.185295	C	-11.795967	-2.949403	0.575623	C	12.825839	0.768833	-0.897318
C	11.967325	-2.638830	0.855325	H	-11.171033	-3.815273	0.782203	H	12.628616	1.282765	-1.835208
H	11.410761	-3.571841	0.906151	H	-12.130048	-2.540630	1.528622	H	12.963214	1.522956	-0.122966
H	12.374200	-2.544053	-0.150879	C	-7.113579	2.121628	-0.173431	C	6.464177	-1.002947	1.119595
C	6.950881	2.008767	-0.281942	H	-7.163488	2.511386	0.843375	H	6.576335	-0.351959	1.986871
H	7.179567	2.163791	-1.336515	H	-6.472783	2.777520	-0.755221	H	5.497802	-1.495364	1.193046
H	6.174352	2.712320	0.002521	H	-8.108826	2.174013	-0.604187	H	7.236587	-1.764836	1.165181
H	7.837788	2.252151	0.295114	C	-11.934498	-0.693288	-0.494683	C	11.989480	-0.885479	0.781137
C	11.885908	-0.153001	1.110051	H	-12.200982	-0.195157	0.437303	H	12.040046	-0.170218	1.601934
H	12.220105	0.028290	0.088588	H	-11.454731	0.034606	-1.142531	H	11.237164	-1.629429	1.026059
H	11.310240	0.708299	1.436009	H	-12.854492	-1.015722	-0.976760	H	12.950634	-1.390326	0.717506
H	12.767752	-0.224496	1.742633	C	9.078900	-0.678755	2.155759	C	-9.288792	2.110019	0.088586
C	-9.088050	-2.023656	-1.076259	H	9.885181	0.008587	1.895322	H	-9.925791	1.899297	0.948907
H	-9.904248	-1.356636	-1.357786	H	8.719978	-0.394880	3.135634	H	-8.896939	3.110007	0.215704
H	-8.735523	-2.497947	-1.982039	H	9.504253	-1.678916	2.244462	H	-9.917975	2.121636	-0.802120
H	-9.498152	-2.807831	-0.439119	C	6.317933	0.179746	2.845836	C	-6.423111	2.862578	0.337445
C	-6.340840	-1.865034	-2.199920	H	5.286312	0.501007	2.885528	H	-5.351407	2.920631	0.468043
H	-5.310689	-1.662657	-2.458235	H	6.446192	-0.637689	3.552616	H	-6.699855	3.515581	-0.488088
H	-6.469727	-2.943845	-2.139006	H	6.946500	1.003926	3.175085	H	-6.899794	3.245235	1.236657
H	-6.974312	-1.501073	-3.005588	C	7.672876	-1.440780	-2.579216	C	-7.985643	-2.656004	-0.661151
C	-7.660842	0.736989	2.838426	H	7.268744	-2.425158	-2.811645	H	-7.792166	-2.944088	-1.693762
H	-7.225149	0.206575	3.684142	H	7.264754	-0.740938	-3.303924	H	-7.427322	-3.335735	-0.022761
H	-7.277967	1.754156	2.859553	H	8.747099	-1.481615	-2.703918	H	-9.042212	-2.788911	-0.468732
H	-8.733508	0.767087	2.978079	O	9.551336	-1.414947	-0.522242	O	-9.854227	-0.611388	-0.372159
O	-9.544174	-0.692490	1.364139	H	10.128192	-1.314500	0.234708	H	-10.412173	0.150207	-0.215559
H	-10.123484	-1.153966	0.758057	H	5.013216	-1.597560	-2.489127	H	-5.414548	-2.558450	-1.365898
H	-4.999734	0.588650	2.851066	H	2.813920	-0.349303	-2.065117	H	-3.015231	-2.056785	-0.598390
H	-2.819854	1.213311	1.648460	C	3.925847	1.868101	-0.744536	C	-3.684799	-0.610595	1.709590
C	-3.966971	1.871262	-0.827194	H	3.348614	2.126880	-1.628751	H	-3.049992	-1.472300	1.898750
H	-3.395581	2.677819	-0.374832	H	3.756788	2.621839	0.020367	H	-3.387255	0.199399	2.370418
H	-3.804814	1.883783	-1.901950	H	4.979893	1.877619	-1.005998	H	-4.710403	-0.881580	1.942329
H	-5.021531	2.049203	-0.639200	H	-1.575042	0.821107	-1.263318	H	1.544708	-0.921462	-0.775056
C	1.461702	2.713030	-0.778410	C	-1.438876	2.905856	-0.829594	C	1.765089	-0.944864	1.354962
H	2.342217	3.102784	-0.277009	H	-2.345189	3.199040	-1.352076	H	2.743796	-1.416930	1.386733
H	1.421378	3.184702	-1.769396	H	-1.313506	3.563081	0.029413	H	1.700051	-0.249466	2.192061
H	0.587731	3.053113	-0.230578	H	-0.601351	3.054606	-1.505430	H	1.013318	-1.716146	1.495532
H	12.801990	-2.699940	1.549725	H	-12.674253	-3.283899	0.028524	H	13.758442	0.218674	-0.997794
C	0.247821	0.478914	-1.212369	C	-0.298070	1.026344	0.442755	C	0.261833	0.591036	0.018210
H	0.095927	0.466191	-2.301847	H	-0.222381	1.668605	1.322489	H	0.306010	1.370327	0.782638
H	0.380651	-0.566688	-0.929466	H	-0.452715	0.011549	0.809682	H	0.177323	1.103375	-0.941134
**α-Tocopherol (C14$^{\bullet}$)**				**α-Tocopherol (C15$^{\bullet}$)**				**α-Tocopherol (C16$^{\bullet}$)**			
C	3.593530	-0.703323	-0.367587	C	3.586799	-0.702337	-0.374920	C	3.539880	-0.425325	-0.254968
C	5.717003	0.491051	-0.150938	C	5.708825	0.491876	-0.143696	C	5.736983	0.537088	-0.026849
C	6.184430	-0.444252	0.821170	C	6.177048	-0.454167	0.817576	C	6.095082	-0.405098	0.931586
C	5.232228	-1.471583	1.370073	C	5.225873	-1.488931	1.354161	C	5.056924	-1.322605	1.523679
C	3.787565	-1.087492	1.083664	C	3.780851	-1.103191	1.071808	C	3.646224	-0.850168	1.202146
C	6.530564	1.571250	-0.646718	C	6.521282	1.578725	-0.626586	C	6.678171	1.410787	-0.576716
C	7.484601	-0.356825	1.236302	C	7.476995	-0.370091	1.234100	C	7.426210	-0.504232	1.340849
H	5.460203	-2.446868	0.942436	H	5.455103	-2.458896	0.915242	H	5.206020	-2.334403	1.143947
H	3.492967	-0.243699	1.705791	H	3.485095	-0.267055	1.703649	H	3.394134	0.014309	1.814614
C	8.287363	0.698132	0.720293	C	8.278687	0.691742	0.730684	C	8.355934	0.366708	0.796974
C	7.822277	1.662611	-0.208642	C	7.812759	1.666458	-0.187076	C	8.000275	1.326797	-0.148421
O	4.517557	0.427599	-0.660195	O	4.509604	0.432988	-0.654027	O	4.441488	0.678521	-0.466298
C	2.223516	-0.105396	-0.641053	C	2.216176	-0.102841	-0.641812	C	2.172541	0.166324	-0.574903
H	2.219417	0.265667	-1.665374	H	2.211949	0.280146	-1.661735	H	2.240449	0.619375	-1.564177
H	2.090763	0.754540	0.014319	H	2.082234	0.749236	0.023509	H	1.986841	0.975316	0.131148
C	1.060423	-1.076224	-0.450001	C	1.054150	-1.077172	-0.462460	C	1.008490	-0.818499	-0.551790
H	1.213345	-1.961309	-1.062275	H	1.208276	-1.954875	-1.084974	H	1.194854	-1.617395	-1.265028
H	1.008635	-1.405090	0.587794	H	1.002437	-1.418186	0.571410	H	0.919965	-1.280771	0.431677
C	-1.505921	-1.231268	-0.466285	C	-1.512008	-1.234976	-0.481342	C	-1.553736	-0.997424	-0.755835
C	-2.757804	-0.423655	-0.815482	C	-2.764721	-0.424788	-0.821477	C	-2.800938	-0.147402	-1.008081
H	-2.801172	-0.294080	-1.899375	H	-2.807920	-0.282624	-1.903797	H	-2.793733	0.186226	-2.048780
H	-2.657129	0.575602	-0.389112	H	-2.665329	0.569541	-0.383444	H	-2.737695	0.753163	-0.395547
C	-4.070315	-1.027527	-0.326005	C	-4.076678	-1.035845	-0.339484	C	-4.124476	-0.843554	-0.709530
H	-4.000048	-1.223647	0.745773	H	-4.006499	-1.244377	0.729954	H	-4.115377	-1.215907	0.315906
H	-4.241167	-1.984149	-0.812027	H	-4.246279	-1.986926	-0.836688	H	-4.244468	-1.705913	-1.360183

C	-5.251392	-0.098709	-0.591249	C	-5.258750	-0.105358	-0.594235	C	-5.310133	0.101041	-0.895617
H	-5.020950	0.884039	-0.177331	H	-5.029567	0.872758	-0.168807	H	-5.118182	1.024394	-0.345728
C	-6.585939	-0.570147	-0.010092	H	-5.374435	0.045131	-1.670181	H	-5.346324	0.399993	-1.954175
H	-6.449157	-0.728305	1.062694	C	-6.592921	-0.585084	-0.019030	C	-6.645456	-0.432139	-0.484900
C	-7.636112	0.527370	-0.194434	H	-6.456273	-0.755592	1.051880	C	-7.752615	0.569462	-0.404283
H	-7.240291	1.458439	0.214034	C	-7.644310	0.513296	-0.190875	H	-7.359295	1.489806	0.030750
H	-7.782337	0.696833	-1.263954	H	-7.249686	1.439991	0.228551	H	-8.074442	0.843787	-1.420045
C	-8.985647	0.245345	0.458491	H	-7.790416	0.695060	-1.258390	C	-8.987326	0.136995	0.385017
H	-8.832925	-0.009652	1.509357	C	-8.993709	0.222115	0.458299	H	-8.679444	-0.217815	1.369795
H	-9.448548	-0.617878	-0.012018	H	-8.841002	-0.044952	1.506164	H	-9.467418	-0.698814	-0.117963
C	-9.924491	1.443273	0.361878	H	-9.455473	-0.636093	-0.022393	C	-9.986070	1.277939	0.545573
H	-9.414930	2.322441	0.759122	C	-9.933910	1.420005	0.375382	H	-9.469076	2.142847	0.963790
H	-10.133275	1.655182	-0.689548	H	-9.425484	2.295064	0.783015	H	-10.352232	1.581768	-0.438233
C	-11.252926	1.274427	1.099401	H	-10.142630	1.643929	-0.673564	C	-11.184997	0.950356	1.434923
H	-11.030762	1.064406	2.147322	C	-11.262366	1.241032	1.110476	H	-10.804406	0.652745	2.413672
C	-12.057549	2.568827	1.026509	H	-11.040268	1.019053	2.155943	C	-12.058087	2.188927	1.613274
H	-11.496599	3.405678	1.436104	C	-12.068464	2.535265	1.052445	H	-11.486767	3.017255	2.025833
H	-12.299416	2.800568	-0.010179	H	-11.508604	3.367925	1.471951	H	-12.460971	2.504036	0.651302
C	-7.037061	-1.886016	-0.639284	H	-12.310295	2.778808	0.018457	C	-6.968656	-1.869215	-0.712096
H	-7.197652	-1.750956	-1.708992	C	-7.042336	-1.894042	-0.663673	H	-7.211076	-2.060318	-1.766116
H	-6.298426	-2.670681	-0.503843	H	-6.302833	-2.679380	-0.537170	H	-6.133483	-2.514985	-0.457024
H	-7.966140	-2.237335	-0.201467	H	-7.971136	-2.251517	-0.230271	H	-7.826910	-2.185888	-0.127029
C	-12.074761	0.116726	0.539069	C	-12.082696	0.089001	0.536419	C	-12.017522	-0.198637	0.872015
H	-12.250829	0.265494	-0.526166	H	-12.258622	0.249987	-0.527062	H	-12.344361	0.039062	-0.140269
H	-11.577103	-0.839525	0.670744	H	-11.583971	-0.868147	0.657080	H	-11.458605	-1.129123	0.837848
H	-13.042026	0.059768	1.032421	H	-13.050040	0.025174	1.028775	H	-12.904305	-0.363570	1.479610
C	8.778416	2.725943	-0.666852	C	8.767802	2.736167	-0.632552	C	9.070340	2.246156	-0.676884
H	9.660463	2.283942	-1.130080	H	9.650496	2.300620	-1.100635	H	9.843288	1.691926	-1.211386
H	8.325357	3.376656	-1.401337	H	8.314207	3.394884	-1.359534	H	8.665770	2.977238	-1.363807
H	9.100146	3.349747	0.167175	H	9.088565	3.350567	0.208796	H	9.549496	2.796192	0.133734
C	5.950246	2.542725	-1.631343	C	5.940130	2.560954	-1.599987	C	6.253217	2.422886	-1.608961
H	4.898900	2.356958	-1.794666	H	4.889047	2.375893	-1.765790	H	5.207419	2.296086	-1.851989
H	6.070051	3.560329	-1.270383	H	6.058652	3.574414	-1.227140	H	6.396033	3.437649	-1.243006
H	6.465032	2.464873	-2.586347	H	6.455287	2.494846	-2.555676	H	6.833061	2.315445	-2.522757
C	8.108332	-1.299135	2.218399	C	8.101526	-1.323077	2.205326	C	7.843042	-1.528613	2.359888
H	8.249141	-0.805743	3.178458	H	8.241482	-0.840759	3.171121	H	7.486451	-1.258312	3.352885
H	7.500420	-2.181253	2.364842	H	7.494593	-2.207545	2.341279	H	7.430701	-2.505192	2.119549
H	9.090215	-1.604912	1.871781	H	9.083864	-1.623655	1.855466	H	8.921605	-1.608064	2.401082
O	9.528086	0.743815	1.179283	O	9.519222	0.733497	1.190558	O	9.657678	0.257912	1.231092
H	10.021266	1.484347	0.809284	H	10.011653	1.478864	0.829377	H	10.205882	0.878169	0.750890
H	5.380349	-1.557466	2.441910	H	5.373779	-1.587145	2.424968	H	5.191060	-1.379719	2.601203
H	3.127727	-1.916227	1.317646	H	3.121904	-1.935361	1.295900	H	2.927334	-1.635997	1.416072
C	3.961197	-1.806547	-1.344133	C	3.956030	-1.793665	-1.364161	C	3.907936	-1.549531	-1.215719
H	3.389659	-2.701013	-1.116089	H	3.385460	-2.691390	-1.146746	H	3.350382	-2.453416	-0.983802
H	3.734634	-1.496447	-2.359554	H	3.729407	-1.471999	-2.375963	H	3.683756	-1.248809	-2.236026
H	5.017601	-2.052262	-1.285953	H	5.012701	-2.038824	-1.308527	H	4.968511	-1.773491	-1.151714
H	-1.501412	-1.394018	0.614385	H	-1.507628	-1.410321	0.597356	H	-1.599329	-1.364564	0.272564
C	-1.501484	-2.590475	-1.161347	C	-1.505794	-2.585974	-1.192216	C	-1.494822	-2.198690	-1.696484
H	-2.391326	-3.160442	-0.915496	H	-2.395048	-3.159797	-0.953307	H	-2.384005	-2.815784	-1.612072
H	-1.474906	-2.457021	-2.242865	H	-1.479052	-2.439878	-2.272095	H	-1.415083	-1.857960	-2.728883
H	-0.643620	-3.191041	-0.870931	H	-0.647321	-3.188898	-0.908564	H	-0.638220	-2.830759	-1.482303
H	-12.991601	2.481638	1.576093	H	-13.002577	2.440591	1.600687	H	-12.895511	1.986774	2.276937
C	-0.263446	-0.414000	-0.824758	C	-0.270374	-0.412148	-0.829871	C	-0.306537	-0.121572	-0.889970
H	-0.271828	-0.210670	-1.897505	H	-0.278675	-0.196323	-1.900175	H	-0.256444	0.263960	-1.910909
H	-0.326915	0.552593	-0.323607	H	-0.335109	0.548457	-0.317495	H	-0.418434	0.745411	-0.237710
**α-Tocopherol (C17$^{\bullet}$)**				**α-Tocopherol (C18$^{\bullet}$)**				**α-Tocopherol (C19$^{\bullet}$)**			
C	3.536776	-0.435726	-0.239580	C	-3.557248	-0.288791	0.387747	C	3.542687	-0.406040	-0.230756
C	5.716368	0.578541	-0.080563	C	-5.784187	0.489379	-0.104469	C	5.751710	0.534323	-0.033319
C	6.118145	-0.339418	0.884252	C	-6.148560	-0.751007	-0.617657	C	6.112705	-0.409951	0.922010
C	5.115214	-1.270257	1.514934	C	-5.107449	-1.813588	-0.855134	C	5.072874	-1.314437	1.530954
C	3.687235	-0.833533	1.221222	C	-3.699582	-1.238496	-0.792365	C	3.662836	-0.829396	1.225715
C	6.624508	1.463714	-0.666189	C	-6.728690	1.497298	0.105526	C	6.694929	1.395436	-0.599063
C	7.460255	-0.401879	1.263854	C	-7.489027	-1.011852	-0.908866	C	7.448414	-0.524157	1.311801
H	5.276980	-2.284059	1.145848	H	-5.220414	-2.607171	-0.114988	H	5.207293	-2.329161	1.153531
H	3.430607	0.034966	1.826169	H	-3.489062	-0.670870	-1.697564	H	3.426126	0.038445	1.839555
C	8.356790	0.481154	0.685173	C	-8.422018	-0.008815	-0.703840	C	8.380320	0.334266	0.752034
C	7.957762	1.416421	-0.267775	C	-8.061036	1.242810	-0.208518	C	8.022187	1.296467	-0.190263
O	4.408108	0.683633	-0.491508	O	-4.479875	0.801860	0.198832	O	4.451552	0.689360	-0.454400
C	2.148328	0.117951	-0.536765	C	-2.196181	0.396057	0.402957	C	2.176474	0.196398	-0.535500
H	2.174934	0.534431	-1.543992	H	-2.242691	1.191946	1.146556	H	2.237535	0.651324	-1.524304
H	1.968053	0.948833	0.144711	H	-2.056092	0.876174	-0.565206	H	2.003260	1.004798	0.174437
C	1.008480	-0.889872	-0.436613	C	-1.007390	-0.509527	0.706334	C	1.006837	-0.781529	-0.502720
H	1.207435	-1.730471	-1.096759	H	-1.142499	-0.974282	1.680019	H	1.178107	-1.574978	-1.225628
H	0.942266	-1.287048	0.576515	H	-0.946100	-1.314084	-0.026851	H	0.931777	-1.252095	0.477793
C	-1.549783	-1.136286	-0.532873	C	1.565767	-0.575061	0.807393	C	-1.555552	-0.956801	-0.684717

C	-2.827160	-0.365085	-0.871253	C	2.797831	0.310766	0.612323	C	-2.810006	-0.093730	-0.837664
H	-2.865659	-0.205319	-1.951370	H	2.833447	1.046441	1.419413	H	-2.810676	0.350578	-1.835872
H	-2.767864	0.624235	-0.415356	H	2.675644	0.874116	-0.313863	H	-2.749064	0.735051	-0.131008
C	-4.118873	-1.033001	-0.410731	C	4.125734	-0.437960	0.553755	C	-4.126212	-0.831950	-0.614699
H	-4.057357	-1.230933	0.661404	H	4.056912	-1.236097	-0.188688	H	-4.090144	-1.352237	0.344814
H	-4.233225	-1.995487	-0.902700	H	4.323176	-0.913669	1.510711	H	-4.251502	-1.591701	-1.381113
C	-5.341065	-0.166075	-0.692583	C	5.284845	0.485644	0.193328	C	-5.322927	0.114012	-0.630110
H	-5.168679	0.831800	-0.288214	H	5.029833	1.028216	-0.718070	H	-5.138943	0.920925	0.080526
H	-5.461833	-0.048777	-1.771688	H	5.404826	1.236878	0.977811	H	-5.401734	0.580789	-1.614857
C	-6.650237	-0.700813	-0.110282	C	6.622833	-0.221750	-0.017029	C	-6.663534	-0.539908	-0.287786
H	-6.513966	-0.832285	0.969425	H	6.497216	-0.952226	-0.819863	H	-6.561807	-1.022222	0.687724
C	-7.759733	0.270814	-0.322426	C	7.685778	0.785582	-0.464506	C	-7.740798	0.539330	-0.169206
H	-7.631629	1.062152	-1.047073	H	7.271409	1.390467	-1.282206	H	-7.397781	1.302395	0.529182
C	-9.105716	0.065700	0.274773	H	7.875200	1.488968	0.351701	H	-7.856150	1.031661	-1.137255
H	-9.006103	-0.455950	1.230524	C	8.964856	0.163686	-0.894663	C	-9.104875	0.033363	0.298849
H	-9.702952	-0.594698	-0.362954	H	8.928286	-0.810297	-1.363312	H	-8.984409	-0.555544	1.212982
C	-9.863137	1.376779	0.483687	C	10.215602	0.956367	-1.014312	H	-9.508295	-0.659935	-0.446584
H	-9.234243	2.051776	1.064020	H	10.170694	1.594727	-1.907097	C	-10.074880	1.133403	0.542516
H	-10.023404	1.855496	-0.484953	H	10.298248	1.644690	-0.167860	H	-9.991582	2.038861	-0.041309
C	-11.210942	1.227306	1.188915	C	11.485949	0.106881	-1.088860	C	-11.277630	0.933796	1.398372
H	-11.035538	0.738737	2.149077	H	11.365726	-0.600003	-1.911504	H	-10.944410	0.554329	2.369448
C	-11.813961	2.604023	1.452201	C	12.699342	0.984113	-1.375418	C	-12.028815	2.243119	1.618019
H	-11.139871	3.223218	2.039457	H	12.582169	1.526870	-2.310521	H	-11.382079	2.994592	2.063280
H	-12.004242	3.114578	0.508787	H	12.830697	1.713131	-0.576505	H	-12.390325	2.627531	0.665533
C	-7.012627	-2.079960	-0.687983	C	7.084693	-0.954398	1.239813	C	-7.045769	-1.603168	-1.315013
H	-7.076556	-2.022989	-1.773544	H	7.128863	-0.259260	2.078424	H	-7.166116	-1.144909	-2.296907
H	-6.269934	-2.827366	-0.423123	H	6.413805	-1.765573	1.506277	H	-6.285382	-2.374303	-1.393180
H	-7.974085	-2.415082	-0.308564	H	8.079034	-1.370344	1.097439	H	-7.979044	-2.093112	-1.054915
C	-12.188683	0.374728	0.384523	C	11.689009	-0.679335	0.202273	C	-12.218762	-0.125648	0.800966
H	-12.331751	0.806877	-0.605766	H	11.811733	0.008331	1.038691	H	-12.586664	0.207778	-0.167635
H	-11.836716	-0.645202	0.261409	H	10.834798	-1.316874	0.415479	H	-11.707448	-1.074486	0.663712
H	-13.157966	0.336068	0.876220	H	12.579082	-1.301605	0.144809	H	-13.072557	-0.288258	1.455394
C	8.994637	2.348909	-0.837901	C	-9.136958	2.281528	-0.027071	C	9.095271	2.201704	-0.736743
H	9.773665	1.800336	-1.369433	H	-9.883294	1.957662	0.699922	H	9.853182	1.637067	-1.281668
H	8.560283	3.049224	-1.538527	H	-8.731338	3.219966	0.325921	H	8.689284	2.936549	-1.418770
H	9.471575	2.933528	-0.050464	H	-9.648996	2.484355	-0.968107	H	9.593313	2.747446	0.065413
C	6.153266	2.448713	-1.704560	C	-6.296434	2.830919	0.657720	C	6.266965	2.409862	-1.627759
H	5.103888	2.298784	-1.917231	H	-5.238537	2.821196	0.880561	H	5.216898	2.293175	-1.857106
H	6.287285	3.472353	-1.360850	H	-6.485542	3.629635	-0.056716	H	6.424646	3.423835	-1.265687
H	6.710705	2.334590	-2.631360	H	-6.837304	3.068740	1.570896	H	6.833947	2.294784	-2.548609
C	7.923571	-1.400944	2.287987	C	-7.912077	-2.351471	-1.445491	C	7.868022	-1.551179	2.327059
H	7.587161	-1.121583	3.285521	H	-7.587873	-2.477958	-2.477585	H	7.529462	-1.274239	3.324535
H	7.524599	-2.389043	2.073673	H	-7.472586	-3.158349	-0.864630	H	7.440274	-2.523233	2.095415
H	9.004119	-1.459226	2.302717	H	-8.989374	-2.450800	-1.417746	H	8.946014	-1.643760	2.352394
O	9.670176	0.409857	1.090910	O	-9.733173	-0.288475	-1.015438	O	9.687050	0.210413	1.166733
H	10.191815	1.036906	0.590251	H	-10.281949	0.460176	-0.781885	H	10.234507	0.827273	0.681308
H	5.276562	-1.308642	2.589466	H	-5.273052	-2.277508	-1.824477	H	5.220223	-1.368766	2.606881
H	2.991587	-1.631534	1.464538	H	-2.967978	-2.038082	-0.718512	H	2.939460	-1.608187	1.449946
C	3.907904	-1.568572	-1.189423	C	-3.865094	-0.966669	1.717212	C	3.889661	-1.535270	-1.193553
H	3.382735	-2.482518	-0.924281	H	-3.290375	-1.883028	1.823939	H	3.328251	-2.434265	-0.952067
H	3.646612	-1.294378	-2.208352	H	-3.617578	-0.297174	2.537009	H	3.654573	-1.235352	-2.211656
H	4.975634	-1.762329	-1.151618	H	-4.920837	-1.212081	1.783348	H	4.949241	-1.767239	-1.142587
H	-1.567556	-1.363007	0.536253	H	1.552882	-1.313084	0.001160	H	-1.563827	-1.385044	0.320836
C	-1.469101	-2.451638	-1.304265	C	1.614121	-1.316829	2.140765	C	-1.536977	-2.100160	-1.696482
H	-2.347137	-3.067073	-1.133778	H	2.502153	-1.936771	2.218940	H	-2.397027	-2.751902	-1.577553
H	-1.398046	-2.251436	-2.373508	H	1.626414	-0.601917	2.963680	H	-1.552909	-1.699543	-2.710210
H	-0.600813	-3.034645	-1.011514	H	0.752115	-1.964749	2.268772	H	-0.647297	-2.713338	-1.589851
H	-12.757982	2.523612	1.985860	H	13.607367	0.388955	-1.436958	H	-12.886981	2.095710	2.269029
C	-0.329568	-0.254142	-0.803793	C	0.300997	0.276515	0.684377	C	-0.310967	-0.076120	-0.811992
H	-0.319993	0.019886	-1.861330	H	0.292329	1.013580	1.490568	H	-0.271581	0.333581	-1.823830
H	-0.441598	0.675469	-0.244534	H	0.351248	0.839587	-0.248423	H	-0.415587	0.774941	-0.137864
**α-Tocopherol (C20$^{\bullet}$)**				**α-Tocopherol (C21$^{\bullet}$)**				**α-Tocopherol (C22$^{\bullet}$)**			
C	3.539108	-0.405239	-0.281924	C	3.562064	-0.702804	-0.363608	C	3.546339	-0.399036	-0.296805
C	5.742258	0.533040	-0.012668	C	5.689425	0.484880	-0.148325	C	5.749187	0.538432	-0.019830
C	6.099798	-0.461289	0.891788	C	6.154690	-0.452121	0.823185	C	6.115839	-0.477183	0.856989
C	5.059705	-1.403214	1.440143	C	5.199714	-1.476613	1.372578	C	5.082358	-1.435966	1.388243
C	3.649704	-0.909259	1.149501	C	3.756034	-1.087941	1.087395	C	3.669038	-0.938597	1.120650
C	6.685447	1.430534	-0.519586	C	6.505967	1.562658	-0.644473	C	6.685931	1.452146	-0.509469
C	7.432426	-0.589472	1.287817	C	7.455452	-0.368858	1.237321	C	7.451398	-0.610509	1.241246
H	5.203645	-2.395324	1.009638	H	5.424309	-2.452497	0.944520	H	5.225967	-2.416431	0.931762
H	3.402672	-0.078709	1.809125	H	3.464559	-0.243388	1.709962	H	3.424842	-0.125708	1.802928
C	8.364108	0.304883	0.786983	C	8.261100	0.683717	0.720946	C	8.376477	0.300399	0.758424
C	8.009070	1.316880	-0.103110	C	7.798301	1.649877	-0.207385	C	8.012371	1.333280	-0.103615
O	4.445203	0.704246	-0.437163	O	4.489389	0.425301	-0.656658	O	4.448837	0.715972	-0.431519
C	2.172994	0.209158	-0.562077	C	2.193711	-0.100535	-0.635853	C	2.176643	0.218930	-0.550728

H	2.234090	0.707616	-1.529653	H	2.189970	0.270797	-1.660079	H	2.227243	0.738008	-1.507992
H	1.999773	0.985410	0.182920	H	2.064156	0.759647	0.019836	H	2.009794	0.978841	0.212352
C	1.002900	-0.768688	-0.571137	C	1.027742	-1.067774	-0.444132	C	1.008140	-0.760626	-0.568330
H	1.173801	-1.533266	-1.324817	H	1.177421	-1.953179	-1.056746	H	1.172877	-1.509674	-1.338825
H	0.926156	-1.277710	0.389824	H	0.975739	-1.396738	0.593621	H	0.941881	-1.289012	0.382872
C	-1.560957	-0.928793	-0.739195	C	-1.539085	-1.214801	-0.458446	C	-1.556571	-0.923105	-0.703845
C	-2.813514	-0.060347	-0.874925	C	-2.788713	-0.403196	-0.806463	C	-2.813665	-0.057732	-0.814820
H	-2.824184	0.388274	-1.871119	H	-2.832524	-0.273215	-1.890289	H	-2.840481	0.398022	-1.807445
H	-2.741257	0.765127	-0.165451	H	-2.684586	0.595635	-0.379923	H	-2.734023	0.762798	-0.100381
C	-4.129929	-0.794082	-0.638174	C	-4.102721	-1.003091	-0.316109	C	-4.123701	-0.798293	-0.564423
H	-4.084336	-1.315925	0.320159	H	-4.032228	-1.199698	0.755565	H	-4.064295	-1.322246	0.391975
H	-4.268191	-1.552264	-1.404055	H	-4.276938	-1.959052	-0.802234	H	-4.266886	-1.555322	-1.330451
C	-5.322842	0.156743	-0.637255	C	-5.281098	-0.070524	-0.580197	C	-5.321886	0.145724	-0.547570
H	-5.118372	0.972144	0.057936	H	-5.047266	0.911396	-0.166216	H	-5.116152	0.958909	0.149968
H	-5.423638	0.610951	-1.625935	H	-5.397352	0.068165	-1.657667	H	-5.434052	0.604295	-1.532996
C	-6.657353	-0.486094	-0.253013	C	-6.616656	-0.537938	0.001887	C	-6.648995	-0.507471	-0.155549
H	-6.541187	-0.932270	0.737922	H	-6.479530	-0.696792	1.074526	H	-6.520811	-0.962302	0.829993
C	-7.737449	0.593481	-0.159844	C	-7.663541	0.562897	-0.181360	C	-7.734162	0.565008	-0.041373
H	-7.363196	1.406308	0.464151	H	-7.264496	1.492624	0.227029	H	-7.366913	1.363389	0.605474
H	-7.898780	1.016810	-1.154200	H	-7.810073	0.733085	-1.250723	H	-7.890455	1.012510	-1.025793
C	-9.069974	0.120632	0.411071	C	-9.013439	0.284923	0.472551	C	-9.069006	0.071337	0.506028
H	-8.902618	-0.348040	1.381893	H	-8.860693	0.029187	1.523233	H	-8.901803	-0.450438	1.450273
H	-9.504850	-0.637071	-0.236348	H	-9.479401	-0.576732	0.002190	H	-9.515278	-0.651625	-0.174237
C	-10.056864	1.275537	0.570213	C	-9.948614	1.485801	0.376970	C	-10.057048	1.209370	0.732577
H	-9.577460	2.082001	1.128180	H	-9.436000	2.363274	0.774034	H	-9.628181	1.928215	1.431402
H	-10.257451	1.693629	-0.427943	H	-10.157557	1.698625	-0.674240	H	-10.219945	1.744404	-0.205806
C	-11.349868	0.930953	1.232780	C	-11.276992	1.320918	1.115491	C	-11.411182	0.739292	1.264850
C	-12.204942	2.060889	1.692063	H	-11.054666	1.109942	2.163185	H	-11.240637	0.167240	2.179426
H	-11.608818	2.854684	2.137366	C	-12.077626	2.617841	1.043550	C	-12.287252	1.950833	1.633303
H	-12.756468	2.508373	0.855389	H	-11.513747	3.452834	1.452914	H	-11.795190	2.558205	2.390448
C	-7.051780	-1.586028	-1.235782	C	-7.072376	-1.852235	-0.627279	H	-12.457298	2.567956	0.753717
H	-7.152051	-1.169273	-2.238165	H	-7.233381	-1.716406	-1.696827	C	-7.049126	-1.600441	-1.143770
H	-6.308053	-2.376312	-1.271345	H	-6.336089	-2.639236	-0.492611	H	-7.171092	-1.173549	-2.139543
H	-7.998606	-2.042402	-0.963945	H	-8.002205	-2.200761	-0.188822	H	-6.298203	-2.382601	-1.201175
C	-12.004052	-0.377334	0.948315	C	-12.102876	0.165929	0.555514	H	-7.986572	-2.068790	-0.860351
H	-12.361726	-0.428854	-0.088095	H	-12.279311	0.315514	-0.509546	C	-12.114199	-0.125619	0.280746
H	-11.323665	-1.214409	1.086001	H	-11.608103	-0.791904	0.686562	H	-11.985433	0.045940	-0.775914
H	-12.865635	-0.529417	1.593279	H	-13.069928	0.111867	1.049609	H	-12.880912	-0.813287	0.593731
C	9.081763	2.258184	-0.585356	C	8.757397	2.710333	-0.666079	C	9.078667	2.290820	-0.567621
H	9.846597	1.730744	-1.157441	H	9.637697	2.265697	-1.130107	H	9.841824	1.781382	-1.158033
H	8.677987	3.031089	-1.225146	H	8.305798	3.362642	-1.400048	H	8.668240	3.078535	-1.184673
H	9.570930	2.755897	0.252469	H	9.081725	3.332921	0.167851	H	9.571210	2.768142	0.279942
C	6.260925	2.498587	-1.493980	C	5.927915	2.536188	-1.628402	C	6.251522	2.542806	-1.454022
H	5.214909	2.386200	-1.742957	H	4.875867	2.353745	-1.790948	H	5.205412	2.430357	-1.702532
H	6.404822	3.491560	-1.072566	H	6.051179	3.553322	-1.267283	H	6.390579	3.525255	-1.007001
H	6.840461	2.441223	-2.412454	H	6.441709	2.456969	-2.583828	H	6.828537	2.513310	-2.375366
C	7.848581	-1.669295	2.248312	C	8.077005	-1.313357	2.218695	C	7.877688	-1.713418	2.170576
H	7.497094	-1.451248	3.255893	H	8.220104	-0.820648	3.178766	H	7.534162	-1.521920	3.186282
H	7.430574	-2.629634	1.957542	H	7.466457	-2.193610	2.365395	H	7.459130	-2.666925	1.858869
H	8.926897	-1.755764	2.280541	H	9.057657	-1.622111	1.871233	H	8.956401	-1.798329	2.191691
O	9.667415	0.166469	1.208035	O	9.502318	0.725412	1.178976	O	9.682723	0.156976	1.168654
H	10.219101	0.798270	0.747269	H	9.997518	1.464493	0.808775	H	10.230531	0.797120	0.714782
H	5.197829	-1.515912	2.512826	H	5.348405	-1.563226	2.444277	H	5.228439	-1.575661	2.456661
H	2.929276	-1.703448	1.323388	H	3.093795	-1.914671	1.321687	H	2.952051	-1.738972	1.279702
C	3.897028	-1.477443	-1.303794	C	3.925522	-1.806926	-1.340716	C	3.898599	-1.444813	-1.347586
H	3.333513	-2.388658	-1.120199	H	3.351372	-2.699660	-1.112447	H	3.337772	-2.361170	-1.182467
H	3.672344	-1.119277	-2.305196	H	3.699134	-1.495865	-2.355882	H	3.666016	-1.062229	-2.338074
H	4.956172	-1.712121	-1.255333	H	4.981199	-2.055952	-1.283424	H	4.958498	-1.678733	-1.312463
H	-1.568600	-1.373674	0.259125	H	-1.534239	-1.377836	0.622179	H	-1.550647	-1.379451	0.289306
C	-1.546691	-2.055447	-1.769591	C	-1.539436	-2.573841	-1.153849	C	-1.550628	-2.037992	-1.747169
H	-2.415621	-2.698564	-1.669417	H	-2.430860	-3.141089	-0.907443	H	-2.417247	-2.683975	-1.645729
H	-1.548359	-1.638014	-2.776650	H	-1.513287	-2.440200	-2.235354	H	-1.563275	-1.609024	-2.749311
H	-0.665292	-2.680958	-1.665076	H	-0.683224	-3.177155	-0.864254	H	-0.667045	-2.662961	-1.658855
H	-12.944748	1.732530	2.418856	H	-13.011516	2.533431	1.593843	H	-13.252724	1.633302	2.017050
C	-0.313923	-0.050015	-0.853272	C	-0.294346	-0.401326	-0.817688	C	-0.313209	-0.039763	-0.822471
H	-0.279879	0.385576	-1.854402	H	-0.302931	-0.197702	-1.890378	H	-0.293610	0.410266	-1.817560
H	-0.410608	0.783605	-0.156567	H	-0.354405	0.565334	-0.316248	H	-0.402120	0.783496	-0.112528

Structures of α-Tocopherol and their corresponding species in the SPLET (SP_step) mechanism at M05-2X/6-311++G(2d,2p) level of theory in gas phase.

**M05-2X/6-311++G(2d,2p)**

**α-Tocopherol**				**α-Tocopherol (O^-)**				**α-Tocopherol ($C1^-$)**			
C	3.59651900	0.43682900	0.33058300	C	3.634060	-0.291274	-0.285060	C	-3.632969	-0.299554	0.368986
C	5.77121200	-0.56232900	0.03334700	C	5.847440	0.572568	0.069177	C	-5.805087	0.616528	-0.124821
C	6.16792900	0.45700700	-0.82590000	C	6.198734	-0.508670	0.869184	C	-6.229812	-0.609232	-0.617857
C	5.16441600	1.45924100	-1.33378700	C	5.143396	-1.488417	1.322119	C	-5.270084	-1.743490	-0.853244
C	3.73717700	0.99743800	-1.07688200	C	3.733022	-0.966495	1.079150	C	-3.829376	-1.254493	-0.801743
C	6.67778500	-1.51871600	0.49745500	C	6.818068	1.468531	-0.383311	C	-6.657259	1.693336	0.077155
C	7.50515700	0.55122000	-1.21615000	C	7.530070	-0.719161	1.220050	C	-7.618479	-0.765056	-0.863855
H	5.33669100	2.42427900	-0.85506500	H	5.279310	-2.437213	0.797463	H	-5.403249	-2.539309	-0.111115
H	3.47117700	0.20436000	-1.77423900	H	3.482038	-0.213045	1.825813	H	-3.590376	-0.703527	-1.711264
C	8.40005700	-0.40192000	-0.75889800	C	8.562123	0.173522	0.796914	C	-8.474403	0.301214	-0.671987
C	8.00476400	-1.43910200	0.08402800	C	8.143401	1.280349	-0.013810	C	-8.047706	1.576196	-0.222896
O	4.46828900	-0.70399300	0.44996700	O	4.511743	0.828875	-0.293848	O	-4.458736	0.845070	0.170687
C	2.21067000	-0.14981400	0.57036800	C	2.256092	0.328210	-0.504910	C	-2.222710	0.283202	0.390809
H	2.24190400	-0.67725700	1.52392600	H	2.314676	0.929848	-1.412880	H	-2.204148	1.057992	1.158297
H	2.03262800	-0.90018500	-0.19957100	H	2.071878	1.017884	0.318949	H	-2.062315	0.782075	-0.564991
C	1.06331100	0.85431900	0.58573900	C	1.097397	-0.655842	-0.622520	C	-1.093918	-0.710315	0.642660
H	1.24033400	1.60137700	1.35577100	H	1.284458	-1.338019	-1.448499	H	-1.288326	-1.266642	1.557366
H	1.00861200	1.38258000	-0.36635400	H	1.025830	-1.261332	0.281400	H	-1.046471	-1.438503	-0.167812
C	-1.50126600	1.05480900	0.68351800	C	-1.466772	-0.831039	-0.783611	C	1.466856	-0.930648	0.711039
C	-2.77112400	0.22248500	0.87473500	C	-2.733394	0.024586	-0.852690	C	2.759303	-0.138412	0.931345
H	-2.84082400	-0.07719700	1.92322300	H	-2.766923	0.525330	-1.823295	H	2.923748	-0.011912	2.003931
H	-2.67683800	-0.69825400	0.29738300	H	-2.660573	0.812318	-0.101468	H	2.644699	0.864777	0.518748
C	-4.06245700	0.92067300	0.45959400	C	-4.034698	-0.741074	-0.633813	C	3.985758	-0.788220	0.298440
H	-3.98483200	1.22324600	-0.58672900	H	-3.971057	-1.295704	0.304863	H	3.881446	-0.752415	-0.787707
H	-4.19550200	1.82944200	1.04050400	H	-4.163204	-1.474584	-1.425061	H	4.023195	-1.841315	0.572281
C	-5.27901300	0.01647300	0.63358700	C	-5.247100	0.183834	-0.589676	C	5.283966	-0.094144	0.700267
H	-5.06562600	-0.94779600	0.16998500	H	-5.052596	0.979871	0.130504	H	5.134245	0.985170	0.642240
H	-5.43410200	-0.17997900	1.69700500	H	-5.367111	0.668980	-1.561457	H	5.506568	-0.319269	1.746043
C	-6.57944700	0.56121200	0.03970200	C	-6.564041	-0.499488	-0.215117	C	6.500397	-0.463186	-0.151310
H	-6.41363000	0.73696100	-1.02621200	H	-6.425736	-0.984473	0.754554	H	6.285756	-0.170715	-1.182345
C	-7.68249800	-0.48977300	0.17857000	C	-7.664213	0.552736	-0.061931	C	7.719383	0.330599	0.322190
H	-7.31249200	-1.43541800	-0.22047200	H	-7.309308	1.328682	0.617777	H	7.437435	1.380709	0.411642
H	-7.87446400	-0.65755300	1.24112500	H	-7.823302	1.037376	-1.028213	H	7.986841	-0.001892	1.328295
C	-8.99413000	-0.14802300	-0.52124400	C	-8.995355	0.019174	0.458946	C	8.942467	0.236735	-0.584434
H	-8.79374900	0.09236500	-1.56749500	H	-8.824089	-0.534545	1.384534	H	8.684807	0.624058	-1.572330
H	-9.43196000	0.73930100	-0.07153800	H	-9.412895	-0.684319	-0.256757	H	9.227277	-0.803598	-0.717187
C	-9.98987600	-1.30094200	-0.44998700	C	-9.999075	1.136995	0.720396	C	10.125878	1.024670	-0.031305
H	-9.50768100	-2.20497900	-0.82491200	H	-9.536737	1.879161	1.372836	H	9.782825	2.018939	0.259002
H	-10.24108200	-1.49593200	0.59553200	H	-10.224355	1.648331	-0.218729	H	10.485472	0.544336	0.882016
C	-11.28601700	-1.08055300	-1.23009700	C	-11.312323	0.679736	1.355448	C	11.298441	1.176921	-0.999752
H	-11.02318100	-0.88985800	-2.27222800	H	-11.073268	0.169877	2.290495	H	10.930455	1.689882	-1.890194
C	-12.14877000	-2.33765600	-1.17145500	C	-12.187545	1.888293	1.675099	C	12.392853	2.031470	-0.367755
H	-11.61424800	-3.20097200	-1.56083400	H	-11.668621	2.588543	2.325569	H	12.004694	2.997605	-0.053423
H	-12.42665700	-2.55124100	-0.13986300	H	-12.449033	2.412936	0.756824	H	12.797743	1.530024	0.510608
C	-6.98054900	1.88334000	0.68937900	C	-6.950085	-1.566371	-1.237192	C	6.758118	-1.967834	-0.118046
H	-7.14367800	1.73829300	1.75760400	H	-7.090606	-1.108078	-2.216566	H	6.928603	-2.291672	0.908989
H	-6.20912200	2.63743700	0.56459400	H	-6.180390	-2.326546	-1.328404	H	5.909419	-2.521160	-0.508574
H	-7.89567900	2.27858900	0.25882100	H	-7.873403	-2.067850	-0.962875	H	7.627793	-2.242100	-0.708008
C	-12.07257500	0.11709200	-0.70398700	C	-12.073255	-0.292046	0.457281	C	11.866157	-0.174955	-1.423480
H	-12.28757800	-0.01517600	0.35629800	H	-12.258703	0.167030	-0.513799	H	12.172310	-0.742468	-0.544641
H	-11.52753600	1.04844000	-0.82545700	H	-11.523281	-1.214292	0.295930	H	11.138380	-0.765969	-1.971608
H	-13.02065300	0.21379500	-1.22790100	H	-13.035110	-0.547945	0.896050	H	12.739231	-0.043436	-2.058901
C	9.03854600	-2.44411700	0.52002900	C	9.243192	2.212636	-0.434536	C	-8.990550	2.581847	-0.018417
H	9.82113300	-1.97637900	1.11954800	H	9.929853	1.711814	-1.118575	H	-8.676748	3.561685	0.290244
H	8.60250900	-3.23155600	1.11962300	H	8.870697	3.116681	-0.906882	H	-9.934422	2.552751	-0.546130
H	9.51116100	-2.91772600	-0.34074600	H	9.840986	2.475620	0.436251	C	-6.150203	3.002602	0.595875
C	6.21047200	-2.61286300	1.42214900	C	6.409748	2.627592	-1.262109	H	-5.073244	2.991075	0.720641
H	5.17189900	-2.46572200	1.68393100	H	5.369303	2.538785	-1.549877	H	-6.420937	3.810113	-0.087474
H	6.30674600	-3.58966300	0.95198500	H	6.539896	3.578931	-0.746499	H	-6.614145	3.242066	1.556333
H	6.79711100	-2.62675900	2.33775700	H	7.018640	2.662505	-2.163346	C	-8.150083	-2.099656	-1.321702
C	7.96525200	1.65727700	-2.12538000	C	7.932855	-1.901827	2.056297	H	-7.875352	-2.302960	-2.357991
H	7.61719700	1.49460300	-3.14459500	H	7.561522	-1.829142	3.081652	H	-7.746052	-2.911008	-0.717614
H	7.57566000	2.61725400	-1.79664200	H	7.561959	-2.843121	1.646748	H	-9.230286	-2.116481	-1.251483
H	9.04608900	1.70939700	-2.14478200	H	9.017282	-1.927405	2.088542	O	-9.846044	0.148648	-0.851956
O	9.70784700	-0.29661000	-1.17568900	O	9.789179	0.012045	1.107569	H	-10.237213	0.823696	-0.290406
H	10.23692600	-0.96076800	-0.73416100	H	5.275581	-1.711960	2.379082	H	-5.456231	-2.207426	-1.822307
H	5.31437700	1.61873200	-2.39882500	H	3.009522	-1.776301	1.156742	H	-3.141252	-2.094547	-0.720067
H	3.04324600	1.82049300	-1.22178100	C	4.003876	-1.244495	-1.419848	C	-3.992365	-0.950710	1.702541
C	3.97276200	1.45514300	1.39978200	H	3.459168	-2.182955	-1.337079	H	-3.504248	-1.917078	1.814007
H	3.43343800	2.38701800	1.25084900	H	3.772744	-0.781332	-2.376999	H	-3.690200	-0.301443	2.521414
H	3.73077000	1.06035600	2.38315000	H	5.068871	-1.453590	-1.387917	H	-5.067228	-1.095439	1.754755
H	5.03790700	1.66455600	1.36904700	H	-1.463450	-1.348005	0.179426	H	1.509973	-1.378342	-0.284858
H	-1.50515700	1.44477000	-0.33756200	C	-1.440002	-1.880186	-1.893090	C	1.341484	-2.052437	1.742109
C	-1.45549000	2.23389400	1.65275400	H	-2.301489	-2.540362	-1.844768	H	2.289707	-2.566711	1.882947

H	-2.34967200	2.84549700	1.58033500	H	-1.441758	-1.388985	-2.866570	H	1.034211	-1.638070	2.701886
H	-1.37746200	1.86855600	2.67677200	H	-0.549493	-2.497778	-1.830449	H	0.601163	-2.789567	1.445197
H	-0.60264800	2.87749000	1.45812400	H	-13.111365	1.586716	2.163542	H	13.211218	2.201112	-1.063828
H	-13.06376900	-2.21362900	-1.74581600	C	-0.229661	0.067423	-0.836347	C	0.253429	-0.001066	0.748333
C	-0.27150300	0.15856900	0.83907100	H	-0.209571	0.585116	-1.798322	H	0.286843	0.581904	1.672044
H	-0.27121500	-0.26754400	1.84495800	H	-0.329888	0.840372	-0.072950	H	0.337722	0.713769	-0.071387
H	-0.36344500	-0.68009900	0.14796800								
**α-Tocopherol (C2$^-$)**				**α-Tocopherol (C3$^-$)**				**α-Tocopherol (C4$^-$)**			
C	3.641799	-0.282076	-0.280935	C	3.628691	-0.329654	-0.238213	C	3.629126	-0.277321	-0.284875
C	5.853305	0.574385	0.066680	C	5.845355	0.555289	0.082488	C	5.814696	0.598502	0.147562
C	6.235478	-0.537389	0.774136	C	6.173553	-0.508382	0.909356	C	6.145446	-0.580316	0.892156
C	5.205976	-1.555717	1.204692	C	5.131186	-1.448860	1.422664	C	5.151849	-1.457537	1.291947
C	3.784845	-1.030076	1.045360	C	3.716578	-0.957150	1.146533	C	3.720200	-1.183332	0.943536
C	6.778258	1.582688	-0.384807	C	6.778984	1.456236	-0.420854	C	6.747593	1.495943	-0.311507
C	7.598980	-0.771664	1.085968	C	7.537905	-0.781881	1.259645	C	7.541761	-0.778935	1.142518
H	5.327013	-2.475071	0.619367	H	5.283092	-2.443062	0.984128	H	5.394161	-2.357163	1.830053
H	3.561594	-0.317296	1.843405	H	3.443421	-0.184203	1.865240	H	3.153401	-0.686132	1.745133
C	8.509570	0.197998	0.681467	C	8.473265	0.177705	0.756093	C	8.456985	0.145500	0.688675
C	8.147674	1.350409	-0.008154	C	8.124622	1.244509	-0.030807	C	8.117600	1.293691	-0.032145
O	4.524927	0.825208	-0.272941	O	4.515107	0.788178	-0.283317	O	4.463172	0.873437	-0.073135
C	2.257301	0.352460	-0.425108	C	2.258904	0.295130	-0.492192	C	2.238676	0.329671	-0.446097
H	2.294719	1.010240	-1.298032	H	2.326239	0.857428	-1.424598	H	2.247283	0.966247	-1.333005
H	2.094237	0.996957	0.442876	H	2.078266	1.020096	0.301767	H	2.073485	0.981815	0.411663
C	1.093899	-0.624095	-0.571461	C	1.091623	-0.682181	-0.572646	C	1.095196	-0.673452	-0.545006
H	1.267228	-1.265709	-1.437245	H	1.275077	-1.399029	-1.369478	H	1.289159	-1.370344	-1.357493
H	1.041754	-1.281819	0.301698	H	1.013841	-1.249318	0.355046	H	1.043263	-1.259089	0.372780
C	-1.477805	-0.804134	-0.695416	C	-1.471901	-0.846381	-0.757662	C	-1.472978	-0.874125	-0.667980
C	-2.750156	0.047140	-0.754164	C	-2.733085	0.016543	-0.835318	C	-2.742851	-0.028090	-0.781216
H	-2.783597	0.565257	-1.719472	H	-2.754685	0.521237	-1.804263	H	-2.770421	0.431249	-1.772476
H	-2.680677	0.828286	0.008865	H	-2.662693	0.800848	-0.080293	H	-2.677727	0.791094	-0.063635
C	-4.052848	-0.722598	-0.550802	C	-4.040328	-0.743282	-0.632254	C	-4.045308	-0.785818	-0.542132
H	-3.997042	-1.284200	0.388291	H	-3.985639	-1.308270	0.300844	H	-3.989515	-1.303494	0.417850
H	-4.170294	-1.458542	-1.346640	H	-4.167629	-1.467654	-1.432059	H	-4.166967	-1.549489	-1.305524
C	-5.273043	0.194368	-0.517768	C	-5.248163	0.187442	-0.585931	C	-5.256549	0.141749	-0.545006
H	-5.091473	0.994335	0.206246	H	-5.054246	0.974915	0.143784	H	-5.071954	0.956601	0.156504
H	-5.383530	0.683500	-1.492424	H	-5.359863	0.683368	-1.553274	H	-5.357019	0.600811	-1.531687
C	-6.594251	-0.493750	-0.159243	C	-6.570484	-0.493520	-0.226309	C	-6.583535	-0.525706	-0.177143
H	-6.467726	-0.968816	0.820948	H	-6.439912	-0.989015	0.739100	H	-6.463171	-0.994159	0.802975
C	-7.705247	0.553385	-0.031546	C	-7.667421	0.561372	-0.068739	C	-7.677295	0.537821	-0.059858
H	-7.370451	1.332132	0.660247	H	-7.312707	1.330108	0.619231	H	-7.330609	1.317674	0.619576
H	-7.839554	1.044100	-1.002318	H	-7.820714	1.054893	-1.031457	H	-7.809324	1.013644	-1.034636
C	-9.051350	0.016023	0.448334	C	-9.002431	0.027547	0.441937	C	-9.025549	0.022847	0.434819
H	-8.907282	-0.543679	1.379121	H	-8.836537	-0.534528	1.363473	H	-8.881517	-0.524545	1.368809
H	-9.445283	-0.691080	-0.282715	H	-9.418918	-0.668692	-0.281425	H	-9.433955	-0.682495	-0.284354
C	-10.071250	1.127213	0.680979	C	-10.004186	1.145796	0.708959	C	-10.023220	1.153478	0.662847
H	-9.636084	1.872045	1.354076	H	-9.541962	1.881843	1.368396	H	-9.568596	1.896389	1.319868
H	-10.263684	1.645242	-0.265725	H	-10.225581	1.664835	-0.226863	H	-10.220575	1.658973	-0.285692
C	-11.407575	0.662776	1.265599	C	-11.320251	0.686806	1.336923	C	-11.356182	0.715887	1.269840
H	-11.200796	0.164642	2.218775	H	-11.084864	0.169343	2.268715	H	-11.145067	0.212372	2.214956
C	-12.310032	1.863794	1.540284	C	-12.193794	1.894761	1.663370	C	-12.226121	1.936418	1.556791
H	-11.827365	2.578528	2.207671	H	-11.675016	2.589141	2.320233	H	-11.715825	2.637409	2.213287
H	-12.543763	2.382268	0.607535	H	-12.452007	2.426645	0.748321	H	-12.459110	2.455133	0.627522
C	-6.957924	-1.578903	-1.172138	C	-6.954937	-1.548345	-1.261485	C	-6.963232	-1.607626	-1.185628
H	-7.098716	-1.136496	-2.162171	H	-7.086816	-1.079621	-2.237145	H	-7.098332	-1.163475	-2.172288
H	-6.175708	-2.332164	-1.250096	H	-6.188116	-2.311073	-1.355341	H	-6.192086	-2.367892	-1.261274
H	-7.878179	-2.093666	-0.899441	H	-7.882343	-2.048305	-0.998328	H	-7.887793	-2.105986	-0.909473
C	-12.119022	-0.333081	0.351038	C	-12.080847	-0.276470	0.429339	C	-12.105353	-0.256496	0.362449
H	-12.264109	0.103444	-0.640552	H	-12.262710	0.190482	-0.538656	H	-12.260521	0.194404	-0.617768
H	-11.553931	-1.256476	0.232586	H	-11.532229	-1.198508	0.262348	H	-11.561987	-1.186426	0.224643
H	-13.100946	-0.592212	0.748726	H	-13.044407	-0.533797	0.863544	H	-13.081057	-0.496869	0.778977
C	9.129926	2.411098	-0.405490	C	9.210972	2.191883	-0.484958	C	9.190164	2.254996	-0.471362
H	9.218604	2.491090	-1.496646	H	9.918409	1.708601	-1.164083	H	9.902551	1.812360	-1.177521
H	8.776247	3.390373	-0.061555	H	8.796494	3.044057	-1.010109	H	8.759029	3.115980	-0.969915
H	10.135004	2.281917	0.002697	H	9.778278	2.580391	0.362983	H	9.773599	2.649104	0.368759
C	6.358031	2.682004	-1.115467	C	6.371989	2.595701	-1.316024	C	6.296571	2.702138	-1.097214
H	5.315685	2.810899	-1.354434	H	5.319011	2.511031	-1.558114	H	5.229870	2.652442	-1.275619
H	7.054502	3.425726	-1.468071	H	6.522165	3.574286	-0.850629	H	6.509654	3.627846	-0.561846
C	8.030049	-2.008007	1.826201	H	6.927993	2.602507	-2.256092	H	6.807327	2.756617	-2.058134
H	7.718352	-2.006646	2.879207	C	7.937450	-1.868363	2.018562	C	7.956943	-1.996166	1.904935
H	7.616908	-2.920264	1.383047	H	7.228463	-2.591160	2.381308	H	7.472641	-2.018698	2.885083
H	9.114044	-2.096133	1.811696	H	8.976454	-2.008891	2.253525	H	7.633528	-2.902645	1.384812
O	9.842250	-0.039259	1.010529	O	9.803073	-0.040120	1.112960	H	9.031614	-2.032438	2.041855
H	10.370871	0.596948	0.529638	H	10.344656	0.577798	0.624984	O	9.802447	-0.093551	0.973429
H	5.380768	-1.842796	2.244137	H	5.272561	-1.587814	2.497197	H	10.305264	0.627053	0.596866
H	3.061701	-1.845837	1.109040	H	3.001936	-1.773600	1.238930	H	3.188693	-2.113323	0.726183

C	3.966215	-1.183498	-1.476307	C	4.002834	-1.320661	-1.337316	C	4.077827	-0.994577	-1.551878
H	3.419351	-2.126911	-1.424708	H	3.432799	-2.242433	-1.241563	H	3.450074	-1.861360	-1.747789
H	3.708529	-0.671294	-2.403939	H	3.807202	-0.878648	-2.312571	H	4.022249	-0.311843	-2.398440
H	5.033542	-1.398343	-1.493304	H	5.060156	-1.557262	-1.267998	H	5.101811	-1.333529	-1.432662
H	-1.476646	-1.338215	0.262508	H	-1.472240	-1.354177	0.210189	H	-1.465761	-1.340599	0.320391
C	-1.441979	-1.840569	-1.818365	C	-1.452276	-1.906264	-1.857134	C	-1.440178	-1.979454	-1.720859
H	-2.310140	-2.498454	-1.792956	H	-2.309713	-2.570422	-1.792953	H	-2.295647	-2.644119	-1.636219
H	-1.424928	-1.339226	-2.790067	H	-1.467348	-1.424526	-2.835303	H	-1.446898	-1.540632	-2.719200
H	-0.554691	-2.467848	-1.751831	H	-0.557997	-2.518559	-1.798288	H	-0.543299	-2.583278	-1.625748
H	-13.251924	1.555443	1.995512	H	-13.119400	1.591451	2.147378	H	-13.164950	1.649348	2.025006
C	-0.241658	0.100120	-0.725861	C	-0.228613	0.043140	-0.819159	C	-0.241731	0.028211	-0.768384
H	-0.239128	0.665491	-1.664693	H	-0.195264	0.536347	-1.793698	H	-0.238285	0.510441	-1.749256
H	-0.333419	0.840909	0.074170	H	-0.331551	0.835679	-0.076432	H	-0.338927	0.828520	-0.032956
**α-Tocopherol ($C5^-$)**				**α-Tocopherol ($C6^-$)**				**α-Tocopherol ($C7^-$)**			
C	3.609139	-0.450095	-0.318897	C	3.599837	-0.377559	-0.309718	C	-3.517573	-0.234274	0.205549
C	5.784225	0.551517	-0.033016	C	5.816025	0.529561	0.040324	C	-5.835711	0.546410	-0.079954
C	6.180164	-0.457574	0.838593	C	6.212261	-0.585321	0.783312	C	-6.209516	-0.610382	-0.772704
C	5.175995	-1.453121	1.358201	C	5.185657	-1.602345	1.203093	C	-5.152869	-1.588201	-1.215228
C	3.749066	-0.993756	1.095274	C	3.779394	-1.041566	1.072233	C	-3.766715	-0.960840	-1.119804
C	6.691418	1.501795	-0.508327	C	6.759305	1.478429	-0.377137	C	-6.810194	1.488230	0.295051
C	7.517219	-0.547724	1.230392	C	7.558403	-0.761697	1.106725	C	-7.556549	-0.848611	-1.061350
H	5.347952	-2.423946	0.891210	H	5.271054	-2.476738	0.556546	H	-5.197808	-2.485799	-0.594346
H	3.483229	-0.192195	1.782927	H	3.603228	-0.304090	1.861730	H	-3.637254	-0.221592	-1.908824
C	8.412735	0.399396	0.761972	C	8.476252	0.198916	0.715783	C	-8.502154	0.088806	-0.686860
C	8.018223	1.426528	-0.093532	C	8.094278	1.322160	-0.011087	C	-8.145307	1.259946	-0.023027
O	4.481507	0.688779	-0.451744	O	4.527236	0.774317	-0.278076	O	-4.562405	0.842714	0.251205
C	2.223656	0.134287	-0.566197	C	2.236842	0.311960	-0.367435	C	-2.184534	0.417559	0.298179
H	2.255457	0.650175	-1.526037	H	2.283456	0.998654	-1.210852	H	-1.931307	0.846366	-0.677846
H	2.045732	0.893977	0.194577	H	2.080666	0.895635	0.544896	C	-1.077457	-0.512547	0.735660
C	1.075809	-0.869400	-0.569834	C	1.075038	-0.646791	-0.580939	H	-1.250339	-0.871082	1.751381
H	1.252715	-1.625778	-1.330741	H	1.293715	-1.215093	-1.480734	H	-0.964208	-1.445623	0.123651
H	1.020543	-1.386112	0.388544	H	1.002408	-1.350628	0.251248	C	1.500353	-0.720742	0.855012
C	-1.488834	-1.069807	-0.666033	C	-1.488727	-0.807633	-0.747076	C	2.782896	0.112176	0.799623
C	-2.758222	-0.239232	-0.867694	C	-2.757118	0.047413	-0.778758	H	2.845833	0.717927	1.707350
H	-2.827435	0.047815	-1.919743	H	-2.782251	0.600220	-1.721078	H	2.704392	0.813935	-0.032309
H	-2.663671	0.688357	-0.301458	H	-2.692336	0.793483	0.015076	C	4.070098	-0.691334	0.637463
C	-4.050031	-0.931734	-0.444588	C	-4.060393	-0.728507	-0.613755	H	4.007797	-1.284223	-0.277792
H	-3.972893	-1.221702	0.605334	H	-4.012653	-1.318332	0.304448	H	4.169103	-1.394329	1.460958
H	-4.183334	-1.847379	-1.014538	H	-4.171023	-1.432264	-1.434405	C	5.302716	0.205731	0.572923
C	-5.266087	-0.029107	-0.629875	C	-5.275599	0.192278	-0.557883	H	5.119208	0.995572	-0.157217
H	-5.052377	0.940580	-0.177867	H	-5.087856	0.972899	0.180978	H	5.436998	0.703294	1.536708
H	-5.420735	0.154580	-1.695636	H	-5.388554	0.699021	-1.519533	C	6.605971	-0.504432	0.201418
C	-6.566980	-0.566010	-0.029892	C	-6.595265	-0.498834	-0.208169	H	6.460526	-0.984906	-0.769568
H	-6.401595	-0.728970	1.038119	H	-6.468465	-0.992132	0.758987	C	7.728801	0.524518	0.054667
C	-7.669471	0.483760	-0.181790	C	-7.701438	0.547937	-0.061404	H	7.389837	1.312254	-0.619634
H	-7.299130	1.433969	0.205939	H	-7.354850	1.324602	0.621901	H	7.896922	0.999683	1.024253
H	-7.861010	0.638805	-1.246354	H	-7.854929	1.033331	-1.028262	C	9.050043	-0.030986	-0.468541
C	-8.981497	0.151114	0.521663	C	-9.034619	0.009092	0.448630	H	8.870570	-0.568718	-1.401974
H	-8.781573	-0.076735	1.570803	H	-8.869293	-0.538132	1.379223	H	9.449616	-0.752631	0.239418
H	-9.419616	-0.741357	0.082548	H	-9.440492	-0.701264	-0.267020	C	10.077542	1.069474	-0.711213
C	-9.976654	1.303574	0.436177	C	-10.047503	1.122615	0.692966	H	9.631431	1.830410	-1.353262
H	-9.494137	2.211833	0.800330	H	-9.593726	1.874491	1.340349	H	10.309988	1.562374	0.236123
H	-10.227426	1.486061	-0.611701	H	-10.270019	1.622988	-0.252799	C	11.383851	0.597573	-1.349820
C	-11.273156	1.093241	1.218461	C	-11.361961	0.664506	1.324825	H	11.138150	0.106960	-2.293437
H	-11.010751	0.915002	2.262903	H	-11.125807	0.169275	2.268491	C	12.282864	1.794136	-1.648024
C	-12.135273	2.349965	1.144376	C	-12.248413	1.870706	1.621580	H	11.779655	2.513344	-2.290137
H	-11.600454	3.217654	1.523490	H	-11.739013	2.583926	2.265542	H	12.549306	2.300494	-0.720894
H	-12.412721	2.551227	0.110192	H	-12.507077	2.380474	0.694074	C	6.964114	-1.581724	1.222545
C	-6.968520	-1.895683	-0.663707	C	-6.962141	-1.558371	-1.244647	H	7.106460	-1.128558	2.204165
H	-7.131232	-1.763451	-1.733657	H	-7.085223	-1.092865	-2.222969	H	6.174731	-2.322182	1.305959
H	-6.197504	-2.648595	-0.529581	H	-6.187878	-2.314712	-1.327521	H	7.878307	-2.103760	0.954043
H	-7.883984	-2.285265	-0.228715	H	-7.889246	-2.064360	-0.991004	C	12.123628	-0.402411	-0.465117
C	-12.060130	-0.110280	0.706575	C	-12.109189	-0.325399	0.435045	H	12.314299	0.037758	0.513721
H	-12.274725	0.009294	-0.355299	H	-12.289924	0.118026	-0.544152	H	11.554868	-1.315813	-0.319716
H	-11.515588	-1.040360	0.839450	H	-11.550854	-1.245538	0.291478	H	13.081969	-0.670012	-0.905007
H	-13.008425	-0.200195	1.231305	H	-13.072740	-0.581975	0.869959	C	-9.228287	2.245747	0.336879
C	9.052639	2.425708	-0.541283	C	9.146080	2.336122	-0.385361	H	-9.931900	1.827101	1.060443
H	9.835191	1.950392	-1.134859	H	9.850955	1.940849	-1.120805	H	-8.812855	3.142184	0.777976
H	8.617183	3.206070	-1.150475	H	8.700588	3.224859	-0.813332	H	-9.795257	2.548012	-0.544877
H	9.525208	2.909435	0.313873	H	9.716025	2.650032	0.490201	C	-6.375020	2.727109	1.035504
C	6.224941	2.584937	-1.446305	C	6.299913	2.633702	-1.227694	H	-5.311905	2.674227	1.232971
H	5.186381	2.435154	-1.706636	H	5.302329	2.429139	-1.594674	H	-6.571405	3.626136	0.451886
H	6.321542	3.567290	-0.987925	H	6.273678	3.565971	-0.662290	H	-6.902267	2.825326	1.983173
H	6.811883	2.587503	-2.361820	H	6.958646	2.777451	-2.080848	C	-7.974870	-2.104803	-1.777335
C	7.976477	-1.642957	2.153048	C	8.007411	-1.981047	1.866323	H	-7.706323	-2.062277	-2.832757

H	7.628172	-1.467832	3.170112	H	7.693955	-1.932135	2.908964	H	-7.476652	-2.972660	-1.352906
H	7.586521	-2.606640	1.835785	H	7.567514	-2.878346	1.438432	H	-9.046731	-2.245490	-1.707427
H	9.057282	-1.695365	2.173434	H	9.086608	-2.071134	1.841181	O	-9.832308	-0.161907	-1.004562
O	9.720338	0.298485	1.180434	O	9.806999	0.015052	1.077095	H	-10.374376	0.488610	-0.559977
H	10.249886	0.957012	0.731102	H	10.339025	0.639187	0.585184	H	-5.358989	-1.912609	-2.235875
H	5.325533	-1.599827	2.425135	H	5.378373	-1.925486	2.225505	H	-2.992833	-1.718336	-1.240174
C	3.985229	-1.481414	-1.375611	H	3.050084	-1.841192	1.177853	C	-3.803196	-1.152002	1.391061
H	3.445400	-2.411163	-1.215627	C	3.865942	-1.213412	-1.526486	H	-3.242461	-2.081227	1.290373
H	3.743749	-1.098399	-2.363749	H	3.249231	-2.116083	-1.507779	H	-3.493832	-0.643258	2.299697
H	5.050261	-1.690960	-1.342002	H	4.914422	-1.514765	-1.567923	H	-4.862216	-1.392154	1.462724
H	-1.493247	-1.447422	0.359675	H	-1.497439	-1.387645	0.179963	H	1.511298	-1.414285	0.009607
C	-1.443322	-2.260519	-1.620962	C	-1.440089	-1.781087	-1.922639	C	1.421633	-1.537383	2.142806
H	-2.337828	-2.870769	-1.541466	H	-2.294774	-2.453256	-1.928251	H	2.294875	-2.171439	2.283658
H	-1.364784	-1.907597	-2.649286	H	-1.433959	-1.226043	-2.861051	H	1.343233	-0.865373	2.998192
H	-0.590859	-2.902136	-1.418302	H	-0.539333	-2.385202	-1.890101	H	0.541449	-2.173071	2.137416
H	-13.050519	2.233320	1.719890	H	-13.173591	1.569348	2.107817	H	13.203537	1.482977	-2.136801
C	-0.258581	-0.176107	-0.831979	C	-0.251761	0.092891	-0.722309	C	0.278782	0.190361	0.705244
H	-0.257758	0.237842	-1.842932	H	-0.230903	0.688486	-1.638635	H	0.292046	0.948694	1.490856
H	-0.350336	0.670880	-0.151072	H	-0.356161	0.799788	0.103794	H	0.362170	0.728686	-0.240728
**α-Tocopherol (C8$^-$)**				**α-Tocopherol (C9$^-$)**				**α-Tocopherol (C10$^-$)**			
C	3.560920	0.624531	-0.128761	C	-3.541340	-0.353176	-0.308657	C	3.583040	-0.302994	-0.314222
C	5.776206	-0.121363	0.489074	C	-5.805982	-0.008764	0.476766	C	5.820077	0.542671	0.040698
C	5.944218	-0.773365	-0.734677	C	-6.041731	0.970237	-0.487686	C	6.223816	-0.648315	0.639932
C	4.775852	-0.919203	-1.676358	C	-4.900857	1.504379	-1.314210	C	5.205556	-1.702841	0.987184
C	3.461883	-0.633668	-0.967623	C	-3.558325	1.112924	-0.713386	C	3.788533	-1.149742	0.931231
C	6.844400	0.005436	1.385841	C	-6.844550	-0.489707	1.283451	C	6.744915	1.547032	-0.265721
C	7.197286	-1.289539	-1.077213	C	-7.337074	1.456853	-0.678914	C	7.576518	-0.860954	0.915814
H	4.901751	-0.234552	-2.518515	H	-4.978719	1.127493	-2.335353	H	5.303212	-2.544118	0.299213
H	3.216537	-1.455636	-0.295215	H	-3.372865	1.696122	0.187018	H	3.599698	-0.514750	1.795907
C	8.243896	-1.165786	-0.183517	C	-8.357977	0.976839	0.123619	C	8.485664	0.138128	0.612771
C	8.086771	-0.531965	1.045131	C	-8.128458	0.018557	1.108317	C	8.088766	1.341487	0.034492
O	4.587517	0.397151	0.890300	O	-4.568940	-0.529459	0.710858	O	4.514510	0.809921	-0.254876
C	2.264892	0.895409	0.624981	C	-2.236293	-0.740297	0.374599	C	2.211134	0.354836	-0.330685
H	2.529553	1.649458	1.398443	H	-2.401190	-1.668513	0.918011	H	2.187076	1.040007	-1.178860
H	2.023851	-0.018551	1.174812	H	-1.997178	0.020799	1.117856	H	2.123241	0.962195	0.571079
C	1.109103	1.277645	-0.276139	C	-1.037736	-0.932883	-0.554746	C	1.028900	-0.604643	-0.430682
H	1.263720	2.309412	-0.602078	H	-1.214382	-1.837265	-1.138956	H	1.125701	-1.237784	-1.308569
C	-1.451036	1.312671	-0.418270	H	-1.024573	-0.106936	-1.309177	H	0.989596	-1.256174	0.444102
C	-2.731907	1.088774	0.388125	C	1.461832	-1.144914	-0.673522	C	-1.512016	-0.791703	-0.505546
H	-2.842705	1.890614	1.123970	C	2.740602	-0.958280	0.145824	C	-2.762197	0.088323	-0.587704
H	-2.624531	0.163567	0.958740	H	2.834869	-1.812116	0.819403	H	-2.736457	0.609510	-1.547772
C	-4.005404	0.999234	-0.450516	H	2.612137	-0.079531	0.780535	H	-2.731694	0.838951	0.211485
H	-3.875828	0.227991	-1.213655	C	4.027584	-0.795825	-0.662392	C	-4.066229	-0.690183	-0.484375
H	-4.157516	1.937227	-0.979511	H	3.938853	0.071429	-1.320399	H	-4.048572	-1.301740	0.422327
C	-5.233487	0.668534	0.390980	H	4.162790	-1.663240	-1.303600	H	-4.117104	-1.367685	-1.331382
H	-5.008470	-0.214989	0.991054	C	5.248808	-0.619514	0.236435	C	-5.289197	0.221263	-0.458954
H	-5.422339	1.479434	1.098854	H	5.041150	0.187915	0.941135	H	-5.145791	0.981708	0.311982
C	-6.512968	0.401003	-0.404269	H	5.385863	-1.521906	0.837985	H	-5.357372	0.755851	-1.410377
H	-6.299519	-0.396091	-1.121135	C	6.562110	-0.308426	-0.486763	C	-6.620806	-0.486217	-0.196665
C	-7.613022	-0.096370	0.533983	H	6.402915	0.577806	-1.106824	H	-6.534560	-1.017190	0.755302
H	-7.219226	-0.934753	1.110343	C	7.646382	0.024666	0.540478	C	-7.741342	0.546528	-0.060188
H	-7.845328	0.691227	1.255736	H	7.263149	0.800101	1.205493	H	-7.431421	1.299267	0.666650
C	-8.898035	-0.545992	-0.156175	H	7.820406	-0.856006	1.163761	H	-7.854102	1.067088	-1.014516
H	-8.651307	-1.269011	-0.936348	C	8.974167	0.497354	-0.043968	C	-9.092786	-0.017821	0.368090
H	-9.370733	0.299951	-0.651076	H	8.790869	1.327191	-0.730221	H	-8.965630	-0.596987	1.285499
C	-9.874021	-1.179554	0.828248	H	9.422415	-0.301877	-0.628655	H	-9.458838	-0.705413	-0.390140
H	-9.351201	-1.959707	1.383770	C	9.949940	0.946112	1.038747	C	-10.125065	1.079581	0.605346
H	-10.183038	-0.429725	1.561421	H	9.454125	1.681285	1.674650	H	-9.701239	1.817584	1.288357
C	-11.123563	-1.792952	0.199659	H	10.192651	0.096291	1.682018	H	-10.318226	1.603847	-0.334026
H	-10.800109	-2.542588	-0.525048	C	11.253157	1.552146	0.517842	C	-11.457286	0.595728	1.178020
C	-11.959701	-2.485317	1.273463	H	10.997028	2.396724	-0.124649	H	-11.250224	0.079399	2.117242
H	-11.385392	-3.254773	1.784535	C	12.091389	2.070180	1.683084	C	-12.366401	1.785833	1.471668
H	-12.283961	-1.758167	2.017709	H	11.536667	2.796899	2.272411	H	-11.887804	2.487527	2.151165
C	-6.955038	1.639533	-1.178938	H	12.365629	1.245060	2.339873	H	-12.595223	2.316272	0.547887
H	-7.166505	2.452636	-0.483416	C	6.987164	-1.461654	-1.393431	C	-6.931741	-1.506685	-1.289257
H	-6.175436	1.973272	-1.857508	H	7.160130	-2.356522	-0.794741	H	-7.026209	-1.000634	-2.250582
H	-7.849560	1.452142	-1.767534	H	6.216906	-1.689535	-2.122801	H	-6.134886	-2.237925	-1.375934
C	-11.971139	-0.754051	-0.530703	H	7.900694	-1.233644	-1.936271	H	-7.857467	-2.042143	-1.092751
H	-12.269962	0.035374	0.158717	C	12.062701	0.550957	-0.302252	C	-12.162080	-0.379068	0.238451
H	-11.431828	-0.297808	-1.355074	H	12.271784	-0.336426	0.295088	H	-12.310888	0.086628	-0.735850
H	-12.874640	-1.209390	-0.930572	H	11.534503	0.238012	-1.197752	H	-11.587343	-1.288586	0.092897
C	9.275935	-0.449911	1.967189	H	13.014151	0.982437	-0.606590	H	-13.138318	-0.656287	0.631074
H	10.085345	0.138611	1.529592	C	-9.289164	-0.438066	1.954290	C	9.137948	2.385142	-0.250694
H	9.014338	0.015752	2.907957	H	-10.051102	-0.938936	1.353739	H	9.869223	2.028665	-0.978732
H	9.667443	-1.442855	2.193325	H	-8.973416	-1.136505	2.717545	H	8.700395	3.288765	-0.653377

C	6.619862	0.718730	2.695756	H	-9.761194	0.404789	2.461039	H	9.674809	2.661464	0.657637
H	5.622672	1.139903	2.717644	C	-6.550024	-1.541611	2.321542	C	6.270864	2.825861	-0.906520
H	6.717184	0.031771	3.535131	H	-5.515072	-1.847613	2.254200	H	5.209465	2.771562	-1.105491
H	7.336200	1.525296	2.837564	H	-6.728103	-1.163199	3.326784	H	6.451885	3.680439	-0.256684
C	7.407713	-1.978488	-2.398963	H	-7.178701	-2.418613	2.179967	H	6.787595	3.007957	-1.846414
H	6.901407	-2.942287	-2.420891	C	-7.617237	2.493635	-1.732161	C	8.037403	-2.149906	1.539409
H	7.001229	-1.382950	-3.212283	H	-7.233062	3.468201	-1.432798	H	7.750856	-2.199632	2.589344
H	8.462685	-2.144097	-2.580071	H	-7.135064	2.229715	-2.669869	H	7.586302	-3.003143	1.039598
O	9.469991	-1.703420	-0.544586	H	-8.682399	2.588895	-1.901219	H	9.114675	-2.239680	1.479671
H	10.110719	-1.493216	0.136966	O	-9.627804	1.490588	-0.072978	O	9.815609	-0.090482	0.913289
H	4.771926	-1.924478	-2.093052	H	-10.243643	1.011171	0.480317	H	10.338128	0.635122	0.573294
H	2.628033	-0.501394	-1.649802	H	-4.977978	2.587839	-1.383142	H	5.411268	-2.096349	1.980335
C	3.968358	1.843486	-0.940515	H	-2.746958	1.302966	-1.408839	H	3.063348	-1.957041	0.944567
H	3.234472	1.987554	-1.730358	C	-3.864068	-1.286707	-1.466405	C	3.850044	-1.082475	-1.594914
H	3.961590	2.717900	-0.292079	H	-3.179966	-1.097212	-2.289305	H	3.266819	-1.998725	-1.607739
H	4.961810	1.733052	-1.368508	H	-3.738711	-2.315933	-1.141577	H	3.562421	-0.480813	-2.452647
H	-1.372342	0.534505	-1.178820	H	-4.886533	-1.151219	-1.810652	H	4.903979	-1.333214	-1.683286
C	-1.447190	2.667704	-1.118120	H	1.469894	-0.352668	-1.462945	H	-1.521676	-1.212858	0.529850
H	-2.294659	2.800191	-1.789330	C	1.507035	-2.481271	-1.425492	C	-1.468691	-1.813865	-1.611976
H	-1.470035	3.467128	-0.374614	H	2.347817	-2.544658	-2.118858	H	-2.343445	-2.463243	-1.619467
H	-0.532827	2.764465	-1.696011	H	1.571757	-3.290547	-0.698039	H	-0.582796	-2.447432	-1.566845
H	-12.847077	-2.947357	0.846742	H	0.597883	-2.624968	-2.003733	H	-13.306315	1.464477	1.915778
C	-0.206280	1.181345	0.465535	H	13.008228	2.539651	1.332721	C	-0.295909	0.137430	-0.542746
H	-0.332896	1.935170	1.281138	C	0.252211	-1.040747	0.217780	H	-0.317869	0.676310	-1.492876
H	-0.260573	0.211855	0.969366	H	0.353909	-0.160211	0.859630	H	-0.367760	0.868860	0.271246
**α-Tocopherol ($C11^-$)**				**α-Tocopherol ($C12^-$)**				**α-Tocopherol ($C13^-$)**			
C	3.586525	-0.421824	-0.343744	C	3.568556	-0.391040	-0.359951	C	-3.534504	-0.312908	-0.335657
C	5.762282	0.580951	-0.014714	C	5.755833	0.572011	-0.002946	C	-5.780719	0.031336	0.480520
C	6.205155	-0.515335	0.722283	C	6.158585	-0.517470	0.765617	C	-6.055062	0.949456	-0.528563
C	5.229995	-1.579463	1.154353	C	5.152141	-1.551876	1.198115	C	-4.949150	1.463454	-1.411119
C	3.789848	-1.113394	0.994393	C	3.725910	-1.059699	0.996439	C	-3.576980	1.122866	-0.841233
C	6.643974	1.598977	-0.394987	C	6.665964	1.562125	-0.387744	C	-6.788526	-0.431100	1.330421
C	7.555991	-0.622654	1.060691	C	7.499368	-0.646760	1.134471	C	-7.363942	1.391754	-0.724072
H	5.394994	-2.485264	0.568729	H	5.310895	-2.473340	0.635856	H	-5.052048	1.041632	-2.411600
H	3.537489	-0.398060	1.775816	H	3.472935	-0.322756	1.757267	H	-3.351699	1.773105	0.003572
C	8.422289	0.388768	0.682519	C	8.394698	0.337862	0.753081	C	-8.360657	0.927889	0.121304
C	7.984032	1.502128	-0.030675	C	7.995892	1.444361	0.006353	C	-8.086557	0.034384	1.155396
O	4.457917	0.740213	-0.382572	O	4.460544	0.751389	-0.399929	O	-4.522422	-0.447003	0.716815
C	2.183435	0.150690	-0.476812	C	2.181933	0.210688	-0.538452	C	-2.213526	-0.633094	0.344542
H	2.150528	0.731584	-1.399006	H	2.196412	0.800241	-1.455580	H	-2.333009	-1.586394	0.859190
H	2.033429	0.851671	0.345062	H	2.014218	0.909105	0.281525	H	-2.048119	0.120905	1.113594
C	1.052129	-0.874292	-0.482158	C	1.030509	-0.789063	-0.602746	C	-1.003274	-0.709013	-0.577455
H	1.231560	-1.620764	-1.254063	H	1.202486	-1.496742	-1.411819	H	-1.147522	-1.500133	-1.310102
H	0.996181	-1.398627	0.470757	H	0.978390	-1.367027	0.320383	H	-0.888731	0.221437	-1.135432
C	-1.502232	-1.113211	-0.540870	C	-1.545439	-0.979259	-0.737873	C	1.532070	-1.049074	-0.684775
C	-2.745759	-0.284856	-0.710951	C	-2.791933	-0.169252	-0.976971	C	2.813377	-1.048132	0.161011
H	-2.851222	0.049081	-1.779601	H	-2.708546	0.782679	-0.440005	H	2.703926	-1.902833	0.874350
H	-2.656935	0.635914	-0.127200	C	-4.043072	-0.876426	-0.518609	H	2.789967	-0.152120	0.791753
C	-4.062047	-0.957052	-0.319388	H	-4.004188	-1.197524	0.554530	C	4.102883	-1.062281	-0.625702
H	-4.008355	-1.220744	0.737996	H	-4.186263	-1.803241	-1.078061	H	4.242690	-2.050448	-1.074812
H	-4.163537	-1.889607	-0.870194	C	-5.291202	-0.013249	-0.689297	C	5.273377	-0.753508	0.276286
C	-5.277150	-0.068137	-0.573470	H	-5.069684	0.981315	-0.296160	H	5.034075	0.128021	0.881243
H	-5.070774	0.923255	-0.163896	H	-5.479103	0.117346	-1.756894	H	5.508050	-1.557912	1.017738
H	-5.412920	0.068723	-1.649307	C	-6.562101	-0.532071	-0.010271	C	6.594988	-0.449956	-0.443709
C	-6.591291	-0.572852	0.027487	H	-6.365875	-0.610975	1.062523	H	6.404408	0.412686	-1.086431
H	-6.441792	-0.692687	1.103890	C	-7.701460	0.469534	-0.208679	C	7.686722	-0.083188	0.562300
C	-7.693387	0.467039	-0.183210	H	-7.350832	1.453728	0.106500	H	7.287562	0.678492	1.236048
H	-7.333464	1.429297	0.184577	H	-7.915338	0.547736	-1.277660	H	7.905471	-0.959338	1.178354
H	-7.857708	0.589719	-1.256834	C	-8.992416	0.147103	0.538892	C	8.984779	0.429223	-0.053063
C	-9.023164	0.151552	0.495427	H	-8.768170	-0.003684	1.597434	H	8.746511	1.246003	-0.738970
H	-8.847864	-0.049414	1.554755	H	-9.404195	-0.788696	0.168267	H	9.442960	-0.358833	-0.645226
H	-9.445486	-0.754938	0.068739	C	-10.029513	1.256334	0.396635	C	9.954592	0.940851	1.005347
C	-10.021137	1.296662	0.358501	H	-9.571518	2.202143	0.690420	H	9.421930	1.642524	1.648202
H	-9.547554	2.216587	0.704895	H	-10.302850	1.361332	-0.656592	H	10.268298	0.113626	1.647799
H	-10.253464	1.449158	-0.698634	C	-11.306361	1.057990	1.214156	C	11.198635	1.646659	0.462253
C	-11.330968	1.104764	1.123272	H	-11.020655	0.953731	2.262596	H	10.865881	2.442225	-0.207260
H	-11.085305	0.951879	2.175798	C	-12.211667	2.279044	1.079231	C	11.982265	2.279417	1.608923
C	-12.196077	2.356353	1.006057	H	-11.697052	3.185366	1.390239	H	11.372033	2.994227	2.157717
H	-11.668341	3.234235	1.371759	H	-12.513583	2.404929	0.039805	H	12.305057	1.507670	2.307540
H	-12.456901	2.532192	-0.037227	C	-6.937164	-1.918457	-0.527814	C	7.004986	-1.620358	-1.325956
C	-6.983389	-1.929042	-0.553952	H	-7.123955	-1.872700	-1.601673	H	7.137151	-2.517554	-0.716472
H	-7.145477	-1.837585	-1.628786	H	-6.123519	-2.617039	-0.360038	H	6.210837	-1.805220	-2.044412
H	-6.194895	-2.657525	-0.395193	H	-7.826940	-2.313090	-0.041215	H	7.929736	-1.433106	-1.870840
H	-7.893954	-2.317448	-0.103692	C	-12.062337	-0.199770	0.794328	C	12.103495	0.699137	-0.322344
C	-12.106982	-0.113064	0.628793	H	-12.295369	-0.154534	-0.269642	H	12.395192	-0.138746	0.309979

H	-12.305217	-0.018911	-0.438965	H	-11.481344	-1.099131	0.973729	H	11.610371	0.301142	-1.203147
H	-11.557854	-1.035646	0.789708	H	-12.999568	-0.287842	1.340550	H	13.009057	1.209469	-0.644804
H	-13.063039	-0.195728	1.141883	C	9.028154	2.481001	-0.355253	C	-9.208559	-0.412755	2.057432
C	8.985187	2.570024	-0.389489	H	9.803453	2.065209	-1.001319	H	-9.963948	-0.973513	1.505868
H	9.757385	2.186678	-1.059025	H	8.585173	3.315583	-0.881937	H	-8.845948	-1.053940	2.853397
H	8.512329	3.404638	-0.889670	H	9.512002	2.882472	0.536071	H	-9.697268	0.438025	2.531997
H	9.477025	2.964227	0.500832	C	6.191940	2.730102	-1.213429	C	-6.444817	-1.406080	2.422074
C	6.127140	2.774160	-1.184121	H	5.150216	2.603671	-1.473714	H	-5.405523	-1.693901	2.343953
H	5.082736	2.629758	-1.423951	H	6.293322	3.665160	-0.665297	H	-6.602337	-0.965266	3.403503
H	6.220431	3.699256	-0.617696	H	6.767859	2.819128	-2.131957	H	-7.058868	-2.301324	2.356241
H	6.679975	2.896136	-2.113101	C	7.962282	-1.829316	1.940628	C	-7.684452	2.357187	-1.831563
C	8.060396	-1.811753	1.831761	H	7.621464	-1.755584	2.972668	H	-7.267566	3.342219	-1.627305
H	7.733500	-1.770531	2.870156	H	7.563348	-2.754378	1.532527	H	-7.262289	2.016322	-2.774464
H	7.677228	-2.736578	1.408137	H	9.043256	-1.887870	1.946282	H	-8.756128	2.459483	-1.945310
H	9.142452	-1.843774	1.819890	O	9.711704	0.194393	1.145871	O	-9.644565	1.397482	-0.079480
O	9.751100	0.266097	1.045017	H	10.227820	0.904535	0.765625	H	-10.234756	0.982646	0.555334
H	10.242023	0.997066	0.671221	H	5.313174	-1.802577	2.244282	H	-5.046312	2.540802	-1.528442
H	5.419544	-1.848713	2.191217	H	3.022907	-1.882466	1.081186	H	-2.807722	1.273144	-1.593921
H	3.103536	-1.950188	1.076124	C	3.933386	-1.323333	-1.508533	C	-3.858069	-1.319095	-1.434074
C	3.945114	-1.322920	-1.519012	H	3.384823	-2.257405	-1.425340	H	-3.216873	-1.164478	-2.297272
H	3.419629	-2.270286	-1.440310	H	3.676916	-0.852471	-2.453606	H	-3.706978	-2.328807	-1.060840
H	3.652520	-0.840666	-2.447685	H	4.998385	-1.540624	-1.507542	H	-4.891025	-1.222480	-1.751473
H	5.014714	-1.515413	-1.548392	H	-1.529453	-1.436703	0.284235	H	1.575701	-0.160792	-1.318740
C	-1.473610	-2.211188	-1.572247	C	-1.419301	-2.150863	-1.720912	C	1.508452	-2.278997	-1.583182
H	-2.348723	-2.857723	-1.526413	H	-2.348774	-2.712720	-1.757010	H	2.384531	-2.252270	-2.224989
H	-1.444152	-1.783748	-2.612943	H	-1.232680	-1.756761	-2.720397	H	1.552285	-3.182675	-0.971339
H	-0.604136	-2.860596	-1.478156	H	-0.620230	-2.844949	-1.446573	H	0.618571	-2.325214	-2.209888
H	-13.120831	2.251697	1.569858	H	-13.113040	2.172017	1.679678	H	12.871731	2.793716	1.246684
C	-0.309421	-0.219842	-0.727834	C	-0.307222	-0.084869	-0.823620	C	0.286836	-0.970915	0.198108
H	-0.281279	0.189399	-1.776283	H	-0.314852	0.393663	-1.804712	H	0.182376	-1.909709	0.745666
H	-0.391404	0.651400	-0.072552	H	-0.413609	0.713199	-0.086055	H	0.432216	-0.185309	0.944402
**α-Tocopherol (C14$^-$)**				**α-Tocopherol (C15$^-$)**				**α-Tocopherol (C16$^-$)**			
C	3.551007	-0.467597	-0.198396	C	3.574800	-0.355985	-0.332016	C	3.566510	-0.419442	-0.293549
C	5.757572	0.506946	-0.141754	C	5.784096	0.544110	0.029123	C	5.761060	0.549905	-0.034992
C	6.099841	-0.229105	0.989013	C	6.184507	-0.600393	0.712527	C	6.145855	-0.451757	0.851382
C	5.046246	-1.002087	1.738825	C	5.171176	-1.644136	1.104660	C	5.125936	-1.418359	1.394538
C	3.644690	-0.586723	1.314793	C	3.749357	-1.116699	0.973370	C	3.706157	-0.938441	1.129322
C	6.711618	1.257188	-0.835935	C	6.700498	1.540685	-0.318576	C	6.684730	1.474986	-0.531067
C	7.425721	-0.247832	1.427279	C	7.531821	-0.778034	1.033981	C	7.484129	-0.559877	1.235278
H	5.186406	-2.070555	1.568158	H	5.298012	-2.530069	0.480694	H	5.278568	-2.400494	0.944392
H	3.400143	0.387064	1.736006	H	3.529766	-0.427851	1.787705	H	3.457266	-0.119673	1.802594
C	8.366143	0.499609	0.738404	C	8.435566	0.212925	0.689537	C	8.394317	0.360853	0.744242
C	8.026848	1.259034	-0.379402	C	8.038575	1.372632	0.026997	C	8.012927	1.380921	-0.124984
O	4.475413	0.561185	-0.621167	O	4.477457	0.772039	-0.316691	O	4.463361	0.703507	-0.449081
C	2.191038	0.053688	-0.644294	C	2.196319	0.282578	-0.437939	C	2.188475	0.178220	-0.545611
H	2.261740	0.275502	-1.709433	H	2.206639	0.934750	-1.311672	H	2.226316	0.688457	-1.508321
H	2.013558	1.001369	-0.136450	H	2.058078	0.923618	0.432468	H	2.015443	0.943931	0.210337
C	1.014021	-0.886752	-0.403004	C	1.026865	-0.690472	-0.551108	C	1.030601	-0.814694	-0.547139
H	1.189255	-1.829977	-0.914933	H	1.175041	-1.345589	-1.406370	H	1.195252	-1.567354	-1.314561
H	0.920489	-1.113294	0.659679	H	0.975740	-1.326291	0.333248	H	0.977457	-1.338438	0.407716
C	-1.551480	-1.086205	-0.545308	C	-1.542880	-0.837820	-0.646795	C	-1.535157	-1.011276	-0.658967
C	-2.804195	-0.322658	-0.984628	C	-2.804234	0.026533	-0.711845	C	-2.809336	-0.173743	-0.796285
H	-2.793325	-0.202492	-2.069729	H	-2.843100	0.516836	-1.688370	H	-2.858452	0.228406	-1.811444
H	-2.761913	0.685095	-0.566237	H	-2.715480	0.823516	0.029913	H	-2.729813	0.687021	-0.128794
C	-4.136967	-0.946148	-0.576535	C	-4.112435	-0.721173	-0.475081	C	-4.107690	-0.909485	-0.475160
H	-4.045250	-1.251216	0.497638	H	-4.049972	-1.274335	0.466423	H	-4.045020	-1.356374	0.518418
H	-4.262225	-1.883698	-1.121580	H	-4.286183	-1.444613	-1.266038	H	-4.257179	-1.722845	-1.181710
C	-5.301942	-0.020927	-0.834639	C	-5.314921	0.210730	-0.427924	C	-5.329667	0.008877	-0.510969
H	-5.092964	0.942093	-0.354796	H	-5.124736	1.000835	0.309011	H	-5.122721	0.871476	0.128402
C	-6.601668	-0.560057	-0.292346	H	-5.439067	0.677414	-1.408255	H	-5.394333	0.417147	-1.558662
H	-6.495865	-0.919303	0.761320	C	-6.627253	-0.500193	-0.085807	C	-6.638172	-0.614852	-0.113441
C	-7.665527	0.539295	-0.270233	H	-6.494325	-0.862614	0.963066	C	-7.682553	0.465577	-0.074725
H	-7.239607	1.410208	0.232038	C	-7.718449	0.570136	0.000773	H	-7.300167	1.326060	0.481906
H	-7.853896	0.840677	-1.302445	H	-7.412344	1.344798	0.714525	H	-7.886450	0.848941	-1.112980
C	-8.982995	0.177216	0.412346	H	-7.808696	1.030783	-0.986167	C	-9.032880	0.085071	0.532797
H	-8.783282	-0.162345	1.431474	C	-9.078134	0.026391	0.417262	H	-8.864546	-0.240756	1.560379
H	-9.447843	-0.656790	-0.109854	H	-8.967805	-0.543093	1.344625	H	-9.441699	-0.768516	-0.004631
C	-9.950680	1.356437	0.457244	H	-9.411614	-0.666098	-0.350176	C	-10.033317	1.237605	0.508512
H	-9.428046	2.220926	0.870911	C	-10.112097	1.129133	0.619254	H	-9.549673	2.126130	0.920768
H	-10.235338	1.629721	-0.562369	H	-9.703031	1.878352	1.301018	H	-10.286428	1.480380	-0.526890
C	-11.222377	1.121858	1.272826	H	-10.284856	1.639139	-0.332602	C	-11.328881	0.990729	1.282243
H	-10.923222	0.862871	2.290399	C	-11.457724	0.656739	1.170535	H	-11.063658	0.766150	2.317355
C	-12.062355	2.395157	1.325310	H	-11.269974	0.150864	2.119897	C	-12.204156	2.241408	1.267458
H	-11.490468	3.224214	1.736519	C	-12.374906	1.848796	1.431803	H	-11.672149	3.095285	1.681835
H	-12.378332	2.672764	0.319919	H	-11.911191	2.558026	2.114615	H	-12.481966	2.486325	0.242202

C	-7.104766	-1.765023	-1.096191	H	-12.580364	2.369851	0.496855	C	-7.027533	-1.662428	-1.123542
H	-7.340936	-1.437041	-2.108899	C	-6.965681	-1.567946	-1.093556	H	-7.096145	-1.217603	-2.155485
H	-6.329678	-2.523971	-1.166066	H	-6.196624	-2.336417	-1.168526	H	-6.313758	-2.482670	-1.184709
H	-7.984954	-2.231829	-0.647915	H	-7.913936	-2.061185	-0.883048	H	-7.997314	-2.111239	-0.914133
C	-12.054610	-0.027945	0.711540	C	-12.141399	-0.330592	0.228789	C	-12.107653	-0.195793	0.720843
H	-12.306791	0.172342	-0.330118	H	-12.275933	0.126589	-0.751659	H	-12.326512	-0.028048	-0.334113
H	-11.514720	-0.968938	0.751041	H	-11.550216	-1.231174	0.097428	H	-11.539759	-1.117247	0.802427
H	-12.983691	-0.143647	1.267757	H	-13.122222	-0.614523	0.607939	H	-13.053032	-0.326396	1.246066
C	9.106545	2.059335	-1.060705	C	9.082280	2.409635	-0.298140	C	9.062137	2.353550	-0.597289
H	9.881074	1.412712	-1.476823	H	9.835173	2.016617	-0.983556	H	9.832135	1.854767	-1.188459
H	8.710235	2.650380	-1.875260	H	8.645963	3.281021	-0.767237	H	8.634944	3.130404	-1.216919
H	9.582740	2.749097	-0.362936	H	9.592455	2.749639	0.603857	H	9.549625	2.844062	0.245871
C	6.301862	2.047878	-2.051334	C	6.228531	2.770387	-1.050361	C	6.230432	2.550853	-1.483324
H	5.261545	1.865866	-2.282696	H	5.173873	2.691903	-1.274580	H	5.185631	2.417723	-1.727298
H	6.430233	3.115920	-1.885279	H	6.382028	3.665718	-0.450859	H	6.352914	3.539333	-1.044447
H	6.899826	1.772054	-2.917184	H	6.769217	2.900174	-1.985133	H	6.805234	2.523895	-2.406387
C	7.824655	-1.055474	2.631718	C	7.992564	-2.017716	1.750174	C	7.927952	-1.647738	2.174318
H	7.460744	-0.594017	3.548873	H	7.678473	-2.004641	2.793233	H	7.583489	-1.451330	3.188812
H	7.404530	-2.056688	2.582496	H	7.568072	-2.908145	1.293950	H	7.520676	-2.609377	1.872952
H	8.902291	-1.133055	2.698993	H	9.071942	-2.094510	1.725017	H	9.007958	-1.717657	2.193590
O	9.665542	0.477140	1.202299	O	9.757368	0.019807	1.033861	O	9.706406	0.243042	1.153932
H	10.220816	0.969718	0.598561	H	10.280206	0.740858	0.684283	H	10.239648	0.885111	0.686060
H	5.170948	-0.845991	2.808018	H	5.353074	-1.964345	2.127879	H	5.278760	-1.547955	2.463459
H	2.911522	-1.301832	1.675407	H	3.036339	-1.934145	1.023843	H	2.996602	-1.741922	1.302105
C	3.918996	-1.766363	-0.904989	C	3.893794	-1.216160	-1.548986	C	3.925399	-1.473315	-1.333361
H	3.341479	-2.595366	-0.504777	H	3.340050	-2.150435	-1.511508	H	3.369971	-2.390252	-1.155406
H	3.709736	-1.675028	-1.967500	H	3.620690	-0.682985	-2.455707	H	3.682571	-1.103723	-2.326163
H	4.975625	-1.986510	-0.780226	H	4.955370	-1.443119	-1.590331	H	4.987583	-1.698911	-1.301286
H	-1.588969	-1.200519	0.541512	H	-1.543980	-1.358439	0.314701	H	-1.527270	-1.445412	0.344380
C	-1.507443	-2.479141	-1.169237	C	-1.529205	-1.883896	-1.759342	C	-1.506003	-2.150490	-1.675531
H	-2.412606	-3.027935	-0.930177	H	-2.431607	-2.485652	-1.736330	H	-2.393243	-2.768647	-1.588306
H	-1.442364	-2.395262	-2.255029	H	-1.482095	-1.389472	-2.730279	H	-1.479571	-1.742113	-2.686503
H	-0.657418	-3.061935	-0.819000	H	-0.677349	-2.555112	-1.676714	H	-0.637771	-2.792310	-1.544237
H	-12.955798	2.258170	1.932375	H	-13.326767	1.533390	1.856705	H	-13.119954	2.095066	1.838631
C	-0.299038	-0.278414	-0.890796	C	-0.299311	0.051048	-0.699825	C	-0.303470	-0.114070	-0.793689
H	-0.253444	-0.147018	-1.974572	H	-0.300063	0.598913	-1.645051	H	-0.299226	0.328999	-1.792478
H	-0.404202	0.719759	-0.463279	H	-0.374008	0.799045	0.091023	H	-0.399264	0.713018	-0.088972
**α-Tocopherol ($C17^-$)**				**α-Tocopherol ($C18^-$)**				**α-Tocopherol ($C19^-$)**			
C	3.544069	-0.413695	-0.303266	C	3.553497	-0.425531	-0.289755	C	3.548600	-0.411998	-0.235920
C	5.730551	0.564386	-0.016231	C	5.743619	0.554096	-0.041473	C	5.760159	0.529771	-0.053139
C	6.123232	-0.459895	0.840331	C	6.126591	-0.428610	0.866205	C	6.123073	-0.403128	0.913371
C	5.111235	-1.451328	1.352896	C	5.106987	-1.386862	1.424581	C	5.083047	-1.300121	1.533015
C	3.688158	-0.972385	1.104154	C	3.687015	-0.916545	1.143692	C	3.673648	-0.814680	1.225646
C	6.646996	1.511520	-0.482744	C	6.666869	1.470583	-0.553220	C	6.703305	1.387347	-0.625856
C	7.461821	-0.568142	1.222917	C	7.463455	-0.526435	1.257505	C	7.458933	-0.509812	1.305443
H	5.268634	-2.416636	0.869584	H	5.263975	-2.377377	0.994789	H	5.214630	-2.318647	1.164799
H	3.435690	-0.174696	1.801138	H	3.432654	-0.085640	1.799889	H	3.441477	0.062187	1.828066
C	8.364616	0.374648	0.761302	C	8.373856	0.385624	0.750642	C	8.389556	0.344267	0.737832
C	7.975340	1.417148	-0.077033	C	7.993837	1.386986	-0.140749	C	8.030257	1.295540	-0.214703
O	4.431869	0.719501	-0.427457	O	4.445832	0.696589	-0.463163	O	4.463627	0.677702	-0.478450
C	2.161770	0.180383	-0.539180	C	2.175312	0.164198	-0.560424	C	2.182624	0.187947	-0.544091
H	2.193863	0.707557	-1.492983	H	2.216445	0.652229	-1.534483	H	2.241922	0.634435	-1.536866
H	1.985349	0.931137	0.230649	H	1.998418	0.946620	0.177155	H	2.009448	1.002463	0.158767
C	1.010911	-0.820645	-0.555270	C	1.019545	-0.830652	-0.543689	C	1.012403	-0.789379	-0.501941
H	1.192517	-1.574696	-1.317345	H	1.194862	-1.607954	-1.283674	H	1.177468	-1.584380	-1.224820
H	0.947256	-1.340893	0.400892	H	0.954863	-1.322476	0.427329	H	0.943009	-1.258626	0.479783
C	-1.553441	-1.027734	-0.684726	C	-1.544116	-1.029079	-0.661042	C	-1.551823	-0.965944	-0.663138
C	-2.825181	-0.209003	-0.918209	C	-2.814253	-0.212788	-0.912253	C	-2.809931	-0.111287	-0.834014
H	-2.866577	0.082162	-1.971064	H	-2.871956	0.028414	-1.976925	H	-2.826148	0.291586	-1.849947
H	-2.752040	0.718441	-0.347457	H	-2.727971	0.739552	-0.386547	H	-2.739410	0.747290	-0.164532
C	-4.127755	-0.904663	-0.533119	C	-4.110705	-0.886044	-0.472080	C	-4.125113	-0.833402	-0.556924
H	-4.064522	-1.226896	0.508658	H	-4.037632	-1.141571	0.587132	H	-4.075814	-1.305168	0.427257
H	-4.262142	-1.803734	-1.131203	H	-4.247553	-1.821123	-1.010223	H	-4.267103	-1.631215	-1.281398
C	-5.337135	0.009452	-0.712238	C	-5.330247	0.006810	-0.686433	C	-5.322637	0.111963	-0.600491
H	-5.154638	0.944880	-0.179117	H	-5.138617	0.976413	-0.221013	H	-5.127948	0.955226	0.064241
H	-5.448417	0.270997	-1.766589	H	-5.453388	0.197080	-1.755479	H	-5.416918	0.528574	-1.606535
C	-6.675467	-0.553996	-0.229883	C	-6.640333	-0.545802	-0.121054	C	-6.660472	-0.514845	-0.202060
H	-6.526639	-0.844707	0.841235	H	-6.498049	-0.776210	0.936727	H	-6.543772	-0.950570	0.793994
C	-7.764983	0.469828	-0.390237	C	-7.755440	0.503811	-0.193432	C	-7.739751	0.568299	-0.122713
H	-7.389423	1.448944	-0.073610	H	-7.397025	1.393550	0.335396	H	-7.397211	1.348910	0.559969
C	-9.002014	0.148992	0.407424	H	-7.813430	0.814899	-1.268078	H	-7.847877	1.044205	-1.099617
H	-8.793903	-0.009699	1.497986	C	-9.080516	0.083785	0.396640	C	-9.116928	0.104002	0.346583
H	-9.451243	-0.790335	0.076352	H	-9.543395	-0.661054	-0.255760	H	-8.956837	-0.587259	1.215692
C	-10.060078	1.244517	0.299186	C	-10.006148	1.271199	0.517874	H	-9.553396	-0.526144	-0.431093
H	-9.559857	2.210971	0.390509	H	-9.475005	2.090445	1.014649	C	-10.031088	1.259450	0.667522

H	-10.489302	1.218436	-0.704862	H	-10.330814	1.696596	-0.466432	H	-9.529119	1.919952	1.383548
C	-11.185408	1.180612	1.332132	C	-11.293884	1.019136	1.306549	C	-11.347489	0.820743	1.245185
H	-10.741300	1.297181	2.322975	H	-10.991860	0.692859	2.302041	H	-11.217172	0.089971	2.082900
C	-12.177773	2.319686	1.112203	C	-12.135723	2.288643	1.423090	C	-12.115986	2.009454	1.817974
H	-11.673573	3.283723	1.137227	H	-11.563384	3.092742	1.884165	H	-11.521771	2.518320	2.576113
H	-12.649265	2.216754	0.134815	H	-12.446505	2.625725	0.433737	H	-12.314512	2.720881	1.016602
C	-6.994536	-1.858979	-0.973047	C	-7.072646	-1.823984	-0.830785	C	-7.068349	-1.628489	-1.163341
H	-6.964366	-1.671906	-2.046583	H	-7.283251	-1.608645	-1.881104	H	-7.208735	-1.217645	-2.164147
H	-6.300146	-2.663885	-0.720780	H	-6.315543	-2.606297	-0.784641	H	-6.324287	-2.420986	-1.218400
H	-7.997106	-2.197639	-0.724674	H	-7.981368	-2.194707	-0.365594	H	-8.008577	-2.070873	-0.849517
C	-11.910980	-0.161212	1.295833	C	-12.109427	-0.099105	0.668704	C	-12.240476	0.112715	0.220810
H	-12.301184	-0.344419	0.294053	H	-12.336939	0.157221	-0.368487	H	-12.441556	0.799987	-0.601311
H	-11.235334	-0.974082	1.545919	H	-11.537007	-1.022248	0.677616	H	-11.748374	-0.766409	-0.189559
H	-12.747149	-0.176828	1.994911	H	-13.051483	-0.259130	1.195495	H	-13.185551	-0.207186	0.669598
C	9.016085	2.413287	-0.518253	C	9.043277	2.351182	-0.629394	C	9.101215	2.198340	-0.769912
H	9.791076	1.939843	-1.123390	H	9.817826	1.840554	-1.204108	H	9.864208	1.630180	-1.304291
H	8.581969	3.204961	-1.114045	H	8.618064	3.112538	-1.269291	H	8.693135	2.920807	-1.463918
H	9.498255	2.882688	0.339962	H	9.524575	2.862438	0.204987	H	9.593607	2.758588	0.025932
C	6.185220	2.612181	-1.402497	C	6.214652	2.526535	-1.528437	C	6.273132	2.390688	-1.664531
H	5.141946	2.478897	-1.653054	H	5.169852	2.390260	-1.770645	H	5.222819	2.270244	-1.890682
H	6.298782	3.587472	-0.933030	H	6.338606	3.523798	-1.110591	H	6.429717	3.408627	-1.313096
H	6.761838	2.618511	-2.324682	H	6.790050	2.479037	-2.450269	H	6.838613	2.266648	-2.585297
C	7.913864	-1.681364	2.127601	C	7.905486	-1.593606	2.220710	C	7.879330	-1.525014	2.332358
H	7.569267	-1.519181	3.148032	H	7.555157	-1.377560	3.229156	H	7.540194	-1.237282	3.326589
H	7.513027	-2.635846	1.796362	H	7.503062	-2.562581	1.936850	H	7.451360	-2.499494	2.111582
H	8.994274	-1.744630	2.143925	H	8.985522	-1.659738	2.247188	H	8.957501	-1.616764	2.358612
O	9.676971	0.254989	1.168513	O	9.683893	0.277889	1.167056	O	9.698366	0.228607	1.154759
H	10.202863	0.925370	0.732977	H	10.217174	0.917736	0.696128	H	10.240404	0.846883	0.665326
H	5.266094	-1.614849	2.416741	H	5.255444	-1.494508	2.496479	H	5.232521	-1.344132	2.609190
H	2.983584	-1.784957	1.254323	H	2.979525	-1.719241	1.328954	H	2.947601	-1.586817	1.462532
C	3.909778	-1.435060	-1.373348	C	3.919662	-1.498767	-1.307279	C	3.891147	-1.554784	-1.183886
H	3.367852	-2.364127	-1.217385	H	3.368844	-2.415283	-1.113133	H	3.326477	-2.447605	-0.928107
H	3.657159	-1.042337	-2.354824	H	3.679552	-1.150191	-2.308368	H	3.652108	-1.268317	-2.204860
H	4.974761	-1.647243	-1.352788	H	4.982612	-1.718540	-1.266970	H	4.950450	-1.789196	-1.133227
H	-1.580331	-1.402025	0.342174	H	-1.568183	-1.374612	0.375804	H	-1.561738	-1.376285	0.350015
C	-1.480820	-2.224468	-1.630660	C	-1.478690	-2.250885	-1.575319	C	-1.527169	-2.127913	-1.653408
H	-2.383921	-2.823063	-1.568942	H	-2.387374	-2.840058	-1.504895	H	-2.407466	-2.753505	-1.546847
H	-1.377027	-1.876178	-2.658841	H	-1.363597	-1.930865	-2.611394	H	-1.507572	-1.744122	-2.673777
H	-0.636632	-2.871014	-1.403758	H	-0.642724	-2.899302	-1.325592	H	-0.654987	-2.760376	-1.511787
H	-12.963212	2.320674	1.867663	H	-13.034094	2.120779	2.018494	H	-13.063197	1.701090	2.266747
C	-0.322963	-0.129703	-0.826423	C	-0.311081	-0.140205	-0.832049	C	-0.308354	-0.085776	-0.803260
H	-0.310258	0.293218	-1.833966	H	-0.299638	0.252679	-1.851594	H	-0.277304	0.319918	-1.817187
H	-0.427947	0.711558	-0.140233	H	-0.409805	0.720799	-0.169972	H	-0.409427	0.767834	-0.131975
**α-Tocopherol (C20$^-$)**				**α-Tocopherol (C21$^-$)**				**α-Tocopherol (C22$^-$)**			
C	3.549539	-0.388463	-0.302409	C	3.543248	-0.435563	-0.331418	C	3.546436	-0.391898	-0.324654
C	5.757971	0.536216	-0.017103	C	5.720818	0.556810	-0.032518	C	5.752469	0.529995	-0.008727
C	6.120049	-0.488391	0.851809	C	6.114768	-0.465376	0.824613	C	6.109443	-0.507995	0.846305
C	5.081007	-1.445797	1.374622	C	5.108488	-1.465774	1.330642	C	5.068073	-1.476186	1.344016
C	3.670739	-0.937702	1.111148	C	3.682528	-0.999400	1.074894	C	3.659213	-0.964368	1.080364
C	6.699917	1.451028	-0.496041	C	6.630035	1.511548	-0.494845	C	6.696171	1.454923	-0.463945
C	7.454774	-0.629936	1.236538	C	7.451782	-0.564175	1.214460	C	7.441027	-0.652278	1.240781
H	5.219059	-2.423041	0.909496	H	5.277945	-2.430333	0.849952	H	5.210092	-2.444441	0.861696
H	3.431505	-0.128156	1.798970	H	3.418890	-0.206974	1.773887	H	3.415214	-0.166650	1.780293
C	8.384081	0.281352	0.763515	C	8.349318	0.387336	0.758984	C	8.371880	0.269689	0.792279
C	8.025216	1.323379	-0.089358	C	7.956846	1.427331	-0.081790	C	8.018036	1.324384	-0.047040
O	4.461874	0.722241	-0.430394	O	4.418239	0.703015	-0.448645	O	4.459581	0.720319	-0.430052
C	2.182940	0.237248	-0.551558	C	2.159036	0.155499	-0.569802	C	2.181549	0.238446	-0.571323
H	2.234926	0.762828	-1.505217	H	2.191623	0.684768	-1.522302	H	2.237012	0.774195	-1.519110
H	2.018599	0.992458	0.216653	H	1.983246	0.904822	0.201673	H	2.016400	0.985650	0.204507
C	1.010540	-0.737841	-0.575852	C	1.008825	-0.845336	-0.587011	C	1.007121	-0.733820	-0.608811
H	1.171218	-1.481408	-1.352619	H	1.183605	-1.591343	-1.358572	H	1.168623	-1.471390	-1.391173
H	0.943361	-1.273909	0.371038	H	0.952770	-1.375354	0.364026	H	0.935000	-1.277473	0.333371
C	-1.553589	-0.897799	-0.714907	C	-1.556327	-1.038339	-0.684785	C	-1.557802	-0.882593	-0.750490
C	-2.813747	-0.035598	-0.817870	C	-2.823843	-0.202026	-0.874125	C	-2.813811	-0.014766	-0.855602
H	-2.842410	0.429479	-1.806409	H	-2.892852	0.099962	-1.921997	H	-2.841401	0.446661	-1.845855
H	-2.736298	0.779212	-0.096528	H	-2.726852	0.717278	-0.294937	H	-2.731306	0.802435	-0.137431
C	-4.122195	-0.780699	-0.572445	C	-4.117092	-0.897374	-0.460184	C	-4.125876	-0.751849	-0.605098
H	-4.057924	-1.317588	0.376769	H	-4.040167	-1.202272	0.585515	H	-4.062256	-1.286633	0.345355
H	-4.268613	-1.528379	-1.347358	H	-4.252809	-1.804591	-1.042900	H	-4.279975	-1.500621	-1.377439
C	-5.325744	0.156652	-0.536932	C	-5.331099	0.010628	-0.632165	C	-5.322990	0.193466	-0.568363
H	-5.126080	0.957608	0.176579	H	-5.114901	0.973352	-0.166657	H	-5.114108	0.996430	0.140536
H	-5.440645	0.633969	-1.513451	H	-5.485792	0.209660	-1.695161	H	-5.439277	0.666688	-1.546661
C	-6.650836	-0.508935	-0.158721	C	-6.632986	-0.531606	-0.039170	C	-6.650318	-0.462291	-0.181668
H	-6.521925	-0.983744	0.817635	H	-6.467505	-0.709971	1.026362	H	-6.519935	-0.932025	0.797240
C	-7.748767	0.549560	-0.024051	C	-7.733066	0.522787	-0.175748	C	-7.740976	0.603091	-0.046821

H	-7.384482	1.341876	0.633555	H	-7.360312	1.466572	0.225137	H	-7.368469	1.398749	0.602587
H	-7.912635	1.012183	-1.000547	H	-7.924717	0.693250	-1.237933	H	-7.910469	1.057810	-1.026641
C	-9.077486	0.040620	0.529014	C	-9.045557	0.183359	0.523586	C	-9.070412	0.098795	0.505085
H	-8.911024	-0.477648	1.474917	H	-8.845699	-0.059703	1.569319	H	-8.894597	-0.435316	1.443833
H	-9.511544	-0.683011	-0.158013	H	-9.485976	-0.701810	0.072165	H	-9.535181	-0.598488	-0.187146
C	-10.089922	1.160503	0.766982	C	-10.038032	1.339245	0.454807	C	-10.071592	1.218197	0.747382
H	-9.614825	1.927747	1.384876	H	-9.553212	2.241151	0.831473	H	-9.628303	1.961399	1.423141
H	-10.275978	1.650613	-0.231855	H	-10.288843	1.537051	-0.590278	H	-10.296108	1.704420	-0.205320
C	-11.395622	0.749563	1.380036	C	-11.334675	1.120971	1.234678	C	-11.396473	0.711551	1.326980
C	-12.215651	1.985636	1.624422	H	-11.072222	0.927433	2.276381	H	-11.138051	0.334871	2.343011
H	-11.710901	2.696076	2.280977	C	-12.193860	2.380637	1.178702	C	-12.314018	1.913582	1.544825
H	-12.423094	2.531832	0.661724	H	-11.656826	3.241645	1.569732	H	-11.844787	2.677175	2.170883
C	-7.040785	-1.586302	-1.168506	C	-7.037944	-1.851279	-0.691442	H	-12.561933	2.343224	0.573988
H	-7.156483	-1.140244	-2.157161	H	-7.200824	-1.703620	-1.759347	C	-7.054177	-1.542130	-1.182984
H	-6.294438	-2.374672	-1.234710	H	-6.268645	-2.607815	-0.568297	H	-7.159145	-1.103666	-2.175969
H	-7.985787	-2.044566	-0.896952	H	-7.954128	-2.244791	-0.261534	H	-6.319832	-2.342499	-1.238356
C	-12.154091	-0.146127	0.440133	C	-12.124715	-0.073372	0.706284	H	-8.008614	-1.981161	-0.912715
H	-12.298774	0.353585	-0.559942	H	-12.339503	0.061640	-0.353698	C	-12.063277	-0.298583	0.424991
H	-11.659427	-1.096834	0.243972	H	-11.582307	-1.006508	0.825794	H	-11.538704	-1.254648	0.388134
H	-13.151106	-0.373582	0.819274	H	-13.072984	-0.168434	1.230154	H	-13.097451	-0.478108	0.727529
C	9.095321	2.281736	-0.543396	C	8.993414	2.430279	-0.515933	C	9.089643	2.292491	-0.476227
H	9.858709	1.775204	-1.136292	H	9.774576	1.961526	-1.116516	H	9.860983	1.796501	-1.067746
H	8.687604	3.074861	-1.155378	H	8.559525	3.220159	-1.113872	H	8.685931	3.092605	-1.081726
H	9.587227	2.751704	0.308777	H	9.467505	2.900810	0.345718	H	9.570934	2.752414	0.387406
C	6.270019	2.552122	-1.430507	C	6.165693	2.608878	-1.417262	C	6.271910	2.569629	-1.384720
H	5.223341	2.445780	-1.679213	H	5.126665	2.465216	-1.679174	H	5.228330	2.463745	-1.646219
H	6.412669	3.529950	-0.974453	H	6.264815	3.584452	-0.945149	H	6.406240	3.540409	-0.911392
H	6.846426	2.528740	-2.352544	H	6.752228	2.622949	-2.332933	H	6.858110	2.563271	-2.300833
C	7.874935	-1.742808	2.156885	C	7.908871	-1.673363	2.121390	C	7.856197	-1.779545	2.145758
H	7.531178	-1.558335	3.173808	H	7.561436	-1.511751	3.140986	H	7.506632	-1.611439	3.163548
H	7.450958	-2.691132	1.836795	H	7.516501	-2.631565	1.790783	H	7.434369	-2.722666	1.808036
H	8.953277	-1.833601	2.177930	H	8.989558	-1.728594	2.140515	H	8.934441	-1.870456	2.171559
O	9.691181	0.129882	1.173890	O	9.656867	0.277470	1.175355	O	9.675364	0.115307	1.212802
H	10.238819	0.773670	0.725234	H	10.187764	0.941009	0.735080	H	10.224909	0.769656	0.782029
H	5.226232	-1.595478	2.441872	H	5.258158	-1.627834	2.395333	H	5.206077	-1.645576	2.409252
H	2.948256	-1.733830	1.264853	H	2.986282	-1.820769	1.218247	H	2.936359	-1.763459	1.215921
C	3.897027	-1.428590	-1.360237	C	3.916431	-1.452788	-1.402723	C	3.899338	-1.415202	-1.396980
H	3.330219	-2.342131	-1.200948	H	3.374483	-2.383424	-1.255580	H	3.330446	-2.330426	-1.255894
H	3.664701	-1.038023	-2.347593	H	3.675412	-1.055337	-2.385257	H	3.673746	-1.008415	-2.379316
H	4.955828	-1.668734	-1.327471	H	4.980981	-1.665289	-1.372579	H	4.957656	-1.657126	-1.361339
H	-1.548857	-1.365345	0.273123	H	-1.561170	-1.430341	0.335509	H	-1.557523	-1.350919	0.237202
C	-1.544436	-2.001927	-1.769851	C	-1.514051	-2.215596	-1.656399	C	-1.550720	-1.985826	-1.806273
H	-2.417422	-2.640433	-1.680201	H	-2.409957	-2.824800	-1.585068	H	-2.429968	-2.616661	-1.723612
H	-1.549741	-1.561344	-2.767222	H	-1.435142	-1.848422	-2.679692	H	-1.544814	-1.544484	-2.803280
H	-0.665307	-2.634555	-1.683551	H	-0.663013	-2.862004	-1.463199	H	-0.677782	-2.626163	-1.714026
H	-13.187735	1.758988	2.064277	H	-13.109120	2.258056	1.752958	H	-13.244042	1.599763	2.017440
C	-0.310292	-0.013026	-0.822137	C	-0.324045	-0.145287	-0.838730	C	-0.310623	-0.002920	-0.853702
H	-0.290088	0.448534	-1.812018	H	-0.322700	0.282847	-1.843758	H	-0.287687	0.462147	-1.841866
H	-0.400492	0.801805	-0.102759	H	-0.413497	0.692247	-0.145927	H	-0.397984	0.809726	-0.131452

Structures of α-Tocopherol and their corresponding species in the SPLET (ETE_step) mechanism at M05-2X/6-311++G(2d,2p) level of theory in gas phase.

**M05-2X/6-311++G(2d,2p)**											
**α-Tocopherol (O$^{\bullet}$)**				**α-Tocopherol (C1$^{\bullet}$)**				**α-Tocopherol (C2$^{\bullet}$)**			
C	3.634060	-0.291274	-0.285060	C	3.640475	-0.368195	-0.299091	C	3.626392	-0.326251	-0.286404
C	5.847440	0.572568	0.069177	C	5.821328	0.603584	0.017448	C	5.834487	0.562972	0.019118
C	6.198734	-0.508670	0.869184	C	6.206059	-0.472250	0.811446	C	6.187106	-0.455323	0.865817
C	5.143396	-1.488417	1.322119	C	5.219956	-1.509774	1.270804	C	5.141918	-1.413241	1.381508
C	3.733022	-0.966495	1.079150	C	3.790113	-1.027243	1.067503	C	3.729711	-0.910035	1.118621
C	6.818068	1.468531	-0.383311	C	6.704362	1.578782	-0.425343	C	6.776597	1.503570	-0.522799
C	7.530070	-0.719161	1.220050	C	7.579994	-0.587938	1.127109	C	7.536667	-0.654690	1.243590
H	5.279310	-2.437213	0.797463	H	5.355048	-2.455217	0.732192	H	5.278416	-2.391665	0.913877
H	3.482038	-0.213045	1.825813	H	3.542883	-0.274325	1.816014	H	3.486708	-0.108913	1.816334
C	8.562123	0.173522	0.796914	C	8.459968	0.376226	0.717849	C	8.461419	0.256330	0.753028
C	8.143401	1.280349	-0.013810	C	8.077171	1.526095	-0.044955	C	8.128328	1.318064	-0.076985
O	4.511743	0.828875	-0.293848	O	4.483765	0.779032	-0.351776	O	4.515021	0.777516	-0.382793
C	2.256092	0.328210	-0.504910	C	2.241507	0.211360	-0.493876	C	2.253355	0.291633	-0.538410
H	2.314676	0.929848	-1.412880	H	2.261083	0.795153	-1.414999	H	2.318572	0.839402	-1.478890
H	2.071878	1.017884	0.318949	H	2.062818	0.914187	0.320003	H	2.077905	1.030160	0.243971
C	1.097397	-0.655842	-0.622520	C	1.102943	-0.800474	-0.558949	C	1.087032	-0.688479	-0.597223
H	1.284458	-1.338019	-1.448499	H	1.295940	-1.516001	-1.355087	H	1.265725	-1.415348	-1.386146

H	1.025830	-1.261332	0.281400	H	1.047820	-1.365699	0.371840	H	1.014430	-1.244532	0.338120
C	-1.466772	-0.831039	-0.783611	C	-1.463332	-1.013969	-0.662258	C	-1.478260	-0.856511	-0.758220
C	-2.733394	0.024586	-0.852690	C	-2.738068	-0.187197	-0.844039	C	-2.739898	0.005067	-0.843182
H	-2.766923	0.525330	-1.823295	H	-2.802695	0.134108	-1.886610	H	-2.765955	0.494396	-1.819788
H	-2.660573	0.812318	-0.101468	H	-2.653029	0.721894	-0.247083	H	-2.665799	0.801282	-0.101163
C	-4.034698	-0.741074	-0.633813	C	-4.028075	-0.902152	-0.453321	C	-4.046388	-0.751205	-0.622462
H	-3.971057	-1.295704	0.304863	H	-3.958116	-1.220901	0.588853	H	-3.987420	-1.303039	0.318279
H	-4.163204	-1.474584	-1.425061	H	-4.145786	-1.802888	-1.050104	H	-4.177964	-1.486704	-1.411377
C	-5.247100	0.183834	-0.589676	C	-5.251053	-0.006319	-0.625565	C	-5.253329	0.180992	-0.583393
H	-5.052596	0.979871	0.130504	H	-5.047776	0.954785	-0.151031	H	-5.057078	0.975717	0.137736
H	-5.367111	0.668980	-1.561457	H	-5.400830	0.200180	-1.687997	H	-5.366124	0.667106	-1.555549
C	-6.564041	-0.499488	-0.215117	C	-6.551419	-0.566007	-0.045521	C	-6.575949	-0.494663	-0.214760
H	-6.425736	-0.984473	0.754554	H	-6.392183	-0.746457	1.020618	H	-6.443566	-0.983752	0.753709
C	-7.664213	0.552736	-0.061931	C	-7.663762	0.474715	-0.187267	C	-7.669893	0.563955	-0.061205
H	-7.309308	1.328682	0.617777	H	-7.302688	1.424037	0.211064	H	-7.312596	1.334714	0.623118
H	-7.823302	1.037376	-1.028213	H	-7.855342	0.639531	-1.250414	H	-7.822267	1.053658	-1.025990
C	-8.995355	0.019174	0.458946	C	-8.973463	0.122469	0.511001	C	-9.006225	0.036295	0.452391
H	-8.824089	-0.534545	1.384534	H	-8.772967	-0.106720	1.559742	H	-8.841487	-0.523305	1.375671
H	-9.412895	-0.684319	-0.256757	H	-9.398653	-0.774273	0.067533	H	-9.425611	-0.660951	-0.268347
C	-9.999075	1.136995	0.720396	C	-9.983546	1.262105	0.427678	C	-10.004121	1.158718	0.716447
H	-9.536737	1.879161	1.372836	H	-9.511774	2.176214	0.791148	H	-9.539424	1.894904	1.373961
H	-10.224355	1.648331	-0.218729	H	-10.238319	1.441944	-0.619747	H	-10.223672	1.676078	-0.220711
C	-11.312323	0.679736	1.355448	C	-11.275986	1.035856	1.212124	C	-11.321790	0.705747	1.345377
H	-11.073268	0.169877	2.290495	H	-11.009487	0.861744	2.256176	H	-11.088244	0.189615	2.278378
C	-12.187545	1.888293	1.675099	C	-12.154859	2.281004	1.138756	C	-12.191172	1.917504	1.668919
H	-11.668621	2.588543	2.325569	H	-11.630324	3.155844	1.515778	H	-11.669937	2.611669	2.324050
H	-12.449033	2.412936	0.756824	H	-12.436466	2.477692	0.104769	H	-12.447473	2.448093	0.752599
C	-6.950085	-1.566371	-1.237192	C	-6.934537	-1.888370	-0.705609	C	-6.966242	-1.555665	-1.241401
H	-7.090606	-1.108078	-2.216566	H	-7.087157	-1.738950	-1.774885	H	-7.103396	-1.092784	-2.219098
H	-6.180390	-2.326546	-1.328404	H	-6.155818	-2.634039	-0.577112	H	-6.200063	-2.319065	-1.334915
H	-7.873403	-2.067850	-0.962875	H	-7.850358	-2.295208	-0.286645	H	-7.892330	-2.053745	-0.970085
C	-12.073255	-0.292046	0.457281	C	-12.048047	-0.178332	0.702637	C	-12.085659	-0.257002	0.439967
H	-12.258703	0.167030	-0.513799	H	-12.265378	-0.062487	-0.359154	H	-12.265663	0.208257	-0.529189
H	-11.523281	-1.214292	0.295930	H	-11.490186	-1.100489	0.835037	H	-11.540274	-1.181369	0.275270
H	-13.035110	-0.547945	0.896050	H	-12.994434	-0.280696	1.228947	H	-13.050235	-0.509798	0.874609
C	9.243192	2.212636	-0.434536	C	9.025194	2.482568	-0.419535	C	9.128173	2.328103	-0.551327
H	9.929853	1.711814	-1.118575	H	8.745011	3.324378	-1.026650	H	9.286705	2.264768	-1.632317
H	8.870697	3.116681	-0.906882	H	9.968575	2.566008	0.096229	H	8.744177	3.333557	-0.360436
H	9.840986	2.475620	0.436251	C	6.250371	2.731470	-1.265783	H	10.098532	2.264410	-0.060503
C	6.409748	2.627592	-1.262109	H	5.192594	2.668288	-1.491221	C	6.386659	2.506850	-1.393992
H	5.369303	2.538785	-1.549877	H	6.451032	3.677009	-0.755001	H	5.355409	2.606379	-1.677413
H	6.539896	3.578931	-0.746499	H	6.812435	2.766165	-2.202547	H	7.100251	3.191739	-1.815741
H	7.018640	2.662505	-2.163346	C	8.070550	-1.780974	1.904324	C	7.940779	-1.798307	2.132283
C	7.932855	-1.901827	2.056297	H	7.740347	-1.742062	2.943560	H	7.592303	-1.677947	3.162762
H	7.561522	-1.829142	3.081652	H	7.686794	-2.708435	1.480483	H	7.545995	-2.752804	1.778018
H	7.561959	-2.843121	1.646748	H	9.153633	-1.813568	1.895592	H	9.021898	-1.877706	2.163297
H	9.017282	-1.927405	2.088542	O	9.810473	0.233641	1.001191	O	9.788849	0.061351	1.141915
O	9.789179	0.012045	1.107569	H	10.252577	0.756202	0.319100	H	10.329544	0.589198	0.556797
H	5.275581	-1.711960	2.379082	H	5.372551	-1.746834	2.324131	H	5.283125	-1.571222	2.449615
H	3.009522	-1.776301	1.156742	H	3.088262	-1.853881	1.165810	H	3.006265	-1.712313	1.254272
C	4.003876	-1.244495	-1.419848	C	4.012955	-1.320200	-1.433612	C	3.979423	-1.355783	-1.358469
H	3.459168	-2.182955	-1.337079	H	3.493444	-2.271379	-1.335307	H	3.434496	-2.286108	-1.207271
H	3.772744	-0.781332	-2.376999	H	3.754758	-0.868828	-2.389239	H	3.740122	-0.954712	-2.340752
H	5.068871	-1.453590	-1.387917	H	5.083466	-1.501412	-1.417038	H	5.044968	-1.561559	-1.325486
H	-1.463450	-1.348005	0.179426	H	-1.467876	-1.421984	0.351848	H	-1.472173	-1.346547	0.218903
C	-1.440002	-1.880186	-1.893090	C	-1.406402	-2.176695	-1.650559	C	-1.466020	-1.936520	-1.837988
H	-2.301489	-2.540362	-1.844768	H	-2.290849	-2.804586	-1.586831	H	-2.321896	-2.600606	-1.754572
H	-1.441758	-1.388985	-2.866570	H	-1.334423	-1.792838	-2.668403	H	-1.489513	-1.473197	-2.824776
H	-0.549493	-2.497778	-1.830449	H	-0.541073	-2.806314	-1.467706	H	-0.570099	-2.546063	-1.775077
H	-13.111365	1.586716	2.163542	H	-13.067657	2.153158	1.716164	H	-13.117860	1.618640	2.153636
C	-0.229661	0.067423	-0.836347	C	-0.237056	-0.109614	-0.797560	C	-0.235760	0.032042	-0.844390
H	-0.209571	0.585116	-1.798322	H	-0.239191	0.339752	-1.793622	H	-0.209752	0.509278	-1.826838
H	-0.329888	0.840372	-0.072950	H	-0.334166	0.713643	-0.088514	H	-0.332684	0.836803	-0.114265
**α-Tocopherol (C3$^{\bullet}$)**				**α-Tocopherol (C4$^{\bullet}$)**				**α-Tocopherol (C5$^{\bullet}$)**			
C	3.628691	-0.329654	-0.238213	C	3.629126	-0.277321	-0.284875	C	-3.257147	-0.999617	-0.380984
C	5.845355	0.555289	0.082488	C	5.814696	0.598502	0.147562	C	-7.123328	-0.683453	0.085482
C	6.173553	-0.508382	0.909356	C	6.145446	-0.580316	0.892156	C	-6.513557	0.295711	-0.762421
C	5.131186	-1.448860	1.422664	C	5.151849	-1.457537	1.291947	C	-5.338584	-0.180087	-1.584242
C	3.716578	-0.957150	1.146533	C	3.720200	-1.183332	0.943536	C	-4.033393	-0.026272	-0.851373
C	6.778984	1.456236	-0.420854	C	6.747593	1.495943	-0.311507	C	-8.241018	-0.236945	0.862465
C	7.537905	-0.781881	1.259645	C	7.541761	-0.778935	1.142518	C	-6.971159	1.607133	-0.822355
H	5.283092	-2.443062	0.984128	H	5.394161	-2.357163	1.830053	H	-5.507321	-1.224635	-1.824893
H	3.443421	-0.184203	1.865240	H	3.153401	-0.686132	1.745133	H	-3.721922	0.995922	-0.665507
C	8.473265	0.177705	0.756093	C	8.456985	0.145500	0.688675	C	-8.061886	1.988768	-0.044971
C	8.124622	1.244509	-0.030807	C	8.117600	1.293691	-0.032145	C	-8.687348	1.076664	0.803404

O	4.515107	0.788178	-0.283317	O	4.463172	0.873437	-0.073135	O	-6.694132	-1.884905	0.138845
C	2.258904	0.295130	-0.492192	C	2.238676	0.329671	-0.446097	C	-1.974138	-0.678628	0.339901
H	2.326239	0.857428	-1.424598	H	2.247283	0.966247	-1.333005	H	-2.013359	-1.100128	1.348223
H	2.078266	1.020096	0.301767	H	2.073485	0.981815	0.411663	H	-1.868975	0.400681	0.445087
C	1.091623	-0.682181	-0.572646	C	1.095196	-0.673452	-0.545006	C	-0.738677	-1.241003	-0.366750
H	1.275077	-1.399029	-1.369478	H	1.289159	-1.370344	-1.357493	H	-0.866974	-2.313548	-0.495257
H	1.013841	-1.249318	0.355046	H	1.043263	-1.259089	0.372780	H	-0.676586	-0.807595	-1.366266
C	-1.471901	-0.846381	-0.757662	C	-1.472978	-0.874125	-0.667980	C	1.835366	-1.348294	-0.323580
C	-2.733085	0.016543	-0.835318	C	-2.742851	-0.028090	-0.781216	C	3.047423	-0.835400	0.456396
H	-2.754685	0.521237	-1.804263	H	-2.770421	0.431249	-1.772476	H	3.054080	-1.304592	1.443310
H	-2.662693	0.800848	-0.080293	H	-2.677727	0.791094	-0.063635	H	2.921896	0.235181	0.625097
C	-4.040328	-0.743282	-0.632254	C	-4.045308	-0.785818	-0.542132	C	4.393792	-1.070232	-0.222117
H	-3.985639	-1.308270	0.300844	H	-3.989515	-1.303494	0.417850	H	4.352902	-0.687062	-1.243978
H	-4.167629	-1.467654	-1.432059	H	-4.166967	-1.549489	-1.305524	H	4.584006	-2.137853	-0.293619
C	-5.248163	0.187442	-0.585931	C	-5.256549	0.141749	-0.545006	C	5.538468	-0.390501	0.522719
H	-5.054246	0.974915	0.143784	H	-5.071954	0.956601	0.156504	H	5.293432	0.665472	0.644823
H	-5.359863	0.683368	-1.553274	H	-5.357019	0.600811	-1.531687	H	5.613711	-0.807785	1.529876
C	-6.570484	-0.493520	-0.226309	C	-6.583535	-0.525706	-0.177143	C	6.904387	-0.498769	-0.158549
H	-6.439912	-0.989015	0.739100	H	-6.463171	-0.994159	0.802975	H	6.797611	-0.134832	-1.183661
C	-7.667421	0.561372	-0.068739	C	-7.677295	0.537821	-0.059858	C	7.910769	0.405370	0.555860
H	-7.312707	1.330108	0.619231	H	-7.330609	1.317674	0.619576	H	7.497797	1.414004	0.600493
H	-7.820714	1.054893	-1.031457	H	-7.809324	1.013644	-1.034636	H	8.014812	0.068009	1.589938
C	-9.002431	0.027547	0.441937	C	-9.025549	0.022847	0.434819	C	9.288884	0.470227	-0.095523
H	-8.836537	-0.534528	1.363473	H	-8.881517	-0.524545	1.368809	H	9.173368	0.687291	-1.159752
H	-9.418918	-0.668692	-0.281425	H	-9.433955	-0.682495	-0.284354	H	9.774982	-0.499304	-0.022515
C	-10.004186	1.145796	0.708959	C	-10.023220	1.153478	0.662847	C	10.175650	1.535824	0.540304
H	-9.541962	1.881843	1.368396	H	-9.568596	1.896389	1.319868	H	9.639439	2.485825	0.535696
H	-10.225581	1.664835	-0.226863	H	-10.220575	1.658973	-0.285692	H	10.349793	1.284297	1.589499
C	-11.320251	0.686806	1.336923	C	-11.356182	0.715887	1.269840	C	11.526904	1.734556	-0.146500
H	-11.084864	0.169343	2.268715	H	-11.145067	0.212372	2.214956	H	11.337781	1.971699	-1.195058
C	-12.193794	1.894761	1.663370	C	-12.226121	1.936418	1.556791	C	12.268719	2.907708	0.487446
H	-11.675016	2.589141	2.320233	H	-11.715825	2.637409	2.213287	H	11.678057	3.819597	0.438182
H	-12.452007	2.426645	0.748321	H	-12.459110	2.455133	0.627522	H	12.473385	2.695948	1.536493
C	-6.954937	-1.548345	-1.261485	C	-6.963232	-1.607626	-1.185628	C	7.391288	-1.945098	-0.208788
H	-7.086816	-1.079621	-2.237145	H	-7.098332	-1.163475	-2.172288	H	7.537199	-2.322595	0.803622
H	-6.188116	-2.311073	-1.355341	H	-6.192086	-2.367892	-1.261274	H	6.672962	-2.588201	-0.707653
H	-7.882343	-2.048305	-0.998328	H	-7.887793	-2.105986	-0.909473	H	8.333909	-2.031457	-0.741494
C	-12.080847	-0.276470	0.429339	C	-12.105353	-0.256496	0.362449	C	12.390829	0.477649	-0.084895
H	-12.262710	0.190482	-0.538656	H	-12.260521	0.194404	-0.617768	H	12.541044	0.179593	0.952623
H	-11.532229	-1.198508	0.262348	H	-11.561987	-1.186426	0.224643	H	11.937222	-0.355119	-0.613843
H	-13.044407	-0.533797	0.863544	H	-13.081057	-0.496869	0.778977	H	13.368338	0.661731	-0.525246
C	9.210972	2.191883	-0.484958	C	9.190164	2.254996	-0.471362	C	-9.849441	1.513843	1.659739
H	9.918409	1.708601	-1.164083	H	9.902551	1.812360	-1.177521	H	-10.791513	1.111090	1.283858
H	8.796494	3.044057	-1.010109	H	8.759029	3.115980	-0.969915	H	-9.733240	1.147800	2.677487
H	9.778278	2.580391	0.362983	H	9.773599	2.649104	0.368759	H	-9.924029	2.595179	1.684687
C	6.371989	2.595701	-1.316024	C	6.296571	2.702138	-1.097214	C	-8.910025	-1.245528	1.754530
H	5.319011	2.511031	-1.558114	H	5.229870	2.652442	-1.275619	H	-8.547442	-2.230214	1.479095
H	6.522165	3.574286	-0.850629	H	6.509654	3.627846	-0.561846	H	-8.671064	-1.081954	2.808233
H	6.927993	2.602507	-2.256092	H	6.807327	2.756617	-2.058134	H	-9.995896	-1.215562	1.661408
C	7.937450	-1.868363	2.018562	C	7.956943	-1.996166	1.904935	C	-6.307127	2.618890	-1.725895
H	7.228463	-2.591160	2.381308	H	7.472641	-2.018698	2.885083	H	-5.224402	2.558685	-1.652336
H	8.976454	-2.008891	2.253525	H	7.633528	-2.902645	1.384812	H	-6.568160	2.446784	-2.771305
O	9.803073	-0.040120	1.112960	H	9.031614	-2.032438	2.041855	H	-6.616692	3.622849	-1.459563
H	10.344656	0.577798	0.624984	O	9.802447	-0.093551	0.973429	O	-8.525580	3.324492	-0.100237
H	5.272561	-1.587814	2.497197	H	10.305264	0.627053	0.596866	H	-9.231149	3.361416	-0.745328
H	3.001936	-1.773600	1.238930	H	3.188693	-2.113323	0.726183	H	-5.280774	0.379275	-2.518353
C	4.002834	-1.320661	-1.337316	C	4.077827	-0.994577	-1.551878	C	-3.594923	-2.463629	-0.505448
H	3.432799	-2.242433	-1.241563	H	3.450074	-1.861360	-1.747789	H	-3.198800	-2.889939	-1.428505
H	3.807202	-0.878648	-2.312571	H	4.022249	-0.311843	-2.398440	H	-3.148685	-3.015897	0.321963
H	5.060156	-1.557262	-1.267998	H	5.101811	-1.333529	-1.432662	H	-4.672415	-2.602286	-0.466201
H	-1.472240	-1.354177	0.210189	H	-1.465761	-1.340599	0.320391	H	1.834839	-0.860327	-1.301632
C	-1.452276	-1.906264	-1.857134	C	-1.440178	-1.979454	-1.720859	C	1.906719	-2.858205	-0.539407
H	-2.309713	-2.570422	-1.792953	H	-2.295647	-2.644119	-1.636219	H	2.791440	-3.143784	-1.101939
H	-1.467348	-1.424526	-2.835303	H	-1.446898	-1.540632	-2.719200	H	1.935747	-3.368997	0.423589
H	-0.557997	-2.518559	-1.798288	H	-0.543299	-2.583278	-1.625748	H	1.039527	-3.217655	-1.084138
H	-13.119400	1.591451	2.147378	H	-13.164950	1.649348	2.025006	H	13.219091	3.087814	-0.009926
C	-0.228613	0.043140	-0.819159	C	-0.241731	0.028211	-0.768384	C	0.547087	-0.949525	0.399518
H	-0.195264	0.536347	-1.793698	H	-0.238285	0.510441	-1.749256	H	0.514795	-1.458063	1.366379
H	-0.331551	0.835679	-0.076432	H	-0.338927	0.828520	-0.032956	H	0.586734	0.119597	0.615651
**α-Tocopherol (C6$^{\bullet}$)**				**α-Tocopherol (C7$^{\bullet}$)**				**α-Tocopherol (C8$^{\bullet}$)**			
C	3.601678	-0.388362	-0.305484	C	-3.546974	-0.084731	-0.476238	C	3.522307	-0.838182	0.429558
C	5.814974	0.530362	0.032688	C	-5.773173	0.000223	0.435490	C	5.721530	-0.103786	-0.209151
C	6.219522	-0.578023	0.780676	C	-6.136022	1.038020	-0.409396	C	5.453198	1.194991	0.210410
C	5.199658	-1.599288	1.206368	C	-5.097608	1.732442	-1.250852	C	4.052570	1.601925	0.590977
C	3.790487	-1.044851	1.078954	C	-3.697981	1.415193	-0.748892	C	3.030102	0.569812	0.137860

C	6.751204	1.482424	-0.393052	C	-6.704198	-0.635247	1.234278	C	7.007553	-0.498075	-0.585541
C	7.567653	-0.743780	1.101782	C	-7.464496	1.441386	-0.470031	C	6.491649	2.125648	0.282213
H	5.286168	-2.474416	0.560777	H	-5.200500	1.426192	-2.292333	H	3.993394	1.737292	1.671889
H	3.614961	-0.304121	1.865576	H	-3.513448	1.931957	0.190932	H	2.872265	0.648115	-0.937108
C	8.478010	0.221782	0.705434	C	-8.378410	0.817484	0.331275	C	7.764809	1.733211	-0.097453
C	8.086990	1.339831	-0.025059	C	-8.026585	-0.224396	1.174441	C	8.036162	0.438726	-0.536878
O	4.524809	0.767297	-0.284370	O	-4.481097	-0.444198	0.558650	O	4.737557	-1.060754	-0.304399
C	2.235857	0.295741	-0.359489	C	-2.198069	-0.358458	0.092272	C	2.556913	-1.889253	-0.134692
H	2.276683	0.980822	-1.204490	H	-2.054071	-0.081358	1.125624	H	3.036111	-2.859468	0.016778
H	2.080966	0.880809	0.552174	C	-1.017549	-0.662599	-0.756466	H	2.494124	-1.725210	-1.209433
C	1.076295	-0.667222	-0.566553	H	-1.220820	-1.536989	-1.375657	C	1.197165	-1.869849	0.464952
H	1.293731	-1.237624	-1.465354	H	-0.836697	0.157831	-1.464770	H	1.077221	-2.131813	1.504825
H	1.007780	-1.368551	0.268159	C	1.502741	-1.144211	-0.771229	C	-1.330648	-1.806100	0.334796
C	-1.488397	-0.832515	-0.723247	C	2.759402	-1.076539	0.102145	C	-2.479569	-1.101789	-0.386475
C	-2.756922	0.021705	-0.769700	H	2.844320	-2.005107	0.671018	H	-2.473113	-1.407092	-1.435833
H	-2.785512	0.552819	-1.724289	H	2.650387	-0.275015	0.833300	H	-2.292882	-0.027006	-0.375721
H	-2.689249	0.785838	0.006561	C	4.047424	-0.844039	-0.681942	C	-3.859922	-1.366882	0.205269
C	-4.059934	-0.749487	-0.582105	H	3.975841	0.111366	-1.204801	H	-3.845270	-1.137983	1.273335
H	-4.011927	-1.312544	0.352617	H	4.158457	-1.611344	-1.444653	H	-4.098218	-2.423086	0.112221
H	-4.171015	-1.476455	-1.382156	C	5.275106	-0.830935	0.223149	C	-4.941943	-0.533989	-0.474515
C	-5.274713	0.173130	-0.552060	H	5.096730	-0.133472	1.042613	H	-4.628338	0.510953	-0.478514
H	-5.087631	0.971754	0.167488	H	5.397161	-1.817343	0.676863	H	-5.024747	-0.835818	-1.521439
H	-5.385128	0.655774	-1.526316	C	6.579718	-0.443667	-0.475392	C	-6.325194	-0.623254	0.173289
C	-6.596023	-0.507556	-0.188467	H	6.421084	0.513307	-0.978751	H	-6.234617	-0.278416	1.206419
H	-6.470242	-0.982892	0.787770	C	7.698213	-0.259538	0.553448	C	-7.293947	0.312892	-0.552652
C	-7.698652	0.545535	-0.061015	H	7.302918	0.269547	1.421175	H	-6.810750	1.281933	-0.685589
H	-7.352848	1.328790	0.615060	H	8.012738	-1.243633	0.908656	H	-7.477258	-0.080919	-1.555264
H	-7.844153	1.020009	-1.034519	C	8.915588	0.499646	0.034186	C	-8.626179	0.532291	0.157213
C	-9.037507	0.018530	0.446563	H	8.592762	1.463014	-0.365376	H	-8.437305	0.905351	1.165929
H	-8.880281	-0.522799	1.381960	H	9.366494	-0.047726	-0.790066	H	-9.149504	-0.414366	0.264478
H	-9.443790	-0.694687	-0.266038	C	9.954235	0.728369	1.127258	C	-9.514129	1.523395	-0.588289
C	-10.045216	1.139675	0.677246	H	9.457362	1.165614	1.994771	H	-8.935879	2.423767	-0.801432
H	-9.592314	1.891231	1.325600	H	10.355048	-0.233733	1.455036	H	-9.794701	1.100023	-1.555867
H	-10.256410	1.637525	-0.272390	C	11.117650	1.637993	0.731799	C	-10.786391	1.927371	0.156081
C	-11.368037	0.692271	1.299143	H	10.702371	2.608018	0.452330	H	-10.490757	2.367067	1.110459
H	-11.143257	0.196953	2.245488	C	12.045056	1.841896	1.926873	C	-11.547802	2.979332	-0.644956
C	-12.248102	1.905462	1.586473	H	11.526399	2.329738	2.749006	H	-10.924548	3.847111	-0.848580
H	-11.739183	2.615689	2.234080	H	12.411203	0.879981	2.283648	H	-11.866695	2.562749	-1.599764
H	-12.494561	2.416077	0.656187	C	6.973226	-1.478364	-1.528318	C	-6.842408	-2.059949	0.185445
C	-6.966896	-1.585176	-1.204706	H	7.102579	-2.452763	-1.057685	H	-6.877388	-2.449199	-0.832265
H	-7.095943	-1.136473	-2.190116	H	6.216234	-1.574249	-2.301218	H	-6.201741	-2.710266	0.773601
H	-6.192082	-2.341819	-1.278827	H	7.906797	-1.213540	-2.016471	H	-7.842045	-2.121105	0.604744
H	-7.891921	-2.087947	-0.937551	C	11.906772	1.089584	-0.453377	C	-11.688939	0.728974	0.437108
C	-12.115174	-0.293637	0.404815	H	12.365667	0.137422	-0.187426	H	-11.940445	0.224490	-0.495614
H	-12.283656	0.149275	-0.576843	H	11.278396	0.930973	-1.324590	H	-11.213671	0.007126	1.094984
H	-11.563049	-1.218503	0.267957	H	12.701521	1.776227	-0.735135	H	-12.616924	1.048840	0.905413
H	-13.084588	-0.541577	0.831627	C	-9.097384	-0.882269	2.003483	C	9.446096	0.098304	-0.943679
C	9.128815	2.363812	-0.400465	H	-9.978983	-1.102592	1.402442	H	10.136113	0.192743	-0.103840
H	9.814383	1.990164	-1.164895	H	-8.755645	-1.821394	2.418412	H	9.523282	-0.916492	-1.309635
H	8.671239	3.263743	-0.791026	H	-9.404650	-0.249872	2.837515	H	9.793731	0.755952	-1.741027
H	9.720821	2.654122	0.468215	C	-6.272975	-1.753386	2.144743	C	7.257662	-1.913193	-1.038840
C	6.280305	2.623895	-1.255597	H	-5.199014	-1.879217	2.107928	H	6.352782	-2.499225	-0.956997
H	5.309191	2.378205	-1.666802	H	-6.561142	-1.549653	3.173255	H	7.586554	-1.939386	-2.075849
H	6.184619	3.549108	-0.685807	H	-6.735603	-2.694113	1.851971	H	8.028888	-2.385388	-0.434755
H	6.969245	2.804507	-2.076725	C	-7.900303	2.551863	-1.383489	C	6.232990	3.530432	0.752829
C	8.026445	-1.957239	1.864884	H	-7.654040	3.525216	-0.960570	H	5.682901	4.097802	0.003255
H	7.714126	-1.907109	2.907815	H	-7.407341	2.477409	-2.348247	H	5.639239	3.531316	1.663401
H	7.592295	-2.859039	1.440584	H	-8.972104	2.518050	-1.537692	H	7.164483	4.045821	0.946484
H	9.106237	-2.039408	1.838441	O	-9.671697	1.265267	0.263635	O	8.765481	2.676096	-0.032897
O	9.811089	0.049078	1.065544	H	-10.187137	0.851379	0.960010	H	9.599555	2.271027	-0.269695
H	10.340651	0.653656	0.547254	H	-5.263045	2.806725	-1.231230	H	3.821115	2.568997	0.151418
H	5.397257	-1.919621	2.228680	H	-2.943528	1.735251	-1.463625	H	2.074308	0.730136	0.630670
H	3.064626	-1.846687	1.191460	C	-3.832087	-0.926051	-1.715165	C	3.786590	-1.068424	1.910899
C	3.865631	-1.228235	-1.519752	H	-3.151949	-0.660886	-2.520776	H	2.902104	-0.827336	2.495125
H	3.252459	-2.133176	-1.495865	H	-3.704650	-1.977294	-1.474110	H	4.047933	-2.110144	2.077479
H	4.915195	-1.525213	-1.564737	H	-4.851994	-0.772894	-2.053105	H	4.610817	-0.451091	2.255889
H	-1.499366	-1.400703	0.211050	H	1.574288	-0.356541	-1.525829	H	-1.323989	-1.467319	1.373670
C	-1.435721	-1.820536	-1.886422	C	1.417946	-2.490865	-1.488887	C	-1.494343	-3.323863	0.317555
H	-2.297241	-2.483948	-1.894424	H	2.318726	-2.693005	-2.061587	H	-2.364690	-3.643029	0.882991
H	-1.412437	-1.276771	-2.831087	H	1.296909	-3.289606	-0.758052	H	-1.609079	-3.671649	-0.709118
H	-0.541630	-2.433333	-1.836204	H	0.577975	-2.532402	-2.176331	H	-0.619851	-3.811852	0.740153
H	-13.179832	1.611665	2.064751	H	12.906320	2.449078	1.657570	H	-12.435221	3.312743	-0.112159
C	-0.252267	0.069306	-0.706857	C	0.249924	-0.882795	0.065645	C	0.005730	-1.408942	-0.295918
H	-0.235226	0.660336	-1.626222	H	0.090935	-1.720794	0.746690	H	0.047316	-1.802850	-1.316654
H	-0.355132	0.780209	0.115974	H	0.415959	-0.002353	0.686181	H	0.031430	-0.317236	-0.409596

α-Tocopherol (C9$^{\bullet}$)				α-Tocopherol (C10$^{\bullet}$)				α-Tocopherol (C11$^{\bullet}$)			
C	-3.535137	-0.430564	-0.303462	C	3.527535	-0.532961	-0.310659	C	3.497758	-0.179199	0.388799
C	-5.779707	0.040593	0.469045	C	5.621709	0.635316	-0.034777	C	5.799547	-0.388200	-0.312594
C	-5.993199	0.954142	-0.562573	C	6.134249	-0.383384	0.761335	C	5.962584	0.996756	-0.289971
C	-4.841728	1.393780	-1.429153	C	5.233484	-1.485638	1.260238	C	4.767660	1.905074	-0.135983
C	-3.506804	1.001952	-0.812476	C	3.766182	-1.129125	1.067313	C	3.462214	1.155189	-0.338302
C	-6.827858	-0.350425	1.311547	C	6.411637	1.688768	-0.475651	C	6.886222	-1.247286	-0.494469
C	-7.275018	1.462923	-0.784112	C	7.479120	-0.374533	1.100753	C	7.243490	1.544740	-0.412199
H	-4.940073	0.950639	-2.421733	H	5.459692	-2.414533	0.734242	H	4.776507	2.357742	0.855147
H	-3.289216	1.642609	0.040744	H	3.473915	-0.378468	1.799215	H	3.308022	0.944657	-1.395227
C	-8.304944	1.072236	0.054057	C	8.270173	0.649938	0.642446	C	8.316283	0.685586	-0.579691
C	-8.097527	0.181549	1.104500	C	7.752920	1.702031	-0.121979	C	8.157796	-0.696227	-0.629003
O	-4.558181	-0.499535	0.732662	O	4.301298	0.678546	-0.411490	O	4.568731	-0.977664	-0.173016
C	-2.240656	-0.815495	0.397715	C	2.093265	-0.050960	-0.480592	C	2.237936	-0.972085	0.121478
H	-2.423230	-1.720945	0.973868	H	2.075597	0.587307	-1.364109	H	2.382819	-1.972130	0.529866
H	-1.995359	-0.030466	1.114000	H	1.844924	0.580643	0.370673	H	2.122497	-1.075875	-0.958423
C	-1.035541	-1.059371	-0.511008	C	1.044017	-1.146273	-0.633867	C	0.998960	-0.335765	0.728651
H	-1.233625	-1.958205	-1.098935	H	1.330335	-1.806028	-1.450193	H	1.152046	-0.117307	1.787811
H	-0.979575	-0.235205	-1.265618	H	0.987882	-1.749547	0.275192	H	0.780071	0.612825	0.246734
C	1.457628	-1.295583	-0.590805	C	-1.535186	-1.337433	-0.441710	C	-1.483835	-0.624749	1.128858
C	2.722822	-1.038087	0.230132	C	-2.805763	-0.784473	-1.088649	C	-2.820005	-1.247834	0.823096
H	2.768583	-1.798926	1.012090	H	-2.764280	-0.987822	-2.164275	H	-3.116762	-1.834914	1.691174
H	2.604689	-0.078793	0.738301	H	-2.826296	0.298322	-0.979590	H	-2.746142	-1.935158	-0.018899
C	4.034757	-1.021414	-0.551088	C	-4.093536	-1.353084	-0.504127	C	-3.911076	-0.209604	0.527876
H	3.961405	-0.305732	-1.373533	H	-4.026301	-1.335661	0.588433	H	-3.616427	0.368018	-0.349859
H	4.201282	-1.998711	-0.999373	H	-4.202857	-2.397432	-0.792840	H	-3.949325	0.492325	1.357923
C	5.221868	-0.649682	0.333272	C	-5.317604	-0.550909	-0.938090	C	-5.282646	-0.841414	0.317831
H	4.976014	0.266368	0.873068	H	-5.080840	0.511987	-0.862426	H	-5.211242	-1.653648	-0.410306
H	5.362428	-1.423698	1.092444	H	-5.533101	-0.749763	-1.989565	H	-5.617328	-1.300845	1.248747
C	6.546297	-0.436175	-0.403062	C	-6.573283	-0.824365	-0.110792	C	-6.354877	0.151185	-0.143967
H	6.388340	0.341372	-1.155357	H	-6.343727	-0.546362	0.920219	H	-6.103228	0.478141	-1.157355
C	7.609719	0.065949	0.575718	C	-7.724257	0.063808	-0.585097	C	-7.722477	-0.528835	-0.189014
H	7.197360	0.911400	1.128432	H	-7.322435	1.058778	-0.785126	H	-7.670396	-1.415821	-0.819802
H	7.806128	-0.717396	1.312031	H	-8.104353	-0.316368	-1.535612	H	-7.970101	-0.884072	0.814190
C	8.925225	0.499182	-0.064200	C	-8.866400	0.213996	0.415237	C	-8.872388	0.342484	-0.680345
H	8.718754	1.231853	-0.847626	H	-8.438407	0.498608	1.379183	H	-8.634193	0.739624	-1.672988
H	9.398815	-0.352307	-0.546660	H	-9.379936	-0.731534	0.565359	H	-9.006792	1.197248	-0.022344
C	9.884254	1.109035	0.953682	C	-9.851621	1.298642	-0.017521	C	-10.179125	-0.440790	-0.725363
H	9.353956	1.878849	1.516364	H	-9.275825	2.152243	-0.377077	H	-10.049267	-1.296595	-1.391852
H	10.178358	0.344943	1.677747	H	-10.449157	0.950303	-0.863866	H	-10.386981	-0.855304	0.261253
C	11.146990	1.728039	0.355482	C	-10.778897	1.797193	1.089487	C	-11.395330	0.375142	-1.172563
H	10.837428	2.496375	-0.355477	H	-10.146751	2.101906	1.926121	H	-11.182411	0.813319	-2.147868
C	11.976928	2.390452	1.451623	C	-11.567110	3.016549	0.619869	C	-12.606407	-0.536346	-1.304864
H	11.395994	3.138252	1.986659	H	-10.906257	3.804225	0.262605	H	-12.434350	-1.303168	-2.059203
H	12.306570	1.642653	2.172359	H	-12.235092	2.739223	-0.195490	H	-12.789016	-1.034657	-0.354842
C	6.999417	-1.708069	-1.115911	C	-6.942380	-2.302105	-0.136399	C	-6.395708	1.378814	0.759457
H	7.154573	-2.504759	-0.387525	H	-7.051161	-2.639553	-1.166181	H	-6.509601	1.078807	1.800378
H	6.249994	-2.044690	-1.825117	H	-6.174610	-2.911632	0.335593	H	-5.476444	1.950009	0.683989
H	7.928849	-1.558541	-1.659585	H	-7.878297	-2.489398	0.381624	H	-7.216376	2.045272	0.508319
C	11.991882	0.696975	-0.387837	C	-11.739255	0.717543	1.582126	C	-11.701094	1.490476	-0.177235
H	12.249912	-0.124066	0.281105	H	-12.329940	0.338608	0.748066	H	-11.864274	1.068439	0.814514
H	11.465611	0.283021	-1.242608	H	-11.213054	-0.117649	2.035415	H	-10.888014	2.203989	-0.116429
H	12.917890	1.142474	-0.745595	H	-12.426011	1.119428	2.323729	H	-12.596436	2.034508	-0.467006
C	-9.265827	-0.181123	1.985391	C	8.671135	2.826491	-0.524343	C	9.375873	-1.551597	-0.858523
H	-10.041538	-0.708775	1.426617	H	9.394974	2.506844	-1.275525	H	10.085561	-1.472089	-0.033349
H	-8.962789	-0.826815	2.798746	H	8.121663	3.658816	-0.940981	H	9.112386	-2.596050	-0.956970
H	-9.716346	0.708324	2.427462	H	9.221622	3.207615	0.336939	H	9.890615	-1.262114	-1.774718
C	-6.558926	-1.333005	2.422126	C	5.808128	2.787240	-1.310561	C	6.671998	-2.739591	-0.547313
H	-5.534667	-1.676558	2.373438	H	4.772993	2.567841	-1.529541	H	5.634838	-2.985847	-0.362364
H	-6.717613	-0.876275	3.397792	H	5.845668	3.742494	-0.789380	H	6.944023	-3.133933	-1.525281
H	-7.216636	-2.196774	2.348863	H	6.338850	2.901223	-2.252229	H	7.280467	-3.247900	0.196596
C	-7.531214	2.429021	-1.908604	C	8.079467	-1.474921	1.926653	C	7.458117	3.034166	-0.362917
H	-7.125143	3.413343	-1.677813	H	7.758332	-1.400836	2.965372	H	7.109326	3.513285	-1.275801
H	-7.054831	2.088801	-2.824355	H	7.777021	-2.451518	1.559220	H	6.909915	3.479460	0.465032
H	-8.593942	2.536444	-2.085916	H	9.161188	-1.415880	1.906773	H	8.508825	3.261679	-0.246182
O	-9.561376	1.606614	-0.175083	O	9.598682	0.600144	0.980199	O	9.569651	1.242167	-0.705293
H	-10.188155	1.173678	0.403518	H	10.062973	1.256898	0.502608	H	10.227465	0.546209	-0.707877
H	-4.884950	2.471868	-1.572062	H	5.431557	-1.673833	2.312390	H	4.839450	2.727644	-0.842869
H	-2.698391	1.112976	-1.527749	H	3.136911	-2.005337	1.211261	H	2.621434	1.751038	0.010719
C	-3.897496	-1.430947	-1.391815	C	3.933617	-1.477766	-1.437050	C	3.705573	-0.005716	1.885888
H	-3.216142	-1.320423	-2.231275	H	3.442002	-2.441259	-1.319906	H	2.939309	0.652748	2.295386
H	-3.798315	-2.438736	-0.997576	H	3.640919	-1.053343	-2.393214	H	3.638629	-0.965363	2.391656
H	-4.918549	-1.289662	-1.737975	H	5.006837	-1.633853	-1.449545	H	4.681657	0.426110	2.073482
H	1.446143	-0.524187	-1.402163	H	-1.596311	-1.158140	0.629078	C	-1.407747	0.304946	2.298381
C	1.556154	-2.649553	-1.305244	C	-1.402115	-2.799861	-0.645847	H	-2.323669	0.873943	2.419957

H	2.339413	-2.666688	-2.067065	H	-1.935809	-3.278092	-1.452569	H	-1.252127	-0.286480	3.204864
H	1.749996	-3.427020	-0.565693	H	-0.758296	-3.380119	-0.006173	H	-0.585216	1.012996	2.229704
H	0.614826	-2.887219	-1.794121	H	-12.177181	3.424733	1.423110	H	-13.497802	0.021085	-1.577279
H	12.861174	2.872507	1.040154	C	-0.317336	-0.531732	-0.938120	C	-0.230291	-1.232173	0.571974
C	0.236909	-1.205993	0.287896	H	-0.408420	-0.372759	-2.014994	H	-0.065318	-2.184358	1.080634
H	0.335476	-0.335851	0.947052	H	-0.372357	0.451188	-0.469437	H	-0.367687	-1.476400	-0.484360
**α-Tocopherol (C12•)**				**α-Tocopherol (C13•)**				**α-Tocopherol (C14•)**			
C	3.568929	-0.387927	-0.357880	C	-3.537986	-0.375341	-0.339353	C	-3.592362	-0.357820	0.275176
C	5.756162	0.574087	0.001066	C	-5.770193	0.055321	0.472776	C	-5.783683	0.547559	-0.135056
C	6.162820	-0.522692	0.757097	C	-6.026627	0.939403	-0.572052	C	-6.300784	-0.707909	-0.435928
C	5.158883	-1.562125	1.183389	C	-4.908595	1.387917	-1.477017	C	-5.379954	-1.890858	-0.587804
C	3.731403	-1.069594	0.991401	C	-3.546978	1.041215	-0.892583	C	-3.924998	-1.461363	-0.718886
C	6.663882	1.568956	-0.377074	C	-6.786283	-0.351533	1.343327	C	-6.602269	1.654896	0.007538
C	7.505189	-0.654624	1.119199	C	-7.325543	1.409018	-0.778177	C	-7.678863	-0.873429	-0.574614
H	5.316449	-2.478123	0.611955	H	-5.023853	0.923339	-2.457516	H	-5.492919	-2.543541	0.278957
H	3.480124	-0.340483	1.760300	H	-3.312331	1.714686	-0.069783	H	-3.734683	-1.071292	-1.717547
C	8.398292	0.334346	0.743842	C	-8.326674	1.006072	0.089250	C	-8.492632	0.224639	-0.428054
C	7.995549	1.448112	0.010144	C	-8.074484	0.140137	1.151086	C	-7.978013	1.491067	-0.130137
O	4.459278	0.755982	-0.389424	O	-4.520319	-0.442707	0.722527	O	-4.436208	0.773643	-0.013201
C	2.181002	0.213580	-0.526549	C	-2.221235	-0.705405	0.350005	C	-2.186614	0.190271	0.059964
H	2.191561	0.810490	-1.438977	H	-2.361429	-1.643326	0.887879	H	-2.074360	1.062975	0.703818
H	2.015294	0.905205	0.299562	H	-2.035538	0.063516	1.100097	H	-2.128537	0.549928	-0.966897
C	1.030453	-0.786921	-0.594919	C	-1.010127	-0.828847	-0.570238	C	-1.045957	-0.786556	0.328309
H	1.202465	-1.490652	-1.407432	H	-1.167822	-1.640087	-1.277620	H	-1.147915	-1.202694	1.326775
H	0.979454	-1.369245	0.325529	H	-0.882971	0.082087	-1.155310	H	-1.090293	-1.617093	-0.376433
C	-1.545617	-0.977992	-0.728726	C	1.534870	-1.175709	-0.648186	C	1.532097	-1.004821	0.271911
C	-2.792224	-0.168314	-0.968340	C	2.801285	-1.094982	0.211913	C	2.795703	-0.157912	0.083959
H	-2.709464	0.783663	-0.431349	H	2.698390	-1.900403	0.982874	H	2.721204	0.731941	0.710716
C	-4.043532	-0.875818	-0.511014	H	2.747907	-0.158252	0.776666	H	2.832703	0.191933	-0.947920
H	-4.006111	-1.195747	0.562521	C	4.103185	-1.132200	-0.553435	C	4.107265	-0.884680	0.385955
H	-4.185182	-1.803359	-1.069676	H	4.266543	-2.144190	-0.932169	H	4.042243	-1.906331	-0.013488
C	-5.291957	-0.013652	-0.684643	C	5.257594	-0.744131	0.341193	H	4.228427	-1.016276	1.461872
H	-5.071647	0.981550	-0.292430	H	4.995838	0.172848	0.879613	C	5.299186	-0.198440	-0.175939
H	-5.478229	0.115497	-1.752702	H	5.486721	-1.491396	1.142541	H	5.174656	0.441353	-1.037146
C	-6.563615	-0.532327	-0.006973	C	6.584815	-0.481088	-0.379397	C	6.649686	-0.319758	0.442660
H	-6.369235	-0.609240	1.066305	H	6.393637	0.315767	-1.101114	H	6.829213	-1.376685	0.669621
C	-7.703355	0.468082	-0.209183	C	7.659943	-0.009824	0.602509	C	7.732413	0.153209	-0.528470
H	-7.354002	1.453088	0.104864	H	7.240932	0.795490	1.210054	H	7.642458	-0.415293	-1.454437
H	-7.915362	0.544222	-1.278684	H	7.898525	-0.823945	1.291586	H	7.532706	1.196898	-0.781244
C	-8.995413	0.146027	0.536630	C	8.946850	0.490796	-0.048521	C	9.156949	0.027060	-0.000527
H	-8.772916	-0.002837	1.595813	H	8.698552	1.257218	-0.786158	H	9.330886	-1.000141	0.327482
H	-9.405886	-0.790687	0.166850	H	9.421642	-0.323830	-0.590908	H	9.279227	0.660508	0.874532
C	-10.033026	1.254297	0.390673	C	9.925295	1.071821	0.967897	C	10.191071	0.397887	-1.057579
H	-9.576206	2.200915	0.683677	H	9.398052	1.809902	1.574784	H	10.024038	-0.222571	-1.939555
H	-10.304609	1.357343	-0.663202	H	10.248023	0.283830	1.653738	H	10.033830	1.431921	-1.373969
C	-11.311138	1.056416	1.206314	C	11.164518	1.734352	0.366266	C	11.646455	0.232977	-0.619712
H	-11.027151	0.954161	2.255421	H	10.826219	2.514902	-0.317909	H	11.787321	-0.803116	-0.306473
C	-12.217110	2.276572	1.067759	C	12.002451	2.384502	1.464042	C	12.576658	0.504763	-1.799010
H	-11.703668	3.183802	1.378052	H	11.417664	3.109633	2.025705	H	12.381345	-0.179890	-2.621017
H	-12.517393	2.400454	0.027616	H	12.354444	1.625088	2.162002	H	12.429776	1.520723	-2.163961
C	-6.936669	-1.919890	-0.522805	C	7.022788	-1.722712	-1.147802	C	6.717055	0.446979	1.776585
H	-7.121573	-1.876130	-1.597069	H	7.152625	-2.557288	-0.454862	H	6.765916	1.517760	1.586211
H	-6.122763	-2.617529	-0.352368	H	6.249500	-1.985781	-1.863642	H	5.834916	0.253391	2.380339
H	-7.827004	-2.314370	-0.037104	H	7.957926	-1.574212	-1.686453	H	7.589063	0.156677	2.356556
C	-12.065465	-0.202624	0.787363	C	12.016525	0.742600	-0.420996	C	12.005717	1.148171	0.547201
H	-12.296609	-0.159457	-0.277106	H	12.309638	-0.086980	0.222792	H	11.861016	2.189766	0.260877
H	-11.484154	-1.101249	0.969420	H	11.476982	0.335061	-1.270253	H	11.397993	0.950919	1.425288
H	-13.003609	-0.290392	1.332062	H	12.923069	1.218289	-0.791002	H	13.048731	1.020693	0.827195
C	9.025649	2.488880	-0.345782	C	-9.215924	-0.238373	2.059465	C	-8.933756	2.649455	-0.003965
H	9.799515	2.079237	-0.997444	H	-9.991917	-0.784332	1.519685	H	-9.683952	2.460739	0.764482
H	8.580297	3.327223	-0.864431	H	-8.885315	-0.871803	2.871552	H	-8.422470	3.563498	0.265058
H	9.511643	2.883675	0.547387	H	-9.673889	0.645886	2.503914	H	-9.454486	2.834986	-0.944668
C	6.185467	2.745079	-1.188541	C	-6.470030	-1.303982	2.467345	C	-5.994298	3.001032	0.299762
H	5.141714	2.622451	-1.442422	H	-5.436943	-1.618156	2.411715	H	-4.914380	2.933157	0.306799
H	6.292221	3.674933	-0.632687	H	-6.630043	-0.833737	3.435937	H	-6.284694	3.730069	-0.453022
H	6.754687	2.841735	-2.110477	H	-7.101594	-2.188474	2.419460	H	-6.319529	3.375668	1.268314
C	7.972076	-1.844941	1.911488	C	-7.630264	2.345778	-1.914929	C	-8.276112	-2.217994	-0.884091
H	7.632517	-1.783479	2.944746	H	-7.242362	3.343386	-1.712552	H	-8.164431	-2.459460	-1.940564
H	7.574564	-2.766326	1.493732	H	-7.168755	1.998409	-2.835566	H	-7.787202	-3.002985	-0.314708
H	9.053160	-1.901165	1.914901	H	-8.698728	2.425284	-2.069339	H	-9.333834	-2.227394	-0.650251
O	9.717119	0.187776	1.129347	O	-9.597753	1.501563	-0.124466	O	-9.846293	0.018187	-0.555394
H	10.230476	0.903907	0.756733	H	-10.202337	1.078652	0.484703	H	-10.295469	0.854411	-0.530667
H	5.323774	-1.822479	2.226597	H	-4.978961	2.461090	-1.639766	H	-5.672318	-2.478485	-1.454230
H	3.029570	-1.893947	1.070351	H	-2.771163	1.148679	-1.644165	H	-3.270851	-2.314177	-0.558382
C	3.931512	-1.308749	-1.516494	C	-3.896613	-1.413037	-1.394907	C	-3.822896	-0.784584	1.718631

H	3.384751	-2.244473	-1.440258	H	-3.262985	-1.296376	-2.269908	H	-3.254738	-1.680944	1.952326
H	3.671138	-0.829495	-2.456248	H	-3.750032	-2.410499	-0.988941	H	-3.515206	0.014807	2.387610
H	4.996857	-1.524304	-1.521012	H	-4.934802	-1.313465	-1.699322	H	-4.874575	-0.990045	1.894705
H	-1.530463	-1.436742	0.292803	H	1.566578	-0.320676	-1.326857	H	1.477056	-1.700469	-0.568716
C	-1.417687	-2.148245	-1.713180	C	1.549087	-2.449644	-1.485465	C	1.578618	-1.807644	1.570374
H	-2.347077	-2.710084	-1.751574	H	2.427496	-2.439645	-2.124402	H	2.314544	-2.604256	1.514257
H	-1.229490	-1.752747	-2.711819	H	1.609036	-3.319501	-0.827523	H	1.849246	-1.161177	2.405507
H	-0.618966	-2.842618	-1.438484	H	0.663550	-2.552740	-2.112353	H	0.620058	-2.263072	1.795121
H	-13.119406	2.169938	1.666886	H	12.873247	2.890463	1.051121	H	13.619531	0.402917	-1.507911
C	-0.307879	-0.082702	-0.811908	C	0.275208	-1.079117	0.215571	C	0.305643	-0.091178	0.187722
H	-0.316524	0.399385	-1.791261	H	0.168035	-1.995821	0.800526	H	0.390962	0.687046	0.948927
H	-0.414180	0.712632	-0.071370	H	0.406411	-0.267490	0.934430	H	0.329833	0.417958	-0.777234
**α-Tocopherol (C15$^{\bullet}$)**				**α-Tocopherol (C16$^{\bullet}$)**				**α-Tocopherol (C17$^{\bullet}$)**			
C	-3.579891	-0.347693	0.338198	C	-3.390423	-0.498043	-0.252157	C	3.541077	-0.394278	-0.278218
C	-5.785291	0.539741	-0.057612	C	-5.568824	0.251303	0.457442	C	5.753644	0.526172	-0.009083
C	-6.186199	-0.629917	-0.694784	C	-5.870991	0.713772	-0.819223	C	6.096215	-0.457291	0.913893
C	-5.175794	-1.690737	-1.046593	C	-4.801698	0.780479	-1.878435	C	5.041700	-1.378270	1.470350
C	-3.752032	-1.164227	-0.933860	C	-3.411755	0.625575	-1.277722	C	3.639573	-0.872382	1.162435
C	-6.699266	1.547581	0.258880	C	-6.536937	0.203266	1.463136	C	6.710645	1.407427	-0.520870
C	-7.534230	-0.820982	-1.004427	C	-7.173880	1.116865	-1.117900	C	7.425074	-0.591363	1.321267
H	-5.306865	-2.552336	-0.390320	H	-4.975233	0.002942	-2.623808	H	5.176613	-2.378510	1.055853
H	-3.528237	-0.511257	-1.776299	H	-3.123180	1.542851	-0.766488	H	3.398432	-0.027004	1.805001
C	-8.437419	0.181766	-0.692218	C	-8.130161	1.073118	-0.116927	C	8.369071	0.285642	0.813895
C	-8.039006	1.365154	-0.073228	C	-7.829613	0.629052	1.169474	C	8.029977	1.287790	-0.092856
O	-4.472101	0.781243	0.271179	O	-4.303358	-0.156653	0.808156	O	4.466201	0.702404	-0.447848
C	-2.200270	0.294120	0.419300	C	-2.044086	-0.595672	0.454924	C	2.182885	0.226797	-0.577605
H	-2.212950	0.983351	1.263954	H	-2.161082	-1.300962	1.277974	H	2.255344	0.712288	-1.551034
H	-2.060871	0.896374	-0.478077	H	-1.830883	0.376198	0.899051	H	2.007772	1.013997	0.155364
C	-1.032539	-0.674169	0.576153	C	-0.873412	-1.031516	-0.420118	C	1.005084	-0.742443	-0.585384
H	-1.189153	-1.297819	1.453055	H	-1.098077	-1.991445	-0.878293	H	1.173399	-1.514222	-1.332514
H	-0.975219	-1.340523	-0.284830	H	-0.719326	-0.317825	-1.229568	H	0.919135	-1.244261	0.378822
C	1.536452	-0.808391	0.664875	C	1.675387	-1.403397	-0.425286	C	-1.557963	-0.896538	-0.783438
C	2.789654	0.066996	0.730529	C	2.895641	-1.385040	0.497699	C	-2.812357	-0.031513	-0.929164
H	2.832044	0.546222	1.711358	H	2.820963	-2.222961	1.195001	H	-2.810384	0.427678	-1.921234
H	2.691875	0.870306	-0.001471	H	2.860580	-0.477190	1.101914	H	-2.751044	0.789046	-0.212175
C	4.099121	-0.669869	0.470490	C	4.241599	-1.440618	-0.216086	C	-4.133542	-0.764791	-0.714669
H	4.031699	-1.198064	-0.482712	H	4.297181	-0.639002	-0.954134	H	-4.088900	-1.316329	0.227536
H	4.269112	-1.424146	1.236012	H	4.337838	-2.377783	-0.759146	H	-4.276879	-1.501137	-1.502493
C	5.293408	0.275619	0.436724	C	5.407817	-1.305111	0.763013	C	-5.327013	0.187154	-0.691577
H	5.121720	1.051072	-0.311139	H	5.275460	-0.396663	1.352653	H	-5.139328	0.971113	0.045198
H	5.386936	0.779721	1.400246	H	5.341776	-2.134798	1.482707	H	-5.421859	0.685444	-1.658648
C	6.627794	-0.425518	0.117164	C	6.760175	-1.288148	0.133014	C	-6.679265	-0.451997	-0.367806
H	6.514725	-0.923952	-0.847401	C	7.901441	-0.643803	0.845899	H	-6.552600	-0.969693	0.616656
C	7.744630	0.618810	-0.007958	H	7.582178	-0.298326	1.828914	C	-7.759609	0.596565	-0.317841
H	7.423285	1.402472	-0.694726	H	8.701934	-1.372428	1.013252	H	-7.377251	1.478066	0.209811
H	7.887209	1.093432	0.965314	C	8.498881	0.543984	0.076202	C	-8.999502	0.111946	0.392034
C	9.068862	0.036342	-0.486928	H	7.730011	1.307340	-0.048783	H	-8.790920	-0.285233	1.419640
H	8.923390	-0.429699	-1.463640	H	8.776068	0.211011	-0.922069	H	-9.447044	-0.730305	-0.140806
H	9.371978	-0.756015	0.195193	C	9.706739	1.141820	0.788346	C	-10.059329	1.202045	0.535406
C	10.161841	1.093909	-0.588706	H	9.438387	1.343934	1.826629	H	-9.567548	2.112679	0.884244
H	9.785900	1.933900	-1.175283	H	10.513323	0.405073	0.814822	H	-10.460894	1.432342	-0.453714
H	10.379924	1.485810	0.407688	C	10.239362	2.429994	0.161648	C	-11.212004	0.874307	1.485969
C	11.466054	0.604342	-1.217974	H	9.425621	3.157365	0.148785	H	-10.791107	0.699107	2.478224
H	11.235294	0.226985	-2.215900	C	11.376759	2.993102	1.008643	C	-12.184868	2.047954	1.574762
C	12.448990	1.763163	-1.355301	H	11.055022	3.169811	2.032390	H	-11.670271	2.956753	1.880957
H	12.021025	2.576398	-1.937011	H	12.207648	2.288890	1.033890	H	-12.632817	2.232518	0.598171
H	12.706800	2.152524	-0.371016	C	7.050387	-2.186285	-1.020972	C	-6.997350	-1.557439	-1.383594
C	6.962972	-1.442229	1.150423	H	6.895137	-3.238253	-0.755763	H	-6.941345	-1.139620	-2.389233
H	6.818469	-2.495694	0.990647	H	6.404595	-1.985211	-1.875506	H	-6.319561	-2.411689	-1.304326
H	7.287553	-1.114939	2.125798	H	8.081027	-2.080375	-1.349190	H	-8.009939	-1.923470	-1.235965
C	12.098597	-0.525444	-0.409931	C	10.713450	2.211396	-1.272547	C	-11.954819	-0.387746	1.056968
H	12.270413	-0.198842	0.615625	H	11.479401	1.436242	-1.297107	H	-12.319346	-0.271699	0.035699
H	11.467498	-1.408845	-0.382624	H	9.902397	1.907209	-1.927512	H	-11.299713	-1.253363	1.082287
H	13.057853	-0.811207	-0.835200	H	11.146956	3.123132	-1.677010	H	-12.810605	-0.581129	1.704155
C	-9.083671	2.410665	0.218484	C	-8.925957	0.622432	2.202537	C	9.113957	2.213365	-0.581191
H	-9.835304	2.038158	0.916279	H	-9.740022	-0.043775	1.913196	H	9.877117	1.672791	-1.143538
H	-8.649951	3.298002	0.659005	H	-8.565392	0.288497	3.165944	H	8.718994	2.981693	-1.232066
H	-9.594615	2.719188	-0.694029	H	-9.341131	1.621233	2.341222	H	9.603663	2.717627	0.252676
C	-6.226751	2.805201	0.941086	C	-6.172155	-0.297531	2.836869	C	6.301774	2.464534	-1.513712
H	-5.169466	2.742116	1.157737	H	-5.143125	-0.628716	2.857400	H	5.256506	2.355923	-1.767479
H	-6.392791	3.676841	0.311081	H	-6.291313	0.486039	3.582589	H	6.449717	3.462649	-1.105758
H	-6.759203	2.965728	1.875642	H	-6.806471	-1.131367	3.128812	H	6.886622	2.389401	-2.427727
C	-7.996977	-2.086114	-1.672904	C	-7.531733	1.601430	-2.495906	C	7.823491	-1.660238	2.301512
H	-7.685656	-2.111728	-2.716514	H	-7.116185	2.591129	-2.681298	H	7.468000	-1.422299	3.303188
H	-7.573324	-2.959922	-1.184760	H	-7.135985	0.933602	-3.256694	H	7.395226	-2.619846	2.023613

H	-9.076176	-2.161122	-1.643007	H	-8.605985	1.661923	-2.612371	H	8.900718	-1.758437	2.341452
O	-9.757099	-0.025994	-1.024355	O	-9.401763	1.493946	-0.434619	O	9.671027	0.141640	1.245552
H	-10.284908	0.702259	-0.697295	H	-9.975342	1.368948	0.321151	H	10.226916	0.765446	0.779352
H	-5.357293	-2.048624	-2.057115	H	-4.869988	1.727890	-2.407562	H	5.172165	-1.475442	2.545695
H	-3.043672	-1.987694	-0.948543	H	-2.682914	0.431752	-2.059606	H	2.907130	-1.652783	1.345876
C	-3.902324	-1.153666	1.591097	C	-3.812839	-1.834449	-0.850795	C	3.895816	-1.487417	-1.278481
H	-3.354341	-2.092292	1.596544	H	-3.244464	-2.051268	-1.751631	H	3.318983	-2.387475	-1.082763
H	-3.632328	-0.582001	2.475317	H	-3.646698	-2.629730	-0.128653	H	3.679108	-1.144340	-2.286786
H	-4.964547	-1.374323	1.638382	H	-4.868492	-1.819990	-1.105637	H	4.952168	-1.733519	-1.219815
H	1.542015	-1.332793	-0.293982	H	1.786205	-0.589043	-1.145675	H	-1.578299	-1.346423	0.212868
C	1.523171	-1.847532	1.783732	C	1.574926	-2.718380	-1.194546	C	-1.532801	-2.020468	-1.816841
H	2.418505	-2.461528	1.768097	H	2.475622	-2.914554	-1.768111	H	-2.417221	-2.643718	-1.734725
H	1.471220	-1.349234	2.752009	H	1.428560	-3.545525	-0.499660	H	-1.511896	-1.597873	-2.821946
H	0.669287	-2.513482	1.700763	H	0.741070	-2.710654	-1.890237	H	-0.662705	-2.661686	-1.699115
H	13.368412	1.442726	-1.839634	H	11.745066	3.930848	0.599138	H	-12.989743	1.851137	2.283097
C	0.289807	0.076575	0.708993	C	0.412142	-1.138120	0.395567	C	-0.307617	-0.020483	-0.879740
H	0.290519	0.640374	1.644517	H	0.301892	-1.932296	1.137518	H	-0.261526	0.417665	-1.879509
H	0.357652	0.811155	-0.094533	H	0.548880	-0.210906	0.953528	H	-0.412466	0.811536	-0.182231
**α-Tocopherol (C18•)**				**α-Tocopherol (C19•)**				**α-Tocopherol (C20•)**			
C	3.553497	-0.425531	-0.289755	C	3.548606	-0.412066	-0.235796	C	3.549539	-0.388463	-0.302409
C	5.743619	0.554096	-0.041473	C	5.760200	0.529682	-0.053225	C	5.757971	0.536216	-0.017103
C	6.126591	-0.428610	0.866205	C	6.123077	-0.403013	0.913487	C	6.120049	-0.488391	0.851809
C	5.106987	-1.386862	1.424581	C	5.083011	-1.299823	1.533334	C	5.081007	-1.445797	1.374622
C	3.687015	-0.916545	1.143692	C	3.673625	-0.814403	1.225863	C	3.670739	-0.937702	1.111148
C	6.666869	1.470583	-0.553220	C	6.703360	1.387150	-0.626062	C	6.699917	1.451028	-0.496041
C	7.463455	-0.526435	1.257505	C	7.458944	-0.509707	1.305554	C	7.454774	-0.629936	1.236538
H	5.263975	-2.377377	0.994789	H	5.214537	-2.318428	1.165343	H	5.219059	-2.423041	0.909496
H	3.432654	-0.085640	1.799889	H	3.441450	0.062593	1.828099	H	3.431505	-0.128156	1.798970
C	8.373856	0.385624	0.750642	C	8.389579	0.344289	0.737843	C	8.384081	0.281352	0.763515
C	7.993837	1.386986	-0.140749	C	8.030294	1.295436	-0.214829	C	8.025216	1.323379	-0.089358
O	4.445832	0.696589	-0.463163	O	4.463678	0.677554	-0.478592	O	4.461874	0.722241	-0.430394
C	2.175312	0.164198	-0.560424	C	2.182657	0.187853	-0.544111	C	2.182940	0.237248	-0.551558
H	2.216445	0.652229	-1.534483	H	2.241980	0.634066	-1.537010	H	2.234926	0.762828	-1.505217
H	1.998418	0.946620	0.177155	H	2.009521	1.002567	0.158523	H	2.018599	0.992458	0.216653
C	1.019545	-0.830652	-0.543689	C	1.012395	-0.789395	-0.501703	C	1.010540	-0.737841	-0.575852
H	1.194862	-1.607954	-1.283674	H	1.177514	-1.584649	-1.224291	H	1.171218	-1.481408	-1.352619
H	0.954863	-1.322476	0.427329	H	0.942888	-1.258293	0.480181	H	0.943361	-1.273909	0.371038
C	-1.544116	-1.029079	-0.661042	C	-1.551847	-0.965895	-0.663072	C	-1.553589	-0.897799	-0.714907
C	-2.814253	-0.212788	-0.912253	C	-2.809889	-0.111145	-0.833937	C	-2.813747	-0.035598	-0.817870
H	-2.871956	0.028414	-1.976925	H	-2.825852	0.292079	-1.849733	H	-2.842410	0.429479	-1.806409
H	-2.727971	0.739552	-0.386547	H	-2.739483	0.747206	-0.164144	H	-2.736298	0.779212	-0.096528
C	-4.110705	-0.886044	-0.472080	C	-4.125139	-0.833374	-0.557434	C	-4.122195	-0.780699	-0.572445
H	-4.037632	-1.141571	0.587132	H	-4.075933	-1.305809	0.426434	H	-4.057924	-1.317588	0.376769
H	-4.247553	-1.821123	-1.010223	H	-4.267101	-1.630667	-1.282482	H	-4.268613	-1.528379	-1.347358
C	-5.330247	0.006810	-0.686433	C	-5.322588	0.112106	-0.600431	C	-5.325744	0.156652	-0.536932
H	-5.138617	0.976413	-0.221013	H	-5.127878	0.954869	0.064919	H	-5.126080	0.957608	0.176579
H	-5.453388	0.197080	-1.755479	H	-5.416805	0.529436	-1.606178	H	-5.440645	0.633969	-1.513451
C	-6.640333	-0.545802	-0.121054	C	-6.660505	-0.514843	-0.202476	C	-6.650836	-0.508935	-0.158721
H	-6.498049	-0.776210	0.936727	H	-6.543889	-0.951212	0.793307	H	-6.521925	-0.983744	0.817635
C	-7.755440	0.503811	-0.193432	C	-7.739701	0.568348	-0.122511	C	-7.748767	0.549560	-0.024051
H	-7.397025	1.393550	0.335396	H	-7.397098	1.348599	0.560549	H	-7.384482	1.341876	0.633555
H	-7.813430	0.814899	-1.268078	H	-7.847868	1.044754	-1.099166	H	-7.912635	1.012183	-1.000547
C	-9.080516	0.083785	0.396640	C	-9.116906	0.103972	0.346655	C	-9.077486	0.040620	0.529014
H	-9.543395	-0.661054	-0.255760	H	-8.956819	-0.587288	1.215755	H	-8.911024	-0.477648	1.474917
C	-10.006148	1.271199	0.517874	H	-9.553312	-0.526171	-0.431055	H	-9.511544	-0.683011	-0.158013
H	-9.475005	2.090445	1.014649	C	-10.031129	1.259381	0.667597	C	-10.089922	1.160503	0.766982
H	-10.330814	1.696596	-0.466432	H	-9.529155	1.919980	1.383532	H	-9.614825	1.927747	1.384876
C	-11.293884	1.019136	1.306549	C	-11.347439	0.820568	1.245347	H	-10.275978	1.650613	-0.231855
H	-10.991860	0.692859	2.302041	H	-11.216993	0.089658	2.082942	C	-11.395622	0.749563	1.380036
C	-12.135723	2.288643	1.423090	C	-12.115902	2.009167	1.818445	C	-12.215651	1.985636	1.624422
H	-11.563384	3.092742	1.884165	H	-11.521568	2.517950	2.576543	H	-11.710901	2.696076	2.280977
H	-12.446505	2.625725	0.433737	H	-12.314625	2.720701	1.017218	H	-12.423094	2.531832	0.661724
C	-7.072646	-1.823984	-0.830785	C	-7.068481	-1.627825	-1.164502	C	-7.040785	-1.586302	-1.168506
H	-7.283251	-1.608645	-1.881104	H	-7.208865	-1.216263	-2.165015	H	-7.156483	-1.140244	-2.157161
H	-6.315543	-2.606297	-0.784641	H	-6.324500	-2.420356	-1.220134	H	-6.294438	-2.374672	-1.234710
H	-7.981368	-2.194707	-0.365594	H	-8.008745	-2.070361	-0.851003	H	-7.985787	-2.044566	-0.896952
C	-12.109427	-0.099105	0.668704	C	-12.240512	0.112658	0.220976	C	-12.154091	-0.146127	0.440133
H	-12.336939	0.157221	-0.368487	H	-12.441833	0.800090	-0.600950	H	-12.298774	0.353585	-0.559942
H	-11.537007	-1.022248	0.677616	H	-11.748324	-0.766288	-0.189671	H	-11.659427	-1.096834	0.243972
H	-13.051483	-0.259130	1.195495	H	-13.185451	-0.207507	0.669859	H	-13.151106	-0.373582	0.819274
C	9.043277	2.351182	-0.629394	C	9.101238	2.198204	-0.770128	C	9.095321	2.281736	-0.543396
H	9.817826	1.840554	-1.204108	H	9.863973	1.630103	-1.304929	H	9.858709	1.775204	-1.136292
H	8.618064	3.112538	-1.269291	H	8.693043	2.920980	-1.463754	H	8.687604	3.074861	-1.155378
H	9.524575	2.862438	0.204987	H	9.593967	2.758135	0.025735	H	9.587227	2.751704	0.308777
C	6.214652	2.526535	-1.528437	C	6.273206	2.390306	-1.664919	C	6.270019	2.552122	-1.430507

H	5.169852	2.390260	-1.770645	H	5.222991	2.269574	-1.891367	H	5.223341	2.445780	-1.679213
H	6.338606	3.523798	-1.110591	H	6.429409	3.408300	-1.313478	H	6.412669	3.529950	-0.974453
H	6.790050	2.479037	-2.450269	H	6.838978	2.266390	-2.585521	H	6.846426	2.528740	-2.352544
C	7.905486	-1.593606	2.220710	C	7.879318	-1.524738	2.332647	C	7.874935	-1.742808	2.156885
H	7.555157	-1.377560	3.229156	H	7.540415	-1.236674	3.326860	H	7.531178	-1.558335	3.173808
H	7.503062	-2.562581	1.936850	H	7.451128	-2.499197	2.112212	H	7.450958	-2.691132	1.836795
H	8.985522	-1.659738	2.247188	H	8.957480	-1.616681	2.358736	H	8.953277	-1.833601	2.177930
O	9.683893	0.277889	1.167056	O	9.698404	0.228602	1.154714	O	9.691181	0.129882	1.173890
H	10.217174	0.917736	0.696128	H	10.240383	0.847015	0.665392	H	10.238819	0.773670	0.725234
H	5.255444	-1.494508	2.496479	H	5.232495	-1.343563	2.609523	H	5.226232	-1.595478	2.441872
H	2.979525	-1.719241	1.328954	H	2.947557	-1.586482	1.462903	H	2.948256	-1.733830	1.264853
C	3.919662	-1.498767	-1.307279	C	3.891092	-1.555116	-1.183468	C	3.897027	-1.428590	-1.360237
H	3.368844	-2.415283	-1.113133	H	3.326429	-2.447860	-0.927402	H	3.330219	-2.342131	-1.200948
H	3.679552	-1.150191	-2.308368	H	3.652033	-1.268946	-2.204525	H	3.664701	-1.038023	-2.347593
H	4.982612	-1.718540	-1.266970	H	4.950387	-1.789541	-1.132766	H	4.955828	-1.668734	-1.327471
H	-1.568183	-1.374612	0.375804	H	-1.561743	-1.376129	0.350122	H	-1.548857	-1.365345	0.273123
C	-1.478690	-2.250885	-1.575319	C	-1.527406	-2.127947	-1.653223	C	-1.544436	-2.001927	-1.769851
H	-2.387374	-2.840058	-1.504895	H	-2.407579	-2.753652	-1.546237	H	-2.417422	-2.640433	-1.680201
H	-1.363597	-1.930865	-2.611394	H	-1.508279	-1.744248	-2.673636	H	-1.549741	-1.561344	-2.767222
H	-0.642724	-2.899302	-1.325592	H	-0.655068	-2.760262	-1.511874	H	-0.665307	-2.634555	-1.683551
H	-13.034094	2.120779	2.018494	H	-13.063005	1.700694	2.267369	H	-13.187735	1.758988	2.064277
C	-0.311081	-0.140205	-0.832049	C	-0.308310	-0.085828	-0.803390	C	-0.310292	-0.013026	-0.822137
H	-0.299638	0.252679	-1.851594	H	-0.277140	0.319480	-1.817461	H	-0.290088	0.448534	-1.812018
H	-0.409805	0.720799	-0.169972	H	-0.409391	0.768041	-0.132435	H	-0.400492	0.801805	-0.102759

**α-Tocopherol (C21$^•$)**				**α-Tocopherol (C22$^•$)**			
C	3.548289	-0.424206	-0.352196	C	3.546319	-0.398746	-0.296867
C	5.724809	0.563953	-0.023137	C	5.749214	0.538493	-0.019527
C	6.129359	-0.494142	0.784703	C	6.115665	-0.477288	0.857185
C	5.129852	-1.520378	1.251008	C	5.082008	-1.435936	1.388331
C	3.700650	-1.047335	1.027091	C	3.668755	-0.938477	1.120545
C	6.626588	1.545585	-0.442770	C	6.686120	1.452134	-0.508993
C	7.468947	-0.602169	1.163511	C	7.451187	-0.610892	1.241481
H	5.298351	-2.461040	0.724859	H	5.225595	-2.416435	0.931920
H	3.439507	-0.287454	1.762224	H	3.424470	-0.125646	1.802862
C	8.358690	0.374602	0.748932	C	8.376462	0.299819	0.758678
C	7.956007	1.449832	-0.041022	C	8.012589	1.332772	-0.103377
O	4.422446	0.720649	-0.428521	O	4.448904	0.716227	-0.431291
C	2.160758	0.171077	-0.555548	C	2.176698	0.219314	-0.550942
H	2.180078	0.729172	-1.491857	H	2.227401	0.738319	-1.508242
H	1.992405	0.896252	0.240134	H	2.009872	0.979301	0.212071
C	1.011703	-0.830977	-0.588356	C	1.008109	-0.760141	-0.568496
H	1.185041	-1.564391	-1.372479	H	1.172887	-1.509375	-1.338796
H	0.957943	-1.376667	0.353961	H	0.941686	-1.288295	0.382821
C	-1.553196	-1.026478	-0.672104	C	-1.556561	-0.922558	-0.704596
C	-2.825767	-0.199208	-0.868727	C	-2.813652	-0.057088	-0.814798
H	-2.906305	0.077337	-1.922927	H	-2.840446	0.399536	-1.807026
H	-2.727478	0.734580	-0.313442	H	-2.734041	0.762813	-0.099633
C	-4.112207	-0.888331	-0.424547	C	-4.123668	-0.797911	-0.565114
H	-4.028156	-1.153717	0.631524	H	-4.064380	-1.322490	0.390949
H	-4.243813	-1.818478	-0.971688	H	-4.266662	-1.554443	-1.331674
C	-5.337195	-0.000193	-0.622879	C	-5.321927	0.146015	-0.547853
H	-5.130262	0.982644	-0.196567	H	-5.116196	0.958970	0.149948
H	-5.500775	0.156209	-1.691891	H	-5.434181	0.604921	-1.533111
C	-6.630558	-0.531209	-0.001829	C	-6.648981	-0.507370	-0.155948
H	-6.459717	-0.658793	1.070606	H	-6.520727	-0.962477	0.829457
C	-7.751582	0.495456	-0.179884	C	-7.734186	0.565034	-0.041398
H	-7.391337	1.465073	0.168789	H	-7.366925	1.363247	0.605643
H	-7.954468	0.611474	-1.247801	H	-7.890566	1.012782	-1.025694
C	-9.054878	0.179010	0.548609	C	-9.068966	0.071146	0.505966
H	-8.837825	-0.019787	1.601090	H	-8.901676	-0.450816	1.450093
H	-9.495609	-0.729645	0.142535	H	-9.515213	-0.651718	-0.174422
C	-10.062999	1.321745	0.440412	C	-10.057110	1.209023	0.732793
H	-9.594447	2.242220	0.796427	H	-9.628167	1.927974	1.431464
H	-10.324279	1.494930	-0.605380	H	-10.220418	1.744007	-0.205548
C	-11.372519	1.134096	1.213183	C	-11.410979	0.738687	1.265504
H	-11.068422	0.855472	2.246405	H	-11.240037	0.166760	2.180079
C	-12.218496	2.382933	1.110393	C	-12.287201	1.950079	1.634146
H	-11.676527	3.265921	1.462211	H	-11.795011	2.557614	2.391080
C	-7.017106	-1.886403	-0.589526	H	-12.457675	2.567084	0.754562
H	-7.174550	-1.791850	-1.664702	C	-7.049128	-1.600071	-1.144456
H	-6.246670	-2.635423	-0.422368	H	-7.171343	-1.172865	-2.140067
H	-7.938526	-2.252107	-0.147928	H	-6.298090	-2.382090	-1.202254
C	-12.146023	-0.069964	0.667897	H	-7.986451	-2.068661	-0.861028
H	-12.332604	0.084229	-0.395155	C	-12.114121	-0.126441	0.281671
H	-11.625287	-1.019637	0.818795	H	-11.985732	0.045231	-0.775017

H	-13.113733	-0.131592	1.163495	H	-12.880590	-0.814235	0.594961
C	8.984549	2.477156	-0.436246	C	9.079239	2.289712	-0.567809
H	9.773364	2.035811	-1.047420	H	9.843090	1.779268	-1.156456
H	8.544995	3.281255	-1.010714	H	8.669441	3.076190	-1.186845
H	9.449738	2.925763	0.442208	H	9.570830	2.768599	0.279429
C	6.149608	2.682568	-1.309027	C	6.251877	2.543220	-1.453144
H	5.106851	2.550050	-1.561522	H	5.204825	2.433216	-1.698750
H	6.255553	3.636284	-0.795681	H	6.394697	3.525591	-1.007200
H	6.722193	2.737223	-2.232101	H	6.826280	2.511596	-2.376073
C	7.936683	-1.749685	2.015825	C	7.877245	-1.713885	2.170823
H	7.591425	-1.638954	3.042928	H	7.533701	-1.522327	3.186509
H	7.548480	-2.693497	1.641593	H	7.458545	-2.667324	1.859095
H	9.017824	-1.799003	2.028618	H	8.955943	-1.798981	2.191992
O	9.671006	0.253049	1.151843	O	9.682668	0.156116	1.168930
H	10.184663	0.965605	0.772255	H	10.230547	0.796460	0.715427
H	5.287455	-1.730601	2.306126	H	5.227934	-1.575599	2.456777
H	3.007271	-1.875796	1.138633	H	2.951711	-1.738841	1.279397
C	3.917586	-1.394167	-1.468071	C	3.898676	-1.444400	-1.347737
H	3.377845	-2.330835	-1.356247	H	3.337620	-2.360673	-1.182944
H	3.667696	-0.956351	-2.430901	H	3.666428	-1.061557	-2.338204
H	4.983043	-1.605150	-1.454101	H	4.958513	-1.678551	-1.312390
H	-1.559396	-1.414698	0.349699	H	-1.550454	-1.379514	0.288274
C	-1.502920	-2.208013	-1.638531	C	-1.550976	-2.036794	-1.748613
H	-2.406471	-2.806588	-1.578201	H	-2.417078	-2.683398	-1.646666
H	-1.406868	-1.844477	-2.661853	H	-1.564966	-1.607201	-2.750470
H	-0.660174	-2.861795	-1.430968	H	-0.666905	-2.661259	-1.661635
H	-13.146186	2.283794	1.680587	H	-13.252473	1.632357	2.018238
C	-0.323520	-0.130164	-0.826136	C	-0.313159	-0.039226	-0.822950
H	-0.328939	0.305610	-1.827922	H	-0.293301	0.410822	-1.818023
H	-0.411196	0.701881	-0.126636	H	-0.402181	0.784028	-0.113012

Structure of α-Tocopherol and the corresponding radical species in the HAT mechanism optimized at the M06-2X/6-311++G(2d,2p) level of theory in gas phase.

**M06-2X/6-311++G(2d,2p)**											
**α-Tocopherol**				**α-Tocopherol (O$^{\bullet}$)**				**α-Tocopherol (C1$^{\bullet}$)**			
C	-3.60970400	-0.36270000	0.33903600	C	3.653263	-0.355192	-0.335018	C	3.651578	-0.321975	-0.333209
C	-5.81748200	0.53355700	-0.00151900	C	5.860192	0.540472	0.012531	C	5.859429	0.579228	0.003456
C	-6.16752600	-0.52451700	-0.83776200	C	6.210322	-0.522353	0.842691	C	6.221787	-0.509971	0.813480
C	-5.11767600	-1.49608300	-1.31623600	C	5.160766	-1.497457	1.314567	C	5.178409	-1.492009	1.276759
C	-3.71080600	-0.97292100	-1.05621200	C	3.753740	-0.973679	1.056631	C	3.766150	-0.971280	1.042736
C	-6.76881700	1.46077900	0.43861000	C	6.811235	1.470938	-0.421339	C	6.790443	1.513435	-0.416468
C	-7.49939500	-0.68549900	-1.23106800	C	7.542007	-0.684804	1.236016	C	7.564279	-0.697861	1.172060
H	-5.25793900	-2.46108800	-0.81899900	H	5.302038	-2.459378	0.811672	H	5.317678	-2.443960	0.753618
H	-3.46489800	-0.19071100	-1.77802800	H	3.506779	-0.195963	1.782929	H	3.519364	-0.212412	1.788644
C	-8.43975700	0.23867400	-0.79811900	C	8.482073	0.242563	0.809300	C	8.506019	0.224786	0.760146
C	-8.09230100	1.31187200	0.02560000	C	8.134512	1.320438	-0.008244	C	8.150349	1.372860	-0.009207
O	-4.52481200	0.74098900	0.41600000	O	4.567693	0.749542	-0.404700	O	4.558745	0.790692	-0.380639
C	-2.24544700	0.28052700	0.57950700	C	2.288756	0.288560	-0.572651	C	2.281681	0.318672	-0.546230
H	-2.30287600	0.82823400	1.52412900	H	2.346519	0.841944	-1.513938	H	2.327775	0.890227	-1.477150
H	-2.08556700	1.02539200	-0.20413000	H	2.127802	1.028619	0.215307	H	2.123380	1.042831	0.256834
C	-1.06541800	-0.68588400	0.62308000	C	1.109403	-0.678353	-0.622879	C	1.107649	-0.654343	-0.604455
H	-1.22688100	-1.42321600	1.41080400	H	1.271939	-1.410854	-1.414878	H	1.271584	-1.376516	-1.405594
H	-0.99698700	-1.23964500	-0.31762900	H	1.040646	-1.237775	0.314451	H	1.044865	-1.225248	0.326391
C	1.50199800	-0.84399800	0.75121400	C	-1.457812	-0.837395	-0.753863	C	-1.459327	-0.825176	-0.725648
C	2.76535300	0.01886100	0.83151000	C	-2.721681	0.025093	-0.829936	C	-2.727420	0.030569	-0.807749
H	2.79853400	0.50836700	1.81153500	H	-2.754464	0.520430	-1.807040	H	-2.762026	0.519472	-1.788012
H	2.68585100	0.82083700	0.09155300	H	-2.643259	0.822682	-0.085137	H	-2.653376	0.833373	-0.068096
C	4.07256800	-0.73514000	0.60081200	C	-4.028564	-0.731138	-0.604722	C	-4.030947	-0.730252	-0.578524
H	4.00773100	-1.29006500	-0.34168300	H	-3.964054	-1.291646	0.334486	H	-3.965640	-1.282332	0.365575
H	4.20987000	-1.47561200	1.38900300	H	-4.164791	-1.466973	-1.397428	H	-4.162317	-1.473358	-1.365187
C	5.28288800	0.19411200	0.55584600	C	-5.239535	0.197028	-0.555098	C	-5.246331	0.192648	-0.539510
H	5.08586400	0.99411300	-0.16409400	H	-5.043575	0.992840	0.169759	H	-5.056417	0.994404	0.180364
H	5.40081000	0.68118500	1.53052800	H	-5.357061	0.689844	-1.526937	H	-5.362658	0.678028	-1.515226
C	6.60470100	-0.48335200	0.17953900	C	-6.561174	-0.483548	-0.183831	C	-6.566353	-0.491045	-0.168202
H	6.46831600	-0.96084300	-0.79813800	H	-6.425194	-0.966787	0.791074	H	-6.432235	-0.966281	0.810877
C	7.70446300	0.57322000	0.03358200	C	-7.661747	0.571405	-0.032375	C	-7.671948	0.560438	-0.029482
H	7.35283700	1.34779400	-0.65460800	H	-7.311145	1.342082	0.660695	H	-7.327886	1.337546	0.659682
H	7.84994400	1.06662300	1.00128100	H	-7.806841	1.070491	-0.997214	H	-7.814222	1.052035	-0.998589

C	9.04660200	0.04503000	-0.46715100	C	-9.003904	0.039343	0.464193	C	-9.014591	0.026716	0.463879
H	8.89069400	-0.52220400	-1.39143600	H	-8.848301	-0.533305	1.385185	H	-8.862111	-0.537202	1.390748
H	9.46035200	-0.65358200	0.26102600	H	-9.416651	-0.655175	-0.268455	H	-9.420075	-0.675794	-0.265152
C	10.05182100	1.16353200	-0.72640000	C	-10.010058	1.155610	0.729380	C	-10.027100	1.141017	0.712833
H	9.59837100	1.89902900	-1.39777700	H	-9.557594	1.887378	1.405480	H	-9.581985	1.880798	1.385073
H	10.25677700	1.69082200	0.21243800	H	-10.214671	1.688370	-0.206440	H	-10.227948	1.664321	-0.229120
C	11.38109100	0.70480900	-1.33128800	C	-11.339469	0.692399	1.330526	C	-11.358488	0.677827	1.309618
H	11.16063000	0.19326400	-2.27424700	H	-11.119364	0.175370	2.270573	H	-11.142713	0.172278	2.256882
C	12.26552900	1.91143700	-1.63802600	C	-12.224936	1.896586	1.643818	C	-12.252019	1.881008	1.603112
H	11.76149500	2.61421900	-2.30229000	H	-11.721861	2.595716	2.312646	H	-11.757920	2.589576	2.268690
H	12.51522600	2.44207700	-0.71642400	H	-12.474305	2.432563	0.725220	H	-12.496307	2.406101	0.676900
C	6.99258800	-1.56346700	1.18878900	C	-6.947597	-1.557864	-1.199810	C	-6.944127	-1.574626	-1.177576
H	7.14261200	-1.11897800	2.17642300	H	-7.097187	-1.107575	-2.184879	H	-7.092157	-1.132291	-2.166470
H	6.22179600	-2.32742600	1.27808200	H	-6.176231	-2.320765	-1.293097	H	-6.168990	-2.334689	-1.262369
H	7.91494400	-2.06668100	0.90337100	H	-7.869828	-2.063385	-0.918091	H	-7.865013	-2.082432	-0.895615
C	12.11996000	-0.27270600	-0.41872300	C	-12.077013	-0.280129	0.411584	C	-12.084688	-0.308420	0.396266
H	12.28111100	0.17771200	0.56408600	H	-12.237737	0.176052	-0.568634	H	-12.239647	0.135654	-0.590416
H	11.56755700	-1.20060100	-0.27730400	H	-11.523888	-1.206796	0.265029	H	-11.526471	-1.234324	0.264922
H	13.09608200	-0.52698700	-0.83294800	H	-13.053271	-0.537528	0.823555	H	-13.063024	-0.565330	0.803558
C	-9.17318600	2.27610300	0.44376600	C	9.215064	2.287867	-0.419834	C	9.105576	2.334678	-0.344877
H	-9.95861000	1.77234900	1.01492700	H	10.001244	1.788057	-0.993413	H	8.850140	3.187119	-0.951556
H	-8.78674200	3.07424100	1.06851800	H	8.828550	3.089472	-1.040088	H	10.115793	2.312275	0.034940
H	-9.64246700	2.74584500	-0.42507800	H	9.683390	2.752715	0.452152	C	6.379832	2.681752	-1.265672
C	-6.35272800	2.59676200	1.33869900	C	6.395055	2.612008	-1.314928	H	5.329903	2.616490	-1.528374
H	-5.29930300	2.52233300	1.58447700	H	5.341861	2.538354	-1.561929	H	6.544598	3.623793	-0.738595
H	-6.52112800	3.56179100	0.85806300	H	6.562457	3.574257	-0.828404	H	6.967460	2.717264	-2.184759
H	-6.92228200	2.58986000	2.26879500	H	6.965300	2.611045	-2.244625	C	7.971798	-1.889321	1.996951
C	-7.90457600	-1.83288300	-2.11691300	C	7.947297	-1.837197	2.115285	H	7.616157	-1.793738	3.024910
H	-7.52225100	-1.69942700	-3.13125100	H	7.564134	-1.710063	3.130119	H	7.547368	-2.808375	1.592313
H	-7.50389000	-2.77557300	-1.74283300	H	7.547515	-2.777898	1.735277	H	9.051948	-1.989255	2.023378
H	-8.98531200	-1.91494200	-2.17373900	H	9.028045	-1.918880	2.172422	O	9.804519	0.011624	1.125010
O	-9.73686600	0.06284600	-1.21723000	O	9.778989	0.065089	1.228316	H	10.382557	0.526211	0.558095
H	-10.29461600	0.73615600	-0.82456300	H	5.294704	-1.695318	2.378849	H	5.326321	-1.712454	2.335039
H	-5.25227400	-1.68766500	-2.38158300	H	3.024885	-1.775764	1.166213	H	3.044582	-1.781481	1.139577
H	-2.98150200	-1.77385100	-1.17112700	C	4.005859	-1.355666	-1.434037	C	4.002142	-1.293232	-1.458939
C	-3.96082200	-1.36996300	1.43231300	H	3.424488	-2.271596	-1.329176	H	3.423944	-2.213208	-1.374684
H	-3.37892400	-2.28486200	1.32154600	H	3.802188	-0.919646	-2.412000	H	3.792115	-0.833179	-2.424337
H	-3.75671300	-0.93966300	2.41271500	H	5.062879	-1.615846	-1.391738	H	5.060063	-1.552130	-1.430270
H	-5.01770100	-1.63058900	1.38923900	H	-1.442620	-1.319323	0.231159	H	-1.444543	-1.304911	0.260451
H	1.48639300	-1.32003700	-0.23666100	C	-1.457571	-1.932048	-1.820499	C	-1.449508	-1.922133	-1.789864
C	1.50327300	-1.94501000	1.81128500	H	-2.313524	-2.596679	-1.715218	H	-2.307238	-2.585563	-1.692130
H	2.35958700	-2.60843000	1.70139800	H	-1.498579	-1.485773	-2.817749	H	-1.479458	-1.478096	-2.788521
H	1.54472700	-1.50467900	2.81115500	H	-0.561591	-2.548513	-1.765365	H	-0.554966	-2.539690	-1.724010
H	0.60766000	-2.56173000	1.75312700	H	-13.159348	1.588757	2.114001	H	-13.188826	1.573591	2.068787
H	13.19979500	1.60704700	-2.11073200	C	-0.213070	0.049654	-0.855760	C	-0.219390	0.068757	-0.825822
C	0.25674600	0.04159700	0.85933400	H	-0.195757	0.528018	-1.841330	H	-0.208521	0.554182	-1.807986
H	0.23984900	0.51404700	1.84776000	H	-0.300619	0.858237	-0.124165	H	-0.308332	0.871669	-0.088175
H	0.34321700	0.85459900	0.13252300								
**α-Tocopherol (C2$^{\bullet}$)**				**α-Tocopherol (C3$^{\bullet}$)**				**α-Tocopherol (C4$^{\bullet}$)**			
C	3.637511	-0.446140	-0.314488	C	3.640787	-0.396724	-0.308317	C	3.631747	-0.384728	-0.310663
C	5.800697	0.566655	-0.051057	C	5.849805	0.511437	-0.009954	C	5.839847	0.522283	-0.002180
C	6.240029	-0.453900	0.775068	C	6.196976	-0.504456	0.878183	C	6.188170	-0.503295	0.874299
C	5.262370	-1.478383	1.296258	C	5.144819	-1.449234	1.402990	C	5.137053	-1.455087	1.388409
C	3.823480	-1.022906	1.085505	C	3.739199	-0.937142	1.115611	C	3.730870	-0.941223	1.107010
C	6.687439	1.581819	-0.524886	C	6.803323	1.414003	-0.494383	C	6.792373	1.431301	-0.476387
C	7.596015	-0.527997	1.122995	C	7.528104	-0.648070	1.280618	C	7.519488	-0.650093	1.274950
H	5.430745	-2.438740	0.799566	H	5.283889	-2.437968	0.954416	H	5.277108	-2.438502	0.928593
H	3.572792	-0.239280	1.804056	H	3.493897	-0.119649	1.797428	H	3.484786	-0.131804	1.798114
C	8.476587	0.466439	0.671524	C	8.470636	0.251826	0.803498	C	8.461031	0.256161	0.808022
C	8.051869	1.526481	-0.108010	C	8.126066	1.283441	-0.072878	C	8.115295	1.297329	-0.056517
O	4.507542	0.691366	-0.455990	O	4.557988	0.700174	-0.438828	O	4.547790	0.714542	-0.428759
C	2.243988	0.145437	-0.510104	C	2.277976	0.236181	-0.582071	C	2.268248	0.249830	-0.577067
H	2.250349	0.684753	-1.461220	H	2.337471	0.736242	-1.552634	H	2.327120	0.760972	-1.541878
H	2.087035	0.893689	0.270857	H	2.118564	1.019291	0.163448	H	2.108096	1.024232	0.177336
C	1.092851	-0.855625	-0.505424	C	1.096241	-0.729122	-0.579083	C	1.087520	-0.716678	-0.584963
H	1.257935	-1.608805	-1.277532	H	1.257254	-1.504914	-1.329056	H	1.249265	-1.483711	-1.343737
H	1.053502	-1.386189	0.450519	H	1.025737	-1.235404	0.387858	H	1.017645	-1.234012	0.376157
C	-1.479639	-1.056846	-0.591165	C	-1.471312	-0.888878	-0.702350	C	-1.479877	-0.877700	-0.709805
C	-2.748365	-0.223430	-0.798042	C	-2.733003	-0.028842	-0.826900	C	-2.742478	-0.017620	-0.824438
H	-2.795965	0.091751	-1.846820	H	-2.764184	0.411492	-1.830045	H	-2.774222	0.434076	-1.822501
H	-2.664279	0.694556	-0.208638	H	-2.652880	0.808719	-0.127548	H	-2.663156	0.812007	-0.115601
C	-4.052501	-0.921004	-0.419620	C	-4.041848	-0.768165	-0.560673	C	-4.050523	-0.761293	-0.566527
H	-3.995203	-1.249728	0.623779	H	-3.979088	-1.275756	0.408272	H	-3.987134	-1.279818	0.396569
H	-4.181264	-1.820787	-1.022386	H	-4.179605	-1.546590	-1.311322	H	-4.187546	-1.531262	-1.325979
C	-5.263976	-0.008140	-0.595485	C	-5.250526	0.164303	-0.563345	C	-5.260173	0.169883	-0.558470

H	-5.052404	0.951870	-0.114974	H	-5.052862	0.998692	0.116256	H	-5.063309	0.996685	0.130571
H	-5.401920	0.207499	-1.661026	H	-5.366458	0.602622	-1.561137	H	-5.376665	0.619415	-1.551194
C	-6.578304	-0.553018	-0.028478	C	-6.573994	-0.491326	-0.155504	C	-6.582915	-0.491729	-0.157999
H	-6.429522	-0.734729	1.042672	H	-6.439585	-0.919959	0.844826	H	-6.447955	-0.931585	0.837372
C	-7.679208	0.501679	-0.175445	C	-7.671997	0.573122	-0.063492	C	-7.682017	0.570454	-0.053771
H	-7.315382	1.443929	0.245573	H	-7.319742	1.380267	0.585849	H	-7.330537	1.370521	0.604687
H	-7.847447	0.689259	-1.242163	H	-7.815484	1.018158	-1.054656	H	-7.826071	1.026598	-1.039790
C	-9.008697	0.152131	0.488099	C	-9.015661	0.072791	0.461191	C	-9.025104	0.062779	0.465304
H	-8.831458	-0.121780	1.533935	H	-8.861832	-0.448157	1.412671	H	-8.870633	-0.468809	1.410775
H	-9.443596	-0.724189	0.005532	H	-9.429856	-0.660361	-0.231932	H	-9.438607	-0.662864	-0.236085
C	-10.000349	1.310822	0.430872	C	-10.019136	1.204547	0.663399	C	-10.029738	1.191111	0.680478
H	-9.524831	2.203043	0.849435	H	-9.565109	1.971651	1.298010	H	-9.576446	1.951412	1.323742
H	-10.220944	1.545258	-0.616992	H	-10.222069	1.684965	-0.300694	H	-10.233272	1.682264	-0.278063
C	-11.320685	1.070794	1.167161	C	-11.349922	0.778744	1.288678	C	-11.360015	0.756825	1.300990
H	-11.085776	0.850120	2.213847	H	-11.131460	0.314229	2.256124	H	-11.140968	0.281552	2.263064
C	-12.182694	2.330621	1.125524	C	-12.232510	2.000661	1.534091	C	-12.243852	1.974946	1.560382
H	-11.660018	3.183906	1.558871	H	-11.727951	2.734647	2.163283	H	-11.739993	2.702247	2.197846
H	-12.435062	2.581450	0.092525	H	-12.480198	2.485359	0.586992	H	-12.492143	2.470139	0.618887
C	-6.971364	-1.874913	-0.686282	C	-6.962703	-1.619502	-1.110394	C	-6.970545	-1.609364	-1.125639
H	-7.118301	-1.732166	-1.760364	H	-7.110800	-1.224301	-2.119047	H	-7.119159	-1.202857	-2.129713
H	-6.202219	-2.633887	-0.550092	H	-6.193202	-2.388307	-1.160731	H	-6.200248	-2.376742	-1.184799
H	-7.896618	-2.271524	-0.270736	H	-7.886296	-2.106310	-0.801487	H	-7.893598	-2.100622	-0.822211
C	-12.091556	-0.115111	0.589793	C	-12.089535	-0.241544	0.424835	C	-12.098653	-0.254331	0.425652
H	-12.281818	0.041859	-0.475048	H	-12.248753	0.159838	-0.579308	H	-12.258394	0.158294	-0.573840
H	-11.546590	-1.051614	0.699870	H	-11.538661	-1.176284	0.330302	H	-11.546814	-1.187358	0.320431
H	-13.055370	-0.230919	1.086462	H	-13.066586	-0.473239	0.849973	H	-13.075418	-0.491873	0.848214
C	8.990214	2.621816	-0.536043	C	9.209176	2.223831	-0.537049	C	9.197376	2.244075	-0.510049
H	9.261514	2.536200	-1.590798	H	9.994328	1.690976	-1.081553	H	9.983027	1.718274	-1.060658
H	8.522257	3.596240	-0.399069	H	8.824892	2.990667	-1.201103	H	8.812223	3.018021	-1.165292
H	9.909479	2.646507	0.047657	H	9.678327	2.735277	0.307991	H	9.666080	2.746354	0.340718
C	6.206046	2.576248	-1.373381	C	6.390321	2.504657	-1.450233	C	6.378135	2.532338	-1.419717
H	5.170285	2.581552	-1.667570	H	5.337042	2.419980	-1.693301	H	5.324919	2.449335	-1.663638
H	6.851042	3.344220	-1.766894	H	6.559932	3.492048	-1.017864	H	6.546761	3.514917	-0.976147
C	8.102147	-1.648418	1.987163	H	6.960914	2.450612	-2.378145	H	6.948688	2.489459	-2.348236
H	7.863735	-1.471289	3.039432	C	7.930194	-1.750799	2.222794	C	7.922825	-1.763059	2.204471
H	7.644246	-2.596868	1.707602	H	7.546964	-1.566506	3.228793	H	7.539508	-1.590634	3.212539
H	9.180255	-1.743231	1.905160	H	9.010714	-1.831841	2.284941	H	7.521816	-2.719071	1.866267
O	9.784848	0.327959	1.069825	O	9.766947	0.094725	1.232400	H	9.003436	-1.843675	2.265592
H	10.336780	0.931359	0.571488	H	10.326366	0.746740	0.807491	O	9.757550	0.095541	1.234989
H	5.444847	-1.652647	2.357263	H	5.277862	-1.587957	2.476694	H	10.316244	0.752935	0.817481
H	3.133490	-1.851265	1.241877	H	3.008309	-1.730098	1.269232	H	3.000823	-1.736640	1.251659
C	3.985010	-1.457720	-1.404128	C	3.991310	-1.457608	-1.349842	C	3.983268	-1.433317	-1.364234
H	3.447391	-2.393420	-1.251551	H	3.407623	-2.364858	-1.194530	H	3.400544	-2.342886	-1.219209
H	3.721259	-1.055818	-2.382087	H	3.789094	-1.076125	-2.350635	H	3.780551	-1.040673	-2.360598
H	5.053014	-1.672265	-1.401762	H	5.047663	-1.717637	-1.292600	H	5.039898	-1.692877	-1.310053
H	-1.492576	-1.438385	0.436797	H	-1.457692	-1.315342	0.307946	H	-1.465707	-1.315627	0.295567
C	-1.425469	-2.249436	-1.545266	C	-1.473390	-2.041128	-1.706489	C	-1.480857	-2.018442	-1.727000
H	-2.321057	-2.864381	-1.472868	H	-2.331033	-2.696769	-1.564867	H	-2.337802	-2.676549	-1.592775
H	-1.338778	-1.900714	-2.577661	H	-1.512910	-1.650878	-2.727024	H	-1.520891	-1.616638	-2.743022
H	-0.571672	-2.892152	-1.335026	H	-0.578967	-2.655779	-1.616710	H	-0.585784	-2.633142	-1.644309
H	-13.115824	2.189983	1.671636	H	-13.167863	1.721746	2.020175	H	-13.178863	1.689538	2.043345
C	-0.246563	-0.161142	-0.742907	C	-0.224329	-0.011933	-0.852755	C	-0.233825	0.002202	-0.850329
H	-0.250369	0.276291	-1.747674	H	-0.205453	0.410862	-1.863383	H	-0.215495	0.436498	-1.856078
H	-0.336116	0.676006	-0.044642	H	-0.310144	0.836281	-0.167282	H	-0.320454	0.842466	-0.155234
**α-Tocopherol (C5$^{\bullet}$)**				**α-Tocopherol (C6$^{\bullet}$)**				**α-Tocopherol (C7$^{\bullet}$)**			
C	-3.600080	-0.247368	-0.346108	C	3.625035	-0.360219	-0.359666	C	3.619294	-0.362817	-0.342127
C	-5.849726	-0.038231	0.481117	C	5.833354	0.527992	-0.001971	C	5.827026	0.537869	-0.013157
C	-6.097061	1.089568	-0.297924	C	6.183727	-0.547635	0.811428	C	6.177499	-0.509911	0.835771
C	-4.958775	1.818783	-0.971281	C	5.133953	-1.528799	1.270073	C	5.127955	-1.475763	1.326332
C	-3.640744	1.208642	-0.665977	C	3.727041	-0.999845	1.022282	C	3.720920	-0.956027	1.060400
C	-6.887488	-0.709449	1.136933	C	6.784630	1.464040	-0.423139	C	6.778074	1.459831	-0.464807
C	-7.403809	1.560560	-0.448109	C	7.515854	-0.717287	1.200191	C	7.509514	-0.665887	1.230597
H	-5.135199	1.830078	-2.058509	H	5.273531	-2.483111	0.752425	H	5.268228	-2.446715	0.840811
H	-2.727125	1.752286	-0.851985	H	3.481938	-0.233006	1.760675	H	3.475101	-0.165146	1.772736
C	-8.430587	0.895191	0.208625	C	8.456161	0.215563	0.786145	C	8.449591	0.253111	0.786167
C	-8.189749	-0.227086	1.003619	C	8.108391	1.306045	-0.014415	C	8.101708	1.316167	-0.050422
O	-4.581322	-0.527793	0.673195	O	4.540422	0.744585	-0.413990	O	4.534196	0.740004	-0.432771
C	-2.273762	-0.657068	0.291190	C	2.260788	0.288347	-0.585402	C	2.254858	0.277220	-0.589978
H	-2.405599	-1.658259	0.708537	H	2.317647	0.855896	-1.518273	H	2.095091	1.031524	0.184601
H	-2.089694	0.015351	1.132560	H	2.101750	1.016517	0.213940	C	1.074976	-0.689840	-0.621448
C	-1.076133	-0.652784	-0.654497	C	1.080429	-0.676583	-0.648476	H	1.236323	-1.436661	-1.400206
H	-1.220025	-1.414957	-1.421225	H	1.241049	-1.397135	-1.451747	H	1.006922	-1.232144	0.325939
H	-1.008359	0.306041	-1.177452	H	1.012563	-1.250098	0.280363	C	-1.492452	-0.849916	-0.746870
C	1.487510	-0.878442	-0.791467	C	-1.487135	-0.831205	-0.777902	C	-2.755975	0.011699	-0.837257
C	2.740911	-0.868854	0.088872	C	-2.750287	0.033527	-0.838933	H	-2.789534	0.489258	-1.823146

H	2.737777	-1.764939	0.719786	H	-2.784084	0.543662	-1.808358	H	-2.676382	0.822617	-0.107121
H	2.678257	-0.015583	0.770765	H	-2.669960	0.819649	-0.082241	C	-4.062995	-0.739656	-0.597018
C	4.062514	-0.794085	-0.672746	C	-4.057553	-0.724805	-0.623204	H	-3.997780	-1.283080	0.352129
H	4.008310	0.012201	-1.412705	H	-3.992150	-1.299560	0.307291	H	-4.200412	-1.489671	-1.376114
H	4.214133	-1.718966	-1.230508	H	-4.195699	-1.448400	-1.426772	C	-5.273455	0.189877	-0.562975
C	5.250215	-0.546897	0.253704	C	-5.267552	0.203640	-0.557638	H	-5.076346	0.998597	0.147134
H	5.046857	0.350750	0.845349	H	-5.069719	0.988176	0.178906	H	-5.391753	0.665055	-1.543466
H	5.329706	-1.372791	0.969978	H	-5.386087	0.711254	-1.521707	C	-6.595042	-0.483180	-0.178063
C	6.599692	-0.372203	-0.450034	C	-6.589278	-0.481248	-0.194703	H	-6.458282	-0.948737	0.805301
H	6.487272	0.423869	-1.195823	H	-6.452275	-0.979352	0.772548	C	-7.694935	0.574909	-0.044617
C	7.654880	0.081585	0.563444	C	-7.688601	0.572320	-0.025563	H	-7.343229	1.357842	0.634006
H	7.280509	0.970785	1.079484	H	-7.336199	1.332062	0.678579	H	-7.840791	1.056496	-1.018194
H	7.763234	-0.693708	1.330748	H	-7.834687	1.086124	-0.982494	C	-9.036835	0.052623	0.462905
C	9.027909	0.399716	-0.022901	C	-9.030510	0.034047	0.464944	H	-8.880553	-0.503316	1.393965
H	8.914876	1.113724	-0.846113	H	-8.874054	-0.552656	1.376901	H	-9.450689	-0.654851	-0.256606
H	9.464028	-0.503824	-0.450569	H	-9.445044	-0.648886	-0.277521	C	-10.042161	1.174028	0.708854
C	9.978500	0.978473	1.021766	C	-10.035182	1.147107	0.748585	H	-9.588630	1.917700	1.371108
H	9.495607	1.834480	1.502716	H	-9.580981	1.868107	1.435012	H	-10.247488	1.689841	-0.236258
H	10.139865	0.235465	1.811410	H	-10.240711	1.694197	-0.178728	C	-11.371171	0.722471	1.319675
C	11.339860	1.424419	0.482685	C	-11.364120	0.676079	1.344683	H	-11.150340	0.222455	2.268711
H	11.163786	2.179597	-0.290596	H	-11.143078	0.144639	2.276437	C	-12.255717	1.932593	1.612001
C	12.162987	2.064842	1.598278	C	-12.247947	1.876206	1.677572	H	-11.751598	2.643474	2.267526
H	11.635372	2.905627	2.049886	H	-11.743177	2.564634	2.356155	H	-12.505781	2.451958	0.684097
H	12.363447	1.334138	2.385369	H	-12.498201	2.426293	0.767598	C	-6.983055	-1.575539	-1.173999
C	7.027740	-1.648215	-1.173942	C	-6.978287	-1.539661	-1.226267	H	-7.133452	-1.143104	-2.166914
H	7.175130	-2.457488	-0.453881	H	-7.128946	-1.074337	-2.204160	H	-6.212163	-2.340401	-1.254240
H	6.279486	-1.972502	-1.895542	H	-6.207800	-2.301784	-1.332307	H	-7.905241	-2.075398	-0.882208
H	7.961560	-1.507560	-1.716040	H	-7.900574	-2.048531	-0.950830	C	-12.110154	-0.266177	0.419277
C	12.115312	0.268253	-0.145876	C	-12.104003	-0.281700	0.412229	H	-12.271678	0.172242	-0.568882
H	12.251530	-0.534384	0.583411	H	-12.265784	0.189452	-0.560705	H	-11.557640	-1.195632	0.288972
H	11.602637	-0.146911	-1.012465	H	-11.551996	-1.206557	0.250780	H	-13.086109	-0.515566	0.836858
H	13.103741	0.595587	-0.469534	H	-13.079879	-0.544402	0.821753	C	9.182306	2.275422	-0.480600
C	-9.358893	-0.883179	1.693550	C	9.189244	2.278591	-0.412945	H	9.967641	1.764894	-1.045839
H	-10.084305	-1.269605	0.971634	H	9.974067	1.786803	-0.995252	H	8.795541	3.065849	-1.114884
H	-9.048429	-1.717935	2.312947	H	8.802557	3.089878	-1.020370	H	9.651772	2.755760	0.382330
H	-9.880668	-0.177024	2.345429	H	9.659349	2.729714	0.465271	C	6.361524	2.584728	-1.378503
C	-6.584835	-1.924831	1.976297	C	6.368186	2.618928	-1.298671	H	5.308037	2.507148	-1.623038
H	-5.533909	-2.183085	1.906799	H	5.314546	2.550021	-1.545137	H	6.529910	3.555552	-0.909677
H	-6.819738	-1.745428	3.026790	H	6.537259	3.573526	-0.797863	H	6.930798	2.566620	-2.308620
H	-7.171685	-2.784488	1.650913	H	6.937010	2.631535	-2.229153	C	7.915154	-1.802360	2.130192
C	-7.687579	2.776027	-1.288313	C	7.921375	-1.883270	2.061249	H	7.533114	-1.656653	3.142950
H	-7.226365	3.666252	-0.855583	H	7.539885	-1.771188	3.078484	H	7.514511	-2.749589	1.767713
H	-7.280558	2.660635	-2.293973	H	7.520107	-2.817720	1.667629	H	8.995920	-1.883547	2.187681
H	-8.755068	2.952786	-1.368045	H	9.002130	-1.966826	2.115474	O	9.746856	0.082598	1.206988
O	-9.702937	1.389348	0.052523	O	9.753543	0.030532	1.200421	H	10.304376	0.751178	0.806000
H	-10.324204	0.832338	0.523946	H	10.311193	0.711835	0.821643	H	5.262908	-1.654368	2.393886
H	-4.967382	2.871840	-0.675948	H	5.269326	-1.742911	2.331023	H	2.991784	-1.755620	1.185259
C	-3.935836	-1.100477	-1.574873	H	2.997581	-1.802814	1.120805	C	3.970248	-1.383230	-1.423194
H	-3.253162	-0.882060	-2.394876	C	3.974989	-1.344234	-1.474281	H	3.388536	-2.296810	-1.301144
H	-3.863828	-2.159353	-1.322385	H	3.392896	-2.261102	-1.382431	H	3.765771	-0.964904	-2.408689
H	-4.951867	-0.890882	-1.909087	H	5.031821	-1.606020	-1.437562	H	5.027184	-1.643141	-1.377279
H	1.477050	0.056651	-1.364152	H	-1.470904	-1.328037	0.199671	H	-1.476469	-1.313917	0.246710
C	1.505176	-2.043798	-1.780292	C	-1.489578	-1.909547	-1.861023	C	-1.493866	-1.963725	-1.793487
H	2.358133	-1.984552	-2.454815	H	-2.346009	-2.574894	-1.764517	H	-2.350036	-2.625900	-1.675288
H	1.567498	-2.993027	-1.241555	H	-1.531678	-1.448154	-2.851322	H	-1.535695	-1.535579	-2.798620
H	0.606877	-2.064810	-2.395554	H	-0.594109	-2.527620	-1.816628	H	-0.598133	-2.579547	-1.728115
H	13.122147	2.425751	1.225874	H	-13.181936	1.562157	2.144471	H	-13.189789	1.633816	2.088655
C	0.233925	-0.903947	0.088522	C	-0.241696	0.056117	-0.868248	C	-0.247380	0.034502	-0.866119
H	0.180668	-1.871140	0.600833	H	-0.225427	0.549363	-1.846472	H	-0.230861	0.494913	-1.860217
H	0.339295	-0.148276	0.872438	H	-0.327349	0.853589	-0.124335	H	-0.333766	0.856260	-0.149212
**α-Tocopherol (C8$^{\bullet}$)**				**α-Tocopherol (C9$^{\bullet}$)**				**α-Tocopherol (C10$^{\bullet}$)**			
C	3.510147	-0.979981	0.322208	C	3.608710	-0.363695	-0.343368	C	3.603446	-0.354320	-0.363584
C	5.651735	-0.050006	-0.239283	C	5.816484	0.535570	-0.010814	C	5.810915	0.528347	0.012603
C	5.279581	1.206562	0.234509	C	6.166485	-0.514934	0.834938	C	6.160860	-0.561554	0.806963
C	3.843066	1.489878	0.600447	C	5.116606	-1.482140	1.322102	C	5.111028	-1.551285	1.246676
C	2.909192	0.401135	0.086976	C	3.709752	-0.961327	1.057323	C	3.704149	-1.018618	1.006772
C	6.972439	-0.330082	-0.606397	C	6.767846	1.458780	-0.459240	C	6.762228	1.472249	-0.390567
C	6.246729	2.206664	0.376692	C	7.498337	-0.672381	1.229729	C	7.492614	-0.737544	1.194181
H	3.753932	1.580212	1.687597	H	5.256878	-2.451586	0.833582	H	5.251672	-2.496080	0.712136
H	2.756250	0.518352	-0.988491	H	3.463825	-0.172645	1.772056	H	3.457813	-0.265280	1.758531
C	7.555819	1.926910	0.009920	C	8.438726	0.247846	0.788510	C	8.432952	0.203029	0.798063
C	7.929856	0.676968	-0.487965	C	8.091313	1.313585	-0.044854	C	8.085587	1.307553	0.016810
O	4.745539	-1.071199	-0.398007	O	4.523833	0.739246	-0.430234	O	4.518360	0.751706	-0.396979
C	2.634899	-2.080794	-0.299558	C	2.244469	0.277354	-0.589674	C	2.239149	0.297563	-0.579238
H	3.187851	-3.019575	-0.190101	H	2.301940	0.816531	-1.539189	H	2.296821	0.881802	-1.501697

H	2.564267	-1.875266	-1.370253	H	2.084567	1.029248	0.187216	H	2.078826	1.011183	0.232867
C	1.271778	-2.195424	0.290517	C	1.064431	-0.689398	-0.624587	C	1.059332	-0.666626	-0.661020
H	1.166237	-2.561520	1.303436	H	1.225916	-1.433796	-1.405632	H	1.221239	-1.372559	-1.476914
C	-1.264527	-2.038692	0.267967	H	0.995958	-1.234663	0.321069	H	0.990661	-1.256763	0.257288
C	-2.420587	-1.342855	-0.454407	C	-1.502982	-0.848633	-0.751390	C	-1.508004	-0.820089	-0.796220
H	-2.495853	-1.747809	-1.470200	C	-2.766324	0.013481	-0.839502	C	-2.771503	0.045013	-0.843194
H	-2.177962	-0.281814	-0.565230	H	-2.799463	0.494141	-1.823898	H	-2.804416	0.572463	-1.803340
C	-3.776111	-1.464073	0.236675	H	-2.686842	0.822088	-0.106795	H	-2.692440	0.817454	-0.072411
H	-3.689109	-1.105840	1.268564	C	-4.073556	-0.738398	-0.602073	C	-4.078652	-0.717677	-0.642658
H	-4.066516	-2.513886	0.294620	H	-4.008761	-1.284813	0.345384	H	-4.014055	-1.309023	0.277440
C	-4.864132	-0.672554	-0.484777	H	-4.210836	-1.485937	-1.383568	H	-4.215509	-1.426786	-1.459255
H	-4.499043	0.342149	-0.669581	C	-5.283868	0.191234	-0.565524	C	-5.289176	0.208879	-0.561849
H	-5.038775	-1.118839	-1.470607	H	-5.086863	0.997684	0.147189	H	-5.092584	0.980151	0.188900
C	-6.200552	-0.579580	0.258174	H	-5.401748	0.669510	-1.544558	H	-5.406832	0.733673	-1.516783
H	-6.021429	-0.053089	1.203145	C	-6.605703	-0.482800	-0.183182	C	-6.610993	-0.483031	-0.212827
C	-7.192931	0.249089	-0.563608	H	-6.469360	-0.951468	0.798761	H	-6.474878	-0.998364	0.745481
H	-6.682752	1.142028	-0.937452	C	-7.705459	0.575055	-0.046789	C	-7.711023	0.566819	-0.026084
H	-7.483012	-0.328671	-1.448752	H	-7.353851	1.355792	0.634411	H	-7.359811	1.313954	0.692004
C	-8.447513	0.691196	0.185440	H	-7.850898	1.059724	-1.018898	H	-7.856242	1.097660	-0.973803
H	-8.155902	1.299894	1.048055	C	-9.047623	0.051410	0.458630	C	-9.053243	0.019200	0.453098
H	-8.971825	-0.179024	0.583331	H	-8.891756	-0.507478	1.387992	H	-8.897566	-0.583715	1.354553
C	-9.394419	1.492868	-0.703842	H	-9.461352	-0.653727	-0.263242	H	-9.466579	-0.650479	-0.302000
H	-8.824037	2.272783	-1.217620	C	-10.052840	1.172212	0.707756	C	-10.058785	1.126520	0.755505
H	-9.790924	0.837648	-1.487897	H	-9.599407	1.913721	1.372499	H	-9.605735	1.835285	1.455304
C	-10.567227	2.149393	0.028010	H	-10.257754	1.691028	-0.235801	H	-10.263496	1.690083	-0.162073
H	-10.153039	2.837864	0.772192	C	-11.382138	0.718970	1.316700	C	-11.388188	0.644245	1.341484
C	-11.414867	2.957339	-0.951708	H	-11.161718	0.215936	2.264236	H	-11.167977	0.096257	2.263801
H	-10.815909	3.704764	-1.473006	C	-12.266574	1.928320	1.612525	C	-12.272985	1.837789	1.694836
H	-11.854495	2.296979	-1.702850	H	-11.762558	2.637051	2.270451	H	-11.769342	2.514151	2.386267
C	-6.759958	-1.965044	0.579612	H	-12.516230	2.450641	0.686171	H	-12.522444	2.404016	0.794594
H	-6.871520	-2.546694	-0.339395	C	-6.993563	-1.571957	-1.182678	C	-6.998284	-1.522924	-1.263692
H	-6.102954	-2.518850	1.248823	H	-7.143544	-1.136379	-2.174281	H	-7.148028	-1.040181	-2.233246
H	-7.736167	-1.903541	1.058183	H	-6.222775	-2.336697	-1.265057	H	-6.227304	-2.282663	-1.382498
C	-11.434425	1.124928	0.756960	H	-7.915935	-2.072570	-0.892775	H	-7.920645	-2.037088	-0.998523
H	-11.811624	0.378839	0.052663	C	-12.120982	-0.266717	0.412948	C	-12.126518	-0.296974	0.391108
H	-10.882525	0.602739	1.537567	H	-12.282091	0.174833	-0.573884	H	-12.287392	0.191503	-0.573396
H	-12.294286	1.606643	1.223522	H	-11.568584	-1.195854	0.279912	H	-11.573875	-1.218526	0.213722
C	9.369496	0.459000	-0.879093	H	-13.097123	-0.517247	0.829409	H	-13.102744	-0.567442	0.794700
H	10.036489	0.536456	-0.015292	C	9.172224	2.274000	-0.471646	C	9.166434	2.287602	-0.362917
H	9.526068	-0.521191	-1.317036	H	9.957664	1.765114	-1.038217	H	9.952173	1.806716	-0.953041
H	9.694506	1.196033	-1.618452	H	8.785812	3.066483	-1.103576	H	8.780062	3.109488	-0.956126
C	7.336346	-1.698321	-1.124988	H	9.641477	2.751543	0.392950	H	9.635294	2.723095	0.523812
H	6.475529	-2.357249	-1.101879	C	6.351804	2.586615	-1.369539	C	6.346246	2.642484	-1.245702
H	7.694398	-1.645732	-2.154462	H	5.298387	2.509986	-1.614677	H	5.292928	2.577526	-1.494605
H	8.128318	-2.148523	-0.525269	H	6.520196	3.555932	-0.897607	H	6.514271	3.588011	-0.727632
C	5.874748	3.564913	0.907407	H	6.921393	2.571331	-2.299513	H	6.916149	2.672067	-2.175139
H	5.296719	4.128716	0.171758	C	7.903472	-1.811744	2.125887	C	7.897696	-1.918616	2.034634
H	5.260633	3.480006	1.804221	H	7.521110	-1.669154	3.138967	H	7.514964	-1.825001	3.053269
H	6.762038	4.141312	1.149303	H	7.502790	-2.757761	1.760297	H	7.497340	-2.846032	1.623822
O	8.478819	2.935475	0.148059	H	8.984205	-1.893299	2.183491	H	8.978427	-2.002632	2.088620
H	9.350080	2.605435	-0.076591	O	9.735817	0.075786	1.209237	O	9.729939	0.011189	1.210473
H	3.544398	2.457726	0.195510	H	10.293589	0.745527	0.810543	H	10.287700	0.699444	0.844649
H	1.934672	0.465752	0.571942	H	5.251162	-1.664121	2.389137	H	5.245266	-1.784356	2.303767
C	3.771467	-1.253490	1.800287	H	2.980435	-1.761181	1.179419	H	2.974964	-1.823566	1.090000
H	2.856294	-1.127118	2.378497	C	3.959859	-1.380767	-1.427515	C	3.955177	-1.317994	-1.495280
H	4.132288	-2.273785	1.928442	H	3.377947	-2.294626	-1.308535	H	3.373422	-2.236633	-1.420598
H	4.526600	-0.574680	2.194483	H	3.755792	-0.959312	-2.411761	H	3.751348	-0.849721	-2.458183
H	-1.193192	-1.615875	1.276895	H	5.016733	-1.641006	-1.382055	H	5.012092	-1.579907	-1.462028
C	-1.495023	-3.544725	0.387576	H	-1.487419	-1.315756	0.240733	H	-1.492672	-1.334393	0.172289
H	-2.364269	-3.775593	1.001412	C	-1.504228	-1.959147	-1.801502	C	-1.508660	-1.878804	-1.898536
H	-1.656306	-3.976569	-0.603599	H	-2.360553	-2.621542	-1.865677	H	-2.364881	-2.546174	-1.815004
H	-0.633195	-4.043343	0.830403	H	-1.545639	-1.527839	-2.805299	H	-0.612944	-2.497159	-1.864198
H	-12.229957	3.470875	-0.440923	H	-0.608624	-2.575328	-1.737758	H	-13.207366	1.514971	2.154920
C	0.057358	-1.751317	-0.450194	H	-13.200862	1.628210	2.087917	C	-0.262890	0.069290	-0.869150
H	0.033460	-2.228681	-1.440567	C	-0.257716	0.035940	-0.867434	H	-0.245719	0.580035	-1.838338
H	0.126388	-0.674069	-0.660808	H	-0.344206	0.855455	-0.147978	H	-0.349798	0.853231	-0.111135
**α-Tocopherol (C11$^{\bullet}$)**				**α-Tocopherol (C12$^{\bullet}$)**				**α-Tocopherol (C13$^{\bullet}$)**			
C	-3.578266	0.510767	-0.263335	C	-3.608448	-0.385835	0.261742	C	-3.579236	-0.162597	0.220444
C	-5.772116	-0.479702	-0.180176	C	-5.796226	0.582586	-0.004294	C	-5.885762	0.445119	-0.096980
C	-6.066604	0.161870	1.020804	C	-6.283017	-0.569882	-0.617076	C	-6.248255	-0.878130	-0.339607
C	-4.985401	0.890916	1.778873	C	-5.336701	-1.670868	-1.027673	C	-5.188899	-1.934210	-0.535498
C	-3.599382	0.527001	1.262350	C	-3.886037	-1.204741	-0.995890	C	-3.812782	-1.316487	-0.751021
C	-6.751109	-1.182975	-0.891323	C	-6.649168	1.628806	0.364730	C	-6.849103	1.446605	0.069463
C	-7.371485	0.128214	1.522102	C	-7.655439	-0.704481	-0.848138	C	-7.600990	-1.227231	-0.395010
H	-5.145015	1.970730	1.696731	H	-5.467327	-2.531633	-0.364164	H	-5.170445	-2.598043	0.334525

H	-3.323442	-0.471571	1.608186	H	-3.678006	-0.568180	-1.858978	H	-3.738225	-0.915090	-1.764108
C	-8.339513	-0.573496	0.817290	C	-8.498795	0.336168	-0.484860	C	-8.554272	-0.231624	-0.232338
C	-8.046238	-1.234844	-0.377385	C	-8.014784	1.501733	0.112901	C	-8.197555	1.099794	-0.008004
O	-4.508194	-0.484128	-0.718636	O	-4.459007	0.770274	0.246941	O	-4.572608	0.843803	-0.030658
C	-2.237512	0.022642	-0.807422	C	-2.200251	0.205232	0.256001	C	-2.261322	0.556696	-0.056529
H	-2.358118	-0.130041	-1.883572	H	-2.122591	0.874061	1.117632	H	-2.250601	1.462907	0.555393
H	-2.041045	-0.960670	-0.372640	H	-2.106649	0.831633	-0.634654	H	-2.272203	0.884213	-1.099240
C	-1.048964	0.944905	-0.556630	C	-1.060982	-0.808627	0.294118	C	-0.997724	-0.253237	0.217166
H	-1.244708	1.923938	-0.997295	H	-1.191747	-1.477129	1.146758	H	-0.995096	-0.589556	1.255316
H	-0.904068	1.101519	0.515346	H	-1.082832	-1.433905	-0.603141	H	-0.981428	-1.152481	-0.405807
C	1.499465	1.100952	-0.822671	C	1.502707	-1.056188	0.217152	C	1.570876	-0.223569	0.033059
C	2.772380	0.351413	-1.074469	C	2.774830	-0.269188	0.219868	C	2.759989	0.688776	-0.289810
H	2.911099	0.223853	-2.161695	H	2.728415	0.767796	-0.092883	H	2.843132	1.457073	0.488812
H	2.663399	-0.664481	-0.681501	C	4.114886	-0.914053	0.320104	H	2.537996	1.238096	-1.217728
C	4.045941	0.972770	-0.501701	H	4.220730	-1.687316	-0.457662	C	4.060349	-0.025496	-0.421772
H	3.903681	1.174318	0.564894	H	4.191839	-1.454102	1.269130	H	4.051864	-1.018686	-0.860150
H	4.227345	1.935300	-0.981742	C	5.266865	0.081828	0.198946	C	5.357437	0.705677	-0.377977
C	5.255408	0.062574	-0.694224	H	5.104401	0.697429	-0.690379	H	5.510022	1.253070	-1.320892
H	5.018449	-0.924839	-0.286586	H	5.238238	0.763827	1.055435	H	5.316639	1.475965	0.402136
H	5.428646	-0.084209	-1.766576	C	6.659993	-0.547781	0.109503	C	6.576772	-0.191179	-0.132451
C	6.555213	0.554516	-0.049707	H	6.682897	-1.183124	-0.784006	H	6.562879	-0.984143	-0.889640
H	6.368017	0.691785	1.021916	C	7.709704	0.553285	-0.070224	C	7.867794	0.609883	-0.313231
C	7.638282	-0.518034	-0.203307	H	7.409491	1.187239	-0.910061	H	7.858278	1.071179	-1.305417
H	7.248720	-1.462501	0.188466	H	7.698978	1.196323	0.817160	H	7.874390	1.435281	0.408226
H	7.820207	-0.683132	-1.271572	C	9.133968	0.060872	-0.313562	C	9.151866	-0.200103	-0.159520
C	8.964879	-0.212766	0.487761	H	9.139570	-0.632331	-1.161785	H	9.115815	-1.066925	-0.828958
H	8.778813	0.045202	1.536144	H	9.479461	-0.503935	0.553195	H	9.216328	-0.594347	0.855603
H	9.426482	0.661823	0.027825	C	10.102820	1.207271	-0.590471	C	10.399445	0.623912	-0.466315
C	9.929310	-1.393635	0.420967	H	9.704763	1.819131	-1.405700	H	10.271412	1.113648	-1.436427
H	9.429131	-2.280620	0.821532	H	10.147075	1.860674	0.288481	H	10.492415	1.427785	0.273001
H	10.153200	-1.617040	-0.628622	C	11.526203	0.776194	-0.952236	C	11.705966	-0.173392	-0.491519
C	11.248968	-1.196430	1.171293	H	11.469244	0.149563	-1.848560	H	11.607928	-0.947538	-1.259921
H	11.010948	-0.987387	2.219649	C	12.379415	2.000242	-1.277953	C	12.871621	0.735554	-0.873936
C	12.081693	-2.475272	1.115257	H	11.937787	2.587470	-2.083742	H	12.696630	1.223559	-1.833373
H	11.534849	-3.323818	1.527645	H	12.463631	2.645744	-0.400569	H	13.005775	1.515595	-0.120862
H	12.338680	-2.713465	0.080385	C	6.963479	-1.423968	1.324495	C	6.495856	-0.841828	1.247655
C	7.002494	1.894421	-0.632040	H	6.942133	-0.823718	2.238054	H	6.603423	-0.082457	2.026805
H	7.200176	1.792539	-1.702508	H	6.236244	-2.227265	1.433096	H	5.532845	-1.332071	1.390006
H	6.242231	2.663132	-0.502133	H	7.946253	-1.886431	1.248562	H	7.276468	-1.587107	1.392839
H	7.912447	2.255330	-0.155015	C	12.178361	-0.041679	0.161286	C	11.984967	-0.860073	0.844246
C	12.052005	-0.019716	0.619655	H	12.179637	0.525957	1.095368	H	12.010862	-0.122442	1.650436
H	12.245374	-0.162161	-0.446660	H	11.657317	-0.981434	0.338959	H	11.224370	-1.600334	1.089093
H	11.529541	0.927740	0.743596	H	13.213854	-0.277285	-0.086558	H	12.949759	-1.367802	0.825275
H	13.015012	0.063169	1.124403	C	-8.997777	2.586874	0.472255	C	-9.295649	2.120840	0.150938
C	-9.152335	-1.986641	-1.073579	H	-9.739752	2.229704	1.192575	H	-9.923746	1.898691	1.018626
H	-9.960489	-1.316531	-1.381357	H	-8.509165	3.446492	0.918490	H	-8.901918	3.122167	0.288897
H	-8.800001	-2.492458	-1.966214	H	-9.535216	2.943328	-0.410933	H	-9.941872	2.147856	-0.730842
H	-9.582898	-2.749985	-0.419705	C	-6.086066	2.865678	1.018195	C	-6.420061	2.869553	0.323820
C	-6.393299	-1.871641	-2.184261	H	-5.013033	2.775949	1.146126	H	-5.339738	2.936982	0.388725
H	-5.358328	-1.680127	-2.445022	H	-6.283259	3.753289	0.415197	H	-6.752746	3.530196	-0.478322
H	-6.527556	-2.951721	-2.105132	H	-6.534354	3.029571	1.999193	H	-6.842210	3.247345	1.256017
H	-7.022685	-1.522100	-3.003491	C	-8.209357	-1.948778	-1.488661	C	-8.016699	-2.653202	-0.637121
C	-7.718066	0.829389	2.807879	H	-7.970115	-1.982098	-2.554035	H	-7.822852	-2.947679	-1.671129
H	-7.267257	0.323326	3.664403	H	-7.786193	-2.844591	-1.033968	H	-7.458648	-3.336919	0.002946
H	-7.346190	1.854440	2.807007	H	-9.289433	-1.986637	-1.387708	H	-9.076550	-2.785649	-0.444592
H	-8.792480	0.851648	2.958760	O	-9.839700	0.179969	-0.742492	O	-9.874712	-0.605245	-0.305725
O	-9.607982	-0.602296	1.345777	H	-10.320664	0.938371	-0.407849	H	-10.433186	0.157083	-0.145881
H	-10.194539	-1.066890	0.746974	H	-5.592874	-2.022305	-2.027909	H	-5.451325	-2.564405	-1.386365
H	-5.059911	0.657865	2.841732	H	-3.212663	-2.060037	-1.043015	H	-3.035281	-2.070757	-0.633438
H	-2.856405	1.228044	1.641367	C	-3.882499	-1.182087	1.536207	C	-3.686240	-0.607969	1.677589
C	-3.972404	1.861644	-0.857710	H	-3.350510	-2.133087	1.523766	H	-3.045010	-1.467764	1.870715
H	-3.382560	2.666705	-0.419252	H	-3.561307	-0.610965	2.407130	H	-3.389858	0.206866	2.337953
H	-3.811299	1.855345	-1.935626	H	-4.947251	-1.387097	1.639080	H	-4.711514	-0.885346	1.919393
H	-5.025488	2.070126	-0.673381	H	1.400159	-1.544416	-0.766480	H	1.541818	-1.013671	-0.726585
C	1.505655	2.593187	-0.882639	C	1.518215	-2.169319	1.270318	C	1.749184	-0.878471	1.401992
H	2.364650	3.015851	-0.363488	H	2.365034	-2.838699	1.125494	H	2.724099	-1.359325	1.480041
H	1.553236	2.950250	-1.922824	H	1.594039	-1.739229	2.271715	H	1.683695	-0.123851	2.190472
H	0.606482	3.020481	-0.441267	H	0.612815	-2.772241	1.222771	H	0.986828	-1.632665	1.592805
H	13.012372	-2.366879	1.672898	H	13.387078	1.712629	-1.578870	H	13.804563	0.175387	-0.942608
C	0.236533	0.362956	-1.147474	C	0.299259	-0.121662	0.386841	C	0.262541	0.564392	-0.056909
H	0.116564	0.304038	-2.242336	H	0.385221	0.387961	1.351938	H	0.305942	1.401921	0.648540
H	0.340954	-0.674373	-0.814827	H	0.357357	0.657312	-0.379024	H	0.186145	1.007708	-1.054174
**α-Tocopherol (C14$^{\bullet}$)**				**α-Tocopherol (C15$^{\bullet}$)**				**α-Tocopherol (C16$^{\bullet}$)**			
C	3.538286	-0.531889	-0.180966	C	-3.593370	-0.330572	0.326046	C	3.555543	-0.418522	-0.279116
C	5.699641	0.529646	-0.188270	C	-5.801022	0.545791	-0.062017	C	5.746219	0.542249	-0.010308

C	6.061178	-0.094962	1.003365	C	-6.214184	-0.639381	-0.667142	C	6.124079	-0.454530	0.886475
C	5.034154	-0.852158	1.807621	C	-5.207849	-1.709310	-1.010568	C	5.097801	-1.417102	1.430979
C	3.618700	-0.534871	1.342832	C	-3.780182	-1.183966	-0.924873	C	3.678303	-0.939633	1.149858
C	6.627494	1.258036	-0.941399	C	-6.710610	1.563973	0.244636	C	6.674019	1.461904	-0.513116
C	7.383038	-0.017096	1.452592	C	-7.569168	-0.836718	-0.950002	C	7.461762	-0.562637	1.278785
H	5.224090	-1.927040	1.725540	H	-5.334144	-2.560475	-0.333978	H	5.255538	-2.407117	0.991338
H	3.326377	0.458157	1.691209	H	-3.559880	-0.555434	-1.790700	H	3.420995	-0.119349	1.823640
C	8.300281	0.709422	0.706168	C	-8.469296	0.174140	-0.643851	C	8.379016	0.353304	0.782219
C	7.940288	1.351470	-0.480653	C	-8.058642	1.373275	-0.057510	C	8.002606	1.367688	-0.100785
O	4.416047	0.490336	-0.677175	O	-4.484523	0.792395	0.243160	O	4.447542	0.696432	-0.430689
C	2.160462	-0.095375	-0.674080	C	-2.209009	0.312115	0.375127	C	2.179264	0.186271	-0.547495
H	2.236919	0.072330	-1.751756	H	-2.207359	1.021562	1.207154	H	2.229483	0.691469	-1.516030
H	1.936401	0.874041	-0.221745	H	-2.081714	0.900007	-0.537414	H	2.005689	0.963306	0.201200
C	1.022672	-1.073164	-0.394040	C	-1.039040	-0.654920	0.532558	C	1.014033	-0.798384	-0.551732
H	1.233317	-2.025703	-0.882630	H	-1.185778	-1.266425	1.424503	H	1.194702	-1.574901	-1.296522
H	0.952992	-1.276958	0.678496	H	-0.999016	-1.341345	-0.318185	H	0.936452	-1.301033	0.416945
C	-1.536053	-1.382506	-0.520223	C	1.535202	-0.805760	0.593195	C	-1.555171	-0.977324	-0.725772
C	-2.817214	-0.644917	-0.920188	C	2.796757	0.060674	0.659317	C	-2.807468	-0.137242	-0.995713
H	-2.821412	-0.501852	-2.006356	H	2.841354	0.543224	1.642310	H	-2.801983	0.176022	-2.046198
H	-2.799933	0.355218	-0.479108	H	2.703866	0.868408	-0.073226	H	-2.746705	0.780967	-0.403739
C	-4.117440	-1.334442	-0.502878	C	4.105102	-0.681632	0.402197	C	-4.131120	-0.828643	-0.681522
H	-4.079509	-1.576411	0.566163	H	4.042390	-1.202270	-0.559262	H	-4.126872	-1.168094	0.359143
H	-4.197941	-2.299843	-1.020121	H	4.262492	-1.450935	1.160548	H	-4.241974	-1.719124	-1.301579
C	-5.324620	-0.505336	-0.779334	C	5.309913	0.253682	0.389185	C	-5.326488	0.097755	-0.905259
H	-5.342368	0.089598	-1.686117	H	5.150054	1.040610	-0.353465	H	-5.140821	1.049150	-0.396546
C	-6.599522	-0.702280	-0.025088	H	5.392174	0.754599	1.360016	H	-5.370222	0.350830	-1.978304
H	-6.369253	-0.642417	1.047802	C	6.643057	-0.448984	0.087320	C	-6.657254	-0.430705	-0.463824
C	-7.607564	0.401804	-0.356224	H	6.547942	-0.955664	-0.878691	C	-7.769476	0.570931	-0.395097
H	-7.122704	1.369970	-0.202401	C	7.766780	0.596993	-0.040548	H	-7.380079	1.495126	0.043544
H	-7.850624	0.344758	-1.423082	H	7.457359	1.355956	-0.765343	H	-8.079852	0.846973	-1.417092
C	-8.896199	0.354136	0.458354	H	7.875393	1.109813	0.921117	C	-9.011887	0.135382	0.381920
H	-8.649956	0.342712	1.526003	C	9.113784	0.017355	-0.458991	H	-8.708961	-0.241791	1.364038
H	-9.426740	-0.576502	0.251614	H	9.000024	-0.513214	-1.410591	H	-9.494774	-0.694195	-0.136754
C	-9.815218	1.536990	0.164721	H	9.425884	-0.726855	0.276502	C	-10.009873	1.275478	0.561031
H	-9.244269	2.464962	0.265269	C	10.187359	1.091862	-0.603958	H	-9.495465	2.128386	1.014058
H	-10.140757	1.490066	-0.880972	H	9.814649	1.877233	-1.268747	H	-10.357058	1.613758	-0.422150
C	-11.053254	1.626301	1.060432	H	10.354350	1.568254	0.368962	C	-11.228381	0.928808	1.419587
H	-10.709374	1.716037	2.096281	C	11.530722	0.592816	-1.141844	H	-10.864374	0.605761	2.400618
C	-11.864927	2.873077	0.715839	H	11.352184	0.143858	-2.124717	C	-12.104042	2.163927	1.617981
H	-11.256917	3.775668	0.786521	C	12.495633	1.762971	-1.318734	H	-11.540471	2.981720	2.068344
H	-12.245865	2.805126	-0.305792	H	12.081133	2.522630	-1.982293	H	-12.490957	2.512790	0.657699
C	-7.178425	-2.104954	-0.282802	H	12.698092	2.234504	-0.354253	C	-6.982358	-1.869466	-0.698510
H	-7.504924	-2.190631	-1.321450	C	6.964869	-1.464937	1.132323	H	-7.212383	-2.059464	-1.758126
H	-6.428093	-2.873823	-0.099723	H	6.999321	-1.162599	2.171125	H	-6.149783	-2.520285	-0.434145
H	-8.030513	-2.312248	0.363901	H	7.237428	-2.480213	0.891437	H	-7.849954	-2.188201	-0.122812
C	-11.929248	0.379276	0.954326	C	12.149174	-0.471287	-0.236781	C	-12.049457	-0.210113	0.816800
H	-12.220684	0.209764	-0.085445	H	12.268149	-0.081233	0.777262	H	-12.359037	0.044901	-0.200004
H	-11.415177	-0.513089	1.308997	H	11.534113	-1.368540	-0.182636	H	-11.487385	-1.142033	0.773835
H	-12.840950	0.494282	1.541338	H	13.134788	-0.764246	-0.599538	H	-12.950289	-0.392600	1.403482
C	8.995408	2.127535	-1.227352	C	-9.099946	2.423177	0.236686	C	9.058658	2.331023	-0.579836
H	9.827156	1.483781	-1.528359	H	-9.843932	2.057685	0.950842	H	9.842944	1.817321	-1.143731
H	8.602386	2.581818	-2.130635	H	-8.663895	3.320285	0.663034	H	8.646596	3.095610	-1.229719
H	9.403879	2.933805	-0.611897	H	-9.629239	2.722060	-0.672223	H	9.534611	2.844991	0.259782
C	6.196887	1.928138	-2.221774	C	-6.225324	2.838965	0.886951	C	6.226401	2.532517	-1.476089
H	5.153317	1.719416	-2.429546	H	-5.152935	2.804490	1.042880	H	5.177693	2.408481	-1.722275
H	6.319873	3.010566	-2.159918	H	-6.449748	3.704245	0.261563	H	6.357943	3.527259	-1.046977
H	6.789892	1.578799	-3.068017	H	-6.705227	2.999747	1.853492	H	6.802091	2.495643	-2.401602
C	7.801865	-0.697166	2.728111	C	-8.043363	-2.116507	-1.584223	C	7.897406	-1.644778	2.229720
H	7.374030	-0.195390	3.598827	H	-7.754800	-2.162723	-2.636870	H	7.517551	-1.458737	3.236726
H	7.458106	-1.731786	2.751188	H	-7.604721	-2.983816	-1.090562	H	7.516178	-2.617059	1.915914
H	8.881885	-0.689063	2.834052	H	-9.124462	-2.197074	-1.530231	H	8.979987	-1.698905	2.284360
O	9.586569	0.782147	1.184777	O	-9.791353	-0.045993	-0.948118	O	9.682464	0.230781	1.200637
H	10.131809	1.270307	0.565937	H	-10.319487	0.689312	-0.633523	H	10.225155	0.883944	0.756396
H	5.142570	-0.609557	2.865411	H	-5.403965	-2.092172	-2.012997	H	5.242684	-1.541117	2.504940
H	2.912437	-1.254215	1.755914	H	-3.071349	-2.011322	-0.925547	H	2.964875	-1.745573	1.318498
C	3.954399	-1.873350	-0.781534	C	-3.900359	-1.109643	1.603774	C	3.918003	-1.483151	-1.312493
H	3.411916	-2.694228	-0.312224	H	-3.342699	-2.045475	1.632346	H	3.360095	-2.402974	-1.137288
H	3.747706	-1.882371	-1.851669	H	-3.631671	-0.513897	2.476013	H	3.691438	-1.120353	-2.315011
H	5.020944	-2.043456	-0.640505	H	-4.962605	-1.342226	1.665228	H	4.981374	-1.715040	-1.267927
H	-1.547811	-1.506038	0.569305	H	1.538068	-1.330015	-0.369901	H	-1.608111	-1.335656	0.309161
C	-1.458205	-2.769121	-1.157855	C	1.513367	-1.852129	1.706941	C	-1.479568	-2.192410	-1.649762
H	-2.306569	-3.390058	-0.874207	H	2.409572	-2.470309	1.695269	H	-2.376586	-2.805818	-1.582994
H	-1.453196	-2.682122	-2.247560	H	1.454628	-1.361653	2.682373	H	-1.368579	-1.868987	-2.688130
H	-0.555358	-3.299446	-0.858514	H	0.657586	-2.519716	1.615662	H	-0.631405	-2.829790	-1.404132
H	-12.719511	2.985084	1.383606	H	13.447681	1.431302	-1.734157	H	-12.957010	1.944646	2.260852

C	-0.317930	-0.526479	-0.880518	C	0.291793	0.087327	0.634865	C	-0.308475	-0.096496	-0.853589
H	-0.283323	-0.400397	-1.968454	H	0.306228	0.671594	1.561669	H	-0.271204	0.317106	-1.867713
H	-0.458819	0.473929	-0.461079	H	0.355840	0.811027	-0.183069	H	-0.414394	0.757734	-0.178612
**α-Tocopherol (C17•)**				**α-Tocopherol (C18•)**				**α-Tocopherol (C19•)**			
C	-3.587212	-0.348671	0.304709	C	-3.574629	-0.281857	0.393244	C	3.549927	-0.466699	-0.156548
C	-5.783879	0.579388	-0.019385	C	-5.796632	0.510407	-0.086527	C	5.752268	0.501310	-0.136171
C	-6.231786	-0.581010	-0.646899	C	-6.180837	-0.732747	-0.585081	C	6.109531	-0.219889	1.000741
C	-5.253504	-1.661542	-1.036149	C	-5.151493	-1.809085	-0.822929	C	5.064625	-0.984968	1.774507
C	-3.813601	-1.167653	-0.962946	C	-3.735423	-1.249313	-0.775455	C	3.654856	-0.580941	1.361006
C	-6.666716	1.606936	0.330808	C	-6.729400	1.533412	0.118769	C	6.696037	1.240127	-0.857882
C	-7.595223	-0.743304	-0.910870	C	-7.528472	-0.981431	-0.862908	C	7.442867	-0.235044	1.421894
H	-5.385138	-2.527626	-0.379800	H	-5.269388	-2.599514	-0.074960	H	5.208561	-2.058758	1.618177
H	-3.595085	-0.522974	-1.817299	H	-3.520886	-0.698424	-1.693957	H	3.405603	0.394049	1.785418
C	-8.468655	0.277760	-0.563666	C	-8.450815	0.035726	-0.661306	C	8.376386	0.502034	0.706110
C	-8.023308	1.451756	0.047525	C	-8.069335	1.291194	-0.183005	C	8.020717	1.243914	-0.422046
O	-4.457375	0.792380	0.266504	O	-4.488279	0.810659	0.205064	O	4.458628	0.554608	-0.594845
C	-2.189830	0.266213	0.343059	C	-2.204382	0.392229	0.388027	C	2.183315	0.057068	-0.592791
H	-2.155923	0.947538	1.197614	H	-2.222779	1.176363	1.150131	H	2.254960	0.307527	-1.654637
H	-2.071884	0.881845	-0.552275	H	-2.085018	0.895219	-0.574844	H	2.001740	0.994819	-0.061842
C	-1.035978	-0.727503	0.441316	C	-1.015700	-0.530970	0.637101	C	1.010906	-0.894832	-0.376229
H	-1.179893	-1.371320	1.310506	H	-1.149982	-1.054818	1.584970	H	1.178497	-1.812307	-0.941502
H	-1.024038	-1.379968	-0.436494	H	-0.961979	-1.296940	-0.142220	H	0.942402	-1.178895	0.678193
C	1.535798	-0.930371	0.460229	C	1.559791	-0.632654	0.662632	C	-1.558314	-1.101839	-0.543752
C	2.813637	-0.086613	0.508504	C	2.809416	0.252887	0.633917	C	-2.811418	-0.270269	-0.836566
H	2.871744	0.407791	1.484885	H	2.871662	0.805737	1.578199	H	-2.802414	0.021313	-1.892867
H	2.731664	0.712778	-0.234097	H	2.687986	1.004573	-0.151911	H	-2.751131	0.659893	-0.263434
C	4.108396	-0.855740	0.259093	C	4.120792	-0.491756	0.397317	C	-4.135187	-0.959730	-0.516836
H	4.036705	-1.387288	-0.696155	H	4.044506	-1.070712	-0.529511	H	-4.103285	-1.344926	0.508138
H	4.237053	-1.615584	1.030251	H	4.287276	-1.210767	1.200137	H	-4.267153	-1.822446	-1.170758
C	5.328208	0.060776	0.240180	C	5.316327	0.452505	0.303443	C	-5.325238	-0.015322	-0.662424
H	5.149205	0.875458	-0.467673	H	5.091553	1.236287	-0.426304	H	-5.131696	0.885183	-0.072438
H	5.447695	0.527634	1.223601	H	5.452037	0.958770	1.265984	H	-5.401834	0.312037	-1.705679
C	6.647316	-0.624132	-0.136766	C	6.634195	-0.218652	-0.090004	C	-6.674888	-0.595558	-0.227734
H	6.506873	-1.083901	-1.129269	H	6.492729	-0.689509	-1.069871	H	-6.573367	-0.932310	0.810674
C	7.744602	0.387182	-0.239872	C	7.740377	0.832844	-0.235473	C	-7.741421	0.502964	-0.263280
H	7.470741	1.414520	-0.451563	H	7.363319	1.653765	-0.864917	H	-7.388220	1.354377	0.324208
C	9.179088	0.001279	-0.363205	H	7.934028	1.285756	0.744888	H	-7.849619	0.861767	-1.293103
H	9.301049	-0.736880	-1.171722	C	9.013206	0.307939	-0.803239	C	-9.115724	0.088284	0.264923
H	9.501932	-0.518684	0.545415	H	8.949267	-0.480937	-1.546517	H	-9.006646	-0.373557	1.253522
C	10.097526	1.192593	-0.624832	C	10.274894	1.095797	-0.732528	H	-9.532599	-0.695867	-0.381808
H	9.702682	1.762120	-1.471045	H	10.220119	1.952087	-1.422734	C	-10.067401	1.231517	0.356653
H	10.065787	1.863474	0.240576	H	10.378406	1.536560	0.266958	H	-10.024941	1.999913	-0.407126
C	11.554506	0.827687	-0.916097	C	11.537828	0.290087	-1.053829	C	-11.254223	1.188256	1.263233
H	11.568478	0.198605	-1.812466	H	11.399809	-0.163835	-2.040690	H	-10.897008	0.942539	2.271026
C	12.363661	2.089806	-1.205353	C	12.757889	1.204956	-1.111392	C	-11.961969	2.539948	1.318640
H	11.929113	2.658697	-2.027981	H	12.631070	1.989615	-1.857983	H	-11.278936	3.325899	1.641136
H	12.383501	2.735976	-0.324710	H	12.916091	1.685234	-0.142962	H	-12.340980	2.807313	0.330307
C	7.006926	-1.755068	0.833427	C	7.047408	-1.298636	0.908573	C	-7.073541	-1.797768	-1.082236
H	7.117078	-1.360980	1.846297	H	7.122281	-0.871974	1.912359	H	-7.206848	-1.490478	-2.122799
H	6.237867	-2.525580	0.845467	H	6.330490	-2.117728	0.942762	H	-6.315638	-2.579459	-1.056277
H	7.944879	-2.232361	0.552781	H	8.021280	-1.714795	0.650611	H	-8.007536	-2.242314	-0.741710
C	12.190162	0.040607	0.228644	C	11.749214	-0.826984	-0.034599	C	-12.236424	0.079931	0.846604
H	12.121873	0.606473	1.161267	H	11.885827	-0.402427	0.963041	H	-12.628223	0.283921	-0.151752
H	11.704876	-0.922364	0.382762	H	10.892043	-1.499028	0.003042	H	-11.751006	-0.895706	0.826882
H	13.245551	-0.147654	0.029322	H	12.636083	-1.414519	-0.273903	H	-13.076487	0.029430	1.540344
C	-9.036929	2.514478	0.388329	C	-9.132253	2.346054	-0.006683	C	9.095181	2.029036	-1.130781
H	-9.781776	2.142040	1.097911	H	-9.884671	2.036621	0.724793	H	9.875621	1.372199	-1.525970
H	-8.575408	3.386062	0.840127	H	-8.717785	3.286903	0.339495	H	8.700438	2.595040	-1.967958
H	-9.568439	2.856524	-0.504045	H	-9.647372	2.549130	-0.949571	H	9.572644	2.743741	-0.454893
C	-6.144500	2.853982	0.998563	C	-6.276052	2.869493	0.651067	C	6.268077	2.019950	-2.075382
H	-5.071294	2.791355	1.139972	H	-5.216942	2.848394	0.882768	H	5.210041	1.877893	-2.266236
H	-6.356868	3.739620	0.397920	H	-6.446424	3.662089	-0.079511	H	6.447858	3.087716	-1.941039
H	-6.609088	3.000896	1.974738	H	-6.819640	3.136942	1.557897	H	6.820100	1.702933	-2.961085
C	-8.106959	-1.996451	-1.568758	C	-7.969314	-2.323040	-1.383481	C	7.855425	-1.018586	2.639038
H	-7.836400	-2.022741	-2.626861	H	-7.641136	-2.470087	-2.414873	H	7.495692	-0.540806	3.553056
H	-7.679707	-2.885009	-1.103723	H	-7.541627	-3.132266	-0.790926	H	7.438987	-2.025963	2.616375
H	-9.188550	-2.055198	-1.498899	H	-9.050745	-2.411244	-1.360028	H	8.936264	-1.092993	2.703934
O	-9.799902	0.093520	-0.851312	O	-9.764383	-0.236809	-0.959014	O	9.674462	0.488832	1.157571
H	-10.305511	0.836189	-0.517693	H	-10.306715	0.524081	-0.745155	H	10.224164	1.007663	0.568065
H	-5.475444	-2.013511	-2.044455	H	-5.330985	-2.283068	-1.788832	H	5.201386	-0.816264	2.843358
H	-3.122660	-2.009437	-0.996028	H	-3.009759	-2.057800	-0.694699	H	2.927393	-1.302273	1.732429
C	-3.885229	-1.153234	1.568699	C	-3.874568	-0.948374	1.734528	C	3.907322	-1.776545	-0.855905
H	-3.340606	-2.097133	1.567066	H	-3.303442	-1.869592	1.848233	H	3.345506	-2.609341	-0.433000
H	-3.594677	-0.581400	2.449883	H	-3.619348	-0.273618	2.551408	H	3.681979	-1.705864	-1.920155
H	-4.949556	-1.372418	1.641995	H	-4.933557	-1.191266	1.812453	H	4.969915	-1.991129	-0.748442

H	1.504263	-1.438062	-0.511261	H	1.551270	-1.230020	-0.256866	H	-1.572536	-1.364383	0.520617
C	1.521280	-1.995204	1.556517	C	1.574159	-1.590397	1.853619	C	-1.540173	-2.395856	-1.354970
H	2.386491	-2.652892	1.491548	H	2.471067	-2.207633	1.863287	H	-2.407020	-3.017864	-1.136879
H	1.531934	-1.520977	2.541706	H	1.541809	-1.027005	2.789936	H	-1.548220	-2.168040	-2.424130
H	0.633308	-2.622879	1.496245	H	0.717920	-2.263363	1.837175	H	-0.652312	-2.990195	-1.143934
H	13.394681	1.848404	-1.465214	H	13.660369	0.644689	-1.356857	H	-12.806754	2.515306	2.006917
C	0.310750	-0.015231	0.548748	C	0.302280	0.241565	0.660231	C	-0.308589	-0.254787	-0.802748
H	0.349947	0.537572	1.493908	H	0.321584	0.895170	1.539402	H	-0.262482	-0.010934	-1.870205
H	0.377910	0.733738	-0.245894	H	0.338783	0.900895	-0.211902	H	-0.417159	0.698847	-0.277347
**α-Tocopherol (C20$^{\bullet}$)**				**α-Tocopherol (C21$^{\bullet}$)**				**α-Tocopherol (C22$^{\bullet}$)**			
C	3.560814	-0.336600	-0.303873	C	3.560491	-0.344248	-0.300001	C	3.562335	-0.355950	-0.362327
C	5.771666	0.545057	0.047823	C	5.768969	0.549192	0.036900	C	5.764546	0.547128	0.010611
C	6.158622	-0.589165	0.759150	C	6.168620	-0.585244	0.740632	C	6.135164	-0.556588	0.777000
C	5.132056	-1.612237	1.177367	C	5.151656	-1.616077	1.163497	C	5.100853	-1.567736	1.205036
C	3.714078	-1.077154	1.021766	C	3.729020	-1.089314	1.021269	C	3.686029	-1.046243	0.993038
C	6.699639	1.522869	-0.326767	C	6.687671	1.534340	-0.341402	C	6.701686	1.511854	-0.377401
C	7.505837	-0.778074	1.081456	C	7.519591	-0.766952	1.051303	C	7.472316	-0.724890	1.149225
H	5.255641	-2.521637	0.580911	H	5.275970	-2.522644	0.562893	H	5.246338	-2.499607	0.649581
H	3.491910	-0.369443	1.823585	H	3.508579	-0.386430	1.827801	H	3.441261	-0.309280	1.761486
C	8.424178	0.193165	0.708730	C	8.428796	0.211458	0.674807	C	8.398519	0.235933	0.767389
C	8.039077	1.344180	0.017659	C	8.030991	1.362558	-0.008817	C	8.030648	1.354436	0.015495
O	4.464005	0.779368	-0.302401	O	4.457145	0.776866	-0.301589	O	4.465277	0.761423	-0.384555
C	2.184417	0.311179	-0.438680	C	2.179710	0.296812	-0.420815	C	2.189310	0.287475	-0.547206
H	2.210305	0.953022	-1.323547	H	2.194275	0.939773	-1.305123	H	2.223821	0.876395	-1.467938
H	2.038101	0.970236	0.420891	H	2.038754	0.954311	0.440838	H	2.043300	0.997166	0.270918
C	1.011992	-0.659138	-0.552388	C	1.010281	-0.678218	-0.524232	C	1.014569	-0.684751	-0.610151
H	1.171281	-1.325285	-1.401702	H	1.166102	-1.346322	-1.372675	H	1.183654	-1.408450	-1.408755
H	0.957232	-1.291558	0.338490	H	0.963422	-1.308176	0.368810	H	0.943715	-1.251520	0.322012
C	-1.558352	-0.817872	-0.674091	C	-1.561200	-0.843072	-0.628550	C	-1.552398	-0.851826	-0.706230
C	-2.821598	0.044936	-0.759297	C	-2.825646	0.018011	-0.712925	C	-2.819749	0.003121	-0.800155
H	-2.843789	0.543580	-1.735022	H	-2.852057	0.511566	-1.691132	H	-2.868077	0.461281	-1.794151
H	-2.750453	0.840446	-0.011411	H	-2.752070	0.817503	0.030462	H	-2.730562	0.829366	-0.088102
C	-4.131984	-0.709524	-0.549386	C	-4.135244	-0.735352	-0.494182	C	-4.121114	-0.743751	-0.523148
H	-4.084468	-1.259615	0.396784	H	-4.085052	-1.279441	0.455310	H	-4.048902	-1.248636	0.446468
H	-4.255223	-1.453757	-1.336240	H	-4.261140	-1.484368	-1.276082	H	-4.263242	-1.523091	-1.272080
C	-5.342964	0.219926	-0.532158	C	-5.345203	0.195475	-0.479343	C	-5.334162	0.182585	-0.516757
H	-5.159882	1.022845	0.188182	H	-5.159639	1.001633	0.236730	H	-5.135218	1.024586	0.153230
H	-5.441405	0.702940	-1.511026	H	-5.444427	0.673969	-1.460338	H	-5.465632	0.610487	-1.517164
C	-6.672706	-0.455001	-0.179720	C	-6.675825	-0.475036	-0.121839	C	-6.646890	-0.477819	-0.087233
H	-6.557845	-0.925251	0.804112	H	-6.560523	-0.941581	0.863764	H	-6.508974	-0.866712	0.928635
C	-7.774527	0.603493	-0.067074	C	-7.774213	0.587385	-0.012003	C	-7.763514	0.570480	-0.041487
H	-7.438631	1.383150	0.623278	H	-7.434808	1.369005	0.674301	H	-7.417722	1.422004	0.551983
H	-7.896738	1.089842	-1.041662	H	-7.896272	1.069676	-0.988473	H	-7.929516	0.950305	-1.055766
C	-9.127801	0.079806	0.404972	C	-9.129948	0.071826	0.464094	C	-9.087285	0.074343	0.533502
H	-9.001906	-0.468327	1.344199	H	-9.001241	-0.474306	1.405083	H	-8.910426	-0.365190	1.521011
H	-9.522511	-0.631336	-0.322054	H	-9.526028	-0.641690	-0.259408	H	-9.492741	-0.723420	-0.091290
C	-10.142243	1.204398	0.608872	C	-10.137452	1.199385	0.667118	C	-10.125453	1.184166	0.658585
H	-9.713007	1.963097	1.270883	H	-9.704917	1.948194	1.337055	H	-9.724553	1.984812	1.286077
H	-10.289132	1.714645	-0.358518	H	-10.317275	1.705260	-0.287119	H	-10.314200	1.625247	-0.326444
C	-11.471592	0.781124	1.152271	C	-11.487399	0.752344	1.245224	C	-11.460820	0.714127	1.248698
C	-12.387521	1.859447	1.626955	H	-11.274937	0.242615	2.198542	H	-11.254233	0.246776	2.216575
H	-11.846226	2.634092	2.171698	C	-12.353579	1.932345	1.521983	C	-12.393480	1.912033	1.497260
H	-12.891028	2.355868	0.784306	H	-11.921360	2.861286	1.865656	H	-11.935675	2.624746	2.185292
C	-7.038769	-1.542228	-1.189502	C	-7.046439	-1.565225	-1.126670	H	-12.602462	2.427893	0.559103
H	-7.170252	-1.104226	-2.182656	H	-7.178930	-1.130647	-2.121158	C	-7.011613	-1.646839	-1.002460
H	-6.264783	-2.304984	-1.258557	H	-6.274342	-2.330021	-1.194382	H	-7.142199	-1.293865	-2.029073
H	-7.965319	-2.046169	-0.919466	H	-7.973672	-2.065884	-0.852844	H	-6.236989	-2.412415	-1.008319
C	-12.082035	-0.496484	0.678918	C	-12.207238	-0.249124	0.338930	H	-7.939421	-2.126051	-0.694364
H	-12.349863	-0.442577	-0.386932	H	-12.344429	0.176924	-0.657035	C	-12.125812	-0.291159	0.371168
H	-11.401821	-1.341957	0.786913	H	-11.649284	-1.178976	0.240865	H	-12.098954	-0.169842	-0.702140
H	-12.995415	-0.723651	1.228273	H	-13.191545	-0.491414	0.739840	H	-12.774652	-1.050564	0.782041
C	9.097162	2.356756	-0.340317	C	9.079863	2.382888	-0.371776	C	9.096356	2.356228	-0.350322
H	9.841006	1.934634	-1.022555	H	9.822542	1.966656	-1.058884	H	9.875673	1.901001	-0.968798
H	8.675463	3.228646	-0.828857	H	8.649223	3.252274	-0.856996	H	8.693401	3.193970	-0.909295
H	9.624021	2.710534	0.550196	H	9.609551	2.739327	0.515999	H	9.576456	2.766775	0.542132
C	6.242847	2.742970	-1.085999	C	6.216882	2.754854	-1.091323	C	6.267616	2.697030	-1.202812
H	5.177227	2.695582	-1.280221	H	5.149557	2.702755	-1.274698	H	5.211801	2.631147	-1.440087
H	6.444236	3.655288	-0.522506	H	6.419518	3.666027	-0.526448	H	6.435128	3.631986	-0.666077
H	6.760742	2.827318	-2.042360	H	6.724263	2.845602	-2.052757	H	6.827165	2.750705	-2.137444
C	7.951922	-2.006767	1.827231	C	7.979402	-1.995524	1.788854	C	7.899083	-1.920705	1.958055
H	7.631172	-1.970556	2.870852	H	7.668055	-1.964634	2.835519	H	7.531255	-1.852064	2.984012
H	7.522341	-2.907696	1.388715	H	7.551010	-2.897447	1.351225	H	7.499764	-2.842084	1.532858
H	9.033429	-2.096582	1.812321	H	9.061238	-2.079016	1.763798	H	8.981113	-1.997527	1.994401
O	9.738317	-0.016558	1.052378	O	9.746909	0.008791	1.007244	O	9.702031	0.049986	1.163020
H	10.279390	0.689254	0.695262	H	10.280658	0.719527	0.648876	H	10.251428	0.749705	0.802272

H	5.307757	-1.907339	2.212738	H	5.337432	-1.913551	2.196437	H	5.248336	-1.820999	2.255375
H	2.992285	-1.890757	1.085433	H	3.012873	-1.907751	1.087111	H	2.966909	-1.860452	1.071124
C	3.883384	-1.229576	-1.501084	C	3.877915	-1.230591	-1.503481	C	3.906409	-1.293226	-1.517026
H	3.317495	-2.160015	-1.457175	H	3.317054	-2.164028	-1.458887	H	3.328609	-2.215459	-1.457824
H	3.636473	-0.711689	-2.427693	H	3.621004	-0.710094	-2.425885	H	3.692629	-0.803560	-2.467096
H	4.944244	-1.475793	-1.521591	H	4.939789	-1.471306	-1.533419	H	4.964567	-1.551906	-1.498425
H	-1.567427	-1.328411	0.296383	H	-1.564827	-1.348150	0.344818	H	-1.524445	-1.300634	0.293825
C	-1.525512	-1.880566	-1.772173	C	-1.531990	-1.911775	-1.720825	C	-1.552505	-1.979945	-1.738164
H	-2.408001	-2.517529	-1.743312	H	-2.413490	-2.549740	-1.684433	H	-2.398065	-2.652882	-1.604368
H	-1.485319	-1.404250	-2.755554	H	-1.496974	-1.440916	-2.707036	H	-1.609760	-1.562288	-2.747462
H	-0.655895	-2.529423	-1.678401	H	-0.661010	-2.558878	-1.627459	H	-0.647952	-2.583264	-1.676790
H	-13.171535	1.464817	2.274580	H	-13.428849	1.832016	1.550208	H	-13.344291	1.586455	1.918769
C	-0.315717	0.076510	-0.719933	C	-0.320475	0.053290	-0.685551	C	-0.312093	0.036100	-0.844279
H	-0.307186	0.622128	-1.670046	H	-0.319363	0.595412	-1.637702	H	-0.306077	0.489667	-1.841492
H	-0.401497	0.832798	0.065893	H	-0.402871	0.812385	0.097939	H	-0.391152	0.864352	-0.133597

Structures of α-Tocopherol in the SET-PT (SET_step) mechanism at M06-2X/6-311++G(2d,2p) level of theory in gas phase.

**M06-2X/6-311++G(2d,2p)**			
α-Tocopherol $^{•+}$			
C	3.63086200	-0.69154400	-0.39372200
C	5.75276800	0.48868400	-0.14802800
C	6.22322800	-0.47179000	0.80258800
C	5.26967800	-1.51259800	1.32755200
C	3.82417100	-1.12037200	1.04998600
C	6.56749700	1.58435100	-0.61562700
C	7.52413700	-0.39353100	1.22387900
H	5.49898900	-2.48080500	0.87557600
H	3.52811300	-0.29351500	1.69927200
C	8.32805400	0.67876100	0.73793500
C	7.85972800	1.66734800	-0.17054100
O	4.55395900	0.43697100	-0.65803700
C	2.25793500	-0.08272700	-0.64939800
H	2.25339500	0.32024500	-1.66516200
H	2.12925300	0.76303400	0.03022600
C	1.09024100	-1.05362700	-0.48258200
H	1.23846600	-1.92365400	-1.12398800
H	1.04660900	-1.41888400	0.54727300
C	-1.48169800	-1.19916800	-0.46891200
C	-2.73821300	-0.38944000	-0.80375600
H	-2.78727100	-0.24673100	-1.88910200
H	-2.63502400	0.60934500	-0.36871900
C	-4.04978400	-0.99854300	-0.31499400
H	-3.97496900	-1.20588600	0.75792500
H	-4.21852200	-1.95679000	-0.80719200
C	-5.23912100	-0.07518000	-0.56836500
H	-5.01060100	0.91172500	-0.15468700
H	-5.36226700	0.06683900	-1.64804700
C	-6.56986900	-0.55271300	0.02146200
H	-6.42639100	-0.69520700	1.09928800
C	-7.63421100	0.53241200	-0.16932100
H	-7.24814000	1.47275300	0.23583900
H	-7.77928200	0.69923400	-1.24286600
C	-8.98503700	0.23951300	0.47876600
H	-8.83382000	-0.02418100	1.53134900
H	-9.44164900	-0.62850500	0.00189100
C	-9.93594000	1.42979800	0.38827700
H	-9.43735500	2.31245500	0.80031800
H	-10.13452800	1.65434600	-0.66601700
C	-11.27314500	1.24269700	1.10958300
H	-11.05873000	1.03244000	2.16279800
C	-12.09507800	2.52750300	1.03600900
H	-11.55071600	3.37267800	1.45848800
H	-12.33146100	2.76643900	-0.00348400
C	-7.00946500	-1.88536300	-0.58297400
H	-7.17460900	-1.77528200	-1.65804000
H	-6.26169300	-2.66326600	-0.43463100
H	-7.93667700	-2.24068200	-0.13659700
C	-12.07247100	0.07146700	0.54124800
H	-12.24305200	0.21475600	-0.52879600
H	-11.56043000	-0.88017500	0.67685700

H	-13.04621300	-0.00419200	1.02569600
C	8.81508700	2.74635400	-0.59666700
H	9.70486000	2.31966000	-1.06658500
H	8.36706000	3.41999900	-1.31816400
H	9.13300300	3.35026900	0.25721300
C	5.98591300	2.58092500	-1.57662700
H	4.93298400	2.39639600	-1.75190300
H	6.09884100	3.59228000	-1.18768600
H	6.50574600	2.53443500	-2.53434400
C	8.13951900	-1.36289200	2.18747300
H	8.20499700	-0.92023500	3.18347300
H	7.56325700	-2.27977200	2.25364000
H	9.15420300	-1.60972100	1.88279700
O	9.56464900	0.71577200	1.20392700
H	10.06097100	1.46664500	0.85578700
H	5.42109200	-1.62876400	2.39957300
H	3.16021000	-1.95575400	1.26190400
C	3.99435700	-1.77451700	-1.39958300
H	3.41716300	-2.67477200	-1.19568300
H	3.77217100	-1.44011700	-2.41144200
H	5.05228800	-2.02943500	-1.34626000
H	-1.47029200	-1.36687400	0.61456800
C	-1.48056500	-2.55931800	-1.16482700
H	-2.36798200	-3.13520100	-0.91018500
H	-1.46442200	-2.42957000	-2.25005600
H	-0.61597100	-3.15981000	-0.88256500
H	-13.03663700	2.42584300	1.57598000
C	-0.23782600	-0.38040300	-0.82688000
H	-0.25688900	-0.15268300	-1.89800400
H	-0.29146800	0.58018800	-0.30638000

Structures of α-Tocopherol in the SET-PT (PT_step) mechanism at M06-2X/6-311++G(2d,2p) level of theory in gas phase.

M06-2X/6-311++G(2d,2p)											
**α-Tocopherol •+**				**α-Tocopherol (O•)**				**α-Tocopherol (C1•)**			
C	3.63086200	-0.69154400	-0.39372200	C	3.674166	-0.678629	-0.390615	C	3.667217	-0.674839	-0.390844
C	5.75276800	0.48868400	-0.14802800	C	5.794306	0.503229	-0.137617	C	5.786307	0.515012	-0.168363
C	6.22322800	-0.47179000	0.80258800	C	6.265325	-0.461181	0.808728	C	6.260168	-0.427172	0.798726
C	5.26967800	-1.51259800	1.32755200	C	5.312767	-1.505772	1.327947	C	5.309817	-1.460907	1.343108
C	3.82417100	-1.12037200	1.04998600	C	3.866949	-1.114141	1.051161	C	3.863034	-1.077392	1.060073
C	6.56749700	1.58435100	-0.61562700	C	6.607927	1.602218	-0.599314	C	6.597752	1.604262	-0.656225
C	7.52413700	-0.39353100	1.22387900	C	7.565807	-0.383217	1.231389	C	7.561279	-0.338090	1.217233
H	5.49898900	-2.48080500	0.87557600	H	5.543715	-2.471487	0.871494	H	5.541187	-2.436419	0.908211
H	3.52811300	-0.29351500	1.69927200	H	3.569293	-0.290813	1.704190	H	3.565474	-0.239865	1.694834
C	8.32805400	0.67876100	0.73793500	C	8.368661	0.692471	0.751227	C	8.361954	0.727435	0.711455
C	7.85972800	1.66734800	-0.17054100	C	7.899707	1.684793	-0.152842	C	7.890196	1.698484	-0.214002
O	4.55395900	0.43697100	-0.65803700	O	4.595956	0.452374	-0.648789	O	4.587141	0.451152	-0.676150
C	2.25793500	-0.08272700	-0.64939800	C	2.300622	-0.070418	-0.644413	C	2.292475	-0.074193	-0.655923
H	2.25339500	0.32024500	-1.66516200	H	2.296317	0.337431	-1.658229	H	2.285909	0.310614	-1.678696
H	2.12925300	0.76303400	0.03022600	H	2.170293	0.771891	0.039174	H	2.162271	0.783205	0.008664
C	1.09024100	-1.05362700	-0.48258200	C	1.134100	-1.043664	-0.483166	C	1.127453	-1.044957	-0.470659
H	1.23846600	-1.92365400	-1.12398800	H	1.283975	-1.910396	-1.128636	H	1.277300	-1.925886	-1.096622
H	1.04660900	-1.41888400	0.54727300	H	1.090172	-1.413930	0.544886	H	1.085765	-1.391927	0.565581
C	-1.48169800	-1.19916800	-0.46891200	C	-1.437652	-1.192695	-0.472170	C	-1.444087	-1.196823	-0.451783
C	-2.73821300	-0.38944000	-0.80375600	C	-2.694993	-0.383040	-0.804079	C	-2.703012	-0.396410	-0.799713
H	-2.78727100	-0.24673100	-1.88910200	H	-2.743415	-0.235175	-1.888764	H	-2.753493	-0.273177	-1.887378
H	-2.63502400	0.60934500	-0.36871900	H	-2.593469	0.613777	-0.364163	H	-2.601978	0.610226	-0.382635
C	-4.04978400	-0.99854300	-0.31499400	C	-4.006120	-0.996234	-0.319260	C	-4.012531	-1.000071	-0.298849
H	-3.97496900	-1.20588600	0.75792500	H	-3.931845	-1.208638	0.752706	H	-3.936139	-1.188082	0.777515
H	-4.21852200	-1.95679000	-0.80719200	H	-4.173204	-1.952326	-0.816192	H	-4.179274	-1.967361	-0.773735
C	-5.23912100	-0.07518000	-0.56836500	C	-5.196497	-0.073248	-0.569099	C	-5.204493	-0.084418	-0.567411
H	-5.01060100	0.91172500	-0.15468700	H	-4.969610	0.911959	-0.150503	H	-4.978119	0.910280	-0.171604
H	-5.36226700	0.06683900	-1.64804700	H	-5.319010	0.073801	-1.648179	H	-5.329054	0.038035	-1.649325
C	-6.56986900	-0.55271300	0.02146200	C	-6.527054	-0.555386	0.017402	C	-6.533431	-0.554781	0.032178
H	-6.42639100	-0.69520700	1.09928800	H	-6.384206	-0.702873	1.094640	H	-6.388538	-0.677690	1.112224
C	-7.63421100	0.53241200	-0.16932100	C	-7.592699	0.529226	-0.168975	C	-7.600754	0.524043	-0.176813
H	-7.24814000	1.47275300	0.23583900	H	-7.208193	1.468120	0.241000	H	-7.216718	1.472437	0.211146
H	-7.77928200	0.69923400	-1.24286600	H	-7.737176	0.701019	-1.241816	H	-7.747298	0.671349	-1.253010
C	-8.98503700	0.23951300	0.47876600	C	-8.943626	0.231413	0.476658	C	-8.950190	0.239271	0.477760
H	-8.83382000	-0.02418100	1.53134900	H	-8.792858	-0.037142	1.528076	H	-8.797270	-0.005248	1.534717

H	-9.44164900	-0.62850500	0.00189100	H	-9.398715	-0.634908	-0.004738	H	-9.405023	-0.638269	0.016881
C	-9.93594000	1.42979800	0.38827700	C	-9.896048	1.420852	0.391167	C	-9.904249	1.425315	0.367059
H	-9.43735500	2.31245500	0.80031800	H	-9.398957	2.302179	0.807832	H	-9.407543	2.316444	0.762811
H	-10.13452800	1.65434600	-0.66601700	H	-10.094134	1.650205	-0.662187	H	-10.104439	1.630542	-0.690863
C	-11.27314500	1.24269700	1.10958300	C	-11.233551	1.228501	1.110538	C	-11.240265	1.247662	1.092942
H	-11.05873000	1.03244000	2.16279800	H	-11.019657	1.013464	2.162893	H	-11.024285	1.056744	2.149515
C	-12.09507800	2.52750300	1.03600900	C	-12.057143	2.512550	1.042515	C	-12.065582	2.528843	0.997339
H	-11.55071600	3.37267800	1.45848800	H	-11.514232	3.356406	1.469473	H	-11.522993	3.382800	1.404148
H	-12.33146100	2.76643900	-0.00348400	H	-12.293054	2.756170	0.004002	H	-12.303590	2.748623	-0.046002
C	-7.00946500	-1.88536300	-0.58297400	C	-6.964409	-1.885696	-0.593775	C	-6.970173	-1.899106	-0.547986
H	-7.17460900	-1.77528200	-1.65804000	H	-7.128882	-1.770663	-1.668426	H	-7.136645	-1.808611	-1.624673
H	-6.26169300	-2.66326600	-0.43463100	H	-6.215712	-2.663307	-0.448603	H	-6.220252	-2.672324	-0.386576
H	-7.93667700	-2.24068200	-0.13659700	H	-7.891485	-2.244394	-0.149825	H	-7.896031	-2.248794	-0.094410
C	-12.07247100	0.07146700	0.54124800	C	-12.030879	0.058955	0.535961	C	-12.037117	0.064452	0.546365
H	-12.24305200	0.21475600	-0.52879600	H	-12.200837	0.207164	-0.533512	H	-12.209107	0.188225	-0.525886
H	-11.56043000	-0.88017500	0.67685700	H	-11.517671	-0.892645	0.667383	H	-11.522491	-0.883309	0.698376
H	-13.04621300	-0.00419200	1.02569600	H	-13.004888	-0.020331	1.019292	H	-13.010190	-0.005063	1.033074
C	8.81508700	2.74635400	-0.59666700	C	8.853947	2.767109	-0.573038	C	8.842353	2.772175	-0.660245
H	9.70486000	2.31966000	-1.06658500	H	9.744647	2.343867	-1.044320	H	9.732767	2.339462	-1.123395
H	8.36706000	3.41999900	-1.31816400	H	8.405570	3.443620	-1.291630	H	9.159539	3.392013	0.182423
H	9.13300300	3.35026900	0.25721300	H	9.170406	3.367331	0.283981	C	6.012664	2.582071	-1.634225
C	5.98591300	2.58092500	-1.57662700	C	6.025744	2.602629	-1.555954	H	4.960045	2.391752	-1.805117
H	4.93298400	2.39639600	-1.75190300	H	4.973196	2.417543	-1.732924	H	6.123360	3.600478	-1.263469
H	6.09884100	3.59228000	-1.18768600	H	6.137025	3.612250	-1.162066	H	6.531685	2.519865	-2.591491
H	6.50574600	2.53443500	-2.53434400	H	6.546367	2.561440	-2.513484	C	8.180096	-1.288557	2.197308
C	8.13951900	-1.36289200	2.18747300	C	8.181750	-1.356383	2.190781	H	8.245399	-0.828066	3.185201
H	8.20499700	-0.92023500	3.18347300	H	8.245878	-0.918437	3.188949	H	7.606267	-2.205587	2.280376
H	7.56325700	-2.27977200	2.25364000	H	7.606663	-2.274338	2.252094	H	9.195118	-1.538171	1.896043
H	9.15420300	-1.60972100	1.88279700	H	9.196994	-1.600391	1.885700	O	9.598901	0.775908	1.175456
O	9.56464900	0.71577200	1.20392700	O	9.604850	0.728886	1.218341	H	10.092945	1.521732	0.813497
H	10.06097100	1.46664500	0.85578700	H	5.463520	-1.626894	2.399512	H	5.462572	-1.557576	2.416873
H	5.42109200	-1.62876400	2.39957300	H	3.203943	-1.951417	1.258548	H	3.201437	-1.910567	1.287507
H	3.16021000	-1.95575400	1.26190400	C	4.039873	-1.756264	-1.401395	C	4.032528	-1.774618	-1.377632
C	3.99435700	-1.77451700	-1.39958300	H	3.463727	-2.658258	-1.202271	H	3.457857	-2.672575	-1.157148
H	3.41716300	-2.67477200	-1.19568300	H	3.818011	-1.417295	-2.411804	H	3.808497	-1.458861	-2.395058
H	3.77217100	-1.44011700	-2.41144200	H	5.098103	-2.010026	-1.348487	H	5.091164	-2.025834	-1.320855
H	5.05228800	-2.02943500	-1.34626000	H	-1.426847	-1.365597	0.610499	H	-1.431196	-1.345178	0.634499
H	-1.47029200	-1.36687400	0.61456800	C	-1.434173	-2.549478	-1.174620	C	-1.440120	-2.569143	-1.123370
C	-1.48056500	-2.55931800	-1.16482700	H	-2.321013	-3.127761	-0.923433	H	-2.325800	-3.142673	-0.857612
H	-2.36798200	-3.13520100	-0.91018500	H	-1.417377	-2.414488	-2.259199	H	-1.425366	-2.458701	-2.210753
H	-1.46442200	-2.42957000	-2.25005600	H	-0.568992	-3.150169	-0.894587	H	-0.573705	-3.162295	-0.831340
H	-0.61597100	-3.15981000	-0.88256500	H	-12.998976	2.407038	1.581267	H	-13.006350	2.434402	1.539992
H	-13.03663700	2.42584300	1.57598000	C	-0.194603	-0.370561	-0.825240	C	-0.202680	-0.381377	-0.825538
C	-0.23782600	-0.38040300	-0.82688000	H	-0.213155	-0.137715	-1.895271	H	-0.223372	-0.172818	-1.900527
H	-0.25688900	-0.15268300	-1.89800400	H	-0.249924	0.587442	-0.300166	H	-0.258296	0.588190	-0.322170
H	-0.29146800	0.58018800	-0.30638000								
**α-Tocopherol (C2•)**				**α-Tocopherol (C3•)**				**α-Tocopherol (C4•)**			
C	3.640073	-0.413714	-0.287871	C	3.661986	-0.714029	-0.364937	C	3.641367	-0.311516	-0.341204
C	5.832638	0.536128	-0.043979	C	5.785352	0.467533	-0.139069	C	5.825227	0.585347	0.089841
C	6.233077	-0.469165	0.819829	C	6.252893	-0.474926	0.830823	C	6.194488	-0.572295	0.826057
C	5.218702	-1.440572	1.371753	C	5.296941	-1.503839	1.374533	C	5.190744	-1.455501	1.239044
C	3.796571	-0.947858	1.132673	C	3.852463	-1.114900	1.087156	C	3.761750	-1.193702	0.902232
C	6.756627	1.500331	-0.551643	C	6.602483	1.552625	-0.626707	C	6.760862	1.524542	-0.320304
C	7.584470	-0.579027	1.175578	C	7.553289	-0.390445	1.252497	C	7.569482	-0.785074	1.121447
H	5.359663	-2.423667	0.912240	H	5.525425	-2.481028	0.941883	H	5.439439	-2.316238	1.841008
H	3.564998	-0.132466	1.821508	H	3.556716	-0.275082	1.719733	H	3.257076	-0.690772	1.737923
C	8.501624	0.365698	0.690817	C	8.359599	0.670935	0.746956	C	8.485230	0.157896	0.705164
C	8.117331	1.410929	-0.128542	C	7.894172	1.642321	-0.181349	C	8.108635	1.314306	-0.000788
O	4.545352	0.691799	-0.457795	O	4.587231	0.407706	-0.649806	O	4.508764	0.829776	-0.184187
C	2.265849	0.210805	-0.516420	C	2.290392	-0.108224	-0.634543	C	2.252593	0.309005	-0.467457
H	2.300826	0.730115	-1.477970	H	2.288002	0.274858	-1.657981	H	2.245118	0.933505	-1.364866
H	2.115954	0.977978	0.247375	H	2.162005	0.750831	0.028254	H	2.115922	0.981882	0.382732
C	1.091118	-0.762729	-0.510566	C	1.120942	-1.073904	-0.450604	C	1.094814	-0.683372	-0.522908
H	1.245066	-1.523298	-1.277296	H	1.268777	-1.956505	-1.074687	H	1.259692	-1.400357	-1.328722
H	1.036306	-1.286616	0.448218	H	1.075198	-1.418931	0.586113	H	1.051272	-1.258372	0.406682
C	-1.481697	-0.920362	-0.631455	C	-1.451238	-1.215230	-0.438032	C	-1.476788	-0.871731	-0.616227
C	-2.737468	-0.050036	-0.746922	C	-2.705990	-0.410276	-0.790532	C	-2.745130	-0.019317	-0.726280
H	-2.750386	0.421248	-1.736364	H	-2.753198	-0.288696	-1.878529	H	-2.768955	0.451569	-1.715714
H	-2.662378	0.765300	-0.021255	H	-2.601902	0.596649	-0.374906	H	-2.678529	0.797301	-0.001135
C	-4.056011	-0.786314	-0.524413	C	-4.019237	-1.007727	-0.291987	C	-4.052884	-0.773056	-0.498301
H	-4.012161	-1.328192	0.426808	H	-3.946354	-1.194210	0.784886	H	-4.000609	-1.309216	0.455660
H	-4.190337	-1.536304	-1.304080	H	-4.188725	-1.975136	-0.765655	H	-4.178185	-1.528590	-1.274062
C	-5.254533	0.159222	-0.511969	C	-5.206757	-0.087671	-0.565144	C	-5.263666	0.156842	-0.489875
H	-5.060598	0.962744	0.204861	H	-4.977324	0.906768	-0.170449	H	-5.080524	0.965029	0.224518
H	-5.345991	0.639402	-1.492928	H	-5.328062	0.033442	-1.647578	H	-5.359846	0.632699	-1.472508

C	-6.593590	-0.495579	-0.157065	C	-6.539128	-0.551576	0.031851	C	-6.595378	-0.512698	-0.134996
H	-6.482669	-0.970366	0.825141	H	-6.397489	-0.673232	1.112470	H	-6.481817	-0.981224	0.849877
C	-7.678587	0.579411	-0.037285	C	-7.601496	0.531241	-0.181694	C	-7.692915	0.550555	-0.024250
H	-7.328631	1.351060	0.654990	H	-7.214572	1.478718	0.205634	H	-7.354864	1.328660	0.666745
H	-7.796117	1.071156	-1.009647	H	-7.744697	0.677305	-1.258510	H	-7.810702	1.037495	-0.999036
C	-9.039346	0.075588	0.437088	C	-8.953746	0.253102	0.469916	C	-9.051042	0.034505	0.444406
H	-8.913149	-0.485219	1.369786	H	-8.804518	0.009765	1.527673	H	-8.925193	-0.519659	1.381088
H	-9.446728	-0.623676	-0.294061	H	-9.410990	-0.623359	0.009368	H	-9.446292	-0.672848	-0.285552
C	-10.034146	1.210844	0.662260	C	-9.902661	1.442842	0.354768	C	-10.058891	1.160927	0.655718
H	-9.585265	1.947826	1.335096	H	-9.403324	2.332610	0.750273	H	-9.622569	1.905969	1.327915
H	-10.211490	1.729824	-0.286720	H	-10.099319	1.647079	-0.704009	H	-10.233434	1.672825	-0.297656
C	-11.382301	0.776907	1.242914	C	-11.241236	1.271889	1.077540	C	-11.407788	0.717692	1.227642
H	-11.189372	0.276061	2.197529	H	-11.028728	1.081895	2.134983	H	-11.218920	0.227577	2.188617
C	-12.258259	1.998419	1.512746	C	-12.061060	2.556266	0.977657	C	-12.299084	1.932247	1.477683
H	-11.760980	2.704193	2.178935	H	-11.516020	3.408693	1.384399	H	-11.816723	2.648810	2.143332
H	-12.479584	2.519145	0.578289	H	-12.295512	2.775235	-0.066660	H	-12.515277	2.442719	0.536362
C	-6.980165	-1.574037	-1.168577	C	-6.979887	-1.895095	-0.547143	C	-6.966284	-1.600601	-1.142227
H	-7.112523	-1.130426	-2.159123	H	-7.143247	-1.805759	-1.624404	H	-7.100838	-1.163380	-2.135302
H	-6.216917	-2.346777	-1.245997	H	-6.233548	-2.671094	-0.382508	H	-6.193609	-2.364522	-1.213430
H	-7.911725	-2.066262	-0.894183	H	-7.908318	-2.240223	-0.095339	H	-7.892679	-2.102689	-0.868242
C	-12.112682	-0.204328	0.327535	C	-12.041527	0.091013	0.530940	C	-12.118974	-0.279546	0.314441
H	-12.247868	0.234553	-0.664360	H	-12.210280	0.213656	-0.541955	H	-12.248010	0.148163	-0.683123
H	-11.568016	-1.140160	0.210472	H	-11.531169	-0.858582	0.685873	H	-11.563830	-1.210995	0.212540
H	-13.100260	-0.441920	0.724136	H	-13.016111	0.026304	1.015282	H	-13.108228	-0.523157	0.703084
C	9.095337	2.455292	-0.593577	C	8.851845	2.711350	-0.626982	C	9.180418	2.293803	-0.399132
H	9.361344	2.323551	-1.644882	H	9.741658	2.274212	-1.087124	H	9.897966	1.845775	-1.093583
H	8.663611	3.450322	-0.489551	H	8.405948	3.371471	-1.362168	H	8.767326	3.169533	-0.888185
H	10.015457	2.465266	-0.010959	H	9.169418	3.331325	0.215439	H	9.738858	2.647464	0.472696
C	6.314027	2.479714	-1.438024	C	6.023890	2.531144	-1.607856	C	6.318248	2.744221	-1.087412
H	6.987862	3.209233	-1.855928	H	4.970940	2.344835	-1.781109	H	5.249844	2.710101	-1.271071
H	5.280117	2.511652	-1.736803	H	6.137806	3.549722	-1.238547	H	6.537412	3.658721	-0.534230
C	8.047654	-1.684011	2.082644	H	6.545087	2.465184	-2.563687	H	6.829767	2.810722	-2.048356
H	7.811965	-1.459751	3.126578	C	8.165718	-1.341757	2.235759	C	8.001087	-2.007942	1.877706
H	7.557136	-2.625278	1.836367	H	8.230389	-0.879851	3.223033	H	7.542509	-2.035718	2.868353
H	9.121958	-1.819875	2.009341	H	9.180473	-1.596030	1.937510	H	7.695604	-2.915792	1.353872
O	9.803044	0.195052	1.099168	O	9.595549	0.715146	1.214029	H	9.078901	-2.027473	2.000130
H	10.379640	0.750010	0.572979	H	10.093559	1.458326	0.852066	O	9.806377	-0.066661	1.008034
H	5.388690	-1.581081	2.439891	H	5.446566	-1.599296	2.448846	H	10.344678	0.636475	0.642176
H	3.079976	-1.748642	1.311308	H	3.186888	-1.944976	1.314315	H	3.229284	-2.132537	0.738660
C	3.966447	-1.471578	-1.339473	C	4.025307	-1.816974	-1.348922	C	4.043582	-1.063169	-1.606560
H	3.403927	-2.387123	-1.156901	H	3.446409	-2.712199	-1.128381	H	3.382980	-1.912364	-1.775086
H	3.717909	-1.098250	-2.332662	H	3.805158	-1.502043	-2.367451	H	3.982897	-0.394594	-2.465012
H	5.028443	-1.713550	-1.323487	H	5.082759	-2.072415	-1.289017	H	5.062906	-1.438429	-1.528072
H	-1.476798	-1.368406	0.369488	H	-1.441718	-1.361781	0.648529	H	-1.466450	-1.329038	0.380440
C	-1.481628	-2.050132	-1.660451	C	-1.451174	-2.588700	-1.107277	C	-1.456472	-1.991977	-1.655341
H	-2.345635	-2.701844	-1.541326	H	-2.339866	-3.158152	-0.842807	H	-2.317781	-2.650239	-1.554233
H	-1.507671	-1.637907	-2.672709	H	-1.433202	-2.480176	-2.194805	H	-1.470730	-1.570928	-2.664259
H	-0.592718	-2.673302	-1.574651	H	-0.587939	-3.184887	-0.812031	H	-0.564803	-2.610275	-1.562296
H	-13.207047	1.712215	1.967523	H	-13.003585	2.466596	1.518067	H	-13.249895	1.639941	1.924298
C	-0.233042	-0.042099	-0.756627	C	-0.205560	-0.405503	-0.810002	C	-0.241469	0.026469	-0.730632
H	-0.216612	0.409849	-1.754664	H	-0.222662	-0.198696	-1.885393	H	-0.243893	0.507991	-1.714833
H	-0.316935	0.786374	-0.047211	H	-0.258490	0.565142	-0.308424	H	-0.327955	0.832390	0.004155
**α-Tocopherol (C5$^{\bullet}$)**				**α-Tocopherol (C6$^{\bullet}$)**				**α-Tocopherol (C7$^{\bullet}$)**			
C	-3.621702	-0.272617	0.298875	C	3.645981	-0.694021	-0.415744	C	3.640203	-0.690769	-0.400509
C	-5.834856	0.562972	-0.130057	C	5.768658	0.481912	-0.156491	C	5.762113	0.490083	-0.157869
C	-6.208771	-0.604667	-0.791408	C	6.239408	-0.491018	0.781227	C	6.232948	-0.468462	0.794507
C	-5.164067	-1.603885	-1.229034	C	5.285769	-1.538083	1.293433	C	5.279638	-1.508281	1.321861
C	-3.788447	-1.168182	-0.880858	C	3.840233	-1.141605	1.022133	C	3.834015	-1.116703	1.043989
C	-6.782634	1.519454	0.247618	C	6.583548	1.583155	-0.610514	C	6.576618	1.584867	-0.627922
C	-7.557005	-0.848961	-1.065310	C	7.540690	-0.418844	1.202450	C	7.533991	-0.389279	1.215213
H	-5.385799	-2.579939	-0.769052	H	5.514247	-2.500464	0.828771	H	5.508863	-2.477374	0.871743
H	-2.930281	-1.619549	-1.354269	H	3.545096	-0.323086	1.682311	H	3.538117	-0.288570	1.691720
C	-8.495606	0.101515	-0.686735	C	8.344742	0.659269	0.729789	C	8.337678	0.682093	0.726865
C	-8.126722	1.285247	-0.044473	C	7.876174	1.659763	-0.165430	C	7.868990	1.668835	-0.183428
O	-4.522534	0.846633	0.158092	O	4.569417	0.437382	-0.666163	O	4.563140	0.437275	-0.667379
C	-2.238045	0.371373	0.349250	C	2.273148	-0.081289	-0.662410	C	2.267153	-0.082552	-0.656947
H	-2.268676	1.147491	1.118480	H	2.267994	0.334808	-1.672865	H	2.138640	0.764556	0.021030
H	-2.074005	0.877457	-0.604980	H	2.145421	0.755660	0.028215	C	1.099577	-1.053192	-0.487810
C	-1.085370	-0.585429	0.637000	C	1.105114	-1.053710	-0.507244	H	1.247647	-1.924487	-1.127527
H	-1.245943	-1.069762	1.601091	H	1.252403	-1.915427	-1.159984	H	1.056307	-1.416395	0.542786
H	-1.055789	-1.383025	-0.112261	H	1.062125	-1.432255	0.517829	C	-1.472348	-1.198870	-0.473003
C	1.485014	-0.777542	0.722262	C	-1.466884	-1.198188	-0.493392	C	-2.729026	-0.389892	-0.809049
C	2.762725	0.067625	0.705482	C	-2.723271	-0.383592	-0.816711	H	-2.778450	-0.249353	-1.894661
H	2.812402	0.646274	1.634989	H	-2.773125	-0.226813	-1.900077	H	-2.625758	0.609766	-0.376040
H	2.688140	0.799256	-0.104780	H	-2.619248	0.609425	-0.368857	C	-4.040397	-0.998103	-0.318641

C	4.058917	-0.719585	0.531857	C	-4.034748	-0.998348	-0.334824	H	-3.965215	-1.203299	0.754665
H	4.005092	-1.304724	-0.392545	H	-3.959179	-1.219606	0.735259	H	-4.209234	-1.957341	-0.808870
H	4.167506	-1.435255	1.347205	H	-4.204346	-1.950059	-0.839256	C	-5.229877	-0.075323	-0.573463
C	5.284291	0.190014	0.483820	C	-5.223837	-0.071213	-0.575258	H	-5.001285	0.912420	-0.161830
H	5.106016	0.982247	-0.249535	H	-4.994505	0.910142	-0.149017	H	-5.353387	0.064533	-1.653385
H	5.399127	0.689864	1.452326	H	-5.347774	0.084837	-1.652911	C	-6.560400	-0.551764	0.017754
C	6.601091	-0.509223	0.131695	C	-6.554346	-0.555710	0.009404	H	-6.416558	-0.692097	1.095815
H	6.476423	-0.982063	-0.849753	H	-6.410078	-0.712221	1.085178	C	-7.624875	0.532910	-0.174844
C	7.719364	0.531142	0.012135	C	-7.618311	0.532303	-0.166453	H	-7.238731	1.474083	0.228311
H	7.392447	1.315145	-0.677635	H	-7.231458	1.467133	0.250542	H	-7.770310	0.697580	-1.248672
H	7.852974	1.016933	0.985447	H	-7.764157	0.713085	-1.237630	C	-8.975468	0.241219	0.474271
C	9.064263	-0.011601	-0.464555	C	-8.968762	0.231679	0.478872	H	-8.823888	-0.020364	1.527328
H	8.922408	-0.563187	-1.400404	H	-8.816834	-0.045699	1.527829	H	-9.432181	-0.627779	-0.000721
H	9.450559	-0.726033	0.263281	H	-9.426178	-0.629872	-0.008838	C	-9.926478	1.431260	0.381719
C	10.091105	1.096448	-0.681836	C	-9.919156	1.423490	0.404571	H	-9.427815	2.314769	0.791834
H	9.664447	1.848260	-1.352678	H	-9.419812	2.300498	0.827609	H	-10.125428	1.653691	-0.672955
H	10.278623	1.606246	0.270244	H	-10.118476	1.661769	-0.646566	C	-11.263434	1.245513	1.103837
C	11.429881	0.630247	-1.259305	C	-11.255877	1.227700	1.124468	H	-11.048659	1.037372	2.157399
H	11.229260	0.143681	-2.219735	H	-11.040724	1.003717	2.174699	C	-12.085474	2.530117	1.027970
C	12.340729	1.829573	-1.512338	C	-12.077241	2.513744	1.068202	H	-11.541028	3.376168	1.448582
H	11.868763	2.553937	-2.176991	H	-11.532130	3.353116	1.501155	H	-12.322214	2.766963	-0.011920
H	12.568170	2.336663	-0.571863	H	-12.314337	2.766236	0.032081	C	-7.000108	-1.885645	-0.583876
C	6.950024	-1.597933	1.145671	C	-6.995074	-1.880210	-0.611888	H	-7.165613	-1.777721	-1.659106
H	7.083510	-1.158882	2.138094	H	-7.161022	-1.756134	-1.685305	H	-6.252237	-2.663203	-0.434227
H	6.165418	-2.350023	1.213774	H	-6.247564	-2.660326	-0.474234	H	-7.927150	-2.240132	-0.136486
H	7.870936	-2.114574	0.880264	H	-7.922103	-2.240838	-0.169406	C	-12.062871	0.073100	0.538104
C	12.127330	-0.379226	-0.348933	C	-12.056227	0.064311	0.541654	H	-12.233813	0.214242	-0.532168
H	12.264987	0.045106	0.648920	H	-12.227592	0.221529	-0.526307	H	-11.550724	-0.878237	0.675443
H	11.557650	-1.301890	-0.247145	H	-11.544542	-0.889251	0.664514	H	-13.036448	-0.001654	1.023022
H	13.111773	-0.638100	-0.739875	H	-13.029619	-0.017151	1.025864	C	8.824139	2.747049	-0.612020
C	-9.206051	2.273041	0.319355	C	8.831719	2.743742	-0.578313	H	9.713785	2.319475	-1.081379
H	-9.914111	1.847254	1.036330	H	9.720909	2.322745	-1.054433	H	8.375831	3.419224	-1.334713
H	-8.798370	3.172378	0.768419	H	8.383445	3.426889	-1.290664	H	9.142296	3.352688	0.240548
H	-9.771792	2.583871	-0.563486	H	9.150610	3.336394	0.283062	C	5.994653	2.579484	-1.590717
C	-6.341950	2.777608	0.951899	C	6.001684	2.592370	-1.558058	H	4.941678	2.394538	-1.765278
H	-5.269967	2.767912	1.114718	H	4.948525	2.410629	-1.734863	H	6.107643	3.591620	-1.203833
H	-6.586793	3.663750	0.364285	H	6.115415	3.598548	-1.156142	H	6.514174	2.531116	-2.548511
H	-6.834011	2.879778	1.920087	H	6.520730	2.558040	-2.516715	C	8.149752	-1.356675	2.180537
C	-7.979741	-2.111726	-1.765511	C	8.156368	-1.400898	2.152913	H	8.215529	-0.912027	3.175630
H	-7.521374	-2.184909	-2.753579	H	8.222856	-0.971210	3.154510	H	7.573572	-2.273458	2.248724
H	-7.670003	-2.993794	-1.202049	H	7.579712	-2.318283	2.207663	H	9.164352	-1.604047	1.876021
H	-9.057233	-2.146025	-1.887680	H	9.170688	-1.644244	1.844250	O	9.574424	0.720113	1.192376
O	-9.812832	-0.163553	-0.973283	O	9.581726	0.689650	1.195227	H	10.070583	1.470321	0.842575
H	-10.362332	0.548933	-0.643317	H	10.078137	1.444733	0.856443	H	5.431413	-1.622298	2.394062
H	-5.246781	-1.776829	-2.305674	H	5.437982	-1.668197	2.363737	H	3.170178	-1.951702	1.257792
C	-3.965117	-0.997128	1.605940	H	3.176034	-1.979345	1.223747	C	4.003437	-1.775724	-1.404326
H	-3.351422	-1.888551	1.725593	C	4.008146	-1.764049	-1.435839	H	3.426369	-2.675607	-1.198440
H	-3.797865	-0.331057	2.453449	H	3.430675	-2.666594	-1.243153	H	3.780897	-1.443358	-2.416777
H	-5.011788	-1.300905	1.603552	H	5.065994	-2.020141	-1.386673	H	5.061402	-2.030467	-1.350842
H	1.485643	-1.407134	-0.175489	H	-1.454696	-1.379919	0.587815	H	-1.460575	-1.364412	0.610806
C	1.431021	-1.691024	1.946440	C	-1.466970	-2.549211	-1.206866	C	-1.471356	-2.560406	-1.166203
H	2.307868	-2.333722	2.005982	H	-2.354464	-3.127920	-0.958991	H	-2.358652	-3.135836	-0.910120
H	1.387936	-1.093250	2.860796	H	-1.451629	-2.405425	-2.290336	H	-1.455579	-2.432823	-2.251694
H	0.555712	-2.338800	1.926934	H	-0.602444	-3.153721	-0.933101	H	-0.606631	-3.160278	-0.883027
H	13.285236	1.521048	-1.961444	H	-13.018419	2.405548	1.607568	H	-13.026849	2.429475	1.568452
C	0.259504	0.138731	0.648054	C	-0.222899	-0.375449	-0.841726	C	-0.228647	-0.380741	-0.833014
H	0.285318	0.833324	1.495175	H	-0.242705	-0.133866	-1.909795	H	-0.248077	-0.155161	-1.904584
H	0.337876	0.752973	-0.253685	H	-0.275657	0.578345	-0.308785	H	-0.282180	0.580883	-0.314414
**α-Tocopherol (C8$^{\bullet}$)**				**α-Tocopherol (C9$^{\bullet}$)**				**α-Tocopherol (C10$^{\bullet}$)**			
C	3.635168	-0.699118	-0.389186	C	3.598660	-0.607696	-0.238373	C	3.625075	-0.692075	-0.416515
C	5.757219	0.481548	-0.146872	C	5.718472	0.530999	-0.186883	C	5.746533	0.483475	-0.145801
C	6.227503	-0.476176	0.806600	C	6.106312	-0.148476	0.965937	C	6.217025	-0.496336	0.784855
C	5.273788	-1.515302	1.334588	C	5.108971	-0.984397	1.728776	C	5.263656	-1.548092	1.287867
C	3.828347	-1.123705	1.055792	C	3.681806	-0.693700	1.282844	C	3.818104	-1.150653	1.018058
C	6.572117	1.575721	-0.617664	C	6.617786	1.331868	-0.899647	C	6.561047	1.588847	-0.590365
C	7.528401	-0.396846	1.227724	C	7.427968	-0.055232	1.412767	C	7.517787	-0.426412	1.208061
H	5.502993	-2.484871	0.885485	H	5.337158	-2.045755	1.588660	H	5.493406	-2.506658	0.816002
H	3.532367	-0.294895	1.702618	H	3.353702	0.266951	1.685854	H	3.521595	-0.337515	1.684244
C	8.332486	0.673899	0.738656	C	8.317520	0.741426	0.705440	C	8.321503	0.655966	0.744669
C	7.864337	1.659860	-0.172760	C	7.929200	1.442056	-0.438746	C	7.853123	1.663012	-0.143274
O	4.558428	0.428489	-0.656787	O	4.434797	0.474730	-0.674460	O	4.547888	0.441962	-0.657126
C	2.262335	-0.090876	-0.646729	C	2.204376	-0.196368	-0.705955	C	2.252027	-0.078522	-0.659931
H	2.257900	0.309095	-1.663679	H	2.270597	0.039779	-1.771037	H	2.247652	0.345401	-1.667131
H	2.133731	0.756906	0.030388	H	1.933567	0.729249	-0.193712	H	2.122879	0.752940	0.037028
C	1.094503	-1.061123	-0.477105	C	1.108745	-1.240871	-0.491450	C	1.084595	-1.053034	-0.513605

H	1.242644	-1.933061	-1.115931	H	1.374249	-2.156352	-1.038143	H	1.233283	-1.909543	-1.172850
C	-1.477456	-1.206281	-0.463135	H	1.053944	-1.531848	0.563090	H	1.040782	-1.439558	0.508451
C	-2.733846	-0.397378	-0.800433	C	-1.490187	-1.396931	-0.451776	C	-1.487301	-1.199632	-0.503712
H	-2.782831	-0.257869	-1.886198	C	-2.711520	-0.558068	-0.840322	C	-2.743978	-0.383536	-0.822083
H	-2.630546	0.602673	-0.368343	H	-2.748959	-0.478173	-1.932392	H	-2.792768	-0.218392	-1.904254
C	-4.045523	-1.004861	-0.309941	H	-2.565297	0.457472	-0.461981	H	-2.641233	0.606057	-0.366421
H	-3.970789	-1.209044	0.763590	C	-4.045218	-1.096167	-0.329544	C	-4.055495	-1.003042	-0.346429
H	-4.214364	-1.964535	-0.799315	H	-3.975261	-1.277119	0.748725	H	-3.980924	-1.232540	0.721987
C	-5.234724	-0.082092	-0.566098	H	-4.249053	-2.061285	-0.794773	H	-4.223786	-1.950941	-0.858419
H	-5.006093	0.906000	-0.155325	C	-5.199528	-0.135964	-0.604577	C	-5.245054	-0.075001	-0.580971
H	-5.357797	0.056754	-1.646201	H	-4.941518	0.845839	-0.196501	H	-5.016968	0.903195	-0.146873
C	-6.565565	-0.557703	0.025070	H	-5.305481	0.001605	-1.686889	H	-5.367934	0.089312	-1.657516
H	-6.422160	-0.697032	1.103319	C	-6.552064	-0.564764	-0.026579	C	-6.575820	-0.565065	-0.001554
C	-7.629753	0.526995	-0.168971	H	-6.419949	-0.724513	1.050289	H	-6.432607	-0.729809	1.073131
H	-7.243577	1.468477	0.233429	C	-7.573348	0.562202	-0.209737	C	-7.640453	0.523446	-0.170135
H	-7.774748	0.690665	-1.243012	H	-7.155918	1.481689	0.211465	H	-7.254798	1.455314	0.254529
C	-8.980651	0.236191	0.479910	H	-7.703997	0.749062	-1.281875	H	-7.785267	0.712423	-1.240037
H	-8.829522	-0.024413	1.533274	C	-8.938945	0.309028	0.424452	C	-8.991372	0.216763	0.471348
H	-9.437354	-0.633171	0.005577	H	-8.804457	0.025017	1.474131	H	-8.840375	-0.068629	1.518288
C	-9.931391	1.426330	0.385857	H	-9.420985	-0.536302	-0.067855	H	-9.447569	-0.641334	-0.023539
H	-9.432709	2.310134	0.795315	C	-9.850584	1.530478	0.345366	C	-9.942628	1.408369	0.405252
H	-10.129896	1.647790	-0.669105	H	-9.323682	2.393003	0.764566	H	-9.444444	2.282458	0.835634
C	-11.268657	1.241539	1.107645	H	-10.043422	1.770628	-0.706620	H	-10.140984	1.654644	-0.644224
H	-11.054323	1.034365	2.161487	C	-11.191772	1.379224	1.067712	C	-11.279982	1.205950	1.122133
C	-12.090415	2.526231	1.030235	H	-10.981323	1.158597	2.119610	H	-11.065804	0.973990	2.170830
H	-11.545961	3.372578	1.450244	C	-11.977375	2.687092	1.001460	C	-12.102303	2.491747	1.074944
H	-12.326714	2.762127	-0.009971	H	-11.405553	3.515706	1.420531	H	-11.558333	3.328160	1.514999
C	-7.005309	-1.892074	-0.575450	H	-12.213763	2.933896	-0.036290	H	-12.338463	2.752088	0.040553
H	-7.170384	-1.785147	-1.650845	C	-7.041374	-1.872627	-0.647375	C	-7.014816	-1.885047	-0.633593
H	-6.257648	-2.669635	-0.424772	H	-7.208771	-1.740257	-1.719599	H	-7.179685	-1.752774	-1.706197
H	-7.932590	-2.245950	-0.128072	H	-6.318020	-2.675869	-0.516497	H	-6.266839	-2.665622	-0.501174
C	-12.068110	0.068742	0.542731	H	-7.977174	-2.204049	-0.199867	H	-7.942043	-2.249826	-0.194945
H	-12.238618	0.208892	-0.527740	C	-12.025667	0.233559	0.496972	C	-12.078770	0.046493	0.529425
H	-11.556203	-0.882564	0.681176	H	-12.192360	0.384610	-0.572673	H	-12.249088	0.211861	-0.537472
H	-13.041887	-0.005357	1.027352	H	-11.540465	-0.732453	0.629385	H	-11.566465	-0.907594	0.645444
C	8.819861	2.737476	-0.602022	H	-13.000941	0.185004	0.982292	H	-13.052628	-0.039489	1.011914
H	9.709601	2.309277	-1.070633	C	8.953464	2.296687	-1.141229	C	8.808261	2.750912	-0.546678
H	8.371960	3.409047	-1.325529	H	9.783888	1.694667	-1.522116	H	9.698306	2.334321	-1.025070
H	9.137815	3.343868	0.250086	H	8.528782	2.825773	-1.987677	H	8.360227	3.439216	-1.254199
C	5.990714	2.569530	-1.581633	H	9.369423	3.049357	-0.466040	H	9.125738	3.337110	0.319622
H	4.937769	2.384624	-1.756416	C	6.159289	2.059445	-2.138282	C	5.979423	2.604931	-1.530689
H	6.103757	3.582014	-1.195675	H	5.124359	1.822819	-2.359200	H	4.926603	2.423742	-1.710060
H	6.510589	2.520142	-2.539182	H	6.239389	3.140344	-2.011869	H	6.091916	3.608048	-1.120850
C	8.143606	-1.363439	2.194208	H	6.764598	1.784531	-3.003034	H	6.499547	2.578450	-2.489010
H	8.209093	-0.917851	3.188900	C	7.876113	-0.795403	2.644133	C	8.133200	-1.415332	2.151552
H	7.567218	-2.280043	2.263054	H	7.448714	-0.351587	3.545954	H	8.198250	-0.993379	3.156526
H	9.158272	-1.611302	1.890314	H	7.554606	-1.836916	2.613702	H	7.557212	-2.333566	2.198543
O	9.569062	0.712121	1.204599	H	8.956953	-0.769707	2.738826	H	9.148051	-1.655479	1.842128
H	10.065502	1.461897	0.854268	O	9.604549	0.825209	1.180194	O	9.557951	0.683704	1.211692
H	5.425133	-1.628322	2.406955	H	10.137526	1.340002	0.572203	H	10.054134	1.441783	0.879326
H	3.164264	-1.958370	1.270142	H	5.211608	-0.795042	2.797980	H	5.414797	-1.686390	2.357297
C	3.998569	-1.785105	-1.391826	H	3.003094	-1.460312	1.655090	H	3.154348	-1.990453	1.212432
H	3.421245	-2.684677	-1.185297	C	4.066441	-1.895318	-0.914488	C	3.989205	-1.753867	-1.444484
H	3.776478	-1.453666	-2.404680	H	3.557968	-2.763129	-0.494428	H	3.412240	-2.658333	-1.259445
H	5.056463	-2.040005	-1.337697	H	3.859903	-1.850885	-1.983805	H	3.767205	-1.398650	-2.449265
H	-1.466127	-1.370788	0.620836	H	5.139034	-2.030872	-0.781376	H	5.047202	-2.009504	-1.396140
C	-1.476469	-2.568481	-1.155030	H	-1.447778	-1.447450	0.644730	H	-1.476154	-1.389741	0.576066
H	-2.363976	-3.143491	-0.898733	C	-1.599147	-2.843586	-0.965830	C	-1.485534	-2.545076	-1.227649
H	-1.460255	-2.441941	-2.240637	H	-2.449746	-3.360300	-0.522250	H	-2.372840	-3.126387	-0.985252
H	-0.611969	-3.168251	-0.870952	H	-1.719628	-2.848709	-2.051213	H	-0.620830	-3.151015	-0.957631
H	-13.032014	2.426292	1.570456	H	-0.701650	-3.411552	-0.721882	H	-13.043986	2.378629	1.612416
C	-0.233457	-0.388743	-0.823457	H	-12.917865	2.612261	1.548160	C	-0.243587	-0.373239	-0.844277
H	-0.252436	-0.164185	-1.895250	C	-0.229633	-0.753947	-0.931800	H	-0.262414	-0.123388	-1.910460
H	-0.286997	0.573389	-0.305799	H	-0.277388	-0.104241	-1.799098	H	-0.297686	0.576348	-0.304008
**α-Tocopherol (C11$^{\bullet}$)**				**α-Tocopherol (C12$^{\bullet}$)**				**α-Tocopherol (C13$^{\bullet}$)**			
C	3.624805	-0.697856	-0.389212	C	3.619428	-0.691656	-0.401159	C	-3.586040	-0.157797	0.255329
C	5.746517	0.483533	-0.147456	C	5.741147	0.488272	-0.152433	C	-5.884501	0.457024	-0.105392
C	6.217226	-0.473817	0.806184	C	6.211227	-0.473826	0.796728	C	-6.248862	-0.867108	-0.340107
C	5.263905	-1.513115	1.334544	C	5.257499	-1.515603	1.319441	C	-5.191102	-1.929570	-0.508617
C	3.818306	-1.122037	1.055843	C	3.812097	-1.122983	1.041890	C	-3.808912	-1.320043	-0.708437
C	6.561012	1.577848	-0.618616	C	6.556024	1.584800	-0.617749	C	-6.845833	1.464723	0.032405
C	7.518154	-0.393979	1.227119	C	7.511937	-0.396220	1.218756	C	-7.602056	-1.210763	-0.414682
H	5.493355	-2.482719	0.885644	H	5.487079	-2.483009	0.865888	H	-5.188681	-2.584795	0.368093
H	3.522150	-0.293165	1.702509	H	3.515688	-0.297275	1.692477	H	-3.715947	-0.929242	-1.724187
C	8.321839	0.676901	0.737690	C	8.316012	0.676964	0.735049	C	-8.553325	-0.209089	-0.280012

C	7.853263	1.662498	-0.173900	C	7.848045	1.667102	-0.171923	C	-8.194302	1.123261	-0.065307
O	4.547677	0.429976	-0.657202	O	4.542577	0.437373	-0.663084	O	-4.570946	0.850487	-0.020122
C	2.251748	-0.090105	-0.646720	C	2.246581	-0.082479	-0.656410	C	-2.261455	0.554174	-0.007744
H	2.247055	0.309621	-1.663766	H	2.242485	0.322259	-1.671473	H	-2.244422	1.451412	0.617285
H	2.122967	0.757798	0.030210	H	2.117533	0.762091	0.024625	H	-2.267854	0.897421	-1.045334
C	1.084242	-1.060678	-0.476712	C	1.078870	-1.053739	-0.491822	C	-1.004333	-0.270016	0.253040
H	1.232573	-1.932722	-1.115349	H	1.227445	-1.922640	-1.134671	H	-1.022108	-0.659295	1.272474
H	1.040758	-1.422653	0.554307	H	1.034785	-1.420789	0.537376	H	-0.975225	-1.136167	-0.414728
C	-1.487669	-1.206638	-0.462373	C	-1.493066	-1.199460	-0.479592	C	1.566320	-0.235022	0.082861
C	-2.744357	-0.398209	-0.799700	C	-2.749476	-0.389228	-0.813608	C	2.760326	0.705555	-0.124200
H	-2.793527	-0.258975	-1.885492	H	-2.646550	0.608806	-0.376787	H	2.840189	1.372948	0.742197
H	-2.641314	0.601978	-0.367862	C	-4.061235	-0.999260	-0.326512	H	2.542958	1.361797	-0.980892
C	-4.055779	-1.005985	-0.308891	H	-3.986903	-1.208464	0.746080	C	4.059046	0.008714	-0.338698
H	-3.980842	-1.209888	0.764679	H	-4.229687	-1.956659	-0.820453	H	4.060818	-0.873232	-0.971523
H	-4.224384	-1.965829	-0.798014	C	-5.250512	-0.075530	-0.578825	C	5.355318	0.701663	-0.097981
C	-5.245303	-0.083650	-0.565114	H	-5.022244	0.910668	-0.163326	H	5.481181	1.516598	-0.827433
H	-5.016928	0.904612	-0.154607	H	-5.373168	0.068359	-1.658316	H	5.330078	1.195170	0.882256
H	-5.368560	0.054899	-1.645234	C	-6.581503	-0.554169	0.009556	C	6.584621	-0.210324	-0.167693
C	-6.575918	-0.559537	0.026341	H	-6.438513	-0.698529	1.087198	H	6.566888	-0.716898	-1.139833
H	-6.432329	-0.698563	1.104605	C	-7.645823	0.531221	-0.179831	C	7.864887	0.624930	-0.095357
C	-7.640471	0.524782	-0.167821	H	-7.259997	1.470880	0.227142	H	7.823251	1.396311	-0.870337
H	-7.254537	1.466481	0.234304	H	-7.790409	0.699902	-1.253151	H	7.886132	1.155063	0.863745
H	-7.785657	0.688150	-1.241881	C	-8.996930	0.237113	0.467121	C	9.159079	-0.166595	-0.260287
C	-8.991193	0.233711	0.481306	H	-8.846183	-0.028402	1.519314	H	9.110418	-0.750639	-1.186041
H	-8.839845	-0.026594	1.534713	H	-9.453269	-0.630102	-0.011473	H	9.257099	-0.884446	0.555298
H	-9.447686	-0.635908	0.007241	C	-9.947865	1.427496	0.378264	C	10.389691	0.735806	-0.296553
C	-9.942317	1.423529	0.387093	H	-9.449525	2.309465	0.792070	H	10.230088	1.522934	-1.039701
H	-9.443859	2.307587	0.796274	H	-10.145980	1.653865	-0.675730	H	10.494713	1.242941	0.669490
H	-10.141029	1.644674	-0.667897	C	-11.285391	1.239059	1.098626	C	11.702871	0.019433	-0.620255
C	-11.279432	1.238491	1.109099	H	-11.071449	1.026984	2.151572	H	11.590028	-0.456258	-1.600254
H	-11.064895	1.031637	2.162963	C	-12.107369	2.523941	1.026907	C	12.846892	1.027252	-0.706157
C	-12.101602	2.522908	1.031489	H	-11.563255	3.368413	1.451107	H	12.639967	1.802728	-1.444466
H	-11.557359	3.369526	1.451224	H	-12.343287	2.764670	-0.012278	H	12.993326	1.515332	0.260228
H	-12.338110	2.758481	-0.008743	C	-7.020737	-1.885792	-0.597400	C	6.533473	-1.272328	0.929769
C	-7.015322	-1.894189	-0.573802	H	-7.185392	-1.773852	-1.672349	H	6.632748	-0.802131	1.911746
H	-7.180571	-1.787571	-1.649201	H	-6.272986	-2.663907	-0.450065	H	5.585005	-1.808939	0.911631
H	-6.267398	-2.671480	-0.423036	H	-7.948133	-2.241944	-0.152070	H	7.333245	-2.003991	0.824194
H	-7.942434	-2.248248	-0.126219	C	-12.084382	0.068771	0.527885	C	12.031529	-1.066145	0.402828
C	-12.078591	0.065309	0.544570	H	-12.254478	0.213910	-0.541986	H	12.079677	-0.636187	1.406591
H	-12.249283	0.205150	-0.525912	H	-11.572345	-0.883074	0.662075	H	11.285386	-1.859598	0.411724
H	-11.566368	-0.885803	0.683176	H	-13.058343	-0.007790	1.011751	H	12.999117	-1.521216	0.189079
H	-13.052281	-0.008978	1.029335	C	8.803534	2.746905	-0.595730	C	-9.289781	2.151592	0.061040
C	8.808394	2.740310	-0.603545	H	9.693550	2.321083	-1.065979	H	-9.933814	1.944580	0.920767
H	9.698207	2.312278	-1.072170	H	8.355798	3.421777	-1.316261	H	-8.893098	3.152672	0.192223
H	8.360188	3.411567	-1.327154	H	9.121018	3.349354	0.259346	H	-9.920811	2.169971	-0.831887
H	9.126269	3.347006	0.248377	C	5.974822	2.583009	-1.577283	C	-6.414495	2.888763	0.276757
C	5.979172	2.571244	-1.582747	H	4.921986	2.398721	-1.753366	H	-5.335424	2.951343	0.363762
H	4.926263	2.385967	-1.757348	H	6.087509	3.593693	-1.186531	H	-6.726257	3.539965	-0.541407
H	6.091949	3.583856	-1.197046	H	6.495100	2.538216	-2.534839	H	-6.854285	3.281417	1.194433
H	6.498938	2.521790	-2.540352	C	8.126934	-1.367218	2.180946	C	-8.020555	-2.637458	-0.647440
C	8.133787	-1.360147	2.193754	H	8.191925	-0.926290	3.177745	H	-7.815643	-2.943396	-1.675974
H	8.199264	-0.914301	3.188331	H	7.550698	-2.284247	2.245252	H	-7.473286	-3.316819	0.006376
H	7.557696	-2.276915	2.262894	H	9.141774	-1.613455	1.876310	H	-9.083255	-2.763527	-0.466479
H	9.148492	-1.607765	1.889787	O	9.552390	0.713240	1.201675	O	-9.874069	-0.577515	-0.372420
O	9.558465	0.715622	1.203463	H	10.048826	1.464748	0.855071	H	-10.431512	0.188213	-0.225992
H	10.054624	1.465469	0.852888	H	5.408426	-1.633624	2.391328	H	-5.443172	-2.567070	-1.357148
H	5.415425	-1.625830	2.406919	H	3.148089	-1.958773	1.252048	H	-3.036817	-2.076412	-0.570362
H	3.154512	-1.956858	1.270480	C	3.983454	-1.772856	-1.408734	C	-3.717324	-0.589249	1.714733
C	3.988415	-1.783969	-1.391639	H	3.406221	-2.673499	-1.206667	H	-3.083089	-1.449897	1.926007
H	3.411400	-2.683673	-1.184819	H	3.761714	-1.436710	-2.420112	H	-3.428128	0.230507	2.372088
H	3.766089	-1.452842	-2.404542	H	5.041376	-2.027802	-1.355366	H	-4.747324	-0.860432	1.943235
H	5.046397	-2.038525	-1.337586	H	-1.482150	-1.369050	0.603599	H	1.551843	-0.945025	-0.752211
C	-1.486346	-2.569003	-1.153942	C	-1.491528	-2.558398	-1.177872	C	1.723114	-1.022328	1.382539
H	-2.373639	-3.144229	-0.897391	H	-2.379027	-3.134777	-0.924641	H	2.696687	-1.510789	1.425468
H	-1.470313	-2.442717	-2.239581	H	-1.474892	-2.426761	-2.262866	H	1.648245	-0.348792	2.240515
H	-0.621622	-3.168435	-0.869833	H	-0.627027	-3.159327	-0.896255	H	0.957018	-1.789281	1.489336
H	-13.043100	2.422803	1.571857	H	-13.049171	2.421285	1.566266	H	13.783480	0.539987	-0.978525
C	-0.243973	-0.388796	-0.823052	C	-0.249079	-0.379998	-0.835561	C	0.260179	0.560892	0.047612
H	-0.263162	-0.164500	-1.894896	H	-0.267662	-0.150416	-1.906296	H	0.302671	1.342761	0.814391
H	-0.297747	0.573442	-0.305617	H	-0.303021	0.579683	-0.313416	H	0.191205	1.078941	-0.913573
**α-Tocopherol (C14$^{\bullet}$)**				**α-Tocopherol (C15$^{\bullet}$)**				**α-Tocopherol (C16$^{\bullet}$)**			
C	3.608713	-0.691545	-0.397531	C	-3.593330	-0.330137	0.326232	C	3.600917	-0.838635	-0.199056
C	5.730466	0.488717	-0.150677	C	-5.801190	0.545597	-0.062223	C	5.533270	0.591138	-0.072188
C	6.200157	-0.471380	0.800701	C	-6.214033	-0.639857	-0.667022	C	6.153795	-0.243645	0.833583
C	5.246166	-1.511940	1.325354	C	-5.207422	-1.709658	-1.010019	C	5.395256	-1.397645	1.473775

C	3.800888	-1.119783	1.046504	C	-3.779924	-1.183859	-0.924503	C	3.886478	-1.284414	1.229594
C	6.545593	1.584164	-0.618096	C	-6.711037	1.563677	0.244005	C	6.193212	1.661516	-0.689062
C	7.500741	-0.392988	1.222969	C	-7.568942	-0.837578	-0.949975	C	7.498426	-0.002964	1.161756
H	5.475797	-2.480341	0.873956	H	-5.333491	-2.560594	-0.333097	H	5.809052	-2.347611	1.128153
H	3.504352	-0.292648	1.695219	H	-3.559886	-0.555417	-1.790463	H	3.481960	-0.512987	1.885286
C	8.305070	0.679077	0.737207	C	-8.469326	0.173179	-0.644250	C	8.146137	1.079139	0.590961
C	7.837481	1.667304	-0.172041	C	-8.058991	1.372591	-0.058227	C	7.512415	1.920068	-0.338031
O	4.532052	0.436831	-0.661595	O	-4.484780	0.792584	0.243070	O	4.218040	0.423395	-0.409146
C	2.236005	-0.082790	-0.654524	C	-2.209122	0.312892	0.375201	C	2.121600	-0.488000	-0.382867
H	2.232267	0.319763	-1.670457	H	-2.207874	1.023018	1.206646	H	2.017545	0.078778	-1.309937
H	2.106823	0.763256	0.024651	H	-2.081624	0.900050	-0.537785	H	1.861234	0.197722	0.426333
C	1.068150	-1.053584	-0.488213	C	-1.039038	-0.653819	0.533842	C	1.099766	-1.616584	-0.387080
H	1.216845	-1.923880	-1.129145	H	-1.185642	-1.264054	1.426674	H	1.339720	-2.329870	-1.177025
H	1.023707	-1.418414	0.541758	H	-0.999049	-1.341452	-0.315926	H	1.139241	-2.156550	0.566086
C	-1.503803	-1.199037	-0.476480	C	1.535171	-0.804565	0.594287	C	-1.539652	-1.869598	-0.409217
C	-2.760032	-0.389407	-0.812634	C	2.796798	0.061863	0.659170	C	-2.710684	-0.968959	-0.796680
H	-2.808242	-0.247145	-1.898076	H	2.841770	0.545242	1.641737	H	-2.749064	-0.955006	-1.890570
H	-2.657148	0.609554	-0.377929	H	2.703700	0.868986	-0.074020	H	-2.486189	0.061019	-0.501250
C	-4.072001	-0.998266	-0.324638	C	4.104995	-0.680754	0.402224	C	-4.089310	-1.332336	-0.260222
H	-3.998026	-1.205168	0.748424	H	4.042161	-1.201662	-0.559079	H	-4.107864	-1.302048	0.834905
H	-4.240388	-1.956711	-0.816571	H	4.262259	-1.449875	1.160786	H	-4.331623	-2.353647	-0.559522
C	-5.261111	-0.074970	-0.579314	C	5.309988	0.254320	0.388908	C	-5.170476	-0.414930	-0.857623
H	-5.032881	0.912099	-0.165867	H	5.150217	1.041132	-0.353887	H	-4.848986	0.628004	-0.794122
C	-6.592332	-0.552217	0.009677	H	5.392418	0.755422	1.359628	H	-5.204505	-0.652275	-1.937967
H	-6.449696	-0.694270	1.087673	C	6.642970	-0.448689	0.087123	C	-6.516191	-0.535254	-0.221877
C	-7.656491	0.532863	-0.182381	H	6.547725	-0.955564	-0.878774	C	-7.514487	0.569762	-0.432531
H	-7.270704	1.473359	0.222690	C	7.766932	0.597001	-0.041005	H	-7.021827	1.543496	-0.301910
H	-7.800723	0.699246	-1.256107	H	7.457649	1.355910	-0.765915	H	-7.859579	0.579037	-1.474054
C	-9.007829	0.240275	0.464778	H	7.875701	1.109971	0.920563	C	-8.769602	0.492823	0.456953
H	-8.857438	-0.022989	1.517587	C	9.113782	0.016961	-0.459384	H	-8.496556	0.546709	1.518161
H	-9.464099	-0.627925	-0.012092	H	8.999927	-0.513605	-1.410972	H	-9.232707	-0.484493	0.309735
C	-9.958623	1.430553	0.373060	H	9.425609	-0.727314	0.276162	C	-9.729801	1.628034	0.174796
H	-9.460330	2.313364	0.785123	C	10.187695	1.091125	-0.604323	H	-9.188735	2.562110	0.376089
H	-10.156385	1.654672	-0.681481	H	9.815253	1.876653	-1.269079	H	-10.000845	1.649746	-0.887951
C	-11.296394	1.243793	1.093404	H	10.354840	1.567425	0.368616	C	-10.999285	1.640880	1.018231
H	-11.082803	1.033964	2.146872	C	11.530901	0.591647	-1.142191	H	-10.682009	1.569718	2.065781
C	-12.118228	2.528594	1.018662	H	11.352240	0.142740	-2.125064	C	-11.692438	2.992481	0.817566
H	-11.574167	3.373926	1.441214	C	12.496203	1.761485	-1.319053	H	-10.987041	3.825589	0.883548
H	-12.353796	2.767109	-0.021113	H	12.081920	2.521338	-1.982527	H	-12.147228	3.046211	-0.178284
C	-7.031502	-1.885102	-0.594550	H	12.698877	2.232875	-0.354545	C	-6.967524	-1.885387	0.214489
H	-7.195808	-1.775460	-1.669789	C	6.964618	-1.464484	1.132334	H	-7.331014	-2.523966	-0.598566
H	-6.283870	-2.662968	-0.445305	H	6.999364	-1.161891	2.171051	H	-6.143763	-2.424042	0.675386
H	-7.959072	-2.240207	-0.148746	H	7.236346	-2.480047	0.891718	H	-7.785350	-1.822332	0.926555
C	-12.095316	0.072354	0.524933	C	12.148969	-0.472632	-0.237065	C	-11.954061	0.502472	0.670909
H	-12.265062	0.215206	-0.545302	H	12.268043	-0.082552	0.776960	H	-12.311280	0.631776	-0.351844
H	-11.583411	-0.879249	0.661332	H	11.533608	-1.369677	-0.182856	H	-11.482018	-0.475139	0.724496
H	-13.069436	-0.003074	1.008656	H	13.134496	-0.765948	-0.599771	H	-12.815885	0.517691	1.336965
C	8.793205	2.746103	-0.597871	C	-9.100557	2.422367	0.235501	C	8.305535	2.993465	-1.019950
H	9.683329	2.319187	-1.066922	H	-9.844701	2.056869	0.949484	H	9.130894	2.541753	-1.575381
H	8.345760	3.419465	-1.319994	H	-8.664789	3.319615	0.661841	H	7.690416	3.549766	-1.726722
H	9.110478	3.350361	0.256006	H	-9.629624	2.721028	-0.673615	H	8.721544	3.729616	-0.325823
C	5.964788	2.580360	-1.579958	C	-6.226097	2.838965	0.886003	C	5.465522	2.517761	-1.703402
H	4.911989	2.395792	-1.755976	H	-5.153739	2.804712	1.042187	H	4.468142	2.126385	-1.863210
H	6.077446	3.591872	-1.191348	H	-6.450516	3.703999	0.260270	H	5.377556	3.552443	-1.369340
H	6.485362	2.533458	-2.537252	H	-6.706244	3.000002	1.852378	H	5.988711	2.520303	-2.663637
C	8.115344	-1.361971	2.187441	C	-8.042778	-2.117692	-1.583821	C	8.218309	-0.907220	2.129813
H	8.180063	-0.918904	3.183309	H	-7.753864	-2.164302	-2.636353	H	7.843627	-0.741420	3.147431
H	7.539001	-2.278805	2.253539	H	-7.604197	-2.984749	-1.089661	H	8.045657	-1.955968	1.877951
H	9.130256	-1.608958	1.883655	H	-9.123884	-2.198369	-1.530152	H	9.287087	-0.715846	2.133290
O	9.541304	0.716241	1.204143	O	-9.791306	-0.047315	-0.948574	O	9.455730	1.262346	0.950558
H	10.037920	1.466955	0.856078	H	-10.319579	0.688137	-0.634548	H	9.816693	2.043661	0.525195
H	5.396744	-1.627668	2.397540	H	-5.403416	-2.092971	-2.012298	H	5.569756	-1.347759	2.554032
H	3.136735	-1.955056	1.258252	H	-3.070837	-2.010994	-0.925076	H	3.368287	-2.219665	1.454270
C	3.972954	-1.774945	-1.402662	C	-3.900164	-1.108975	1.604145	C	4.151705	-1.870848	-1.190126
H	3.395573	-2.675097	-1.198838	H	-3.342474	-2.044787	1.632844	H	3.853621	-2.896600	-0.967302
H	3.751565	-1.440956	-2.414831	H	-3.631407	-0.513063	2.476250	H	3.839368	-1.646863	-2.209305
H	5.030835	-2.029875	-1.348412	H	-4.962400	-1.341566	1.665748	H	5.236932	-1.809836	-1.111176
H	-1.493244	-1.366296	0.607078	H	1.537887	-1.330089	-0.368111	H	-1.634379	-2.123773	0.650497
C	-1.502174	-2.559474	-1.171832	C	1.513423	-1.849441	1.709446	C	-1.487493	-3.145973	-1.249121
H	-2.389807	-3.135224	-0.917641	H	2.409499	-2.467821	1.698352	H	-2.478192	-3.586120	-1.376502
H	-1.485184	-2.430175	-2.257102	H	1.455085	-1.357614	2.684221	H	-1.086315	-2.927756	-2.244066
H	-0.637818	-3.159877	-0.888651	H	0.657485	-2.516985	1.619352	H	-0.846505	-3.891973	-0.788438
H	-13.060209	2.427188	1.557943	H	13.448113	1.429526	-1.734557	H	-12.485773	3.164173	1.548045
C	-0.259627	-0.380459	-0.833820	C	0.291784	0.088610	0.634932	C	-0.280827	-0.994671	-0.593855
H	-0.277851	-0.153181	-1.905053	H	0.306365	0.674146	1.560935	H	-0.326647	-0.557780	-1.597733

H	-0.313642	0.580348	-0.313759	H	0.355686	0.811187	-0.184004	H	-0.368395	-0.145170	0.084924
**α-Tocopherol (C17•)**				**α-Tocopherol (C18•)**				**α-Tocopherol (C19•)**			
C	-3.587236	-0.348638	0.305292	C	3.593449	-0.691631	-0.391507	C	3.563177	-0.408366	-0.246121
C	-5.783872	0.579431	-0.019099	C	5.715624	0.488672	-0.148511	C	5.765671	0.536576	-0.026930
C	-6.231688	-0.580938	-0.646747	C	6.187237	-0.471660	0.801677	C	6.147755	-0.447479	0.882076
C	-5.253335	-1.661471	-1.035831	C	5.234334	-1.512413	1.327924	C	5.120731	-1.389369	1.460291
C	-3.813448	-1.167584	-0.962421	C	3.788489	-1.120253	1.052029	C	3.701546	-0.904599	1.190286
C	-6.666749	1.606999	0.330940	C	6.529773	1.584290	-0.617235	C	6.694685	1.436943	-0.561254
C	-7.595073	-0.743174	-0.910987	C	7.488646	-0.393317	1.221404	C	7.490390	-0.562898	1.254631
H	-5.385055	-2.527514	-0.379443	H	5.463124	-2.480679	0.875810	H	5.263400	-2.389031	1.037705
H	-3.594780	-0.522896	-1.816733	H	3.493190	-0.293311	1.701552	H	3.460615	-0.069973	1.852364
C	-8.468531	0.277945	-0.563995	C	8.291962	0.678922	0.734351	C	8.409098	0.333359	0.725873
C	-8.023280	1.451901	0.047337	C	7.822533	1.667373	-0.173703	C	8.028921	1.335365	-0.169491
O	-4.457418	0.792399	0.267020	O	4.516208	0.436865	-0.657082	O	4.461997	0.696290	-0.429488
C	-2.189870	0.266254	0.343846	C	2.220206	-0.082876	-0.645629	C	2.188081	0.202708	-0.506877
H	-2.155739	0.946846	1.198974	H	2.214446	0.319954	-1.661444	H	2.233588	0.701766	-1.478765
H	-2.072144	0.882634	-0.550997	H	2.092319	0.762978	0.034029	H	2.024329	0.985061	0.238521
C	-1.036013	-0.727603	0.440904	C	1.052731	-1.053775	-0.477284	C	1.017788	-0.776056	-0.496506
H	-1.180219	-1.372833	1.308993	H	1.200207	-1.923888	-1.118745	H	1.182227	-1.547408	-1.250163
H	-1.023839	-1.378634	-0.437959	H	1.010335	-1.418889	0.552673	H	0.956325	-1.285569	0.469618
C	1.535731	-0.930522	0.459513	C	-1.519187	-1.199363	-0.460524	C	-1.553274	-0.948546	-0.644669
C	2.813575	-0.086825	0.508478	C	-2.776117	-0.389706	-0.793981	C	-2.811369	-0.085945	-0.789554
H	2.871470	0.407125	1.485103	H	-2.826473	-0.247149	-1.879288	H	-2.812788	0.371634	-1.785508
H	2.731760	0.712904	-0.233774	H	-2.672428	0.609142	-0.359207	H	-2.750076	0.739736	-0.074349
C	4.108375	-0.855880	0.259071	C	-4.087092	-0.998765	-0.303569	C	-4.129567	-0.825290	-0.574878
H	4.036736	-1.387446	-0.696177	H	-4.010992	-1.205957	0.769289	H	-4.093354	-1.359984	0.380678
H	4.237015	-1.615707	1.030249	H	-4.256399	-1.957084	-0.795432	H	-4.253745	-1.581349	-1.350355
C	5.328132	0.060705	0.240222	C	-5.276748	-0.075461	-0.555649	C	-5.330936	0.116696	-0.580835
H	5.149096	0.875425	-0.467578	H	-5.047753	0.911507	-0.142382	H	-5.149327	0.921301	0.137879
H	5.447584	0.527498	1.223678	H	-5.401185	0.066405	-1.635203	H	-5.410113	0.595627	-1.563458
C	6.647316	-0.624072	-0.136746	C	-6.606782	-0.552937	0.035832	C	-6.673042	-0.541513	-0.244035
H	6.506949	-1.083797	-1.129276	H	-6.462015	-0.695277	1.113506	H	-6.570803	-1.024898	0.734895
C	7.744496	0.387358	-0.239763	C	-7.671372	0.532141	-0.153832	C	-7.756411	0.534024	-0.123681
H	7.470556	1.414634	-0.451656	H	-7.284836	1.472546	0.250736	H	-7.424594	1.289399	0.593595
C	9.179030	0.001570	-0.362843	H	-7.817727	0.698811	-1.227226	H	-7.856691	1.046064	-1.087031
H	9.301108	-0.736937	-1.171013	C	-9.021418	0.239307	0.495908	C	-9.129606	0.021706	0.312114
H	9.501880	-0.517957	0.546039	H	-9.478582	-0.628786	0.019699	H	-9.025332	-0.591578	1.215832
C	10.097386	1.192852	-0.624902	C	-9.972451	1.429561	0.406388	H	-9.524313	-0.656387	-0.456575
H	9.702538	1.762055	-1.471329	H	-9.473391	2.312285	0.817710	C	-10.104432	1.119167	0.568233
H	10.065626	1.864047	0.240260	H	-10.172301	1.653958	-0.647700	H	-10.032261	2.020980	-0.031100
C	11.554377	0.827879	-0.916045	C	-11.308790	1.242535	1.129315	C	-11.362962	0.880669	1.336559
H	11.568367	0.198596	-1.812269	H	-11.093114	1.032430	2.182303	H	-11.099297	0.358763	2.264474
C	12.363535	2.089941	-1.205577	C	-12.130835	2.527315	1.056543	C	-12.053626	2.194281	1.695914
H	11.928899	2.658734	-2.028226	H	-11.585986	3.372560	1.478254	H	-11.395924	2.837563	2.280062
H	12.383487	2.736229	-0.325023	H	-12.368464	2.766102	0.017299	H	-12.332924	2.732241	0.787594
C	7.007041	-1.754991	0.833411	C	-7.047073	-1.885679	-0.567893	C	-7.050788	-1.610996	-1.268364
H	7.117136	-1.360938	1.846299	H	-7.213502	-1.775752	-1.642776	H	-7.184205	-1.157667	-2.254324
H	6.238069	-2.525596	0.845407	H	-6.299110	-2.663547	-0.420334	H	-6.281979	-2.377514	-1.352482
H	7.945057	-2.232186	0.552786	H	-7.973745	-2.240954	-0.120360	H	-7.979316	-2.113453	-1.001820
C	12.190046	0.041073	0.228882	C	-12.108772	0.071211	0.562098	C	-12.324599	-0.035423	0.559337
H	12.121665	0.607113	1.161394	H	-12.280632	0.214347	-0.507761	H	-12.618831	0.439467	-0.378547
H	11.704857	-0.921919	0.383166	H	-11.596551	-0.880402	0.697229	H	-11.857529	-0.991459	0.323830
H	13.245463	-0.147108	0.029631	H	-13.081933	-0.004399	1.047719	H	-13.226838	-0.231832	1.140412
C	-9.036951	2.514610	0.388004	C	8.777362	2.746338	-0.601120	C	9.087212	2.278078	-0.683507
H	-9.781815	2.142196	1.097588	H	9.666581	2.319595	-1.072040	H	9.857678	1.745361	-1.248735
H	-8.575493	3.386245	0.839764	H	8.328461	3.419874	-1.322176	H	8.673007	3.033834	-1.342337
H	-9.568447	2.856540	-0.504419	H	9.096285	3.350378	0.252296	H	9.580916	2.803583	0.138594
C	-6.144630	2.854009	0.998849	C	5.947023	2.580718	-1.577679	C	6.242682	2.495184	-1.535781
H	-5.071397	2.791520	1.140113	H	4.893889	2.396145	-1.751673	H	5.187221	2.382516	-1.757424
H	-6.357212	3.739720	0.398399	H	6.060396	3.592130	-1.189015	H	6.398206	3.495908	-1.129352
H	-6.609124	3.000671	1.975113	H	6.465714	2.534104	-2.536010	H	6.797740	2.431239	-2.472390
C	-8.106823	-1.996270	-1.568974	C	8.105197	-1.362532	2.184398	C	7.930240	-1.631016	2.219363
H	-7.837368	-2.021892	-2.627378	H	8.171855	-0.919734	3.180258	H	7.578447	-1.415250	3.230673
H	-7.678693	-2.884833	-1.104790	H	7.529032	-2.279414	2.251381	H	7.525015	-2.603315	1.937548
H	-9.188324	-2.055554	-1.498069	H	9.119521	-1.609384	1.878546	H	9.012759	-1.702984	2.249419
O	-9.799700	0.093787	-0.852045	O	9.529111	0.716022	1.198862	O	9.717868	0.204173	1.125206
H	-10.305493	0.836028	-0.517752	H	10.025003	1.466856	0.850025	H	10.259797	0.844290	0.661522
H	-5.475110	-2.013495	-2.044153	H	5.387030	-1.628426	2.399780	H	5.280411	-1.493873	2.534226
H	-3.122521	-2.009381	-0.995414	H	3.124798	-1.955618	1.264856	H	2.983780	-1.701153	1.383474
C	-3.885446	-1.153259	1.569200	C	3.955764	-1.774737	-1.397650	C	3.902699	-1.494541	-1.264853
H	-3.340878	-2.097188	1.567630	H	3.378831	-2.674975	-1.192936	H	3.341323	-2.406911	-1.063377
H	-3.595025	-0.581477	2.450460	H	3.732364	-1.440483	-2.409290	H	3.662480	-1.149532	-2.270484
H	-4.949797	-1.372402	1.642308	H	5.013763	-2.029627	-1.345554	H	4.965071	-1.733006	-1.232618
H	1.503788	-1.436789	-0.512708	H	-1.506485	-1.366917	0.622965	H	-1.560085	-1.381671	0.362791
C	1.521664	-1.996980	1.554221	C	-1.518859	-2.559610	-1.156250	C	-1.534068	-2.093490	-1.656424

H	2.386162	-2.655391	1.487102	H	-2.405960	-3.135474	-0.900469	H	-2.400121	-2.743706	-1.543963
H	1.534090	-1.524266	2.540117	H	-1.504014	-2.430013	-2.241516	H	-1.540466	-1.696702	-2.675109
H	0.633026	-2.623729	1.494173	H	-0.653917	-3.160046	-0.874936	H	-0.646772	-2.714610	-1.543652
H	13.394516	1.848475	-1.465528	H	-13.071747	2.425713	1.597652	H	-12.961892	2.015678	2.271777
C	0.310702	-0.015530	0.549827	C	-0.275759	-0.380624	-0.820091	C	-0.307382	-0.065832	-0.766202
H	0.349874	0.535412	1.496071	H	-0.296105	-0.153054	-1.891223	H	-0.281931	0.373877	-1.769548
H	0.377892	0.734998	-0.243347	H	-0.328799	0.580039	-0.299661	H	-0.403446	0.770845	-0.068058
**α-Tocopherol (C20$^{\bullet}$)**				**α-Tocopherol (C21$^{\bullet}$)**				**α-Tocopherol (C22$^{\bullet}$)**			
C	3.560820	-0.336825	-0.303668	C	3.577140	-0.691065	-0.393737	C	3.558254	-0.361928	-0.346919
C	5.771637	0.545087	0.047655	C	5.702696	0.482577	-0.148043	C	5.765683	0.532826	0.000686
C	6.158790	-0.589014	0.759064	C	6.170175	-0.479352	0.802573	C	6.113078	-0.526612	0.836327
C	5.132370	-1.612145	1.177505	C	5.213402	-1.517198	1.327537	C	5.061598	-1.498514	1.310417
C	3.714328	-1.077183	1.022045	C	3.769118	-1.120491	1.049971	C	3.655692	-0.974225	1.047679
C	6.699468	1.522974	-0.327098	C	6.520819	1.575712	-0.615642	C	6.718540	1.460492	-0.435148
C	7.506064	-0.777761	1.081224	C	7.471321	-0.405127	1.223864	C	7.443746	-0.688474	1.233332
H	5.255912	-2.521579	0.581097	H	5.439709	-2.486111	0.875561	H	5.202677	-2.462777	0.811976
H	3.492209	-0.369391	1.823803	H	3.475626	-0.292720	1.699257	H	3.408427	-0.192954	1.770059
C	8.424249	0.193580	0.708391	C	8.278559	0.664666	0.737920	C	8.385587	0.236117	0.804617
C	8.038927	1.344549	0.017368	C	7.813301	1.654701	-0.170556	C	8.040792	1.310619	-0.018535
O	4.463930	0.779224	-0.302478	O	4.503732	0.434581	-0.658052	O	4.474279	0.741270	-0.420266
C	2.184362	0.310844	-0.438380	C	2.206108	-0.077994	-0.649413	C	2.194896	0.282435	-0.589510
H	2.210097	0.952549	-1.323350	H	2.202817	0.324990	-1.665177	H	2.254312	0.830813	-1.533616
H	2.038128	0.970032	0.421104	H	2.080049	0.768162	0.030211	H	2.034139	1.026785	0.194438
C	1.011979	-0.659556	-0.551783	C	1.035408	-1.045268	-0.482597	C	1.014372	-0.683275	-0.635630
H	1.171227	-1.325868	-1.400975	H	1.180935	-1.915750	-1.124003	H	1.175789	-1.419215	-1.424680
H	0.957327	-1.291792	0.339232	H	0.990644	-1.410388	0.547258	H	0.945356	-1.238811	0.303981
C	-1.558375	-0.818398	-0.673499	C	-1.536970	-1.182832	-0.468927	C	-1.552827	-0.840530	-0.762197
C	-2.821612	0.044391	-0.759113	C	-2.790967	-0.369212	-0.803771	C	-2.816441	0.022249	-0.838501
H	-2.843717	0.542687	-1.735018	H	-2.839583	-0.226351	-1.889117	H	-2.853071	0.511724	-1.818420
H	-2.750514	0.840170	-0.011510	H	-2.684682	0.629249	-0.368734	H	-2.734518	0.824221	-0.098805
C	-4.132043	-0.709941	-0.549011	C	-4.104421	-0.974244	-0.315009	C	-4.122700	-0.731926	-0.602918
H	-4.084641	-1.259632	0.397398	H	-4.030249	-1.181818	0.757910	H	-4.054143	-1.286637	0.339478
H	-4.255242	-1.454500	-1.335560	H	-4.276130	-1.931963	-0.807207	H	-4.263232	-1.472649	-1.390347
C	-5.342991	0.219562	-0.532297	C	-5.290889	-0.047197	-0.568380	C	-5.333059	0.197013	-0.553231
H	-5.159935	1.022809	0.187684	H	-5.059309	0.938994	-0.154702	H	-5.131045	1.000584	0.161331
H	-5.441347	0.702126	-1.511394	H	-5.413594	0.095203	-1.648062	H	-5.458708	0.679254	-1.529373
C	-6.672780	-0.455157	-0.179639	C	-6.623111	-0.520601	0.021447	C	-6.651174	-0.479775	-0.163221
H	-6.557946	-0.925103	0.804343	H	-6.480076	-0.663540	1.099273	H	-6.509357	-0.944264	0.819922
C	-7.774522	0.603455	-0.067317	C	-7.684083	0.567819	-0.169336	C	-7.754689	0.574122	-0.027080
H	-7.438588	1.383243	0.622866	H	-7.295098	1.506958	0.235824	H	-7.396214	1.370264	0.632715
H	-7.896637	1.089578	-1.042032	H	-7.828636	0.735090	-1.242881	H	-7.920327	1.039453	-1.005435
C	-9.127865	0.079995	0.404767	C	-9.035811	0.279111	0.478751	C	-9.082610	0.052223	0.514423
H	-9.002095	-0.467896	1.344151	H	-8.885412	0.014949	1.531334	H	-8.906871	-0.477650	1.456697
H	-9.522572	-0.631304	-0.322107	H	-9.495113	-0.587487	0.001876	H	-9.512664	-0.678118	-0.173846
C	-10.142242	1.204726	0.608285	C	-9.983018	1.472339	0.388262	C	-10.097535	1.166378	0.749900
H	-9.712951	1.963643	1.270008	H	-9.481698	2.353445	0.800303	H	-9.672463	1.907104	1.433105
H	-10.289139	1.714599	-0.359293	H	-10.180909	1.697502	-0.666032	H	-10.291460	1.690091	-0.192972
C	-11.471580	0.781701	1.151893	C	-11.320797	1.289385	1.109568	C	-11.431282	0.672105	1.318627
C	-12.387348	1.860203	1.626472	H	-11.107035	1.078465	2.162783	H	-11.225302	0.132926	2.249256
H	-11.845911	2.634931	2.170959	C	-12.138742	2.576734	1.035994	C	-12.342941	1.863085	1.662657
H	-12.890937	2.356470	0.783788	H	-11.591761	3.420217	1.458473	H	-11.860048	2.524378	2.383891
C	-7.038930	-1.542676	-1.189073	C	-7.066838	-1.851882	-0.582989	H	-12.562933	2.439760	0.762811
H	-7.170667	-1.104939	-2.182311	H	-7.231640	-1.741289	-1.658055	C	-7.039990	-1.573567	-1.157176
H	-6.264876	-2.305365	-1.258105	H	-6.321482	-2.632100	-0.434646	H	-7.182252	-1.143329	-2.152211
H	-7.965365	-2.046662	-0.918725	H	-7.995147	-2.204324	-0.136612	H	-6.272825	-2.342896	-1.230601
C	-12.082238	-0.495897	0.678811	C	-12.123751	0.120640	0.541233	H	-7.966901	-2.066429	-0.868659
H	-12.350505	-0.441987	-0.386927	H	-12.293887	0.264457	-0.528811	C	-12.120037	-0.255011	0.374795
H	-11.402029	-1.341401	0.786557	H	-11.614664	-0.832585	0.676842	H	-12.107946	-0.044791	-0.686566
H	-12.995418	-0.723021	1.228527	H	-13.097723	0.048001	1.025681	H	-12.819329	-0.995981	0.732770
C	9.096791	2.357406	-0.340483	C	8.772002	2.730739	-0.596682	C	9.123130	2.275136	-0.432294
H	9.839926	1.936064	-1.023989	H	9.660447	2.301288	-1.066600	H	9.910322	1.771782	-1.001376
H	8.674701	3.229950	-0.827522	H	8.326066	3.405770	-1.318179	H	8.738817	3.073868	-1.057601
H	9.624556	2.709995	0.549950	H	9.091789	3.333665	0.257198	H	9.589687	2.744023	0.438484
C	6.242508	2.742875	-1.086550	C	5.942328	2.574084	-1.576642	C	6.305421	2.598035	-1.334637
H	5.177074	2.694822	-1.281632	H	4.888832	2.392822	-1.751918	H	5.252446	2.525016	-1.582743
H	6.442835	3.655278	-0.522801	H	6.058392	3.585084	-1.187701	H	6.473834	3.562273	-0.852432
H	6.761136	2.827660	-2.042466	H	6.462014	2.525983	-2.534359	H	6.876915	2.591734	-2.263556
C	7.952383	-2.006353	1.827026	C	8.083693	-1.376392	2.187458	C	7.846021	-1.837223	2.118749
H	7.632363	-1.969801	2.870865	H	8.150544	-0.933940	3.183458	H	7.460990	-1.704901	3.132215
H	7.522346	-2.907317	1.389044	H	7.504591	-2.291481	2.253625	H	7.445950	-2.779250	1.742366
H	9.033869	-2.096357	1.811424	H	9.097607	-1.626367	1.882782	H	8.926567	-1.919828	2.178439
O	9.738431	-0.015884	1.052049	O	9.515263	0.697842	1.203912	O	9.681403	0.059422	1.227354
H	10.279662	0.688861	0.693064	H	10.013911	1.447173	0.855772	H	10.240357	0.733403	0.837556
H	5.308232	-1.907144	2.212880	H	5.364455	-1.633833	2.399558	H	5.193325	-1.691907	2.375797
H	2.992604	-1.890838	1.085908	H	3.102570	-1.953810	1.261889	H	2.925775	-1.774991	1.159696

C	3.883311	-1.229919	-1.500811	C	3.937275	-1.775161	-1.399598	C	3.911167	-1.367883	-1.440795
H	3.317533	-2.160418	-1.456712	H	3.357292	-2.673621	-1.195698	H	3.327888	-2.282263	-1.333016
H	3.636206	-0.712176	-2.427448	H	3.716127	-1.440073	-2.411457	H	3.710191	-0.935813	-2.421063
H	4.944195	-1.476015	-1.521434	H	4.994410	-2.033358	-1.346275	H	4.967621	-1.629797	-1.395294
H	-1.567590	-1.328711	0.297091	H	-1.526084	-1.350573	0.614553	H	-1.535479	-1.318374	0.224785
C	-1.525329	-1.881349	-1.771331	C	-1.540055	-2.542979	-1.164842	C	-1.555954	-1.939611	-1.824258
H	-2.407927	-2.518165	-1.742626	H	-2.429253	-3.116108	-0.910200	H	-2.412383	-2.602886	-1.714363
H	-1.484712	-1.405268	-2.754806	H	-1.523509	-2.413282	-2.250071	H	-1.598706	-1.497403	-2.823238
H	-0.655856	-2.530332	-1.677104	H	-0.677327	-3.146150	-0.882580	H	-0.660460	-2.556749	-1.768533
H	-13.171296	1.465783	2.274308	H	-13.080611	2.477995	1.575965	H	-13.288633	1.524115	2.084474
C	-0.315763	0.076010	-0.719385	C	-0.290564	-0.367929	-0.826895	C	-0.307574	0.045020	-0.870610
H	-0.307246	0.621543	-1.669549	H	-0.308921	-0.140151	-1.898019	H	-0.291230	0.518430	-1.858583
H	-0.401582	0.832370	0.066369	H	-0.341227	0.592824	-0.306395	H	-0.393332	0.857311	-0.142919

Structures of α-Tocopherol and their corresponding species in the SPLET (SP_step) mechanism at M06-2X/6-311++G(2d,2p) level of theory in gas phase.

**M06-2X/6-311++G(2d,2p)**											
**α-Tocopherol**				**α-Tocopherol (O^-)**				**α-Tocopherol ($C1^-$)**			
C	-3.60970400	-0.36270000	0.33903600	C	3.650445	-0.258197	-0.254196	C	3.647004	-0.353181	-0.334272
C	-5.81748200	0.53355700	-0.00151900	C	5.868261	0.581577	0.112110	C	5.852934	0.559640	-0.027401
C	-6.16752600	-0.52451700	-0.83776200	C	6.258460	-0.579739	0.775095	C	6.206159	-0.467574	0.845190
C	-5.11767600	-1.49608300	-1.31623600	C	5.223866	-1.612105	1.161087	C	5.158999	-1.424174	1.358450
C	-3.71080600	-0.97292100	-1.05621200	C	3.801208	-1.079869	1.027148	C	3.750685	-0.913757	1.081465
C	-6.76881700	1.46077900	0.43861000	C	6.816885	1.532479	-0.278917	C	6.801716	1.473041	-0.500693
C	-7.49939500	-0.68549900	-1.23106800	C	7.605303	-0.815839	1.049230	C	7.538725	-0.611527	1.242715
H	-5.25793900	-2.46108800	-0.81899900	H	5.343281	-2.502462	0.532398	H	5.301138	-2.405726	0.895303
H	-3.46489800	-0.19071100	-1.77802800	H	3.572294	-0.413845	1.863220	H	3.503516	-0.107241	1.775570
C	-8.43975700	0.23867400	-0.79811900	C	8.618604	0.130442	0.686653	C	8.476547	0.299054	0.776716
C	-8.09230100	1.31187200	0.02560000	C	8.159703	1.314616	0.009530	C	8.125884	1.341829	-0.083893
O	-4.52481200	0.74098900	0.41600000	O	4.519800	0.860558	-0.171487	O	4.559438	0.749255	-0.450792
C	-2.24544700	0.28052700	0.57950700	C	2.258625	0.367996	-0.361307	C	2.281035	0.278013	-0.595973
H	-2.30287600	0.82823400	1.52412900	H	2.285950	1.075868	-1.195021	H	2.336400	0.792319	-1.559309
H	-2.08556700	1.02539200	-0.20413000	H	2.088730	0.957859	0.543525	H	2.120033	1.049580	0.161149
C	-1.06541800	-0.68588400	0.62308000	C	1.104217	-0.609012	-0.564483	C	1.103259	-0.692084	-0.604502
H	-1.22688100	-1.42321600	1.41080400	H	1.285027	-1.201139	-1.463349	H	1.265823	-1.456263	-1.365976
H	-0.99698700	-1.23964500	-0.31762900	H	1.054876	-1.313070	0.271596	H	1.036903	-1.212604	0.355145
C	1.50199800	-0.84399800	0.75121400	C	-1.467207	-0.798363	-0.687913	C	-1.463879	-0.860595	-0.724699
C	2.76535300	0.01886100	0.83151000	C	-2.743033	0.048495	-0.731525	C	-2.729336	-0.004041	-0.834132
H	2.79853400	0.50836700	1.81153500	H	-2.779679	0.580864	-1.688974	H	-2.764472	0.450645	-1.830722
H	2.68585100	0.82083700	0.09155300	H	-2.675771	0.818526	0.042997	H	-2.651127	0.823626	-0.122884
C	4.07256800	-0.73514000	0.60081200	C	-4.043101	-0.727889	-0.537386	C	-4.034576	-0.752518	-0.575909
H	4.00773100	-1.29006500	-0.34168300	H	-3.989436	-1.292574	0.399863	H	-3.967660	-1.273828	0.385445
H	4.20987000	-1.47561200	1.38900300	H	-4.154577	-1.461357	-1.336421	H	-4.170769	-1.520549	-1.337471
C	5.28288800	0.19411200	0.55584600	C	-5.266081	0.185550	-0.506657	C	-5.247053	0.174912	-0.562538
H	5.08586400	0.99411300	-0.16409400	H	-5.087645	0.986572	0.216994	H	-5.051334	1.000176	0.128669
H	5.40081000	0.68118500	1.53052800	H	-5.376255	0.673625	-1.481876	H	-5.366919	0.627157	-1.553627
C	6.60470100	-0.48335200	0.17953900	C	-6.586462	-0.504854	-0.149747	C	-6.566954	-0.491993	-0.161473
H	6.46831600	-0.96084300	-0.79813800	H	-6.462377	-0.974847	0.833165	H	-6.428644	-0.934513	0.832256
C	7.70446300	0.57322000	0.03358200	C	-7.700097	0.540619	-0.031673	C	-7.669091	0.566486	-0.051746
H	7.35283700	1.34779400	-0.65460800	H	-7.369449	1.323839	0.657095	H	-7.318737	1.365584	0.608486
H	7.84994400	1.06662300	1.00128100	H	-7.831590	1.025887	-1.005593	H	-7.816526	1.025238	-1.036054
C	9.04660200	0.04503000	-0.46715100	C	-9.047374	0.004138	0.445729	C	-9.009569	0.053088	0.468441
H	8.89069400	-0.52220400	-1.39143600	H	-8.907160	-0.547539	1.381828	H	-8.851568	-0.480951	1.411947
H	9.46035200	-0.65358200	0.26102600	H	-9.436316	-0.709514	-0.281588	H	-9.422260	-0.671645	-0.234366
C	10.05182100	1.16353200	-0.72640000	C	-10.070284	1.115717	0.663266	C	-10.017218	1.177660	0.689129
H	9.59837100	1.89902900	-1.39777700	H	-9.640079	1.866817	1.332582	H	-9.564962	1.937352	1.333840
H	10.25677700	1.69082200	0.21243800	H	-10.257641	1.625354	-0.289002	H	-10.224185	1.671153	-0.267473
C	11.38109100	0.70480900	-1.33128800	C	-11.409646	0.654745	1.243599	C	-11.344907	0.737375	1.310954
H	11.16063000	0.19326400	-2.27424700	H	-11.208692	0.167727	2.203724	H	-11.122470	0.259798	2.271108
C	12.26552900	1.91143700	-1.63802600	C	-12.316290	1.856991	1.498292	C	-12.231945	1.951969	1.575897
H	11.76149500	2.61421900	-2.30229000	H	-11.840059	2.579981	2.161405	H	-11.729033	2.678835	2.214603
H	12.51522600	2.44207700	-0.71642400	H	-12.543729	2.364631	0.558030	H	-12.483646	2.449312	0.636442
C	6.99258800	-1.56346700	1.18878900	C	-6.943108	-1.595765	-1.158833	C	-6.953108	-1.607809	-1.131797
H	7.14261200	-1.11897800	2.17642300	H	-7.080463	-1.158654	-2.151681	H	-7.104986	-1.198652	-2.134305
H	6.22179600	-2.32742600	1.27808200	H	-6.158811	-2.347645	-1.229259	H	-6.180585	-2.372633	-1.194882
H	7.91494400	-2.06668100	0.90337100	H	-7.863357	-2.111263	-0.887693	H	-7.874039	-2.102833	-0.828046
C	12.11996000	-0.27270600	-0.41872300	C	-12.112562	-0.352795	0.335275	C	-12.082206	-0.273326	0.433965
H	12.28111100	0.17771200	0.56408600	H	-12.250878	0.071825	-0.662415	H	-12.245222	0.141900	-0.563920

H	11.56755700	-1.20060100	-0.27730400	H	-11.545122	-1.276600	0.232142	H	-11.527726	-1.204326	0.324743
H	13.09608200	-0.52698700	-0.83294800	H	-13.097011	-0.608840	0.728615	H	-13.057387	-0.515171	0.857744
C	-9.17318600	2.27610300	0.44376600	C	9.241637	2.289522	-0.360226	C	9.204146	2.293290	-0.536652
H	-9.95861000	1.77234900	1.01492700	H	9.967134	1.813618	-1.025494	H	9.990293	1.771610	-1.090463
H	-8.78674200	3.07424100	1.06851800	H	8.860298	3.191245	-0.836798	H	9.673022	2.794368	0.314728
H	-9.64246700	2.74584500	-0.42507800	H	9.810611	2.567323	0.530362	C	6.382210	2.575719	-1.439770
C	-6.35272800	2.59676200	1.33869900	C	6.364261	2.782583	-0.999859	H	5.328764	2.490240	-1.681836
H	-5.29930300	2.52233300	1.58447700	H	5.290865	2.766508	-1.165587	H	6.548719	3.557432	-0.993492
H	-6.52112800	3.56179100	0.85806300	H	6.606688	3.679932	-0.425962	H	6.951021	2.537466	-2.369559
H	-6.92228200	2.58986000	2.26879500	H	6.859604	2.879586	-1.968297	C	7.947339	-1.726124	2.167967
C	-7.90457600	-1.83288300	-2.11691300	C	8.045110	-2.077865	1.739743	H	7.565525	-1.557998	3.177332
H	-7.52225100	-1.69942700	-3.13125100	H	7.731178	-2.108732	2.789814	H	7.548578	-2.682310	1.827603
H	-7.50389000	-2.77557300	-1.74283300	H	7.642664	-2.976549	1.262092	H	9.028311	-1.803613	2.226672
H	-8.98531200	-1.91494200	-2.17373900	H	9.132476	-2.114957	1.712786	O	9.774409	0.141090	1.200583
O	-9.73686600	0.06284600	-1.21723000	O	9.853843	-0.047112	0.934908	H	10.330245	0.801485	0.783999
H	-10.29461600	0.73615600	-0.82456300	H	5.398252	-1.950231	2.184667	H	5.294917	-1.577885	2.429750
H	-5.25227400	-1.68766500	-2.38158300	H	3.080750	-1.900245	1.045209	H	3.023370	-1.711854	1.225107
H	-2.98150200	-1.77385100	-1.17112700	C	3.984687	-1.083432	-1.500010	C	3.999612	-1.397418	-1.391795
C	-3.96082200	-1.36996300	1.43231300	H	3.442417	-2.030221	-1.505353	H	3.419969	-2.309215	-1.248426
H	-3.37892400	-2.28486200	1.32154600	H	3.723578	-0.520666	-2.397614	H	3.793688	-1.002313	-2.386528
H	-3.75671300	-0.93966300	2.41271500	H	5.052633	-1.293595	-1.527492	H	5.057139	-1.653902	-1.340537
H	-5.01770100	-1.63058900	1.38923900	H	-1.469097	-1.359304	0.254381	H	-1.446345	-1.301587	0.279279
H	1.48639300	-1.32003700	-0.23666100	C	-1.418528	-1.802076	-1.839561	C	-1.463415	-1.998178	-1.745425
C	1.50327300	-1.94501000	1.81128500	H	-2.287470	-2.459260	-1.841906	H	-2.318068	-2.659324	-1.611526
H	2.35958700	-2.60843000	1.70139800	H	-1.390716	-1.273504	-2.796392	H	-1.506724	-1.593353	-2.760113
H	1.54472700	-1.50467900	2.81115500	H	-0.532621	-2.432188	-1.780924	H	-0.566299	-2.610382	-1.666436
H	0.60766000	-2.56173000	1.75312700	H	-13.261063	1.551940	1.949687	H	-13.165104	1.662199	2.059844
H	13.19979500	1.60704700	-2.11073200	C	-0.236148	0.113179	-0.684215	C	-0.220813	0.023558	-0.864987
C	0.25674600	0.04159700	0.85933400	H	-0.239952	0.717302	-1.598764	H	-0.205838	0.461020	-1.869419
H	0.23984900	0.51404700	1.84776000	H	-0.329556	0.819305	0.146426	H	-0.308614	0.861394	-0.167116
H	0.34321700	0.85459900	0.13252300								
**α-Tocopherol (C2$^-$)**				**α-Tocopherol (C3$^-$)**				**α-Tocopherol (C4$^-$)**			
C	3.632033	-0.361769	-0.333888	C	3.640787	-0.396724	-0.308317	C	3.631747	-0.384728	-0.310663
C	5.839012	0.550080	-0.031704	C	5.849805	0.511437	-0.009954	C	5.839847	0.522283	-0.002180
C	6.190895	-0.472774	0.846534	C	6.196976	-0.504456	0.878183	C	6.188170	-0.503295	0.874299
C	5.142524	-1.425305	1.364867	C	5.144819	-1.449234	1.402990	C	5.137053	-1.455087	1.388409
C	3.734858	-0.914727	1.084904	C	3.739199	-0.937142	1.115611	C	3.730870	-0.941223	1.107010
C	6.788946	1.459747	-0.509847	C	6.803323	1.414003	-0.494383	C	6.792373	1.431301	-0.476387
C	7.523235	-0.616145	1.245023	C	7.528104	-0.648070	1.280618	C	7.519488	-0.650093	1.274950
H	5.283551	-2.409541	0.907108	H	5.283889	-2.437968	0.954416	H	5.277108	-2.438502	0.928593
H	3.488562	-0.104137	1.774559	H	3.493897	-0.119649	1.797428	H	3.484786	-0.131804	1.798114
C	8.462206	0.290755	0.774186	C	8.470636	0.251826	0.803498	C	8.461031	0.256161	0.808022
C	8.112902	1.329232	-0.092155	C	8.126066	1.283441	-0.072878	C	8.115295	1.297329	-0.056517
O	4.545798	0.738923	-0.456302	O	4.557988	0.700174	-0.438828	O	4.547790	0.714542	-0.428759
C	2.266853	0.269617	-0.599221	C	2.277976	0.236181	-0.582071	C	2.268248	0.249830	-0.577067
H	2.322959	0.778587	-1.565344	H	2.337471	0.736242	-1.552634	H	2.327120	0.760972	-1.541878
H	2.106674	1.045500	0.153654	H	2.118564	1.019291	0.163448	H	2.108096	1.024232	0.177336
C	1.087920	-0.699104	-0.602613	C	1.096241	-0.729122	-0.579083	C	1.087520	-0.716678	-0.584963
H	1.249670	-1.467625	-1.359879	H	1.257254	-1.504914	-1.329056	H	1.249265	-1.483711	-1.343737
H	1.020816	-1.214295	0.359854	H	1.025737	-1.235404	0.387858	H	1.017645	-1.234012	0.376157
C	-1.479403	-0.865203	-0.722241	C	-1.471312	-0.888878	-0.702350	C	-1.479877	-0.877700	-0.709805
C	-2.743820	-0.007749	-0.836525	C	-2.733003	-0.028842	-0.826900	C	-2.742478	-0.017620	-0.824438
H	-2.778282	0.441528	-1.835589	H	-2.764184	0.411492	-1.830045	H	-2.774222	0.434076	-1.822501
H	-2.664715	0.823697	-0.129797	H	-2.652880	0.808719	-0.127548	H	-2.663156	0.812007	-0.115601
C	-4.049988	-0.753245	-0.574397	C	-4.041848	-0.768165	-0.560673	C	-4.050523	-0.761293	-0.566527
H	-3.983821	-1.269375	0.389799	H	-3.979088	-1.275756	0.408272	H	-3.987134	-1.279818	0.396569
H	-4.186999	-1.525261	-1.331771	H	-4.179605	-1.546590	-1.311322	H	-4.187546	-1.531262	-1.325979
C	-5.261357	0.175693	-0.566259	C	-5.250526	0.164303	-0.563345	C	-5.260173	0.169883	-0.558470
H	-5.064743	1.004485	0.120457	H	-5.052862	0.998692	0.116256	H	-5.063309	0.996685	0.130571
H	-5.380553	0.622660	-1.559820	H	-5.366458	0.602622	-1.561137	H	-5.376665	0.619415	-1.551194
C	-6.582106	-0.487435	-0.161739	C	-6.573994	-0.491326	-0.155504	C	-6.582915	-0.491729	-0.157999
H	-6.444456	-0.924685	0.834411	H	-6.439585	-0.919959	0.844826	H	-6.447955	-0.931585	0.837372
C	-7.682992	0.572944	-0.057947	C	-7.671997	0.573122	-0.063492	C	-7.682017	0.570454	-0.053771
H	-7.331770	1.375217	0.597958	H	-7.319742	1.380267	0.585849	H	-7.330537	1.370521	0.604687
H	-7.829750	1.026488	-1.044767	H	-7.815484	1.018158	-1.054656	H	-7.826071	1.026598	-1.039790
C	-9.024151	0.063995	0.464852	C	-9.015661	0.072791	0.461191	C	-9.025104	0.062779	0.465304
H	-8.866912	-0.465069	1.411283	H	-8.861832	-0.448157	1.412671	H	-8.870633	-0.468809	1.410775
H	-9.437615	-0.664072	-0.234043	H	-9.429856	-0.660361	-0.231932	H	-9.438607	-0.662864	-0.236085
C	-10.030484	1.190959	0.679255	C	-10.019136	1.204547	0.663399	C	-10.029738	1.191111	0.680478
H	-9.577405	1.953620	1.319869	H	-9.565109	1.971651	1.298010	H	-9.576446	1.951412	1.323742
H	-10.236736	1.679466	-0.280057	H	-10.222069	1.684965	-0.300694	H	-10.233272	1.682264	-0.278063
C	-11.358780	0.755664	1.303294	C	-11.349922	0.778744	1.288678	C	-11.360015	0.756825	1.300990
H	-11.137039	0.283073	2.266072	H	-11.131460	0.314229	2.256124	H	-11.140968	0.281552	2.263064
C	-12.244400	1.972746	1.561475	C	-12.232510	2.000661	1.534091	C	-12.243852	1.974946	1.560382
H	-11.740704	2.702488	2.196271	H	-11.727951	2.734647	2.163283	H	-11.739993	2.702247	2.197846

H	-12.495384	2.465249	0.619284	H	-12.480198	2.485359	0.586992	H	-12.492143	2.470139	0.618887
C	-6.969467	-1.608072	-1.126007	C	-6.962703	-1.619502	-1.110394	C	-6.970545	-1.609364	-1.125639
H	-7.120725	-1.204216	-2.130756	H	-7.110800	-1.224301	-2.119047	H	-7.119159	-1.202857	-2.129713
H	-6.197850	-2.374151	-1.184807	H	-6.193202	-2.388307	-1.160731	H	-6.200248	-2.376742	-1.184799
H	-7.891029	-2.100330	-0.819683	H	-7.886296	-2.106310	-0.801487	H	-7.893598	-2.100622	-0.822211
C	-12.097172	-0.258931	0.431736	C	-12.089535	-0.241544	0.424835	C	-12.098653	-0.254331	0.425652
H	-12.259560	0.151032	-0.568424	H	-12.248753	0.159838	-0.579308	H	-12.258394	0.158294	-0.573840
H	-11.543791	-1.191175	0.327677	H	-11.538661	-1.176284	0.330302	H	-11.546814	-1.187358	0.320431
H	-13.072696	-0.497293	0.856696	H	-13.066586	-0.473239	0.849973	H	-13.075418	-0.491873	0.848214
C	9.192360	2.276917	-0.549955	C	9.209176	2.223831	-0.537049	C	9.197376	2.244075	-0.510049
H	9.977955	1.751282	-1.100801	H	9.994328	1.690976	-1.081553	H	9.983027	1.718274	-1.060658
H	8.804533	3.048594	-1.206296	H	8.824892	2.990667	-1.201103	H	8.812223	3.018021	-1.165292
H	9.661723	2.782078	0.298739	H	9.678327	2.735277	0.307991	H	9.666080	2.746354	0.340718
C	6.370882	2.557779	-1.454991	C	6.390321	2.504657	-1.450233	C	6.378135	2.532338	-1.419717
H	6.538506	3.541716	-1.014059	H	5.337042	2.419980	-1.693301	H	5.324919	2.449335	-1.663638
H	6.939768	2.513769	-2.384479	H	6.559932	3.492048	-1.017864	H	6.546761	3.514917	-0.976147
C	7.930395	-1.726159	2.176405	H	6.960914	2.450612	-2.378145	H	6.948688	2.489459	-2.348236
H	7.548650	-1.552067	3.184784	C	7.930194	-1.750799	2.222794	C	7.922825	-1.763059	2.204471
H	7.530536	-2.683713	1.841214	H	7.546964	-1.566506	3.228793	H	7.539508	-1.590634	3.212539
H	9.011267	-1.804617	2.235681	H	9.010714	-1.831841	2.284941	H	7.521816	-2.719071	1.866267
O	9.759822	0.133559	1.199088	O	9.766947	0.094725	1.232400	H	9.003436	-1.843675	2.265592
H	10.316502	0.791005	0.778980	H	10.326366	0.746740	0.807491	O	9.757550	0.095541	1.234989
H	5.278118	-1.573325	2.437009	H	5.277862	-1.587957	2.476694	H	10.316244	0.752935	0.817481
H	3.006571	-1.711158	1.232803	H	3.008309	-1.730098	1.269232	H	3.000823	-1.736640	1.251659
C	3.983531	-1.412187	-1.385643	C	3.991310	-1.457608	-1.349842	C	3.983268	-1.433317	-1.364234
H	3.402780	-2.322495	-1.237375	H	3.407623	-2.364858	-1.194530	H	3.400544	-2.342886	-1.219209
H	3.778210	-1.022276	-2.382547	H	3.789094	-1.076125	-2.350635	H	3.780551	-1.040673	-2.360598
H	5.040744	-1.669650	-1.332839	H	5.047663	-1.717637	-1.292600	H	5.039898	-1.692877	-1.310053
H	-1.462527	-1.300725	0.284134	H	-1.457692	-1.315342	0.307946	H	-1.465707	-1.315627	0.295567
C	-1.480164	-2.008345	-1.736738	C	-1.473390	-2.041128	-1.706489	C	-1.480857	-2.018442	-1.727000
H	-2.335625	-2.667728	-1.599348	H	-2.331033	-2.696769	-1.564867	H	-2.337802	-2.676549	-1.592775
H	-1.522856	-1.609017	-2.753628	H	-1.512910	-1.650878	-2.727024	H	-1.520891	-1.616638	-2.743022
H	-0.583791	-2.621179	-1.654282	H	-0.578967	-2.655779	-1.616710	H	-0.585784	-2.633142	-1.644309
H	-13.177968	1.686738	2.046870	H	-13.167863	1.721746	2.020175	H	-13.178863	1.689538	2.043345
C	-0.235262	0.016685	-0.867186	C	-0.224329	-0.011933	-0.852755	C	-0.233825	0.002202	-0.850329
H	-0.219632	0.448636	-1.873990	H	-0.205453	0.410862	-1.863383	H	-0.215495	0.436498	-1.856078
H	-0.322153	0.858425	-0.173913	H	-0.310144	0.836281	-0.167282	H	-0.320454	0.842466	-0.155234
**α-Tocopherol (C5^{-})**				**α-Tocopherol (C6^{-})**				**α-Tocopherol (C7^{-})**			
C	-3.271554	-0.983579	-0.380922	C	3.625035	-0.360219	-0.359666	C	-3.527182	-0.204562	0.210409
C	-7.113189	-0.694601	0.124754	C	5.833354	0.527992	-0.001971	C	-5.844968	0.534902	-0.116154
C	-6.542625	0.269350	-0.772546	C	6.183727	-0.547635	0.811428	C	-6.211965	-0.659432	-0.750875
C	-5.368579	-0.209957	-1.596556	C	5.133953	-1.528799	1.270073	C	-5.145537	-1.648305	-1.149003
C	-4.057712	-0.028460	-0.877100	C	3.727041	-0.999845	1.022282	C	-3.763397	-1.005309	-1.079691
C	-8.233159	-0.243946	0.904612	C	6.784630	1.464040	-0.423139	C	-6.826969	1.489860	0.212050
C	-7.026439	1.572018	-0.868077	C	7.515854	-0.717287	1.200191	C	-7.559881	-0.923544	-1.022288
H	-5.525175	-1.264153	-1.820437	H	5.273531	-2.483111	0.752425	H	-5.187128	-2.521732	-0.488312
H	-3.751400	1.004772	-0.724356	H	3.481938	-0.233006	1.760675	H	-3.638120	-0.305674	-1.908824
C	-8.114009	1.957811	-0.085069	C	8.456161	0.215563	0.786145	C	-8.515204	0.022758	-0.687095
C	-8.724553	1.051322	0.785328	C	8.108391	1.306045	-0.014415	C	-8.164255	1.231081	-0.083193
O	-6.655188	-1.880288	0.213874	O	4.540422	0.744585	-0.413990	O	-4.574409	0.856281	0.200708
C	-1.985037	-0.634863	0.324643	C	2.260788	0.288347	-0.585402	C	-2.197798	0.470964	0.276982
H	-2.018490	-1.025050	1.349316	H	2.317647	0.855896	-1.518273	H	-1.947639	0.853046	-0.722782
H	-1.883809	0.450699	0.399292	H	2.101750	1.016517	0.213940	C	-1.082804	-0.431088	0.755638
C	-0.746059	-1.211649	-0.366748	C	1.080429	-0.676583	-0.648476	H	-1.257186	-0.745986	1.789981
H	-0.872407	-2.290903	-0.471910	H	1.241049	-1.397135	-1.451747	H	-0.963194	-1.396129	0.189903
H	-0.684513	-0.803579	-1.380778	H	1.012563	-1.250098	0.280363	C	1.499875	-0.628512	0.891074
C	1.833999	-1.299657	-0.334759	C	-1.487135	-0.831205	-0.777902	C	2.785153	0.200869	0.806396
C	3.050366	-0.800754	0.451439	C	-2.750287	0.033527	-0.838933	H	2.833286	0.867068	1.675876
H	3.054270	-1.278991	1.437790	H	-2.784084	0.543662	-1.808358	H	2.721480	0.851831	-0.071197
H	2.929379	0.271605	0.632759	H	-2.669960	0.819649	-0.082241	C	4.077520	-0.608347	0.718265
C	4.398460	-1.035471	-0.226494	C	-4.057553	-0.724805	-0.623204	H	4.020588	-1.280725	-0.145291
H	4.360404	-0.644568	-1.249258	H	-3.992150	-1.299560	0.307291	H	4.173905	-1.243981	1.600076
H	4.582520	-2.107139	-0.310128	H	-4.195699	-1.448400	-1.426772	C	5.314822	0.276884	0.586754
C	5.551384	-0.371699	0.522230	C	-5.267552	0.203640	-0.557638	H	5.131046	1.023705	-0.191742
H	5.310309	0.685548	0.668098	H	-5.069719	0.988176	0.178906	H	5.458380	0.837651	1.517843
H	5.631329	-0.807172	1.524992	H	-5.386087	0.711254	-1.521707	C	6.613138	-0.462721	0.250018
C	6.915463	-0.469233	-0.168203	C	-6.589278	-0.481248	-0.194703	H	6.462071	-0.978609	-0.706009
H	6.804795	-0.068420	-1.182928	H	-6.452275	-0.979352	0.772548	C	7.754190	0.541875	0.062271
C	7.935881	0.405955	0.566271	C	-7.688601	0.572320	-0.025563	H	7.421428	1.317667	-0.633939
H	7.535517	1.421908	0.632802	H	-7.336199	1.332062	0.678579	H	7.939920	1.047496	1.016990
H	8.032886	0.047476	1.597466	H	-7.834687	1.086124	-0.982494	C	9.061977	-0.051538	-0.456092
C	9.318385	0.463367	-0.079349	C	-9.030510	0.034047	0.464944	H	8.863181	-0.617493	-1.372999
H	9.209478	0.685165	-1.146929	H	-8.874054	-0.552656	1.376901	H	9.455573	-0.764203	0.269899
H	9.794660	-0.515817	-0.012188	H	-9.445044	-0.648886	-0.277521	C	10.113496	1.016836	-0.742046
C	10.219758	1.514514	0.562512	C	-10.035182	1.147107	0.748585	H	9.673279	1.781805	-1.388878

H	9.694027	2.474046	0.566426	H	-9.580981	1.868107	1.435012	H	10.378158	1.523410	0.193558
H	10.389893	1.254940	1.613964	H	-10.240711	1.694197	-0.178728	C	11.395699	0.501171	-1.400436
C	11.575670	1.703056	-0.122649	C	-11.364120	0.676079	1.344683	H	11.115627	0.018458	-2.342795
H	11.386285	1.965238	-1.168988	H	-11.143078	0.144639	2.276437	C	12.332427	1.664175	-1.719224
C	12.341842	2.852666	0.527896	C	-12.247947	1.876206	1.677572	H	11.845314	2.402356	-2.357222
H	11.766249	3.778473	0.500185	H	-11.743177	2.564634	2.356155	H	12.637014	2.167094	-0.798260
H	12.552559	2.621374	1.574780	H	-12.498201	2.426293	0.767598	C	6.953340	-1.512057	1.307647
C	7.391765	-1.917151	-0.277590	C	-6.978287	-1.539661	-1.226267	H	7.089207	-1.034067	2.281784
H	7.537430	-2.342087	0.719266	H	-7.128946	-1.074337	-2.204160	H	6.157486	-2.248461	1.405415
H	6.668353	-2.537278	-0.804158	H	-6.207800	-2.301784	-1.332307	H	7.869036	-2.050432	1.064822
H	8.336212	-1.989528	-0.815599	H	-7.900574	-2.048531	-0.950830	C	12.112269	-0.530299	-0.530667
C	12.415491	0.427084	-0.095472	C	-12.104003	-0.281700	0.412229	H	12.346147	-0.101063	0.447126
H	12.568685	0.098031	0.935534	H	-12.265784	0.189452	-0.560705	H	11.506005	-1.420585	-0.369768
H	11.940158	-0.386826	-0.640965	H	-11.551996	-1.206557	0.250780	H	13.050227	-0.843366	-0.991145
H	13.396541	0.598195	-0.540258	H	-13.079879	-0.544402	0.821753	C	-9.256139	2.222627	0.237211
C	-9.915527	1.488595	1.604419	C	9.189244	2.278591	-0.412945	H	-9.971201	1.818756	0.961976
H	-10.394402	2.360273	1.162143	H	9.974067	1.786803	-0.995252	H	-8.853524	3.136227	0.662900
H	-10.653328	0.689153	1.673330	H	8.802557	3.089878	-1.020370	H	-9.816827	2.503754	-0.659661
H	-9.629333	1.742061	2.630776	H	9.659349	2.729714	0.465271	C	-6.398319	2.773284	0.879409
C	-8.853501	-1.229063	1.858702	C	6.368186	2.618928	-1.298671	H	-5.324429	2.762902	1.038671
H	-8.195488	-2.092420	1.932832	H	5.314546	2.550021	-1.545137	H	-6.646519	3.642662	0.265991
H	-9.002995	-0.800879	2.853838	H	6.537259	3.573526	-0.797863	H	-6.890067	2.902874	1.846020
H	-9.831192	-1.581721	1.511236	H	6.937010	2.631535	-2.229153	C	-7.968003	-2.217423	-1.676811
C	-6.395179	2.562580	-1.818951	C	7.921375	-1.883270	2.061249	H	-7.677848	-2.234639	-2.730349
H	-5.313295	2.610360	-1.683476	H	7.539885	-1.771188	3.078484	H	-7.477443	-3.065432	-1.197704
H	-6.573532	2.277613	-2.859152	H	7.520107	-2.817720	1.667629	H	-9.043814	-2.358053	-1.621006
H	-6.809984	3.555848	-1.669998	H	9.002130	-1.966826	2.115474	O	-9.840630	-0.262420	-0.980923
O	-8.617708	3.270392	-0.199985	O	9.753543	0.030532	1.200421	H	-10.387313	0.441238	-0.630254
H	-8.449823	3.721909	0.628897	H	10.311193	0.711835	0.821643	H	-5.349337	-2.024192	-2.156228
H	-5.321890	0.333149	-2.544287	H	5.269326	-1.742911	2.331023	H	-2.980820	-1.762752	-1.163724
C	-3.599555	-2.454448	-0.467292	H	2.997581	-1.802814	1.120805	C	-3.806970	-1.068675	1.441541
H	-3.213474	-2.898887	-1.389661	C	3.974989	-1.344234	-1.474281	H	-3.224868	-1.992660	1.398745
H	-3.135078	-2.989988	0.364821	H	3.392896	-2.261102	-1.382431	H	-3.520198	-0.508643	2.331003
H	-4.678694	-2.602404	-0.408945	H	5.031821	-1.606020	-1.437562	H	-4.863958	-1.331383	1.517283
H	1.837305	-0.795559	-1.308697	H	-1.470904	-1.328037	0.199671	H	1.515757	-1.353579	0.067737
C	1.899861	-2.806758	-0.579425	C	-1.489578	-1.909547	-1.861023	C	1.419252	-1.406848	2.203646
H	2.790547	-3.088186	-1.140554	H	-2.346009	-2.574894	-1.764517	H	2.287200	-2.049855	2.359159
H	1.916314	-3.341959	0.374032	H	-1.531678	-1.448154	-2.851322	H	1.353257	-0.711754	3.045533
H	1.035095	-3.153245	-1.142449	H	-0.594109	-2.527620	-1.816628	H	0.529886	-2.034844	2.221679
H	13.294537	3.028691	0.027147	H	-13.181936	1.562157	2.144471	H	13.235035	1.319245	-2.225218
C	0.543345	-0.903473	0.389405	C	-0.241696	0.056117	-0.868248	C	0.273771	0.272745	0.700567
H	0.518111	-1.402541	1.365189	H	-0.225427	0.549363	-1.846472	H	0.284593	1.066492	1.455299
H	0.577657	0.170613	0.598027	H	-0.327349	0.853589	-0.124335	H	0.362443	0.775436	-0.268060
**α-Tocopherol (C8$^-$)**				**α-Tocopherol (C9$^-$)**				**α-Tocopherol (C10$^-$)**			
C	3.613845	-0.369591	-0.336168	C	-3.561709	-0.441581	-0.312031	C	3.603446	-0.354320	-0.363584
C	5.821710	0.527752	0.000947	C	-5.805131	0.015144	0.467328	C	5.810915	0.528347	0.012603
C	6.171618	-0.527087	0.841324	C	-6.030122	0.935103	-0.559565	C	6.160860	-0.561554	0.806963
C	5.121650	-1.496665	1.323558	C	-4.884080	1.381795	-1.432379	C	5.111028	-1.551285	1.246676
C	3.714843	-0.974371	1.061454	C	-3.542847	0.995346	-0.822694	C	3.704149	-1.018618	1.006772
C	6.773157	1.453146	-0.442772	C	-6.847178	-0.382298	1.318562	C	6.762228	1.472249	-0.390567
C	7.503458	-0.686676	1.235292	C	-7.316041	1.445396	-0.765931	C	7.492614	-0.737544	1.194181
H	5.261823	-2.463619	0.830096	H	-4.987547	0.939061	-2.428606	H	5.251672	-2.496080	0.712136
H	3.469000	-0.189323	1.780204	H	-3.322137	1.640873	0.030132	H	3.457813	-0.265280	1.758531
C	8.443932	0.235699	0.798764	C	-8.341061	1.047621	0.079485	C	8.432952	0.203029	0.798063
C	8.096614	1.305711	-0.029148	C	-8.122218	0.147818	1.123919	C	8.085587	1.307553	0.016810
O	4.529074	0.733691	-0.417415	O	-4.581530	-0.523154	0.719150	O	4.518360	0.751706	-0.396979
C	2.249664	0.272839	-0.579186	C	-2.260409	-0.818928	0.388340	C	2.239149	0.297563	-0.579238
H	2.307177	0.816848	-1.525938	H	-2.434522	-1.736583	0.953989	H	2.296821	0.881802	-1.501697
H	2.089843	1.020775	0.201533	H	-2.030863	-0.040663	1.122156	H	2.078826	1.011183	0.232867
C	1.069532	-0.693608	-0.619019	C	-1.046747	-1.030383	-0.520201	C	1.059332	-0.666626	-0.661020
H	1.230937	-1.434027	-1.403854	H	-1.229120	-1.930876	-1.118470	H	1.221239	-1.372559	-1.476914
C	-1.497897	-0.851945	-0.746606	H	-1.012317	-0.199624	-1.274045	H	0.990661	-1.256763	0.257288
C	-2.761157	0.010730	-0.830305	C	1.453062	-1.215720	-0.611803	C	-1.508004	-0.820089	-0.796220
H	-2.794259	0.496410	-1.812234	C	2.720678	-0.942991	0.204869	C	-2.771503	0.045013	-0.843194
H	-2.681589	0.815580	-0.093482	H	2.772822	-1.696887	0.997746	H	-2.804416	0.572463	-1.803340
C	-4.068460	-0.742225	-0.596701	H	2.599616	0.022485	0.707697	H	-2.692440	0.817454	-0.072411
H	-4.003708	-1.293473	0.347955	C	4.035482	-0.930707	-0.574515	C	-4.078652	-0.717677	-0.642658
H	-4.205820	-1.485753	-1.381999	H	3.960950	-0.227352	-1.411828	H	-4.014055	-1.309023	0.277440
C	-5.278681	0.187326	-0.555397	H	4.205264	-1.916628	-1.011890	H	-4.215509	-1.426786	-1.459255
H	-5.081591	0.990110	0.161420	C	5.224329	-0.546036	0.303747	C	-5.289176	0.208879	-0.561849
H	-5.396524	0.670603	-1.531976	H	4.983997	0.386379	0.824454	H	-5.092584	0.980151	0.188900
C	-6.600577	-0.488522	-0.176484	H	5.353043	-1.303711	1.085945	H	-5.406832	0.733673	-1.516783
H	-6.464270	-0.962207	0.803053	C	6.559146	-0.362133	-0.425859	C	-6.610993	-0.483031	-0.212827
C	-7.700230	0.568730	-0.034684	H	6.416633	0.407126	-1.194682	H	-6.474878	-0.998364	0.745481
H	-7.348539	1.345947	0.650487	C	7.621711	0.146078	0.553868	C	-7.711023	0.566819	-0.026084

H	-7.845631	1.058367	-1.004306	H	7.228809	1.033047	1.060117	H	-7.359811	1.313954	0.692004
C	-9.042439	0.042643	0.468070	H	7.769176	-0.609548	1.334292	H	-7.856242	1.097660	-0.973803
H	-8.886617	-0.520995	1.394567	C	8.972754	0.495575	-0.065454	C	-9.053243	0.019200	0.453098
H	-9.456244	-0.658762	-0.257385	H	8.821526	1.194789	-0.895618	H	-8.897566	-0.583715	1.354553
C	-10.047545	1.162257	0.722923	H	9.424745	-0.400863	-0.492192	H	-9.466579	-0.650479	-0.302000
H	-9.594034	1.900320	1.391436	C	9.930834	1.115239	0.948918	C	-10.058785	1.126520	0.755505
H	-10.252418	1.685899	-0.217972	H	9.427835	1.951766	1.443535	H	-9.605735	1.835285	1.455304
C	-11.376881	0.706041	1.329560	H	10.146698	0.382030	1.734949	H	-10.263496	1.690083	-0.162073
H	-11.156500	0.198158	2.274515	C	11.255906	1.617033	0.370097	C	-11.388188	0.644245	1.341484
C	-12.261197	1.913952	1.631561	H	11.024394	2.368720	-0.392093	H	-11.167977	0.096257	2.263801
H	-11.757106	2.619268	2.293090	C	12.091221	2.283634	1.461131	C	-12.272985	1.837789	1.694836
H	-12.510812	2.441017	0.707887	H	11.546104	3.101204	1.934370	H	-11.769342	2.514151	2.386267
C	-6.988553	-1.572527	-1.181521	H	12.344902	1.557916	2.237629	H	-12.522444	2.404016	0.794594
H	-7.138502	-1.131881	-2.170887	C	7.003931	-1.649287	-1.119661	C	-6.998284	-1.522924	-1.263692
H	-6.217840	-2.336911	-1.267810	H	7.163552	-2.438257	-0.379253	H	-7.148028	-1.040181	-2.233246
H	-7.910971	-2.074524	-0.894166	H	6.248588	-1.998595	-1.821149	H	-6.227304	-2.282663	-1.382498
C	-12.115830	-0.274949	0.420798	H	7.932432	-1.513389	-1.674521	H	-7.920645	-2.037088	-0.998523
H	-12.276906	0.171646	-0.563767	C	12.053511	0.495863	-0.293351	C	-12.126518	-0.296974	0.391108
H	-11.563523	-1.203449	0.283017	H	12.237496	-0.309773	0.422253	H	-12.287392	0.191503	-0.573396
H	-13.091990	-0.527507	0.835985	H	11.526625	0.072052	-1.147001	H	-11.573875	-1.218526	0.213722
C	9.177614	2.268186	-0.451045	H	13.020390	0.860100	-0.643606	H	-13.102744	-0.567442	0.794700
H	9.962999	1.762121	-1.020213	C	-9.284013	-0.226542	2.010597	C	9.166434	2.287602	-0.362917
H	8.791273	3.063920	-1.078918	H	-10.077028	-0.727483	1.446197	H	9.952173	1.806716	-0.953041
H	9.646921	2.741266	0.415972	H	-8.982565	-0.902284	2.804216	H	8.780062	3.109488	-0.956126
C	6.357215	2.585652	-1.347301	H	-9.721939	0.655558	2.487170	H	9.635294	2.723095	0.523812
H	5.303788	2.510377	-1.592816	C	-6.565547	-1.368207	2.424644	C	6.346246	2.642484	-1.245702
H	6.525705	3.552531	-0.870431	H	-5.531289	-1.692406	2.384742	H	5.292928	2.577526	-1.494605
H	6.926793	2.575058	-2.277347	H	-6.743219	-0.923391	3.405782	H	6.514271	3.588011	-0.727632
C	7.908492	-1.830636	2.125621	H	-7.204943	-2.248856	2.341899	H	6.916149	2.672067	-2.175139
H	7.526154	-1.693180	3.139419	C	-7.581360	2.419927	-1.882551	C	7.897696	-1.918616	2.034634
H	7.507715	-2.774736	1.755212	H	-7.125136	3.390341	-1.673576	H	7.514964	-1.825001	3.053269
H	8.989218	-1.912589	2.182796	H	-7.155209	2.061523	-2.820274	H	7.497340	-2.846032	1.623822
O	9.741010	0.061369	1.218595	H	-8.647724	2.571324	-2.020243	H	8.978427	-2.002632	2.088620
H	10.298843	0.733081	0.823318	O	-9.596410	1.582040	-0.140092	O	9.729939	0.011189	1.210473
H	5.256198	-1.684101	2.389648	H	-10.214955	1.181771	0.472188	H	10.287700	0.699444	0.844649
H	2.985450	-1.774768	1.179475	H	-4.934054	2.462640	-1.578053	H	5.245266	-1.784356	2.303767
C	3.964885	-1.381152	-1.425495	H	-2.736373	1.112617	-1.543709	H	2.974964	-1.823566	1.090000
H	3.382885	-2.295549	-1.311173	C	-3.913495	-1.440629	-1.409425	C	3.955177	-1.317994	-1.495280
H	3.760849	-0.954660	-2.407575	H	-3.220191	-1.330185	-2.243136	H	3.373422	-2.236633	-1.420598
H	5.021734	-1.641721	-1.381374	H	-3.822344	-2.453935	-1.019146	H	3.751348	-0.849721	-2.458183
H	-1.482371	-1.324125	0.243121	H	-4.932384	-1.297588	-1.771365	H	5.012092	-1.579907	-1.462028
C	-1.499262	-1.957087	-1.802371	H	1.424288	-0.432339	-1.415086	H	-1.492672	-1.334393	0.172289
H	-2.355650	-2.619981	-1.689918	C	1.576850	-2.557106	-1.350719	C	-1.508660	-1.878804	-1.898536
H	-1.540641	-1.520658	-2.803953	H	2.356155	-2.543669	-2.120828	H	-2.364881	-2.546174	-1.815004
H	-0.603717	-2.573671	-1.741781	H	1.796750	-3.348964	-0.629337	H	-0.612944	-2.497159	-1.864198
H	-13.195509	1.611511	2.105426	H	0.636178	-2.810944	-1.840017	H	-13.207366	1.514971	2.154920
C	-0.252547	0.033087	-0.858148	H	13.023824	2.682940	1.059938	C	-0.262890	0.069290	-0.869150
H	-0.235572	0.501673	-1.848410	C	0.232419	-1.159778	0.273538	H	-0.245719	0.580035	-1.838338
H	-0.338951	0.848930	-0.134518	H	0.317306	-0.288029	0.938456	H	-0.349798	0.853231	-0.111135
**α-Tocopherol (C11$^-$)**				**α-Tocopherol (C12$^-$)**				**α-Tocopherol (C13$^-$)**			
C	3.603537	-0.368656	-0.337012	C	3.620520	-0.852234	-0.214750	C	-3.54141900	-0.42946900	-0.32120000
C	5.811173	0.529031	0.000690	C	5.627902	0.541122	-0.205838	C	-5.76774900	0.06400700	0.46513300
C	6.161423	-0.526382	0.840204	C	6.115850	-0.079687	0.936759	C	-6.00275700	0.93446500	-0.59917100
C	5.111753	-1.496636	1.321725	C	5.222085	-1.028951	1.709889	C	-4.86984500	1.34066300	-1.50807400
C	3.704786	-0.974524	1.060121	C	3.758738	-0.903519	1.301174	C	-3.51726100	0.97731200	-0.90941200
C	6.762336	1.455044	-0.442346	C	6.395567	1.467496	-0.924404	C	-6.79635300	-0.29928700	1.34404400
C	7.493331	-0.685914	1.233967	C	7.422893	0.203235	1.366476	C	-7.29157600	1.43124000	-0.81784000
H	5.252166	-2.463157	0.827483	H	5.576719	-2.059044	1.578054	H	-4.99173100	0.85919000	-2.48367200
H	3.458766	-0.190119	1.779512	H	3.346956	0.020684	1.708998	H	-3.26925900	1.66903000	-0.10140400
C	8.433522	0.237070	0.798121	C	8.175158	1.126404	0.662590	C	-8.30646600	1.06874000	0.05552300
C	8.085858	1.307646	-0.028916	C	7.680092	1.768882	-0.471266	C	-8.07447700	0.21878100	1.13833600
O	4.518454	0.734944	-0.417431	O	4.365037	0.312111	-0.677328	O	-4.53234400	-0.45910600	0.72788200
C	2.239163	0.273588	-0.579437	C	2.209815	-0.597556	-0.733865	C	-2.23371600	-0.77167300	0.38753600
H	2.296470	0.818369	-1.525757	H	2.312084	-0.438182	-1.810407	H	-2.38741600	-1.71270700	0.92309400
H	2.079180	1.020856	0.201888	H	1.909228	0.353721	-0.295584	H	-2.05569700	-0.00596700	1.14734800
C	1.059298	-0.693155	-0.619972	C	1.081436	-1.595794	-0.460315	C	-1.00917900	-0.89588700	-0.51588000
H	1.220863	-1.432902	-1.405408	H	1.301132	-2.581880	-0.917261	H	-1.17310200	-1.68621100	-1.25095800
H	0.990990	-1.244004	0.322454	H	0.983281	-1.773953	0.602723	H	-0.86146000	0.02974400	-1.07922800
C	-1.508095	-0.852104	-0.747536	C	-1.426130	-1.861648	-0.510261	C	1.53771500	-1.24272400	-0.58627300
C	-2.771600	0.010286	-0.830472	C	-2.775048	-1.206112	-0.915708	C	2.80680100	-1.23648800	0.27916200
H	-2.804894	0.496740	-1.812011	H	-2.684525	-0.143818	-0.686775	H	2.69800900	-2.09716900	0.99033400
H	-2.692212	0.814569	-0.093011	C	-4.093581	-1.579385	-0.243465	H	2.75856200	-0.34229800	0.91515100
C	-4.078679	-0.743218	-0.597393	H	-4.028647	-1.505499	0.888396	C	4.10682400	-1.21792000	-0.49453400
H	-4.013719	-1.295203	0.346819	H	-4.373931	-2.601903	-0.413544	H	4.27782000	-2.20953900	-0.93189300
H	-4.215878	-1.486158	-1.383276	C	-5.272888	-0.682902	-0.701043	C	5.27440300	-0.86114500	0.39955200

C	-5.289156	0.185962	-0.555275	H	-4.893816	0.343131	-0.713790	H	4.99773900	0.00476500	1.01618600
H	-5.092247	0.988229	0.162171	H	-5.523215	-0.951450	-1.728345	H	5.55731500	-1.65711300	1.13682000
H	-5.407191	0.669986	-1.531462	C	-6.606253	-0.634510	0.112336	C	6.56994500	-0.49203000	-0.33897700
C	-6.610842	-0.490555	-0.176826	H	-6.331073	-0.301042	1.123902	H	6.32002800	0.34522700	-0.99951500
H	-6.474346	-0.964985	0.802325	C	-7.590475	0.354189	-0.478450	C	7.65986000	-0.03172200	0.63435300
C	-7.710780	0.566277	-0.034117	H	-7.088100	1.314537	-0.651219	H	7.24267000	0.75685900	1.27034700
H	-7.359265	1.343044	0.651654	H	-7.905521	0.005024	-1.466142	H	7.92081500	-0.85877800	1.30485100
H	-7.856375	1.056648	-1.003339	C	-8.792522	0.548760	0.437224	C	8.93095900	0.49515100	-0.03019600
C	-9.052814	0.039416	0.468296	H	-8.431934	0.811279	1.448233	H	8.65717400	1.23866800	-0.78749100
H	-8.896781	-0.524919	1.394333	H	-9.316112	-0.404716	0.533636	H	9.42692100	-0.31877200	-0.56211400
H	-9.466467	-0.661525	-0.257696	C	-9.767607	1.650481	-0.045825	C	9.90424900	1.12782100	0.96154200
C	-10.058216	1.158546	0.724101	H	-9.193237	2.564519	-0.219657	H	9.36417400	1.87145000	1.55595200
H	-9.604871	1.896201	1.393177	H	-10.188626	1.354667	-1.017334	H	10.25215100	0.36605900	1.66995300
H	-10.263290	1.682883	-0.216363	C	-10.948697	2.008233	0.896902	C	11.12332200	1.80458500	0.32974000
C	-11.387389	0.701477	1.330452	H	-10.526053	2.315235	1.860479	H	10.75570100	2.56498200	-0.36779800
H	-11.166812	0.192900	2.274988	C	-11.727318	3.175936	0.313050	C	11.96364100	2.50274800	1.39728300
C	-12.272024	1.908900	1.633469	H	-11.072981	4.032073	0.141848	H	11.37041300	3.22799200	1.95567000
H	-11.768090	2.613828	2.295531	H	-12.165410	2.894886	-0.644460	H	12.35314400	1.77134800	2.10993000
H	-12.521839	2.436633	0.710231	C	-7.342240	-1.972363	0.239161	C	7.03584800	-1.65423500	-1.21138300
C	-6.998575	-1.573865	-1.182705	H	-7.617038	-2.336387	-0.755452	H	7.25779700	-2.52622800	-0.58617000
H	-7.148705	-1.132471	-2.171710	H	-6.721785	-2.729012	0.691948	H	6.23864000	-1.92086200	-1.90438000
H	-6.227656	-2.337967	-1.269649	H	-8.262361	-1.877598	0.842613	H	7.92963600	-1.41425800	-1.79187600
H	-7.920837	-2.076348	-0.895697	C	-11.894164	0.842351	1.149564	C	11.98502100	0.81737500	-0.45529800
C	-12.126119	-0.278994	0.420950	H	-12.291723	0.460221	0.201408	H	12.31559900	0.00549200	0.19811600
H	-12.287377	0.168343	-0.563248	H	-11.390877	0.023802	1.650235	H	11.43809000	0.37565000	-1.28670900
H	-11.573562	-1.207229	0.282396	H	-12.740131	1.162095	1.757311	H	12.87386400	1.30653800	-0.85761500
H	-13.102185	-0.532154	0.835992	C	8.569058	2.774043	-1.164628	C	-9.22620900	-0.11301300	2.05396900
C	9.166566	2.270758	-0.450108	H	9.485325	2.309080	-1.541112	H	-10.01938100	-0.65051400	1.52489000
H	9.952058	1.765367	-1.019726	H	8.074996	3.236986	-2.006389	H	-8.91553800	-0.73923300	2.88373200
H	8.779966	3.066886	-1.077323	H	8.864681	3.576052	-0.481098	H	-9.66562000	0.79298700	2.48081000
H	9.635793	2.743276	0.417259	C	5.819038	2.141102	-2.154963	C	-6.50344800	-1.23094000	2.49303100
C	6.346026	2.588155	-1.345945	H	4.828970	1.756272	-2.371421	H	-5.47100500	-1.56096000	2.46010000
H	5.292606	2.512783	-1.591458	H	5.730631	3.217300	-2.007153	H	-6.66992900	-0.73800800	3.45255100
H	6.514275	3.554701	-0.868313	H	6.446540	1.983658	-3.037325	H	-7.14582300	-2.11220100	2.46083900
H	6.915553	2.578462	-2.276033	C	7.996078	-0.465872	2.588373	C	-7.57024600	2.35288600	-1.97491400
C	7.898735	-1.830473	2.123358	H	7.512786	-0.102675	3.497058	H	-7.11746100	3.33376800	-1.81344000
H	7.516418	-1.693933	3.137289	H	7.838294	-1.546346	2.552423	H	-7.15155400	1.95483200	-2.89975400
H	7.498198	-2.774388	1.752220	H	9.061865	-0.275575	2.673342	H	-8.63816600	2.49369500	-2.11004100
H	8.979486	-1.912171	2.180405	O	9.433249	1.402254	1.137084	O	-9.56178300	1.58934500	-0.17603000
O	9.730674	0.062766	1.217736	H	9.869588	2.003661	0.534759	H	-10.18088000	1.20589100	0.44683300
H	10.288297	0.734948	0.822963	H	5.311027	-0.812175	2.770513	H	-4.91744000	2.41424300	-1.69657900
H	5.246416	-1.684886	2.387658	H	3.171137	-1.713766	1.696438	H	-2.73376200	1.05263600	-1.66129100
H	2.975622	-1.775218	1.177545	C	4.185831	-2.098176	-0.837892	C	-3.91031900	-1.48194100	-1.36383700
C	3.954794	-1.379250	-1.427167	H	3.727429	-2.985283	-0.387657	H	-3.25937500	-1.40349100	-2.23400800
H	3.373056	-2.293900	-1.313541	H	3.979817	-2.096552	-1.906282	H	-3.79847300	-2.47799300	-0.93597500
H	3.750582	-0.952031	-2.408894	H	5.269599	-2.148241	-0.700055	H	-4.94297900	-1.36274100	-1.69115600
H	5.011719	-1.639560	-1.383316	H	-1.346876	-1.938740	0.619757	H	1.58156600	-0.33816400	-1.20281500
C	-1.509214	-1.956403	-1.804182	C	-1.140890	-3.283998	-1.040401	C	1.53911900	-2.44924900	-1.52120800
H	-2.365412	-2.619625	-1.692209	H	-1.964613	-3.945722	-0.852620	H	2.43145600	-2.41397900	-2.14532600
H	-1.550774	-1.519186	-2.805414	H	-1.006012	-3.246121	-2.120321	H	1.56425300	-3.37405900	-0.93465200
H	-0.613494	-2.572787	-1.744137	H	-0.245226	-3.736937	-0.571373	H	0.66266800	-2.48179300	-2.17308400
H	-13.206223	1.605820	2.107146	H	-12.538996	3.487751	0.974137	H	12.81478000	3.02470000	0.95632800
C	-0.262997	0.033363	-0.858444	C	-0.251633	-1.043935	-0.960578	C	0.26861900	-1.18790400	0.27052800
H	-0.246211	0.502744	-1.848332	H	-0.246105	-0.951290	-2.047438	H	0.15182400	-2.13366500	0.81181800
H	-0.349585	0.848604	-0.134158	H	-0.397132	-0.031181	-0.575132	H	0.39441600	-0.41182600	1.03325500
**α-Tocopherol (C14$^-$)**				**α-Tocopherol (C15$^-$)**				**α-Tocopherol (C16$^-$)**			
C	3.551007	-0.467597	-0.198396	C	-3.587676	-0.311826	0.331726	C	3.582688	-0.367310	-0.334536
C	5.757572	0.506946	-0.141754	C	-5.804373	0.546486	-0.059913	C	5.790131	0.532681	-0.001747
C	6.099841	-0.229105	0.989013	C	-6.210983	-0.645691	-0.657554	C	6.141123	-0.518865	0.842296
C	5.046246	-1.002087	1.738825	C	-5.196465	-1.710091	-0.993414	C	5.092014	-1.487360	1.328559
C	3.644690	-0.586723	1.314793	C	-3.773236	-1.171927	-0.914085	C	3.684744	-0.966933	1.065230
C	6.711618	1.257188	-0.835935	C	-6.722754	1.561011	0.237055	C	6.740766	1.457069	-0.449288
C	7.425721	-0.247832	1.427279	C	-7.564805	-0.852868	-0.940172	C	7.473255	-0.676157	1.236205
H	5.186406	-2.070555	1.568158	H	-5.315314	-2.556270	-0.309102	H	5.232579	-2.456013	0.838553
H	3.400143	0.387064	1.736006	H	-3.560132	-0.545781	-1.783349	H	3.438735	-0.179433	1.781236
C	8.366143	0.499609	0.738404	C	-8.471902	0.153510	-0.641894	C	8.412922	0.245230	0.795872
C	8.026848	1.259034	-0.379402	C	-8.069151	1.359615	-0.065165	C	8.064515	1.311996	-0.035763
O	4.475413	0.561185	-0.621167	O	-4.494587	0.803934	0.246111	O	4.497162	0.736260	-0.420236
C	2.191038	0.053688	-0.644294	C	-2.207322	0.338054	0.373257	C	2.217975	0.273348	-0.579237
H	2.261740	0.275502	-1.709433	H	-2.207972	1.056856	1.197280	H	2.274685	0.813944	-1.527990
H	2.013558	1.001369	-0.136450	H	-2.082513	0.916367	-0.545828	H	2.058041	1.024018	0.198830
C	1.014021	-0.886752	-0.403004	C	-1.032394	-0.621856	0.541617	C	1.038451	-0.694000	-0.614986
H	1.189255	-1.829977	-0.914933	H	-1.170265	-1.216315	1.446541	H	1.199962	-1.437167	-1.397198
H	0.920489	-1.113294	0.659679	H	-0.995865	-1.325651	-0.295225	H	0.970741	-1.240685	0.329904
C	-1.551480	-1.086205	-0.545308	C	1.542294	-0.775288	0.603329	C	-1.528936	-0.854461	-0.740766

C	-2.804195	-0.322658	-0.984628	C	2.811949	0.082119	0.636741	C	-2.792795	0.007086	-0.827000
H	-2.793325	-0.202492	-2.069729	H	2.857407	0.603988	1.600353	H	-2.826679	0.489167	-1.810675
H	-2.761913	0.685095	-0.566237	H	2.723757	0.861509	-0.128203	H	-2.713397	0.814665	-0.093150
C	-4.136967	-0.946148	-0.576535	C	4.116871	-0.678986	0.418458	C	-4.099497	-0.745858	-0.590031
H	-4.045250	-1.251216	0.497638	H	4.055337	-1.245130	-0.519200	H	-4.033938	-1.293621	0.356594
H	-4.262225	-1.883698	-1.121580	H	4.274745	-1.401446	1.218723	H	-4.236749	-1.492329	-1.372551
C	-5.301942	-0.020927	-0.834639	C	5.333245	0.237225	0.372034	C	-5.310301	0.183054	-0.551531
H	-5.092964	0.942093	-0.354796	H	5.157645	1.025692	-0.374269	H	-5.113389	0.988570	0.162265
C	-6.601668	-0.560057	-0.292346	H	5.450222	0.717539	1.350404	H	-5.428923	0.662697	-1.529806
H	-6.495865	-0.919303	0.761320	C	6.644174	-0.490932	0.047905	C	-6.631578	-0.492264	-0.169528
C	-7.665527	0.539295	-0.270233	H	6.517654	-0.852153	-1.005277	C	-7.731848	0.564786	-0.031050
H	-7.239607	1.410208	0.232038	C	7.751787	0.567070	-0.031545	H	-7.380335	1.344719	0.651119
H	-7.853896	0.840677	-1.302445	H	7.465790	1.343097	-0.756571	H	-7.878029	1.050796	-1.002378
C	-8.982995	0.177216	0.412346	H	7.834747	1.041344	0.953438	C	-9.073476	0.039665	0.474259
H	-8.783282	-0.162345	1.431474	C	9.114661	0.008006	-0.420879	H	-8.916847	-0.520496	1.402726
H	-9.447843	-0.656790	-0.109854	H	9.019348	-0.561276	-1.354042	H	-9.487172	-0.664644	-0.248441
C	-9.950680	1.356437	0.457244	H	9.424270	-0.694886	0.352552	C	-10.079186	1.159549	0.725515
H	-9.428046	2.220926	0.870911	C	10.171946	1.095465	-0.594268	H	-9.625835	1.900334	1.391120
H	-10.235338	1.629721	-0.562369	H	9.792147	1.857952	-1.283199	H	-10.284847	1.679630	-0.217181
C	-11.222377	1.121858	1.272826	H	10.326408	1.601343	0.366695	C	-11.407936	0.704685	1.334446
H	-10.923222	0.862871	2.290399	C	11.527801	0.607132	-1.111590	H	-11.186776	0.200388	2.281138
C	-12.062355	2.395157	1.325310	H	11.362399	0.133959	-2.085884	C	-12.292892	1.913116	1.632471
H	-11.490468	3.224214	1.736519	C	12.483650	1.782537	-1.308443	H	-11.788943	2.621161	2.291187
H	-12.378332	2.672764	0.319919	H	12.063537	2.523989	-1.989948	H	-12.543289	2.436653	0.707005
C	-7.104766	-1.765023	-1.096191	H	12.671424	2.277179	-0.352015	C	-7.019329	-1.580171	-1.170426
H	-7.340936	-1.437041	-2.108899	C	6.958882	-1.565271	1.059897	H	-7.170036	-1.143227	-2.161317
H	-6.329678	-2.523971	-1.166066	H	6.176720	-2.327232	1.117755	H	-6.248163	-2.344366	-1.254300
H	-7.984954	-2.231829	-0.647915	H	7.902702	-2.075424	0.849744	H	-7.941285	-2.081715	-0.880804
C	-12.054610	-0.027945	0.711540	C	12.152518	-0.431018	-0.181147	C	-12.146682	-0.280085	0.429615
H	-12.306791	0.172342	-0.330118	H	12.259313	-0.017203	0.824981	H	-12.308517	0.162819	-0.556492
H	-11.514720	-0.968938	0.751041	H	11.535102	-1.324506	-0.107934	H	-11.593839	-1.208722	0.294951
H	-12.983691	-0.143647	1.267757	H	13.143385	-0.727010	-0.531950	H	-13.122481	-0.531760	0.846186
C	9.106545	2.059335	-1.060705	C	-9.116532	2.406423	0.219843	C	9.144690	2.273627	-0.461678
H	9.881074	1.412712	-1.476823	H	-9.855104	2.046394	0.942693	H	9.930132	1.766002	-1.029377
H	8.710235	2.650380	-1.875260	H	-8.683374	3.312015	0.631241	H	8.757534	3.066821	-1.092259
H	9.582740	2.749097	-0.362936	H	-9.652296	2.690198	-0.690383	H	9.614104	2.750164	0.403386
C	6.301862	2.047878	-2.051334	C	-6.244960	2.843317	0.870439	C	6.323659	2.586004	-1.357733
H	5.261545	1.865866	-2.282696	H	-5.173441	2.811683	1.032839	H	5.270165	2.509155	-1.602468
H	6.430233	3.115920	-1.885279	H	-6.466532	3.702453	0.235240	H	6.491750	3.554723	-0.884467
H	6.899826	1.772054	-2.917184	H	-6.730815	3.012347	1.832716	H	6.892801	2.572393	-2.288007
C	7.824655	-1.055474	2.631718	C	-8.028850	-2.140864	-1.565609	C	7.879454	-1.816607	2.130498
H	7.460744	-0.594017	3.548873	H	-7.725292	-2.199074	-2.613410	H	7.497510	-1.675709	3.143972
H	7.404530	-2.056688	2.582496	H	-7.593573	-3.001033	-1.056437	H	7.479113	-2.762308	1.763721
H	8.902291	-1.133055	2.698993	H	-9.110455	-2.223471	-1.524364	H	8.960260	-1.897652	2.187454
O	9.665542	0.477140	1.202299	O	-9.795746	-0.076700	-0.943290	O	9.710313	0.073268	1.215713
H	10.220816	0.969718	0.598561	H	-10.323318	0.661409	-0.635001	H	10.267522	0.743898	0.817726
H	5.170948	-0.845991	2.808018	H	-5.391296	-2.103088	-1.992379	H	5.227192	-1.670827	2.395261
H	2.911522	-1.301832	1.675407	H	-3.056058	-1.991424	-0.912588	H	2.955927	-1.767367	1.186513
C	3.918996	-1.766363	-0.904989	C	-3.887790	-1.085588	1.613833	C	3.933864	-1.382603	-1.420341
H	3.341479	-2.595366	-0.504777	H	-3.319448	-2.014529	1.646120	H	3.352512	-2.296954	-1.302413
H	3.709736	-1.675028	-1.967500	H	-3.620094	-0.482693	2.481296	H	3.729084	-0.959822	-2.403869
H	4.975625	-1.986510	-0.780226	H	-4.948058	-1.327577	1.679663	H	4.990903	-1.642327	-1.375779
H	-1.588969	-1.200519	0.541512	H	1.540088	-1.331540	-0.342170	H	-1.512631	-1.323025	0.250665
C	-1.507443	-2.479141	-1.169237	C	1.520230	-1.786362	1.749337	C	-1.530087	-1.963439	-1.792500
H	-2.412606	-3.027935	-0.930177	H	2.423561	-2.392787	1.753104	H	-2.385992	-2.626473	-1.677226
H	-1.442364	-2.395262	-2.255029	H	1.466238	-1.263716	2.708636	H	-1.572227	-1.530687	-2.795645
H	-0.657418	-3.061935	-0.819000	H	0.666443	-2.462036	1.683544	H	-0.634113	-2.579220	-1.730095
H	-12.955798	2.258170	1.932375	H	13.444927	1.454877	-1.709772	H	-13.226781	1.611797	2.107881
C	-0.299038	-0.278414	-0.890796	C	0.300306	0.120779	0.619983	C	-0.284213	0.030965	-0.856126
H	-0.253444	-0.147018	-1.974572	H	0.317044	0.731061	1.530142	H	-0.268014	0.495953	-1.848095
H	-0.404202	0.719759	-0.463279	H	0.364762	0.821056	-0.218424	H	-0.370800	0.849380	-0.135430
**α-Tocopherol ($C17^-$)**				**α-Tocopherol ($C18^-$)**				**α-Tocopherol ($C19^-$)**			
C	3.560147	-0.373316	-0.288546	C	3.572293	-0.365795	-0.334485	C	-3.555348	-0.195568	0.385890
C	5.772402	0.525609	0.029597	C	5.780070	0.533717	-0.002622	C	-5.801664	0.483944	-0.154732
C	6.141527	-0.542932	0.845040	C	6.131443	-0.518307	0.840667	C	-6.115717	-0.787558	-0.642138
C	5.101583	-1.524035	1.326490	C	5.082502	-1.486901	1.327095	C	-5.027505	-1.813874	-0.837785
C	3.688886	-1.001674	1.095928	C	3.675141	-0.966143	1.064912	C	-3.641901	-1.184911	-0.771118
C	6.714302	1.464467	-0.410013	C	6.730555	1.458199	-0.450288	C	-6.776876	1.463210	0.011547
C	7.480875	-0.703280	1.213646	C	7.463799	-0.675986	1.233661	C	-7.447513	-1.106428	-0.948422
H	5.236873	-2.480824	0.812013	H	5.222624	-2.455325	0.836509	H	-5.122500	-2.594417	-0.075720
H	3.451411	-0.230087	1.831532	H	3.429690	-0.178972	1.781471	H	-3.435665	-0.634383	-1.691702
C	8.410629	0.229892	0.777568	C	8.403319	0.245493	0.793209	C	-8.427194	-0.117762	-0.782369
C	8.045019	1.314548	-0.021816	C	8.054542	1.312730	-0.037666	C	-8.100335	1.166910	-0.311092
O	4.477886	0.733706	-0.365545	O	4.486868	0.737691	-0.420198	O	-4.522387	0.851581	0.161850
C	2.193025	0.272787	-0.496959	C	2.207517	0.275180	-0.578007	C	-2.215650	0.541079	0.394345

H	2.246301	0.857366	-1.419592	H	2.263708	0.816250	-1.526520	H	-2.287153	1.347130	1.129170
H	2.033084	0.986346	0.315266	H	2.048175	1.025476	0.200542	H	-2.082449	1.021628	-0.577255
C	1.012525	-0.691049	-0.579323	C	1.027835	-0.691985	-0.613509	C	-1.008000	-0.337844	0.713884
H	1.177812	-1.400462	-1.391734	H	1.188751	-1.434776	-1.396199	H	-1.159470	-0.817826	1.679298
H	0.934977	-1.277239	0.341056	H	0.960639	-1.239140	0.331146	H	-0.920049	-1.132979	-0.030360
C	-1.557831	-0.835800	-0.759608	C	-1.539653	-0.852021	-0.737762	C	1.540380	-0.432012	0.883329
C	-2.816413	0.033369	-0.856574	C	-2.803444	0.009747	-0.822767	C	2.810852	0.416696	0.774780
H	-2.811541	0.555030	-1.821235	H	-2.837877	0.492332	-1.806175	H	2.833280	1.132445	1.604941
H	-2.757779	0.813901	-0.091180	H	-2.723474	0.816942	-0.088556	H	2.754749	1.017708	-0.138302
C	-4.140633	-0.709042	-0.691607	C	-4.110104	-0.743133	-0.585362	C	4.111234	-0.379039	0.757383
H	-4.104269	-1.308594	0.225136	H	-4.044029	-1.291387	0.360944	H	4.037469	-1.165179	-0.002553
H	-4.272559	-1.412168	-1.516230	H	-4.247951	-1.489188	-1.368175	H	4.245268	-0.880541	1.718569
C	-5.337493	0.238474	-0.637632	C	-5.320753	0.185929	-0.545632	C	5.336470	0.481602	0.462163
H	-5.164974	0.981717	0.148474	H	-5.123281	0.991054	0.168449	H	5.166165	1.027541	-0.471777
H	-5.404583	0.797471	-1.577287	H	-5.439920	0.666085	-1.523589	H	5.439110	1.245081	1.242496
C	-6.702782	-0.412980	-0.380307	C	-6.641885	-0.489398	-0.163144	C	6.655418	-0.290076	0.345512
H	-6.580430	-1.032281	0.551390	H	-6.504256	-0.959944	0.817720	H	6.510179	-1.081903	-0.398654
C	-7.775941	0.635118	-0.239006	C	-7.741920	0.567736	-0.023441	C	7.775036	0.625283	-0.150114
H	-7.399335	1.442252	0.408684	H	-7.389870	1.347273	0.658904	H	7.474770	1.068163	-1.105972
C	-9.042859	0.086362	0.370690	H	-7.888640	1.054260	-0.994430	H	7.896139	1.467411	0.539753
H	-8.878742	-0.439617	1.353662	C	-9.083305	0.042546	0.482441	C	9.128414	-0.065409	-0.339629
H	-9.485558	-0.684695	-0.271906	H	-9.497553	-0.661337	-0.240357	H	8.930394	-1.054799	-0.839239
C	-10.102859	1.163412	0.609243	C	-10.088700	1.162443	0.734897	H	9.514749	-0.329664	0.649185
H	-9.616676	2.027467	1.076409	H	-9.634828	1.902827	1.400594	C	10.123728	0.788770	-1.088683
H	-10.470002	1.514005	-0.361941	H	-10.294878	1.683033	-0.207407	H	9.672529	1.101273	-2.040903
C	-11.292347	0.746885	1.478507	C	-11.417133	0.707457	1.344428	C	11.419781	0.069719	-1.373875
H	-10.909201	0.483358	2.470671	H	-11.195451	0.202648	2.290725	H	11.252951	-0.937545	-1.839453
C	-12.275792	1.905255	1.639861	C	-12.301731	1.915861	1.643621	C	12.286629	0.861077	-2.355833
H	-11.776753	2.789586	2.040095	H	-11.797270	2.623500	2.302381	H	11.742693	1.045409	-3.285030
H	-12.699230	2.177038	0.669177	H	-12.552634	2.439903	0.718578	H	12.533863	1.830968	-1.916768
C	-7.022045	-1.423603	-1.495117	C	-7.030417	-1.576742	-1.164352	C	7.040781	-0.946936	1.671269
H	-7.021101	-0.906602	-2.458719	H	-7.181682	-1.139273	-2.154927	H	7.233040	-0.181837	2.427803
H	-6.314863	-2.259640	-1.525951	H	-6.259410	-2.341003	-1.249098	H	6.259181	-1.610127	2.048239
H	-8.017199	-1.846245	-1.350454	H	-7.952261	-2.078304	-0.874407	H	7.950808	-1.530377	1.553591
C	-12.007610	-0.478945	0.913430	C	-12.156584	-0.276749	0.439559	C	12.245327	-0.202604	-0.108067
H	-12.331612	-0.283520	-0.112688	H	-12.318973	0.166678	-0.546220	H	12.486729	0.748551	0.374282
H	-11.344171	-1.342441	0.893006	H	-11.603956	-1.205396	0.304077	H	11.685381	-0.805904	0.606857
H	-12.889699	-0.733575	1.506264	H	-13.132157	-0.528499	0.856614	H	13.175043	-0.735423	-0.342815
C	9.113395	2.292967	-0.440211	C	9.134585	2.274426	-0.463769	C	-9.200348	2.191184	-0.164418
H	9.887461	1.808487	-1.043063	H	9.919600	1.766980	-1.032217	H	-9.959663	1.864909	0.552752
H	8.708069	3.105135	-1.034454	H	8.747146	3.067994	-1.093705	H	-8.821365	3.145658	0.185558
H	9.601420	2.742669	0.429166	H	9.604608	2.750457	0.401242	H	-9.701880	2.375063	-1.119173
C	6.275622	2.613368	-1.282623	C	6.313038	2.587655	-1.357899	C	-6.381346	2.819803	0.530334
H	5.218391	2.534483	-1.510278	H	5.259380	2.511078	-1.602012	H	-5.317440	2.850916	0.748300
H	6.444417	3.570836	-0.786896	H	6.481561	3.556109	-0.884246	H	-6.598982	3.599517	-0.201016
H	6.827794	2.628237	-2.223289	H	6.881595	2.574436	-2.288536	H	-6.924633	3.063427	1.443605
C	7.904577	-1.861359	2.076768	C	7.870397	-1.816947	2.127120	C	-7.812671	-2.477921	-1.456401
H	7.534568	-1.745764	3.097906	H	7.489108	-1.676510	3.140905	H	-7.474052	-2.618349	-2.485106
H	7.503167	-2.800301	1.693991	H	7.469693	-2.762405	1.760114	H	-7.342252	-3.254735	-0.853844
H	8.986577	-1.937853	2.117862	H	8.951227	-1.898173	2.183358	H	-8.886716	-2.625511	-1.434743
O	9.718839	0.053987	1.170849	O	9.700949	0.073136	1.212149	O	-9.731456	-0.443613	-1.103990
H	10.262491	0.737270	0.776173	H	10.258003	0.743889	0.814154	H	-10.303719	0.305608	-0.915539
H	5.253880	-1.730834	2.386807	H	5.218322	-1.670929	2.393618	H	-5.162291	-2.313729	-1.798308
H	2.962235	-1.804458	1.211170	H	2.946287	-1.766536	1.186244	H	-2.878713	-1.953727	-0.666769
C	3.895739	-1.361652	-1.403268	C	3.922646	-1.380585	-1.421026	C	-3.853368	-0.852230	1.732164
H	3.317221	-2.278734	-1.294285	H	3.341239	-2.294914	-1.303198	H	-3.237721	-1.740784	1.872402
H	3.667615	-0.917984	-2.372227	H	3.717309	-0.957275	-2.404211	H	-3.646389	-0.150901	2.540788
H	4.954282	-1.619017	-1.386295	H	4.979676	-1.640480	-1.377258	H	-4.899105	-1.145237	1.796000
H	-1.576413	-1.340890	0.214126	H	-1.522793	-1.321091	0.253420	H	1.547406	-1.140602	0.046934
C	-1.529072	-1.907206	-1.849219	C	-1.541619	-1.960465	-1.790059	C	1.504566	-1.227262	2.188902
H	-2.410743	-2.542787	-1.795404	H	-2.397544	-2.623437	-1.714585	H	2.383216	-1.865152	2.285212
H	-1.516176	-1.436933	-2.836547	H	-1.584326	-1.527197	-2.792957	H	1.487691	-0.549228	3.045945
H	-0.652331	-2.550153	-1.768197	H	-0.645693	-2.576403	-1.728528	H	0.626158	-1.866722	2.245816
H	-13.102394	1.645913	2.306072	H	-13.235365	1.614431	2.119463	H	13.216109	0.334691	-2.599952
C	-0.306556	0.046607	-0.803782	C	-0.294878	0.033288	-0.853451	C	0.299954	0.448955	0.741170
H	-0.270672	0.561480	-1.770588	H	-0.279235	0.498779	-1.845194	H	0.274842	1.177175	1.560219
H	-0.404956	0.828623	-0.045034	H	-0.380899	0.851349	-0.132286	H	0.384220	1.038771	-0.177605
**α-Tocopherol (C20$^-$)**				**α-Tocopherol (C21$^-$)**				**α-Tocopherol (C22$^-$)**			
C	3.560962	-0.337518	-0.357350	C	3.560318	-0.332220	-0.365487	C	-3.565589	-0.334518	0.304186
C	5.776045	0.531861	0.011563	C	5.774467	0.530634	0.023732	C	-5.776477	0.550924	-0.046857
C	6.117832	-0.553760	0.816503	C	6.123722	-0.582378	0.787020	C	-6.182851	-0.604535	-0.712115
C	5.059433	-1.530430	1.264276	C	5.070649	-1.578189	1.204398	C	-5.169375	-1.649584	-1.107376
C	3.657758	-0.986557	1.020179	C	3.666018	-1.030064	0.987340	C	-3.745363	-1.120011	-0.991385
C	6.735542	1.466231	-0.395552	C	6.728576	1.483065	-0.353080	C	-6.692175	1.549876	0.303534
C	7.447786	-0.735082	1.207851	C	7.456121	-0.772948	1.165321	C	-7.536099	-0.792740	-1.009644

H	5.190902	-2.480643	0.737102	H	5.201842	-2.508158	0.642240	H	-5.290921	-2.535339	-0.475748
H	3.419494	-0.222621	1.763680	H	3.429751	-0.293722	1.758793	H	-3.528728	-0.443777	-1.821283
C	8.395255	0.195417	0.806634	C	8.398309	0.175233	0.794049	C	-8.441511	0.198235	-0.658821
C	8.056886	1.295897	0.015912	C	8.052441	1.302703	0.045755	C	-8.037860	1.369398	-0.014403
O	4.488778	0.760186	-0.404071	O	4.484392	0.769326	-0.377344	O	-4.465228	0.786824	0.278074
C	2.201065	0.320323	-0.580352	C	2.197752	0.329633	-0.557600	C	-2.183689	0.308232	0.394346
H	2.260706	0.892432	-1.510298	H	2.249772	0.931923	-1.468737	H	-2.189491	0.975614	1.260471
H	2.044461	1.045223	0.222398	H	2.045711	1.027753	0.269378	H	-2.049949	0.941186	-0.486636
C	1.016308	-0.639344	-0.649013	C	1.013633	-0.628980	-0.648731	C	-1.013574	-0.664422	0.514430
H	1.173972	-1.356113	-1.456372	H	1.168809	-1.323431	-1.475817	H	-1.165663	-1.314907	1.377317
H	0.947056	-1.218272	0.276409	H	0.947731	-1.232802	0.260921	H	-0.969427	-1.313017	-0.365385
C	-1.549907	-0.794370	-0.777911	C	-1.554432	-0.777020	-0.766687	C	1.558770	-0.828857	0.613552
C	-2.818184	0.063225	-0.836880	C	-2.820236	0.084831	-0.813845	C	2.825432	0.029521	0.694119
H	-2.859928	0.568457	-1.808852	H	-2.861932	0.600509	-1.780256	H	2.852857	0.526590	1.670817
H	-2.737526	0.854300	-0.085212	H	-2.736566	0.867562	-0.053863	H	2.752121	0.827517	-0.051198
C	-4.120667	-0.699524	-0.607798	C	-4.124759	-0.676482	-0.591861	C	4.135330	-0.723464	0.475756
H	-4.044729	-1.268978	0.325541	H	-4.052538	-1.250751	0.338730	H	4.083602	-1.270408	-0.472360
H	-4.262274	-1.429359	-1.405464	H	-4.265770	-1.401944	-1.393623	H	4.265236	-1.471086	1.258692
C	-5.339240	0.217996	-0.536935	C	-5.340719	0.244632	-0.521021	C	5.347350	0.205203	0.455938
H	-5.143730	1.009885	0.192592	H	-5.144316	1.034670	0.210056	H	5.164106	1.008313	-0.264726
H	-5.471371	0.717790	-1.503674	H	-5.469294	0.746711	-1.486974	H	5.445988	0.688670	1.434886
C	-6.651838	-0.475718	-0.157592	C	-6.656223	-0.445656	-0.146032	C	6.678828	-0.466983	0.105238
H	-6.503950	-0.959782	0.815543	H	-6.515964	-0.921506	0.832330	H	6.567645	-0.932821	-0.881852
C	-7.771546	0.559229	0.002243	C	-7.775489	0.591793	-0.004073	C	7.788128	0.585674	0.002512
H	-7.417903	1.347045	0.676299	H	-7.431787	1.384365	0.668397	H	7.449852	1.379767	-0.672479
H	-7.944729	1.042853	-0.966188	H	-7.936192	1.069906	-0.977576	H	7.917862	1.055123	0.985205
C	-9.092026	0.015578	0.545089	C	-9.106917	0.055313	0.518719	C	9.139351	0.065171	-0.480120
H	-8.913919	-0.520710	1.483000	H	-8.931888	-0.488803	1.454205	H	9.005170	-0.475838	-1.425658
H	-9.509211	-0.708726	-0.157500	H	-9.513700	-0.670657	-0.188725	H	9.558125	-0.638920	0.239660
C	-10.134450	1.105753	0.801658	C	-10.137106	1.160200	0.749986	C	10.167937	1.171452	-0.675831
H	-9.674466	1.883968	1.423299	H	-9.705865	1.919348	1.412094	H	9.768649	1.917011	-1.380226
H	-10.338141	1.600501	-0.194085	H	-10.356628	1.667890	-0.195277	H	10.338668	1.667666	0.286815
C	-11.425312	0.652409	1.422200	C	-11.474705	0.712479	1.355888	C	11.522567	0.649782	-1.176165
C	-12.287539	1.860795	1.670205	H	-11.201907	0.148257	2.279631	H	11.318509	0.276585	-2.209297
H	-11.805514	2.590873	2.327832	C	-12.377684	1.906205	1.578794	C	12.464927	1.843082	-1.345718
H	-12.523426	2.404949	0.710785	H	-11.883138	2.670587	2.193295	H	12.047516	2.606077	-2.012826
C	-7.028322	-1.555275	-1.172284	C	-7.026841	-1.533644	-1.153520	H	12.655942	2.289133	-0.365946
H	-7.170394	-1.106475	-2.159663	H	-7.162720	-1.093555	-2.145574	C	7.040129	-1.560831	1.109647
H	-6.261527	-2.326335	-1.257250	H	-6.258770	-2.304000	-1.227044	H	7.143446	-1.130432	2.109610
H	-7.959572	-2.043689	-0.893035	H	-7.958974	-2.021831	-0.875919	H	6.282945	-2.344669	1.152811
C	-12.155535	-0.274614	0.487043	C	-12.159668	-0.310276	0.438492	H	7.988804	-2.027812	0.853535
H	-12.322264	0.209054	-0.519585	H	-12.290510	0.125874	-0.555508	C	12.122429	-0.370269	-0.235097
H	-11.627906	-1.212349	0.294805	H	-11.605953	-1.252632	0.353871	H	11.567931	-1.314901	-0.226166
H	-13.145731	-0.538363	0.869760	H	-13.154601	-0.537484	0.826920	H	13.163789	-0.586508	-0.501683
C	9.144717	2.265915	-0.370428	C	9.135203	2.289937	-0.309685	C	-9.083034	2.402105	0.324191
H	9.931550	1.773924	-0.950117	H	9.920655	1.822336	-0.910961	H	-9.825808	2.005500	1.022973
H	8.764905	3.081411	-0.976665	H	8.749862	3.125978	-0.883547	H	-8.648434	3.281546	0.787346
H	9.610848	2.711329	0.513004	H	9.604250	2.703930	0.587351	H	-9.613540	2.738172	-0.571163
C	6.328782	2.632640	-1.260417	C	6.313394	2.679033	-1.172427	C	-6.213654	2.794057	1.008312
H	5.273797	2.576578	-1.504285	H	5.256802	2.629555	-1.410608	H	-5.142726	2.752047	1.172153
H	6.509014	3.581389	-0.752462	H	6.495517	3.609221	-0.631968	H	-6.431848	3.687204	0.420864
H	6.895042	2.646573	-2.192601	H	6.872806	2.727715	-2.107589	H	-6.702267	2.910874	1.976864
C	7.842686	-1.911691	2.059572	C	7.859346	-1.978370	1.971647	C	-8.001851	-2.042615	-1.706749
H	7.462319	-1.803861	3.077691	H	7.486575	-1.907639	2.995847	H	-7.699298	-2.042217	-2.756498
H	7.431786	-2.838907	1.659098	H	7.447152	-2.891545	1.541413	H	-7.568479	-2.930922	-1.246824
H	8.922705	-2.006173	2.112683	H	8.939872	-2.072435	2.013304	H	-9.083524	-2.125585	-1.669807
O	9.691994	-0.001126	1.223100	O	9.697640	-0.031617	1.196944	O	-9.763689	-0.011531	-0.977170
H	10.251159	0.684605	0.855134	H	10.252775	0.669280	0.852099	H	-10.291471	0.711335	-0.634912
H	5.192134	-1.755903	2.323287	H	5.209714	-1.841658	2.253766	H	-5.361802	-1.981081	-2.128743
H	2.920613	-1.782754	1.113253	H	2.931257	-1.831094	1.055671	H	-3.030084	-1.940319	-1.035949
C	3.906106	-1.316416	-1.478027	C	3.902268	-1.269059	-1.522583	C	-3.875411	-1.181553	1.537361
H	3.314951	-2.228031	-1.393693	H	3.316438	-2.186332	-1.466887	H	-3.313393	-2.114929	1.518355
H	3.705460	-0.856990	-2.445755	H	3.692828	-0.776454	-2.471946	H	-3.610624	-0.631805	2.440316
H	4.960765	-1.587771	-1.441840	H	4.958448	-1.535933	-1.503039	H	-4.937083	-1.421221	1.583140
H	-1.533306	-1.295247	0.197678	H	-1.539253	-1.291950	0.201550	H	1.565478	-1.344477	-0.354337
C	-1.546749	-1.869328	-1.864759	C	-1.554206	-1.836177	-1.868781	C	1.523649	-1.886578	1.716448
H	-2.411398	-2.524907	-1.779242	H	-2.423592	-2.487224	-1.796436	H	2.413228	-2.513325	1.695853
H	-1.576447	-1.402959	-2.853407	H	-1.575988	-1.355682	-2.850812	H	1.474609	-1.405056	2.697088
H	-0.656937	-2.496217	-1.814535	H	-0.668968	-2.469647	-1.823401	H	0.659875	-2.544023	1.620879
H	-13.251265	1.600963	2.118294	H	-13.307504	1.609393	2.080871	H	13.424575	1.516919	-1.753336
C	-0.305743	0.095212	-0.863386	C	-0.308674	0.111522	-0.839286	C	0.318556	0.068918	0.656591
H	-0.290311	0.594573	-1.838590	H	-0.297661	0.632243	-1.803325	H	0.320655	0.629232	1.598241
H	-0.392573	0.887858	-0.114490	H	-0.390197	0.887687	-0.072781	H	0.399483	0.812937	-0.141377

Structures of α-Tocopherol and their corresponding species in the SPLET (ETE_step) mechanism at M06-2X/6-311++G(2d,2p) level of theory in gas phase.

**M06-2X/6-311++G(2d,2p)**											
**α-Tocopherol (O•)**				**α-Tocopherol (C1•)**				**α-Tocopherol (C2•)**			
C	3.650359	-0.258340	-0.254146	C	3.652965	-0.290495	-0.281588	C	3.641799	-0.282076	-0.280935
C	5.868169	0.581481	0.112153	C	5.870613	0.573082	0.060545	C	5.853305	0.574385	0.066680
C	6.258356	-0.579759	0.775291	C	6.216851	-0.550032	0.810757	C	6.235478	-0.537389	0.774136
C	5.223762	-1.612137	1.161231	C	5.189111	-1.562698	1.237382	C	5.205976	-1.555717	1.204692
C	3.801118	-1.079889	1.027270	C	3.777892	-1.014742	1.059455	C	3.784845	-1.030076	1.045360
C	6.816796	1.532381	-0.278887	C	6.793159	1.524849	-0.357334	C	6.778258	1.582688	-0.384807
C	7.605170	-0.815752	1.049601	C	7.589918	-0.737270	1.107179	C	7.598980	-0.771664	1.085968
H	5.343181	-2.502473	0.532509	H	5.281888	-2.496472	0.662504	H	5.327013	-2.475071	0.619367
H	3.572237	-0.413763	1.863277	H	3.562466	-0.283048	1.842603	H	3.561594	-0.317296	1.843405
C	8.618479	0.130544	0.687035	C	8.510236	0.202387	0.724021	C	8.509570	0.197998	0.681467
C	8.159583	1.314615	0.009730	C	8.170447	1.397665	0.003677	C	8.147674	1.350409	-0.008154
O	4.519725	0.860414	-0.171557	O	4.540408	0.819014	-0.288277	O	4.524927	0.825208	-0.272941
C	2.258575	0.367900	-0.361329	C	2.272607	0.348059	-0.450387	C	2.257301	0.352460	-0.425108
H	2.286086	1.076058	-1.194795	H	2.316983	0.988055	-1.336437	H	2.294719	1.010240	-1.298032
H	2.088507	0.957453	0.543672	H	2.105662	1.009929	0.403772	H	2.094237	0.996957	0.442876
C	1.104213	-0.609019	-0.565147	C	1.105469	-0.625529	-0.585919	C	1.093899	-0.624095	-0.571461
H	1.285267	-1.200796	-1.464200	H	1.279452	-1.281479	-1.440621	H	1.267228	-1.265709	-1.437245
H	1.054630	-1.313402	0.270642	H	1.047976	-1.268813	0.297194	H	1.041754	-1.281819	0.301698
C	-1.467193	-0.798331	-0.688913	C	-1.464769	-0.801557	-0.716239	C	-1.477805	-0.804134	-0.695416
C	-2.743036	0.048517	-0.732359	C	-2.736659	0.050206	-0.777377	C	-2.750156	0.047140	-0.754164
H	-2.780025	0.580617	-1.689945	H	-2.771382	0.564435	-1.744735	H	-2.783597	0.565257	-1.719472
H	-2.675528	0.818757	0.041930	H	-2.666241	0.834217	-0.017385	H	-2.680677	0.828286	0.008865
C	-4.043026	-0.727841	-0.537502	C	-4.038733	-0.719308	-0.569202	C	-4.052848	-0.722598	-0.550802
H	-3.989021	-1.292147	0.399952	H	-3.979292	-1.280822	0.369712	H	-3.997042	-1.284200	0.388291
H	-4.154768	-1.461619	-1.336212	H	-4.158891	-1.455365	-1.364492	H	-4.170294	-1.458542	-1.346640
C	-5.266003	0.185593	-0.506688	C	-5.259258	0.196986	-0.531489	C	-5.273043	0.194368	-0.517768
H	-5.087413	0.986748	0.216777	H	-5.075730	0.996795	0.192208	H	-5.091473	0.994335	0.206246
H	-5.376383	0.673480	-1.481978	H	-5.373870	0.686280	-1.505584	H	-5.383530	0.683500	-1.492424
C	-6.586291	-0.504760	-0.149387	C	-6.578262	-0.492860	-0.168138	C	-6.594251	-0.493750	-0.159243
H	-6.462029	-0.974554	0.833599	H	-6.446923	-0.969146	0.810815	H	-6.467726	-0.968816	0.820948
C	-7.699943	0.540703	-0.031382	C	-7.689907	0.552741	-0.033734	C	-7.705247	0.553385	-0.031546
H	-7.369321	1.324009	0.657297	H	-7.352723	1.330921	0.657545	H	-7.370451	1.332132	0.660247
H	-7.831459	1.025851	-1.005359	H	-7.829700	1.044584	-1.003155	H	-7.839554	1.044100	-1.002318
C	-9.047196	0.004204	0.446049	C	-9.032722	0.012626	0.452304	C	-9.051350	0.016023	0.448334
H	-8.906979	-0.547472	1.382149	H	-8.882634	-0.549624	1.380596	H	-8.907282	-0.543679	1.379121
H	-9.436151	-0.709461	-0.281259	H	-9.430090	-0.692809	-0.278489	H	-9.445283	-0.691080	-0.282715
C	-10.070125	1.115769	0.663577	C	-10.052379	1.121966	0.694392	C	-10.071250	1.127213	0.680979
H	-9.639978	1.866854	1.332947	H	-9.614150	1.864931	1.367573	H	-9.636084	1.872045	1.354076
H	-10.257446	1.625432	-0.288684	H	-10.250782	1.643055	-0.249400	H	-10.263684	1.645242	-0.265725
C	-11.409504	0.654724	1.243782	C	-11.384804	0.654056	1.285160	C	-11.407575	0.662776	1.265599
H	-11.208614	0.167706	2.203918	H	-11.171800	0.151645	2.234715	H	-11.200796	0.164642	2.218775
C	-12.316269	1.856904	1.498391	C	-12.286169	1.853273	1.571068	C	-12.310032	1.863794	1.540284
H	-11.840185	2.579926	2.161577	H	-11.799614	2.565231	2.238591	H	-11.827365	2.578528	2.207671
H	-12.543648	2.364547	0.558118	H	-12.526354	2.375865	0.642277	H	-12.543763	2.382268	0.607535
C	-6.943080	-1.595878	-1.158179	C	-6.945316	-1.576985	-1.180926	C	-6.957924	-1.578903	-1.172138
H	-7.080879	-1.158947	-2.151042	H	-7.090168	-1.133526	-2.169902	H	-7.098716	-1.136496	-2.162171
H	-6.158646	-2.347605	-1.228764	H	-6.163321	-2.330069	-1.262827	H	-6.175708	-2.332164	-1.250096
H	-7.863131	-2.111513	-0.886626	H	-7.864323	-2.092295	-0.905181	H	-7.878179	-2.093666	-0.899441
C	-12.112224	-0.352849	0.335344	C	-12.101357	-0.337932	0.370385	C	-12.119022	-0.333081	0.351038
H	-12.250457	0.071813	-0.662340	H	-12.252964	0.103238	-0.618171	H	-12.264109	0.103444	-0.640552
H	-11.544676	-1.276591	0.232227	H	-11.536594	-1.260463	0.243906	H	-11.553931	-1.256476	0.232586
H	-13.096699	-0.609021	0.728536	H	-13.080596	-0.599555	0.773002	H	-13.100946	-0.592212	0.748726
C	9.241507	2.289554	-0.359983	C	9.153068	2.326120	-0.348937	C	9.129926	2.411098	-0.405490
H	9.967049	1.813697	-1.025241	H	8.906091	3.205547	-0.921599	H	9.218604	2.491090	-1.496646
H	8.860163	3.191282	-0.836539	H	10.120794	2.334741	0.132426	H	8.776247	3.390373	-0.061555
H	9.810433	2.567361	0.530633	C	6.380957	2.727266	-1.149985	H	10.135004	2.281917	0.002697
C	6.364235	2.782375	-1.000061	H	5.316212	2.721228	-1.366851	C	6.358031	2.682004	-1.115467
H	5.290899	2.766141	-1.166148	H	6.628053	3.648508	-0.608938	H	5.315685	2.810899	-1.354434
H	6.606345	3.679803	-0.426141	H	6.934936	2.774768	-2.094743	H	7.054502	3.425726	-1.468071
H	6.859902	2.879378	-1.968326	C	8.032233	-1.978787	1.838581	C	8.030049	-2.008007	1.826201
C	8.044959	-2.077698	1.740284	H	7.711069	-1.963726	2.884587	H	7.718352	-2.006646	2.879207
H	7.730723	-2.108567	2.790263	H	7.602408	-2.876631	1.387891	H	7.616908	-2.920264	1.383047
H	7.642772	-2.976455	1.262546	H	9.115453	-2.065006	1.821248	H	9.114044	-2.096133	1.811696
H	9.132334	-2.114656	1.713633	O	9.850814	-0.010654	1.000316	O	9.842250	-0.039259	1.010529
O	9.853712	-0.046919	0.935425	H	10.325701	0.502909	0.333910	H	10.370871	0.596948	0.529638
H	5.398119	-1.950314	2.184796	H	5.335563	-1.850333	2.282740	H	5.380768	-1.842796	2.244137
H	3.080654	-1.900247	1.045435	H	3.039784	-1.816369	1.130351	H	3.061701	-1.845837	1.109040
C	3.984619	-1.083638	-1.499909	C	3.985913	-1.212902	-1.457271	C	3.966215	-1.183498	-1.476307

H	3.442504	-2.030516	-1.505157	H	3.425434	-2.147505	-1.400978	H	3.419351	-2.126911	-1.424708
H	3.723380	-0.520972	-2.397537	H	3.749715	-0.713153	-2.397808	H	3.708529	-0.671294	-2.403939
H	5.052594	-1.293649	-1.527452	H	5.050080	-1.444278	-1.450805	H	5.033542	-1.398343	-1.493304
H	-1.469097	-1.359460	0.253270	H	-1.461756	-1.328698	0.245468	H	-1.476646	-1.338215	0.262508
C	-1.418484	-1.801790	-1.840797	C	-1.432790	-1.846108	-1.831761	C	-1.441979	-1.840569	-1.818365
H	-2.287565	-2.458782	-1.843526	H	-2.299690	-2.505098	-1.797048	H	-2.310140	-2.498454	-1.792956
H	-1.390342	-1.272981	-2.797490	H	-1.421870	-1.351989	-2.807263	H	-1.424928	-1.339226	-2.790067
H	-0.532729	-2.432114	-1.782147	H	-0.544456	-2.471903	-1.765254	H	-0.554691	-2.467848	-1.751831
H	-13.261071	1.551762	1.949661	H	-13.224866	1.542243	2.031011	H	-13.251924	1.555443	1.995512
C	-0.236125	0.113211	-0.684999	C	-0.227255	0.100490	-0.755728	C	-0.241658	0.100120	-0.725861
H	-0.239817	0.717410	-1.599495	H	-0.220881	0.652522	-1.702513	H	-0.239128	0.665491	-1.664693
H	-0.329625	0.819279	0.145680	H	-0.320205	0.852242	0.033881	H	-0.333419	0.840909	0.074170
**α-Tocopherol (C3•)**				**α-Tocopherol (C4•)**				**α-Tocopherol (C5•)**			
C	3.641462	-0.294023	-0.247075	C	-3.642755	-0.239240	0.286166	C	-3.271554	-0.983579	-0.380922
C	5.861987	0.555986	0.116117	C	-5.836727	0.591387	-0.177682	C	-7.113189	-0.694601	0.124754
C	6.197487	-0.567564	0.861305	C	-6.177567	-0.643375	-0.828010	C	-6.542625	0.269350	-0.772546
C	5.155162	-1.540098	1.316680	C	-5.186160	-1.547141	-1.174151	C	-5.368579	-0.209957	-1.596556
C	3.738549	-1.025369	1.089932	C	-3.749788	-1.245718	-0.866488	C	-4.057712	-0.028460	-0.877100
C	6.794556	1.490407	-0.331028	C	-6.765930	1.519322	0.230465	C	-8.233159	-0.243946	0.904612
C	7.567670	-0.871546	1.172716	C	-7.579558	-0.860172	-1.044481	C	-7.026439	1.572018	-0.868077
H	5.299123	-2.505212	0.807031	H	-5.431198	-2.485254	-1.647413	H	-5.525175	-1.264153	-1.820437
H	3.472944	-0.306462	1.869898	H	-3.191282	-0.814415	-1.715651	H	-3.751400	1.004772	-0.724356
C	8.504019	0.120316	0.725023	C	-8.492268	0.094675	-0.643657	C	-8.114009	1.957811	-0.085069
C	8.147540	1.246411	0.025454	C	-8.142509	1.293635	-0.010725	C	-8.724553	1.051322	0.785328
O	4.531024	0.816775	-0.219635	O	-4.484492	0.883540	-0.000414	O	-6.655188	-1.880288	0.213874
C	2.267958	0.350993	-0.444622	C	-2.248394	0.381926	0.375128	C	-1.985037	-0.634863	0.324643
H	2.328326	0.986675	-1.333240	H	-2.240093	1.087629	1.212227	H	-2.018490	-1.025050	1.349316
H	2.090068	1.017117	0.404083	H	-2.098429	0.970213	-0.534132	H	-1.883809	0.450699	0.399292
C	1.098496	-0.617743	-0.592902	C	-1.100453	-0.611314	0.528471	C	-0.746059	-1.211649	-0.366748
H	1.277687	-1.272082	-1.447687	H	-1.287052	-1.256478	1.389211	H	-0.872407	-2.290903	-0.471910
H	1.032849	-1.262680	0.288254	H	-1.061092	-1.262220	-0.349677	H	-0.684513	-0.803579	-1.380778
C	-1.470435	-0.786541	-0.750220	C	1.472316	-0.818284	0.633668	C	1.833999	-1.299657	-0.334759
C	-2.740927	0.067906	-0.802662	C	2.750410	0.022206	0.715987	C	3.050366	-0.800754	0.451439
H	-2.772290	0.596722	-1.762292	H	2.782917	0.519052	1.692656	H	3.054270	-1.278991	1.437790
H	-2.671842	0.840190	-0.030609	H	2.688983	0.820330	-0.030101	H	2.929379	0.271605	0.632759
C	-4.044077	-0.703574	-0.609119	C	4.050598	-0.749134	0.502705	C	4.398460	-1.035471	-0.226494
H	-3.986205	-1.279981	0.320816	H	3.995986	-1.293781	-0.446319	H	4.360404	-0.644568	-1.249258
H	-4.163411	-1.427081	-1.416006	H	4.162738	-1.499203	1.286087	H	4.582520	-2.107139	-0.310128
C	-5.264719	0.211955	-0.558022	C	5.272727	0.165854	0.490540	C	5.551384	-0.371699	0.522230
H	-5.079429	1.004258	0.173484	H	5.097401	0.974595	-0.225243	H	5.310309	0.685548	0.668098
H	-5.383103	0.711318	-1.526560	H	5.375472	0.643426	1.471881	H	5.631329	-0.807172	1.524992
C	-6.581422	-0.483238	-0.196814	C	6.597458	-0.517214	0.135286	C	6.915463	-0.469233	-0.168203
H	-6.445460	-0.968562	0.777040	H	6.479671	-0.982100	-0.850807	H	6.804795	-0.068420	-1.182928
C	-7.695041	0.558165	-0.047630	C	7.707189	0.533677	0.028632	C	7.935881	0.405955	0.566271
H	-7.355645	1.331812	0.647671	H	7.378016	1.317592	-0.660033	H	7.535517	1.421908	0.632802
H	-7.842559	1.057090	-1.012277	H	7.829066	1.016452	1.005098	H	8.032886	0.047476	1.597466
C	-9.032679	0.009555	0.443329	C	9.060811	0.005460	-0.439832	C	9.318385	0.463367	-0.079349
H	-8.873216	-0.562974	1.363759	H	8.931086	-0.542378	-1.379705	H	9.209478	0.685165	-1.146929
H	-9.434438	-0.688630	-0.292028	H	9.446607	-0.709975	0.287430	H	9.794660	-0.515817	-0.012188
C	-10.052768	1.113284	0.707732	C	10.080850	1.122456	-0.642916	C	10.219758	1.514514	0.562512
H	-9.609867	1.849435	1.385345	H	9.653202	1.875324	-1.311887	H	9.694027	2.474046	0.566426
H	-10.261669	1.645023	-0.227851	H	10.257843	1.627765	0.313652	H	10.389893	1.254940	1.613964
C	-11.378102	0.634954	1.306212	C	11.427169	0.670353	-1.214000	C	11.575670	1.703056	-0.122649
H	-11.153789	0.118209	2.245411	H	11.236752	0.188920	-2.179050	H	11.386285	1.965238	-1.168988
C	-12.277508	1.828406	1.620913	C	12.331652	1.877562	-1.452441	C	12.341842	2.852666	0.527896
H	-11.784183	2.530304	2.294101	H	11.858900	2.603088	-2.115296	H	11.766249	3.778473	0.500185
H	-12.528827	2.365294	0.703277	H	12.548010	2.379877	-0.506658	H	12.552559	2.621374	1.574780
C	-6.949894	-1.558586	-1.218409	C	6.954555	-1.612292	1.139712	C	7.391765	-1.917151	-0.277590
H	-7.096392	-1.106699	-2.203349	H	7.090391	-1.179312	2.134607	H	7.537430	-2.342087	0.719266
H	-6.167749	-2.310631	-1.308068	H	6.170734	-2.364919	1.206028	H	6.668353	-2.537278	-0.804158
H	-7.868309	-2.076602	-0.945670	H	7.875648	-2.125763	0.867288	H	8.336212	-1.989528	-0.815599
C	-12.103893	-0.343580	0.384301	C	12.125672	-0.340583	-0.306052	C	12.415491	0.427084	-0.095472
H	-12.269821	0.113977	-0.594469	H	12.254342	0.078272	0.695360	H	12.568685	0.098031	0.935534
H	-11.538175	-1.261987	0.234321	H	11.559989	-1.266549	-0.213377	H	11.940158	-0.386826	-0.640965
H	-13.076944	-0.615255	0.795202	H	13.114165	-0.591258	-0.692753	H	13.396541	0.598195	-0.540258
C	9.231286	2.223399	-0.372278	C	-9.211181	2.281505	0.377930	C	-9.915527	1.488595	1.604419
H	9.918261	1.800354	-1.114831	H	-9.917012	1.886456	1.123019	H	-10.394402	2.360273	1.162143
H	8.814789	3.128495	-0.806304	H	-8.779513	3.179183	0.814736	H	-10.653328	0.689153	1.673330
H	9.830715	2.527520	0.491610	H	-9.811615	2.618176	-0.479157	H	-9.629333	1.742061	2.630776
C	6.381875	2.694222	-1.135432	C	-6.306609	2.783069	0.916921	C	-8.853501	-1.229063	1.858702
H	5.320562	2.640435	-1.364417	H	-5.230214	2.766877	1.059408	H	-8.195488	-2.092420	1.932832
H	6.551953	3.640358	-0.606530	H	-6.558010	3.671056	0.330453	H	-9.002995	-0.800879	2.853838
H	6.920342	2.765994	-2.086907	H	-6.782133	2.897577	1.894335	H	-9.831192	-1.581721	1.511236
C	7.972606	-2.012696	1.844470	C	-8.005076	-2.130497	-1.710989	C	-6.395179	2.562580	-1.818951
H	7.264490	-2.757816	2.170711	H	-7.535278	-2.228567	-2.697154	H	-5.313295	2.610360	-1.683476

H	9.016456	-2.177948	2.054764	H	-7.674348	-3.001679	-1.131598	H	-6.573532	2.277613	-2.859152
O	9.833624	-0.132501	1.046506	H	-9.084572	-2.179114	-1.830877	H	-6.809984	3.555848	-1.669998
H	10.380309	0.494440	0.573095	O	-9.837468	-0.168170	-0.892745	O	-8.617708	3.270392	-0.199985
H	5.305678	-1.759908	2.379811	H	-10.344315	0.565033	-0.543420	H	-8.449823	3.721909	0.628897
H	3.017804	-1.844876	1.130748	H	-3.209144	-2.156836	-0.583797	H	-5.321890	0.333149	-2.544287
C	4.000877	-1.209442	-1.419506	C	-4.064988	-0.856308	1.617530	C	-3.599555	-2.454448	-0.467292
H	3.413922	-2.128775	-1.395741	H	-3.421175	-1.696123	1.882431	H	-3.213474	-2.898887	-1.389661
H	3.815495	-0.694337	-2.363621	H	-4.012719	-0.103112	2.406432	H	-3.135078	-2.989988	0.364821
H	5.056282	-1.473372	-1.370685	H	-5.087449	-1.222136	1.543911	H	-4.678694	-2.602404	-0.408945
H	-1.472038	-1.328552	0.203072	H	1.465238	-1.324086	-0.339310	H	1.837305	-0.795559	-1.308697
C	-1.436265	-1.813557	-1.881873	C	1.428707	-1.887215	1.725071	C	1.899861	-2.806758	-0.579425
H	-2.302816	-2.473563	-1.858584	H	2.287495	-2.556173	1.675311	H	2.790547	-3.088186	-1.140554
H	-1.424480	-1.304009	-2.849419	H	1.422905	-1.415687	2.711915	H	1.916314	-3.341959	0.374032
H	-0.547619	-2.439642	-1.823895	H	0.531908	-2.498771	1.642631	H	1.035095	-3.153245	-1.142449
H	-13.210614	1.508969	2.086464	H	13.281918	1.579096	-1.896735	H	13.294537	3.028691	0.027147
C	-0.230628	0.112973	-0.770756	C	0.243681	0.094665	0.691923	C	0.543345	-0.903473	0.389405
H	-0.214141	0.672722	-1.713056	H	0.253530	0.639205	1.643442	H	0.518111	-1.402541	1.365189
H	-0.329238	0.858479	0.024142	H	0.338401	0.852150	-0.092395	H	0.577657	0.170613	0.598027
**α-Tocopherol (C6•)**				**α-Tocopherol (C7•)**				**α-Tocopherol (C8•)**			
C	-3.606381	-0.306453	0.373421	C	-3.536292	-0.198628	0.223617	C	3.594291	-0.493843	-0.331760
C	-5.830404	0.514885	-0.084382	C	-5.849653	0.546145	-0.095754	C	5.803780	0.476090	-0.217124
C	-6.182459	-0.650444	-0.775142	C	-6.219404	-0.643260	-0.738101	C	6.072672	-0.159651	0.999581
C	-5.118957	-1.661545	-1.116393	C	-5.154923	-1.630835	-1.144664	C	4.960942	-0.861962	1.741196
C	-3.728616	-1.060634	-0.974600	C	-3.772355	-0.989476	-1.073209	C	3.596797	-0.473785	1.188030
C	-6.808916	1.458150	0.266739	C	-6.829215	1.500624	0.240129	C	6.814350	1.170627	-0.901336
C	-7.518581	-0.881188	-1.113923	C	-7.568075	-0.903095	-1.009612	C	7.368152	-0.130267	1.526486
H	-5.206744	-2.509696	-0.430487	H	-5.196151	-2.508732	-0.489931	H	5.103443	-1.945722	1.671062
H	-3.544071	-0.366685	-1.805083	H	-3.647937	-0.283408	-1.897022	H	3.347297	0.543745	1.500304
C	-8.471549	0.074925	-0.793390	C	-8.520942	0.043294	-0.668062	C	8.359043	0.560448	0.844505
C	-8.132770	1.248100	-0.120389	C	-8.167125	1.247150	-0.057060	C	8.097124	1.219911	-0.356625
O	-4.556504	0.812881	0.243822	O	-4.577922	0.862897	0.221442	O	4.574135	0.483842	-0.789243
C	-2.256684	0.417343	0.424222	C	-2.204380	0.474469	0.298179	C	2.247822	-0.043071	-0.896677
H	-2.338374	1.163004	1.217814	H	-1.966542	0.886448	-0.693389	H	2.440539	0.180201	-1.972165
H	-2.085921	0.945651	-0.522270	C	-1.088643	-0.451239	0.728469	H	2.023187	0.927621	-0.436646
C	-1.081469	-0.501648	0.727805	H	-1.251705	-0.803840	1.752446	C	1.109561	-1.014243	-0.637147
H	-1.292476	-0.992867	1.677862	H	-0.983559	-1.395039	0.125674	H	1.242249	-1.876012	-1.304615
H	-1.004396	-1.284297	-0.035388	C	1.494391	-0.670936	0.818873	C	-1.462780	-1.139317	-0.529648
C	1.483871	-0.650668	0.888981	C	2.783189	0.155254	0.760172	C	-2.739294	-0.356504	-0.853672
C	2.761967	0.193074	0.852302	H	2.842159	0.778440	1.660387	H	-2.824493	-0.246071	-1.941198
H	2.805954	0.807032	1.759460	H	2.713523	0.848541	-0.083883	H	-2.632716	0.657347	-0.452544
H	2.694691	0.892663	0.013250	C	4.071705	-0.652643	0.620810	C	-4.034659	-0.960609	-0.313951
C	4.056247	-0.605727	0.717462	H	4.012906	-1.269599	-0.282917	H	-3.938353	-1.118367	0.766161
H	3.990265	-1.247492	-0.168388	H	4.165589	-1.342278	1.461041	H	-4.188243	-1.945154	-0.758650
H	4.166188	-1.271403	1.574342	C	5.311575	0.235397	0.543700	C	-5.247875	-0.075006	-0.588226
C	5.290300	0.285633	0.599659	H	5.135938	1.018031	-0.200755	H	-5.032883	0.932648	-0.218874
H	5.102670	1.049000	-0.161682	H	5.445766	0.751142	1.501666	H	-5.385180	0.022529	-1.671890
H	5.438992	0.824676	1.542408	C	6.614066	-0.487748	0.187016	C	-6.566808	-0.547275	0.032721
C	6.585750	-0.448600	0.239860	H	6.469270	-0.975129	-0.784670	H	-6.409235	-0.652832	1.112969
H	6.422545	-0.959672	-0.716611	C	7.749095	0.529734	0.034619	C	-7.649917	0.514755	-0.180920
C	7.722062	0.559089	0.040340	H	7.423989	1.309471	-0.660797	H	-7.276139	1.470533	0.198632
H	7.385755	1.325326	-0.664759	H	7.907406	1.026984	0.998526	H	-7.799833	0.652345	-1.258225
H	7.906467	1.076027	0.989061	C	9.075868	-0.044132	-0.456984	C	-8.996009	0.219522	0.476514
C	9.031480	-0.037431	-0.470649	H	8.905753	-0.612175	-1.378325	H	-8.839522	-0.010778	1.536308
H	8.831105	-0.631694	-1.369129	H	9.464327	-0.750878	0.277492	H	-9.436852	-0.670950	0.026068
H	9.435526	-0.725178	0.273211	C	10.115822	1.041509	-0.719712	C	-9.967299	1.390184	0.353325
C	10.071167	1.030827	-0.796569	H	9.685437	1.785905	-1.396411	H	-9.483377	2.291572	0.741474
H	9.626442	1.758348	-1.482351	H	10.332581	1.568791	0.216711	H	-10.167348	1.584220	-0.707022
H	10.320432	1.584312	0.116307	C	11.433988	0.543453	-1.317885	C	-11.303159	1.203224	1.076973
C	11.366114	0.502648	-1.419542	H	11.202728	0.031503	-2.258072	H	-11.087802	1.017969	2.134683
H	11.100154	-0.043389	-2.330877	C	12.352621	1.722936	-1.629848	C	-12.143124	2.474632	0.975518
C	12.279231	1.663517	-1.807381	H	11.872375	2.432093	-2.304924	H	-11.610372	3.335110	1.381744
H	11.780859	2.347451	-2.495191	H	12.608177	2.256382	-0.711313	H	-12.377564	2.689903	-0.069808
H	12.565791	2.231978	-0.919366	C	6.957960	-1.566131	1.213765	C	-6.995405	-1.905621	-0.520601
C	6.944143	-1.502625	1.286924	H	7.107781	-1.113170	2.197816	H	-7.181394	-1.830412	-1.595683
H	7.103214	-1.028141	2.259208	H	6.157566	-2.298807	1.301945	H	-6.222682	-2.656667	-0.367255
H	6.148995	-2.237569	1.400248	H	7.866882	-2.105142	0.948066	H	-7.906174	-2.269210	-0.044924
H	7.852119	-2.043583	1.022776	C	12.143403	-0.447675	-0.396742	C	-12.086889	0.009547	0.533996
C	12.102162	-0.456730	-0.485811	H	12.317460	0.006987	0.581992	H	-12.258594	0.128618	-0.539015
H	12.326691	0.039589	0.461823	H	11.560617	-1.355234	-0.246270	H	-11.557910	-0.929796	0.687085
H	11.514711	-1.347315	-0.267338	H	13.111373	-0.736980	-0.807999	H	-13.059760	-0.071469	1.020790
H	13.046612	-0.779681	-0.925622	C	-9.256585	2.238942	0.270231	C	9.221177	1.967508	-1.030876
C	-9.218055	2.255825	0.171216	H	-9.965032	1.837145	1.002783	H	10.023312	1.293517	-1.349331
H	-9.921537	1.897086	0.930964	H	-8.851027	3.154102	0.689662	H	8.878866	2.495445	-1.915182
H	-8.805171	3.192073	0.534353	H	-9.825486	2.516126	-0.622512	H	9.660420	2.712711	-0.360903
H	-9.792584	2.485847	-0.730352	C	-6.397277	2.778285	0.916280	C	6.487767	1.849139	-2.208348

C	-6.395356	2.664778	1.069829	H	-5.323228	2.764936	1.074268	H	5.461502	1.638050	-2.490302
H	-5.425044	2.479564	1.521465	H	-6.645025	3.652454	0.309617	H	6.603268	2.932786	-2.133684
H	-6.314561	3.560201	0.446659	H	-6.887652	2.901361	1.884469	H	7.143889	1.503149	-3.009033
H	-7.112172	2.875149	1.863693	C	-7.979272	-2.192188	-1.671637	C	7.679442	-0.830289	2.822988
C	-7.918881	-2.152931	-1.814864	H	-7.688547	-2.204306	-2.725086	H	7.222800	-0.310875	3.668733
H	-7.581106	-2.155312	-2.854281	H	-7.491178	-3.044038	-1.196951	H	7.280602	-1.845109	2.820296
H	-7.464808	-3.017276	-1.329295	H	-9.055493	-2.330202	-1.617138	H	8.751120	-0.875757	2.992506
H	-8.998426	-2.274410	-1.810647	O	-9.847328	-0.235980	-0.964230	O	9.626106	0.585823	1.402133
O	-9.785559	-0.166830	-1.172531	H	-10.394585	0.450648	-0.581810	H	10.222247	1.019934	0.791077
H	-10.353179	0.444990	-0.702743	H	-5.361154	-1.998908	-2.154269	H	5.023227	-0.620011	2.804526
H	-5.276726	-2.043663	-2.128005	H	-2.990495	-1.746764	-1.164098	H	2.796586	-1.127975	1.528314
H	-2.975805	-1.848546	-1.015239	C	-3.815078	-1.075382	1.446934	C	3.982670	-1.854075	-0.897914
C	-3.887318	-1.072252	1.637772	H	-3.242589	-2.004400	1.387888	H	3.286805	-2.599250	-0.512450
H	-3.228457	-1.947588	1.697272	H	-3.515338	-0.528895	2.340600	H	3.899333	-1.826355	-1.985323
H	-4.922898	-1.432118	1.644948	H	-4.873811	-1.328905	1.527310	H	5.004914	-2.132515	-0.635532
H	1.487022	-1.297988	0.003157	H	1.497720	-1.358791	-0.035722	H	-1.396308	-1.268112	0.556496
C	1.433387	-1.542825	2.129165	C	1.421939	-1.506093	2.096794	C	-1.466856	-2.525580	-1.168179
H	2.293868	-2.209862	2.186893	H	2.286384	-2.161939	2.212975	H	-2.330173	-3.124689	-0.870068
H	1.417458	-0.926976	3.032359	H	1.372000	-0.848364	2.969266	H	-1.474628	-2.432631	-2.259663
H	0.535501	-2.157621	2.133699	H	0.527905	-2.127601	2.098601	H	-0.562242	-3.057562	-0.876593
H	13.192975	1.306717	-2.284202	H	13.282858	1.389519	-2.091700	H	-13.086068	2.372062	1.514236
C	0.246499	0.248774	0.797690	C	0.272269	0.245245	0.681230	C	-0.214504	-0.353955	-0.958563
H	0.233732	0.923966	1.661549	H	0.299991	1.008669	1.466195	H	-0.358336	-0.120258	-2.045162
H	0.346320	0.885752	-0.087923	H	0.350037	0.784646	-0.268435	H	-0.259427	0.621992	-0.456445
**α-Tocopherol (C9$^{\bullet}$)**				**α-Tocopherol (C10$^{\bullet}$)**				**α-Tocopherol (C11$^{\bullet}$)**			
C	3.607025	-0.467273	-0.228093	C	-3.595683	-0.273417	0.334104	C	3.450190	0.662697	-0.247706
C	5.788104	0.534113	-0.051873	C	-5.835514	0.541691	-0.051496	C	5.600912	-0.123007	0.513895
C	6.193396	-0.405201	0.893623	C	-6.231633	-0.670252	-0.618809	C	5.758708	-0.905536	-0.625286
C	5.187927	-1.345705	1.510672	C	-5.203447	-1.726260	-0.937787	C	4.617912	-1.108012	-1.582323
C	3.759733	-0.896243	1.228528	C	-3.788486	-1.163813	-0.888131	C	3.295863	-0.669401	-0.971012
C	6.695821	1.436199	-0.619171	C	-6.769413	1.550474	0.223211	C	6.644171	0.048161	1.428922
C	7.539396	-0.477509	1.266085	C	-7.584603	-0.901227	-0.888279	C	6.985605	-1.508627	-0.888051
H	5.347409	-2.357376	1.124442	H	-5.301965	-2.555807	-0.230238	H	4.811080	-0.553434	-2.506542
H	3.510628	-0.035554	1.852791	H	-3.593798	-0.557097	-1.775611	H	2.956008	-1.407746	-0.236784
C	8.437501	0.416832	0.699728	C	-8.503890	0.099910	-0.612438	C	8.015920	-1.338573	0.015721
C	8.033380	1.376049	-0.230046	C	-8.114186	1.325180	-0.069231	C	7.863510	-0.579890	1.175303
O	4.481428	0.647212	-0.461168	O	-4.533941	0.825091	0.241302	O	4.402014	0.492404	0.814355
C	2.218615	0.101543	-0.512764	C	-2.224937	0.395511	0.337189	C	2.165199	1.049481	0.484873
H	2.266170	0.605288	-1.481312	H	-2.203320	1.098648	1.174791	H	2.397336	1.902444	1.128446
H	2.006630	0.870874	0.233267	H	-2.144463	0.992506	-0.576032	H	1.904878	0.218975	1.147152
C	1.084414	-0.920294	-0.536444	C	-1.035276	-0.555223	0.447022	C	0.974137	1.397699	-0.404526
H	1.258026	-1.635383	-1.350310	H	-1.131426	-1.184370	1.332759	H	1.243117	2.218249	-1.072059
H	1.090059	-1.523878	0.380872	H	-0.998817	-1.220149	-0.422119	H	0.700584	0.551309	-1.038395
C	-1.519485	-1.070778	-0.605152	C	1.510456	-0.746408	0.512054	C	-1.483246	2.031835	-0.396583
C	-2.737914	-0.147782	-0.705155	C	2.767383	0.130119	0.582216	C	-2.828380	2.058837	0.261038
H	-2.702070	0.370508	-1.669553	H	2.750779	0.665794	1.538392	H	-3.253848	3.076476	0.240959
H	-2.647944	0.625347	0.063245	H	2.738078	0.877278	-0.224185	H	-2.734433	1.795428	1.316456
C	-4.085819	-0.847475	-0.558486	C	4.069783	-0.654355	0.480787	C	-3.822475	1.104910	-0.428423
H	-4.075347	-1.475840	0.339156	H	4.047168	-1.275070	-0.423696	H	-3.442622	0.082567	-0.358053
H	-4.240015	-1.517317	-1.405119	H	4.119400	-1.332344	1.332441	H	-3.864774	1.347995	-1.494463
C	-5.246224	0.140172	-0.470998	C	5.302780	0.245356	0.448585	C	-5.216968	1.162280	0.177766
H	-5.039928	0.855655	0.330525	H	5.158154	1.019877	-0.312486	H	-5.128323	1.044540	1.263457
H	-5.292187	0.723954	-1.397571	H	5.388700	0.771713	1.406990	H	-5.653761	2.152722	0.010816
C	-6.618626	-0.492587	-0.218430	C	6.626509	-0.470817	0.160094	C	-6.191731	0.107281	-0.355645
H	-6.563844	-1.041011	0.729575	H	6.526627	-0.973991	-0.809633	H	-5.757670	-0.880458	-0.168322
C	-7.674195	0.606709	-0.062200	C	7.762258	0.550365	0.041833	C	-7.516029	0.188756	0.408722
H	-7.310999	1.335731	0.668377	H	7.463575	1.320614	-0.676218	H	-7.298462	0.132531	1.480781
H	-7.765364	1.145675	-1.012186	H	7.877679	1.059682	1.005732	H	-7.953392	1.177713	0.237169
C	-9.053738	0.121514	0.375757	C	9.111815	-0.021683	-0.386110	C	-8.548713	-0.878507	0.053061
H	-8.955098	-0.478439	1.287277	H	8.985119	-0.593415	-1.312385	H	-8.101604	-1.874297	0.136105
H	-9.470408	-0.538458	-0.386039	H	9.466764	-0.725682	0.367809	H	-8.856473	-0.764378	-0.987118
C	-10.023983	1.271986	0.632261	C	10.162317	1.064098	-0.603738	C	-9.768876	-0.788751	0.959941
H	-9.541030	2.007318	1.282913	H	9.757641	1.814495	-1.289870	H	-9.433449	-0.809114	1.997774
H	-10.231973	1.788080	-0.312000	H	10.345727	1.584108	0.343914	H	-10.243903	0.188358	0.807581
C	-11.353542	0.856298	1.266198	C	11.500896	0.569088	-1.157417	C	-10.835456	-1.882558	0.778217
H	-11.131061	0.382874	2.228370	H	11.304753	0.076313	-2.115744	H	-10.365328	-2.857778	0.956847
C	-12.221324	2.085158	1.528221	C	12.439494	1.747230	-1.409633	C	-11.940497	-1.696439	1.810320
H	-11.694868	2.818724	2.139757	H	11.991294	2.472981	-2.089321	H	-11.536298	-1.702772	2.821852
H	-12.490464	2.566936	0.585493	H	12.660668	2.260764	-0.470941	H	-12.443210	-0.738941	1.652419
C	-6.991768	-1.481963	-1.322246	C	6.931784	-1.532320	1.216260	C	-6.405925	0.262939	-1.860950
H	-6.984747	-0.981369	-2.294246	H	7.049935	-1.062221	2.196557	H	-6.840905	1.246718	-2.067084
H	-6.292452	-2.315342	-1.369346	H	6.120672	-2.253899	1.291074	H	-5.467777	0.183495	-2.407413
H	-7.984321	-1.901964	-1.167974	H	7.846237	-2.081845	0.990720	H	-7.078971	-0.492562	-2.264760
C	-12.109814	-0.151352	0.402108	C	12.165660	-0.445036	-0.228115	C	-11.419238	-1.891508	-0.636552
H	-12.274798	0.258536	-0.597709	H	12.300415	-0.012173	0.766595	H	-11.830346	-0.906591	-0.867163

H	-11.566719	-1.089544	0.296234	H	11.569596	-1.350123	-0.121822	H	-10.672109	-2.134354	-1.389786
H	-13.084845	-0.377643	0.834808	H	13.148438	-0.733083	-0.604140	H	-12.225010	-2.619533	-0.731825
C	9.072478	2.314289	-0.789409	C	-9.173138	2.367435	0.190649	C	9.040464	-0.472912	2.113818
H	9.848083	1.770452	-1.336821	H	-9.907325	2.019369	0.924321	H	9.893911	0.018721	1.639305
H	8.642455	3.033329	-1.478479	H	-8.748281	3.287363	0.578669	H	8.805289	0.094974	3.009747
H	9.562717	2.881744	0.006378	H	-9.713547	2.621760	-0.725555	H	9.365230	-1.466537	2.438551
C	6.218918	2.450427	-1.628102	C	-6.304379	2.853146	0.824086	C	6.436731	0.888845	2.664305
H	5.149900	2.359491	-1.785051	H	-5.233220	2.831000	0.991217	H	5.449180	1.335465	2.659196
H	6.427408	3.466991	-1.292166	H	-6.528473	3.694306	0.165582	H	6.528854	0.286090	3.570429
H	6.717326	2.313703	-2.589017	H	-6.795089	3.044231	1.779936	H	7.170299	1.691458	2.734326
C	8.003370	-1.500513	2.267702	C	-8.035086	-2.211374	-1.477147	C	7.178496	-2.340711	-2.127660
H	7.624163	-1.272638	3.266215	H	-7.746033	-2.289181	-2.527866	H	6.638203	-3.286941	-2.052377
H	7.642131	-2.495499	2.005318	H	-7.575762	-3.051059	-0.955165	H	6.798871	-1.825782	-3.012430
H	9.086948	-1.528796	2.318721	H	-9.114612	-2.311822	-1.417292	H	8.228812	-2.566576	-2.283092
O	9.750638	0.332202	1.096940	O	-9.828666	-0.152319	-0.905872	O	9.204491	-1.967409	-0.274739
H	10.284317	0.956378	0.587732	H	-10.363567	0.575433	-0.586413	H	9.867968	-1.761137	0.389566
H	5.352669	-1.406981	2.587037	H	-5.403696	-2.148445	-1.924157	H	4.563892	-2.156913	-1.873698
H	3.055239	-1.693449	1.463900	H	-3.056599	-1.969179	-0.871713	H	2.530719	-0.590077	-1.739935
C	3.966884	-1.588726	-1.200481	C	-3.861384	-1.023500	1.636721	C	3.943013	1.763777	-1.180810
H	3.415583	-2.499273	-0.965078	H	-3.259284	-1.929846	1.683808	H	3.330488	1.808345	-2.079345
H	3.727685	-1.287597	-2.220456	H	-3.596870	-0.393256	2.484947	H	3.901484	2.731818	-0.681511
H	5.031944	-1.811293	-1.153686	H	-4.913483	-1.296295	1.722591	H	4.974281	1.580780	-1.481700
H	-1.546866	-1.559984	0.380325	H	1.514339	-1.169399	-0.525905	C	-1.351585	2.734597	-1.707281
C	-1.560818	-2.189940	-1.658604	C	1.469612	-1.769034	1.622039	H	-2.317009	2.821267	-2.209330
H	-2.438735	-2.821945	-1.530967	H	2.341824	-2.428880	1.611829	H	-0.962477	3.751700	-1.575025
H	-1.587570	-1.760190	-2.662387	H	0.579271	-2.403383	1.572522	H	-0.665174	2.223442	-2.385546
H	-0.681496	-2.828959	-1.587683	H	13.385830	1.416150	-1.839992	H	-12.687970	-2.487147	1.736621
H	-13.145266	1.815591	2.040529	C	0.292723	0.185623	0.548110	C	-0.249240	1.780895	0.417441
C	-0.252822	-0.282698	-0.698511	H	0.316425	0.737868	1.494404	H	-0.002382	2.673618	1.020321
H	-0.304959	0.775726	-0.922582	H	0.360889	0.914533	-0.272326	H	-0.449675	0.994569	1.147751
**α-Tocopherol (C12•)**				**α-Tocopherol (C13•)**				**α-Tocopherol (C14•)**			
C	-3.603319	-0.369390	0.256394	C	-3.57917600	0.16262400	-0.21947400	C	3.551007	-0.467597	-0.198396
C	-5.780602	0.598853	-0.069701	C	-5.88576700	-0.44520900	0.09710200	C	5.757572	0.506946	-0.141754
C	-6.299126	-0.614616	-0.515826	C	-6.24842200	0.87805300	0.33940000	C	6.099841	-0.229105	0.989013
C	-5.378694	-1.767330	-0.830591	C	-5.18922200	1.93421000	0.53567500	C	5.046246	-1.002087	1.738825
C	-3.925493	-1.315065	-0.896705	C	-3.81314600	1.31659900	0.75178700	C	3.644690	-0.586723	1.314793
C	-6.607664	1.697370	0.175834	C	-6.84898900	-1.44673900	-0.06971100	C	6.711618	1.257188	-0.835935
C	-7.675985	-0.760316	-0.675681	C	-7.60119500	1.22712700	0.39405200	C	7.425721	-0.247832	1.427279
H	-5.498287	-2.553218	-0.078310	H	-5.17049000	2.59799700	-0.33437800	H	5.186406	-2.070555	1.568158
H	-3.753406	-0.768584	-1.826781	H	-3.73894300	0.91531500	1.76494400	H	3.400143	0.387064	1.736006
C	-8.493458	0.324184	-0.422677	C	-8.55436800	0.23149200	0.23090200	C	8.366143	0.499609	0.738404
C	-7.977587	1.558205	-0.018243	C	-8.19749900	-1.09992800	0.00685700	C	8.026848	1.259034	-0.379402
O	-4.436368	0.792929	0.131134	O	-4.57254600	-0.84382800	0.03144000	O	4.475413	0.561185	-0.621167
C	-2.186816	0.191569	0.153669	C	-2.26126600	-0.55649500	0.05797200	C	2.191038	0.053688	-0.644294
H	-2.099813	0.996808	0.887972	H	-2.25037800	-1.46287000	-0.55370400	H	2.261740	0.275502	-1.709433
H	-2.081628	0.654638	-0.830957	H	-2.27230500	-0.88372600	1.10077300	H	2.013558	1.001369	-0.136450
C	-1.059685	-0.814543	0.370036	C	-0.99769500	0.25346800	-0.21576900	C	1.014021	-0.886752	-0.403004
H	-1.169460	-1.281192	1.350302	H	-0.99489000	0.58933600	-1.25406300	H	1.189255	-1.829977	-0.914933
H	-1.120480	-1.615918	-0.371925	H	-0.98162400	1.15298200	0.40682000	H	0.920489	-1.113294	0.659679
C	1.512101	-1.073015	0.434004	C	1.57090600	0.22412800	-0.03164600	C	-1.551480	-1.086205	-0.545308
C	2.782996	-0.298807	0.288029	C	2.76007900	-0.68793000	0.29177100	C	-2.804195	-0.322658	-0.984628
H	2.739995	0.783768	0.303440	H	2.84311000	-1.45682100	-0.48628400	H	-2.793325	-0.202492	-2.069729
C	4.083619	-0.958115	-0.012330	H	2.53827600	-1.23655700	1.22014500	H	-2.761913	0.685095	-0.566237
H	4.137528	-1.212656	-1.083150	C	4.06047400	0.02644500	0.42296100	C	-4.136967	-0.946148	-0.576535
H	4.134881	-1.919065	0.509102	H	4.05203200	1.02005300	0.86039000	H	-4.045250	-1.251216	0.497638
C	5.290746	-0.091834	0.349075	C	5.35749900	-0.70491600	0.38019400	H	-4.262225	-1.883698	-1.121580
H	5.110273	0.921319	-0.021945	H	5.51036200	-1.25041300	1.32416100	C	-5.301942	-0.020927	-0.834639
H	5.360233	-0.008745	1.438797	H	5.31633100	-1.47677700	-0.39833900	H	-5.092964	0.942093	-0.354796
C	6.630593	-0.580118	-0.207992	C	6.57684900	0.19130000	0.13242900	C	-6.601668	-0.560057	-0.292346
H	6.538591	-0.616349	-1.300155	H	6.56333600	0.98581000	0.88799700	H	-6.495865	-0.919303	0.761320
C	7.727716	0.430031	0.138970	C	7.86784600	-0.60956100	0.31438800	C	-7.665527	0.539295	-0.270233
H	7.401766	1.422111	-0.187918	H	7.85860400	-1.06885200	1.30750600	H	-7.239607	1.410208	0.232038
H	7.823632	0.486249	1.229305	H	7.87409200	-1.43640000	-0.40541700	H	-7.853896	0.840677	-1.302445
C	9.097317	0.141556	-0.469820	C	9.15194800	0.19996600	0.15864000	C	-8.982995	0.177216	0.412346
H	8.994603	0.025165	-1.554278	H	9.11653000	1.06774800	0.82686800	H	-8.783282	-0.162345	1.431474
H	9.479394	-0.805896	-0.087763	H	9.21575900	0.59276000	-0.85708600	H	-9.447843	-0.656790	-0.109854
C	10.101650	1.252210	-0.174639	C	10.39961600	-0.62383200	0.46569800	C	-9.950680	1.356437	0.457244
H	9.683912	2.204930	-0.514537	H	10.27187100	-1.11287600	1.43619700	H	-9.428046	2.220926	0.870911
H	10.227199	1.346883	0.910416	H	10.49237200	-1.42822800	-0.27307000	H	-10.235338	1.629721	-0.562369
C	11.479271	1.069344	-0.815161	C	11.70611900	0.17354100	0.48994600	C	-11.222377	1.121858	1.272826
H	11.337667	0.988582	-1.898362	H	11.60841400	0.94796200	1.25811600	H	-10.923222	0.862871	2.290399
C	12.358095	2.286588	-0.537037	C	12.87202800	-0.73518000	0.87210500	C	-12.062355	2.395157	1.325310
H	11.887652	3.205142	-0.889356	H	12.69749400	-1.22296800	1.83173600	H	-11.490468	3.224214	1.736519
H	12.530063	2.390602	0.536902	H	13.00596700	-1.51539100	0.11917300	H	-12.378332	2.672764	0.319919
C	6.966808	-1.983869	0.292112	C	6.49552800	0.83919000	-1.24894900	C	-7.104766	-1.765023	-1.096191

H	7.056506	-1.983553	1.381759	H	6.60291400	0.07831000	-2.02665300	H	-7.340936	-1.437041	-2.108899
H	6.192990	-2.700074	0.018957	H	5.53246500	1.32913800	-1.39198000	H	-6.329678	-2.523971	-1.166066
H	7.907081	-2.345911	-0.120897	H	7.27607100	1.58421400	-1.39579200	H	-7.984954	-2.231829	-0.647915
C	12.175966	-0.203486	-0.336638	C	11.98434600	0.85976400	-0.84621200	C	-12.054610	-0.027945	0.711540
H	12.303011	-0.182021	0.748606	H	12.00969100	0.12187200	-1.65217800	H	-12.306791	0.172342	-0.330118
H	11.614152	-1.100042	-0.594845	H	11.22361600	1.59997300	-1.09080400	H	-11.514720	-0.968938	0.751041
H	13.165086	-0.294408	-0.786816	H	12.94917300	1.36745800	-0.82801500	H	-12.983691	-0.143647	1.267757
C	-8.931538	2.704534	0.201215	C	-9.29549900	-2.12097400	-0.15275600	C	9.106545	2.059335	-1.060705
H	-9.628816	2.496766	1.018090	H	-9.92359800	-1.89827000	-1.02029500	H	9.881074	1.412712	-1.476823
H	-8.410758	3.623398	0.450038	H	-8.90170100	-3.12217900	-0.29141000	H	8.710235	2.650380	-1.875260
H	-9.520225	2.905547	-0.698185	H	-9.94173500	-2.14864500	0.72900000	H	9.582740	2.749097	-0.362936
C	-6.010964	3.002363	0.638306	C	-6.41977300	-2.86972800	-0.32354400	C	6.301862	2.047878	-2.051334
H	-4.940435	2.903606	0.779474	H	-5.33935600	-2.93735300	-0.38663600	H	5.261545	1.865866	-2.282696
H	-6.184338	3.794929	-0.091514	H	-6.75394600	-3.53042600	0.47792400	H	6.430233	3.115920	-1.885279
H	-6.452102	3.324105	1.582634	H	-6.84040500	-3.24732000	-1.25651900	H	6.899826	1.772054	-2.917184
C	-8.263113	-2.072986	-1.116486	C	-8.01704000	2.65310400	0.63590400	C	7.824655	-1.055474	2.631718
H	-8.031827	-2.275817	-2.164622	H	-7.82316700	2.94777500	1.66984800	H	7.460744	-0.594017	3.548873
H	-7.856456	-2.898570	-0.531446	H	-7.45906000	3.33676400	-0.00429600	H	7.404530	-2.056688	2.582496
H	-9.343153	-2.069647	-1.005941	H	-9.07690400	2.78543400	0.44338200	H	8.902291	-1.133055	2.698993
O	-9.840355	0.145168	-0.607444	O	-9.87485900	0.60508900	0.30347300	O	9.665542	0.477140	1.202299
H	-10.294627	0.939634	-0.384616	H	-10.43322100	-0.15747200	0.14435800	H	10.220816	0.969718	0.598561
H	-5.669039	-2.218641	-1.780588	H	-5.45202800	2.56443700	1.38640200	H	5.170948	-0.845991	2.808018
H	-3.253713	-2.172551	-0.884563	H	-3.03563500	2.07089200	0.63440700	H	2.911522	-1.301832	1.675407
C	-3.857318	-1.016188	1.617252	C	-3.68575600	0.60783500	-1.67670400	C	3.918996	-1.766363	-0.904989
H	-3.372119	-1.990157	1.681476	H	-3.04462000	1.46772900	-1.86970300	H	3.341479	-2.595366	-0.504777
H	-3.466220	-0.379339	2.410628	H	-3.38899400	-0.20702000	-2.33687700	H	3.709736	-1.675028	-1.967500
H	-4.924963	-1.152616	1.784612	H	-4.71099800	0.88500200	-1.91887600	H	4.975625	-1.986510	-0.780226
H	1.467562	-1.828861	-0.362936	H	1.54182500	1.01473000	0.72747100	H	-1.588969	-1.200519	0.541512
C	1.482305	-1.818348	1.778863	C	1.74914600	0.87815500	-1.40101000	C	-1.507443	-2.479141	-1.169237
H	2.378199	-2.425024	1.907274	H	2.72410200	1.35886900	-1.47942600	H	-2.412606	-3.027935	-0.930177
H	1.442416	-1.100736	2.600814	H	1.68351200	0.12306600	-2.18903000	H	-1.442364	-2.395262	-2.255029
H	0.618813	-2.478682	1.854545	H	0.98684300	1.63230400	-1.59221000	H	-0.657418	-3.061935	-0.819000
H	13.329828	2.192766	-1.022627	H	13.80493200	-0.17488500	0.94026300	H	-12.955798	2.258170	1.932375
C	0.305895	-0.141144	0.269484	C	0.26262400	-0.56387400	0.05888800	C	-0.299038	-0.278414	-0.890796
H	0.374093	0.651155	1.022674	H	0.30608800	-1.40187900	-0.64599000	H	-0.253444	-0.147018	-1.974572
H	0.386234	0.349576	-0.704600	H	0.18628100	-1.00651000	1.05646000	H	-0.404202	0.719759	-0.463279
**α-Tocopherol (C15•)**				**α-Tocopherol (C16•)**				**α-Tocopherol (C17•)**			
C	-3.586604	-0.310263	0.332174	C	3.586357	-0.347415	-0.290314	C	3.554021	-0.372848	-0.328424
C	-5.803831	0.545373	-0.061871	C	5.795937	0.558517	0.022700	C	5.765237	0.522803	0.005837
C	-6.207206	-0.646105	-0.663229	C	6.209327	-0.570943	0.728067	C	6.098263	-0.502642	0.889351
C	-5.190055	-1.707580	-1.000387	C	5.200378	-1.604561	1.163320	C	5.034418	-1.446418	1.392144
C	-3.768214	-1.166663	-0.916839	C	3.774469	-1.083318	1.032880	C	3.635598	-0.922788	1.092388
C	-6.724557	1.557676	0.235503	C	6.707500	1.548964	-0.362987	C	6.729528	1.427930	-0.454779
C	-7.560198	-0.855022	-0.948598	C	7.563986	-0.741742	1.030082	C	7.423829	-0.653626	1.308170
H	-5.308410	-2.556049	-0.318854	H	5.322784	-2.512083	0.563596	H	5.173768	-2.431339	0.934984
H	-3.555264	-0.537228	-1.783698	H	3.559211	-0.377021	1.837680	H	3.387907	-0.108206	1.776465
C	-8.469513	0.149200	-0.649974	C	8.464139	0.241175	0.645215	C	8.375724	0.247249	0.852611
C	-8.070033	1.354601	-0.069580	C	8.053897	1.387471	-0.038362	C	8.045713	1.289637	-0.015889
O	-4.495175	0.804340	0.247697	O	4.485643	0.777052	-0.309164	O	4.484831	0.719614	-0.440639
C	-2.207334	0.341710	0.379386	C	2.201357	0.284712	-0.399344	C	2.197925	0.268620	-0.611079
H	-2.214327	1.063381	1.200918	H	2.201208	0.921120	-1.288603	H	2.274270	0.787541	-1.570567
H	-2.077627	0.917141	-0.540850	H	2.066683	0.948468	0.458589	H	2.028184	1.037482	0.146935
C	-1.032302	-0.615931	0.558978	C	1.035250	-0.696888	-0.481258	C	1.012250	-0.691785	-0.647731
H	-1.171587	-1.201005	1.469706	H	1.183604	-1.372236	-1.325608	H	1.176285	-1.445408	-1.419651
H	-0.993921	-1.328374	-0.270505	H	1.001686	-1.320032	0.417393	H	0.929604	-1.227244	0.302723
C	1.542166	-0.769229	0.622302	C	-1.536659	-0.886311	-0.562723	C	-1.555665	-0.841004	-0.825804
C	2.812369	0.087515	0.650676	C	-2.816948	-0.048118	-0.654468	C	-2.816524	0.020477	-0.955979
H	2.861638	0.609972	1.613796	H	-2.860729	0.420515	-1.644590	H	-2.822776	0.493140	-1.945448
H	2.722105	0.866569	-0.114348	H	-2.744580	0.772951	0.067088	H	-2.752175	0.837552	-0.230359
C	4.115879	-0.674590	0.427256	C	-4.115146	-0.809312	-0.392368	C	-4.134795	-0.720087	-0.740119
H	4.051647	-1.236580	-0.512735	H	-4.057179	-1.317753	0.575339	H	-4.079346	-1.284472	0.197896
H	4.274824	-1.400673	1.224132	H	-4.245504	-1.586023	-1.147875	H	-4.277068	-1.455043	-1.534650
C	5.333572	0.240087	0.382064	C	-5.352254	0.091989	-0.392464	C	-5.339642	0.218807	-0.699791
H	5.157110	1.033012	-0.359424	H	-5.175729	0.911291	0.315316	H	-5.150739	1.006500	0.037364
H	5.454103	0.714728	1.362741	H	-5.395614	0.579434	-1.409454	H	-5.447418	0.721291	-1.667176
C	6.641975	-0.489264	0.050274	C	-6.662200	-0.578244	-0.076497	C	-6.684632	-0.434212	-0.361027
H	6.511671	-0.843698	-1.004806	C	-7.725530	0.483169	0.015108	H	-6.542428	-0.936332	0.632592
C	7.752236	0.566359	-0.025380	H	-7.394669	1.280185	0.692482	C	-7.783250	0.598933	-0.318979
H	7.465978	1.348594	-0.743659	H	-7.868526	0.983262	-0.985873	H	-7.407598	1.495356	0.195891
H	7.839936	1.032862	0.962820	C	-9.109484	0.017283	0.473645	C	-9.008394	0.105501	0.413754
C	9.112236	0.006081	-0.423792	H	-8.999998	-0.498821	1.432954	H	-8.785157	-0.270573	1.449290
H	9.010773	-0.558608	-1.359092	H	-9.491135	-0.718731	-0.236956	H	-9.445138	-0.759871	-0.098880
H	9.424225	-0.701227	0.344706	C	-10.114771	1.158522	0.612581	C	-10.093592	1.174307	0.546333
C	10.170535	1.092145	-0.598208	H	-9.685077	1.927194	1.264289	H	-9.620907	2.106301	0.875051
H	9.788745	1.857332	-1.283062	H	-10.264496	1.635829	-0.363184	H	-10.508277	1.380107	-0.446576

H	10.330287	1.595041	0.363524	C	-11.481860	0.757679	1.174803	C	-11.235408	0.841333	1.511864
C	11.523132	0.603371	-1.123634	H	-11.318136	0.294159	2.153725	H	-10.802337	0.701573	2.508402
H	11.351699	0.131052	-2.097315	C	-12.365407	1.988758	1.370621	C	-12.237523	1.992886	1.584374
C	12.478772	1.778274	-1.324934	H	-11.885626	2.718349	2.024790	H	-11.742500	2.925149	1.861578
H	12.054849	2.521350	-2.002297	H	-12.549590	2.475043	0.409044	H	-12.706576	2.145036	0.608667
H	12.673481	2.271177	-0.369003	C	-7.011707	-1.557008	-1.169602	C	-6.989726	-1.566063	-1.354766
C	6.956039	-1.570279	1.056023	H	-7.094798	-1.040156	-2.169080	H	-6.948826	-1.171904	-2.374011
H	6.170964	-2.330328	1.108965	H	-6.267232	-2.347075	-1.291267	H	-6.293800	-2.408192	-1.264923
H	7.896482	-2.083629	0.836467	H	-7.966487	-2.060084	-1.001597	H	-7.996290	-1.952470	-1.193757
C	12.152565	-0.435886	-0.197683	C	-12.191626	-0.259406	0.282535	C	-11.947359	-0.453095	1.124209
H	12.267008	-0.022232	0.807657	H	-12.302708	0.142600	-0.728148	H	-12.330943	-0.376949	0.102838
H	11.534114	-1.328259	-0.119830	H	-11.630687	-1.189504	0.210930	H	-11.265957	-1.301755	1.161362
H	13.140476	-0.733526	-0.555352	H	-13.188292	-0.490089	0.665118	H	-12.790742	-0.658220	1.788522
C	-9.119887	2.398800	0.215975	C	9.092342	2.415554	-0.410497	C	9.136017	2.235173	-0.452958
H	-9.861738	2.034120	0.933169	H	9.834556	2.004561	-1.101647	H	9.921081	1.712941	-1.008252
H	-8.689641	3.302067	0.635418	H	8.649928	3.279447	-0.895213	H	8.756644	3.020116	-1.098605
H	-9.651283	2.687140	-0.695302	H	9.624767	2.779633	0.472802	H	9.604576	2.723725	0.406023
C	-6.249946	2.839422	0.872449	C	6.221837	2.763783	-1.112730	C	6.330649	2.529983	-1.403461
H	-5.177666	2.811837	1.030488	H	5.154071	2.699837	-1.289905	H	5.283029	2.441851	-1.668865
H	-6.478002	3.700351	0.242109	H	6.417689	3.678187	-0.550352	H	6.483809	3.512137	-0.952789
H	-6.732522	3.001966	1.837556	H	6.722896	2.857888	-2.077354	H	6.920335	2.493713	-2.320404
C	-8.021396	-2.142169	-1.577836	C	8.036379	-1.965332	1.768321	C	7.809055	-1.766773	2.245269
H	-7.723802	-2.193697	-2.627735	H	7.730158	-1.934104	2.816524	H	7.405299	-1.595052	3.245490
H	-7.579219	-3.002679	-1.075398	H	7.610140	-2.870685	1.335638	H	7.413766	-2.722339	1.898915
H	-9.102413	-2.230020	-1.531001	H	9.118739	-2.042025	1.736997	H	8.888422	-1.847462	2.327498
O	-9.792148	-0.081886	-0.956152	O	9.789035	0.049968	0.969354	O	9.668815	0.082446	1.296184
H	-10.322841	0.650224	-0.639066	H	10.310519	0.765407	0.602900	H	10.232752	0.734006	0.877053
H	-5.382316	-2.097910	-2.000896	H	5.397592	-1.898287	2.195421	H	5.153649	-1.596340	2.466151
H	-3.049082	-1.984442	-0.916883	H	3.060507	-1.902022	1.110097	H	2.896111	-1.709801	1.232111
C	-3.888467	-1.088333	1.611293	C	3.896554	-1.238278	-1.491933	C	3.911179	-1.426822	-1.374025
H	-3.319506	-2.017010	1.641281	H	3.335931	-2.170970	-1.436801	H	3.318760	-2.330213	-1.230871
H	-3.622246	-0.488225	2.481152	H	3.626305	-0.723003	-2.413304	H	3.718163	-1.036816	-2.373247
H	-4.948688	-1.331106	1.674789	H	4.958925	-1.477011	-1.532432	H	4.965695	-1.693542	-1.309547
H	1.540134	-1.331927	-0.319353	H	-1.531422	-1.382562	0.415485	H	-1.571667	-1.300869	0.170073
C	1.518876	-1.772657	1.775207	C	-1.495143	-1.967130	-1.643148	C	-1.528707	-1.961642	-1.864762
H	2.421882	-2.379511	1.783256	H	-2.388929	-2.586605	-1.610350	H	-2.419204	-2.583239	-1.794844
H	1.464909	-1.243538	2.730875	H	-1.445489	-1.504533	-2.633209	H	-1.495352	-1.537798	-2.872438
H	0.664812	-2.448316	1.713446	H	-0.631999	-2.624982	-1.533485	H	-0.662048	-2.611936	-1.742645
H	13.437042	1.450428	-1.733190	H	-13.332997	1.725309	1.803231	H	-13.030450	1.796860	2.310629
C	0.300472	0.127368	0.631913	C	-0.304582	0.021052	-0.633223	C	-0.303817	0.037209	-0.913473
H	0.317180	0.744870	1.537230	H	-0.317181	0.558969	-1.588036	H	-0.265127	0.502211	-1.905095
H	0.364929	0.820881	-0.212071	H	-0.388856	0.782857	0.147631	H	-0.402670	0.856060	-0.194827
**α-Tocopherol (C18$^{\bullet}$)**				**α-Tocopherol (C19$^{\bullet}$)**				**α-Tocopherol (C20$^{\bullet}$)**			
C	3.570183	-0.388883	-0.285813	C	3.567053	-0.386555	-0.287448	C	3.561146	-0.337619	-0.352411
C	5.780201	0.528605	-0.014003	C	5.776569	0.531696	-0.017123	C	5.776238	0.533421	0.012557
C	6.145738	-0.489347	0.864959	C	6.145582	-0.490713	0.855048	C	6.122844	-0.555958	0.810399
C	5.104280	-1.440173	1.400806	C	5.106771	-1.445549	1.388903	C	5.067667	-1.536674	1.257053
C	3.692690	-0.932051	1.134658	C	3.693836	-0.937561	1.129707	C	3.664388	-0.993077	1.021648
C	6.723285	1.439666	-0.505776	C	6.717062	1.446259	-0.507019	C	6.732528	1.471855	-0.392750
C	7.483573	-0.627493	1.247577	C	7.484653	-0.629871	1.232773	C	7.454436	-0.736652	1.196382
H	5.241507	-2.426308	0.945670	H	5.243631	-2.429143	0.928217	H	5.198024	-2.483719	0.723960
H	3.453962	-0.117301	1.821677	H	3.456303	-0.126882	1.821954	H	3.428852	-0.232748	1.769683
C	8.414814	0.278969	0.760869	C	8.413556	0.280063	0.747998	C	8.398800	0.197620	0.796653
C	8.052192	1.314180	-0.103177	C	8.047392	1.319511	-0.109499	C	8.055857	1.301095	0.012081
O	4.485877	0.712265	-0.425949	O	4.480247	0.716388	-0.423894	O	4.487169	0.761606	-0.397471
C	2.202137	0.239864	-0.537789	C	2.197654	0.242255	-0.532488	C	2.199490	0.319321	-0.566868
H	2.253471	0.759311	-1.498702	H	2.245759	0.766322	-1.491017	H	2.256313	0.899938	-1.491703
H	2.042362	1.007636	0.223471	H	2.039339	1.006200	0.232876	H	2.042688	1.036656	0.242626
C	1.023463	-0.729408	-0.549780	C	1.020183	-0.728360	-0.545680	C	1.016437	-0.641936	-0.642774
H	1.181614	-1.487282	-1.318687	H	1.177740	-1.482885	-1.317959	H	1.172673	-1.348478	-1.459392
H	0.957327	-1.258403	0.405588	H	0.957068	-1.261243	0.407711	H	0.951314	-1.232329	0.275745
C	-1.547823	-0.887587	-0.692479	C	-1.550832	-0.888749	-0.680236	C	-1.550286	-0.798967	-0.766942
C	-2.808478	-0.028338	-0.837093	C	-2.812076	-0.029084	-0.815803	C	-2.819399	0.057194	-0.829260
H	-2.820893	0.415155	-1.839933	H	-2.829317	0.418609	-1.816442	H	-2.862385	0.557468	-1.803736
H	-2.738652	0.808963	-0.135692	H	-2.740326	0.804872	-0.110983	H	-2.738947	0.852193	-0.081707
C	-4.126637	-0.759333	-0.591946	C	-4.128028	-0.763222	-0.569254	C	-4.121311	-0.705022	-0.594882
H	-4.073777	-1.284791	0.368261	H	-4.073165	-1.287696	0.391513	H	-4.045250	-1.266949	0.342929
H	-4.271052	-1.524626	-1.355879	H	-4.270020	-1.528881	-1.332969	H	-4.262317	-1.441169	-1.386809
C	-5.330779	0.180831	-0.584235	C	-5.332305	0.176233	-0.560433	C	-5.340392	0.212421	-0.531600
H	-5.144704	0.983261	0.138138	H	-5.142934	0.984903	0.152396	H	-5.146530	1.008052	0.194283
H	-5.413422	0.665306	-1.564149	H	-5.425913	0.652142	-1.543596	H	-5.470395	0.707272	-1.501172
C	-6.668643	-0.480178	-0.236322	C	-6.669978	-0.478676	-0.201184	C	-6.653875	-0.479403	-0.151788
H	-6.566166	-0.986779	0.729732	H	-6.548763	-0.973778	0.770221	H	-6.507691	-0.960453	0.823098
C	-7.777869	0.568803	-0.065933	C	-7.758550	0.590833	-0.052788	C	-7.773045	0.556891	0.002979
H	-7.437269	1.276106	0.702775	H	-7.430019	1.315592	0.700175	H	-7.420574	1.345682	0.676522

H	-7.783394	1.165770	-1.016391	H	-7.846088	1.149921	-0.990656	H	-7.942969	1.038883	-0.966841
C	-9.132210	0.024535	0.330343	C	-9.145765	0.085451	0.345069	C	-9.095680	0.015631	0.542904
H	-9.569208	-0.498945	-0.529532	H	-9.000604	-0.696037	1.141128	H	-8.920758	-0.520162	1.481708
C	-10.060079	1.148438	0.735063	H	-9.566617	-0.465714	-0.503632	H	-9.512149	-0.708676	-0.160119
H	-9.550246	1.791421	1.465753	C	-10.071988	1.202148	0.761916	C	-10.137121	1.107610	0.795769
H	-10.336278	1.839795	-0.105593	H	-9.591265	1.774969	1.568122	H	-9.678217	1.884504	1.419884
C	-11.387330	0.707354	1.365915	C	-11.406296	0.703213	1.250384	H	-10.335773	1.603450	-0.200429
H	-11.132108	0.118668	2.251995	H	-11.300552	-0.100398	2.026300	C	-11.431475	0.656340	1.410702
C	-12.237296	1.905227	1.791154	C	-12.211408	1.828760	1.900852	C	-12.292064	1.866584	1.655939
H	-11.682746	2.555304	2.471773	H	-11.652183	2.276332	2.725797	H	-11.811572	2.594819	2.316749
H	-12.519485	2.502236	0.919555	H	-12.394942	2.612516	1.161680	H	-12.521775	2.412301	0.695897
C	-7.078196	-1.523766	-1.272023	C	-7.071290	-1.541469	-1.223441	C	-7.029782	-1.561891	-1.163568
H	-7.241674	-1.042786	-2.242786	H	-7.225602	-1.078302	-2.202585	H	-7.172276	-1.115728	-2.152070
H	-6.329626	-2.309017	-1.401361	H	-6.316765	-2.322866	-1.330841	H	-6.262599	-2.332762	-1.246575
H	-8.012511	-1.987071	-0.957057	H	-8.006135	-2.016194	-0.930261	H	-7.960747	-2.050050	-0.882913
C	-12.171748	-0.191413	0.415126	C	-12.254618	0.077166	0.135436	C	-12.160023	-0.267790	0.471232
H	-12.362579	0.336502	-0.525365	H	-12.431014	0.825532	-0.642085	H	-12.321249	0.217494	-0.535513
H	-11.595369	-1.088639	0.192353	H	-11.742738	-0.768880	-0.326191	H	-11.633914	-1.206641	0.280172
H	-13.135714	-0.486474	0.840078	H	-13.217797	-0.282106	0.516906	H	-13.152511	-0.529568	0.849380
C	9.122469	2.266386	-0.573630	C	9.115619	2.274597	-0.578640	C	9.141118	2.273527	-0.375380
H	9.898217	1.747224	-1.144563	H	9.891081	1.757988	-1.152197	H	9.931690	1.781123	-0.949498
H	8.720224	3.043491	-1.214861	H	8.711553	3.053089	-1.217000	H	8.760380	3.083868	-0.987897
H	9.607580	2.764993	0.270260	H	9.601348	2.771318	0.265998	H	9.602665	2.725875	0.507008
C	6.288869	2.533934	-1.447959	C	6.278946	2.545473	-1.441696	C	6.320358	2.643005	-1.248674
H	5.235102	2.435305	-1.683773	H	5.223827	2.449512	-1.672439	H	5.262063	2.593002	-1.479019
H	6.445747	3.519359	-1.005859	H	6.439674	3.528548	-0.995886	H	6.512226	3.589044	-0.740092
H	6.853332	2.498421	-2.380602	H	6.838373	2.513488	-2.377486	H	6.874311	2.657117	-2.188307
C	7.903984	-1.732667	2.178912	C	7.908929	-1.739669	2.156811	C	7.854572	-1.916651	2.040959
H	7.528170	-1.558363	3.189496	H	7.535959	-1.571253	3.169442	H	7.479989	-1.813171	3.061692
H	7.505992	-2.692965	1.849173	H	7.511108	-2.698635	1.822988	H	7.441816	-2.842341	1.638961
H	8.985796	-1.805078	2.230186	H	8.990937	-1.811213	2.204534	H	8.934908	-2.010891	2.087601
O	9.720731	0.126529	1.168847	O	9.720566	0.126287	1.150939	O	9.697304	0.001151	1.207576
H	10.267647	0.783166	0.735341	H	10.265449	0.787933	0.722446	H	10.251935	0.696572	0.851199
H	5.253013	-1.582774	2.472060	H	5.258806	-1.593647	2.458923	H	5.205091	-1.767846	2.314227
H	2.964819	-1.725998	1.295648	H	2.967506	-1.733441	1.288495	H	2.928246	-1.790268	1.114258
C	3.911050	-1.444111	-1.335786	C	3.906190	-1.435715	-1.344149	C	3.903129	-1.310549	-1.479270
H	3.334302	-2.354137	-1.171895	H	3.331636	-2.347758	-1.183564	H	3.314038	-2.223668	-1.396585
H	3.686135	-1.062074	-2.331430	H	3.678179	-1.048654	-2.337157	H	3.697186	-0.846960	-2.443894
H	4.970129	-1.697659	-1.298916	H	4.965733	-1.687755	-1.311516	H	4.958472	-1.579969	-1.449117
H	-1.559284	-1.326283	0.312715	H	-1.556975	-1.331336	0.323295	H	-1.537367	-1.302675	0.207231
C	-1.523260	-2.028729	-1.708714	C	-1.529945	-2.025551	-1.701407	C	-1.540086	-1.870592	-1.857027
H	-2.413793	-2.648937	-1.627200	H	-2.415221	-2.652733	-1.614758	H	-2.413081	-2.517030	-1.788714
H	-1.487328	-1.625777	-2.724750	H	-1.505294	-1.618590	-2.716068	H	-1.547288	-1.400796	-2.844505
H	-0.657868	-2.677189	-1.571801	H	-0.659592	-2.669109	-1.574933	H	-0.657996	-2.507153	-1.793490
H	-13.157073	1.590961	2.292535	H	-13.172234	1.472731	2.288159	H	-13.258667	1.608659	2.098948
C	-0.298919	-0.008005	-0.804125	C	-0.303430	-0.007252	-0.793955	C	-0.307305	0.092910	-0.844676
H	-0.274553	0.447193	-1.800786	H	-0.282960	0.450659	-1.789360	H	-0.295094	0.603396	-1.814156
H	-0.390496	0.817872	-0.092649	H	-0.393376	0.816568	-0.079983	H	-0.393549	0.876906	-0.086670

**α-Tocopherol (C21$^{\bullet}$)**				**α-Tocopherol (C22$^{\bullet}$)**			
C	3.557911	-0.334227	-0.344032	C	-3.565589	-0.334518	0.304186
C	5.773240	0.534575	0.023043	C	-5.776477	0.550924	-0.046857
C	6.130025	-0.573601	0.790035	C	-6.182851	-0.604535	-0.712115
C	5.081680	-1.568430	1.221340	C	-5.169375	-1.649584	-1.107376
C	3.675030	-1.022544	1.012802	C	-3.745363	-1.120011	-0.991385
C	6.722776	1.486388	-0.366244	C	-6.692175	1.549876	0.303534
C	7.465543	-0.759763	1.159413	C	-7.536099	-0.792740	-1.009644
H	5.208642	-2.501086	0.662699	H	-5.290921	-2.535339	-0.475748
H	3.445282	-0.280916	1.781107	H	-3.528728	-0.443777	-1.821283
C	8.403454	0.187352	0.775117	C	-8.441511	0.198235	-0.658821
C	8.050308	1.309301	0.021790	C	-8.037860	1.369398	-0.014403
O	4.479785	0.768638	-0.370196	O	-4.465228	0.786824	0.278074
C	2.192542	0.323466	-0.530811	C	-2.183689	0.308232	0.394346
H	2.241396	0.930604	-1.438910	H	-2.189491	0.975614	1.260471
H	2.037916	1.016697	0.299847	H	-2.049949	0.941186	-0.486636
C	1.012187	-0.639283	-0.627849	C	-1.013574	-0.664422	0.514430
H	1.167656	-1.323358	-1.463580	H	-1.165663	-1.314907	1.377317
H	0.951757	-1.254063	0.274944	H	-0.969427	-1.313017	-0.365385
C	-1.555649	-0.796025	-0.747805	C	1.558770	-0.828857	0.613552
C	-2.823950	0.061019	-0.814014	C	2.825432	0.029521	0.694119
H	-2.864840	0.558864	-1.789751	H	2.852857	0.526590	1.670817
H	-2.744238	0.857895	-0.068439	H	2.752121	0.827517	-0.051198
C	-4.127393	-0.698655	-0.579779	C	4.135330	-0.723464	0.475756
H	-4.057249	-1.251046	0.364028	H	4.083602	-1.270408	-0.472360
H	-4.264623	-1.442433	-1.365176	H	4.265236	-1.471086	1.258692
C	-5.344752	0.221927	-0.533362	C	5.347350	0.205203	0.455938

H	-5.153332	1.024530	0.185276	H	5.164106	1.008313	-0.264726
H	-5.467386	0.707218	-1.508601	H	5.445988	0.688670	1.434886
C	-6.662664	-0.462140	-0.155057	C	6.678828	-0.466983	0.105238
H	-6.524815	-0.932143	0.826528	H	6.567645	-0.932821	-0.881852
C	-7.777284	0.581298	-0.019923	C	7.788128	0.585674	0.002512
H	-7.430307	1.373456	0.651362	H	7.449852	1.379767	-0.672479
H	-7.932981	1.057897	-0.995062	H	7.917862	1.055123	0.985205
C	-9.112958	0.054585	0.501723	C	9.139351	0.065171	-0.480120
H	-8.942329	-0.492183	1.436448	H	9.005170	-0.475838	-1.425658
H	-9.526244	-0.666264	-0.207042	H	9.558125	-0.638920	0.239660
C	-10.132985	1.168665	0.735985	C	10.167937	1.171452	-0.675831
H	-9.690741	1.923828	1.395472	H	9.768649	1.917011	-1.380226
H	-10.351627	1.677963	-0.208562	H	10.338668	1.667666	0.286815
C	-11.472021	0.735721	1.348072	C	11.522567	0.649782	-1.176165
H	-11.201473	0.168256	2.269971	H	11.318509	0.276585	-2.209297
C	-12.358742	1.941843	1.576239	C	12.464927	1.843082	-1.345718
H	-11.848707	2.701930	2.182908	H	12.047516	2.606077	-2.012826
C	-7.036262	-1.554757	-1.156501	H	12.655942	2.289133	-0.365946
H	-7.174241	-1.119205	-2.150261	C	7.040129	-1.560831	1.109647
H	-6.268823	-2.326048	-1.228347	H	7.143446	-1.130432	2.109610
H	-7.967899	-2.041412	-0.874836	H	6.282945	-2.344669	1.152811
C	-12.176853	-0.276061	0.435186	H	7.988804	-2.027812	0.853535
H	-12.308612	0.164629	-0.556818	C	12.122429	-0.370269	-0.235097
H	-11.637137	-1.226403	0.344016	H	11.567931	-1.314901	-0.226166
H	-13.172220	-0.490283	0.829733	H	13.163789	-0.586508	-0.501683
C	9.129468	2.293970	-0.351435	C	-9.083034	2.402105	0.324191
H	9.918894	1.816540	-0.939582	H	-9.825808	2.005500	1.022973
H	8.742331	3.114753	-0.945765	H	-8.648434	3.281546	0.787346
H	9.593865	2.730388	0.537460	H	-9.613540	2.738172	-0.571163
C	6.299331	2.678412	-1.187192	C	-6.213654	2.794057	1.008312
H	5.237216	2.636662	-1.400871	H	-5.142726	2.752047	1.172153
H	6.502699	3.611588	-0.659801	H	-6.431848	3.687204	0.420864
H	6.837252	2.713338	-2.135629	H	-6.702267	2.910874	1.976864
C	7.877102	-1.959551	1.969852	C	-8.001851	-2.042615	-1.706749
H	7.517642	-1.880171	2.998221	H	-7.699298	-2.042217	-2.756498
H	7.458173	-2.875339	1.552085	H	-7.568479	-2.930922	-1.246824
H	8.957938	-2.054979	1.998616	H	-9.083524	-2.125585	-1.669807
O	9.705941	-0.015684	1.169338	O	-9.763689	-0.011531	-0.977170
H	10.255602	0.690645	0.826958	H	-10.291471	0.711335	-0.634912
H	5.229758	-1.826228	2.270894	H	-5.361802	-1.981081	-2.128743
H	2.941276	-1.823396	1.093013	H	-3.030084	-1.940319	-1.035949
C	3.892688	-1.278321	-1.497478	C	-3.875411	-1.181553	1.537361
H	3.308734	-2.196083	-1.430889	H	-3.313393	-2.114929	1.518355
H	3.674799	-0.792811	-2.448573	H	-3.610624	-0.631805	2.440316
H	4.949496	-1.543173	-1.484487	H	-4.937083	-1.421221	1.583140
H	-1.550775	-1.308284	0.221928	H	1.565478	-1.344477	-0.354337
C	-1.535033	-1.857625	-1.847529	C	1.523649	-1.886578	1.716448
H	-2.417461	-2.493473	-1.805513	H	2.413228	-2.513325	1.695853
H	-1.512700	-1.378779	-2.830407	H	1.474609	-1.405056	2.697088
H	-0.662974	-2.506206	-1.769602	H	0.659875	-2.544023	1.620879
H	-13.288641	1.656477	2.084159	H	13.424575	1.516919	-1.753336
C	-0.313247	0.097949	-0.807738	C	0.318556	0.068918	0.656591
H	-0.304417	0.630429	-1.765313	H	0.320655	0.629232	1.598241
H	-0.398277	0.864601	-0.032075	H	0.399483	0.812937	-0.141377

Structure of trolox and the corresponding radical species in the HAT mechanism optimized at the M05-2X/6-311++G(2d,2p) level of theory in gas phase.

**M05-2X/6-311++G(2d,2p)**											
**Trolox**				**Trolox (O1$^{\bullet}$)**				**Trolox (O2$^{\bullet}$)**			
C	-2.42613900	0.83636800	-0.04307100	C	-2.448411	0.868490	-0.048456	C	-2.248524	0.773246	-0.169281
C	-1.16224200	1.37329400	0.18384500	C	-1.192043	1.371640	0.145847	C	-0.988431	1.296347	0.112542
C	-0.09593100	0.49971500	0.38891600	C	-0.115760	0.474076	0.334656	C	0.041243	0.407266	0.419105
C	-0.26005000	-0.87884600	0.36339600	C	-0.268443	-0.921058	0.339790	C	-0.153439	-0.968554	0.461196
C	-1.52236200	-1.41507900	0.10377100	C	-1.517767	-1.449321	0.124436	C	-1.412550	-1.488378	0.164465
C	-2.58522800	-0.54770200	-0.08867400	C	-2.660796	-0.573813	-0.071457	C	-2.439506	-0.607009	-0.141300
O	-3.82169800	-1.10328400	-0.32230400	O	-3.796497	-1.051101	-0.250377	O	-3.672439	-1.147337	-0.416276
H	-4.44632200	-0.41093100	-0.53821800	O	1.085330	1.072915	0.537177	H	-4.284568	-0.446952	-0.642024
O	1.13172600	1.08491700	0.64430500	C	-0.882185	2.841578	0.145840	O	1.284113	0.973888	0.638689
C	-0.93286300	2.86166000	0.20063700	H	-1.767571	3.426950	-0.063476	C	-0.734928	2.781342	0.089475
H	-1.31077500	3.31956100	-0.70971200	H	-0.126897	3.061457	-0.604250	H	-0.923703	3.190644	-0.899928
H	0.12327100	3.07967700	0.27578900	H	-0.477506	3.150213	1.106824	H	0.291585	2.998020	0.349688
H	-1.43884800	3.32727500	1.04438900	C	-3.646737	1.744323	-0.260194	H	-1.383333	3.293518	0.796588
C	-3.63715400	1.70776700	-0.24902300	H	-4.532034	1.126914	-0.339374	C	-3.420759	1.655569	-0.509955
H	-4.42603800	1.45358600	0.45912100	H	-3.771524	2.441296	0.565055	H	-4.233601	1.524764	0.205237
H	-3.40390500	2.75417500	-0.10659500	H	-3.544847	2.327591	-1.173241	H	-3.148829	2.702248	-0.501226
H	-4.03982700	1.59937500	-1.25729000	C	-1.749905	-2.926868	0.097772	H	-3.807095	1.432277	-1.505247
C	-1.72782300	-2.90331300	0.04055500	H	-1.020414	-3.425773	-0.536058	C	-1.656685	-2.972696	0.167405
H	-0.96702400	-3.37321600	-0.57757000	H	-1.660224	-3.351299	1.097760	H	-0.854948	-3.495802	-0.346326
H	-1.66597000	-3.34940600	1.03235400	H	-2.748159	-3.129645	-0.267899	H	-1.705106	-3.358731	1.184709
H	-2.70100600	-3.13584500	-0.37143800	C	3.390246	1.125690	1.000152	H	-2.593038	-3.204976	-0.322889
C	3.45351100	1.07583500	0.99056000	H	4.331072	0.593040	0.916100	C	3.539330	0.979972	1.364325
H	4.37648700	0.52488500	0.84381500	H	3.457734	2.068456	0.465847	H	4.417947	0.343953	1.412772
H	3.51718100	2.03461400	0.48483100	H	3.179319	1.328181	2.045660	H	3.712213	1.762971	0.630645
H	3.29649800	1.24700700	2.05112800	C	0.923806	-1.812051	0.581601	H	3.415989	1.461712	2.340280
C	0.91168600	-1.79913200	0.59976800	H	0.642355	-2.627639	1.241337	C	0.991923	-1.872966	0.828631
H	0.61946900	-2.60144400	1.27215300	H	1.236639	-2.269061	-0.358768	H	0.621978	-2.728737	1.385658
H	1.19805200	-2.27531800	-0.33938000	C	2.079001	-1.026872	1.185308	H	1.468460	-2.251095	-0.075712
C	2.10129800	-1.04636700	1.17604500	H	1.864011	-0.766219	2.219811	C	2.013307	-1.105699	1.661825
H	1.92609300	-0.80177500	2.22173400	H	3.002093	-1.595894	1.157597	H	1.591716	-0.916309	2.657527
H	3.01112800	-1.63289700	1.10571900	C	2.261464	0.282276	0.432420	H	2.921694	-1.684886	1.799370
C	2.27450800	0.28046200	0.45085800	C	2.520808	0.020968	-1.047208	C	2.345764	0.178962	1.000032
C	2.48722300	0.07446800	-1.04593600	O	1.872240	0.446283	-1.956028	C	1.409408	0.723774	-2.345651
O	1.91116100	0.64649700	-1.92312000	O	3.606399	-0.759821	-1.219414	O	1.083516	1.826205	-2.459915
O	3.46511300	-0.82801400	-1.28376900	H	3.725234	-0.871441	-2.170612	O	1.744500	-0.377986	-2.256848
H	3.57051000	-0.87504000	-2.24162600								
**Trolox (C1$^{\bullet}$)**				**Trolox (C2$^{\bullet}$)**				**Trolox (C3$^{\bullet}$)**			
C	-2.479023	-0.863856	0.024264	C	-2.478043	0.792304	-0.040108	C	-2.412074	0.803916	-0.009092
C	-1.181884	-1.422506	-0.154393	C	-1.180735	1.344860	0.150451	C	-1.144879	1.350374	0.207592
C	-0.098456	-0.524893	-0.358643	C	-0.118004	0.491494	0.364063	C	-0.063158	0.479065	0.418907
C	-0.271411	0.843091	-0.367140	C	-0.270490	-0.898830	0.380971	C	-0.200791	-0.890132	0.407947
C	-1.542555	1.378256	-0.121084	C	-1.528389	-1.460704	0.127537	C	-1.478531	-1.461488	0.139746
C	-2.620252	0.509425	0.073211	C	-2.603689	-0.624709	-0.083359	C	-2.562849	-0.571310	-0.053116
O	-3.843580	1.101155	0.298006	O	-3.820927	-1.199487	-0.328046	O	-3.780351	-1.143386	-0.289519
H	-4.437650	0.461437	0.688942	H	-4.383921	-0.565708	-0.774307	H	-4.424969	-0.460818	-0.478757
O	1.120340	-1.120755	-0.566514	O	1.107930	1.085150	0.599653	O	1.153259	1.090778	0.657643
C	-0.947312	-2.794713	-0.118964	C	-0.985515	2.831855	0.137241	C	-0.924119	2.836937	0.205287
H	-1.741670	-3.493752	0.060694	H	-1.377743	3.258129	-0.783525	H	-1.336722	3.285406	-0.694869
H	0.048809	-3.174434	-0.237567	H	0.064389	3.078527	0.210027	H	0.132333	3.062389	0.243416
C	-3.665665	-1.775535	0.168670	H	-1.510279	3.299758	0.968863	H	-1.405494	3.307091	1.061720
H	-4.599268	-1.271795	-0.065126	C	-3.597384	1.615157	-0.170550	C	-3.628777	1.669333	-0.211236
H	-3.588170	-2.606745	-0.525162	H	-4.600253	1.227591	-0.201637	H	-4.421018	1.397810	0.486571
H	-3.739323	-2.190969	1.173211	H	-3.499910	2.683775	-0.175836	H	-3.405374	2.714613	-0.046440
C	-1.747043	2.864808	-0.078499	C	-1.701685	-2.954003	0.084297	H	-4.021701	1.576132	-1.224490
H	-0.963337	3.347777	0.499617	H	-0.931702	-3.415956	-0.528201	C	-1.683356	-2.836069	0.058501
H	-1.722957	3.291245	-1.081369	H	-1.630160	-3.384422	1.082043	H	-0.875505	-3.531659	0.185545
H	-2.704777	3.105580	0.363103	H	-2.670217	-3.211499	-0.323314	H	-2.662539	-3.223501	-0.148156
C	3.433322	-1.125307	-0.971796	C	3.430229	1.105783	0.937702	C	3.484468	1.128066	0.943139

H	4.359618	-0.570351	-0.869334	H	4.357523	0.558445	0.805859	H	4.413194	0.592655	0.775888
H	3.509680	-2.063847	-0.431094	H	3.485221	2.049664	0.403817	H	3.516702	2.083039	0.427356
H	3.250074	-1.337802	-2.020592	H	3.273237	1.307259	1.992907	H	3.353767	1.305541	2.006235
C	0.897166	1.760592	-0.627613	C	0.910354	-1.797723	0.636992	C	0.977704	-1.792470	0.657310
H	0.599022	2.549465	-1.312890	H	0.627899	-2.588105	1.327721	H	0.701237	-2.562922	1.372959
H	1.195065	2.253633	0.299130	H	1.197929	-2.294228	-0.292079	H	1.244676	-2.308630	-0.266225
C	2.076098	0.991185	-1.204270	C	2.096429	-1.021114	1.188564	C	2.174433	-1.014641	1.184542
H	1.878105	0.711375	-2.237051	H	1.923472	-0.748556	2.227608	H	2.028726	-0.760325	2.232308
H	2.986495	1.580162	-1.173418	H	3.009253	-1.604387	1.131052	H	3.089465	-1.589890	1.093310
C	2.269130	-0.308036	-0.434740	C	2.257303	0.284925	0.424296	C	2.306996	0.306033	0.441959
C	2.508069	-0.033014	1.046632	C	2.464387	0.032386	-1.065746	C	2.484084	0.096420	-1.059194
O	1.907062	-0.516081	1.958954	O	1.860199	0.550984	-1.956961	O	1.897644	0.679168	-1.922461
O	3.533497	0.829963	1.222811	O	3.466608	-0.848844	-1.279505	O	3.446582	-0.815498	-1.318498
H	3.648161	0.932817	2.175209	H	3.564295	-0.930414	-2.235933	H	3.531654	-0.862649	-2.278429
**Trolox (C4$^{\bullet}$)**				**Trolox (C5$^{\bullet}$)**				**Trolox (C6$^{\bullet}$)**			
C	-2.396567	0.830421	-0.077601	C	-2.402597	0.823277	-0.134546	C	-2.454712	0.835970	-0.073551
C	-1.143330	1.376175	0.216255	C	-1.157148	1.356208	0.188226	C	-1.198829	1.385457	0.166871
C	-0.104666	0.513534	0.510371	C	-0.132146	0.477606	0.527669	C	-0.130079	0.522404	0.400848
C	-0.261675	-0.893720	0.513403	C	-0.309892	-0.897969	0.526641	C	-0.281727	-0.857543	0.385042
C	-1.533638	-1.434164	0.185487	C	-1.551621	-1.433907	0.185068	C	-1.535150	-1.406564	0.109410
C	-2.561641	-0.563443	-0.093943	C	-2.577961	-0.560057	-0.138426	C	-2.601829	-0.549813	-0.107793
O	-3.789210	-1.104979	-0.396253	O	-3.793145	-1.108973	-0.472614	O	-3.829703	-1.117826	-0.355121
H	-4.398768	-0.404718	-0.628358	H	-4.406651	-0.409012	-0.695841	H	-4.455588	-0.433592	-0.592183
O	1.113519	1.063463	0.843222	O	1.080473	1.031329	0.900573	O	1.087117	1.122027	0.675610
C	-0.908535	2.862679	0.199341	C	-0.900288	2.838496	0.158455	C	-0.980204	2.875451	0.166835
H	-1.194526	3.284890	-0.760492	H	-1.181520	3.255656	-0.804778	H	-1.369780	3.320936	-0.744476
H	0.138004	3.077928	0.364608	H	0.149938	3.038654	0.318540	H	0.075127	3.101611	0.229104
H	-1.488663	3.361739	0.973003	H	-1.471403	3.353073	0.928876	H	-1.481063	3.346342	1.010748
C	-3.583410	1.698028	-0.393319	C	-3.574630	1.697963	-0.494840	C	-3.669943	1.695126	-0.304581
H	-4.413508	1.490134	0.283157	H	-4.424751	1.503174	0.160106	H	-4.469702	1.432968	0.388173
H	-3.345988	2.748530	-0.295268	H	-3.332986	2.747784	-0.398604	H	-3.450448	2.743869	-0.157336
H	-3.933088	1.538726	-1.414835	H	-3.894706	1.530331	-1.524078	H	-4.051682	1.582432	-1.320472
C	-1.741742	-2.919338	0.154940	C	-1.774557	-2.920722	0.174111	C	-1.727367	-2.896893	0.056301
H	-1.046453	-3.390456	-0.537430	H	-1.237362	-3.388906	-0.649462	H	-0.954195	-3.365699	-0.547118
H	-1.572727	-3.355504	1.138200	H	-1.418458	-3.373475	1.096374	H	-1.675747	-3.333936	1.052698
H	-2.750660	-3.156664	-0.154739	H	-2.826223	-3.148869	0.061955	H	-2.692706	-3.141074	-0.367160
C	3.460166	1.064595	1.014936	C	3.432602	1.011677	1.094154	C	3.394066	1.104914	1.010162
H	4.371951	0.514988	0.803464	H	4.345248	0.503358	0.800477	H	3.273726	2.157733	1.185916
H	3.498410	2.034176	0.527835	H	3.455194	2.038049	0.741285	H	4.356289	0.633098	1.084032
H	3.365390	1.211331	2.086452	H	3.344122	1.005617	2.175698	C	0.893068	-1.767055	0.646078
C	0.829204	-1.701748	0.849346	C	0.827393	-1.813629	0.902419	H	0.596655	-2.566552	1.319954
H	0.717404	-2.768966	0.907122	H	0.606022	-2.304308	1.854354	H	1.198253	-2.247128	-0.284949
C	2.143283	-1.085229	1.183546	H	0.890232	-2.628524	0.173874	C	2.067462	-1.004241	1.239612
H	2.242861	-0.931673	2.261656	C	2.128310	-1.110236	0.987619	H	1.877307	-0.753884	2.280381
H	2.969560	-1.714859	0.867947	H	3.025720	-1.637722	1.258309	H	2.985068	-1.579133	1.182042
C	2.252240	0.283094	0.524286	C	2.224035	0.291526	0.509550	C	2.237025	0.326517	0.506964
C	2.352816	0.172917	-0.995327	C	2.339391	0.375619	-1.020393	C	2.469514	0.102960	-0.996163
O	1.755679	0.849182	-1.779879	O	1.743436	1.136859	-1.723283	O	1.906339	0.672539	-1.881945
O	3.264442	-0.747841	-1.370965	O	3.255138	-0.494917	-1.492633	O	3.446109	-0.805742	-1.208485
H	3.299525	-0.724381	-2.334826	H	3.313374	-0.348602	-2.444591	H	3.574552	-0.857034	-2.163300

Structures of trolox in the SET-PT (SET_step) mechanism at M05-2X/6-311++G(2d,2p) level of theory in gas phase.

**M05-2X/6-311++G(2d,2p)**			
Trolox $^{\bullet+}$			
C	-2.43511800	-0.86801600	0.06278800
C	-1.19849700	-1.40426300	-0.16016400
C	-0.09629900	-0.50224900	-0.33905300
C	-0.24545400	0.91202700	-0.35494800
C	-1.48335900	1.44398700	-0.12800700
C	-2.56892800	0.54634000	0.07780700
O	-3.75009600	1.10413900	0.27793500
H	-4.44194200	0.44534300	0.40767900
O	1.07210500	-1.08116600	-0.51272700
C	-0.95903900	-2.88452600	-0.18940600
H	-1.13422200	-3.31115800	0.79575900
H	0.05487700	-3.11435800	-0.48187600
H	-1.63823800	-3.36295800	-0.88890600
C	-3.66253600	-1.69765200	0.30550100
H	-4.40515000	-1.53954000	-0.47681000

H	-3.43009600	-2.75302700	0.32280300
H	-4.11321700	-1.45393700	1.26765400
C	-1.72305100	2.92242100	-0.10664700
H	-0.90042800	3.43433000	0.38126500
H	-1.80136000	3.30589000	-1.12319300
H	-2.64138500	3.16328900	0.41161100
C	3.38535900	-1.18702500	-0.99796800
H	4.33357100	-0.66483100	-0.94348200
H	3.45698400	-2.11204400	-0.43466100
H	3.16055100	-1.41673800	-2.03472800
C	0.94806100	1.78794000	-0.62103400
H	0.66509100	2.58833800	-1.29774700
H	1.25085500	2.26071300	0.31426200
C	2.10585000	0.99053000	-1.20717100
H	1.91065700	0.72645400	-2.24427600
H	3.02811200	1.56029500	-1.17040200
C	2.29099900	-0.30515600	-0.43640800
C	2.50301300	-0.00104400	1.04471900
O	1.67653700	-0.16975900	1.89364700
O	3.71080800	0.52292300	1.25347200
H	3.80451700	0.71496000	2.19638500

Structures of trolox in the SET-PT (PT_step) mechanism at M05-2X/6-311++G(2d,2p) level of theory in gas phase.

**M05-2X/6-311++G(2d,2p)**											
Trolox •+				**Trolox (O1•)**				**Trolox (O2•)**			
C	-2.43511800	-0.86801600	0.06278800	C	-2.441338	-0.861699	0.067476	C	-2.340561	-0.819300	0.174824
C	-1.19849700	-1.40426300	-0.16016400	C	-1.200427	-1.394693	-0.148496	C	-1.084453	-1.340049	0.201501
C	-0.09629900	-0.50224900	-0.33905300	C	-0.110235	-0.509684	-0.339433	C	0.085482	-0.449945	0.026593
C	-0.24545400	0.91202700	-0.35494800	C	-0.250022	0.890248	-0.348543	C	-0.192609	1.001802	-0.156725
C	-1.48335900	1.44398700	-0.12800700	C	-1.487923	1.439677	-0.125410	C	-1.459711	1.484769	-0.170209
C	-2.56892800	0.54634000	0.07780700	C	-2.636515	0.579047	0.088780	C	-2.532487	0.572961	-0.011123
O	-3.75009600	1.10413900	0.27793500	O	-3.769828	1.055782	0.279842	O	-3.789008	1.092516	-0.045300
H	-4.44194200	0.44534300	0.40767900	O	1.092236	-1.106875	-0.543222	H	-4.434894	0.394062	0.072928
O	1.07210500	-1.08116600	-0.51272700	C	-0.967219	-2.883810	-0.169181	O	0.915969	-0.920519	-1.032483
C	-0.95903900	-2.88452600	-0.18940600	H	-1.182666	-3.314965	0.805288	C	-0.783620	-2.797269	0.398910
H	-1.13422200	-3.31115800	0.79575900	H	0.057939	-3.115469	-0.421553	H	-1.165432	-3.152688	1.353504
H	0.05487700	-3.11435800	-0.48187600	H	-1.618637	-3.361049	-0.895939	H	0.286517	-2.963192	0.384370
H	-1.63823800	-3.36295800	-0.88890600	C	-3.666701	-1.686213	0.300444	H	-1.225019	-3.403147	-0.389506
C	-3.66253600	-1.69765200	0.30550100	H	-4.380216	-1.524222	-0.504916	C	-3.561626	-1.685430	0.338606
H	-4.40515000	-1.53954000	-0.47681000	H	-3.443160	-2.742721	0.371272	H	-4.205076	-1.629684	-0.539751
H	-3.43009600	-2.75302700	0.32280300	H	-4.159605	-1.358293	1.211387	H	-3.290632	-2.723823	0.473604
H	-4.11321700	-1.45393700	1.26765400	C	-1.703112	2.919652	-0.104773	H	-4.143706	-1.385935	1.210430
C	-1.72305100	2.92242100	-0.10664700	H	-0.972113	3.413049	0.531739	C	-1.751904	2.952704	-0.347228
H	-0.90042800	3.43433000	0.38126500	H	-1.603658	3.340370	-1.105316	H	-1.108759	3.550499	0.291136
H	-1.80136000	3.30589000	-1.12319300	H	-2.701181	3.133270	0.255747	H	-1.582611	3.258997	-1.377390
H	-2.64138500	3.16328900	0.41161100	C	3.395491	-1.177908	-1.005148	H	-2.781697	3.171985	-0.098185
C	3.38535900	-1.18702500	-0.99796800	H	4.338907	-0.648574	-0.930306	C	3.287619	-1.114828	-1.450316
H	4.33357100	-0.66483100	-0.94348200	H	3.461194	-2.114494	-0.459740	H	4.251858	-0.768102	-1.091777
H	3.45698400	-2.11204400	-0.43466100	H	3.180326	-1.391812	-2.047544	H	3.196589	-2.176599	-1.244332
H	3.16055100	-1.41673800	-2.03472800	C	0.946740	1.772245	-0.604667	H	3.218695	-0.951673	-2.521806
C	0.94806100	1.78794000	-0.62103400	H	0.664028	2.584191	-1.268263	C	1.044013	1.848759	-0.292937
H	0.66509100	2.58833800	-1.29774700	H	1.266641	2.235209	0.330446	H	0.809688	2.797384	-0.761921
H	1.25085500	2.26071300	0.31426200	C	2.095869	0.980080	-1.208478	H	1.423825	2.073687	0.706354
C	2.10585000	0.99053000	-1.20717100	H	1.875924	0.711682	-2.239938	C	2.147817	1.131991	-1.090781
H	1.91065700	0.72645400	-2.24427600	H	3.021394	1.545454	-1.188325	H	1.942615	1.193103	-2.157129
H	3.02811200	1.56029500	-1.17040200	C	2.272394	-0.322332	-0.444199	H	3.114117	1.589002	-0.895050
C	2.29099900	-0.30515600	-0.43640800	C	2.534758	-0.046530	1.032321	C	2.196858	-0.355178	-0.745143
C	2.50301300	-0.00104400	1.04471900	O	1.879834	-0.449566	1.946461	C	2.247782	-0.499882	0.770705
O	1.67653700	-0.16975900	1.89364700	O	3.631495	0.721243	1.194264	O	0.979452	-0.594540	1.204740
O	3.71080800	0.52292300	1.25347200	H	3.750859	0.844837	2.143918	O	3.209925	-0.496928	1.478176
H	3.80451700	0.71496000	2.19638500								
**Trolox (C1•)**				**Trolox (C2•)**				**Trolox (C3•)**			
C	-2.520372	-0.788993	0.033086	C	-2.480912	-0.763780	0.105179	C	-2.423736	-0.785686	0.031289
C	-1.236487	-1.383256	-0.125287	C	-1.207641	-1.335266	-0.171975	C	-1.164877	-1.354510	-0.176401
C	-0.129682	-0.517235	-0.340335	C	-0.134638	-0.497834	-0.395185	C	-0.073179	-0.503745	-0.417709
C	-0.268376	0.854154	-0.378049	C	-0.257648	0.895293	-0.365242	C	-0.193117	0.866921	-0.444805
C	-1.527313	1.425688	-0.151762	C	-1.504787	1.474849	-0.101218	C	-1.461835	1.462220	-0.186227
C	-2.627554	0.588097	0.052984	C	-2.593268	0.654878	0.107280	C	-2.556450	0.592073	0.037494

O	-3.837120	1.214555	0.257401	O	-3.804468	1.250416	0.327987	O	-3.765007	1.186479	0.264585
H	-4.449355	0.598009	0.657420	H	-4.502076	0.614460	0.166881	H	-4.417597	0.517843	0.475071
O	1.075181	-1.147301	-0.527503	O	1.074106	-1.108461	-0.674523	O	1.133917	-1.137738	-0.645198
C	-1.036492	-2.759848	-0.060304	C	-1.038097	-2.825631	-0.182372	C	-0.963391	-2.843190	-0.133322
H	-0.049403	-3.166511	-0.164236	H	-1.305058	-3.244855	0.785660	H	-1.377923	-3.260899	0.780636
H	-1.849216	-3.435109	0.127978	H	-0.013208	-3.091651	-0.399200	H	0.089877	-3.083409	-0.169310
C	-3.730255	-1.667803	0.188065	H	-1.682736	-3.283095	-0.930790	H	-1.454487	-3.330750	-0.974378
H	-4.649562	-1.145898	-0.061938	C	-3.581815	-1.572399	0.391603	C	-3.650288	-1.629258	0.263922
H	-3.669315	-2.514559	-0.488409	H	-3.504909	-2.642028	0.355740	H	-4.443759	-1.365326	-0.435410
H	-3.820285	-2.060858	1.200242	H	-4.525581	-1.175379	0.721666	H	-3.441851	-2.681311	0.124537
C	-1.694770	2.917434	-0.141058	C	-1.657266	2.970975	-0.063238	H	-4.034853	-1.504758	1.277008
H	-0.903789	3.392412	0.433789	H	-0.902101	3.419473	0.577502	C	-1.648506	2.841117	-0.142962
H	-1.651894	3.322509	-1.152115	H	-1.542055	3.401083	-1.057024	H	-0.832518	3.522239	-0.294125
H	-2.649632	3.191040	0.287430	H	-2.634219	3.245734	0.310955	H	-2.621499	3.246873	0.057717
C	3.390008	-1.217590	-0.915882	C	3.390131	-1.166798	-1.049168	C	3.462801	-1.213403	-0.941840
H	4.329162	-0.683819	-0.818014	H	4.330943	-0.643837	-0.913996	H	4.399243	-0.685580	-0.794733
H	3.439220	-2.146412	-0.355601	H	3.432672	-2.128616	-0.547052	H	3.485723	-2.153760	-0.399388
H	3.208621	-1.446990	-1.961434	H	3.213023	-1.330128	-2.107806	H	3.323823	-1.419157	-1.998771
C	0.924371	1.736819	-0.649097	C	0.937321	1.777522	-0.613783	C	0.995480	1.746418	-0.725892
H	0.650481	2.518426	-1.352579	H	0.661555	2.594058	-1.276041	H	0.724849	2.499730	-1.461747
H	1.228345	2.241491	0.269373	H	1.248102	2.239928	0.325382	H	1.274199	2.285132	0.181142
C	2.087568	0.926664	-1.201401	C	2.099063	0.995270	-1.207880	C	2.179122	0.938464	-1.237052
H	1.889552	0.630586	-2.229631	H	1.906149	0.761993	-2.252952	H	2.024316	0.656489	-2.276398
H	3.012143	1.493398	-1.176080	H	3.024699	1.557226	-1.143973	H	3.102001	1.504055	-1.166950
C	2.242989	-0.360779	-0.404080	C	2.242960	-0.338844	-0.490769	C	2.298845	-0.362304	-0.457952
C	2.478832	-0.061388	1.073056	C	2.478603	-0.142669	1.003667	C	2.487017	-0.112386	1.035656
O	1.860162	-0.510895	1.990712	O	1.882216	-0.684800	1.886026	O	1.897591	-0.662182	1.918280
O	3.523983	0.779688	1.238951	O	3.497734	0.715449	1.231326	O	3.463043	0.793488	1.263777
H	3.634909	0.899104	2.189858	H	3.612836	0.760964	2.188203	H	3.553945	0.866994	2.221517
**Trolox (C4$^{\bullet}$)**				**Trolox (C5$^{\bullet}$)**				**Trolox (C6$^{\bullet}$)**			
C	-2.407938	-0.811019	0.104137	C	-2.412918	-0.803661	0.160393	C	-2.464859	-0.814905	0.102375
C	-1.164335	-1.380332	-0.185805	C	-1.176751	-1.360211	-0.158025	C	-1.217739	-1.386461	-0.132115
C	-0.116632	-0.538918	-0.508119	C	-0.142799	-0.503271	-0.524752	C	-0.139441	-0.543452	-0.393897
C	-0.255109	0.869829	-0.543814	C	-0.302858	0.874134	-0.555077	C	-0.273242	0.838310	-0.411394
C	-1.517373	1.434895	-0.219838	C	-1.535303	1.434063	-0.218213	C	-1.517704	1.410246	-0.141684
C	-2.554554	0.584925	0.088046	C	-2.570553	0.581547	0.132493	C	-2.593836	0.573107	0.103369
O	-3.772494	1.149874	0.386418	O	-3.776339	1.153845	0.461439	O	-3.812656	1.163039	0.344547
H	-4.389894	0.463343	0.637999	H	-4.397742	0.467180	0.703343	H	-4.446027	0.492818	0.600980
O	1.091625	-1.112860	-0.836513	O	1.060061	-1.081486	-0.892034	O	1.068062	-1.165552	-0.661001
C	-0.949057	-2.869026	-0.135063	C	-0.938901	-2.844645	-0.095026	C	-1.018357	-2.878733	-0.096680
H	-1.231524	-3.264116	0.837290	H	-1.215615	-3.234759	0.880792	H	-1.408780	-3.296701	0.827207
H	0.092925	-3.102285	-0.304597	H	0.106901	-3.062592	-0.260653	H	0.033620	-3.120109	-0.158676
H	-1.543038	-3.378687	-0.891082	H	-1.524629	-3.369937	-0.847006	H	-1.529734	-3.363509	-0.926297
C	-3.603521	-1.654887	0.449830	C	-3.593443	-1.654207	0.549580	C	-3.689521	-1.652221	0.361998
H	-4.436068	-1.452764	-0.225405	H	-4.444813	-1.466302	-0.105775	H	-4.490777	-1.395911	-0.331227
H	-3.380540	-2.710510	0.375926	H	-3.365023	-2.709064	0.479886	H	-3.484836	-2.706936	0.237911
H	-3.943169	-1.465917	1.469660	H	-3.905683	-1.455644	1.575704	H	-4.062402	-1.510624	1.377552
C	-1.705712	2.923020	-0.223540	C	-1.738871	2.923519	-0.240929	C	-1.690185	2.903827	-0.124252
H	-0.999199	3.401284	0.452378	H	-1.189654	3.403881	0.567570	H	-0.907743	3.376955	0.463649
H	-1.538252	3.333221	-1.218177	H	-1.383579	3.349679	-1.176092	H	-1.638037	3.315616	-1.131319
H	-2.709118	3.181092	0.087445	H	-2.786636	3.168060	-0.126898	H	-2.649996	3.171047	0.297847
C	3.436693	-1.149497	-1.025128	C	3.410903	-1.096836	-1.101417	C	3.372629	-1.186966	-1.010870
H	4.357286	-0.607537	-0.832967	H	4.331878	-0.593024	-0.826565	H	3.237212	-2.242298	-1.158399
H	3.465324	-2.107767	-0.515513	H	3.423077	-2.114330	-0.723233	H	4.340269	-0.729861	-1.103743
H	3.332299	-1.320083	-2.092212	H	3.315018	-1.116548	-2.182174	C	0.911301	1.725736	-0.702627
C	0.843754	1.654994	-0.906859	C	0.843363	1.765988	-0.959870	H	0.620529	2.512025	-1.394245
H	0.745624	2.721911	-0.989438	H	0.621061	2.238188	-1.920871	H	1.229168	2.224750	0.214080
C	2.146993	1.013115	-1.235754	H	0.922560	2.596080	-0.250275	C	2.071656	0.933092	-1.284687
H	2.236323	0.832524	-2.310578	C	2.134146	1.043555	-1.039039	H	1.871156	0.659652	-2.317679
H	2.983914	1.638993	-0.941436	H	3.035869	1.552149	-1.330796	H	2.996979	1.497218	-1.247460
C	2.242918	-0.340362	-0.544684	C	2.215610	-0.347034	-0.526983	C	2.229162	-0.380933	-0.520153
C	2.356509	-0.195310	0.971101	C	2.340330	-0.394838	1.003810	C	2.474898	-0.123002	0.975461
O	1.756669	-0.844872	1.775908	O	1.739842	-1.130978	1.729186	O	1.910957	-0.663428	1.878892
O	3.282654	0.722198	1.317962	O	3.269979	0.475585	1.448347	O	3.464239	0.778157	1.158656
H	3.324695	0.721290	2.281832	H	3.332933	0.352092	2.403235	H	3.599993	0.851310	2.111035

Structures of trolox and their corresponding species in the SPLET (SPL_step) mechanism at M05-2X/6-311++G(2d,2p) level of theory in gas phase.

**M05-2X/6-311++G(2d,2p)**		
**Trolox**	**Trolox (O1$^-$)**	**Trolox (O2$^-$)**
C -2.42613900 0.83636800 -0.04307100	C -2.414880 0.816675 -0.070209	C -2.440203 0.833729 -0.030077
C -1.16224200 1.37329400 0.18384500	C -1.157906 1.349380 0.175605	C -1.170253 1.361567 0.181208
C -0.09593100 0.49971500 0.38891600	C -0.087870 0.496013 0.431536	C -0.107022 0.490951 0.433462
C -0.26005000 -0.87884600 0.36339600	C -0.257966 -0.882510 0.421393	C -0.274135 -0.888612 0.408156
C -1.52236200 -1.41507900 0.10377100	C -1.510172 -1.426538 0.147535	C -1.538351 -1.418224 0.137503
C -2.58522800 -0.54770200 -0.08867400	C -2.651956 -0.600633 -0.102100	C -2.599072 -0.551782 -0.061872
O -3.82169800 -1.10328400 -0.32230400	O -3.805618 -1.087455 -0.342537	O -3.854567 -1.104563 -0.294787
H -4.44632200 -0.41093100 -0.53821800	O 1.154812 1.096551 0.739552	H -4.388975 -0.455541 -0.750706
O 1.13172600 1.08491700 0.64430500	C -0.944917 2.841668 0.166766	O 1.083258 1.078506 0.725762
C -0.93286300 2.86166000 0.20063700	H -1.471769 3.295405 -0.669259	C -0.843148 2.827029 0.097365
H -1.31077500 3.31956100 -0.70971200	H 0.109605 3.077557 0.084733	H -1.611304 3.387813 -0.422696
H 0.12327100 3.07967700 0.27578900	H -1.329036 3.300897 1.078900	H 0.093436 2.929707 -0.443301
H -1.43884800 3.32727500 1.04438900	C -3.593867 1.714345 -0.324827	H -0.697598 3.263384 1.085106
C -3.63715400 1.70776700 -0.24902300	H -4.492966 1.109568 -0.271602	C -3.656607 1.702121 -0.234031
H -4.42603800 1.45358600 0.45912100	H -3.651438 2.525815 0.400772	H -4.493173 1.335168 0.359610
H -3.40390500 2.75417500 -0.10659500	H -3.552008 2.169902 -1.317187	H -3.469137 2.722080 0.075123
H -4.03982700 1.59937500 -1.25729000	C -1.708577 -2.916120 0.102455	H -3.971705 1.730722 -1.279642
C -1.72782300 -2.90331300 0.04055500	H -0.993335 -3.407700 -0.559416	C -1.736425 -2.907220 0.053370
H -0.96702400 -3.37321600 -0.57757000	H -1.604850 -3.377988 1.087739	H -0.966292 -3.356656 -0.568736
H -1.66597000 -3.34940600 1.03235400	H -2.715708 -3.102743 -0.255181	H -1.669246 -3.369962 1.037786
H -2.70100600 -3.13584500 -0.37143800	C 3.487445 1.068166 0.969924	H -2.707859 -3.139961 -0.364859
C 3.45351100 1.07583500 0.99056000	H 4.401077 0.520658 0.753569	C 3.410641 1.095287 1.041633
H 4.37648700 0.52488500 0.84381500	H 3.519932 2.042832 0.492113	H 4.336735 0.565882 0.841992
H 3.51718100 2.03461400 0.48483100	H 3.390867 1.201336 2.043691	H 3.442250 2.066190 0.554076
H 3.29649800 1.24700700 2.05112800	C 0.912367 -1.802979 0.679885	H 3.286324 1.239024 2.114807
C 0.91168600 -1.79913200 0.59976800	H 0.629786 -2.563388 1.405481	C 0.911784 -1.795467 0.616474
H 0.61946900 -2.60144400 1.27215300	H 1.166548 -2.337641 -0.235782	H 0.634117 -2.630597 1.258014
H 1.19805200 -2.27531800 -0.33938000	C 2.132663 -1.043695 1.183403	H 1.214480 -2.210815 -0.345272
C 2.10129800 -1.04636700 1.17604500	H 2.011019 -0.797076 2.237145	C 2.096340 -1.038243 1.202329
H 1.92609300 -0.80177500 2.22173400	H 3.041781 -1.628239 1.070605	H 1.932406 -0.831578 2.261590
H 3.01112800 -1.63289700 1.10571900	C 2.269098 0.297263 0.472440	H 3.007816 -1.613052 1.082294
C 2.27450800 0.28046200 0.45085800	C 2.447350 0.158675 -1.040347	C 2.259183 0.287035 0.480398
C 2.48722300 0.07446800 -1.04593600	O 2.106439 0.959303 -1.860933	C 2.501654 0.074814 -1.053148
O 1.91116100 0.64649700 -1.92312000	O 3.162121 -0.945449 -1.381242	O 1.832609 0.776626 -1.826522
O 3.46511300 -0.82801400 -1.28376900	H 3.251963 -0.907397 -2.340441	O 3.385237 -0.776793 -1.294818
H 3.57051000 -0.87504000 -2.24162600		
**Trolox (C1$^-$)**	**Trolox (C2$^-$)**	**Trolox (C3$^-$)**
C -2.506251 0.786090 -0.067802	C -2.490479 0.803892 -0.027869	C -2.377482 0.778210 -0.022469
C -1.229305 1.414502 0.129989	C -1.181326 1.333890 0.169648	C -1.122626 1.378662 0.249120
C -0.143078 0.496684 0.329862	C -0.116311 0.477246 0.390896	C -0.071076 0.506573 0.488044
C -0.290382 -0.864975 0.344959	C -0.225905 -0.909490 0.397421	C -0.182880 -0.875476 0.453803
C -1.549532 -1.460061 0.096595	C -1.500134 -1.453774 0.107351	C -1.432739 -1.502400 0.128558
C -2.617980 -0.595802 -0.103762	C -2.570649 -0.625768 -0.087473	C -2.508301 -0.583853 -0.093497
O -3.857003 -1.192804 -0.343475	O -3.804854 -1.171419 -0.406384	O -3.741636 -1.166622 -0.379222
H -4.430765 -0.511522 -0.689768	H -4.265694 -0.464098 -0.876101	H -4.323640 -0.481081 -0.702976
O 1.092891 1.122528 0.557761	O 1.119717 1.094300 0.652758	O 1.166450 1.104968 0.785394
C -1.042978 2.784066 0.153407	C -1.010525 2.820407 0.152902	C -0.921594 2.869621 0.227445
H -1.855947 3.463532 -0.028766	H -1.375829 3.229922 -0.791734	H -1.297425 3.316566 -0.694508
H -0.059018 3.193391 0.287793	H 0.026902 3.104533 0.279972	H 0.136491 3.095426 0.291046
C -3.681093 1.705332 -0.207833	H -1.608964 3.284116 0.941732	H -1.418814 3.378953 1.058034
H -4.648119 1.204287 -0.197278	C -3.625888 1.592510 -0.216555	C -3.599560 1.636144 -0.252294
H -3.685124 2.415745 0.623224	H -4.617912 1.181387 -0.119004	H -4.427890 1.320393 0.383757
H -3.616782 2.305671 -1.120103	H -3.557121 2.665321 -0.214906	H -3.396158 2.675884 -0.024976
C -1.713137 -2.953603 0.040497	C -1.669141 -2.947163 0.015877	H -3.941943 1.594719 -1.289888
H -0.972159 -3.422346 -0.610396	H -0.914717 -3.386293 -0.636120	C -1.610063 -2.872166 0.053699
H -1.617145 -3.430673 1.021037	H -1.563671 -3.420603 0.993246	H -0.796175 -3.554917 0.220978
H -2.696100 -3.202285 -0.343932	H -2.651002 -3.189874 -0.372588	H -2.575910 -3.277442 -0.185652
C 3.395136 1.145246 0.968699	C 3.447618 1.108278 0.908163	C 3.508664 1.125725 0.901601

H	4.329699	0.602712	0.857168	H	4.371220	0.563844	0.730149	H	4.421692	0.588397	0.658067
H	3.447494	2.089136	0.433984	H	3.469441	2.055209	0.376969	H	3.502405	2.087800	0.397685
H	3.211732	1.347674	2.019829	H	3.338780	1.299929	1.971825	H	3.458751	1.284278	1.975194
C	0.888227	-1.764567	0.633287	C	0.931104	-1.815830	0.727016	C	0.966634	-1.780632	0.770757
H	0.587757	-2.543242	1.331714	H	0.645938	-2.539482	1.492629	H	0.663470	-2.483034	1.552201
H	1.206892	-2.277911	-0.276028	H	1.238772	-2.404218	-0.140940	H	1.198114	-2.398706	-0.101382
C	2.051562	-0.976264	1.212068	C	2.120569	-1.009168	1.227546	C	2.202734	-1.010006	1.213287
H	1.828378	-0.679434	2.235731	H	1.959339	-0.706826	2.261172	H	2.131228	-0.751106	2.268806
H	2.973119	-1.551387	1.207068	H	3.045398	-1.577069	1.171907	H	3.112328	-1.585956	1.063074
C	2.226990	0.322231	0.434033	C	2.246654	0.291254	0.443727	C	2.287310	0.319023	0.477642
C	2.524192	0.053941	-1.042085	C	2.438454	0.063705	-1.054953	C	2.379705	0.158104	-1.039161
O	2.119097	0.669417	-1.981544	O	2.080923	0.797091	-1.929563	O	2.012082	0.961972	-1.847746
O	3.427921	-0.957588	-1.198043	O	3.191101	-1.038323	-1.318518	O	3.046743	-0.964090	-1.406589
H	3.581250	-1.013867	-2.147718	H	3.285807	-1.058084	-2.277640	H	3.055830	-0.954555	-2.370432
**Trolox (C4$^-$)**				**Trolox (C5$^-$)**				**Trolox (C6$^-$)**			
C	-2.435038	0.839489	-0.078687	C	-2.484136	0.872988	0.004595	C	-2.327150	1.258690	0.111140
C	-1.156489	1.360030	0.216215	C	-1.186910	1.381645	0.085187	C	-1.494376	1.651263	1.150061
C	-0.127129	0.504424	0.514413	C	-0.096069	0.502999	0.159224	C	-0.439362	0.803626	1.620763
C	-0.245836	-0.919016	0.511979	C	-0.305169	-0.885383	0.192668	C	-0.294482	-0.459544	0.958801
C	-1.539897	-1.425622	0.177144	C	-1.606147	-1.382360	0.100957	C	-1.126787	-0.838811	-0.091980
C	-2.569672	-0.549593	-0.093540	C	-2.673968	-0.502205	0.014981	C	-2.134933	0.025421	-0.511825
O	-3.815292	-1.100861	-0.396472	O	-3.958798	-1.036284	-0.055748	O	-2.993980	-0.358888	-1.567460
H	-4.403208	-0.376226	-0.604733	H	-4.572103	-0.315332	-0.190751	H	-2.640748	0.003326	-2.379768
O	1.119500	1.067293	0.836426	O	1.129091	1.075574	0.186158	O	0.339723	1.147165	2.572162
C	-0.909526	2.847321	0.193510	C	-0.933563	2.868393	0.086918	C	-1.669113	2.982684	1.826525
H	-1.212560	3.273742	-0.761579	H	-1.188965	3.319390	-0.872262	H	-1.769045	3.796027	1.107346
H	0.142806	3.053287	0.339204	H	0.115270	3.054924	0.276002	H	-0.802391	3.157678	2.455054
H	-1.472110	3.357531	0.976231	H	-1.519710	3.370041	0.855477	H	-2.557249	3.003990	2.462659
C	-3.623538	1.717091	-0.369819	C	-3.690004	1.772691	-0.107435	C	-3.439230	2.170290	-0.343673
H	-4.460309	1.530343	0.312171	H	-4.358144	1.661499	0.749010	H	-4.142650	1.635765	-0.971684
H	-3.367654	2.764775	-0.258970	H	-3.402150	2.814734	-0.161078	H	-3.973287	2.577820	0.511740
H	-4.005530	1.597525	-1.390352	H	-4.267045	1.557848	-1.010113	H	-3.049055	3.019314	-0.907599
C	-1.729857	-2.908289	0.131903	C	-1.854201	-2.868410	0.087181	C	-0.948919	-2.171674	-0.778193
H	-1.044846	-3.358238	-0.592294	H	-1.145601	-3.365059	-0.569586	H	0.037449	-2.249625	-1.233990
H	-1.490029	-3.357543	1.099393	H	-1.727175	-3.295837	1.081660	H	-1.043136	-2.991133	-0.067877
H	-2.747264	-3.169788	-0.135199	H	-2.861571	-3.085029	-0.248132	H	-1.698717	-2.303886	-1.548706
C	3.452350	1.083783	1.011477	C	3.300911	1.005896	1.176354	C	1.967309	0.372411	-1.168516
H	4.372211	0.539948	0.815969	H	4.265015	0.520350	1.267339	H	2.045782	0.344715	-2.243399
H	3.481939	2.048412	0.511142	H	3.425489	1.977940	0.705849	H	1.519148	1.228585	-0.689700
H	3.345319	1.245431	2.080114	H	2.866390	1.142924	2.162334	C	0.837149	-1.318004	1.442758
C	0.853045	-1.722455	0.792795	C	0.871744	-1.826071	0.345478	H	0.813380	-1.335148	2.530969
H	0.770879	-2.794375	0.799690	H	0.571525	-2.655618	0.983637	H	0.763526	-2.338317	1.076291
C	2.140296	-1.082574	1.206221	H	1.085054	-2.260756	-0.637162	C	2.210722	-0.720920	1.049821
H	2.228927	-0.883400	2.284082	C	2.081121	-1.155012	0.949858	H	2.230817	0.283303	1.462003
H	3.001263	-1.685859	0.923852	H	2.080932	-1.137783	2.035643	H	3.008003	-1.304692	1.501943
C	2.250094	0.279459	0.530996	C	2.352362	0.136893	0.374929	C	2.388250	-0.655943	-0.435789
C	2.375022	0.124207	-0.978042	C	2.825916	-0.120483	-1.030062	C	2.937435	-1.813733	-1.178827
O	1.810366	0.779073	-1.808305	O	2.145714	-0.473401	-1.965951	O	3.153728	-1.875193	-2.366616
O	3.316932	-0.791723	-1.329427	O	4.175723	0.035612	-1.202070	O	3.198621	-2.880953	-0.383961
H	3.300493	-0.808754	-2.292164	H	4.331753	-0.270892	-2.100844	H	3.520788	-3.566964	-0.978324

Structures of trolox and their corresponding species in the SPLET (ETE_step) mechanism at M05-2X/6-311++G(2d,2p) level of theory in gas phase.

**M05-2X/6-311++G(2d,2p)**											
**Trolox (O1$^\bullet$)**				**Trolox (O2$^\bullet$)**				**Trolox (C1$^\bullet$)**			
C	-2.466583	-0.894659	0.020538	C	-2.292858	-0.818317	0.070512	C	-2.477815	-0.874767	0.040667
C	-1.202107	-1.371206	-0.187784	C	-1.010149	-1.312130	-0.154974	C	-1.181931	-1.429624	-0.158014
C	-0.139161	-0.451078	-0.339990	C	0.018442	-0.399493	-0.381191	C	-0.102266	-0.528345	-0.365852
C	-0.313229	0.940833	-0.294793	C	-0.194826	0.973180	-0.386479	C	-0.277283	0.839386	-0.359092
C	-1.571098	1.441788	-0.064525	C	-1.475988	1.464367	-0.140515	C	-1.546322	1.370154	-0.093512
C	-2.701160	0.542450	0.095212	C	-2.504463	0.559111	0.077532	C	-2.620471	0.497730	0.104495
O	-3.844578	0.995606	0.287175	O	-3.759906	1.071734	0.300401	O	-3.842046	1.085341	0.348887
O	1.071562	-1.023751	-0.560051	H	-4.363809	0.357897	0.505600	H	-4.431084	0.440566	0.739138
C	-0.869929	-2.835322	-0.240717	O	1.276980	-0.943481	-0.565618	O	1.115009	-1.120195	-0.593439
H	-1.747089	-3.441279	-0.056926	C	-0.729864	-2.792976	-0.154652	C	-0.944895	-2.801711	-0.138363
H	-0.114140	-3.071508	0.503899	H	-1.016147	-3.242149	0.793487	H	-1.736091	-3.503713	0.043665
H	-0.457166	-3.102034	-1.210757	H	0.321988	-2.984217	-0.315352	H	0.050401	-3.178634	-0.271880

C	-3.651963	-1.795734	0.196141
H	-4.546479	-1.195052	0.297329
H	-3.765334	-2.462046	-0.655684
H	-3.542556	-2.412302	1.086126
C	-1.826100	2.913644	0.015136
H	-1.103799	3.400930	0.665940
H	-1.744268	3.374787	-0.969166
H	-2.827082	3.087669	0.388129
C	3.378262	-1.024484	-1.017101
H	4.310559	-0.480947	-0.910692
H	3.458734	-1.984867	-0.517066
H	3.173365	-1.192119	-2.069953
C	0.865827	1.858134	-0.500341
H	0.573558	2.692719	-1.130974
H	1.169214	2.285419	0.456944
C	2.034491	1.113085	-1.128497
H	1.826176	0.886839	-2.172420
H	2.948650	1.694765	-1.077344
C	2.235090	-0.219531	-0.422910
C	2.486433	-0.008105	1.066042
O	1.842136	-0.476009	1.956788
O	3.559369	0.782498	1.269875
H	3.673982	0.861351	2.224865

H	-1.290391	-3.293843	-0.940930
C	-3.467945	-1.729144	0.310816
H	-4.252520	-1.560734	-0.427370
H	-3.185549	-2.771182	0.247513
H	-3.896818	-1.570899	1.301473
C	-1.740619	2.944529	-0.101604
H	-0.991974	3.452747	0.500241
H	-1.708091	3.374300	-1.101802
H	-2.717723	3.145231	0.317675
C	3.571525	-0.897436	-1.145430
H	4.447561	-0.256988	-1.104276
H	3.704010	-1.723404	-0.452539
H	3.504709	-1.314049	-2.155925
C	0.956252	1.908088	-0.644420
H	0.611194	2.776604	-1.197653
H	1.356888	2.262647	0.304943
C	2.050106	1.189988	-1.426959
H	1.708989	1.035418	-2.458658
H	2.957362	1.785211	-1.471651
C	2.352461	-0.122893	-0.803552
C	2.199771	-0.565265	2.255288
O	2.707913	-1.599050	2.168077
O	1.695468	0.467147	2.375387

C	-3.661443	-1.789641	0.189651
H	-4.598253	-1.285380	-0.029780
H	-3.590076	-2.614552	-0.512290
H	-3.723783	-2.214017	1.191215
C	-1.752512	2.855888	-0.034231
H	-0.963123	3.334479	0.539784
H	-1.740193	3.292048	-1.033126
H	-2.705612	3.090892	0.420305
C	3.423438	-1.117124	-1.023908
H	4.349925	-0.561595	-0.926413
H	3.507387	-2.060733	-0.493245
H	3.228783	-1.319766	-2.072596
C	0.886954	1.761231	-0.623602
H	0.580046	2.556191	-1.297894
H	1.194226	2.245816	0.304543
C	2.060702	0.999430	-1.220563
H	1.851958	0.729470	-2.253842
H	2.970495	1.589532	-1.193731
C	2.264015	-0.307064	-0.466046
C	2.518666	-0.046282	1.015239
O	1.928276	-0.539096	1.929269
O	3.544835	0.816414	1.188719
H	3.669649	0.910135	2.140784

Trolox (C2$^{\bullet}$)

C	-2.473896	-0.894877	0.033711
C	-1.163495	-1.414779	-0.158864
C	-0.121885	-0.534840	-0.368769
C	-0.308283	0.851388	-0.379983
C	-1.579547	1.381325	-0.124455
C	-2.634134	0.518464	0.082805
O	-3.865082	1.062301	0.329596
H	-4.412234	0.413367	0.773783
O	1.118199	-1.097382	-0.606579
C	-0.931944	-2.896593	-0.151791
H	-1.314069	-3.336268	0.766905
H	0.123729	-3.117170	-0.224968
H	-1.444625	-3.373700	-0.985727
C	-3.572905	-1.745300	0.160449
H	-4.584888	-1.382329	0.192866
H	-3.449447	-2.811241	0.161168
C	-1.789281	2.869758	-0.075205
H	-1.030912	3.347933	0.539335
H	-1.728140	3.305820	-1.071192
H	-2.763878	3.101864	0.333197
C	3.440476	-1.059899	-0.943387
H	4.354079	-0.490683	-0.808623
H	3.518117	-2.004393	-0.413417
H	3.289072	-1.260694	-1.999540
C	0.850247	1.779938	-0.632120
H	0.548666	2.565811	-1.319911
H	1.125324	2.279768	0.298942
C	2.055173	1.034800	-1.186261
H	1.889372	0.762367	-2.226500
H	2.953435	1.639989	-1.125910
C	2.247601	-0.270037	-0.427295
C	2.447916	-0.018845	1.063913
O	1.856780	-0.556402	1.952653
O	3.427682	0.886265	1.281838
H	3.523183	0.965990	2.238642

Trolox (C3$^{\bullet}$)

C	-2.435284	-0.804934	-0.000935
C	-1.158298	-1.332851	-0.206025
C	-0.086219	-0.445758	-0.399800
C	-0.242685	0.921378	-0.382877
C	-1.531085	1.473736	-0.126343
C	-2.605282	0.567811	0.049143
O	-3.833083	1.121918	0.274972
H	-4.470484	0.429516	0.452579
O	1.141152	-1.039477	-0.627654
C	-0.917355	-2.816277	-0.209642
H	-1.333955	-3.275557	0.683195
H	0.142428	-3.027179	-0.237071
H	-1.382600	-3.287879	-1.074156
C	-3.642223	-1.688048	0.182287
H	-4.430552	-1.422553	-0.522238
H	-3.402882	-2.729063	0.012911
H	-4.047227	-1.607056	1.191819
C	-1.755612	2.844941	-0.040060
H	-0.955979	3.552187	-0.154164
H	-2.742336	3.217814	0.157364
C	3.475771	-1.043342	-0.886275
H	4.395081	-0.496294	-0.705247
H	3.515128	-2.000724	-0.375473
H	3.359805	-1.216503	-1.951788
C	0.926128	1.841086	-0.613706
H	0.647303	2.611587	-1.328401
H	1.175295	2.355796	0.315591
C	2.139396	1.082614	-1.131182
H	2.009308	0.832181	-2.181932
H	3.045364	1.669733	-1.026188
C	2.281462	-0.240301	-0.394278
C	2.438290	-0.036584	1.109929
O	1.849660	-0.631667	1.963218
O	3.385498	0.886610	1.385274
H	3.458714	0.929737	2.346371

Trolox (C4$^{\bullet}$)

C	-2.359029	-0.839582	0.029255
C	-1.091399	-1.357585	-0.252892
C	-0.058436	-0.471087	-0.490714
C	-0.235464	0.933140	-0.447739
C	-1.522590	1.444368	-0.132427
C	-2.544465	0.550445	0.090641
O	-3.786636	1.064245	0.381416
H	-4.391444	0.348022	0.573849
O	1.175183	-0.992239	-0.812863
C	-0.835833	-2.840454	-0.282806
H	-1.137297	-3.300209	0.654797
H	0.217130	-3.035599	-0.431372
H	-1.391212	-3.319563	-1.086639
C	-3.540512	-1.733815	0.285177
H	-4.356716	-1.513992	-0.404312
H	-3.285455	-2.776933	0.157190
H	-3.917406	-1.614815	1.302498
C	-1.752672	2.924564	-0.054559
H	-1.081326	3.379993	0.671137
H	-1.566017	3.397808	-1.017264
H	-2.772151	3.137045	0.238118
C	3.525075	-0.955780	-0.926306
H	4.423522	-0.402077	-0.672632
H	3.565164	-1.941736	-0.473428
H	3.458612	-1.064924	-2.004422
C	0.851581	1.767263	-0.727732
H	0.725944	2.834267	-0.750462
C	2.182136	1.180882	-1.050824
H	2.310266	1.067642	-2.130903
H	2.991316	1.809819	-0.692435
C	2.294547	-0.208689	-0.438221
C	2.356645	-0.151922	1.086405
O	1.750452	-0.864064	1.831272
O	3.245464	0.766960	1.517159
H	3.257494	0.709235	2.480139

Trolox (C5$^{\bullet}$)

C	-2.398581	-0.847426	0.227844
C	-1.167724	-1.376826	-0.151553
C	-0.167044	-0.496339	-0.553164
C	-0.353578	0.878026	-0.559530
C	-1.579887	1.410701	-0.161596
C	-2.582675	0.534806	0.224082
O	-3.782897	1.080420	0.613233
H	-4.379793	0.379495	0.875120
O	1.028681	-1.047417	-0.980614
C	-0.900015	-2.857098	-0.117169
H	-1.127777	-3.263742	0.864525
H	0.141662	-3.053146	-0.329582

Trolox (C6$^{\bullet}$)

C	-2.685588	0.497563	0.218283
C	-2.531290	-0.857070	0.066867
C	-1.276843	-1.383564	-0.450775
C	-0.213083	-0.457798	-0.822141
C	-0.384382	0.890187	-0.651975
C	-1.619085	1.345311	-0.135972
O	-1.734306	2.684430	0.012656
H	-2.597957	2.904627	0.367893
O	-1.106077	-2.611316	-0.570606
C	-3.605823	-1.841862	0.414709
H	-3.868423	-1.780173	1.468984
H	-3.253917	-2.842547	0.201087

H	-1.507319	-3.384640	-0.850231	H	-4.508911	-1.655708	-0.162996
C	-3.544869	-1.724612	0.658075	C	-3.948372	1.119430	0.752832
H	-4.428555	-1.543254	0.045029	H	-4.399295	1.792431	0.022905
H	-3.301400	-2.774071	0.562452	H	-4.684439	0.363790	0.991505
H	-3.813198	-1.546007	1.700172	H	-3.757158	1.684357	1.665942
C	-1.811568	2.896208	-0.157074	C	0.654263	1.934578	-0.954739
H	-1.235368	3.377697	0.631732	H	0.988703	2.409359	-0.034992
H	-1.506635	3.339507	-1.102006	H	1.516986	1.511780	-1.450805
H	-2.857433	3.119645	0.006782	H	0.235566	2.713583	-1.585535
C	3.367680	-1.015956	-1.294444	C	1.858928	-0.284271	1.950851
H	4.290696	-0.498498	-1.053979	H	2.218296	0.532468	2.554988
H	3.415140	-2.037714	-0.930811	H	1.066422	-0.908733	2.331622
H	3.224055	-1.023892	-2.370044	C	1.047380	-1.092122	-1.324868
C	0.756961	1.795834	-1.003813	H	0.776808	-1.949568	-1.932364
H	0.483986	2.273598	-1.948898	H	1.621474	-0.407875	-1.937288
H	0.851630	2.619875	-0.289140	C	1.921768	-1.610843	-0.161613
C	2.056351	1.099145	-1.147283	H	1.335718	-2.324961	0.408637
H	2.935016	1.628225	-1.471168	H	2.777406	-2.132441	-0.580996
C	2.185754	-0.295900	-0.657588	C	2.386463	-0.517444	0.755061
C	2.379576	-0.360045	0.865342	C	3.472925	0.401564	0.317079
O	1.824684	-1.115217	1.607271	O	3.887434	1.348937	0.930973
O	3.313213	0.521203	1.279209	O	3.979702	0.068318	-0.891184
H	3.420788	0.387451	2.228712	H	4.675409	0.709575	-1.078349

Structure of trolox and the corresponding radical species in the HAT mechanism optimized at the M06-2X/6-311++G(2d,2p) level of theory in gas phase.

**M06-2X/6-311++G(2d,2p)**											
**Trolox**				**Trolox (O1$^\bullet$)**				**Trolox (O2$^\bullet$)**			
C	-2.42589000	0.83929300	-0.04265000	C	-2.450660	0.866970	-0.046931	C	-2.475649	0.843114	-0.012743
C	-1.15910900	1.37560300	0.18403500	C	-1.191350	1.372659	0.138674	C	-1.218125	1.390256	0.237904
C	-0.09148700	0.49975900	0.38968100	C	-0.112856	0.474922	0.335354	C	-0.142556	0.520696	0.431299
C	-0.25813700	-0.88083000	0.36341900	C	-0.266850	-0.923349	0.347016	C	-0.290880	-0.862517	0.391663
C	-1.52326000	-1.41656200	0.10546100	C	-1.518019	-1.454064	0.136970	C	-1.550146	-1.406683	0.126737
C	-2.58808400	-0.54730000	-0.08660800	C	-2.665356	-0.578946	-0.060215	C	-2.622527	-0.544978	-0.065588
O	-3.82203800	-1.10477000	-0.31690900	O	-3.801236	-1.056367	-0.233275	O	-3.848043	-1.113786	-0.305773
H	-4.45326300	-0.41386300	-0.52421400	O	1.086422	1.074057	0.536746	H	-4.497813	-0.426653	-0.462102
O	1.13466100	1.08336500	0.64536900	C	-0.877697	2.843230	0.117623	O	1.084327	1.118425	0.642233
C	-0.92734300	2.86481400	0.19959400	H	-1.756944	3.432175	-0.122234	C	-1.010617	2.882406	0.300290
H	-1.28630900	3.32235400	-0.72248000	H	-0.105142	3.048432	-0.623433	H	-1.262912	3.353128	-0.650821
H	0.12964000	3.08586600	0.29634800	H	-0.488810	3.173494	1.081521	H	0.022133	3.120344	0.528912
H	-1.45230900	3.33894700	1.03072800	C	-3.649874	1.742925	-0.262839	H	-1.641381	3.331600	1.068246
C	-3.63608400	1.71323800	-0.25042100	H	-4.546498	1.131794	-0.289813	C	-3.692896	1.704125	-0.235368
H	-4.42348000	1.47334900	0.46910700	H	-3.746765	2.482837	0.531807	H	-4.453029	1.522725	0.529433
H	-3.40162700	2.76518400	-0.12735900	H	-3.572422	2.284715	-1.207334	H	-3.453611	2.761762	-0.206702
H	-4.05254500	1.59109500	-1.25474400	C	-1.748448	-2.933722	0.117767	H	-4.146260	1.506340	-1.211012
C	-1.72902800	-2.90596200	0.04324900	H	-1.024773	-3.436642	-0.525120	C	-1.746626	-2.897197	0.053293
H	-0.98351900	-3.37639800	-0.59871100	H	-1.643050	-3.358948	1.119194	H	-0.958253	-3.366809	-0.534653
H	-1.63636600	-3.35848100	1.03305900	H	-2.753419	-3.143353	-0.234963	H	-1.722537	-3.346205	1.048726
H	-2.71535100	-3.14298700	-0.34236000	C	3.392205	1.139364	0.994637	H	-2.704349	-3.137522	-0.397280
C	3.45643400	1.08234100	0.99010400	H	4.338880	0.611304	0.907888	C	3.415713	1.149128	1.034188
H	4.38515900	0.53555600	0.84169300	H	3.455136	2.083176	0.455239	H	4.308710	0.533819	0.930361
H	3.51803100	2.04415500	0.48311500	H	3.189608	1.348711	2.043511	H	3.479348	1.988085	0.341263
H	3.30536700	1.25653600	2.05411300	C	0.929229	-1.811960	0.589726	H	3.406516	1.558396	2.052664
C	0.91509400	-1.80222700	0.59893700	H	0.650235	-2.634148	1.247884	C	0.894065	-1.762366	0.639637
H	0.62262300	-2.61094500	1.26912300	H	1.243985	-2.269419	-0.354180	H	0.584306	-2.627289	1.226422
H	1.20147000	-2.27982300	-0.34393900	C	2.085400	-1.023813	1.191364	H	1.274423	-2.148090	-0.310014
C	2.10663500	-1.05019200	1.17618000	H	1.873439	-0.764945	2.230295	C	2.004105	-1.008314	1.366267
H	1.93362600	-0.81193700	2.22703500	H	3.012374	-1.592676	1.163168	H	1.731531	-0.897297	2.426655
H	3.01955600	-1.63768600	1.10387300	C	2.263080	0.288258	0.434712	H	2.941697	-1.560984	1.327974
C	2.27735000	0.28233500	0.45296100	C	2.515217	0.018242	-1.051014	C	2.198931	0.338816	0.764683
C	2.48268500	0.07311100	-1.05077900	O	1.850396	0.429278	-1.953734	C	3.116442	-0.269303	-1.986913
O	1.89262300	0.63983100	-1.92103800	O	3.605805	-0.753388	-1.224953	O	2.784903	0.771724	-2.360629
O	3.46083300	-0.82643000	-1.29265100	H	3.720596	-0.878006	-2.176055	O	3.467617	-1.318558	-1.646930
H	3.56201700	-0.88140500	-2.25153100								
**Trolox (C1$^\bullet$)**				**Trolox (C2$^\bullet$)**				**Trolox (C3$^\bullet$)**			
C	-2.494979	0.893683	-0.005810	C	-2.477420	0.794237	-0.046161	C	-2.410723	0.806803	-0.009829
C	-1.189660	1.340619	0.359665	C	-1.177224	1.347019	0.147108	C	-1.141272	1.352739	0.208132
C	-0.148155	0.371873	0.422873	C	-0.113641	0.492719	0.364658	C	-0.057962	0.479255	0.420454
C	-0.374174	-0.965260	0.157009	C	-0.267836	-0.900484	0.383954	C	-0.197415	-0.892094	0.407433
C	-1.660645	-1.388497	-0.204243	C	-1.526783	-1.463153	0.129673	C	-1.477621	-1.463865	0.139336
C	-2.701142	-0.449950	-0.263599	C	-2.604418	-0.626282	-0.086055	C	-2.564186	-0.571197	-0.053086
O	-3.934478	-0.954408	-0.594429	O	-3.817042	-1.204206	-0.329276	O	-3.778388	-1.145192	-0.287885
H	-4.595654	-0.267611	-0.507727	H	-4.395071	-0.564197	-0.749603	H	-4.432058	-0.464313	-0.458911
O	1.078587	0.859403	0.790888	O	1.110275	1.085334	0.600726	O	1.156645	1.089226	0.660680

C	-0.912155	2.674682	0.653505	C	-0.981281	2.835012	0.129613	C	-0.918379	2.840420	0.205157
H	0.089628	2.978157	0.904774	H	-1.362054	3.259643	-0.800580	H	-1.309722	3.288474	-0.708681
H	-1.680932	3.428947	0.635692	H	0.069687	3.086513	0.216369	H	0.139410	3.069109	0.267698
C	-3.597955	1.911853	-0.107143	H	-1.520163	3.310851	0.951694	H	-1.421795	3.318992	1.047926
H	-3.867097	2.307884	0.874224	C	-3.596329	1.618611	-0.183244	C	-3.626635	1.674351	-0.215043
H	-3.279524	2.753935	-0.722018	H	-4.603804	1.233122	-0.222755	H	-4.419882	1.413559	0.490608
H	-4.507129	1.529701	-0.568290	H	-3.501603	2.691304	-0.188078	H	-3.403397	2.725128	-0.064591
C	-1.927435	-2.835650	-0.510429	C	-1.700151	-2.957740	0.091819	H	-4.029285	1.569925	-1.226553
H	-1.157160	-3.244705	-1.164746	H	-0.937432	-3.423808	-0.532482	C	-1.684069	-2.840045	0.058027
H	-1.928861	-3.437237	0.402405	H	-1.610478	-3.389212	1.090978	H	-0.875851	-3.541314	0.184116
H	-2.891851	-2.959813	-0.991449	H	-2.676764	-3.221582	-0.300534	H	-2.666585	-3.230340	-0.147723
C	3.370785	0.691566	1.272464	C	3.432841	1.116039	0.933905	C	3.487918	1.135116	0.941973
H	4.285849	0.130619	1.096163	H	4.365882	0.572641	0.801535	H	4.422202	0.604314	0.771411
H	3.503529	1.716500	0.929319	H	3.484491	2.060740	0.394812	H	3.516926	2.093307	0.425354
H	3.154447	0.705437	2.339322	H	3.282736	1.324770	1.991793	H	3.364315	1.314964	2.008593
C	0.750768	-1.966544	0.270383	C	0.914177	-1.799228	0.647334	C	0.982157	-1.795713	0.657023
H	0.396404	-2.867417	0.771218	H	0.629657	-2.590667	1.341555	H	0.704853	-2.570898	1.372498
H	1.070986	-2.279661	-0.728852	H	1.202673	-2.304407	-0.281241	H	1.248615	-2.315390	-0.269190
C	1.932224	-1.378407	1.030574	C	2.101870	-1.020198	1.196281	C	2.181590	-1.018788	1.183551
H	1.701592	-1.306293	2.094885	H	1.930888	-0.749001	2.239392	H	2.040998	-0.772101	2.237157
H	2.824547	-1.989724	0.913642	H	3.017858	-1.604604	1.139720	H	3.099734	-1.594381	1.086713
C	2.202550	0.042355	0.544188	C	2.259468	0.288225	0.427989	C	2.310277	0.308364	0.444143
C	2.490605	0.052279	-0.960161	C	2.457344	0.025171	-1.067820	C	2.478108	0.097178	-1.064207
O	1.934712	0.725619	-1.774196	O	1.837561	0.533227	-1.953355	O	1.878576	0.677501	-1.919004
O	3.492007	-0.799651	-1.269602	O	3.460206	-0.853408	-1.282441	O	3.438467	-0.813753	-1.329553
H	3.641304	-0.726189	-2.220932	H	3.552084	-0.949251	-2.239135	H	3.518360	-0.867421	-2.290576
**Trolox (C4$^{\bullet}$)**				**Trolox (C5$^{\bullet}$)**				**Trolox (C6$^{\bullet}$)**			
C	-2.399569	0.834248	-0.072186	C	-2.410447	0.830574	-0.123068	C	-2.451061	0.847996	-0.077441
C	-1.142256	1.378718	0.215484	C	-1.159283	1.363260	0.186198	C	-1.191023	1.390752	0.169599
C	-0.101271	0.513541	0.507371	C	-0.127219	0.483870	0.514038	C	-0.125400	0.520224	0.405430
C	-0.261073	-0.896155	0.511921	C	-0.305938	-0.894239	0.513413	C	-0.285208	-0.861139	0.383420
C	-1.536610	-1.435226	0.186310	C	-1.552633	-1.429959	0.180578	C	-1.542616	-1.403421	0.102046
C	-2.567156	-0.562379	-0.090349	C	-2.585888	-0.555581	-0.130774	C	-2.606723	-0.539608	-0.116536
O	-3.792234	-1.106319	-0.388368	O	-3.800311	-1.108413	-0.453490	O	-3.833306	-1.103325	-0.368753
H	-4.403463	-0.410340	-0.634121	H	-4.417664	-0.412670	-0.684826	H	-4.461582	-0.417008	-0.599002
O	1.116801	1.063628	0.831724	O	1.085254	1.043720	0.869625	O	1.091559	1.111920	0.688083
C	-0.903750	2.865787	0.193181	C	-0.902723	2.846884	0.154334	C	-0.963914	2.880627	0.174778
H	-1.205119	3.290627	-0.764297	H	-1.212729	3.271463	-0.800236	H	-1.334067	3.331562	-0.745808
H	0.148250	3.082642	0.342212	H	0.154193	3.050547	0.286020	H	0.093490	3.105275	0.258214
H	-1.471832	3.371421	0.975781	H	-1.452770	3.362860	0.943324	H	-1.480663	3.358102	1.009226
C	-3.587565	1.705106	-0.381133	C	-3.587352	1.707868	-0.465753	C	-3.661111	1.715616	-0.310975
H	-4.425124	1.482967	0.286218	H	-4.444675	1.485773	0.175719	H	-4.466948	1.460348	0.382197
H	-3.358047	2.758813	-0.263665	H	-3.359166	2.760434	-0.334696	H	-3.438860	2.767521	-0.166651
H	-3.934322	1.562749	-1.409443	H	-3.901792	1.570293	-1.504393	H	-4.047050	1.603051	-1.328488
C	-1.744724	-2.921452	0.154943	C	-1.771705	-2.918305	0.165465	C	-1.740867	-2.893954	0.043909
H	-1.046772	-3.394751	-0.538206	H	-1.194030	-3.391374	-0.631518	H	-0.985509	-3.363717	-0.587008
H	-1.573204	-3.360960	1.139854	H	-1.454856	-3.371072	1.106081	H	-1.658009	-3.341612	1.036759
H	-2.755949	-3.163769	-0.154498	H	-2.818929	-3.154161	0.007471	H	-2.721279	-3.137298	-0.352556
C	3.462440	1.072400	1.012561	C	3.433245	1.034021	1.087619	C	3.395711	1.092866	1.038869
H	4.379170	0.522412	0.808430	H	4.353725	0.520818	0.816407	H	4.350363	0.607036	1.159935
H	3.504958	2.041654	0.518197	H	3.459879	2.054374	0.708951	H	3.311039	2.165580	1.105406
H	3.366810	1.229338	2.085542	H	3.337220	1.057721	2.171445	C	0.886103	-1.777854	0.645534
C	0.832617	-1.705346	0.847579	C	0.835304	-1.814601	0.876092	H	0.583581	-2.584064	1.314166
H	0.722036	-2.776533	0.909650	H	0.596535	-2.354482	1.799464	H	1.191780	-2.258692	-0.289431
C	2.146782	-1.085354	1.184692	H	0.925038	-2.597878	0.110599	C	2.064310	-1.022442	1.244583
H	2.244400	-0.931863	2.266380	C	2.126237	-1.096650	1.026402	H	1.876286	-0.781026	2.291460
H	2.977845	-1.716843	0.873547	H	3.022516	-1.622480	1.317540	H	2.982996	-1.601779	1.184253
C	2.256235	0.285046	0.521564	C	2.230330	0.296561	0.509981	C	2.238635	0.314568	0.520959
C	2.356870	0.165401	-1.003537	C	2.353894	0.336683	-1.026660	C	2.470358	0.099207	-0.988739
O	1.745954	0.828148	-1.787906	O	1.753017	1.073286	-1.749803	O	1.907309	0.680666	-1.866025
O	3.276177	-0.748858	-1.372798	O	3.274207	-0.543690	-1.468886	O	3.437664	-0.816188	-1.208321
H	3.309801	-0.739738	-2.337908	H	3.338537	-0.428836	-2.425744	H	3.567429	-0.866412	-2.163996

Structures of trolox in the SET-PT (SET_step) mechanism at M06-2X/6-311++G(2d,2p) level of theory in gas phase.

**M06-2X/6-311++G(2d,2p)**			
Trolox $^{\bullet+}$			
C	-2.43572900	-0.87217000	0.06489200
C	-1.19730900	-1.40977600	-0.16023500

C	-0.09239300	-0.50648900	-0.34022200
C	-0.24373200	0.91116500	-0.36187000
C	-1.48320900	1.44450800	-0.13397800
C	-2.57101400	0.54605400	0.07809000
O	-3.74872300	1.10711500	0.27930700
H	-4.44537500	0.45211300	0.41293600
O	1.07642200	-1.08462500	-0.50402000
C	-0.95670500	-2.89135700	-0.18991500
H	-1.12153700	-3.32084200	0.79921800
H	0.05686600	-3.12393400	-0.49326100
H	-1.64432700	-3.37437900	-0.88257300
C	-3.66295600	-1.70262700	0.31302500
H	-4.41144400	-1.54780600	-0.46842200
H	-3.43301900	-2.76172500	0.33451700
H	-4.11488200	-1.45539200	1.27715300
C	-1.72375800	2.92431300	-0.11786900
H	-0.89480800	3.44267500	0.35935800
H	-1.81312800	3.30568200	-1.13749900
H	-2.63937300	3.17034100	0.40955000
C	3.39055200	-1.20058200	-0.98253900
H	4.34363600	-0.68125700	-0.92505100
H	3.45709000	-2.12394400	-0.41002300
H	3.17552700	-1.44083800	-2.02207500
C	0.95075100	1.78591100	-0.63663800
H	0.66651100	2.58532200	-1.31955300
H	1.25387200	2.26893900	0.29755300
C	2.10947800	0.98297900	-1.21654900
H	1.91438800	0.71291400	-2.25560400
H	3.03508300	1.55373600	-1.18604300
C	2.29341900	-0.31083500	-0.43418700
C	2.49751400	0.01360200	1.05051500
O	1.65293300	-0.11765400	1.88722500
O	3.71455300	0.51098400	1.26385000
H	3.80399500	0.72366300	2.20380300

Structures of trolox in the SET-PT (PT_step) mechanism at M06-2X/6-311++G(2d,2p) level of theory in gas phase.

**M06-2X/6-311++G(2d,2p)**											
Trolox $^{\bullet+}$				**Trolox (O1$^{\bullet}$)**				**Trolox (O2$^{\bullet}$)**			
C	-2.43572900	-0.87217000	0.06489200	C	-2.464187	-0.847808	0.044419	C	-2.360373	-0.755118	0.142898
C	-1.19730900	-1.40977600	-0.16023500	C	-1.217351	-1.372348	-0.170103	C	-1.101605	-1.309596	-0.090851
C	-0.09239300	-0.50648900	-0.34022200	C	-0.122630	-0.490841	-0.348956	C	-0.048254	-0.450092	-0.407189
C	-0.24373200	0.91116500	-0.36187000	C	-0.252215	0.909829	-0.339051	C	-0.213483	0.929006	-0.491336
C	-1.48320900	1.44450800	-0.13397800	C	-1.493050	1.459048	-0.115510	C	-1.468737	1.482342	-0.232506
C	-2.57101400	0.54605400	0.07809000	C	-2.653439	0.601270	0.081470	C	-2.523179	0.630160	0.074370
O	-3.74872300	1.10711500	0.27930700	O	-3.779202	1.095846	0.271810	O	-3.747070	1.205939	0.305196
H	-4.44537500	0.45211300	0.41293600	O	1.066585	-1.107852	-0.555884	H	-4.373988	0.533007	0.575616
O	1.07642200	-1.08462500	-0.50402000	C	-0.933741	-2.848795	-0.199394	O	1.181395	-1.051643	-0.596059
C	-0.95670500	-2.89135700	-0.18991500	H	-0.245562	-3.114888	0.603789	C	-0.871604	-2.797700	-0.005312
H	-1.12153700	-3.32084200	0.79921800	H	-0.452006	-3.131597	-1.135284	H	-1.155074	-3.181830	0.975133
H	0.05686600	-3.12393400	-0.49326100	H	-1.843863	-3.429088	-0.088911	H	0.172109	-3.039051	-0.173128
H	-1.64432700	-3.37437900	-0.88257300	C	-3.675336	-1.706890	0.262251	H	-1.465752	-3.329064	-0.750210
C	-3.66295600	-1.70262700	0.31302500	H	-3.890667	-2.312604	-0.619648	C	-3.559163	-1.607143	0.472208
H	-4.41144400	-1.54780600	-0.46842200	H	-3.527269	-2.389621	1.099521	H	-4.370544	-1.440055	-0.241328
H	-3.43301900	-2.76172500	0.33451700	H	-4.535331	-1.077867	0.468932	H	-3.324047	-2.665820	0.445179
H	-4.11488200	-1.45539200	1.27715300	C	-1.698129	2.942080	-0.078653	H	-3.943963	-1.388306	1.472653
C	-1.72375800	2.92431300	-0.11786900	H	-0.963989	3.425628	0.567221	C	-1.677798	2.972319	-0.275530
H	-0.89480800	3.44267500	0.35935800	H	-1.588492	3.376407	-1.075720	H	-0.893830	3.488893	0.278225
H	-1.81312800	3.30568200	-1.13749900	H	-2.698219	3.164825	0.279787	H	-1.652798	3.344206	-1.302058
H	-2.63937300	3.17034100	0.40955000	C	3.372052	-1.206016	-1.009330	H	-2.638284	3.238789	0.153696
C	3.39055200	-1.20058200	-0.98253900	H	4.327129	-0.694775	-0.914519	C	3.465579	-1.129025	-1.202966
H	4.34363600	-0.68125700	-0.92505100	H	3.417572	-2.156235	-0.479514	H	4.369350	-0.521995	-1.212467
H	3.45709000	-2.12394400	-0.41002300	H	3.168947	-1.401028	-2.060879	H	3.579282	-1.921457	-0.464277
H	3.17552700	-1.44083800	-2.02207500	C	0.957555	1.781243	-0.576286	H	3.366024	-1.601026	-2.189244
C	0.95075100	1.78591100	-0.63663800	H	0.690172	2.613313	-1.226853	C	0.959989	1.805122	-0.847455
H	0.66651100	2.58532200	-1.31955300	H	1.281251	2.225679	0.370777	H	0.627611	2.641170	-1.462394
H	1.25387200	2.26893900	0.29755300	C	2.099941	0.980022	-1.186973	H	1.389348	2.227662	0.064982
C	2.10947800	0.98297900	-1.21654900	H	1.882921	0.735044	-2.228236	C	2.020881	0.997626	-1.589746
H	1.91438800	0.71291400	-2.25560400	H	3.036355	1.532918	-1.153873	H	1.665577	0.788914	-2.610551
H	3.03508300	1.55373600	-1.18604300	C	2.255653	-0.342304	-0.443361	H	2.950450	1.557315	-1.681816

C	2.29341900	-0.31083500	-0.43418700	C	2.509033	-0.090144	1.045351	C	2.281931	-0.287432	-0.887094
C	2.49751400	0.01360200	1.05051500	O	1.835779	-0.497820	1.943253	C	2.104062	-0.524647	2.123926
O	1.65293300	-0.11765400	1.88722500	O	3.611903	0.661958	1.227974	O	2.610517	-1.562643	2.085883
O	3.71455300	0.51098400	1.26385000	H	3.726371	0.777082	2.180314	O	1.600611	0.513293	2.192617
H	3.80399500	0.72366300	2.20380300								
**Trolox (C1$^{\bullet}$)**				**Trolox (C2$^{\bullet}$)**				**Trolox (C3$^{\bullet}$)**			
C	-2.522557	-0.789432	0.038469	C	-2.482861	-0.764901	0.104094	C	-2.423168	-0.789089	0.033526
C	-1.236328	-1.384958	-0.122691	C	-1.205136	-1.337957	-0.167445	C	-1.162240	-1.358695	-0.173018
C	-0.126957	-0.518259	-0.338896	C	-0.130812	-0.501188	-0.396930	C	-0.068701	-0.507113	-0.419180
C	-0.266485	0.855850	-0.376039	C	-0.254674	0.895269	-0.375795	C	-0.190042	0.865669	-0.450249
C	-1.526904	1.427886	-0.151527	C	-1.501895	1.476963	-0.110621	C	-1.461124	1.462867	-0.194431
C	-2.631263	0.589998	0.054247	C	-2.593710	0.657769	0.105332	C	-2.558240	0.591517	0.032694
O	-3.834069	1.223376	0.254702	O	-3.796455	1.261192	0.327421	O	-3.763396	1.189004	0.255126
H	-4.472837	0.596127	0.595382	H	-4.497247	0.609633	0.268894	H	-4.424969	0.522842	0.451788
O	1.075206	-1.148033	-0.528619	O	1.075654	-1.112642	-0.673990	O	1.136452	-1.140710	-0.645446
C	-1.036048	-2.763447	-0.061332	C	-1.034738	-2.829378	-0.171909	C	-0.958995	-2.848344	-0.122676
H	-0.046313	-3.173414	-0.166682	H	-1.310418	-3.249686	0.796826	H	-1.350622	-3.261049	0.807553
H	-1.849919	-3.444555	0.122964	H	-0.005822	-3.099378	-0.380543	H	0.095271	-3.093127	-0.183737
C	-1.694814	2.921109	-0.146275	H	-1.674892	-3.293213	-0.924870	H	-1.473877	-3.347353	-0.946445
H	-0.904930	3.402170	0.431198	C	-3.585849	-1.575493	0.381630	C	-3.649142	-1.633301	0.273307
H	-1.645246	3.325756	-1.160628	H	-3.508690	-2.649182	0.354336	H	-4.443561	-1.383906	-0.435127
H	-2.654015	3.200957	0.276544	H	-4.544892	-1.184303	0.686585	H	-3.440914	-2.691170	0.154241
C	3.390298	-1.229244	-0.912837	C	-1.652903	2.974532	-0.076904	H	-4.043555	-1.492052	1.283587
H	4.335437	-0.700148	-0.813600	H	-0.896416	3.427172	0.564854	C	-1.648990	2.843608	-0.157114
H	3.436205	-2.160159	-0.349703	H	-1.533303	3.404587	-1.073547	H	-0.832330	3.529556	-0.310229
H	3.214715	-1.463344	-1.961435	H	-2.632336	3.256390	0.294822	H	-2.625114	3.253500	0.040823
C	0.928314	1.737881	-0.650165	C	3.392938	-1.184048	-1.038548	C	3.465370	-1.226759	-0.936733
H	0.653912	2.523747	-1.354050	H	4.339157	-0.665057	-0.901482	H	4.407466	-0.703194	-0.788182
H	1.233241	2.246646	0.270203	H	3.430653	-2.145396	-0.528450	H	3.484507	-2.167914	-0.389263
C	2.092961	0.926422	-1.201955	H	3.225926	-1.357661	-2.100274	H	3.333711	-1.439578	-1.996297
H	1.898010	0.635823	-2.235719	C	0.942706	1.775456	-0.632987	C	0.999899	1.744889	-0.735218
H	3.021122	1.493329	-1.174142	H	0.667324	2.591647	-1.301526	H	0.728885	2.499913	-1.474275
C	2.243766	-0.366398	-0.405336	H	1.254076	2.248578	0.305086	H	1.278220	2.290950	0.172134
C	2.472071	-0.062706	1.078437	C	2.106349	0.987286	-1.219662	C	2.186126	0.935295	-1.242083
O	1.839167	-0.505771	1.988483	H	1.917231	0.751477	-2.268194	H	2.036560	0.656256	-2.286341
O	3.518066	0.774748	1.248254	H	3.035586	1.549742	-1.157093	H	3.112270	1.501471	-1.168738
H	3.624798	0.904274	2.199339	C	2.244436	-0.346467	-0.493089	C	2.301398	-0.368605	-0.460339
C	-3.725438	-1.678420	0.200438	C	2.467785	-0.134859	1.007464	C	2.480203	-0.110596	1.039450
H	-3.778152	-2.113904	1.201020	O	1.853968	-0.663439	1.885084	O	1.877919	-0.654874	1.915752
H	-4.663327	-1.159542	0.006640	O	3.486856	0.721478	1.235957	O	3.453699	0.795832	1.270057
H	-3.685640	-2.502088	-0.511203	H	3.594596	0.782585	2.193825	H	3.539566	0.879705	2.228400
**Trolox (C4$^{\bullet}$)**				**Trolox (C5$^{\bullet}$)**				**Trolox (C6$^{\bullet}$)**			
C	-2.411255	-0.815056	0.102779	C	-2.421041	-0.810661	0.153228	C	-2.382074	-0.802276	0.111169
C	-1.163832	-1.384535	-0.179636	C	-1.179430	-1.368317	-0.150443	C	-1.130731	-1.350353	-0.165814
C	-0.113701	-0.542150	-0.503531	C	-0.138412	-0.512080	-0.509799	C	-0.072328	-0.483897	-0.447272
C	-0.254372	0.868974	-0.545727	C	-0.298975	0.867790	-0.545841	C	-0.226340	0.898935	-0.440290
C	-1.519853	1.434313	-0.225557	C	-1.536016	1.429031	-0.219062	C	-1.475661	1.446276	-0.130225
C	-2.559881	0.583662	0.083052	C	-2.578267	0.577381	0.123698	C	-2.533585	0.586848	0.133250
O	-3.775090	1.152402	0.375055	O	-3.782905	1.154968	0.439687	O	-3.750602	1.156127	0.414755
H	-4.394506	0.471582	0.641864	H	-4.408099	0.473947	0.692760	H	-4.379721	0.472086	0.649552
O	1.094114	-1.117575	-0.822324	O	1.063961	-1.097530	-0.858570	O	1.139255	-1.075709	-0.752159
C	-0.945293	-2.873569	-0.118170	C	-0.942283	-2.853822	-0.079716	C	-0.903819	-2.840387	-0.161644
H	-1.242966	-3.267519	0.853572	H	-1.247784	-3.247402	0.889499	H	0.152025	-3.065163	-0.264957
H	0.102166	-3.108979	-0.270965	H	0.110361	-3.075473	-0.216380	H	-1.256831	-3.285235	0.768623
H	-1.527808	-3.392796	-0.880999	H	-1.507507	-3.384026	-0.848265	H	-1.436100	-3.324376	-0.982395
C	-3.608335	-1.660527	0.445347	C	-3.606663	-1.662272	0.529008	C	-3.586169	-1.663754	0.393721
H	-4.446899	-1.448823	-0.224182	H	-4.464919	-1.449229	-0.114317	H	-4.409681	-1.422485	-0.283591
H	-3.392813	-2.720248	0.359999	H	-3.392246	-2.721139	0.429258	H	-3.368807	-2.718859	0.265190
H	-3.947324	-1.481445	1.470481	H	-3.913306	-1.488912	1.564609	H	-3.945935	-1.529292	1.418093
C	-1.707684	2.923549	-0.233952	C	-1.735373	2.920018	-0.243439	C	-1.670617	2.937591	-0.095525
H	-0.997697	3.406324	0.440164	H	-1.146620	3.406910	0.536952	H	-1.600923	3.365311	-1.098012
H	-1.538451	3.333166	-1.232062	H	-1.418328	3.342676	-1.197890	H	-0.902861	3.416303	0.513185
H	-2.712956	3.188171	0.076613	H	-2.778402	3.174066	-0.085909	H	-2.643768	3.191363	0.312026
C	3.437939	-1.163783	-1.020269	C	3.410233	-1.125179	-1.092861	C	3.463830	-1.062432	-0.969453
H	4.363603	-0.620894	-0.838683	H	4.339354	-0.617274	-0.841909	H	4.415802	-0.566239	-1.070991
H	3.471560	-2.119496	-0.499559	H	3.426037	-2.135203	-0.686874	H	3.401058	-2.138726	-0.984274
H	3.331353	-1.348998	-2.087696	H	3.306135	-1.176847	-2.174969	C	0.934270	1.815375	-0.750779
C	0.847529	1.653473	-0.911861	C	0.851578	1.762531	-0.941685	H	1.204543	2.371202	0.150019
H	0.750934	2.723955	-1.002812	H	0.613291	2.279838	-1.878020	H	0.626767	2.557369	-1.488763
C	2.150584	1.006510	-1.241373	H	0.957052	2.565387	-0.198828	C	2.151946	1.052175	-1.260397
H	2.237982	0.822425	-2.319148	C	2.131894	1.023614	-1.080903	H	3.060861	1.635197	-1.127660
H	2.992420	1.634764	-0.953495	H	3.032897	1.529344	-1.392373	H	2.047347	0.808096	-2.317939
C	2.246467	-0.346751	-0.541926	C	2.221357	-0.356278	-0.527261	C	2.281235	-0.278126	-0.525341
C	2.360689	-0.187276	0.978541	C	2.355493	-0.356236	1.008971	C	2.469066	-0.027944	0.985486

O	1.747163	-0.820232	1.785164	O	1.750140	-1.064721	1.756091	O	3.019572	0.935578	1.441017
O	3.295153	0.723923	1.315943	O	3.290595	0.523358	1.420642	O	2.016335	-1.042641	1.733484
H	3.336354	0.740304	2.280664	H	3.360284	0.433790	2.379823	H	2.210749	-0.821390	2.653419

Structures of trolox and their corresponding species in the SPLET (SPL_step) mechanism at M06-2X/6-311++G(2d,2p) level of theory in gas phase.

**M06-2X/6-311++G(2d,2p)**											
**Trolox**				**Trolox (O1$^-$)**				**Trolox (O2$^-$)**			
C	-2.42589000	0.83929300	-0.04265000	C	-2.417629	0.820232	-0.068396	C	-2.440391	0.835039	-0.023620
C	-1.15910900	1.37560300	0.18403500	C	-1.157273	1.351228	0.174659	C	-1.167402	1.362228	0.183574
C	-0.09148700	0.49975900	0.38968100	C	-0.085838	0.495323	0.431046	C	-0.102048	0.491141	0.436094
C	-0.25813700	-0.88083000	0.36341900	C	-0.258804	-0.885385	0.422426	C	-0.272711	-0.891202	0.415217
C	-1.52326000	-1.41656200	0.10546100	C	-1.513641	-1.429866	0.153602	C	-1.539658	-1.420255	0.146365
C	-2.58808400	-0.54730000	-0.08660800	C	-2.659967	-0.601458	-0.095233	C	-2.602729	-0.552423	-0.056835
O	-3.82203800	-1.10477000	-0.31690900	O	-3.811773	-1.086243	-0.329564	O	-3.855615	-1.107030	-0.290098
H	-4.45326300	-0.41386300	-0.52421400	O	1.156376	1.093899	0.733427	H	-4.374087	-0.481552	-0.797559
O	1.13466100	1.08336500	0.64536900	C	-0.940643	2.844416	0.166331	O	1.088466	1.076733	0.723491
C	-0.92734300	2.86481400	0.19959400	H	-1.464418	3.304713	-0.672340	C	-0.850442	2.830685	0.094712
H	-1.28630900	3.32235400	-0.72248000	H	0.117015	3.083127	0.089263	H	-1.605515	3.377234	-0.465538
H	0.12964000	3.08586600	0.29634800	H	-1.328865	3.306969	1.078806	H	0.108933	2.940661	-0.409253
H	-1.45230900	3.33894700	1.03072800	C	-3.594195	1.721699	-0.327749	H	-0.752579	3.286992	1.083094
C	-3.63608400	1.71323800	-0.25042100	H	-4.498413	1.117068	-0.304930	C	-3.648796	1.716943	-0.226418
H	-4.42348000	1.47334900	0.46910700	H	-3.671450	2.521752	0.413823	H	-4.522620	1.299641	0.277865
H	-3.40162700	2.76518400	-0.12735900	H	-3.533632	2.202411	-1.310618	H	-3.488543	2.709210	0.185735
H	-4.05254500	1.59109500	-1.25474400	C	-1.711972	-2.920645	0.112905	H	-3.899160	1.841870	-1.285243
C	-1.72902800	-2.90596200	0.04324900	H	-0.997892	-3.417422	-0.551728	C	-1.738390	-2.910832	0.067720
H	-0.98351900	-3.37639800	-0.59871100	H	-1.601461	-3.383270	1.100614	H	-0.983604	-3.363761	-0.576333
H	-1.63636600	-3.35848100	1.03305900	H	-2.722650	-3.115650	-0.239946	H	-1.641388	-3.375718	1.051921
H	-2.71535100	-3.14298700	-0.34236000	C	3.488393	1.076112	0.969324	H	-2.722923	-3.149385	-0.323774
C	3.45643400	1.08234100	0.99010400	H	4.407773	0.531006	0.756717	C	3.416354	1.100917	1.029545
H	4.38515900	0.53555600	0.84169300	H	3.523018	2.052778	0.488573	H	4.346282	0.572128	0.829252
H	3.51803100	2.04415500	0.48311500	H	3.392904	1.215214	2.045455	H	3.448396	2.072465	0.536104
H	3.30536700	1.25653600	2.05411300	C	0.915676	-1.805601	0.674910	H	3.301136	1.253894	2.105606
C	0.91509400	-1.80222700	0.59893700	H	0.636123	-2.575325	1.397118	C	0.914857	-1.798743	0.624819
H	0.62262300	-2.61094500	1.26912300	H	1.168688	-2.338841	-0.246675	H	0.638340	-2.636313	1.269393
H	1.20147000	-2.27982300	-0.34393900	C	2.138997	-1.047961	1.178181	H	1.214235	-2.220821	-0.339370
C	2.10663500	-1.05019200	1.17618000	H	2.024057	-0.810246	2.238009	C	2.104126	-1.039807	1.203187
H	1.93362600	-0.81193700	2.22703500	H	3.050672	-1.633016	1.056999	H	1.947647	-0.835842	2.267476
H	3.01955600	-1.63768600	1.10387300	C	2.272178	0.299223	0.470402	H	3.017343	-1.617234	1.079276
C	2.27735000	0.28233500	0.45296100	C	2.449690	0.156731	-1.048753	C	2.261643	0.288001	0.476924
C	2.48268500	0.07311100	-1.05077900	O	2.095908	0.951595	-1.868057	C	2.493130	0.066641	-1.066535
O	1.89262300	0.63983100	-1.92103800	O	3.168461	-0.944792	-1.386424	O	1.812829	0.765306	-1.831551
O	3.46083300	-0.82643000	-1.29265100	H	3.256426	-0.916977	-2.347173	O	3.371443	-0.786468	-1.312923
H	3.56201700	-0.88140500	-2.25153100								
**Trolox (C1$^-$)**				**Trolox (C2$^-$)**				**Trolox (C3$^-$)**			
C	-2.496080	0.870388	-0.018409	C	-2.494437	0.808435	-0.022936	C	-2.381350	0.780747	-0.019089
C	-1.204411	1.373017	0.373956	C	-1.179527	1.336481	0.168769	C	-1.120533	1.379429	0.245288
C	-0.167150	0.375957	0.417715	C	-0.113661	0.476926	0.388271	C	-0.067690	0.504613	0.483392
C	-0.379705	-0.954040	0.159504	C	-0.227041	-0.911667	0.395949	C	-0.184069	-0.879222	0.452829
C	-1.661558	-1.424514	-0.219606	C	-1.505790	-1.455099	0.111383	C	-1.438892	-1.505501	0.133603
C	-2.680734	-0.479384	-0.291711	C	-2.578255	-0.625423	-0.079803	C	-2.517138	-0.583153	-0.085865
O	-3.933153	-0.962795	-0.659417	O	-3.812856	-1.174936	-0.381979	O	-3.747656	-1.167979	-0.364965
H	-4.551551	-0.236937	-0.583218	H	-4.285837	-0.478690	-0.856246	H	-4.341428	-0.482983	-0.672483
O	1.085077	0.879128	0.796312	O	1.121841	1.091773	0.644879	O	1.169223	1.101896	0.774188
C	-0.958144	2.696422	0.690548	C	-1.002972	2.823280	0.147411	C	-0.917122	2.870482	0.219176
H	0.037521	3.019302	0.944992	H	-1.382785	3.236366	-0.793711	H	-1.294173	3.318976	-0.705559
H	-1.739424	3.438901	0.667293	H	0.040148	3.106436	0.257132	H	0.143533	3.100295	0.281612
C	-3.591235	1.889322	-0.116807	H	-1.586693	3.296875	0.945937	H	-1.415071	3.387439	1.048972
H	-3.797283	2.346213	0.859501	C	-3.627423	1.601442	-0.205091	C	-3.601867	1.643186	-0.248070
H	-3.279356	2.710583	-0.772993	H	-4.624527	1.191305	-0.129119	H	-4.437156	1.328191	0.384728
H	-4.539826	1.516607	-0.510233	H	-3.559376	2.677225	-0.202164	H	-3.401261	2.686127	-0.016978
C	-1.904094	-2.879213	-0.516159	C	-1.675790	-2.949911	0.024313	H	-3.945420	1.606773	-1.288604
H	-1.193296	-3.276493	-1.249082	H	-0.920419	-3.394534	-0.628376	C	-1.619093	-2.875523	0.059147
H	-1.821629	-3.517462	0.373570	H	-1.567299	-3.424004	1.004550	H	-0.804782	-3.563469	0.222712
H	-2.903940	-3.018575	-0.920252	H	-2.660445	-3.198810	-0.362287	H	-2.588413	-3.283101	-0.176211
C	3.371313	0.711572	1.261154	C	3.447863	1.115930	0.911959	C	3.509698	1.132174	0.907652
H	4.289888	0.159492	1.065303	H	4.378604	0.578009	0.733477	H	4.430031	0.599568	0.668602
H	3.482018	1.741786	0.925274	H	3.468159	2.067912	0.383300	H	3.505895	2.098817	0.405604
H	3.168760	0.713435	2.331173	H	3.340489	1.306830	1.978989	H	3.456534	1.291416	1.983978
C	0.745662	-1.954670	0.299764	C	0.933678	-1.818602	0.716217	C	0.969564	-1.785087	0.759563
H	0.385461	-2.839076	0.828916	H	0.651639	-2.554755	1.476170	H	0.671339	-2.498354	1.537811

H	1.069020	-2.306225	-0.686055	H	1.242677	-2.402911	-0.159334	H	1.200471	-2.401489	-0.119089
C	1.928448	-1.355207	1.046190	C	2.124714	-1.014226	1.221943	C	2.207574	-1.015942	1.204484
H	1.690743	-1.260630	2.107969	H	1.964862	-0.720460	2.261648	H	2.140070	-0.765806	2.265638
H	2.824922	-1.966074	0.945204	H	3.052442	-1.583347	1.163542	H	3.119920	-1.592486	1.049779
C	2.186018	0.065061	0.546746	C	2.250485	0.293147	0.442408	C	2.292418	0.320543	0.474191
C	2.519738	0.079574	-0.952663	C	2.446161	0.063006	-1.061948	C	2.391701	0.159926	-1.048622
O	2.185791	0.901906	-1.750114	O	2.077916	0.790757	-1.935445	O	2.002939	0.953534	-1.855768
O	3.358730	-0.940442	-1.293086	O	3.203703	-1.035573	-1.320988	O	3.078268	-0.951106	-1.411812
H	3.544993	-0.818835	-2.231712	H	3.299955	-1.065438	-2.280670	H	3.087861	-0.951010	-2.376686
**Trolox ($C4^-$)**				**Trolox ($C5^-$)**				**Trolox ($C6^-$)**			
C	-2.432723	0.840767	-0.079420	C	-2.479187	0.870557	-0.004635	C	-2.319427	1.280408	0.129835
C	-1.150577	1.359943	0.215692	C	-1.180744	1.379681	0.091231	C	-1.481610	1.656700	1.172938
C	-0.122816	0.502110	0.521486	C	-0.092833	0.497603	0.192250	C	-0.427278	0.794617	1.632943
C	-0.245524	-0.924851	0.522836	C	-0.301617	-0.891369	0.228530	C	-0.293628	-0.464482	0.952013
C	-1.543325	-1.429609	0.186615	C	-1.602840	-1.388689	0.117387	C	-1.122114	-0.819386	-0.112313
C	-2.572796	-0.550777	-0.088812	C	-2.671014	-0.507223	0.008318	C	-2.129956	0.055910	-0.517773
O	-3.815640	-1.102980	-0.389054	O	-3.950506	-1.043939	-0.080666	O	-2.987022	-0.309926	-1.576798
H	-4.406307	-0.382149	-0.608816	H	-4.565143	-0.330226	-0.252832	H	-2.708483	0.168616	-2.359556
O	1.121524	1.062054	0.846793	O	1.132624	1.070286	0.247532	O	0.353647	1.120192	2.584863
C	-0.898483	2.847378	0.184885	C	-0.925550	2.867260	0.081211	C	-1.647312	2.980178	1.869978
H	-1.199498	3.272560	-0.775008	H	-1.199542	3.316239	-0.877039	H	-1.783746	3.804228	1.164634
H	0.156848	3.055144	0.331073	H	0.128473	3.060095	0.251959	H	-0.760821	3.163387	2.473953
H	-1.461839	3.368615	0.964039	H	-1.499077	3.377684	0.858048	H	-2.513578	2.985982	2.541230
C	-3.619421	1.720797	-0.373243	C	-3.683783	1.770988	-0.135596	C	-3.435173	2.196830	-0.312358
H	-4.457568	1.544912	0.315159	H	-4.389926	1.626532	0.688515	H	-4.176817	1.653378	-0.893582
H	-3.363541	2.773351	-0.276457	H	-3.403571	2.819996	-0.138646	H	-3.929776	2.647603	0.548572
H	-4.011950	1.592254	-1.392222	H	-4.226728	1.587282	-1.069600	H	-3.058552	3.021348	-0.926332
C	-1.736857	-2.912901	0.141818	C	-1.849423	-2.875975	0.112053	C	-0.942851	-2.138216	-0.827349
H	-1.042941	-3.368929	-0.574947	H	-1.142945	-3.378866	-0.547955	H	0.066111	-2.232623	-1.235378
H	-1.508883	-3.366857	1.113726	H	-1.711069	-3.301145	1.109255	H	-1.092197	-2.979106	-0.146722
H	-2.754188	-3.176000	-0.137029	H	-2.861424	-3.100417	-0.214216	H	-1.655375	-2.232972	-1.641855
C	3.455088	1.085834	1.013871	C	3.323799	1.017439	1.153605	C	2.005615	0.362605	-1.168415
H	4.378802	0.543386	0.815758	H	4.292418	0.528971	1.206908	H	2.085910	0.345980	-2.246901
H	3.486490	2.054641	0.514827	H	3.437167	1.995560	0.684779	H	1.574298	1.228448	-0.683208
H	3.354177	1.248443	2.085977	H	2.930508	1.153481	2.160306	C	0.828518	-1.342246	1.427900
C	0.852530	-1.729415	0.806853	C	0.878463	-1.828935	0.394404	H	0.805246	-1.371293	2.519210
H	0.771048	-2.804502	0.813448	H	0.580956	-2.647787	1.053918	H	0.742106	-2.362866	1.054108
C	2.147654	-1.090804	1.201564	H	1.069141	-2.292246	-0.585295	C	2.213819	-0.763844	1.042130
H	2.255674	-0.900110	2.282645	C	2.108907	-1.159068	0.963624	H	2.255216	0.237298	1.469193
H	3.007650	-1.694799	0.904124	H	2.115263	-1.125316	2.053415	H	3.002156	-1.369503	1.489642
C	2.250867	0.280581	0.533535	C	2.340494	0.153532	0.382883	C	2.398105	-0.683955	-0.443830
C	2.366961	0.139842	-0.983752	C	2.777312	-0.101416	-1.043280	C	2.917418	-1.856177	-1.196636
O	1.788170	0.799742	-1.798618	O	2.063001	-0.442480	-1.956042	O	3.166746	-1.902874	-2.376938
O	3.304724	-0.772305	-1.350437	O	4.120718	0.042310	-1.252017	O	3.098346	-2.947227	-0.413229
H	3.283775	-0.786609	-2.314178	H	4.256698	-0.263779	-2.155393	H	3.405876	-3.643633	-1.004874

Structures of trolox and their corresponding species in the SPLET (ETE_step) mechanism at M06-2X/6-311++G(2d,2p) level of theory in gas phase.

**M06-2X/6-311++G(2d,2p)**											
**Trolox (O1•)**				**Trolox (O2•)**				**Trolox (C1•)**			
C	-2.468348	-0.895127	0.019190	C	-2.233382	-0.840370	0.111308	C	2.472580	-0.879481	-0.065919
C	-1.201121	-1.372741	-0.185739	C	-0.976419	-1.342755	-0.223013	C	1.175795	-1.425587	0.173350
C	-0.136743	-0.450707	-0.343167	C	0.045697	-0.434849	-0.509589	C	0.096325	-0.517202	0.365243
C	-0.312763	0.944218	-0.296679	C	-0.147082	0.942491	-0.454777	C	0.278027	0.852481	0.339136
C	-1.572308	1.445942	-0.066362	C	-1.401366	1.441396	-0.092523	C	1.556801	1.373899	0.099345
C	-2.705834	0.545426	0.092530	C	-2.425244	0.542226	0.176629	C	2.633936	0.494569	-0.083730
O	-3.849232	0.997294	0.283744	O	-3.647740	1.065911	0.513687	O	3.855642	1.088978	-0.281464
O	1.071957	-1.022015	-0.567718	H	-4.248417	0.357003	0.749494	H	4.540081	0.419794	-0.290939
C	-0.864366	-2.837785	-0.226184	O	1.274930	-0.987167	-0.812300	O	-1.120454	-1.101138	0.602612
H	-1.734637	-3.450072	-0.013606	C	-0.708183	-2.825401	-0.261565	C	0.941828	-2.798752	0.225966
H	-0.089883	-3.062134	0.507264	H	-0.960122	-3.289008	0.692584	H	-0.054114	-3.173948	0.387615
H	-0.468732	-3.120656	-1.202334	H	0.338460	-3.023045	-0.464200	H	1.739293	-3.512853	0.106131
C	-3.653722	-1.798127	0.196707	H	-1.303127	-3.313047	-1.035253	C	3.616404	-1.827970	-0.301623
H	-4.559800	-1.202805	0.249171	C	-3.399724	-1.745558	0.415320	H	3.873283	-2.377138	0.606556

H	-3.739072	-2.504752	-0.628972
H	-3.567768	-2.378614	1.117157
C	-1.826171	2.919690	0.014122
H	-1.109960	3.407133	0.676948
H	-1.728644	3.387514	-0.968952
H	-2.834025	3.098533	0.375489
C	3.378729	-1.031914	-1.025179
H	4.316916	-0.493104	-0.915395
H	3.456224	-1.996251	-0.525437
H	3.180036	-1.200589	-2.082099
C	0.869288	1.860883	-0.500702
H	0.577714	2.705284	-1.124381
H	1.176311	2.283500	0.461810
C	2.038047	1.116746	-1.133284
H	1.830717	0.898036	-2.182331
H	2.955918	1.698463	-1.080371
C	2.236015	-0.222714	-0.431671
C	2.483080	-0.010827	1.064300
O	1.824429	-0.469563	1.948367
O	3.561204	0.770066	1.271470
H	3.673549	0.856781	2.227066

H	-4.254488	-1.516019	-0.226462
H	-3.154329	-2.790198	0.256078
H	-3.725892	-1.645806	1.454795
C	-1.636356	2.924119	0.009588
H	-0.841462	3.403039	0.581748
H	-1.652255	3.388571	-0.978725
H	-2.585084	3.132660	0.493429
C	3.616488	-0.930020	-1.131453
H	4.487092	-0.295328	-0.975684
H	3.679256	-1.788717	-0.463706
H	3.653698	-1.305622	-2.162405
C	0.987222	1.889109	-0.757446
H	0.622324	2.722050	-1.359309
H	1.356958	2.312247	0.179871
C	2.125716	1.174369	-1.481291
H	1.858648	1.043400	-2.540569
H	3.040899	1.763360	-1.450052
C	2.367690	-0.165856	-0.877995
C	1.580127	-0.343290	2.199468
O	1.570219	-1.495222	2.113173
O	1.595215	0.807063	2.315972

H	3.347891	-2.562353	-1.061328
H	4.522608	-1.342427	-0.659866
C	1.776025	2.860197	0.053726
H	1.009207	3.349652	-0.547279
H	1.728585	3.295900	1.055268
H	2.748059	3.097034	-0.365743
C	-3.429866	-1.094634	1.027946
H	-4.360681	-0.542591	0.918741
H	-3.513174	-2.049313	0.510741
H	-3.243033	-1.284144	2.083577
C	-0.887326	1.781545	0.583484
H	-0.581900	2.594283	1.242385
H	-1.191682	2.250799	-0.357905
C	-2.065795	1.032668	1.191819
H	-1.862012	0.786515	2.235386
H	-2.977411	1.625055	1.150899
C	-2.267749	-0.291512	0.461221
C	-2.513561	-0.052470	-1.031639
O	-1.910346	-0.557766	-1.929358
O	-3.537497	0.806882	-1.224801
H	-3.657685	0.892862	-2.179196

Trolox (C2$^{\bullet}$)

C	-2.473176	-0.899601	0.040214
C	-1.159716	-1.417632	-0.159214
C	-0.118368	-0.534852	-0.370934
C	-0.307804	0.854060	-0.378264
C	-1.580283	1.382522	-0.117790
C	-2.636075	0.516884	0.092088
O	-3.862661	1.061809	0.341495
H	-4.423586	0.403925	0.757378
O	1.119818	-1.094262	-0.613468
C	-0.926009	-2.900259	-0.154271
H	-1.294890	-3.342143	0.772671
H	0.130924	-3.124217	-0.243980
H	-1.453456	-3.382853	-0.979830
C	-3.570637	-1.753201	0.171892
H	-4.587535	-1.393846	0.216001
H	-3.448677	-2.823143	0.167524
C	-1.791277	2.871876	-0.066986
H	-1.040465	3.351536	0.561465
H	-1.712569	3.314189	-1.062315
H	-2.774181	3.107608	0.327548
C	3.442071	-1.063355	-0.948857
H	4.361212	-0.497665	-0.812664
H	3.518128	-2.010969	-0.417830
H	3.296189	-1.266856	-2.008355
C	0.850708	1.784697	-0.635246
H	0.545395	2.574430	-1.322542
H	1.127414	2.289261	0.297239
C	2.057153	1.040673	-1.191868
H	1.891967	0.774151	-2.237113
H	2.958107	1.647607	-1.131141
C	2.248668	-0.269794	-0.434931
C	2.441559	-0.014742	1.062937
O	1.836252	-0.546404	1.944634
O	3.421676	0.887498	1.284197
H	3.512412	0.977206	2.241595

Trolox (C3$^{\bullet}$)

C	-2.435292	-0.808525	0.001849
C	-1.156330	-1.334896	-0.208706
C	-0.083448	-0.444656	-0.403757
C	-0.242154	0.924460	-0.380797
C	-1.532648	1.476054	-0.119829
C	-2.608344	0.566766	0.055155
O	-3.832589	1.121847	0.283269
H	-4.477553	0.430657	0.445744
O	1.141722	-1.035669	-0.637746
C	-0.912710	-2.819295	-0.215963
H	-1.306739	-3.280520	0.690137
H	0.148717	-3.032761	-0.269987
H	-1.401118	-3.297582	-1.067682
C	-3.640683	-1.694720	0.188539
H	-4.431892	-1.437633	-0.520705
H	-3.401685	-2.740733	0.029264
H	-4.052636	-1.606300	1.197838
C	-1.759030	2.848487	-0.028997
H	-0.959565	3.561946	-0.141838
H	-2.748727	3.223340	0.170983
C	3.475862	-1.046758	-0.898133
H	4.401041	-0.504390	-0.714807
H	3.513742	-2.008703	-0.389119
H	3.364288	-1.219472	-1.967252
C	0.926910	1.846433	-0.611959
H	0.645348	2.623744	-1.323441
H	1.177646	2.361865	0.320995
C	2.141791	1.090700	-1.133868
H	2.014172	0.850875	-2.190706
H	3.050880	1.678170	-1.023892
C	2.282331	-0.240696	-0.404254
C	2.433544	-0.039720	1.107219
O	1.834359	-0.635237	1.951734
O	3.378809	0.882114	1.388766
H	3.449202	0.928929	2.350890

Trolox (C4$^{\bullet}$)

C	-2.357878	-0.850666	0.019568
C	-1.084422	-1.360866	-0.259855
C	-0.052527	-0.466392	-0.488930
C	-0.237521	0.939285	-0.437477
C	-1.530104	1.442156	-0.121278
C	-2.551114	0.540852	0.092311
O	-3.792735	1.050613	0.382175
H	-4.397696	0.334287	0.580371
O	1.182689	-0.981210	-0.806326
C	-0.819694	-2.843247	-0.294997
H	-1.134485	-3.313679	0.636502
H	0.239094	-3.035553	-0.428859
H	-1.361062	-3.324616	-1.111101
C	-3.537068	-1.753918	0.262074
H	-4.360450	-1.521128	-0.419192
H	-3.284595	-2.798023	0.110964
H	-3.913995	-1.657628	1.285081
C	-1.765784	2.921920	-0.032218
H	-1.093339	3.376994	0.697381
H	-1.578675	3.405983	-0.993112
H	-2.788365	3.133490	0.262077
C	3.531801	-0.942553	-0.928741
H	4.433304	-0.386439	-0.678580
H	3.579761	-1.931350	-0.475275
H	3.464932	-1.054691	-2.009490
C	0.849229	1.780597	-0.711034
H	0.720858	2.851423	-0.730381
C	2.182169	1.198083	-1.041247
H	2.308849	1.093154	-2.125718
H	2.993887	1.829562	-0.682625
C	2.300124	-0.197611	-0.434775
C	2.361706	-0.142371	1.096068
O	1.744167	-0.848810	1.835848
O	3.255153	0.770561	1.526950
H	3.265664	0.720193	2.491317

Trolox (C5$^{\bullet}$)

C	-2.399922	-0.861707	0.204756
C	-1.159303	-1.384806	-0.158559
C	-0.152158	-0.498132	-0.539990
C	-0.344195	0.878189	-0.540763
C	-1.579413	1.404515	-0.154405
C	-2.588490	0.522737	0.210681
O	-3.792029	1.066402	0.585513
H	-4.391721	0.366434	0.848403
O	1.048011	-1.049091	-0.947441
C	-0.887143	-2.865686	-0.128375
H	-1.146611	-3.285760	0.843128
H	0.164095	-3.060563	-0.309463
H	-1.469636	-3.392815	-0.886073
C	-3.550927	-1.747479	0.608185
H	-4.438733	-1.539884	0.004402
H	-3.318023	-2.798892	0.476200

Trolox (C6$^{\bullet}$)

C	-2.684472	0.494508	0.217919
C	-2.544369	-0.862136	0.052826
C	-1.290329	-1.400580	-0.462905
C	-0.216110	-0.478683	-0.825376
C	-0.371360	0.871300	-0.638591
C	-1.604239	1.337213	-0.120754
O	-1.698881	2.674029	0.044979
H	-2.562040	2.907565	0.396781
O	-1.128194	-2.628867	-0.584727
C	-3.634023	-1.837196	0.388028
H	-3.892620	-1.790878	1.447377
H	-3.302869	-2.844200	0.155478
H	-4.540930	-1.624741	-0.180171
C	-3.942514	1.125526	0.756609
H	-4.382208	1.820153	0.035689
H	-4.695434	0.377781	0.981890

H	-3.819333	-1.603386	1.658779	H	-3.748229	1.676006	1.681405
C	-1.812221	2.890784	-0.139809	C	0.686620	1.904577	-0.918783
H	-1.203697	3.375295	0.626750	H	1.055740	2.331956	0.015064
H	-1.542846	3.339462	-1.097030	H	1.532359	1.485142	-1.454068
H	-2.853484	3.117828	0.063908	H	0.274811	2.723908	-1.506360
C	3.383196	-1.018742	-1.274062	C	1.850704	-0.260005	1.951973
H	4.310103	-0.494620	-1.049663	H	2.196419	0.575074	2.545433
H	3.437565	-2.035847	-0.889663	H	1.071481	-0.895357	2.351613
H	3.237231	-1.051730	-2.352057	C	1.046777	-1.117955	-1.321179
C	0.769962	1.806622	-0.962338	H	0.780410	-1.987795	-1.918319
H	0.483458	2.337395	-1.877373	H	1.621079	-0.439566	-1.946210
H	0.887348	2.596330	-0.207268	C	1.925785	-1.612630	-0.148991
C	2.059508	1.099803	-1.167352	H	1.346356	-2.330853	0.429837
H	2.936109	1.631908	-1.503537	H	2.791239	-2.131365	-0.560748
C	2.201099	-0.288434	-0.646527	C	2.375642	-0.499813	0.754896
C	2.396052	-0.315949	0.883019	C	3.438642	0.441857	0.289398
O	1.836624	-1.052836	1.638379	O	3.830077	1.410273	0.882906
O	3.327133	0.576446	1.275892	O	3.941407	0.097124	-0.916551
H	3.436897	0.469364	2.229513	H	4.620876	0.749480	-1.128551
